# Supplementary material for: The impact of pulmonary rehabilitation on sleep quality in patients with chronic obstructive pulmonary disease: A systematic review and meta-analysis
Source: PLoS One. 2025 Jun 4;20(6):e0318424. doi: 10.1371/journal.pone.0318424 (PMC12136455; doi:10.1371/journal.pone.0318424)
Supplement: S1 File — (DOCX) [file pone.0318424.s001.docx]

GRADE evidence profile

**Question:** Pulmonary rehabilitation compared to no pulmonary rehabilitation for sleep quality in patients with chronic obstructive pulmonary disease

**Setting:** PR has a beneficial effect on sleep quality in COPD patients as defined by the PSQI.

**Bibliography:**

| **Certainty assessment** | | | | | | | **№ of patients** | | **Effect** | | **Certainty** | **Importance** |
| --- | --- | --- | --- | --- | --- | --- | --- | --- | --- | --- | --- | --- |
| **№ of studies** | **Study design** | **Risk of bias** | **Inconsistency** | **Indirectness** | **Imprecision** | **Other considerations** | **pulmonary rehabilitation** | **no pulmonary rehabilitation** | **Relative (95% CI)** | **Absolute (95% CI)** |  |  |
| **PR has a beneficial effect on sleep quality in COPD patients as defined by the PSQI.** | | | | | | | | | | | | |
| 3 | randomised trials | not serious | not serious | not serious | not serious | none | 203 | 202 | - | MD **3.95 higher** (0.36 higher to 7.55 higher) | ⨁⨁⨁⨁ High | CRITICAL |
| **PR has a beneficial effect on sleep quality in COPD patients as defined by the PSQI.** | | | | | | | | | | | | |
| 4 | non-randomised studies | not serious | serious^a^ | not serious | not serious | none | 169/- |  | not estimable |  | ⨁◯◯◯ Very low^a^ | IMPORTANT |

**CI:** confidence interval; **MD:** mean difference

#### Explanations

a. There is no consistent definition of for PR in terms of time, activities, or periodicity of implementation.
