# Supplementary material for: The impact of pulmonary rehabilitation on sleep quality in patients with chronic obstructive pulmonary disease: A systematic review and meta-analysis
Source: PLoS One. 2025 Jun 4;20(6):e0318424. doi: 10.1371/journal.pone.0318424 (PMC12136455; doi:10.1371/journal.pone.0318424)
Supplement: S2 File — (DOC) [file pone.0318424.s002.doc]

Database: Embase <1974 to 2023 February 23>, Ovid MEDLINE(R) ALL <1946 to February 23, 2023>. Search date: 23 February 2023

Search Strategy:

--------------------------------------------------------------------------------

1 pulmonary rehabilitat*.mp. [mp=ti, ab, hw, tn, ot, dm, mf, dv, kf, fx, dq, bt, nm, ox, px, rx, an, ui, sy, ux, mx] (16419)

2 sleep.mp. [mp=ti, ab, hw, tn, ot, dm, mf, dv, kf, fx, dq, bt, nm, ox, px, rx, an, ui, sy, ux, mx] (629421)

3 (chronic obstructive pulmonary disease or COPD or chronic bronchitis or emphysema).mp. [mp=ti, ab, hw, tn, ot, dm, mf, dv, kf, fx, dq, bt, nm, ox, px, rx, an, ui, sy, ux, mx] (306980)

4 1 and 2 and 3 (324)

5 remove duplicates from 4 (258)

Database: Cochrane Central Register of Controlled Trials (CENTRAL). Search date: 3 March 2023

Search Strategy:

--------------------------------------------------------------------------------

90 Trials matching (pulmonary rehabilitat*) AND (sleep) AND (chronic obstructive pulmonary disease OR COPD OR chronic bronchitis OR emphysema) in All Text

Cochrane Central Register of Controlled Trials

Issue 2 of 12, February 2023

1.

STATE-OF-THE-ART REVIEW ON IMPROVING TREATMENT ADHERENCE IN COPD BY TARGETING CONCURRENT PSYCHOLOGICAL SYMPTOMS.

Moales E.-A., Cojocaru D.-C., Szalontay A.S., Ghiciuc C.M., Gaina M.-A., Boisteanu D., Fatu A.M., Vascu M.B., Cozma S., Robu S., Mitu F., Dima-Cozma L.C.

Embase

Farmacia. 70(6) (pp 1018-1027), 2022. Date of Publication: 2022.

[Review]

AN: 2018959562

Chronic obstructive pulmonary disease (COPD), a condition with significant global impact, is characterised by progressive limitation of airflow, impaired lung structure and persistent respiratory symptoms. Anxiety and depression are the most common mental disorders in COPD patients and are associated with lesser treatment adherence. We conducted a literature search on PubMed, Cochrane Central Register of Controlled Trials, Web of Science and Scopus, up to July 1, 2022, regarding COPD anxiety and depression concurrence, an underlying mechanism such as hypoxemia, smoking, systemic inflammation and finally regarding pharmacological and alternative interventions that can raise treatment adherence of COPD patients through improving psychological outcomes. Acute dyspnoea, low body mass index, female gender, poor lung function, poor performance and regular smoking are risk factors for the association between depression and COPD. Pulmonary rehabilitation and telerehabilitation increase exercise tolerance and improve the mental status and patient-related outcomes. Psychotherapy and respiratory recovery significantly alleviate symptoms and increase the quality of life. Although more research is needed, immersive virtual reality facilitated interventions reveal promising results. Improving COPD patients' treatment adherence should target concurrent psychological symptoms such as anxiety and depression.

Copyright © 2022, Romanian Society for Pharmaceutical Sciences. All rights reserved.

Status

Embase

Institution

(Moales, Cojocaru, Szalontay, Ghiciuc, Gaina, Boisteanu, Fatu, Vascu, Cozma, Mitu, Dima-Cozma) "Grigore T. Popa" University of Medicine and Pharmacy Iasi, Romania (Cojocaru, Mitu, Dima-Cozma) Faculty of Medicine, Department of Medical Specialties I, Galati, Romania

(Szalontay, Gaina, Boisteanu) Faculty of Medicine, Department of Medical Specialties III, Galati, Romania

(Ghiciuc) Faculty of Medicine, Department of Morpho-Functional Sciences II, Galati, Romania

(Fatu) Faculty of Dentistry, Department of implantology, Removable Prosthesis, Dental Prosthesis Technology, Galati, Romania

(Vascu) Faculty of Dentistry, Department of Odontology, Periodontology, Fixed Prosthesis, Galati, Romania

(Cozma) Faculty of Medicine, Department of Surgery II, Galati, Romania

(Robu) "Dunarea de Jos" University,, Faculty of Medicine and Pharmacy, Department of Pharmaceutical Sciences, Galati, Romania

Publisher

Romanian Society for Pharmaceutical Sciences

Year of Publication

2022

Link to the Ovid Full Text or citation:

[Click here for full text options](https://ovidsp.ovid.com/ovidweb.cgi?T=JS&CSC=Y&NEWS=N&PAGE=fulltext&D=emexb&AN=2018959562)

Link to the External Link Resolver:

[LibKey NHS](https://libkey.io/libraries/2789/openurl?genre=article&sid=OVID:emexb&genre=article&id=pmid:&id=doi:10.31925%2Ffarmacia.2022.6.3&issn=0014-8237&volume=70&issue=6&spage=1018&pages=1018-1027&date=2022&title=Farmacia&atitle=STATE-OF-THE-ART+REVIEW+ON+IMPROVING+TREATMENT+ADHERENCE+IN+COPD+BY+TARGETING+CONCURRENT+PSYCHOLOGICAL+SYMPTOMS&aulast=Moales)

2.

Promoting Chronic Obstructive Pulmonary Disease Wellness through Remote Monitoring and Health Coaching A Clinical Trial.

Benzo R., Hoult J., McEvoy C., Clark M., Benzo M., Johnson M., Novotny P.

Embase

Annals of the American Thoracic Society. 19(11) (pp 1808-1817), 2022. Date of Publication: November 2022.

[Article]

AN: 2022011111

Rationale: Quality of life (QoL) matters the most to patients with chronic obstructive pulmonary disease (COPD) and is associated with healthcare usage and survival. Pulmonary rehabilitation is the most effective intervention in improving QoL but has low uptake and adherence. Home-based programs are a proposed solution. However, there is a knowledge gap on effective and sustainable home-based programs impacting QoL in patients with COPD.

Objective(s): To determine whether remote patient monitoring with health coaching improves the physical and emotional disease-specific QoL measured by the Chronic Respiratory Questionnaire (CRQ).

Method(s): This multicenter clinical trial enrolled 375 adult patients with COPD, randomized to a 12-week remote patient monitoring with health coaching (n = 188) or wait-list usual care (n = 187). Primary outcomes include physical and emotional QoL measured by the CRQ summary scores. Prespecified secondary outcomes included the CRQ domains: dyspnea, CRQ-fatigue, CRQ-emotions, CRQ-mastery, daily physical activity, self-management abilities, symptoms of depression/anxiety, emergency room/hospital admissions, and sleep.

Result(s): Participant age: 69 6 9 years; 59% women; forced expiratory volume in 1 second percent predicted: 45 6 19. At 12 weeks, there was a significant and clinically meaningful difference between the intervention versus the control group in the physical and emotional CRQ summary scores: change difference (95% confidence interval): 0.54 points (0.36-0.73), P, 0.001; 0.51 (0.39-0.69), P, 0.001, respectively. In addition, all CRQ domains, self-management, daily physical activity, sleep, and depression scores improved (P, 0.01). CRQ changes were maintained at 24 weeks.

Conclusion(s): Remote monitoring with health coaching promotes COPD wellness and behavior change, given its effect on all aspects of QoL, self-management, daily physical activity, sleep, and depression scores. It represents an effective option for home-based rehabilitation. Clinical trial registered with clinicaltrials.gov (NCT 03480386).

Copyright © 2022 by the American Thoracic Society.

PMID

35914215 [<https://www.ncbi.nlm.nih.gov/pubmed/?term=35914215>]

Status

Embase

Institution

(Benzo, Hoult, Benzo) Mindful Breathing Laboratory, Division of Pulmonary, Critical Care, and Sleep Medicine, Saint Paul, MN, United States (Clark) Department of Psychology, Mayo Clinic, Jacksonville, FL, United States

(Novotny) Clinical Trials and Biostatistics, Mayo Clinic, Rochester, MN, United States

(McEvoy) Health Partners Research Foundation, Saint Paul, MN, United States

(Johnson) Division of Pulmonary, Critical Care and Sleep Medicine, Mayo Clinic, Jacksonville, FL, United States

Publisher

American Thoracic Society

Clinical Trial Number

<https://clinicaltrials.gov/show/NCT03480386>

Year of Publication

2022

Link to the Ovid Full Text or citation:

[Click here for full text options](https://ovidsp.ovid.com/ovidweb.cgi?T=JS&CSC=Y&NEWS=N&PAGE=fulltext&D=emexb&AN=2022011111)

Link to the External Link Resolver:

[LibKey NHS](https://libkey.io/libraries/2789/openurl?genre=article&sid=OVID:emexb&genre=article&id=pmid:35914215&id=doi:10.1513%2FAnnalsATS.202203-214OC&issn=2329-6933&volume=19&issue=11&spage=1808&pages=1808-1817&date=2022&title=Annals+of+the+American+Thoracic+Society&atitle=Promoting+Chronic+Obstructive+Pulmonary+Disease+Wellness+through+Remote+Monitoring+and+Health+Coaching+A+Clinical+Trial&aulast=Benzo)

3.

Greek Guidelines for the Management of COPD, a Proposal of a Holistic Approach Based on the needs of the Greek Community.

Tzanakis N., Kosmas E., Papaioannou A.I., Hillas G., Zervas E., Loukides S., Bakakos P., Katsaounou P., Boutou A., Perlikos P., Rovina N., Dimakou K., Steiropoulos P., Stratakos G., Emmanouil P., Tryfon S., Koulouris N.

Embase

Journal of Personalized Medicine. 12(12) (no pagination), 2022. Article Number: 1997. Date of Publication: December 2022.

[Article]

AN: 2020730656

Despite that COPD remains one of the most common respiratory diseases worldwide, it can be managed effectively with certain treatments and, more importantly, be prevented by the early implementation of various measures. The pathology and pathophysiology of this disease continue to be studied, with new pharmacological and invasive therapies emerging. In this consensus paper, the Working Group of the Hellenic Thoracic Society aimed to consolidate the up-to-date information and new advances in the treatment of COPD. Local and international data on its prevalence are presented, with revised strategies on the diagnostic approach and the evaluation of risk assessment and disease severity classification. Emphasis is placed on the management and therapy of patients with COPD, covering both common principles, specialized modalities, and algorithms to distinguish between home care and the need for hospitalization. Although pharmacological treatment is commonly recognized in COPD, an integrative approach of pulmonary rehabilitation, physical activity, patient education, and self-assessment should be encountered for a comprehensive treatment, prevention of exacerbations, and increased quality of life in patients.

Copyright © 2022 by the authors.

Status

Embase

Author NameID

Loukides, Stelios; ORCID: <https://orcid.org/0000-0002-4278-9922> Bakakos, Petros; ORCID: <https://orcid.org/0000-0001-9256-9656>

Rovina, Nikolleta; ORCID: <https://orcid.org/0000-0003-0138-5582>

Stratakos, Grigorios; ORCID: <https://orcid.org/0000-0002-5861-3454>

Koulouris, Nikolaos; ORCID: <https://orcid.org/0000-0002-1719-7220>

Katsaounou, Paraskevi; ORCID: <https://orcid.org/0000-0002-8736-619X>

Perlikos, Photis; ORCID: <https://orcid.org/0000-0002-2579-6522>

Boutou, Afroditi; ORCID: <https://orcid.org/0000-0001-7366-2038>

Tryfon, Stavros; ORCID: <https://orcid.org/0000-0001-5102-0480>

Steiropoulos, Paschalis; ORCID: <https://orcid.org/0000-0001-7121-6253>

Institution

(Tzanakis) Department of Thoracic Medicine, University Hospital of Heraklion, Medical School, University of Crete, Heraklion 71303, Greece (Kosmas) Department of Pulmonary Medicine PNOH, Metropolitan Hospital, Neo Faliro, Athens 18547, Greece

(Papaioannou, Loukides) 2nd Respiratory Medicine Department, "Attikon" University Hospital, Athens 15772, Greece

(Hillas, Dimakou) 5th Pulmonary Department, "Sotiria" Chest Diseases Hospital, Athens 15772, Greece

(Zervas) 7th Pulmonary Department, "Sotiria" Chest Diseases Hospital, Athens 15772, Greece

(Bakakos, Rovina, Stratakos, Emmanouil, Koulouris) 1st University Department of Respiratory Medicine, National and Kapodistrian University of Athens, Athens 15772, Greece

(Katsaounou, Perlikos) Department of Respiratory Medicine, Evangelismos General Hospital, Athens 15772, Greece

(Boutou, Tryfon) Department of Respiratory Medicine, G. Papanikolaou Hospital, Thessaloniki 54642, Greece

(Steiropoulos) Department of Respiratory Medicine, Medical School, Democritus University of Thrace, Alexandroupolis 68100, Greece

Publisher

MDPI

Year of Publication

2022

Link to the Ovid Full Text or citation:

[Click here for full text options](https://ovidsp.ovid.com/ovidweb.cgi?T=JS&CSC=Y&NEWS=N&PAGE=fulltext&D=emexb&AN=2020730656)

Link to the External Link Resolver:

[LibKey NHS](https://libkey.io/libraries/2789/openurl?genre=article&sid=OVID:emexb&genre=article&id=pmid:&id=doi:10.3390%2Fjpm12121997&issn=2075-4426&volume=12&issue=12&spage=1997&pages=&date=2022&title=Journal+of+Personalized+Medicine&atitle=Greek+Guidelines+for+the+Management+of+COPD%2C+a+Proposal+of+a+Holistic+Approach+Based+on+the+needs+of+the+Greek+Community&aulast=Tzanakis)

4.

2022 Brazilian Thoracic Association recommendations for long-term home oxygen therapy. Recomendacoes para oxigenoterapia domiciliar prolongada da Sociedade Brasileira de Pneumologia e Tisiologia (2022) <Recomendacoes para oxigenoterapia domiciliar prolongada da Sociedade Brasileira de Pneumologia e Tisiologia (2022).>

de Oliveira Castellano M.V.C., Pereira L.F.F., Feitosa P.H.R., Knorst M.M., Salim C., Rodrigues M.M., Ferreira E.V.M., de Menezes Duarte R.L., Togeiro S.M., Stanzani L.Z.L., Junior P.M., de Melo Schelini K.N., Coelho L.S., de Sousa T.L.F., de Almeida M.B., Alvarez A.E.

Embase

Jornal Brasileiro de Pneumologia. 48(5) (no pagination), 2022. Article Number: e20220179. Date of Publication: 2022.

[Article]

AN: 2018864811

Some chronic respiratory diseases can cause hypoxemia and, in such cases, long-term home oxygen therapy (LTOT) is indicated as a treatment option primarily to improve patient quality of life and life expectancy. Home oxygen has been used for more than 70 years, and support for LTOT is based on two studies from the 1980s that demonstrated that oxygen use improves survival in patients with COPD. There is evidence that LTOT has other beneficial effects such as improved cognitive function, improved exercise capacity, and reduced hospitalizations. LTOT is indicated in other respiratory diseases that cause hypoxemia, on the basis of the same criteria as those used for COPD. There has been an increase in the use of LTOT, probably because of increased life expectancy and a higher prevalence of chronic respiratory diseases, as well as greater availability of LTOT in the health care system. The first Brazilian Thoracic Association consensus statement on LTOT was published in 2000. Twenty-two years later, we present this updated version. This document is a nonsystematic review of the literature, conducted by pulmonologists who evaluated scientific evidence and international guidelines on LTOT in the various diseases that cause hypoxemia and in specific situations (i.e., exercise, sleep, and air travel). These recommendations, produced with a view to clinical practice, contain several charts with information on indications for LTOT, oxygen sources, accessories, strategies for improved efficiency and effectiveness, and recommendations for the safe use of LTOT, as well as a LTOT prescribing model.

Copyright © 2022 Sociedade Brasileira de Pneumologia e Tisiologia.

PMID

36350954 [<https://www.ncbi.nlm.nih.gov/pubmed/?term=36350954>]

Status

Embase

Institution

(de Oliveira Castellano, Rodrigues) Hospital do Servidor Publico Estadual de Sao Paulo - IAMSPE, SP, Sao Paulo, Brazil (Pereira) Hospital das Clinicas, Universidade Federal de Minas Gerais, UFMG, MG, Belo Horizonte, Brazil

(Feitosa, Stanzani) Hospital Regional da Asa Norte, DF, Brasilia, Brazil

(Knorst) Faculdade de Medicina, Universidade Federal do Rio Grande do Sul, UFRGS, RS, Porto Alegre, Brazil

(Knorst) Hospital de Clinicas de Porto Alegre - HCPA, RS, Porto Alegre, Brazil

(Salim, Junior) AC Camargo Cancer Center, SP, Sao Paulo, Brazil

(Salim) Hospital da Policia Militar de Sao Paulo, SP, Sao Paulo, Brazil

(Ferreira) Escola Paulista de Medicina, Universidade Federal de Sao Paulo - Unifesp, SP, Sao Paulo, Brazil

(de Menezes Duarte) Instituto de Doencas do Torax, Universidade Federal do Rio de Janeiro - UFRJ, RJ, Rio de Janeiro, Brazil

(Togeiro) Disciplina de Clinica Medica e Medicina Laboratorial, Universidade Federal de Sao Paulo - Unifesp, SP, Sao Paulo, Brazil

(de Melo Schelini) Hospital Universitario Julio Muller, Universidade Federal de Mato Grosso - UFMT, MT, Cuiaba, Brazil

(Coelho) Universidade Estadual Julio de Mesquita Filho - UNESP, SP, Botucatu, Brazil

(de Sousa) Hospital Universitario Alcides Carneiro, Universidade Federal de Campina Grande - HUAC/UFCG, PB, Campina Grande, Brazil

(de Almeida) Instituto da Crianca, Universidade de Sao Paulo - USP, SP, Sao Paulo, Brazil

(Alvarez) Departamento de Pneumologia, Sociedade de Pediatria de Sao Paulo - SPSP, SP, Campinas, Brazil

Publisher

Sociedade Brasileira de Pneumologia e Tisiologia

Year of Publication

2022

Link to the Ovid Full Text or citation:

[Click here for full text options](https://ovidsp.ovid.com/ovidweb.cgi?T=JS&CSC=Y&NEWS=N&PAGE=fulltext&D=emexb&AN=2018864811)

Link to the External Link Resolver:

[LibKey NHS](https://libkey.io/libraries/2789/openurl?genre=article&sid=OVID:emexb&genre=article&id=pmid:36350954&id=doi:10.36416%2F1806-3756%2Fe20220179&issn=1806-3713&volume=48&issue=5&spage=e20220179&pages=&date=2022&title=Jornal+Brasileiro+de+Pneumologia&atitle=Recomendacoes+para+oxigenoterapia+domiciliar+prolongada+da+Sociedade+Brasileira+de+Pneumologia+e+Tisiologia+(2022)&aulast=de+Oliveira+Castellano)

5.

Sleep Quality and Self-Reported Symptoms of Anxiety and Depression Are Associated with Physical Activity in Patients with Severe COPD.

Neale C.D., Christensen P.E., Dall C., Ulrik C.S., Godtfredsen N., Hansen H.

Embase

International Journal of Environmental Research and Public Health. 19(24) (no pagination), 2022. Article Number: 16804. Date of Publication: December 2022.

[Article]

AN: 2020821031

Sleep quantity, quality and symptoms of depression or anxiety potentially affect the level of daily physical activity (PAL) and plausibly counteracts benefits from pulmonary rehabilitation programs. Their collective impact on PAL is sparsely investigated, particularly in patients with severely progressed chronic obstructive pulmonary disease (COPD).

Aim(s): To investigate if sleep quantity, quality and symptoms from self-reported hospital anxiety and depression scores (HADS) are associated with PAL.

Method(s): In this exploratory cross-sectional study data were analysed from 148 participants with COPD; GOLD grade II-IV; GOLD group B to D (52% female, mean 69.7 +/- SD of 8.4 years, FEV1% predicted 33.6 +/- 10.9, 6MWD 327 +/- 122 m, CAT 20 +/- 7 points), eligible for conventional outpatient hospital-based pulmonary rehabilitation. Participants had sleep and PAL measured 24 h per day for five consecutive days with an activPAL monitor. Adjusted negative binomial regression was applied to investigate the associations with PAL.

Result(s): Participants walked median (25th, 75th percentile) of 2358 (1325.75; 3822.25) steps per day and 14% walked >5000 steps per day on average. Time in bed (TIB) were a median (25th, 75th percentile) of 8.3 (7.1; 9.7) hours and numbers of nocturnal sleeping bouts (NSB) were 1.5 (0.8; 3), Anxiety (HADS-A) and depression (HADS-D) scores were median (25th, 75th percentile) of 5 (3; 8) points and 3 (2; 6) points, respectively, whereof 29% (HADS-A) and 15% (HADS-D) reported scores >=8 points indicating significant symptoms. The fully adjusted rate ratio (RR) for steps per day for TIB (hours) [RR 0.97 (95% CI: 0.92; 1.02)], NSB (numbers) [RR 1.02 (95% CI: 0.97; 1.07)] were not significantly associated with number of steps per day, while there was a significantly association with number of steps per day for HADS-A [RR 1.04 (95% CI: 1.01; 1.07)] and HADS-D [RR 0.95 (95% CI: 0.91; 0.99)].

Conclusion(s): This exploratory cross-sectional study found a statistically significant association between HADS-A and HADS-D with numbers of steps per day in patients with severe COPD.

Copyright © 2022 by the authors.

PMID

36554684 [<https://www.ncbi.nlm.nih.gov/pubmed/?term=36554684>]

Status

In-Process

Author NameID

Hansen, Henrik; ORCID: <https://orcid.org/0000-0001-5464-4088>

Institution

(Neale, Dall) Department of Physical and Occupational Therapy, Copenhagen University Hospital, Copenhagen 2400, Denmark (Christensen) Department of Quality, Copenhagen University Hospital, Hillerod 3400, Denmark

(Dall, Ulrik, Godtfredsen) Institute for Clinical Medicine, University of Copenhagen, Copenhagen 2200, Denmark

(Ulrik, Godtfredsen, Hansen) Respiratory Research Unit and Department of Respiratory Medicine, Copenhagen University Hospital, Hvidovre 2650, Denmark

Publisher

MDPI

Year of Publication

2022

Link to the Ovid Full Text or citation:

[Click here for full text options](https://ovidsp.ovid.com/ovidweb.cgi?T=JS&CSC=Y&NEWS=N&PAGE=fulltext&D=emexb&AN=2020821031)

Link to the External Link Resolver:

[LibKey NHS](https://libkey.io/libraries/2789/openurl?genre=article&sid=OVID:emexb&genre=article&id=pmid:36554684&id=doi:10.3390%2Fijerph192416804&issn=1661-7827&volume=19&issue=24&spage=16804&pages=&date=2022&title=International+Journal+of+Environmental+Research+and+Public+Health&atitle=Sleep+Quality+and+Self-Reported+Symptoms+of+Anxiety+and+Depression+Are+Associated+with+Physical+Activity+in+Patients+with+Severe+COPD&aulast=Neale)

6.

Telemedicine and virtual respiratory care in the era of COVID-19.

Pinnock H., Murphie P., Vogiatzis I., Poberezhets V.

Embase

ERJ Open Research. 8(3) (no pagination), 2022. Article Number: 00111-2022. Date of Publication: 01 Jul 2022.

[Article]

AN: 2017661411

The World Health Organization defines telemedicine as an interaction between a health care provider and a patient when the two are separated by distance. The COVID-19 pandemic has forced a dramatic shift to telephone and video consulting for follow up and routine ambulatory care for reasons of infection control. Short Message Service (text) messaging has proved a useful adjunct to remote consulting allowing transfer of photographs and documents. Maintaining non-communicable diseases care is a core component of pandemic preparedness and telemedicine has developed to enable (for example) remote monitoring of sleep apnoea, telemonitoring of chronic obstructive pulmonary disease, digital support for asthma self-management, remote delivery of pulmonary rehabilitation. There are multiple exemplars of telehealth instigated rapidly to provide care for people with COVID-19, to manage the spread of the pandemic, or to maintain safe routine diagnostic or treatment services. Despite many positive examples of equivalent functionality and safety, there remain questions about the impact of remote delivery of care on rapport and the longer-term impact on patient/professional relationships. Although telehealth has the potential to contribute to universal health coverage by providing cost-effective accessible care, there is a risk of increasing social health inequalities if the digital divide excludes those most in need of care. As we emerge from the pandemic, the balance of remote versus face-to-face consulting, and the specific role of digital health in different clinical and healthcare contexts will evolve. What is clear is that telemedicine in one form or another will be part of the new norm.

Copyright © The authors 2022. All rights reserved.

Status

In-Process

Institution

(Pinnock) Usher Institute, The University of Edinburgh, Edinburgh, United Kingdom (Murphie) NHS Dumfries and Galloway, Scotland, United Kingdom

(Vogiatzis) Department of Sport, Exercise and Rehabilitation, Faculty of Health and Life Sciences, Northumbria University Newcastle, Newcastle upon Tyne, United Kingdom

(Poberezhets) Department of Propedeutics of Internal Medicine, National Pirogov Memorial Medical University, Vinnytsya, Ukraine

Publisher

European Respiratory Society

Year of Publication

2022

Link to the Ovid Full Text or citation:

[Click here for full text options](https://ovidsp.ovid.com/ovidweb.cgi?T=JS&CSC=Y&NEWS=N&PAGE=fulltext&D=emexb&AN=2017661411)

Link to the External Link Resolver:

[LibKey NHS](https://libkey.io/libraries/2789/openurl?genre=article&sid=OVID:emexb&genre=article&id=pmid:&id=doi:10.1183%2F23120541.00111-2022&issn=2312-0541&volume=8&issue=3&spage=00111-2022&pages=&date=2022&title=ERJ+Open+Research&atitle=Telemedicine+and+virtual+respiratory+care+in+the+era+of+COVID-19&aulast=Pinnock)

7.

Using a smartphone application maintains physical activity following pulmonary rehabilitation in patients with COPD: A randomised controlled trial.

Spielmanns M., Gloeckl R., Jarosch I., Leitl D., Schneeberger T., Boeselt T., Huber S., Kaur-Bollinger P., Ulm B., Mueller C., Bjoerklund J., Spielmanns S., Windisch W., Pekacka-Egli A.-M., Koczulla A.R.

Embase

Thorax. (no pagination), 2022. Date of Publication: 2022.

[Article]

AN: 638111303

Background: Evidence suggests that patients with COPD struggle to maintain improved physical activity (PA) after completing pulmonary rehabilitation (PR). Smartphone applications (apps) providing a comprehensive training programme have conferred healthy benefits. This study was conducted to determine whether regular usage of an app maintains PA following PR.

Method(s): Patients with stage II-IV COPD were enrolled in a 6-month trial following PR. After the screening period, participants were randomised into the Kaia COPD app group (intervention group (IG)) or the control group (CG). The primary outcome was PA (daily steps), measured using an activity tracker. Secondary outcomes included the COPD Assessment Test (CAT), the Chronic Respiratory Disease Questionnaire (CRQ) and the 1 min Sit-to-Stand Test (STST).

Result(s): Sixty participants completed the study. The median steps from baseline to 6 months were significantly different between the groups, in favour of the IG (-105.3, IQR -1970.1 to 2105.8, vs CG -1173.0, IQR -3813.1 to -93.8; p=0.007). CAT was significantly decreased in the IG (15.1+/-8.6 vs 19.7+/-6.4, p=0.02), whereas the CRQ subdomains for dyspnoea (4.5+/-1.7 vs 3.7+/-1.3, p=0.033) and fatigue (4.5+/-1.4 vs 3.5+/-1.3, p=0.028) improved significantly in the IG. The STST at 6 months was not significant. Sleep duration and sleep efficiency showed no significant differences between the two groups at any time.

Conclusion(s): A comprehensive program by using the Kaia app following PR maintained PA and improved symptoms in patients with COPD at 6 months. The app might be an important accessory tool for enhanced COPD care. Trial registration number: DRKS00017275.

Copyright © Author(s) (or their employer(s)) 2022. Re-use permitted under CC BY-NC. No commercial re-use. See rights and permissions. Published by BMJ.

PMID

35450945 [<https://www.ncbi.nlm.nih.gov/pubmed/?term=35450945>]

Status

Article-in-Press

Author NameID

Spielmanns, Marc; ORCID: <https://orcid.org/0000-0003-4541-6977> Windisch, Wolfram; ORCID: <https://orcid.org/0000-0001-7574-2672>

Institution

(Spielmanns, Spielmanns, Pekacka-Egli) Pulmonary Medicine, Zuercher RehaZentren Klinik Wald, Wald, Switzerland (Spielmanns, Windisch) Faculty of Health, Department for Pulmonary Medicine, University Witten Herdecke, Witten, Germany

(Gloeckl, Jarosch, Leitl, Schneeberger, Koczulla) Pulmonary Rehabilitation, Philipps-Universitat Marburg, Marburg, Germany

(Gloeckl, Jarosch, Leitl, Schneeberger, Koczulla) Institute for Pulmonary Rehabilitation Research, Schoen Klinik Berchtesgadener Land, Schoenau am Koenigssee, Germany

(Boeselt) Pulmonary Medicine, Philipps University Marburg, Faculty of Medicine, Marburg, Germany

(Huber, Kaur-Bollinger, Mueller, Bjoerklund) Kaia Health GmbH, Bavaria, Munich, Germany

(Ulm) Unabhaengige Statistische Beratungen Bernhard Ulm, Bavaria, Munich, Germany

(Windisch) Department of Pneumology, Cologne Merheim Hospital, Kliniken der Stadt Koeln, Koeln, Germany

Publisher

BMJ Publishing Group

Year of Publication

2022

Link to the Ovid Full Text or citation:

[Click here for full text options](https://ovidsp.ovid.com/ovidweb.cgi?T=JS&CSC=Y&NEWS=N&PAGE=fulltext&D=emexb&AN=638111303)

Link to the External Link Resolver:

[LibKey NHS](https://libkey.io/libraries/2789/openurl?genre=article&sid=OVID:emexb&genre=article&id=pmid:35450945&id=doi:10.1136%2Fthoraxjnl-2021-218338&issn=0040-6376&volume=&issue=&spage=thoraxjnl&pages=&date=2022&title=Thorax&atitle=Using+a+smartphone+application+maintains+physical+activity+following+pulmonary+rehabilitation+in+patients+with+COPD%3A+A+randomised+controlled+trial&aulast=Spielmanns)

8.

Pulmonary rehabilitation training for improving pulmonary function and exercise tolerance in patients with stable chronic obstructive pulmonary disease.

Jin L., An W., Li Z., Jiang L., Chen C.

Embase

American Journal of Translational Research. 13(7) (pp 8330-8336), 2021. Date of Publication: 2021.

[Article]

AN: 2013835508

Objective: To investigate the effect of pulmonary rehabilitation training on pulmonary function and exercise tolerance in patients with stable chronic obstructive pulmonary disease (COPD).

Method(s): By a random number table method, 90 patients with COPD admitted to our hospital from January 2019 to January 2020 were divided into three groups: the control group (conventional treatment), the observation group A (conventional treatment + pulmonary rehabilitation training three times a week) and the observation group B (conventional treatment + pulmonary rehabilitation training five times a week), with 30 patients in each group. The pulmonary function, exercise tolerance (the 6-min walking distance (6MWD)), sleep quality (Pittsburgh Sleep Quality Index (PSQI)) and quality of life (generic quality of life inventory-74 (GQOLI-74)) before and after intervention were compared among the three groups. Also, the satisfaction rate was recorded in all groups.

Result(s): After 6 months of intervention, the FEV1%, FVC% and FEV1/FVC as well as the GQOLI-74 scores increased significantly, while the PSQI scores decreased markedly in all groups as compared to those before intervention; the index levels and GQOLI-74 scores were significantly higher, and PSQI scores were markedly lower in the observation group B than in the other two groups (all P<0.05). After 3 and 6 months of intervention, the 6MWDs of the three groups were significantly increased compared with those before intervention, and the 6MWD was significantly longer in the observation group B than in the other two groups (P<0.05). Moreover, the satisfaction rate was significantly higher in observation group B than in the other two groups (P<0.05).

Conclusion(s): For patients with stable COPD, pulmonary rehabilitation training based on drug therapy can improve the pulmonary function, exercise tolerance, sleep quality and quality of life more effectively than drug treatment alone. What's more, the therapeutic effect of training five times a week is significantly better than that of training three times a week.

Copyright © 2021 E-Century Publishing Corporation. All rights reserved.

Status

In-Process

Institution

(Jin, Chen) Departments of Quality Control Section, Xintai Hospital of Traditional Chinese Medicine, Shandong Province, Xintai, China (An) Departments of Geriatrics, Xintai Hospital of Traditional Chinese Medicine, Shandong Province, Xintai, China

(Li) Traditional Chinese Medicine Hall, Xintai Hospital of Traditional Chinese Medicine, Shandong Province, Xintai, China

(Jiang) Departments of Pharmacy, Xintai Hospital of Traditional Chinese Medicine, Shandong Province, Xintai, China

Publisher

E-Century Publishing Corporation

Year of Publication

2021

Link to the Ovid Full Text or citation:

[Click here for full text options](https://ovidsp.ovid.com/ovidweb.cgi?T=JS&CSC=Y&NEWS=N&PAGE=fulltext&D=emexb&AN=2013835508)

Link to the External Link Resolver:

[LibKey NHS](https://libkey.io/libraries/2789/openurl?genre=article&sid=OVID:emexb&genre=article&id=pmid:&id=doi:&issn=1943-8141&volume=13&issue=7&spage=8330&pages=8330-8336&date=2021&title=American+Journal+of+Translational+Research&atitle=Pulmonary+rehabilitation+training+for+improving+pulmonary+function+and+exercise+tolerance+in+patients+with+stable+chronic+obstructive+pulmonary+disease&aulast=Jin)

9.

Grape Phytochemicals and Vitamin D in the Alleviation of Lung Disorders.

Santa K.

Embase

Endocrine, Metabolic and Immune Disorders - Drug Targets. 22(13) (pp 1276-1292), 2022. Date of Publication: November 2022.

[Review]

AN: 2018697054

Background: Typical lung diseases are pneumonia, asthma, sleep apnea syndrome (SA), interstitial pneumonia (IP), lung cancer, and chronic obstructive pulmonary disease (COPD). Corona-virus disease 2019 (COVID-19) is a type of viral pneumonia. Many researchers have reported that phy-tochemicals (chemical compounds produced by plants) and vitamin D are useful in stimulating our immunity. This review discusses the alleviation of lung diseases by grape phytochemicals and vitamin D.

Discussion(s): Pneumonia is an acute inflammation caused by the infection of pathogens; the worst case is a fatal cytokine storm in the lung. In asthma, allergens, tobacco smoke, or air pollution may cause seizures. Lung diseases caused by lung fibrosis may manifest chronic inflammation, progress into al-veolar fibrosis, and cause respiratory malfunction. SA is a lifestyle disease related to obesity and metabolic syndrome. To alleviate these symptoms, changing the eating habit is one of the strategies. Improvement in the daily lifestyle reduces the risk of lung cancer. Self-management, including nutritional management and exercise, is very important for COPD patients in addition to pharmacotherapy.

Conclusion(s): The intake of grape phytochemicals and vitamin D prevents the progress of lung diseases. Both phytochemicals and vitamin D prevent the production of proinflammatory cytokine, TNF-alpha, that is responsible for inflammation and lung diseases. Daily intake of grape phytochemicals is important. The optimum vitamin D level in serum is > 30 ng/mL. For the prevention of lung diseases, up-regulating immunity and maintaining good gut microbiota are important because gut microbiota change depending on what we eat.

Copyright © 2022 Bentham Science Publishers.

PMID

35388768 [<https://www.ncbi.nlm.nih.gov/pubmed/?term=35388768>]

Status

Embase

Institution

(Santa) Department of Biotechnology, Tokyo College of Biotechnology, Ota-ku, Tokyo, Japan

Publisher

Bentham Science Publishers

Year of Publication

2022

Link to the Ovid Full Text or citation:

[Click here for full text options](https://ovidsp.ovid.com/ovidweb.cgi?T=JS&CSC=Y&NEWS=N&PAGE=fulltext&D=emexa&AN=2018697054)

Link to the External Link Resolver:

[LibKey NHS](https://libkey.io/libraries/2789/openurl?genre=article&sid=OVID:emexa&genre=article&id=pmid:35388768&id=doi:10.2174%2F1871530322666220407002936&issn=1871-5303&volume=22&issue=13&spage=1276&pages=1276-1292&date=2022&title=Endocrine%2C+Metabolic+and+Immune+Disorders+-+Drug+Targets&atitle=Grape+Phytochemicals+and+Vitamin+D+in+the+Alleviation+of+Lung+Disorders&aulast=Santa)

10.

Analysis of the Effect of Mindfulness Behavior Intervention Combined with Progressive Breathing Training on Pulmonary Function Rehabilitation in Patients with Chronic Obstructive Pulmonary Disease.

Yu S., Fan H.

Embase

Emergency Medicine International. 2022 (no pagination), 2022. Article Number: 1698918. Date of Publication: 2022.

[Article]

AN: 2020204194

Purpose. Studies have shown that 50%-70% of patients with chronic obstructive pulmonary disease (COPD) have fatigue in addition to respiratory symptoms, so relieving respiratory symptoms and reducing fatigue are the main treatment objectives for COPD patients. This study focuses on the effect of positive behavioral intervention combined with progressive breathing training on pulmonary function rehabilitation in patients with COPD. Methods. 86 patients who underwent COPD treatment in our hospital between August 2020 and December 2021 were selected as study subjects and were divided into control (n = 43) and study groups (n = 43) using the random number table method. Patients in the control group were given conventional care, treatment, and health guidance, while patients in the study group were given positive behavioral intervention combined with progressive breathing training on this basis. Patients in both groups were compared on the basis of Multidimensional Fatigue Inventory 20 (MFI-20) score, the Medical Coping Questionnaire (MCMQ score), the Massive Attentional Awareness Scale (MAAS) score, and pulmonary function indicators (including the percentage of forced expiratory volume one second (FEV1%), peak expiratory flow (PEF), forced vital capacity (FVC), and 6-min walk distance (6MWD)) and quality of life (MCMQ) scores before and after 12 weeks of intervention. Results. After 12 weeks of intervention, the study group had higher MFI-20 scores (comprehensive fatigue, physical fatigue, reduced activity, decreased power, and mental fatigue), confrontation scores on the MCMQ scale, MAAS scores (observation, description, nonjudgmental to intrinsic experience, nonresponsiveness to intrinsic experience, and perceived behavior), FEV1%, PEF, FVC, and 6MWD levels than the control group (P<0.05). The scores of avoidance and submission on the MCMQ scale, and all scores of quality of life (cough, expectoration, shortness of breath, chest tightness, housework, going out, sleep, and energy) were lower than those of the control group (P<0.05). Conclusion. Positive behavioral interventions combined with progressive breathing training have a strengthening effect on the clinical treatment of COPD patients. Positive behavioral interventions combined with progressive breathing training are simple to implement as individual self-regulation methods and can be practiced on their own after being familiar with certain methods and techniques, and long-term adherence helps individuals cope with the stimulation of adverse events. Trail Registration. The clinical registration number for this research is L2020083.

Copyright © 2022 Shan Yu and Hui Fan.

Status

Embase

Author NameID

Fan, Hui; ORCID: <https://orcid.org/0000-0002-6646-7661>

Institution

(Yu, Fan) Respiratory and Critical Care Medicine, Renmin Hospital of Wuhan University, Hubei, Wuhan 430060, China

Publisher

Hindawi Limited

Year of Publication

2022

Link to the Ovid Full Text or citation:

[Click here for full text options](https://ovidsp.ovid.com/ovidweb.cgi?T=JS&CSC=Y&NEWS=N&PAGE=fulltext&D=emed23&AN=2020204194)

Link to the External Link Resolver:

[LibKey NHS](https://libkey.io/libraries/2789/openurl?genre=article&sid=OVID:emed23&genre=article&id=pmid:&id=doi:10.1155%2F2022%2F1698918&issn=2090-2840&volume=2022&issue=&spage=1698918&pages=&date=2022&title=Emergency+Medicine+International&atitle=Analysis+of+the+Effect+of+Mindfulness+Behavior+Intervention+Combined+with+Progressive+Breathing+Training+on+Pulmonary+Function+Rehabilitation+in+Patients+with+Chronic+Obstructive+Pulmonary+Disease&aulast=Yu)

11.

Intensive Intervention Improves Outcomes for Chronic Obstructive Pulmonary Disease Patients: A Medical Consortium-Based Management.

Zhao S., Zheng L., Zhu M., Shui Y., Bao X., Zhao J.

Embase

Canadian Respiratory Journal. 2022 (no pagination), 2022. Article Number: 6748330. Date of Publication: 2022.

[Article]

AN: 2019260861

Chronic obstructive pulmonary disease (COPD) is a major cause of morbidity and mortality. Strategies involving multidimensional approaches for the treatment of COPD are needed. This study aimed to evaluate the efficiency of medical consortium-based management for COPD. Patients were grouped in accordance with whether the hospitals they went to were under the medical consortium. We enrolled 141 COPD patients in the management group and 147 COPD patients in the control group. There was no predetermined sex and disease severity inclusion or exclusion criteria. Patients in the control group were managed by standard care, while patients in the management group were managed with intensive medical intervention jointly by specialists in the hospital and general practitioners and healthcare workers in community health centers. There was no difference in the basal demographics between the two groups. The basal condition of the management group was worse than that of the control group, demonstrated by a higher CAT score and a lower pulmonary function index. Half-year intensive intervention decreased CAT score from 17.28 to 15.62 and the Barthel ADL index from 73 to 60 in the management group, which was associated with better pulmonary rehabilitation, pursed-lip breathing, oxygen usage, and medicine regularity. The benefits became more obvious after one-year intensive intervention in the management group. There was a difference in mMRC grades and smoking cessation between the two groups. This study shows that a one-year intensive intervention improves the patients' health status and pulmonary function, suggesting that our medical consortium-based management is effective in the treatment of COPD.

Copyright © 2022 Shunjin Zhao et al.

PMID

35795169 [<https://www.ncbi.nlm.nih.gov/pubmed/?term=35795169>]

Status

Embase

Author NameID

Zhao, Jun; ORCID: <https://orcid.org/0000-0002-5051-2341>

Institution

(Zhao, Zheng, Zhu, Shui, Bao) Department of Respiratory and Critical Care Medicine, The Second Affiliated Hospital of Zhejiang University, Lanxi Branch, Lanxi People's Hospital, Zhejiang, China (Zhao) Department of Respiratory and Critical Care Medicine, Zhejiang Medical and Health Group Hangzhou Hospital, Zhejiang, China

Publisher

Hindawi Limited

Year of Publication

2022

Link to the Ovid Full Text or citation:

[Click here for full text options](https://ovidsp.ovid.com/ovidweb.cgi?T=JS&CSC=Y&NEWS=N&PAGE=fulltext&D=emed23&AN=2019260861)

Link to the External Link Resolver:

[LibKey NHS](https://libkey.io/libraries/2789/openurl?genre=article&sid=OVID:emed23&genre=article&id=pmid:35795169&id=doi:10.1155%2F2022%2F6748330&issn=1198-2241&volume=2022&issue=&spage=6748330&pages=&date=2022&title=Canadian+Respiratory+Journal&atitle=Intensive+Intervention+Improves+Outcomes+for+Chronic+Obstructive+Pulmonary+Disease+Patients%3A+A+Medical+Consortium-Based+Management&aulast=Zhao)

12.

Evaluation of the Norwegian version of the Dyspnoea-12 questionnaire in patients with COPD.

Garratt A.M., Nerheim E.M., Einvik G., Stavem K., Edvardsen A.

Embase

BMJ Open Respiratory Research. 9(1) (no pagination), 2022. Article Number: e001262. Date of Publication: 24 May 2022.

[Article]

AN: 638076565

Background The Dyspnoea-12 (D-12) questionnaire is widely used and tested in patients with breathing difficulties. The objective of this study was to translate and undertake the first evaluation of the measurement properties of the Norwegian version of the D-12 in patients with chronic obstructive pulmonary disease (COPD) attending a 4-week inpatient pulmonary rehabilitation programme. Methods Confirmatory factor analysis was used to assess structural validity. Fit to the Rasch partial credit model and differential item functioning (DIF) were assessed in relation to age, sex and comorbidity. Based on a priori hypotheses, validity was assessed through comparisons with scores for the COPD Assessment Test (CAT), Hospital Anxiety and Depression Scales (HADS) and clinical variables. Results There were 203 (86%) respondents with a mean age (SD) of 65.2 (9.0) years, and 49% were female. The D-12 showed satisfactory structural validity including presence of physical and affective domains. There was acceptable fit to Rasch model including unidimensionality for the two domains, and no evidence of DIF. Correlations with scores for the CAT, HADS and clinical variables were as hypothesised and highest for domains assessing similar aspects of health. Conclusions The Norwegian version of the D-12 showed good evidence for validity and internal consistency in this group of patients with COPD, including support for two separate domains. Further testing for these measurement properties is recommended in other Norwegian patients with dyspnoea.

Copyright © 2022 BMJ Publishing Group. All rights reserved.

PMID

35609940 [<https://www.ncbi.nlm.nih.gov/pubmed/?term=35609940>]

Status

Embase

Author NameID

Garratt, Andrew Malcolm; ORCID: <https://orcid.org/0000-0002-1000-4780> Stavem, Knut; ORCID: <https://orcid.org/0000-0003-4512-8000>

Institution

(Garratt) Division for Health Services, Norwegian Institute of Public Health, Oslo, Norway (Nerheim, Edvardsen) Lhl Hospital Gardermoen, Jessheim, Norway

(Einvik, Stavem) Institute of Clinical Medicine, University of Oslo, Oslo, Norway

(Einvik, Stavem, Edvardsen) Department of Pulmonary Medicine, Akershus University Hospital, Lorenskog, Norway

Publisher

BMJ Publishing Group

Year of Publication

2022

Link to the Ovid Full Text or citation:

[Click here for full text options](https://ovidsp.ovid.com/ovidweb.cgi?T=JS&CSC=Y&NEWS=N&PAGE=fulltext&D=emed23&AN=638076565)

Link to the External Link Resolver:

[LibKey NHS](https://libkey.io/libraries/2789/openurl?genre=article&sid=OVID:emed23&genre=article&id=pmid:35609940&id=doi:10.1136%2Fbmjresp-2022-001262&issn=2052-4439&volume=9&issue=1&spage=e001262&pages=&date=2022&title=BMJ+Open+Respiratory+Research&atitle=Evaluation+of+the+Norwegian+version+of+the+Dyspnoea-12+questionnaire+in+patients+with+COPD&aulast=Garratt)

13.

SPACE FOR COPD delivered as a maintenance programme on pulmonary rehabilitation discharge: protocol of a randomised controlled trial evaluating the long-term effects on exercise tolerance and mental well-being.

Alqahtani K.A., Gerlis C., Nolan C.M., Gardiner N., Szczepura A., Man W., Singh S.J., Houchen-Wolloff L.

Embase

BMJ Open. 12(4) (no pagination), 2022. Article Number: e055513. Date of Publication: 25 Apr 2022.

[Article]

AN: 638040599

Introduction The benefits achieved during pulmonary rehabilitation (PR) are known to be sustained for 6-12 months after the initial programme. Several maintenance trials have been conducted but were heterogeneous in terms of duration, frequency and labour cost. There is no consensus on one best strategy. SPACE FOR COPD (Self-management Programme of Activity, Coping and Education for Chronic Obstructive Pulmonary Disease) is a home-based self-management programme, which has been shown previously to be effective in primary and secondary care settings and is to be tested here as a maintenance programme. The aim is to evaluate the efficacy of the SPACE FOR COPD programme (manual and group sessions), on exercise tolerance and mental well-being, compared with usual care following PR in patients with COPD. Methods and analysis A prospective, multicentre, single-blinded randomised controlled trial requiring 116 participants with a clinical diagnosis of COPD who have finished PR within 4 weeks will be randomised 1:1 to either a usual care group or a SPACE FOR COPD programme group. The intervention comprises a home-based manual and 4, 2-hour group sessions adopting motivational interviewing techniques over 12 months. The primary outcome is endurance capacity measured by the Endurance Shuttle Walking Test at 12 months. Secondary outcomes are: maximal exercise capacity, health-related quality of life, mood, patient activation, physical activity, lung function and healthcare costs. The measures will be taken at baseline, 6 and 12 months. Patient interviews and staff focus groups will be conducted to explore barriers, facilitators and views about the intervention at the end of the study. A framework analysis will be used for the interpretation of qualitative data. Ethics and dissemination The trial was granted ethical approval from Health Research Authority and Health and Care Research Wales (HCRW19/EM/0267 on 10 October 2019). Results will be made available to all stakeholders through a dissemination event, conferences and peer-reviewed publications. Trial registration number ISRCTN30110012.

Copyright © 2022 BMJ Publishing Group. All rights reserved.

PMID

35470190 [<https://www.ncbi.nlm.nih.gov/pubmed/?term=35470190>]

Status

Embase

Author NameID

Alqahtani, Khaled A; ORCID: <https://orcid.org/0000-0001-5235-8467> Houchen-Wolloff, Linzy; ORCID: <https://orcid.org/0000-0003-4940-8835>

Nolan, Claire M; ORCID: <https://orcid.org/0000-0001-9067-599X>

Szczepura, Ala; ORCID: <https://orcid.org/0000-0001-6244-9872>

Institution

(Alqahtani) Respiratory Therapy Department, Jazan University, Jazan, Saudi Arabia (Alqahtani, Singh, Houchen-Wolloff) Respiratory Sciences, University of Leicester, Leicester, United Kingdom

(Gerlis, Gardiner, Singh, Houchen-Wolloff) Centre of Exercise and Rehabilitation Science, Leicester Biomedical Research Centre- Respiratory, Glenfield Hospital Respiratory Medicine Department, Leicester, United Kingdom

(Nolan, Man) Department of Respiratory Medicine, Royal Brompton and Harefield Nhs Foundation Trust, London, United Kingdom

(Szczepura) Faculty of Health and Life Sciences, Coventry University, Coventry, United Kingdom

(Man) Faculty of Medicine, Imperial College London, London, United Kingdom

Publisher

BMJ Publishing Group

Clinical Trial Number

ISRCTN30110012/ISRCTN

Year of Publication

2022

Link to the Ovid Full Text or citation:

[Click here for full text options](https://ovidsp.ovid.com/ovidweb.cgi?T=JS&CSC=Y&NEWS=N&PAGE=fulltext&D=emed23&AN=638040599)

Link to the External Link Resolver:

[LibKey NHS](https://libkey.io/libraries/2789/openurl?genre=article&sid=OVID:emed23&genre=article&id=pmid:35470190&id=doi:10.1136%2Fbmjopen-2021-055513&issn=2044-6055&volume=12&issue=4&spage=e055513&pages=&date=2022&title=BMJ+Open&atitle=SPACE+FOR+COPD+delivered+as+a+maintenance+programme+on+pulmonary+rehabilitation+discharge%3A+protocol+of+a+randomised+controlled+trial+evaluating+the+long-term+effects+on+exercise+tolerance+and+mental+well-being&aulast=Alqahtani)

14.

Community-Based Pulmonary Rehabilitation Programs in Individuals With COPD.

Barbosa M., Andrade R., de Melo C.A., Torres R.

Embase

Respiratory Care. 67(5) (pp 579-593), 2022. Date of Publication: 01 May 2022.

[Article]

AN: 2016536049

BACKGROUND: Community-based pulmonary rehabilitation (PR) programs can be offered to patients with COPD, but the literature on its effects is still not well summarized. Our purpose was to investigate the health-, physical-, and respiratory-related effects of community-based PR in individuals with COPD as compared to control groups.

METHOD(S): The PubMed and Embase databases were searched up to May 17, 2021. We included randomized control trials that compared the effects of community-based PR as compared to control groups in individuals with COPD. The risk of bias was judged using the Cochrane Risk of Bias 2 (RoB2). Meta-analysis was performed using a ran-dom-effects model to estimate the standardized mean difference (SMD) with 95% CI of the mean changes from baseline between groups. The Grading of Recommendations Assessment, Development, and Evaluation was used to interpret certainty of results.

RESULT(S): We included 10 randomized control studies comprising a total of 9,350 participants with weighted mean age of 62.3 6 2.38 y. The community-based interventions were based on exercise programs (resistance and/or endurance). All studies were judged as high risk and/or some concerns in one or more domains the risk of bias. All meta-analyses displayed very low certainty of evidence. The community-based PR interventions were significantly superior to control interventions in improving the St. George Respiratory Questionnaire Activity subscore (20.40 [95% CI 20.72 to 20.08]; k 5 5, n 5 382) and total score (20.73 [95% CI 21.29 to 20.18]; k 5 4, n 5 268) and the Chronic Respiratory Disease Questionnaire dyspnea subscore (0.36 [95% CI 0.03-0.69]; k 5 6, n 5 550). The mean changes from baseline were not different between the groups for all other outcomes.

CONCLUSION(S): Community-based PR tended to result in superior health-related quality of life and symptoms than control interventions, but the findings were inconsistent across outcomes and with very low certainty of evidence. Further studies are warranted for stronger conclusions.

Copyright © American Association for Respiratory Care. All rights reserved.

PMID

35473839 [<https://www.ncbi.nlm.nih.gov/pubmed/?term=35473839>]

Status

Embase

Institution

(Barbosa) Gaia/Espinho Hospital Center, North Rehabilitation Center, Gaia, Portugal (Barbosa) Camara Municipal de Arouca, Complexo Municipal Desportivo de Arouca e Piscinas Municipais de Escariz, Portugal

(Andrade) Clinica Espregueira-FIFA Medical Centre of Excellence, Porto, Portugal

(Andrade) Dom Henrique Research Centre, Porto, Portugal

(Andrade) Porto Biomechanics Laboratory (LABIOMEP), Faculty of Sports, University of Porto, Porto, Portugal

(de Melo) School of Allied Health Technologies, Polytechnic Institute of Porto, Porto, Portugal

(de Melo, Torres) CIR, Center for Rehabilitation Research, Polytechnic Institute of Porto, Porto, Portugal

(Torres) CESPU, North Polytechnic Institute of Health, Paredes, Portugal

Publisher

American Association for Respiratory Care

Year of Publication

2022

Link to the Ovid Full Text or citation:

[Click here for full text options](https://ovidsp.ovid.com/ovidweb.cgi?T=JS&CSC=Y&NEWS=N&PAGE=fulltext&D=emed23&AN=2016536049)

Link to the External Link Resolver:

[LibKey NHS](https://libkey.io/libraries/2789/openurl?genre=article&sid=OVID:emed23&genre=article&id=pmid:35473839&id=doi:10.4187%2Frespcare.09627&issn=0020-1324&volume=67&issue=5&spage=579&pages=579-593&date=2022&title=Respiratory+Care&atitle=Community-Based+Pulmonary+Rehabilitation+Programs+in+Individuals+With+COPD&aulast=Barbosa)

15.

Validity and reliability of a new incremental step test for people with chronic obstructive pulmonary disease.

Vilarinho R., Serra L., Aguas A., Alves C., Silva P.M., Caneiras C., Montes A.M.

Embase

BMJ Open Respiratory Research. 9(1) (no pagination), 2022. Article Number: e001158. Date of Publication: 06 Apr 2022.

[Article]

AN: 637692382

Background Incremental step tests (IST) can be used to assess exercise capacity in people with chronic obstructive pulmonary disease (COPD). The development of a new step test based on the characteristics of the incremental shuttle walk test (ISWT) is an important study to explore. We aimed to develop a new IST based on the ISWT in people with COPD, and assess its validity (construct validity) and reliability, according to Consensus-based Standards for the selection of health status Measurement Instruments (COSMIN) recommendations. Methods A cross-sectional study was conducted in participants recruited from hospitals/clinics. During the recruitment, the participants who presented a 6-minute walk test (6MWT) report in the previous month were also identified and the respective data was collected. Subsequently, participants attended two sessions at their homes. IST was conducted on the first visit, along with the 1 min sit-to-stand (1MSTS) test. IST was repeated on a second visit, performed 5-7 days after the first one. Spearman's correlations were used for construct validity, by comparing the IST with the 6MWT and the 1MSTS. Intraclass correlation coefficient (ICC 2,1), SE of measurement (SEM) and minimal detectable change at 95% CI (MDC95) were used for reliability. The learning effect was explored with the Wilcoxon signed-rank test. Results 50 participants (70.8+/-7.5 years) were enrolled. IST was significant and moderate correlated with the 6MWT (rho=0.50, p=0.020), and with the 1MSTS (rho=0.46, p=0.001). IST presented an ICC 2,1 =0.96, SEM=10.1 (16.6%) and MDC95=27.9 (45.8%) for the number of steps. There was a statistically significant difference between the two attempts of the IST (p=0.030). Conclusion Despite the significant and moderate correlations with the 6MWT and 1MSTS, the inability to full compliance with the COSMIN recommendations does not yet allow the IST to be considered valid in people with COPD. On the other hand, the IST is a reliable test based on its high ICC, but a learning effect and an indeterminate' measurement error were shown. Trial registration number NCT04715659.

Copyright © 2022 Georg Thieme Verlag. All rights reserved.

PMID

35387847 [<https://www.ncbi.nlm.nih.gov/pubmed/?term=35387847>]

Status

Embase

Institution

(Vilarinho, Silva, Montes) Department of Physiotherapy and Center for Rehabilitation Research, School of Health of Polytechnic, Institute of Porto, Porto, Portugal (Vilarinho, Serra, Aguas, Caneiras) Healthcare Department, Nippon Gases Portugal, Maia, Portugal

(Alves) Pulmonology Department, Centro Hospitalar Barreiro, Montijo, Barreiro, Portugal

(Alves) Pulmonology Coordination, Clinica Cuf Almada, Almada, Portugal

(Silva) Fisiomato, Matosinhos, Portugal

(Caneiras) Microbiology Research Laboratory on Environmental Health, Institute of Environmental Health, Faculty of Medicine, University of Lisbon, Lisbon, Portugal

(Caneiras) Institute for Preventive Medicine and Public Health, Faculty of Medicine, University of Lisbon, Lisbon, Portugal

(Montes) Department of Physiotherapy, Santa Maria Health School, Porto, Portugal

Publisher

BMJ Publishing Group

Year of Publication

2022

Link to the Ovid Full Text or citation:

[Click here for full text options](https://ovidsp.ovid.com/ovidweb.cgi?T=JS&CSC=Y&NEWS=N&PAGE=fulltext&D=emed23&AN=637692382)

Link to the External Link Resolver:

[LibKey NHS](https://libkey.io/libraries/2789/openurl?genre=article&sid=OVID:emed23&genre=article&id=pmid:35387847&id=doi:10.1136%2Fbmjresp-2021-001158&issn=2052-4439&volume=9&issue=1&spage=e001158&pages=&date=2022&title=BMJ+Open+Respiratory+Research&atitle=Validity+and+reliability+of+a+new+incremental+step+test+for+people+with+chronic+obstructive+pulmonary+disease&aulast=Vilarinho)

16.

Nasal High Flow Therapy For Symptom Management in People Receiving Palliative Care.

Huang J.Y., Steele P., Dabscheck E., Smallwood N.

Embase

Journal of Pain and Symptom Management. 63(2) (pp e237-e245), 2022. Date of Publication: February 2022.

[Article]

AN: 2015376512

For patients with chronic non-malignant lung disease, severe chronic breathlessness can significantly impact quality of life, causing significant disability, distress, social isolation, and recurrent hospital admissions. Caregivers for people with challenging symptoms, such as severe breathlessness, are also profoundly impacted. Despite increasing research focused on breathlessness over recent years, this symptom remains extremely difficult to manage, with no effective treatment that completely relieves breathlessness. A new potential treatment for relieving breathlessness in patients at home is nasal high flow (NHF) therapy. NHF therapy is a respiratory support system that delivers heated, humidified air (together with oxygen if required) with flows of up to 60 L/min. This case describes a patient with very severe chronic obstructive pulmonary disease who received domiciliary NHF therapy (approximately 8 hours/day, flow rate of 20 L/min) over twelve months with good effect for the relief of severe chronic breathlessness. We discuss the management principles for severe chronic breathlessness, the physiological effects of NHF therapy and the evidence for long-term use in the community setting. With the support of respiratory and palliative care clinicians together, domiciliary NHF therapy has great potential for improving current symptom management approaches in people with life-limiting illnesses.

Copyright © 2021 American Academy of Hospice and Palliative Medicine

PMID

34600084 [<https://www.ncbi.nlm.nih.gov/pubmed/?term=34600084>]

Status

Embase

Author NameID

Huang, Joanna Yilin; ORCID: <https://orcid.org/0000-0003-2391-1184> Steele, Patrick; ORCID: <https://orcid.org/0000-0002-1409-5771>

Smallwood, Natasha; ORCID: <https://orcid.org/0000-0002-3403-3586>

Institution

(Huang, Dabscheck, Smallwood) Department of Respiratory Medicine, Alfred Hospital, Melbourne, Victoria, Australia (Steele) Department of Palliative Care, Royal Melbourne Hospital, Melbourne, Victoria, Australia

(Steele) Department of Palliative Care, Peter MacCallum Cancer Centre, Parkville, Melbourne, VIC, Australia

(Steele) Department of Palliative Care, Monash Health, Clayton Road, Clayton, VIC, Australia

(Steele) Palliative Care South East, Sladen Street, Cranbourne, VIC, Australia

(Dabscheck) Central Clinical School (Alfred Hospital), Monash University, Melbourne, Victoria, Australia

(Smallwood) Department of Immunology & Respiratory Medicine, Central Clinical School (Alfred Hospital), Monash University, Melbourne, Victoria, Australia

Publisher

Elsevier Inc.

Year of Publication

2022

Link to the Ovid Full Text or citation:

[Click here for full text options](https://ovidsp.ovid.com/ovidweb.cgi?T=JS&CSC=Y&NEWS=N&PAGE=fulltext&D=emed23&AN=2015376512)

Link to the External Link Resolver:

[LibKey NHS](https://libkey.io/libraries/2789/openurl?genre=article&sid=OVID:emed23&genre=article&id=pmid:34600084&id=doi:10.1016%2Fj.jpainsymman.2021.09.016&issn=0885-3924&volume=63&issue=2&spage=e237&pages=e237-e245&date=2022&title=Journal+of+Pain+and+Symptom+Management&atitle=Nasal+High+Flow+Therapy+For+Symptom+Management+in+People+Receiving+Palliative+Care&aulast=Huang)

17.

HOME REHABILITATION FOR COPD: A RANDOMIZED STUDY OF REMOTE ACTIVITY MONITORING AND HEALTH COACHING.

BENZO R.P., E MCEVOY C., M. CLARK M., BENZO M., M JOHNSON M., NOVOTNY P.A.U.L.

Embase

Chest. Conference: CHEST 2022 Annual Meeting. Nashville United States. 162(4 Supplement) (pp A1981), 2022. Date of Publication: October 2022.

[Conference Abstract]

AN: 2020470267

SESSION TITLE: COPD Outcomes from the Hospital to Home SESSION TYPE: Original Investigations PRESENTED ON: 10/17/22 1:30 PM - 2:30 PM PURPOSE: Quality of life (QoL) is what matters the most to patients and in COPD is associated with healthcare utilization and survival. Pulmonary Rehabilitation is the most effective in improving QoL but has low uptake and adherence. Home-based programs are a proposed solution. There is a knowledge gap on effective and sustainable home-based programs that can impact QoL in patients with COPD.

OBJECTIVE(S): To determine whether remote patient monitoring with health coaching improves physical and emotional disease-specific quality of life measured by the Chronic Respiratory Questionnaire (CRQ).

METHOD(S): This multicenter clinical trial enrolled 375 adult patients with COPD, randomized to a 12-week remote patient monitoring with health coaching (N=188) or wait-list usual care (n = 187). Measurements Primary outcomes: Physical and Emotional quality of life. Prespecified secondary outcomes included CRQ-dyspnea, CRQ-fatigue, CRQ-emotions, CRQ-mastery, daily physical activity, self-management abilities, symptoms of depression/anxiety, ER/Hospital admissions, and sleep.

RESULT(S): Participants age, 69+9 years; 59% women, FEV1% 45+19. At 12 weeks there was a significant and clinically meaningful difference between the intervention vs. the control group in the physical and emotional CRQ summary scores: ((Change difference (95% CI) 0.54 points (0.36, 0.73) p<0.001, 0.51 (0.39, 0.69) p<0.001 respectively. All CRQ domains, Self-management, Sleep, and Depression scores improved (p<0.01) but physical activity did not reach significance. CRQ changes were maintained at 24 weeks.

CONCLUSION(S): Remote monitoring with health coaching promotes COPD Wellness and behavior change given its effect on all aspects of QoL, self-management, sleep, and depression scores. It represents an effective option for home-based rehabilitation. CLINICAL IMPLICATIONS: Remote monitoring with Health Coaching is a billable practice using of Remote Patient Monitoring and Remote therapeutic monitoring CPT codes and may rerpesent a feasible and effective tool in the portfolio for the rehabilitaiton of patient COPD DISCLOSURES: No relevant relationships by Roberto Benzo No relevant relationships by Maria Benzo No relevant relationships by Matthew Clark No relevant relationships by Margaret Johnson Scientific Medical Advisor relationship with Respirtech a Philips Company Please note: $5001 - $20000 by Charlene McEvoy, value=Grant/Research Support Research investigator relationship with GSK Please note: 2020-2021 Added 06/30/2022 by Charlene McEvoy, value=Grant/Research research investigator relationship with AstraZeneca Please note: 2018-present Added 06/30/2022 by Charlene McEvoy, value=Grant/Research Support No relevant relationships by Paul Novotny

Copyright © 2022 American College of Chest Physicians

Status

CONFERENCE ABSTRACT

Publisher

Elsevier Inc.

Year of Publication

2022

Link to the Ovid Full Text or citation:

[Click here for full text options](https://ovidsp.ovid.com/ovidweb.cgi?T=JS&CSC=Y&NEWS=N&PAGE=fulltext&D=emed23&AN=2020470267)

Link to the External Link Resolver:

[LibKey NHS](https://libkey.io/libraries/2789/openurl?genre=article&sid=OVID:emed23&genre=article&id=pmid:&id=doi:10.1016%2Fj.chest.2022.08.1631&issn=0012-3692&volume=162&issue=4+Supplement&spage=A1981&pages=A1981&date=2022&title=Chest&atitle=HOME+REHABILITATION+FOR+COPD%3A+A+RANDOMIZED+STUDY+OF+REMOTE+ACTIVITY+MONITORING+AND+HEALTH+COACHING&aulast=BENZO)

18.

Prospective Out-Patient Study of Pulmonary Rehabilitation for Long COVID: Pilot Study.

Windt M.R., Flanagan L., Mullaney H.

Embase

American Journal of Respiratory and Critical Care Medicine. Conference: International Conferenceof the American Thoracic Society, ATS 2022. San Francisco, CA United States. 205(1) (no pagination), 2022. Date of Publication: May 2022.

[Conference Abstract]

AN: 638404484

Rationale: WHO defines long Covid as a condition with at least one symptom that usually begins within three months of the onset of confirmed or probable infection with the coronavirus, persists for at least two months, and cannot be explained by other diagnoses. The most common symptoms are fatigue, shortness of breath, and cognitive problems. We have previously published that pulmonary rehabilitation (PR) reduces sensations of dyspnea, which is a shared complaint, in chronic obstructive pulmonary disease (COPD), asthma and obstructive sleep apnea (OSA) patients. Some of the primary goals of PR are to restore functional capacity and cardiopulmonary fitness in patients with lung disease. PR studies with COPD have examined changes to physiological variables that quantify improvements in functional capacity and cardiorespiratory fitness. To our knowledge there has never been a prospective outpatient study of the efficacy of pulmonary rehabilitation in long Covid. We believe that improvement in dyspnea and functional capacity as shown in COPD and asthma following PR can also benefit patients with similar symptoms of long Covid.

Method(s): Nine long Covid patients completed our eight-week home-based PR program. The program consisted of one-session-per-week in which the patients performed exercise supervised by an exercise physiologist and received education on a variety of topics including nutrition, balance, and energy conservation. Patients completed individualized cardiovascular and resistance exercises, as well as inspiratory muscle strengthening using a Respironics Threshold Inspiratory Muscle Trainer(IMT). SF-36 health questionnaire and Baecke fitness questionnaire were obtained pre-and post-PR. VO2 max was determined using a six-minute walk test (6MWT) prior to and after completion of the PR program.

Result(s): Significant improvements were seen in the 6MWT gait speed (P=<0.0001), IMT strength (P=<0.0001), and Baecke fitness questionnaire (P=0.002). No significant difference was seen in VO2 max nor the SF-36 questionnaire.

Conclusion(s): After 8 weeks of our home based PR program, a significant improvement in parameters was observed in all long Covid patients. These findings show that our PR program leads to positive improvement in functional capacity and cardiorespiratory fitness in patients with long Covid and suggests PR be considered in patients with long Covid. A randomized control trial would be necessary to confirm and better identify the benefits of PR in long Covid patients. (Figure Presented).

Status

CONFERENCE ABSTRACT

Institution

(Windt) Ctr for Asthma Allergy and Resp Disease, North Hampton, NH, United States (Flanagan, Mullaney) Pulmonary Rehabilitation, Ctr for Asthma Allergy and Resp Disease, North Hampton, NH, United States

Publisher

American Thoracic Society

Year of Publication

2022

Link to the Ovid Full Text or citation:

[Click here for full text options](https://ovidsp.ovid.com/ovidweb.cgi?T=JS&CSC=Y&NEWS=N&PAGE=fulltext&D=emed23&AN=638404484)

Link to the External Link Resolver:

[LibKey NHS](https://libkey.io/libraries/2789/openurl?genre=article&sid=OVID:emed23&genre=article&id=pmid:&id=doi:10.1164%2Fajrccm-conference.2022.205.1_MeetingAbstracts.A4962&issn=1535-4970&volume=205&issue=1&spage=&pages=&date=2022&title=American+Journal+of+Respiratory+and+Critical+Care+Medicine&atitle=Prospective+Out-Patient+Study+of+Pulmonary+Rehabilitation+for+Long+COVID%3A+Pilot+Study&aulast=Windt)

19.

Effect of Home-Based Rehabilitation with Health Coaching on Chronic Obstructive Pulmonary Disease Outcomes: A Randomized Study.

Benzo R., Hoult J.P., Mcevoy C.E., Clark M., Benzo M.V., Johnson M.M., Novotny P.

Embase

American Journal of Respiratory and Critical Care Medicine. Conference: International Conferenceof the American Thoracic Society, ATS 2022. San Francisco, CA United States. 205(1) (no pagination), 2022. Date of Publication: May 2022.

[Conference Abstract]

AN: 638407840

Importance Pulmonary rehabilitation (PR) is the guideline-recommended most effective nonpharmacological therapy for people with chronic obstructive pulmonary disease (COPD) improving all outcomes. Despite the proven benefits, PR programs have low participant uptake, insufficient attendance, and high drop-out rates. Home programs are proposed as a solution: however, there is no randomized study to date in the US to inform remote programs.Objective To determine whether unsupervised home-based rehabilitation with technology and health coaching improve physical and emotional disease specific quality of life, daily physical activity, and self-management in patients with moderate to severe Chronic Obstructive Pulmonary Disease (COPD).Design, Setting, and Participants This multicenter, randomized, allocation-concealed, clinical trial enrolled 235 adult patients with COPD, of a planned sample size of 200, between March 2018 and December 2021 from two major health care systems in the US.Interventions Participants were randomized to unsupervised home-based rehabilitation with health coaching (N=116) or standard care (n = 119).Main Outcomes and Measures The primary outcome were disease-specific physical and emotional quality of life after the 12-week intervention. Prespecified secondary outcomes included measured daily physical activity, selfmanagement abilities, sleep, and symptoms of depression and anxiety.Results Among 307 patients who were randomized (mean age, 69 years; 56% women) 235 (77%) completed the intervention and had measures. The was a significant difference in the intervention compared to the control group in the primary outcomes, daily physical activity, self-management, sleep, and depression scores: (adjusted Difference, Mean Change (95% CI) 0.47 points (0.27, 0.67) p<0.001, 0.48 (0.27, 0.69)p<0.001 for the physical and emotional quality of life respectively. Daily steps 655.83 (148.03, 1163.64) p<0.0116, selfmanagement 3.83 (1.85, 5.79) p<0.001, depression PHQ-9 -1.2 (-2.04, -0.35) p<0.0056 and total sleep time 54 min (6.74, 102.96) p<0.025 .Conclusions and Relevance Among patients with moderate to severe COPD, unsupervised home-based rehabilitation with monitoring technology and health coaching improved quality of life, daily physical activity, and self-management. This intervention represents an opportunity to increase the uptake of rehabilitation in COPD and to inform options of remote care that are now in increased demand in the context of the COVID-19 pandemic. (Figure Presented).

Status

CONFERENCE ABSTRACT

Institution

(Benzo) Pulm and CCM, Mayo Clinic, Rochester, MN, United States (Hoult, Benzo) Mindful Breathing Lab, Mayo Clinic, Rochester, MN, United States

(Mcevoy) Health Partners Institute, Minneapolis, MN, United States

(Clark) Psychology, Mayo Clinic, Rochester, MN, United States

(Johnson) Div of Pulm Med, Mayo Clinic - Jacksonville, Jacksonville, FL, United States

(Novotny) Statistics, Mayo Clinic, Rochester, MN, United States

Publisher

American Thoracic Society

Year of Publication

2022

Link to the Ovid Full Text or citation:

[Click here for full text options](https://ovidsp.ovid.com/ovidweb.cgi?T=JS&CSC=Y&NEWS=N&PAGE=fulltext&D=emed23&AN=638407840)

Link to the External Link Resolver:

[LibKey NHS](https://libkey.io/libraries/2789/openurl?genre=article&sid=OVID:emed23&genre=article&id=pmid:&id=doi:10.1164%2Fajrccm-conference.2022.205.1_MeetingAbstracts.A1027&issn=1535-4970&volume=205&issue=1&spage=&pages=&date=2022&title=American+Journal+of+Respiratory+and+Critical+Care+Medicine&atitle=Effect+of+Home-Based+Rehabilitation+with+Health+Coaching+on+Chronic+Obstructive+Pulmonary+Disease+Outcomes%3A+A+Randomized+Study&aulast=Benzo)

20.

Outcomes Following Comprehensive Pulmonary Rehabilitation After COVID-19 Infection.

O'Beirne S.L., O'Mahony A.M., Tonge P., Jackson N., Kidney D., Nolan G.

Embase

American Journal of Respiratory and Critical Care Medicine. Conference: International Conferenceof the American Thoracic Society, ATS 2022. San Francisco, CA United States. 205(1) (no pagination), 2022. Date of Publication: May 2022.

[Conference Abstract]

AN: 638407127

RATIONALE: The Coronavirus disease 2019 (COVID-19) pandemic caused by infection with severe acute respiratory syndrome coronavirus 2 (SARS-CoV-2) leads to a wide spectrum of illness from mild symptoms to respiratory failure, and has been associated with significant morbidity and mortality. Persistent, debilitating post-COVID sequelae including dyspnea, fatigue and cognitive dysfunction have been reported in survivors both of severe acute illness requiring hospitalization and those with milder initial disease with many patients experiencing ongoing symptoms that impact on exercise capacity, QOL and productivity up to 6 months later. Pulmonary rehabilitation programs (PRP) have been suggested as an approach to address these issues in a holistic manner.

METHOD(S): Demographic data along with pre and post rehabilitation outcome measures including 6-minute walk test (6MWT) distance, arm grip strength (AGS), Chronic Obstructive Pulmonary Disease (COPD) Assessment Tool (CAT) and St. George's Respiratory Questionnaire (SGRQ) scores, Hospital Anxiety and Depression Scale (HADS) and the Post- COVID-19 Functional Scale (PCFS) were collected prospectively from individuals enrolled in a post-COVID-19 PRP. The program included twice weekly exercises classes and weekly occupational therapy sessions addressing additional symptoms including fatigue, sleep disturbance and neurocognitive deficits. Descriptive statistics were used to summarize patient demographics, pre and post rehabilitation measures were compared using a paired t-test with pvalues <0.05 considered significant.

RESULT(S): Of 96 patients assessed, 71 individuals completed an 8-week outpatient PRP. Of those completing the program, the mean age was 52.3+/-14.1 years, 55% were female. Overall, 35% of individuals had been hospitalized, the majority of whom developed respiratory failure requiring oxygen therapy +/- additional supports including non-invasive and invasive ventilation. A clinically and statistically significant improvement in the 6MWT of 99.7+/-65.2 m from 413.1+/-78.5 to 512.8+/-88.1 m (p<0.0001) was observed post rehabilitation. Significant improvements were also seen in right (64.3+/-23.4 to 70.9+/-23.5 lbs) and left (62.3+/-23.7 to 68.0+/-22.7 lbs) AGS, both p values <0.0001. Additionally, a clinically and statistically significant improvement was demonstrated in symptoms scores including the CAT (18.9+/-6.4 to 13.1+/-6.), SGRQ (43.0+/-18.3 to 32.7+/-18.6) and the HADS (14.3+/-7.1 to 12.0+/-7.4), all p values <0.0001 along with a significant improvement in the PCFS from 2 to 1 point (p<0.0001).

CONCLUSION(S): A comprehensive PRP for individuals with ongoing symptoms following COVID-19 infection is associated with significant and clinically meaningful improvement in 6MWT distance, peripheral muscle strength, CAT, SGRQ and HADS scores and the PCFS. These data support the role of PRPs in individuals suffering from post-acute COVID syndrome.

Status

CONFERENCE ABSTRACT

Institution

(O'Beirne) Respiratory Medicine, St Michael's Hospital, St Vincent's University Hospital, Dublin, Ireland (O'Mahony, Nolan) Respiratory Medicine, St Vincent's University Hospital, Dublin, Ireland

(Tonge, Jackson) Respiratory Medicine, St Michael's Hospital, Dun Laoghaire, Ireland

(Kidney) Speech and Language Therapy, St Vincent's University Hospital, Dublin, Ireland

Publisher

American Thoracic Society

Year of Publication

2022

Link to the Ovid Full Text or citation:

[Click here for full text options](https://ovidsp.ovid.com/ovidweb.cgi?T=JS&CSC=Y&NEWS=N&PAGE=fulltext&D=emed23&AN=638407127)

Link to the External Link Resolver:

[LibKey NHS](https://libkey.io/libraries/2789/openurl?genre=article&sid=OVID:emed23&genre=article&id=pmid:&id=doi:10.1164%2Fajrccm-conference.2022.205.1_MeetingAbstracts.A3713&issn=1535-4970&volume=205&issue=1&spage=&pages=&date=2022&title=American+Journal+of+Respiratory+and+Critical+Care+Medicine&atitle=Outcomes+Following+Comprehensive+Pulmonary+Rehabilitation+After+COVID-19+Infection&aulast=O'Beirne)

21.

Factors influencing physical activity levels in chronic obstructive pulmonary disease.

Myers J., Morris N., Chambers D., Yerkovich S., Hopkins P., Walsh J.

Embase

Respirology. Conference: TSANZSRS 2022, The Australia and New Zealand Society of Respiratory Science and The Thoracic Society of Australia and New Zealand, ANZSRS/TSANZ Annual Scientific Meeting for Leaders in Lung Health and Respiratory Science. Virtual. 27(SUPPL 1) (pp 131), 2022. Date of Publication: March 2022.

[Conference Abstract]

AN: 637794859

Introduction/Aim: Reduced physical activity level (PAL) in the chronic obstructive pulmonary disease (COPD) population is linked with poorer survival. However, the clinical characteristics contributing to PAL, across COPD severity, remains unknown. This study determined baseline characteristics contributing to PAL across a range of disease severity in a pre-pulmonary rehabilitation (PR) and a pre-lung transplantation (LTx) population.

Method(s): PAL was measured using Sensewear (Pro3 and MF) armband worn for 3 days minimum and > 22 hrs. PAL was defined as the mean daily energy expenditure averaged over the evening/sleep energy expenditure. Using a retrospective cross-sectional design, all COPD at a single institution attending either pre-PR or pre-assessment LTx between 2008 and 2016 were considered for inclusion. Baseline characteristics of demographics (age, sex, lung condition, body mass index [BMI]); lung function (FEV1% predicted), quadricep strength (QS%) and 6-min walk distance (6MWD) were analysed using univariable and multivariable regression.

Result(s): 72 pre-PR assessment (49 males; age 66 +/- 8 year; FEV1 49% [95%CI 45-52]) and 98 pre-LTx assessment (41 male; age 54 +/- 7 year; FEV1 24% [95% CI 23-28]) patients were included. Pre-PR participants had significantly (p < 0.001) higher PAL (1.50 [95%CI 1.45-1.55] vs. 1.38 [95%CI 1.33-1.43]) and 6MWD (436 +/- 99 m vs. 305 +/- 87 m), however pre-LTx participants were significantly (p < 0.001) younger and had a higher QS% (59% [95%CI 55-67] vs. 88% [95%CI 84-93]). Multivariable analysis found the pre-PR group BMI both. (beta = -0.05, p = 0.02) and 6MWD (beta = 0.06, p = 0.01) were independent predictors of increased PAL (R2 = 14.7%). In the LTx group only QS% (beta = 0.02, p = 0.05) was an independent predictor of increased PAL (R2 = 3.9%).

Conclusion(s): 6MWD and BMI are significant but account for 14% of the total PAL variance in pre-PR patients. In the Pre- LTx group only QS% was significant accounting for 3.8%. Highlighting that PAL is a highly complex measure that is difficult to influence with 86% variance unable to be explained. Based on our findings, patient selection for LTx tend to be sicker, younger yet stronger than the pre-PR group.

Status

CONFERENCE ABSTRACT

Institution

(Myers, Morris, Walsh) Physiotherapy Department, Prince Charles Hospital, Brisbane, Australia (Chambers, Yerkovich, Hopkins, Walsh) Queensland Lung Transplant Service, Prince Charles Hospital, Brisbane, Australia

(Myers, Morris, Walsh) School of Allied Health Sciences, Griffith University, Gold Coast, Australia

(Chambers, Hopkins) School of Medicine, University of Queensland, Brisbane, Australia

Publisher

Blackwell Publishing

Year of Publication

2022

Link to the Ovid Full Text or citation:

[Click here for full text options](https://ovidsp.ovid.com/ovidweb.cgi?T=JS&CSC=Y&NEWS=N&PAGE=fulltext&D=emed23&AN=637794859)

Link to the External Link Resolver:

[LibKey NHS](https://libkey.io/libraries/2789/openurl?genre=article&sid=OVID:emed23&genre=article&id=pmid:&id=doi:10.1111%2Fresp.14226&issn=1323-7799&volume=27&issue=SUPPL+1&spage=131&pages=131&date=2022&title=Respirology&atitle=Factors+influencing+physical+activity+levels+in+chronic+obstructive+pulmonary+disease&aulast=Myers)

22.

New Zealand COPD Guidelines: Quick Reference Guide.

Hancox R.J., Jones S., Baggott C., Chen D., Corna N., Davies C., Fingleton J., Hardy J., Hussain S., Poot B., Reid J., Travers J., Turner J., Young R.

Embase

New Zealand Medical Journal. 134(1530) (pp 76-110), 2021. Date of Publication: 19 Feb 2021.

[Article]

AN: 2017254581

The purpose of the Asthma and Respiratory Foundation of New Zealand's COPD Guidelines: Quick Reference Guide is to provide simple, practical, evidence-based recommendations for the diagnosis, assessment, and management of chronic obstructive pulmonary disease (COPD) in clinical practice. The intended users are health professionals responsible for delivering acute and chronic COPD care in community and hospital settings, and those responsible for the training of such health professionals.

Copyright © NZMA

PMID

33651780 [<https://www.ncbi.nlm.nih.gov/pubmed/?term=33651780>]

Status

Embase

Institution

(Hancox) Waikato District Health Board, Hamilton, New Zealand (Hancox, Reid) University of Otago, Dunedin, New Zealand

(Jones, Corna) Middlemore Hospital, Counties Manukau, Auckland, New Zealand

(Baggott, Fingleton, Hardy) Medical Research Institute of New Zealand, New Zealand

(Chen) Canterbury Clinical Network, Christchurch, New Zealand

(Davies) Tu Kotahi Maori Asthma Trust, New Zealand

(Fingleton) Capital and Coast District Health Board, Wellington, New Zealand

(Hussain, Young) Auckland District Health Board, New Zealand

(Poot, Travers) Hutt Valley District Health Board, Lower Hutt, New Zealand

(Poot) School of Nursing, Midwifery and Health Practice, Victoria University of Wellington, Wellington, New Zealand

(Reid) Best Practice Advisory Centre (BPAC), Dunedin, New Zealand

(Turner) Asthma and Respiratory Foundation of New Zealand, New Zealand

Publisher

New Zealand Medical Association

Year of Publication

2021

Link to the Ovid Full Text or citation:

[Click here for full text options](https://ovidsp.ovid.com/ovidweb.cgi?T=JS&CSC=Y&NEWS=N&PAGE=fulltext&D=emed22&AN=2017254581)

Link to the External Link Resolver:

[LibKey NHS](https://libkey.io/libraries/2789/openurl?genre=article&sid=OVID:emed22&genre=article&id=pmid:33651780&id=doi:&issn=0028-8446&volume=134&issue=1530&spage=76&pages=76-110&date=2021&title=New+Zealand+Medical+Journal&atitle=New+Zealand+COPD+Guidelines%3A+Quick+Reference+Guide&aulast=Hancox)

23.

Perioperative Pulmonary Support of the Elderly.

Entriken C., Pritts T.A.

Embase

Current Geriatrics Reports. 10(4) (pp 167-174), 2021. Date of Publication: December 2021.

[Review]

AN: 2014160722

Purpose of Review: With the projected increase in the geriatric patient population, it is of the utmost importance to understand and optimize conditions in the perioperative period to ensure the best surgical outcome. Age-associated changes in respiratory physiology affect the surgical management of geriatric patients. This review focuses on perioperative pulmonary management of elderly individuals. Recent Findings: The physiological changes associated with aging include both physical and biochemical alterations that are detrimental to pulmonary function. There is an increased prevalence of chronic lung disease such as COPD and interstitial lung disease which can predispose patients to postoperative pulmonary complications. Additionally, elderly patients, especially those with chronic lung disease, are at risk for frailty. Screening tools have been developed to evaluate risk and aid in the judicious selection of patients for surgical procedures. The concept of "prehabilitation" has been developed to best prepare patients for surgery and may be more influential in the reduction of postoperative pulmonary complications than postoperative rehabilitation. Understanding the age-associated changes in metabolism of drugs has led to dose adjustments in the intraoperative and postoperative periods, reducing respiratory depression and lung protective ventilation and minimally invasive procedures have yielded reductions in postoperative pulmonary complications.

Summary: The perioperative management of the geriatric population can be divided into three key areas: preoperative risk mitigation, intraoperative considerations, and postoperative management. Preoperative considerations include patient selection and thorough history and physical, along with smoking cessation and prehabilitation in a subset of patients. Operative aspects include careful selection of anesthetic agents, lung protective ventilation, and choice of surgical procedure. Postoperative management should focus on selective use of agents that may contribute to respiratory depression and encouragement of rehabilitation.

Copyright © 2021, The Author(s), under exclusive licence to Springer Science+Business Media, LLC, part of Springer Nature.

Status

Embase

Author NameID

Pritts, Timothy A.; ORCID: <https://orcid.org/0000-0003-2495-3255>

Institution

(Entriken, Pritts) Section of General Surgery, Department of Surgery, University of Cincinnati, Cincinnati, OH, United States

Publisher

Springer

Year of Publication

2021

Link to the Ovid Full Text or citation:

[Click here for full text options](https://ovidsp.ovid.com/ovidweb.cgi?T=JS&CSC=Y&NEWS=N&PAGE=fulltext&D=emed22&AN=2014160722)

Link to the External Link Resolver:

[LibKey NHS](https://libkey.io/libraries/2789/openurl?genre=article&sid=OVID:emed22&genre=article&id=pmid:&id=doi:10.1007%2Fs13670-021-00369-3&issn=2196-7865&volume=10&issue=4&spage=167&pages=167-174&date=2021&title=Current+Geriatrics+Reports&atitle=Perioperative+Pulmonary+Support+of+the+Elderly&aulast=Entriken)

24.

The respiratory rehabilitation Maugeri network service reconfiguration after 1 year of COVID-19.

Vitacca M., Ceriana P., Balbi B., Bruschi C., Aliani M., Maniscalco M., Fanfulla F., Diasparra A., Rizzello L., Sereni D., Spanevello A.

Embase

Monaldi Archives for Chest Disease. 91(4) (no pagination), 2021. Article Number: 1843. Date of Publication: 01 Jan 2021.

[Review]

AN: 2016416772

As part of the Italian Health Service the respiratory ICS Maugeri network were reconfigured and several in-hospital programs were suspended to be substituted by workforce and facilities reorganization for acute and post-acute COVID-19 care need. The present review shows the time course variation of respiratory ICS network in terms of admissions diagnosis and outcomes. A comparative review of the admissions and outcome measures data (anthropometric, admission diagnosis, provenience, comorbidities, disability, symptoms, effort tolerance, disease impact, length of stay and discharge destinations) over 1 year period (March 2020-March 2021) was undertaken and compared to retrospective data from a corresponding 1 year (March 2019-March 2020) period to determine the impact of the network relocation on the delivery of pulmonary specialist rehabilitation to patients with complex needs during the pandemic episode. One of the changes implemented at the respiratory Maugeri network was the relocation of the Pulmonary Rehabilitation units from its 351 beds base to a repurposed 247 beds and a reduction in total number of admitted patients (n=3912 in pre-COVID time; n=2089 in post-COVID time). All respiratory diagnosis, except COVID sequelae, decreased (chronic respiratory failure-CRF, COPD, obstructive sleep apnoea syndrome-OSAS, interstitial lung disease-ILD, tracheostomized patients and other mixed diseases decreased of 734, 705, 157, 87, 79 and 326 units, respectively). During the pandemic time, 265 post COVID sequelae with CRF were admitted for rehabilitation (12.62%), percentage of patients coming from acute hospital increased, LOS and NIV use remained stable while CPAP indication decreased. Disease impact, dyspnoea and effort tolerance as their improvements after rehabilitation, were similar in the two periods. Only baseline disability, expressed by Barthel index, seems higher in the 2 observation time as its improvement. Hospital deaths and transfers to acute hospitals were higher during pandemic crisis while home destination decreased. This review demonstrated impact of coronavirus pandemic situation, specifically the relocation of the respiratory inpatient rehabilitation wards in a huge Italian network.

Copyright © the Author(s), 2021.

PMID

34935324 [<https://www.ncbi.nlm.nih.gov/pubmed/?term=34935324>]

Status

Embase

Institution

(Vitacca, Ceriana, Balbi, Bruschi, Aliani, Maniscalco, Fanfulla, Diasparra, Rizzello, Sereni, Spanevello) Department of Respiratory Rehabilitation, ICS Maugeri IRCCS, Pavia, Italy

Publisher

Page Press Publications

Year of Publication

2021

Link to the Ovid Full Text or citation:

[Click here for full text options](https://ovidsp.ovid.com/ovidweb.cgi?T=JS&CSC=Y&NEWS=N&PAGE=fulltext&D=emed22&AN=2016416772)

Link to the External Link Resolver:

[LibKey NHS](https://libkey.io/libraries/2789/openurl?genre=article&sid=OVID:emed22&genre=article&id=pmid:34935324&id=doi:10.4081%2Fmonaldi.2021.1843&issn=1122-0643&volume=91&issue=4&spage=1843&pages=&date=2021&title=Monaldi+Archives+for+Chest+Disease&atitle=The+respiratory+rehabilitation+Maugeri+network+service+reconfiguration+after+1+year+of+COVID-19&aulast=Vitacca)

25.

Epilogue to contemporary perspectives in COPD: New Horizons.

Jenkins C.R.

Embase

Respirology. 26(8) (pp 742-744), 2021. Date of Publication: August 2021.

[Editorial]

AN: 2013001138

PMID

34184367 [<https://www.ncbi.nlm.nih.gov/pubmed/?term=34184367>]

Status

Embase

Author NameID

Jenkins, Christine R.; ORCID: <https://orcid.org/0000-0003-2717-5647>

Institution

(Jenkins) Respiratory Group, The George Institute for Global Health, Sydney, NSW, Australia (Jenkins) Faculty of Medicine, UNSW Sydney, NSW, Australia

(Jenkins) Concord Clinical School, University of Sydney, Sydney, NSW, Australia

Publisher

John Wiley and Sons Inc

Year of Publication

2021

Link to the Ovid Full Text or citation:

[Click here for full text options](https://ovidsp.ovid.com/ovidweb.cgi?T=JS&CSC=Y&NEWS=N&PAGE=fulltext&D=emed22&AN=2013001138)

Link to the External Link Resolver:

[LibKey NHS](https://libkey.io/libraries/2789/openurl?genre=article&sid=OVID:emed22&genre=article&id=pmid:34184367&id=doi:10.1111%2Fresp.14105&issn=1323-7799&volume=26&issue=8&spage=742&pages=742-744&date=2021&title=Respirology&atitle=Epilogue+to+contemporary+perspectives+in+COPD%3A+New+Horizons&aulast=Jenkins)

26.

Self-reported sleep disturbance and mild cognitive impairment in COPD patients with severe airflow limitation.

Barata P.I., Marc M.S., Tudorache E., Frandes M., Crisan A.F., Olar D.C., Oancea C.

Embase

Clinical Respiratory Journal. 15(7) (pp 808-814), 2021. Date of Publication: July 2021.

[Article]

AN: 2010902577

Introduction: COPD has multiple extrapulmonary manifestations and the latest studies have focused on cognitive dysfunction effects on sleep quality. The purpose of this study is to assess if there is a relationship between sleep quality and cognitive decline in COPD patients with severe airflow limitation.

Method(s): We performed an observational study to determine if there is a link between cognitive function and sleep quality. The included patients were divided into two groups: a group with COPD patients and control group. We evaluated lung volumes, cognitive function, sleep quality and disease impact on the quality of life.

Result(s): Most of the COPD patients presented mild cognitive impairment (MCI) (95.7%), compared with only 24.1% in the control group (Pearson chi-square chi2(1) = 42.560, p < 0.001). We observed that all the COPD patients were poor sleepers, while only 13.8% of the control patients presented a poor sleep (Pearson chi-square chi2(1) = 60.379, p < 0.001). We observed that poor sleep was significantly associated with MCI (OR = 9.200; 95% CI = 3.656-23.153; p < 0.001). At the same time, when considering only the COPD patients with moderate disease impact, poor sleep was also a risk factor for MCI (OR = 1.210; 95% CI = 1.016-1.440; p < 0.001).

Conclusion(s): COPD patients with severe airflow limitation report a high prevalence of poor sleep quality and cognitive function. We observed a significant association between cognitive function and sleep quality.

Copyright © 2021 John Wiley & Sons Ltd

PMID

33749073 [<https://www.ncbi.nlm.nih.gov/pubmed/?term=33749073>]

Status

Embase

Author NameID

Marc, Monica Steluta; ORCID: <https://orcid.org/0000-0003-1160-9164> Oancea, Cristian; ORCID: <https://orcid.org/0000-0003-2083-0581>

Institution

(Barata, Olar) Faculty of Medicine, Department of Physiology, "Vasile Goldis" University of Arad, Arad, Romania (Marc, Tudorache, Crisan, Oancea) Department of Pulmonology, University of Medicine and Pharmacy "Victor Babes" Timisoara, Timisoara, Romania

(Frandes) Department of Biostatistics and Medical Informatics, University of Medicine and Pharmacy "Victor Babes" Timisoara, Timisoara, Romania

Publisher

John Wiley and Sons Inc

Year of Publication

2021

Link to the Ovid Full Text or citation:

[Click here for full text options](https://ovidsp.ovid.com/ovidweb.cgi?T=JS&CSC=Y&NEWS=N&PAGE=fulltext&D=emed22&AN=2010902577)

Link to the External Link Resolver:

[LibKey NHS](https://libkey.io/libraries/2789/openurl?genre=article&sid=OVID:emed22&genre=article&id=pmid:33749073&id=doi:10.1111%2Fcrj.13366&issn=1752-6981&volume=15&issue=7&spage=808&pages=808-814&date=2021&title=Clinical+Respiratory+Journal&atitle=Self-reported+sleep+disturbance+and+mild+cognitive+impairment+in+COPD+patients+with+severe+airflow+limitation&aulast=Barata)

27.

Step-up and step-down treatment approaches for COPD: A holistic view of progressive therapies.

Lopez-Campos J.L., Carrasco Hernandez L., Ruiz-Duque B., Reinoso-Arija R., Caballero-Eraso C.

Embase

International Journal of COPD. 16 (pp 2065-2076), 2021. Date of Publication: 2021.

[Review]

AN: 2007879742

Recent advances in inhaled drugs and a clearer definition of the disease have made the task of managing COPD more complex. Different proposals have been put forward which combine all the available treatments and the different clinical presentations in an effort to select the best therapeutic options for each clinical context. As COPD is a chronic progressive disease, the escalation of therapy has traditionally been considered the most natural way to tackle it. However, the notion of COPD as a constantly progressing disease has recently been challenged and, in specific areas, this points to the possibility of a deescalation in treatment. In this context, the clinician requires simple, specific recommendations to guide these changes in treatment in their daily clinical practice. To accomplish this, the first step must be a correct evaluation and an accurate initial preliminary diagnosis of the patient's condition. Thereafter, the first escalation in therapy must be introduced with caution as the disease progresses, since clinical trials are not designed with clinical decision-making in mind. During this escalation, three possibilities are open to change the current treatment for a different one within the same family, to increase non-pharmacological interventions or to increase the pharmacological therapies. Beyond that point, a patient with persistent symptoms represents a complex clinical scenario which requires a specialized approach, including the evaluation of different respiratory and non-respiratory comorbidities. Unfortunately, there are few de-escalation studies available, and these are mainly observational in nature. The debate on de-escalation in pharmacological treatment, therefore, involves two main discussion points: the withdrawal of bronchodilators and the withdrawal of inhaled steroids. Altogether, the scheme for modifying treatment must be more personalized than just adding molecules, and the therapeutic response and its conditioning factors should be evaluated at each step before proceeding further.

Copyright © 2021 Lopez-Campos et al.

PMID

34285480 [<https://www.ncbi.nlm.nih.gov/pubmed/?term=34285480>]

Status

Embase

Institution

(Lopez-Campos, Carrasco Hernandez, Ruiz-Duque, Reinoso-Arija, Caballero-Eraso) Unidad Medico-Quirurgica de Enfermedades Respiratorias, Instituto de Biomedicina de Sevilla (IBiS), Hospital Universitario Virgen del Rocio/ Universidad de Sevilla, Seville, Spain (Lopez-Campos, Carrasco Hernandez, Ruiz-Duque, Reinoso-Arija, Caballero-Eraso) Centro de Investigacion Biomedica en Red de Enfermedades Respiratorias (CIBERES), Instituto de Salud Carlos III, Madrid, Spain

Publisher

Dove Medical Press Ltd

Year of Publication

2021

Link to the Ovid Full Text or citation:

[Click here for full text options](https://ovidsp.ovid.com/ovidweb.cgi?T=JS&CSC=Y&NEWS=N&PAGE=fulltext&D=emed22&AN=2007879742)

Link to the External Link Resolver:

[LibKey NHS](https://libkey.io/libraries/2789/openurl?genre=article&sid=OVID:emed22&genre=article&id=pmid:34285480&id=doi:10.2147%2FCOPD.S275943&issn=1176-9106&volume=16&issue=&spage=2065&pages=2065-2076&date=2021&title=International+Journal+of+COPD&atitle=Step-up+and+step-down+treatment+approaches+for+COPD%3A+A+holistic+view+of+progressive+therapies&aulast=Lopez-Campos)

28.

Extra-pulmonary manifestations of COPD and the role of pulmonary rehabilitation: a symptom-centered approach.

Machado A., Marques A., Burtin C.

Embase

Expert Review of Respiratory Medicine. 15(1) (pp 131-142), 2021. Date of Publication: 2021.

[Review]

AN: 2007563547

Introduction: Chronic obstructive pulmonary disease (COPD) is a complex and heterogenous disease that is associated with a range of respiratory and non-respiratory symptoms, which highly contribute to the daily burden of the disease. Symptoms burden remains high despite optimal bronchodilator therapy, but pulmonary rehabilitation (PR) is an effective intervention to improve patients' symptoms. A comprehensive interdisciplinary approach within the framework of a PR program is warranted to tackle these complex symptoms and their consequences. Areas covered: This narrative review describes how symptoms of dyspnea, fatigue, cough, sputum, anxiety, depression, pain, sleep disturbances, and cognitive decline arise in COPD and can contribute to several non-pulmonary manifestations of the disease. It also describes evidence of the effectiveness of interdisciplinary PR programs to counteract these symptoms. A literature search was performed on PubMed and Scopus between June and July 2020. Expert opinion: Respiratory and non-respiratory symptoms are highly prevalent, often not comprehensively assessed, and result in several extra-pulmonary manifestations of the disease (physical, emotional and social). Interdisciplinary PR programs can improve these negative manifestations through different pathways, contributing for an effective symptoms' management. A thorough assessment of symptoms (beyond dyspnea) should be routinely performed and may support the identification of treatable traits, allowing the tailoring of PR interventions and assessment of their real-life impact.

Copyright © 2020 Informa UK Limited, trading as Taylor & Francis Group.

PMID

33225762 [<https://www.ncbi.nlm.nih.gov/pubmed/?term=33225762>]

Status

Embase

Author NameID

Machado, Ana; ORCID: <https://orcid.org/0000-0002-4427-2695>

Institution

(Machado, Marques) Respiratory Research and Rehabilitation Laboratory (Lab 3R), School of Health Sciences (ESSUA), University of Aveiro, Aveiro, Portugal (Machado, Marques) Institute of Biomedicine (Ibimed), University of Aveiro, Aveiro, Portugal

(Burtin) REVAL - Rehabilitation Research Center, Faculty of Rehabilitation Sciences, Hasselt University, Diepenbeek, Belgium

(Burtin) BIOMED - Biomedical Research Institute, Hasselt University, Diepenbeek, Belgium

Publisher

Taylor and Francis Ltd.

Year of Publication

2021

Link to the Ovid Full Text or citation:

[Click here for full text options](https://ovidsp.ovid.com/ovidweb.cgi?T=JS&CSC=Y&NEWS=N&PAGE=fulltext&D=emed22&AN=2007563547)

Link to the External Link Resolver:

[LibKey NHS](https://libkey.io/libraries/2789/openurl?genre=article&sid=OVID:emed22&genre=article&id=pmid:33225762&id=doi:10.1080%2F17476348.2021.1854737&issn=1747-6348&volume=15&issue=1&spage=131&pages=131-142&date=2021&title=Expert+Review+of+Respiratory+Medicine&atitle=Extra-pulmonary+manifestations+of+COPD+and+the+role+of+pulmonary+rehabilitation%3A+a+symptom-centered+approach&aulast=Machado)

29.

Ers international congress 2020 virtual: Highlights from the allied respiratory professionals assembly.

Smith E., Thomas M., Calik-Kutukcu E., Torres-Sanchez I., Granados-Santiago M., Quijano-Campos J.C., Sylvester K., Burtin C., Sajnic A., De Brandt J., Cruz J.

Embase

ERJ Open Research. 7(1) (pp 1-14), 2021. Article Number: 00808-2020. Date of Publication: 2021.

[Article]

AN: 2006072678

This article provides an overview of outstanding sessions that were (co)organised by the Allied Respiratory Professionals Assembly during the European Respiratory Society International Congress 2020, which this year assumed a virtual format. The content of the sessions was mainly targeted at allied respiratory professionals, including respiratory function technologists and scientists, physiotherapists, and nurses. Short take-home messages related to spirometry and exercise testing are provided, highlighting the importance of quality control. The need for quality improvement in sleep interventions is underlined as it may enhance patient outcomes and the working capacity of healthcare services. The promising role of digital health in chronic disease management is discussed, with emphasis on the value of end-user participation in the development of these technologies. Evidence on the effectiveness of airway clearance techniques in chronic respiratory conditions is provided along with the rationale for its use and challenges to be addressed in future research. The importance of assessing, preventing and reversing frailty in respiratory patients is discussed, with a clear focus on exercise-based interventions. Research on the impact of disease-specific fear and anxiety on patient outcomes draws attention to the need for early assessment and intervention. Finally, advances in nursing care related to treatment adherence, self-management and patients' perspectives in asthma and chronic obstructive pulmonary disease are provided, highlighting the need for patient engagement and shared decision making. This highlights article provides readers with valuable insight into the latest scientific data and emerging areas affecting clinical practice of allied respiratory professionals.

Copyright © ERS 2021.

Status

Embase

Institution

(Smith) Children's Lung Health, Wal-Yan Respiratory Centre, Telethon Kids Institute, Perth, Australia (Thomas) Cardiopulmonary Exercise Testing Service, University Hospitals Birmingham, Birmingham, United Kingdom

(Calik-Kutukcu) Faculty of Physical Therapy and Rehabilitation, Hacettepe University, Ankara, Turkey

(Torres-Sanchez) Dept of Physical Therapy, Faculty of Health Sciences, University of Granada, Granada, Spain

(Granados-Santiago) Dept of Nursing, Faculty of Health Sciences, University of Granada, Granada, Spain

(Quijano-Campos) Research and Development, Royal Papworth Hospital NHS Foundation Trust, Cambridge Biomedical Campus, Cambridge, United Kingdom

(Sylvester) Respiratory Physiology, Royal Papworth and Cambridge University Hospitals NHS Foundation Trusts, Cambridge, United Kingdom

(Burtin, De Brandt) REVAL - Rehabilitation Research Center, BIOMED - Biomedical Research Institute, Faculty of Rehabilitation Sciences, Hasselt University, Diepenbeek, Belgium

(Sajnic) Dept for Respiratory Diseases Jordanovac, University Hospital Center, Zagreb, Croatia

(Cruz) Center for Innovative Care and Health Technology (ciTechCare), School of Health Sciences (ESSLei), Polytechnic of Leiria, Leiria, Portugal

Publisher

European Respiratory Society

Year of Publication

2021

Link to the Ovid Full Text or citation:

[Click here for full text options](https://ovidsp.ovid.com/ovidweb.cgi?T=JS&CSC=Y&NEWS=N&PAGE=fulltext&D=emed22&AN=2006072678)

Link to the External Link Resolver:

[LibKey NHS](https://libkey.io/libraries/2789/openurl?genre=article&sid=OVID:emed22&genre=article&id=pmid:&id=doi:10.1183%2F23120541.00808-2020&issn=2312-0541&volume=7&issue=1&spage=1&pages=1-14&date=2021&title=ERJ+Open+Research&atitle=Ers+international+congress+2020+virtual%3A+Highlights+from+the+allied+respiratory+professionals+assembly&aulast=Smith)

30.

Utility of self-administered questionnaires for identifying individuals at risk of COPD in Japan: The OCEAN (Okinawa COPD casE finding assessmeNt) study.

Tamaki K., Sakihara E., Miyata H., Hirahara N., Kirichek O., Tawara R., Akiyama S., Katsumata M., Haruya M., Ishii T., Simard E.P., Miller B.E., Tal-Singer R., Kaise T.

Embase

International Journal of COPD. 16 (pp 1771-1782), 2021. Date of Publication: 2021.

[Article]

AN: 2007667760

Purpose: A considerable proportion of patients with chronic obstructive pulmonary disease (COPD) remain undiagnosed and untreated even though they may have a burden of respiratory symptoms that impact quality of life. The OCEAN study assessed the ability of screening questionnaires to identify individuals with, or at risk of, COPD by comparing questionnaire outcomes with spirometric measures of lung function.

Method(s): This observational study included participants >=40 years of age presenting for their annual health examination at a single medical center in Okinawa, Japan. Participants completed COPD screening questionnaires (CAPTURE and COPD-Q), the Chronic Airways Assessment Test (CAAT), and general demographic and health-related questionnaires. The performance characteristics of CAPTURE and COPD-Q were compared with spirometry-based airflow limitation by calculating the area under the receiver operating characteristic (ROC-AUC) curve.

Result(s): A total of 2518 participants were included in the study; 79% of whom were <60 years of age (mean 52.0 years). A total of 52 (2.1%) participants had airflow limitation defined as forced expiratory volume in 1 second (FEV1)/forced vital capacity (FVC) <0.7, and 420 (16.7%) participants were classified as Preserved Ratio Impaired Spirometry (PRISm). Among participants with PRISm, 75 (17.9%) had a CAAT total score >=10. Airflow limitation and PRISm were more prevalent in current smokers versus past smokers. For the CAPTURE questionnaire, ROC-AUC for screening airflow limitation, PRISm, and PRISm with a CAAT total score >=10 were 0.59, 0.55, and 0.69, respectively; for COPD-Q, these three clinical features were 0.67, 0.58 and 0.68, respectively.

Conclusion(s): This study demonstrated that CAPTURE and COPD-Q appear to be effective screening tools for identifying symptomatic individuals with undiagnosed, or at risk of developing COPD in adults >=40 years of age in Okinawa. Furthermore, early diagnosis and management of PRISm is important to improve future outcomes and the societal burden of disease.

Copyright © 2021 Tamaki et al.

PMID

34168439 [<https://www.ncbi.nlm.nih.gov/pubmed/?term=34168439>]

Status

Embase

Institution

(Tamaki) Department of Breast Surgery, Nahanishi Clinic, Okinawa, Japan (Sakihara) Lifestyle Related Disease Medical Center, Naha Medical Association, Okinawa, Japan

(Miyata, Hirahara) Health Policy and Management, School of Medicine, Keio University, Tokyo, Japan

(Kirichek) Value Evidence and Outcomes, GSK, Stockley Park, United Kingdom

(Tawara, Akiyama, Katsumata, Kaise) Japan Development, GSK, Tokyo, Japan

(Haruya) Government Affairs and Market Access, GSK, Tokyo, Japan

(Ishii) Medical Japan, GSK, Tokyo, Japan

(Simard, Miller, Tal-Singer) Value Evidence and Outcomes, GSK, Collegeville, PA, United States

Publisher

Dove Medical Press Ltd

Year of Publication

2021

Link to the Ovid Full Text or citation:

[Click here for full text options](https://ovidsp.ovid.com/ovidweb.cgi?T=JS&CSC=Y&NEWS=N&PAGE=fulltext&D=emed22&AN=2007667760)

Link to the External Link Resolver:

[LibKey NHS](https://libkey.io/libraries/2789/openurl?genre=article&sid=OVID:emed22&genre=article&id=pmid:34168439&id=doi:10.2147%2FCOPD.S302259&issn=1176-9106&volume=16&issue=&spage=1771&pages=1771-1782&date=2021&title=International+Journal+of+COPD&atitle=Utility+of+self-administered+questionnaires+for+identifying+individuals+at+risk+of+COPD+in+Japan%3A+The+OCEAN+(Okinawa+COPD+casE+finding+assessmeNt)+study&aulast=Tamaki)

31.

Incorporating remote patient monitoring in virtual pulmonary rehabilitation programs.

Jangalee J.V., Ghasvareh P., Guenette J.A., Road J.

Embase

Canadian Journal of Respiratory Therapy. 57 (pp 83-89), 2021. Date of Publication: 2021.

[Article]

AN: 2014003194

Most pulmonary rehabilitation (PR) programs have had to adapt due to the COVID-19 pandemic and associated restrictions. Current alternative home-based programs have limitations and require modification. In this paper, we outline a novel method to monitor home-based PR programs, which has the potential to improve PR safety and efficacy. This new method is based on a remote patient monitoring (RPM) system with connected smart devices that enables the Respiratory Therapist (RT) to have real-time access to patient data including heart rate and peripheral oxygen saturation during exercise. The RPM system also monitors daily physical activity, sedentary time, sleep quality, rescue inhaler use, and maintenance inhaler adherence, among other variables, which has the added advantage of predicting patterns consistent with symptoms that may require medical intervention. To increase privacy, data are anonymized at all levels and only the RT has access to patient information. RPM systems have the potential to give practitioners a holistic view of the participants' health status to better evaluate them during the entire PR program and to improve self-management. As this is not a formal research study, we cannot make definitive conclusions about the efficacy of the system, and further research is needed to examine safety and to compare our approach to other ways of conducting PR.

Copyright © 2021 Canadian Society of Respiratory Therapists. All rights reserved.

Status

Embase

Institution

(Jangalee) Vancouver General Hospital Pulmonary Rehabilitation Program, Vancouver, BC, Canada (Ghasvareh) Agartee Technology Inc., Vancouver, BC, Canada

(Guenette) Centre for Heart and Lung Innovation, Providence Health Care Research Institute and The University of British Columbia, St. Paul's Hospital, Vancouver, BC, Canada

(Guenette) Department of Physical Therapy, Faculty of Medicine, The University of British Columbia, Vancouver, BC, Canada

(Road) The Lung Centre at Vancouver General Hospital, Vancouver, BC, Canada

Publisher

Canadian Society of Respiratory Therapists

Year of Publication

2021

Link to the Ovid Full Text or citation:

[Click here for full text options](https://ovidsp.ovid.com/ovidweb.cgi?T=JS&CSC=Y&NEWS=N&PAGE=fulltext&D=emed22&AN=2014003194)

Link to the External Link Resolver:

[LibKey NHS](https://libkey.io/libraries/2789/openurl?genre=article&sid=OVID:emed22&genre=article&id=pmid:&id=doi:10.29390%2Fcjrt-2021-015&issn=1205-9838&volume=57&issue=&spage=83&pages=83-89&date=2021&title=Canadian+Journal+of+Respiratory+Therapy&atitle=Incorporating+remote+patient+monitoring+in+virtual+pulmonary+rehabilitation+programs&aulast=Jangalee)

32.

Remote-Management of COPD: Evaluating the Implementation of Digital Innovation to Enable Routine Care (RECEIVER): The protocol for a feasibility and service adoption observational cohort study.

Taylor A., Lowe D.J., McDowell G., Lua S., Burns S., McGinness P., Carlin C.M.

Embase

BMJ Open Respiratory Research. 8(1) (no pagination), 2021. Article Number: e000905. Date of Publication: 30 Aug 2021.

[Article]

AN: 635867949

Introduction Reductions in exacerbation and hospitalisations are the outcomes rated as most important by people with chronic obstructive pulmonary disease (COPD). Most COPD management is currently based on a reactive approach, and delays in recognising treatable opportunities underpin COPD care quality gaps. Innovations that empower COPD self-management, facilitate integrated clinical care and support delivery of evidence-based treatment interventions are urgently required. Methods and analysis The Remote-Management of COPD: Evaluating the Implementation of Digital Innovation to Enable Routine Care trial is a prospective observational cohort hybrid implementation and effectiveness study that will explore the adoption of a digital service model for people with a high-risk' COPD and evaluate the feasibility of this approach versus current standards of care. People with COPD, who have had recent severe exacerbation and/or COPD-obstructive sleep apnoea overlap or chronic hypercapnic respiratory failure requiring home non-invasive ventilation (NIV) or continuous positive airway pressure (CPAP), with internet access will be recruited into the study and enrolled into the digital service. Study endpoints will examine participant utilisation, clinical service impact and clinical outcomes compared with historical and contemporary control patient data. The digital infrastructure will also provide a foundation to explore the feasibility of approaches to predict outcomes and exacerbation in people with COPD through machine learning analysis. Ethics and dissemination Ethical approval for this clinical trial has been obtained from the West of Scotland Research Ethics Service. The trial will commence in September 2019 for a duration of 2 years. Results will be presented at local, national and international meetings, as well as submission for publication to peer-reviewed journals.

Copyright © Author(s) (or their employer(s)) 2021. Re-use permitted under CC BY. Published by BMJ.

PMID

34462271 [<https://www.ncbi.nlm.nih.gov/pubmed/?term=34462271>]

Status

Embase

Institution

(Taylor, McDowell, Lua, Carlin) Respiratory Medicine, Queen Elizabeth University Hospital, Glasgow, United Kingdom (Lowe) Emergency Medicine, Queen Elizabeth University Hospital, Glasgow, United Kingdom

(Burns, McGinness) Lenus Digital Health, StormID, Edinburgh, United Kingdom

Publisher

BMJ Publishing Group

Clinical Trial Number

<https://clinicaltrials.gov/show/04240353>

Year of Publication

2021

Link to the Ovid Full Text or citation:

[Click here for full text options](https://ovidsp.ovid.com/ovidweb.cgi?T=JS&CSC=Y&NEWS=N&PAGE=fulltext&D=emed22&AN=635867949)

Link to the External Link Resolver:

[LibKey NHS](https://libkey.io/libraries/2789/openurl?genre=article&sid=OVID:emed22&genre=article&id=pmid:34462271&id=doi:10.1136%2Fbmjresp-2021-000905&issn=2052-4439&volume=8&issue=1&spage=e000905&pages=&date=2021&title=BMJ+Open+Respiratory+Research&atitle=Remote-Management+of+COPD%3A+Evaluating+the+Implementation+of+Digital+Innovation+to+Enable+Routine+Care+(RECEIVER)%3A+The+protocol+for+a+feasibility+and+service+adoption+observational+cohort+study&aulast=Taylor)

33.

Factors of vascular endothelial dysfunction and thrombogenic risk in patients with chronic obstructive pulmonary disease in combination with obstructive sleep apnea after an exacerbation. B akTopbl pcka ekx epod yBbobHblx coeTaH dcyHk oce obocTpeH xpoHecko c cHdpoMoM HdoTe obcTpykTBHo obcTpykTBHoo cocydoB TpoMboeHHoo boe3Hbyu aHo cHa <B akTopbl pcka ekx epod yBbobHblx coeTaH dcyHk oce obocTpeH xpoHecko c cHdpoMoM HdoTe obcTpykTBHo obcTpykTBHoo cocydoB TpoMboeHHoo boe3Hbyu aHo cHa.>

Tseymakh I.Ya., Shoykhet Y.N.

Embase

Pulmonologiya. 31(3) (pp 329-337), 2021. Date of Publication: 2021.

[Article]

AN: 2013300153

Obstructive sleep apnea (OSA) is a common disease. The incidence is higher in patients with chronic obstructive pulmonary disease with moderate to severe bronchial obstruction or hypoxemia. OSA is associated with increased risks of fatal outcomes of acute cardiovascular diseases in such patients. Objective. To assess the effect of long-term non-invasive ventilation of the lungs in the spontaneous breathing mode with positive airway pressure (CPAP) on the indicators of systemic inflammation, insulin resistance, and thrombogenic risk together with clinical outcomes during the rehabilitation period after an exacerbation in patients with concomitant chronic obstructive pulmonary disease and obstructive sleep apnea who do not need long-term oxygen therapy. Methods. The effects of long-term CPAP therapy as a part of complex treatment were analyzed in an open-label, prospective, comparative six-week study that enrolled 65 patients with concomitant chronic obstructive pulmonary disease and obstructive sleep apnea. The main group included 26 people who received CPAP therapy as a part of complex treatment. The comparison group included 39 people who did not use non-invasive ventilation of the lungs. The patients' age was 55.5 +/- 2.1 years in the main group and 57.1 +/- 1.5 years in the comparison group (p > 0.1). Men prevailed in both groups - 92.3% in the main group and 100.0% in the comparison group (p > 0.1). Results. The clinical efficacy of CPAP therapy was confirmed by an improvement in the quality of life of patients on the SF-36 questionnaire, a decrease in the degree of daytime sleepiness on the Epworth scale (Johns, 1991). CPAP-therapy was associated with a drop in the serum levels of tumor necrosis factor alpha and the blood level of endothelin 1, a more pronounced decrease in the levels of C-reactive protein, interleukin 8, C-peptide, vascular endothelial growth factor, homocysteine versus the comparison group. Conclusion. The use of CPAP-therapy in patients with concomitant chronic obstructive pulmonary disease and obstructive sleep apnea who do not need long-term oxygen therapy during the rehabilitation period after an exacerbation is associated with a decrease in systemic inflammation, vascular endothelial dysfunction, and hyperhomocysteinemia.

Copyright © 2021 Medical Education. All rights reserved.

Status

Embase

Author NameID

Shoykhet, Yakov N.; ORCID: <https://orcid.org/0000-0002-5253-4325>

Institution

(Tseymakh, Shoykhet) Altay State Medical University, Healthcare Ministry of Russia, pr. Lenina 40, Altay Region, Barnaul 656060, Russian Federation

Publisher

Medical Education

Year of Publication

2021

Link to the Ovid Full Text or citation:

[Click here for full text options](https://ovidsp.ovid.com/ovidweb.cgi?T=JS&CSC=Y&NEWS=N&PAGE=fulltext&D=emed22&AN=2013300153)

Link to the External Link Resolver:

[LibKey NHS](https://libkey.io/libraries/2789/openurl?genre=article&sid=OVID:emed22&genre=article&id=pmid:&id=doi:10.18093%2F0869-0189-2021-31-3-329-337&issn=0869-0189&volume=31&issue=3&spage=329&pages=329-337&date=2021&title=Pulmonologiya&atitle=B+akTopbl+pcka+ekx+epod+yBbobHblx+coeTaH+dcyHk+oce+obocTpeH+xpoHecko+c+cHdpoMoM+HdoTe+obcTpykTBHo+obcTpykTBHoo+cocydoB++TpoMboeHHoo+boe3Hbyu+aHo+cHa&aulast=Tseymakh)

34.

COPD Management during the COVID-19 pandemic.

Salvi S., Dhar R., Mahesh P., Udwadia Z., Behra D.

Embase

Lung India. 38(7 Supplement 1) (pp S80-S85), 2021. Date of Publication: March 2021.

[Note]

AN: 634474347

Status

Embase

Institution

(Salvi) Department of Clinical Research, Pulmocare Research and Education (PURE) Foundation, Pune, India (Dhar) Department of Respiratory Medicine, National Allergy Asthma Bronchitis Institute, Kolkota, West Bengal, India

(Mahesh) Department of Respiratory Medicine, Jss Medical College, Mysuru, Karnataka, India

(Udwadia) Department of Respiratory Medicine, Hinduja Hospital and Research Centre, Mumbai, Maharashtra, India

(Behra) Department of Pulmonary Medicine, Pgimer, Chandigarh, India

Publisher

Wolters Kluwer Medknow Publications

Year of Publication

2021

Link to the Ovid Full Text or citation:

[Click here for full text options](https://ovidsp.ovid.com/ovidweb.cgi?T=JS&CSC=Y&NEWS=N&PAGE=fulltext&D=emed22&AN=634474347)

Link to the External Link Resolver:

[LibKey NHS](https://libkey.io/libraries/2789/openurl?genre=article&sid=OVID:emed22&genre=article&id=pmid:&id=doi:10.4103%2Flungindia.lungindia-685-20&issn=0970-2113&volume=38&issue=7+Supplement+1&spage=S80&pages=S80-S85&date=2021&title=Lung+India&atitle=COPD+Management+during+the+COVID-19+pandemic&aulast=Salvi)

35.

Other options usually better than benzodiazepines in alleviating dyspnoea, sleeping problems and anxiety in COPD patients.

Fenton C., Kang C.

Embase

Drugs and Therapy Perspectives. 37(1) (pp 19-24), 2021. Date of Publication: January 2021.

[Review]

AN: 2006996281

Chronic obstructive pulmonary disease (COPD) is common, especially in older people, and causes progressive dyspnoea that is often accompanied by anxiety and depression. Benzodiazepines (BZDs) are regularly prescribed to COPD patients but can cause significant respiratory adverse events. There are usually better treatment alternatives, such as pulmonary rehabilitation and treatment with antidepressants. The increasing prescription of BZDs with age is concerning, particularly as COPD becomes more prevalent.

Copyright © 2020, Springer Nature Switzerland AG.

Status

Embase

Institution

(Fenton, Kang) Springer Nature, Private Bag 65901, Mairangi Bay, Auckland 0754, New Zealand

Publisher

Adis

Year of Publication

2021

Link to the Ovid Full Text or citation:

[Click here for full text options](https://ovidsp.ovid.com/ovidweb.cgi?T=JS&CSC=Y&NEWS=N&PAGE=fulltext&D=emed22&AN=2006996281)

Link to the External Link Resolver:

[LibKey NHS](https://libkey.io/libraries/2789/openurl?genre=article&sid=OVID:emed22&genre=article&id=pmid:&id=doi:10.1007%2Fs40267-020-00787-x&issn=1172-0360&volume=37&issue=1&spage=19&pages=19-24&date=2021&title=Drugs+and+Therapy+Perspectives&atitle=Other+options+usually+better+than+benzodiazepines+in+alleviating+dyspnoea%2C+sleeping+problems+and+anxiety+in+COPD+patients&aulast=Fenton)

36.

COPD assessment test for the evaluation of COVID-19 symptoms.

Daynes E., Gerlis C., Briggs-Price S., Jones P., Singh S.J.

Embase

Thorax. 76(2) (pp 185-187), 2021. Date of Publication: 01 Feb 2021.

[Article]

AN: 633377309

There is evidence to demonstrate the ongoing symptoms of COVID-19; however, there are currently no agreed outcomes to assess these symptoms. This study examined the use of the chronic obstructive pulmonary disease (COPD) assessment test (CAT) for patients recovering from COVID-19. 131 patients who were admitted with COVID-19 were followed up over the phone to assess symptoms. The median (IQR) CAT score was 10 (5-16). Cough, phlegm and chest tightness domains were within range for healthy people, but there was evidence of significant breathlessness, loss of energy, and activity and sleep disturbance. The CAT is a useful tool to assess symptoms of COVID-19 recovery.

Copyright © Author(s) (or their employer(s)) 2021. No commercial re-use. See rights and permissions. Published by BMJ.

PMID

33148809 [<https://www.ncbi.nlm.nih.gov/pubmed/?term=33148809>]

Status

Embase

Institution

(Daynes, Gerlis, Briggs-Price, Singh) Cers, Nihr Leicester Biomedical Research Centre Respiratory Diseases, Leicester, East Midlands, United Kingdom (Daynes, Singh) Department of Respiratory Sciences, University of Leicester, Leicester Leicestershire, United Kingdom

(Jones) Institute of Infection and Immunology, University of London, London, United Kingdom

Publisher

BMJ Publishing Group

Year of Publication

2021

Link to the Ovid Full Text or citation:

[Click here for full text options](https://ovidsp.ovid.com/ovidweb.cgi?T=JS&CSC=Y&NEWS=N&PAGE=fulltext&D=emed22&AN=633377309)

Link to the External Link Resolver:

[LibKey NHS](https://libkey.io/libraries/2789/openurl?genre=article&sid=OVID:emed22&genre=article&id=pmid:33148809&id=doi:10.1136%2Fthoraxjnl-2020-215916&issn=0040-6376&volume=76&issue=2&spage=185&pages=185-187&date=2021&title=Thorax&atitle=COPD+assessment+test+for+the+evaluation+of+COVID-19+symptoms&aulast=Daynes)

37.

LONG-TERM IMPACT OF PULMONARY REHABILITATION ON SLEEP IN COPD PATIENTS AS MEASURED BY ACTIGRAPHY.

Mak J., Ellstrom K., Band Mani Benedict S., Thapamagar S.

Embase

Chest. Conference: CHEST 2021 Annual Meeting. Virtual, Online. 160(4 Supplement) (pp A2175), 2021. Date of Publication: October 2021.

[Conference Abstract]

AN: 2014930266

TOPIC: Pulmonary Rehabilitation TYPE: Original Investigations PURPOSE: Chronic obstructive pulmonary disease (COPD) is associated with poor sleep quality. Compared to the general population, COPD patients have delayed sleep initiation, impaired sleep maintenance, increased nightmares, and excessive daytime sleepiness. Poor sleep negatively affects functional capacity, peripheral muscle strength, and health-related quality of life (HRQL), and is associated with increased COPD exacerbations and mortality. Pulmonary rehabilitation (PR) has been shown to improve daytime function, HRQL, and subjective sleep quality in COPD. It has also been shown to improve sleep subjectively but not objectively, as measured by actigraphy in short duration (8-12 weeks). Long-term effects of PR on sleep have not been studied well. We hypothesized that actigraphy would demonstrate subjective and objective improvement in sleep with long-term exercise following completion of PR.

METHOD(S): This was a retrospective study of COPD patients who completed PR at Veteran's Affairs Loma Linda from 2013 to 2020. Participants underwent 8 weeks of structured PR and 12 months of unstructured exercise program (home and in-center). Actigraphic watch recordings before PR, after 8 weeks of PR, and 12 months after completion of PR were taken. Data were collected on sleep variables, including total time in bed (TBT), total sleep time (TST), sleep onset latency (SOL), sleep efficiency (SE), wakefulness after sleep onset (WASO), and total nocturnal awakenings. Data on spirometry, six-minute walk distance (6MWD), Pittsburgh Sleep Quality Index (PSQI), St. George's Respiratory Questionnaire (SGRQ), and modified Medical Research Council Dyspnea (mMRC) score were also collected. Paired comparisons (t-tests) were performed to assess the impact of PR on sleep variables.

RESULT(S): Out of 98 participants enrolled for PR during the study period, 16 completed 12-month post-PR assessments including actigraphy and were included in the final analysis. All participants were male with an average age of 72 +/- 8 yrs and more than two-thirds (69%) had severe COPD (GOLD stage 3 or 4). Details of subjective and objective sleep outcomes are in the table. There was sustained improvement in 6MWD (mean difference, 68.8m, 95% CI -1.3 to 139, p=0.05) and SGRQ (mean difference, - 8.1 points, 95% CI-16.3 to 0.1, p=0.05), as well as stable mMRC score (mean difference, -0.4 points, -1.2 to 0.5, p=0.37). However, objective sleep variables from actigraphy worsened (TBT, TST) or did not improve (SOL, SE, WASO, Awakenings). PSQI did not improve significantly after 12 months post-PR (mean change, -1.3, 95% CI -3.1 to 0.5, p=0.14).

CONCLUSION(S): Pulmonary rehabilitation with an unstructured exercise program after completion of 8 weeks of structured program was not associated with qualitative or quantitative improvements in sleep. Further studies are needed to evaluate whether long-term structured exercise programs following PR could improve sleep in COPD patients. CLINICAL IMPLICATIONS: Sleep is significantly impaired in patients with COPD. Poor sleep can lead to increased cognitive impairment, cardiovascular disease, COPD exacerbations, and mortality. Sleep quality in COPD patients has not been shown to improve after completion of pulmonary rehabilitation. However, long-term structured exercise programs following completion of pulmonary rehabilitation may improve sleep quality in this population. DISCLOSURES: No relevant relationships by Shawn Band Mani Benedict, source=Web Response No relevant relationships by Kathleen Ellstrom, source=Web Response No relevant relationships by Joseph Mak, source=Admin input No relevant relationships by Suman Thapamagar, source=Web Response

Copyright © 2021 American College of Chest Physicians

Status

CONFERENCE ABSTRACT

Publisher

Elsevier Inc.

Year of Publication

2021

Link to the Ovid Full Text or citation:

[Click here for full text options](https://ovidsp.ovid.com/ovidweb.cgi?T=JS&CSC=Y&NEWS=N&PAGE=fulltext&D=emed22&AN=2014930266)

Link to the External Link Resolver:

[LibKey NHS](https://libkey.io/libraries/2789/openurl?genre=article&sid=OVID:emed22&genre=article&id=pmid:&id=doi:10.1016%2Fj.chest.2021.07.1921&issn=0012-3692&volume=160&issue=4+Supplement&spage=A2175&pages=A2175&date=2021&title=Chest&atitle=LONG-TERM+IMPACT+OF+PULMONARY+REHABILITATION+ON+SLEEP+IN+COPD+PATIENTS+AS+MEASURED+BY+ACTIGRAPHY&aulast=Mak)

38.

COMPARING QUALITY OF LIFE IN ADEQUATELY AND INADEQUATELY OXYGENATED PATIENTS WITH EXERTION WITH VARYING PULMONARY DISEASES: A RETROSPECTIVE REVIEW.

Tahlor A., Washburn T., LaForme Fiss A., Miller C.

Embase

Chest. Conference: CHEST 2021 Annual Meeting. Virtual, Online. 160(4 Supplement) (pp A2347), 2021. Date of Publication: October 2021.

[Conference Abstract]

AN: 2014929350

TOPIC: Respiratory Care TYPE: Original Investigations PURPOSE: The primary aim of this study was to compare the quality of life (QoL) between: 1) adequately oxygenated (SpO2 88% and above) and inadequately oxygenated (SpO2 < 88%) patients with exertion, and 2) patient disease state. A secondary aim attempted to determine the contribution of several patient demographic and 6-minute walk test variables to quality of life as measured by the St. George Respiratory Questionnaire (SGRQ) symptom, activity, life impact, and total scores among patients presenting to a pulmonary rehab program.

METHOD(S): All patient charts from Piedmont Pulmonary and Sleep Medicine's pulmonary rehabilitation program from 2013-2017 (n=421), which contained a satisfactorily completed SGRQ and a 6-minute walk test, were retrospectively analyzed. Patients were classified by the following disease states: 1) asthma (n=23), 2) bronchiectasis (n=6), 3) interstitial lung disease (n=58) including patients with idiopathic pulmonary fibrosis, pulmonary fibrosis, sarcoidosis, and cryptogenic organizing pneumonia, 4) primary pulmonary hypertension (n=7), 5) restrictive lung disease (n=4), 6) stage 2 COPD (n=115), 7) stage 3 COPD (n=129), 8) stage 4 COPD (n=49), and 9) other (n=30) which included patients with chronic respiratory failure with hypoxia, lung cancer, structural emphysema, lung transplantation, and dyspnea. Correlation coefficients were calculated between each of the SGRQ categories and patient oxygenation state. Stepwise regression was used to determine the contribution of 6-minute walk test distance, supplemental O2 flowrate, age, gender, pulmonary disease process, highest reported Borg score (dyspnea) with exertion, and nadir SpO2 with exertion to QoL.

RESULT(S): No significant correlations existed between patient oxygenation state (adequately [n=264] or inadequately [n=157]) and the symptom, activity, life impact, or total SGRQ scores. Similarly, no significant correlations were noted between patient disease state and the various SGRQ scores. The main contributors to variation in SGRQ total score were highest reported Borg score (dyspnea), patient age, 6-minute walk test distance, and nadir SpO2 (totaling 25.3% variation) with the highest reported Borg score accounting for the largest variation among these parameters at 14.2%.

CONCLUSION(S): Neither the adequacy of oxygenation or pulmonary disease state showed significant correlations with either symptom, activity, life impact or total SGRQ scores. The highest reported Borg score (dyspnea) accounts for the largest single contributor to total SGRQ score. CLINICAL IMPLICATIONS: Pulmonologists might be better served to emphasize a patient's highest reported Borg score with exertion for those patients who are inadequately oxygenated (and, thus, qualify for supplemental oxygen) when determining what type of oxygen delivery system that patient should use. DISCLOSURES: No relevant relationships by Alyssa LaForme Fiss, source=Web Response No relevant relationships by Chad Miller, source=Web Response, value=Consulting fee Removed 04/27/2021 by Chad Miller, source=Web Response No relevant relationships by Chad Miller, source=Web Response, value=Honoraria Removed 04/27/2021 by Chad Miller, source=Web Response No relevant relationships by Chad Miller, source=Web Response, value=Honoraria Removed 04/27/2021 by Chad Miller, source=Web Response No relevant relationships by Adam Tahlor, source=Web Response No relevant relationships by Timothy Washburn, source=Web Response

Copyright © 2021 American College of Chest Physicians

Status

CONFERENCE ABSTRACT

Publisher

Elsevier Inc.

Year of Publication

2021

Link to the Ovid Full Text or citation:

[Click here for full text options](https://ovidsp.ovid.com/ovidweb.cgi?T=JS&CSC=Y&NEWS=N&PAGE=fulltext&D=emed22&AN=2014929350)

Link to the External Link Resolver:

[LibKey NHS](https://libkey.io/libraries/2789/openurl?genre=article&sid=OVID:emed22&genre=article&id=pmid:&id=doi:10.1016%2Fj.chest.2021.07.2037&issn=0012-3692&volume=160&issue=4+Supplement&spage=A2347&pages=A2347&date=2021&title=Chest&atitle=COMPARING+QUALITY+OF+LIFE+IN+ADEQUATELY+AND+INADEQUATELY+OXYGENATED+PATIENTS+WITH+EXERTION+WITH+VARYING+PULMONARY+DISEASES%3A+A+RETROSPECTIVE+REVIEW&aulast=Tahlor)

39.

Pulmonary Rehabilitation and Its Impact on Sleep Quality: A Prospective Analysis.

Khalid F., Krishnan V.

Embase

American Journal of Respiratory and Critical Care Medicine. Conference: American Thoracic Society International Conference, ATS 2021. Virtual. 203(9) (no pagination), 2021. Date of Publication: May 2021.

[Conference Abstract]

AN: 635310002

Rationale: Poor quality sleep is common in patients with heart failure, chronic obstructive pulmonary disease (COPD) and other chronic cardiopulmonary disorders. In patients with COPD and heart failure, cardiac and pulmonary rehabilitation (CRPR) has important health benefits such as improvement in symptoms, exercise tolerance and quality of life. However, the short-term and sustained effects of CRPR on sleep quality are controversial. We hypothesize that CRPR results in improved sleep quality in patients with chronic lung disease and heart failure.

Method(s): The primary outcome measure is the change in individual Pittsburgh sleep quality index scores at the beginning, end and 3 months after CRPR. Patients will also be screened for sleep apnea prior to starting pulmonary rehabilitation using Berlin questionnaire. Secondary outcome measures include change in the hospital anxiety and depression scale, insomnia severity index, COPD assessment test (for COPD patients), Kansas-city cardiomyopathy questionnaire (for heart failure patients) at the beginning, end and 3-months after rehabilitation. Paired-sample Student's t-tests will be used to compare means before and after the rehabilitation program, as well as comparisons between cardiac and pulmonary patients. Pearson's product co-efficient (r) and linear regression models will be used to analyze association between changes in Pittsburgh sleep quality index and the secondary outcomes. The minimum clinically important difference (MCID) for each questionnaire, as reported in the literature, will be used to detect significant change before and after cardiac and pulmonary rehabilitation. Sample size calculations with assumptions of prior standard deviation of 4.6, 90% power, Type 1 error of 0.05, and true difference in response of matched pairs of 3 or more, then 27 subjects are needed to reject the null hypothesis. We propose to recruit 27 pulmonary and 27 cardiac patients for this study.

Result(s): Baseline characteristics of 6 subjects recruited so far in the study are shown in the Table 1. Subjects were primarily COPD patients with moderate to severe disease, primarily females.

Conclusion(s): CRPR may play an important role in improving sleep quality in the short-term and 3-months after rehabilitation. Additional recruitment to the calculated sample size will allow further analysis of the effect of CRPR.

Status

CONFERENCE ABSTRACT

Institution

(Khalid) Pulmonary, Critical Care and Sleep Medicine, Case Western Reserve University, Metrohealth Campus, Cleveland, OH, United States (Krishnan) MetroHealth Medical Center, Cleveland, OH, United States

Publisher

American Thoracic Society

Year of Publication

2021

Link to the Ovid Full Text or citation:

[Click here for full text options](https://ovidsp.ovid.com/ovidweb.cgi?T=JS&CSC=Y&NEWS=N&PAGE=fulltext&D=emed22&AN=635310002)

Link to the External Link Resolver:

[LibKey NHS](https://libkey.io/libraries/2789/openurl?genre=article&sid=OVID:emed22&genre=article&id=pmid:&id=doi:10.1164%2Fajrccm-conference.2021.TP104&issn=1535-4970&volume=203&issue=9&spage=&pages=&date=2021&title=American+Journal+of+Respiratory+and+Critical+Care+Medicine&atitle=Pulmonary+Rehabilitation+and+Its+Impact+on+Sleep+Quality%3A+A+Prospective+Analysis&aulast=Khalid)

40.

Copd care program: Reducing copd readmissions.

Villamizar J.P., De Diego A., Schweitzer M.D., Aboubkar M., Kosseifi S.

Embase

American Journal of Respiratory and Critical Care Medicine. Conference: American Thoracic Society International Conference, ATS 2021. Virtual. 203(9) (no pagination), 2021. Date of Publication: May 2021.

[Conference Abstract]

AN: 635308525

Introduction: COPD is not only the third leading cause of death in the US, but also a main cause of morbidity associated with a significant economic burden. Approximately one fifth of all COPD patients are readmitted within 30 days following discharge. Additionally, readmission costs are on average 18% more expensive than initial stays. A number of strategies have been identified to reduce the severity and frequency of exacerbations. Nonetheless, in many cases the application of such strategies is either inadequate or lacks proper guidance. This prompted the Centers for Medicare and Medicaid Services (CMS) back in 2014 to dictate that a 30-day unplanned readmission following hospitalization for a COPD exacerbation will be penalized with reduced reimbursement. We aimed to quantify the effect of a comprehensive COPD care program that could potentially reduce COPD readmissions in the first 30 days following an acute exacerbation.

Method(s): COPD exacerbations requiring hospitalizations were identified daily for a year (June 2018-June 2019) at Holy Cross Hospital (Fort Lauderdale, Fl) utilizing a COPD nurse navigator. COPD navigators would provide an initial assessment of patients, which included CAT score, proper inhaler technique, medication accessibility, pulmonology follow up, sleep disorder screening and tobacco cessation. If agreed, patients were then referred to the outpatient COPD clinic upon discharge. At the clinic patients were managed on a weekly basis for one month by internal medicine residents and attendings. At the clinic patients also had access to a respiratory therapist who would asses for weekly spirometry, CAT score, 6 minute walk test, proper inhaler technique and medication supply. Pulmonology, pulmonary rehabilitation, tobacco cessation and PFTs referrals were provided accordingly. Patients having multiple exacerbations during the one-year period were counted only as 1.

Result(s): 335 patients were hospitalized for COPD exacerbations. Males were 49.53% of the patients with a mean age of 75.62 years (+/-16.66) and females were 50.46% with a mean age 77.24 years (+/-12.19). 66 patients were followed up by their regular pulmonologist, meanwhile 20 were followed up at the outpatient COPD clinic. A total of 39 (11.6%) patients were readmitted during the first year of the intervention, compared to 60 (16.5%) patients in the prior year. This yielded a 31.3% reduction in readmissions in comparison the prior year. Sub analysis of patients followed in the COPD clinic showed that only one (5%) of them was readmitted. Percentage risk adjustment also decreased from 20.4% to 14.4%.

Status

CONFERENCE ABSTRACT

Institution

(Villamizar, De Diego, Schweitzer, Aboubkar) Internal Medicine, University of Miami at Holy Cross Hospital, Fort Lauderdale, FL, United States (Kosseifi) Division of Pulmonary and Critical Care, Holy Cross Hospital, Fort Lauderdale, FL, United States

Publisher

American Thoracic Society

Year of Publication

2021

Link to the Ovid Full Text or citation:

[Click here for full text options](https://ovidsp.ovid.com/ovidweb.cgi?T=JS&CSC=Y&NEWS=N&PAGE=fulltext&D=emed22&AN=635308525)

Link to the External Link Resolver:

[LibKey NHS](https://libkey.io/libraries/2789/openurl?genre=article&sid=OVID:emed22&genre=article&id=pmid:&id=doi:10.1164%2Fajrccm-conference.2021.203.1_MeetingAbstracts.A1698&issn=1535-4970&volume=203&issue=9&spage=&pages=&date=2021&title=American+Journal+of+Respiratory+and+Critical+Care+Medicine&atitle=Copd+care+program%3A+Reducing+copd+readmissions&aulast=Villamizar)

41.

Secondary polycythemia vera may be a clue for primary care physicians to start oxygen supplementation in chronic obstructive pulmonary disease.

Wang G.C., Nayak R.P.

Embase

American Journal of Respiratory and Critical Care Medicine. Conference: American Thoracic Society International Conference, ATS 2021. Virtual. 203(9) (no pagination), 2021. Date of Publication: May 2021.

[Conference Abstract]

AN: 635307673

Supplemental oxygen is used by over 1.5 million adults in the United States with respiratory disorders, including chronic obstructive pulmonary disease (COPD), which affects approximately 32 million individuals in the United States, and is the sixth leading cause of death worldwide. Although long-term supplemental oxygen is recommended for patients with chronic hypoxemia (PaO2 <= 55 mm Hg or SpO2 <= 88%), as this case illustrates, supplemental oxygen is not always prescribed when indicated, suggesting external challenges in prescribing. A 61-year-old male with a >110 pack-year smoking history and an eight year diagnosis of COPD presented to the pulmonary clinic following a hospitalization for pneumonia. Hemoglobin was 19.7 g/dL and hematocrit was 58.2 g/dL. Since initial diagnosis, the patient reported progressively worsening dyspnea on exertion and at rest, as well as frequent wheezing. The patient never had an acute exacerbation requiring steroids or antibiotics, emergency department visits or hospitalizations. He used a beclomethasone inhaler regularly and albuterol when necessary. In the pulmonary clinic, exercise pulse oximetry evaluation showed a baseline oxygen saturation of 94% at rest, with desaturation to 86% after walking in the hallway for three minutes. Pulmonary Function Testing (PFT) confirmed the diagnosis of very severe COPD with FEV1 of 0.94 L (32% of predicted) and DLCO of 48% predicted with moderate air trapping. Compared to the patient's prior PFT eight years prior, the FEV1 had decreased by 820 mL. The patient was initiated on fluticasone-umeclidinium-vilanterol, and as needed albuterol. He was prescribed oxygen 2 L with activity and sleep and referred for pulmonary rehabilitation. The patient quit smoking in order to be eligible for lung transplantation consideration. Two months after starting oxygen supplementation, hemoglobin and hematocrit had improved to 16.2 g/dL and 50.2 g/dL, respectively. The patient had never been considered for nor trialed on supplemental oxygen for his persistent dyspnea. Although significant literature exists on the effects of supplemental oxygen on patients' quality of life and improvement in survival, primary care physicians must be aware of the known consequences of chronic hypoxia, including secondary polycythemia and cor pulmonale, and also the importance of serial PFT to assess airflow limitation and progression of the COPD. Timely interventions will improve management of the disease burden, leading to improved quality of life. Primary care physicians are in need of guidance from the pulmonary community to develop a standardized COPD treatment algorithm and a system to assess and reassess oxygen need.

Status

CONFERENCE ABSTRACT

Institution

(Wang) Internal Medicine, Saint Louis University School of Medicine, St. Louis, MO, United States (Nayak) St Louis Univ, Saint Louis, MO, United States

Publisher

American Thoracic Society

Year of Publication

2021

Link to the Ovid Full Text or citation:

[Click here for full text options](https://ovidsp.ovid.com/ovidweb.cgi?T=JS&CSC=Y&NEWS=N&PAGE=fulltext&D=emed22&AN=635307673)

Link to the External Link Resolver:

[LibKey NHS](https://libkey.io/libraries/2789/openurl?genre=article&sid=OVID:emed22&genre=article&id=pmid:&id=doi:10.1164%2Fajrccm-conference.2021.203.1_MeetingAbstracts.A2382&issn=1535-4970&volume=203&issue=9&spage=&pages=&date=2021&title=American+Journal+of+Respiratory+and+Critical+Care+Medicine&atitle=Secondary+polycythemia+vera+may+be+a+clue+for+primary+care+physicians+to+start+oxygen+supplementation+in+chronic+obstructive+pulmonary+disease&aulast=Wang)

42.

Care for patients with advanced chronic obstructive pulmonary disease.

Harrison A.

Embase

Respirology. Conference: TSANZSRS 2021 Australia and New Zealand Society of Respiratory Science and the Thoracic Society of Australia and New Zealand Annual Scientific Meeting, ANZSRS/TSANZ. Virtual. 26(SUPPL 2) (pp 145), 2021. Date of Publication: April 2021.

[Conference Abstract]

AN: 635067731

Introduction/Aim: Patients with advanced chronic obstructive pulmonary disease (COPD) experience a high symptom burden with significant comorbidities. Their management often necessitates a multi-disciplinary approach, with both pharmacological and non-pharmacological approaches to treatment of their respiratory disease. Our study evaluated the respiratory management and care needs of patients with advanced COPD attending the Oxygen Service at a tertiary centre in Melbourne.

Method(s): We retrospectively analyzed the medical records of patients who were prescribed domiciliary oxygen therapy for COPD between January 2012 and December 2019 at Austin Health. Data collected included patient demographics, comorbidities, physiologic parameters, and respiratory management. A random sample was selected for this preliminary analysis.

Result(s): Of the 263 patients included (mean age 75.0 years, 123 female, FEV1 46.6% predicted), thirty two had an overlap diagnosis of interstitial lung disease. Common comorbidities were cardiac disease (58%) pulmonary hypertension (52%), and sleep disordered breathing and hypoventilation syndrome (36%). The mean Charlson comorbidity index score was 6.2 (SD 2.2). 166 (63%) patients were referred to pulmonary rehabilitation. Hospital Admission Risk Program and allied health services including dietician, occupational therapy, speech pathology and social work were involved in the management of 145 (55%) patients. 249 patients were prescribed ambulatory oxygen therapy, eight nocturnal oxygen therapy and 107 long-term oxygen therapy. In terms of inhaler therapies, 193 (73%) patients were prescribed "triple therapy" including inhaled corticosteroid, long-acting beta-2-agonist and long-acting muscarinic antagonist. Only 20% of patients were referred to palliative care services.

Conclusion(s): Patients with advanced COPD have high levels of comorbidity and require multidisciplinary team management. The majority of patients received appropriate disease-specific pharmacological therapies, although referrals to pulmonary rehabilitation, a strongly evidence based intervention, was suboptimal and only a fifth of patients had been referred to palliative care services.

Status

CONFERENCE ABSTRACT

Institution

(Harrison) Austin Health, Preston, Australia

Publisher

Blackwell Publishing

Year of Publication

2021

Link to the Ovid Full Text or citation:

[Click here for full text options](https://ovidsp.ovid.com/ovidweb.cgi?T=JS&CSC=Y&NEWS=N&PAGE=fulltext&D=emed22&AN=635067731)

Link to the External Link Resolver:

[LibKey NHS](https://libkey.io/libraries/2789/openurl?genre=article&sid=OVID:emed22&genre=article&id=pmid:&id=doi:10.1111%2Fresp.14022&issn=1323-7799&volume=26&issue=SUPPL+2&spage=145&pages=145&date=2021&title=Respirology&atitle=Care+for+patients+with+advanced+chronic+obstructive+pulmonary+disease&aulast=Harrison)

43.

Should you recommend inhaled corticosteroids for this patient with chronic obstructive pulmonary disease?: Grand rounds discussion from beth Israel deaconess medical center.

Burns R.B., Anandaiah A., Rice M.B., Smetana G.W.

Embase

Annals of Internal Medicine. 172(11) (pp 735-742), 2020. Date of Publication: 02 Jun 2020.

[Review]

AN: 2015542292

Approximately 12 million adults in the United States receive a diagnosis of chronic obstructive pulmonary disease (COPD) each year, and it is the fourth leading cause of death. Chronic obstructive pulmonary disease refers to a group of diseases that cause airflow obstruction and a constellation of symptoms, including cough, sputum production, and shortness of breath. The main risk factor for COPD is tobacco smoke, but other environmental exposures also may contribute. The GOLD (Global Initiative for Chronic Obstructive Lung Disease) 2020 Report aims to provide a nonbiased review of the current evidence for the assessment, diagnosis, and treatment of patients with COPD. To date, no conclusive evidence exists that any existing medications for COPD modify mortality. The mainstay of treatment for COPD is inhaled bronchodilators, whereas the role of inhaled corticosteroids is less clear. Inhaled corticosteroids have substantial risks, including an increased risk for pneumonia. Here, 2 experts, both pulmonologists, reflect on the care of a woman with severe COPD, a 50-pack-year smoking history, frequent COPD exacerbations, and recurrent pneumonia. They consider the indications for inhaled corticosteroids in COPD, when inhaled corticosteroids should be withdrawn, and what other treatments are available.

Copyright © 2020 American College of Physicians

PMID

32479149 [<https://www.ncbi.nlm.nih.gov/pubmed/?term=32479149>]

Status

Embase

Institution

(Burns, Anandaiah, Rice, Smetana) Beth Israel Deaconess Medical Center, Boston, MA, United States

Publisher

American College of Physicians

Year of Publication

2020

Link to the Ovid Full Text or citation:

[Click here for full text options](https://ovidsp.ovid.com/ovidweb.cgi?T=JS&CSC=Y&NEWS=N&PAGE=fulltext&D=emed21&AN=2015542292)

Link to the External Link Resolver:

[LibKey NHS](https://libkey.io/libraries/2789/openurl?genre=article&sid=OVID:emed21&genre=article&id=pmid:32479149&id=doi:10.7326%2FM20-1058&issn=0003-4819&volume=172&issue=11&spage=735&pages=735-742&date=2020&title=Annals+of+Internal+Medicine&atitle=Should+you+recommend+inhaled+corticosteroids+for+this+patient+with+chronic+obstructive+pulmonary+disease%3F%3A+Grand+rounds+discussion+from+beth+Israel+deaconess+medical+center&aulast=Burns)

44.

Massive Traumatic Subcutaneous Emphysema.

Fernandes D., Pereira S., Guedes C., Silva D.

Embase

Acta medica (Hradec Kralove). 63(4) (pp 194-197), 2020. Date of Publication: 2020.

[Article]

AN: 633877864

74 year-old-man, former smoker, with chronic obstructive pulmonary disease GOLD grade 4, group D, with emphysema component, treated in a pulmonary rehabilitation program, on oxygen therapy and nocturnal bi-level positive airway pressure (BiPAP) ventilation. During the night he had a traumatic rib fracture (5-11th right ribs) but still he used BiPAP ventilation during the sleep. In the morning after he presented with a diffuse and massive emphysema in the face, thorax and abdominal regions. On physical examination, the patient presented with massive swelling and crepitus on palpation. A chest computed tomography (CT) scan confirmed a diffuse subcutaneous emphysema and revealed a mediastinal emphysema and bilateral small pneumothorax. A fast resolution of the emphysema was of paramount importance as the patient was severely agitated due to his inability to open both eyes, and the need to reintroduce BiPAP ventilation as soon as possible. It was placed a fenestrated subcutaneous catheter on left hemithorax and a subcutaneous ostomy on right hemithorax for comparative purpose. It was also performed a confluent centripetal massage towards drainage orifices, with immediate and substantial improvement of emphysema, especially in left hemithorax, and progressive ocular opening. Further emphysema absorption occurred during hospitalization.

PMID

33355081 [<https://www.ncbi.nlm.nih.gov/pubmed/?term=33355081>]

Institution

(Fernandes, Pereira, Guedes, Silva) Department of Internal Medicine, Centro Hospitalar Medio Ave, Vila Nova de Famalicao, Portugal

Publisher

NLM (Medline)

Year of Publication

2020

Link to the Ovid Full Text or citation:

[Click here for full text options](https://ovidsp.ovid.com/ovidweb.cgi?T=JS&CSC=Y&NEWS=N&PAGE=fulltext&D=emed21&AN=633877864)

Link to the External Link Resolver:

[LibKey NHS](https://libkey.io/libraries/2789/openurl?genre=article&sid=OVID:emed21&genre=article&id=pmid:33355081&id=doi:10.14712%2F18059694.2020.63&issn=1805-9694&volume=63&issue=4&spage=194&pages=194-197&date=2020&title=Acta+medica+(Hradec+Kralove)&atitle=Massive+Traumatic+Subcutaneous+Emphysema&aulast=Fernandes)

45.

Treatment of severe stable copd: The multidimensional approach of treatable traits.

van Dijk M., Gan C.T., Koster T.D., Wijkstra P.J., Slebos D.-J., Kerstjens H.A.M., van der Vaart H., Duiverman M.L.

Embase

ERJ Open Research. 6(3) (pp 1-15), 2020. Article Number: 00322-2019. Date of Publication: 2020.

[Review]

AN: 2005690750

Now that additional treatment options for severe chronic obstructive pulmonary disease (COPD) have emerged in recent years, patients with severe COPD should not be left in the rather hopeless situation of "there is nothing to improve" any more. Inertia or fatalism is a disservice to our patients. Ranging from advanced care planning to quite intense and demanding therapies such as multidisciplinary pulmonary rehabilitation, (endoscopic) lung volume reduction, chronic noninvasive ventilation and lung transplantation, caregivers should try to provide a personalised treatment for every severe COPD patient. In this review, we aim to describe the multidimensional approach to these patients at our centre along the lines of treatable traits leading to specific additional treatment modalities on top of standard care.

Copyright © ERS 2020.

Status

Embase

Institution

(van Dijk, Gan, Koster, Wijkstra, Slebos, Kerstjens, van der Vaart, Duiverman) Dept of Pulmonary Diseases, University of Groningen, University Medical Center Groningen, Groningen, Netherlands (van Dijk, Gan, Koster, Wijkstra, Slebos, Kerstjens, van der Vaart, Duiverman) Groningen Research Institute of Asthma and COPD (GRIAC), University of Groningen, Groningen, Netherlands

(van der Vaart) Centre of Rehabilitation Beatrixoord, Haren, University of Groningen, University Medical Center Groningen, Groningen, Netherlands

Publisher

European Respiratory Society

Year of Publication

2020

Link to the Ovid Full Text or citation:

[Click here for full text options](https://ovidsp.ovid.com/ovidweb.cgi?T=JS&CSC=Y&NEWS=N&PAGE=fulltext&D=emed21&AN=2005690750)

Link to the External Link Resolver:

[LibKey NHS](https://libkey.io/libraries/2789/openurl?genre=article&sid=OVID:emed21&genre=article&id=pmid:&id=doi:10.1183%2F23120541.00322-2019&issn=2312-0541&volume=6&issue=3&spage=1&pages=1-15&date=2020&title=ERJ+Open+Research&atitle=Treatment+of+severe+stable+copd%3A+The+multidimensional+approach+of+treatable+traits&aulast=van+Dijk)

46.

Gender does not impact the short-or long-term outcomes of home-based pulmonary rehabilitation in patients with copd.

Grosbois J.-M., Gephine S., Diot A.S., Kyheng M., Machuron F., Terce G., Wallaert B., Chenivesse C., Le Rouzic O.

Embase

ERJ Open Research. 6(4) (pp 1-10), 2020. Article Number: 00032-2020. Date of Publication: 01 Oct 2020.

[Article]

AN: 2005601609

Pulmonary rehabilitation, whether performed at home or in a specialised centre, is effective in the management of COPD. As gender-related differences in COPD were previously reported, we aimed to evaluate the impact of gender on pulmonary rehabilitation outcomes in the short, medium and long term. In this retrospective observational study of 170 women and 310 men with COPD, we compared the outcomes of an 8-week home-based pulmonary rehabilitation programme including an individualised plan of retraining exercises, physical activities, therapeutic education, and psychosocial and self-management support. Exercise tolerance, anxiety, depression, and quality of life (QOL) were assessed using the 6-min stepper test (6MST), Hospital Anxiety and Depression Scale (HADS) and Visual Simplified Respiratory Questionnaire (VSRQ), respectively. Assessments were carried out before, immediately after the pulmonary rehabilitation programme (T2) and at 8 (T8) and 14 months (T14). At baseline, women were younger (mean 62.1 versus 65.3 years), more often nonsmokers (24.7% versus 7.7%) and had a higher body mass index (28 versus 26.4 kg.m-2). They more often lived alone (50.6% versus 24.5%) and experienced social deprivation (66.7% versus 56.4%). They had significantly lower exercise tolerance (-34 strokes, 6MST) and higher anxiety and depression (+3.2 HADS total score), but there were no between gender differences in QOL (VSRQ). Both groups showed similar improvements in all outcome measures at T2, T8 and T14 with a tendency for men to lose QOL profits over time. Despite some differences in baseline characteristics, women and men with COPD had similar short-, medium-and long-term benefits of a home-based pulmonary rehabilitation programme.

Copyright © ERS 2020.

Status

Embase

Institution

(Grosbois) FormAction Sante, Perenchies, France (Grosbois, Terce) CH Bethune, Service de Pneumologie et Rehabilitation Respiratoire, Bethune, France

(Gephine) Univ. Lille, Univ. Artois, Univ. Littoral Cote D'opale, ULR 7369-Urepsss-Lille, Lille, France

(Gephine) Institut universitaire de cardiologie et de pneumologie de Quebec, Universite Laval, Quebec, Canada

(Diot, Wallaert, Chenivesse, Le Rouzic) CHU Lille, Service de Pneumologie et Immuno-Allergologie, Centre de Reference Constitutif des Maladies Pulmonaires Rares, Lille, France

(Kyheng, Machuron) CHU Lille, Department de Biostatistiques, Lille, France

(Kyheng, Machuron) Univ. Lille, CHU Lille, ULR 2694-METRICS: evaluation des technologies de sante et des pratiques medicales, Lille, France

(Wallaert, Chenivesse, Le Rouzic) Univ. Lille, Lille, France

(Wallaert, Chenivesse, Le Rouzic) Inserm, CNRS, Institut Pasteur de Lille, U1019-UMR 8204-CIIL-Centre for Infection and Immunity of Lille, Lille, France

Publisher

European Respiratory Society

Year of Publication

2020

Link to the Ovid Full Text or citation:

[Click here for full text options](https://ovidsp.ovid.com/ovidweb.cgi?T=JS&CSC=Y&NEWS=N&PAGE=fulltext&D=emed21&AN=2005601609)

Link to the External Link Resolver:

[LibKey NHS](https://libkey.io/libraries/2789/openurl?genre=article&sid=OVID:emed21&genre=article&id=pmid:&id=doi:10.1183%2F23120541.00032-2020&issn=2312-0541&volume=6&issue=4&spage=1&pages=1-10&date=2020&title=ERJ+Open+Research&atitle=Gender+does+not+impact+the+short-or+long-term+outcomes+of+home-based+pulmonary+rehabilitation+in+patients+with+copd&aulast=Grosbois)

47.

The Complex Relationship between Poor Sleep Quality and Chronic Obstructive Pulmonary Disease.

Hogan N., Cypro A., Malhotra A.

Embase

Clinical Pulmonary Medicine. 27(6) (pp 168-174), 2020. Date of Publication: November 2020.

[Article]

AN: 633929080

Sleep-related symptoms are prevalent among patients with chronic obstructive pulmonary disease (COPD). The disease process often manifests with nocturnal respiratory symptoms. Long-acting antimuscarinic medications improve nocturnal COPD symptoms, though their effect on sleep quality requires further investigation. Those with COPD often suffer from comorbidities that negatively impact sleep, including obstructive sleep apnea (OSA) and mood disorders such as anxiety and depression. Sleep quality is also predictive of COPD exacerbations. Patients with concurrent COPD and OSA suffer from overlap syndrome (OVS), characterized by a synergistic effect on poor health outcomes. The intersection of COPD and OSA offers the clinical pulmonary audience a useful lens for ongoing basic, clinical, and translational research. Patients with OVS experience higher mortality compared with either COPD or OSA alone. This observation is attributable to the compound effect each condition has on adverse cardiovascular events. A complex interplay exists between COPD, sleep symptoms, and OSA. COPD appears to influence important nonanatomical contributors to OSA. The presence of underlying COPD makes the definitive diagnosis of OSA a challenge. Chronic noninvasive ventilation (NIV) is the backbone of therapy for OVS, OSA, and hypercarbic COPD. NIV is additionally a well-established treatment for acute COPD exacerbations and emerging research demonstrates that NIV decreases mortality and hospitalizations in patients with hypercarbic COPD. Clinicians often need to individualize therapeutic interventions for patients with COPD, OSA, and OVS, balancing the benefits and adverse effects of such interventions. NIV can have unwanted impact on the quality of life for some patients with COPD. Certain medications used for COPD, such as corticosteroids, have adverse effects on sleep quality. Future therapeutic approaches are needed to improve the sleep symptoms and health outcomes of patients suffering from COPD and OVS.

Copyright © 2020 Lippincott Williams and Wilkins. All rights reserved.

Status

Embase

Institution

(Hogan, Cypro) Department of Medicine (Malhotra) Division of Pulmonary and Critical Care Medicine, University of California, San Diego, San diego, CA, United States

Publisher

Lippincott Williams and Wilkins

Year of Publication

2020

Link to the Ovid Full Text or citation:

[Click here for full text options](https://ovidsp.ovid.com/ovidweb.cgi?T=JS&CSC=Y&NEWS=N&PAGE=fulltext&D=emed21&AN=633929080)

Link to the External Link Resolver:

[LibKey NHS](https://libkey.io/libraries/2789/openurl?genre=article&sid=OVID:emed21&genre=article&id=pmid:&id=doi:10.1097%2FCPM.0000000000000384&issn=1068-0640&volume=27&issue=6&spage=168&pages=168-174&date=2020&title=Clinical+Pulmonary+Medicine&atitle=The+Complex+Relationship+between+Poor+Sleep+Quality+and+Chronic+Obstructive+Pulmonary+Disease&aulast=Hogan)

48.

An update on pulmonary rehabilitation techniques for patients with chronic obstructive pulmonary disease.

Wouters E.F.M., Posthuma R., Koopman M., Liu W.-Y., Sillen M.J., Hajian B., Sastry M., Spruit M., Franssen F.M.

Embase

Expert Review of Respiratory Medicine. (no pagination), 2020. Date of Publication: 2020.

[Review]

AN: 2004082563

Introduction: Pulmonary rehabilitation (PR) is one of the core components in the management of patients with chronic obstructive pulmonary disease (COPD). In order to achieve the maximal level of independence, autonomy, and functioning of the patient, targeted therapies and interventions based on the identification of physical, emotional and social traits need to be provided by a dedicated, interdisciplinary PR team. Areas covered: The review discusses cardiopulmonary exercise testing in the selection of different modes of training modalities. Neuromuscular electrical stimulation as well as gait assessment and training are discussed as well as add-on therapies as oxygen, noninvasive ventilator support or endoscopic lung volume reduction in selected patients. The potentials of pulsed inhaled nitric oxide in patients with underlying pulmonary hypertension is explored as well as nutritional support. The impact of sleep quality on outcomes of PR is reviewed. Expert opinion: Individualized, comprehensive intervention based on thorough assessment of physical, emotional, and social traits in COPD patients forms a continuous challenge for health-care professionals and PR organizations in order to dynamically implement and adapt these strategies based on dynamic, more optimal understanding of underlying pathophysiological mechanisms.

Copyright © 2020, © 2020 The Author(s). Published by Informa UK Limited, trading as Taylor & Francis Group.

PMID

31931636 [<https://www.ncbi.nlm.nih.gov/pubmed/?term=31931636>]

Status

Article-in-Press

Author NameID

Hajian, Bita; ORCID: <https://orcid.org/0000-0003-0453-968X>

Institution

(Wouters, Posthuma, Koopman, Sillen, Hajian, Sastry, Spruit, Franssen) Department of Respiratory Medicine, Maastricht University Medical Center+, Netherlands (Wouters, Posthuma, Liu, Spruit, Franssen) CIRO+, center of expertise for chronic organ failure, Horn, Netherlands

(Wouters, Koopman, Liu, Spruit, Franssen) NUTRIM School of Nutrition and Translational Research in Metabolism, Maastricht University Medical Center Maastricht, Netherlands

Publisher

Taylor and Francis Ltd.

Year of Publication

2020

Link to the Ovid Full Text or citation:

[Click here for full text options](https://ovidsp.ovid.com/ovidweb.cgi?T=JS&CSC=Y&NEWS=N&PAGE=fulltext&D=emed21&AN=2004082563)

Link to the External Link Resolver:

[LibKey NHS](https://libkey.io/libraries/2789/openurl?genre=article&sid=OVID:emed21&genre=article&id=pmid:31931636&id=doi:10.1080%2F17476348.2020.1700796&issn=1747-6348&volume=14&issue=2&spage=149&pages=&date=2020&title=Expert+Review+of+Respiratory+Medicine&atitle=An+update+on+pulmonary+rehabilitation+techniques+for+patients+with+chronic+obstructive+pulmonary+disease&aulast=Wouters)

49.

The Impact of Pulmonary Rehabilitation on 24-Hour Movement Behavior in People With Chronic Obstructive Pulmonary Disease: New Insights From a Compositional Perspective.

Burge A.T., Palarea-Albaladejo J., Holland A.E., Abramson M.J., McDonald C.F., Mahal A., Hill C.J., Lee A.L., Cox N.S., Lahham A., Moore R., Nicolson C., O'Halloran P., Gillies R., Chastin S.F.M.

Embase

Journal of physical activity & health. 18(1) (pp 13-20), 2020. Date of Publication: 11 Dec 2020.

[Article]

AN: 633749823

BACKGROUND: Physical activity levels are low in people with chronic obstructive pulmonary disease, and there is limited knowledge about how pulmonary rehabilitation transforms movement behaviors. This study analyzed data from a pulmonary rehabilitation trial and identified determinants of movement behaviors.

METHOD(S): Objectively assessed time in daily movement behaviors (sleep, sedentary, light-intensity physical activity, and moderate- to vigorous-intensity physical activity) from a randomized controlled trial (n = 73 participants) comparing home- and center-based pulmonary rehabilitation was analyzed using conventional and compositional analytical approaches. Regression analysis was used to assess relationships between movement behaviors, participant features, and response to the interventions.

RESULT(S): Compositional analysis revealed no significant differences in movement profiles between the home- and center-based groups. At end rehabilitation, conventional analyses identified positive relationships between exercise capacity (6-min walk distance), light-intensity physical activity, and moderate- to vigorous-intensity physical activity time. Compositional analyses identified positive relationships between a 6-minute walk distance and moderate- to vigorous-intensity physical activity time, accompanied by negative relationships with sleep and sedentary time (relative to other time components) and novel relationships between body mass index and light-intensity physical activity/sedentary time.

CONCLUSION(S): Compositional analyses following pulmonary rehabilitation identified unique associations between movement behaviors that were not evident in conventional analyses.

PMID

33307537 [<https://www.ncbi.nlm.nih.gov/pubmed/?term=33307537>]

Publisher

NLM (Medline)

Year of Publication

2020

Link to the Ovid Full Text or citation:

[Click here for full text options](https://ovidsp.ovid.com/ovidweb.cgi?T=JS&CSC=Y&NEWS=N&PAGE=fulltext&D=emed21&AN=633749823)

Link to the External Link Resolver:

[LibKey NHS](https://libkey.io/libraries/2789/openurl?genre=article&sid=OVID:emed21&genre=article&id=pmid:33307537&id=doi:10.1123%2Fjpah.2020-0322&issn=1543-5474&volume=18&issue=1&spage=13&pages=13-20&date=2020&title=Journal+of+physical+activity+%26+health&atitle=The+Impact+of+Pulmonary+Rehabilitation+on+24-Hour+Movement+Behavior+in+People+With+Chronic+Obstructive+Pulmonary+Disease%3A+New+Insights+From+a+Compositional+Perspective&aulast=Burge)

50.

High flow nasal cannula in acute hypercapnic exacerbation of chronic obstructive pulmonary disease: An emerging utility.

Mittal A., Varshney M., Rathi V., Ish P.

Embase

Monaldi Archives for Chest Disease. 90(1) (pp 9-12), 2020. Date of Publication: 2020.

[Article]

AN: 2004865282

High flow nasal cannula (HFNC) provides warmed and humidified air with flow rates up to 60 liters/min with relatively fixed oxygen content (FiO2). It has been extensively evaluated for hypoxemic respiratory failure and has been used in mild acute respiratory distress syndrome, pre-intubation, bronchoscopy and pediatric obstructive sleep apnea. Recent data has suggested a role in stable hypercapnic chronic obstructive pulmonary disease (COPD) and even in acute exacerbations, though, the use has not been advocated by any guidelines yet. We present a case of acute hypercapnic exacerbation of COPD, intolerant to non-invasive ventilation, showing response and improvement on use of HFNC. This case highlights this potential mechanisms and prospects for the same.

© Copyright the Author(s), 2020 Licensee PAGEPress, Italy

PMID

31970968 [<https://www.ncbi.nlm.nih.gov/pubmed/?term=31970968>]

Status

Embase

Institution

(Mittal, Rathi, Ish) Department of Pulmonary, Critical Care And Sleep Medicine, Vardhman Mahavir Medical College and Safdarjung Hospital, New Delhi, India (Varshney) Department of Medicine, Vardhman Mahavir Medical College And Safdarjung Hospital, New Delhi, India

Publisher

Page Press Publications

Year of Publication

2020

Link to the Ovid Full Text or citation:

[Click here for full text options](https://ovidsp.ovid.com/ovidweb.cgi?T=JS&CSC=Y&NEWS=N&PAGE=fulltext&D=emed21&AN=2004865282)

Link to the External Link Resolver:

[LibKey NHS](https://libkey.io/libraries/2789/openurl?genre=article&sid=OVID:emed21&genre=article&id=pmid:31970968&id=doi:10.4081%2Fmonaldi.2020.1158&issn=1122-0643&volume=90&issue=1&spage=9&pages=9-12&date=2020&title=Monaldi+Archives+for+Chest+Disease&atitle=High+flow+nasal+cannula+in+acute+hypercapnic+exacerbation+of+chronic+obstructive+pulmonary+disease%3A+An+emerging+utility&aulast=Mittal)

51.

Masterplan 2025 of the Austrian Society of Pneumology (ASP)-the expected burden and management of respiratory diseases in Austria. Masterplan 2025 der Osterreichischen Gesellschaft fur Pneumologie (OGP) - die erwartete Entwicklung und Versorgung respiratorischer Erkrankungen in Osterreich <Masterplan 2025 der Osterreichischen Gesellschaft fur Pneumologie (OGP) - die erwartete Entwicklung und Versorgung respiratorischer Erkrankungen in Osterreich.>

Studnicka M., Baumgartner B., Bolitschek J., Doberer D., Eber E., Eckmayr J., Hartl S., Hesse P., Jaksch P., Kink E., Kneussl M., Lamprecht B., Olschewski H., Pfleger A., Pohl W., Prior C., Puelacher C., Renner A., Steflitsch W., Stelzmuller I., Taubl H., Vonbank K., Wagner M., Wantke F., Wass R.

Embase

Wiener Klinische Wochenschrift. 132(Supplement 3) (pp 89-113), 2020. Date of Publication: 01 Sep 2020.

[Article]

AN: 2006821577

Scientific Members of the Austrian Society of Pneumology describe the expected development in respiratory health and provide guidance towards patient-oriented and cost-efficient respiratory care in Austria.

Method(s): In November 2017, respiratory care providers (physicians, nurses, physiotherapists) together with patient's advocacy groups and experts in health development, collaborated in workshops on: respiratory health and the environment, bronchial asthma and allergy, COPD, pediatric respiratory disease, respiratory infections, sleep disorders, interventional pneumology, thoracic oncology and orphan diseases.

Result(s): Respiratory disease is extremely prevalent and driven by ill-health behavior, i.e. cigarette smoking, over-eating and physical inactivity. For the majority of respiratory diseases increased prevalence, but decreased hospitalizations are expected. The following measures should be implemented to deal with future challenges: 1. Screening and case-finding should be implemented for lung cancer and COPD. 2. E-health solutions (telemedicine, personal apps) should be used to facilitate patient management. 3. Regional differences in respiratory care should be reduced through E-health and harmonization of health insurance benefits across Austria. 4. Patient education and awareness, to reduce respiratory health illiteracy should be increased, which is essential for sleep disorders but relevant also for other respiratory diseases. 5. Respiratory care should be inter-professional, provided via disease-specific boards beyond lung cancer (for ILDs, sleep, allergy) 6. Programs for outpatient's pulmonary rehabilitation can have a major impact on respiratory health. 7. Increased understanding of molecular pathways will drive personalized medicine, targeted therapy (for asthma, lung cancer) and subsequently health care costs.

Copyright © 2020, The Author(s).

PMID

32990821 [<https://www.ncbi.nlm.nih.gov/pubmed/?term=32990821>]

Status

Embase

Institution

(Studnicka) Landeskrankenhaus Salzburg, Universitatsklinik fur Pneumologie/Lungenheilkunde, Mullner Hauptstrase 48, Salzburg 5020, Austria (Studnicka) Paracelsus Medical University, Salzburg, Austria

(Baumgartner) Abteilung fur Pulmologie, Salzkammergut-Klinikum Vocklabruck, Vocklabruck, Austria

(Bolitschek) Abteilung fur Pneumologie, Ordensklinikum Linz GmbH Elisabethinen, Linz, Austria

(Doberer, Vonbank) Klin. Abteilung fur Pulmologie, Medizinische Universitat Wien, Wien, Austria

(Eber, Pfleger, Wagner) Univ.-Klinik fur Kinder- und Jugendheilkunde, Medizinische Universitat Graz, Graz, Austria

(Eckmayr) Abteilung fur Lungenkrankheiten, Klinikum Wels-Grieskirchen, Wels, Austria

(Hartl) 2. Interne Lungenabteilung, Otto Wagner-Spital, Wien, Austria

(Hesse) Ordination Dr. Judith & Dr. Peter Hesse, Schwechat, Austria

(Jaksch) Klin. Abteilung fur Thoraxchirurgie, Medizinische Universitat Wien, Wien, Austria

(Kink) Lungenabteilung, LKH Graz II - Standort Enzenbach, Gratwein-Strasengel, Austria

(Kneussl) ehem. 2. Medizinische Abteilung mit Pneumologie, Wilhelminenspital Wien, Wien, Austria

(Lamprecht, Wass) Klinik fur Lungenheilkunde, Kepler Universitatsklinikum, Linz, Austria

(Olschewski) Klinische Abteilung fur Pulmonologie, LKH-Univ. Klinikum Graz, Graz, Austria

(Pohl, Renner) Abteilung fur Atmungs- und Lungenerkrankungen, Krankenhaus Hietzing, Wien, Austria

(Prior) Ordination Univ.-Prof. Dr. Christian Prior, Innsbruck, Austria

(Puelacher) REHAMED-Tirol GmbH, Innsbruck, Austria

(Steflitsch) Wahlarzt-Ordination fur Lungenheilkunde, Ollersbach bei Neulengbach, Austria

(Stelzmuller) Ordination Doz. Dr. Ingrid Stelzmuller, Salzburg, Austria

(Taubl) Standort Natters, Pulmologie, LKH Hochzirl-Natters, Natters, Austria

(Wantke) FAZ Floridsdorfer Allergiezentrum GmbH, Wien, Austria

Publisher

Springer (E-mail: journals@springer.at)

Year of Publication

2020

Link to the Ovid Full Text or citation:

[Click here for full text options](https://ovidsp.ovid.com/ovidweb.cgi?T=JS&CSC=Y&NEWS=N&PAGE=fulltext&D=emed21&AN=2006821577)

Link to the External Link Resolver:

[LibKey NHS](https://libkey.io/libraries/2789/openurl?genre=article&sid=OVID:emed21&genre=article&id=pmid:32990821&id=doi:10.1007%2Fs00508-020-01722-w&issn=0043-5325&volume=132&issue=Supplement+3&spage=89&pages=89-113&date=2020&title=Wiener+Klinische+Wochenschrift&atitle=Masterplan+2025+der+Osterreichischen+Gesellschaft+fur+Pneumologie+(OGP)+-+die+erwartete+Entwicklung+und+Versorgung+respiratorischer+Erkrankungen+in+Osterreich&aulast=Studnicka)

52.

Management of the COPD patient with comorbidities: An experts recommendation document.

Iglesias J.R., Diez-Manglano J., Garcia F.L., Peromingo J.A.D., Almagro P., Aguilar J.M.V.

Embase

International Journal of COPD. 15 (pp 1015-1037), 2020. Date of Publication: 2020.

[Article]

AN: 2004309137

Background: Chronic obstructive pulmonary disease (COPD) is associated with multiple comorbidities, which impact negatively on patients and are often underdiagnosed, thus lacking a proper management due to the absence of clear guidelines.

Purpose(s): To elaborate expert recommendations aimed to help healthcare professionals to provide the right care for treating COPD patients with comorbidities.

Method(s): A modified RAND-UCLA appropriateness method consisting of nominal groups to draw up consensus recommendations (6 Spanish experts) and 2-Delphi rounds to validate them (23 Spanish experts) was performed.

Result(s): A panel of Spanish internal medicine experts reached consensus on 73 recommendations and 81 conclusions on the clinical consequences of the presence of comorbidities. In general, the experts reached consensus on the issues raised with regard to cardiovascular comorbidity and metabolic disorders. Consensus was reached on the use of selective serotonin reuptake inhibitors in cases of depression and the usefulness of referring patients with anxiety to respiratory rehabilitation programmes. The results also showed consensus on the usefulness of investigating the quality of sleep, the treatment of pain with opioids and the evaluation of osteoporosis by lateral chest radiography.

Conclusion(s): This study provides conclusions and recommendations that are intended to improve the management of the complexity of patients with COPD and important comorbidities, usually excluded from clinical trials.

Copyright © 2020 Recio Iglesias et al.

PMID

32440113 [<https://www.ncbi.nlm.nih.gov/pubmed/?term=32440113>]

Status

Embase

Institution

(Iglesias) Internal Medicine Department, Quironsalud Valencia Hospital, Valencian Community, Valencia, Spain (Diez-Manglano) Internal Medicine Department, Royo Villanova Hospital, Aragon, Zaragoza, Spain

(Garcia) Internal Medicine DepartmentGeneral University Hospital of Elche, Valencian Community, Alicante, Spain

(Peromingo) Internal Medicine Department, University Clinical Hospital of Santiago de Compostela, a Coruna, Galicia, Spain

(Almagro) Internal Medicine Department, Mutua Terrassa University Hospital, Catalonia, Terrassa, Barcelona, Spain

(Aguilar) Internal Medicine Department, University Hospital Virgen del Rocio, Andalusia, Seville, Spain

(Aguilar) CIBER of Epidemiology and Public Health, Madrid, Community of Madrid, Spain

Publisher

Dove Medical Press Ltd (E-mail: angela@dovepress.com)

Year of Publication

2020

Link to the Ovid Full Text or citation:

[Click here for full text options](https://ovidsp.ovid.com/ovidweb.cgi?T=JS&CSC=Y&NEWS=N&PAGE=fulltext&D=emed21&AN=2004309137)

Link to the External Link Resolver:

[LibKey NHS](https://libkey.io/libraries/2789/openurl?genre=article&sid=OVID:emed21&genre=article&id=pmid:32440113&id=doi:10.2147%2FCOPD.S242009&issn=1176-9106&volume=15&issue=&spage=1015&pages=1015-1037&date=2020&title=International+Journal+of+COPD&atitle=Management+of+the+COPD+patient+with+comorbidities%3A+An+experts+recommendation+document&aulast=Iglesias)

53.

The profile of copd patients with prolonged hospitalizations: A retrospective analysis of 200 hospital admissions.

El-Qader E.A., Israeli-Shani L., Shochet G.E., Dovrish Z., King D.A., Dahan D., Wand O., Shitrit D.

Embase

Israel Medical Association Journal. 22(10) (pp 552-556), 2020. Date of Publication: October 2020.

[Article]

AN: 2008446990

Background: Patients with severe chronic obstructive pulmonary disease (COPD) experience frequent exacerbations and need to be hospitalized, resulting in an economic and social burden. Although data exist regarding reasons of frequent hospitalizations, there is no data available about the impact on the length of stay (LOS).

Objective(s): To characterize the causes of prolonged hospitalizations in COPD patients.

Method(s): A retrospective study was conducted of patients who were diagnosed and treated in the pulmonary department for severe COPD exacerbations. All patient demographic data and medical history were collected. Data regarding the disease severity were also collected (including Global Initiative for Obstructive Lung Disease [GOLD] criteria, pulmonologist follow-up, prior hospitalizations, and LOS).

Result(s): The study comprised 200 patients, average age 69.5 +/- 10.8 years, 61 % males. Of these patients, 89 (45%) were hospitalized for up to 4 days, 111 (55%) for 5 days or more, and 34 (17%) for more than 7 days. Single patients had longer LOS compared with married patients (48% vs. 34%, P = 0.044). Multivariate analysis showed that the number of prior hospital admissions in the last year was a predictor of LOS (P = 0.038, odds ratio [OR] = 0.807, 95% confidence interval [95%CI] = 0.659-0.988), as well as the use of non-invasive respiratory support by bilev-el positive airway pressure (BiPAP) during the hospitalization (P = 0.024, OR = 4.662,95%CI = 1.229-17.681).

Conclusion(s): Fewer previous hospitalizations due to COPD exacerbations and the need for non-invasive respiratory support by BiPAP were found as predictors of longer LOS.

Copyright © 2020 Israel Medical Association. All rights reserved.

PMID

33070485 [<https://www.ncbi.nlm.nih.gov/pubmed/?term=33070485>]

Status

Embase

Institution

(El-Qader, Dovrish) Department of Internal Medicine D, Meir Medical Center, KfarSaba, Israel (Israeli-Shani, Shochet, Wand, Shitrit) Department of Pulmonary Medicine, Meir Medical Center, KfarSaba, Israel

(King, Dahan) Pulmonary Intensive Care Unit, Meir Medical Center, KfarSaba, Israel

(Israeli-Shani, Shochet, King, Dahan, Wand, Shitrit) Sackler Faculty of Medicine, Tel Aviv University, Tel Aviv, Israel

Publisher

Israel Medical Association

Year of Publication

2020

Link to the Ovid Full Text or citation:

[Click here for full text options](https://ovidsp.ovid.com/ovidweb.cgi?T=JS&CSC=Y&NEWS=N&PAGE=fulltext&D=emed21&AN=2008446990)

Link to the External Link Resolver:

[LibKey NHS](https://libkey.io/libraries/2789/openurl?genre=article&sid=OVID:emed21&genre=article&id=pmid:33070485&id=doi:&issn=1565-1088&volume=22&issue=10&spage=552&pages=552-556&date=2020&title=Israel+Medical+Association+Journal&atitle=The+profile+of+copd+patients+with+prolonged+hospitalizations%3A+A+retrospective+analysis+of+200+hospital+admissions&aulast=El-Qader)

54.

Patient outcomes following GPs' educations about COPD: a cluster randomized controlled trial.

Sandelowsky H., Krakau I., Modin S., Stallberg B., Johansson S.-E., Nager A.

Embase

npj Primary Care Respiratory Medicine. 30(1) (no pagination), 2020. Article Number: 44. Date of Publication: 01 Dec 2020.

[Article]

AN: 2006970779

This study aimed to compare patient outcomes following case method learning and traditional lectures as methods for continuing medical education (CME) about chronic obstructive pulmonary disease (COPD) for general practitioners (GPs) in Sweden. In a pragmatic cluster randomized controlled trial, COPD patients (n = 425; case method group n = 209, traditional lectures group n = 216) from 24 primary health care centers replied to questionnaires prior to and 18 months after a 2 x 2-h CME was given to GPs (n = 255). We measured changes in the scores of the Clinical COPD Questionnaire (CCQ), symptoms, needs for disease information, exacerbations, smoking, and use of pulmonary rehabilitation. The changes over time were similar for both CME methods. Patients who had used pulmonary rehabilitation increased from 13.2 to 17.8% (P = 0.04), and prevalence of smoking decreased from 28.9 to 25.1% (P = 0.003). In conclusion, neither of the used CME methods was superior than the other regarding patient outcomes. CME's primary value may lay in improving GPs' adherence to guidelines, which should lead to long-term positive changes in patient health.

Copyright © 2020, The Author(s).

PMID

33060615 [<https://www.ncbi.nlm.nih.gov/pubmed/?term=33060615>]

Status

Embase

Author NameID

Sandelowsky, Hanna; ORCID: <https://orcid.org/0000-0002-1721-6540> Stallberg, Bjorn; ORCID: <https://orcid.org/0000-0001-8497-7326>

Institution

(Sandelowsky, Krakau) Karolinska Institutet, Department of Medicine, Division of Clinical Epidemiology, Stockholm SE-171 76, Sweden (Sandelowsky) Academic Primary Health Care Centre, Stockholm County Council, Box 45436, Stockholm SE-104 31, Sweden

(Sandelowsky, Modin, Nager) Karolinska Institutet, NVS, Section for Family Medicine and Primary Care, Alfred Nobels Alle 23, Huddinge, Stockholm SE-141 83, Sweden

(Stallberg) Uppsala University, Department of Public Health and Caring Sciences, Family Medicine and Preventive Medicine, Box 564, Uppsala SE-751 22, Sweden

(Johansson) Lund University, Center for Primary Health Care Research, Department of Clinical Sciences, Malmo SE-205 02, Sweden

Publisher

Nature Research

Clinical Trial Number

<https://clinicaltrials.gov/show/NCT02213809>

Year of Publication

2020

Link to the Ovid Full Text or citation:

[Click here for full text options](https://ovidsp.ovid.com/ovidweb.cgi?T=JS&CSC=Y&NEWS=N&PAGE=fulltext&D=emed21&AN=2006970779)

Link to the External Link Resolver:

[LibKey NHS](https://libkey.io/libraries/2789/openurl?genre=article&sid=OVID:emed21&genre=article&id=pmid:33060615&id=doi:10.1038%2Fs41533-020-00204-w&issn=2055-1010&volume=30&issue=1&spage=44&pages=&date=2020&title=npj+Primary+Care+Respiratory+Medicine&atitle=Patient+outcomes+following+GPs'+educations+about+COPD%3A+a+cluster+randomized+controlled+trial&aulast=Sandelowsky)

55.

Impact of a specialized ambulatory clinic on refractory breathlessness in subjects with advanced copd.

Elbehairy A.F., McIsaac H., Hill E., Norman P.A., Day A.G., Neder J.A., O'donnell D.E., Harle I.A.

Embase

Respiratory Care. 65(4) (pp 444-454), 2020. Date of Publication: 01 Apr 2020.

[Article]

AN: 2005048762

BACKGROUND: Severe exertional dyspnea is a commonly reported symptom in patients with COPD, especially in the advanced stages. Our objective was to assess the preliminary impact of comprehensive, individualized management provided by a specialized tertiary center clinic on exertional dyspnea and patient-centered outcomes in patients with advanced COPD.

METHOD(S): This retrospective analysis included 45 subjects with COPD who were evaluated in a newly established dyspnea clinic over 3 years. Those with severe exertional dyspnea (Medical Research Council dyspnea score of >4/5), despite optimal disease-targeted therapy were eligible for referral. We used the revised Edmonton Symptom Assessment System (ESAS-r) to assess symptoms. Responders were defined as those whose change from baseline to 2-months met the minimum clinically important difference of <-1 in ESAS-r score for shortness of breath.

RESULT(S): Subjects (mean +/- SD age 70 +/- 7 years) had an average FEV1 of 36 +/- 18% predicted and a Medical Research Council dyspnea score of 4.7 +/- 0.4. Responses to the intervention were variable and mean change in the ESAS-r score for shortness of breath in the total group was-0.32 +/- 3.39, P = .53. Forty-seven percent of the subjects were identified as responders, and 42, 40, 40, and 33% met the minimum clinically important difference for improvement in ESAS-r scores for tiredness, anxiety, well-being, and depression, respectively. Responders had fewer emergency department annual visits in the 2 years after their first clinic visit compared with nonresponders (mean +/- SD, 1.38 +/- 1.63 vs 4.45 +/- 5.52, P = .034).

CONCLUSION(S): Although the impact of our specialized advanced dyspnea clinic was variable, as evaluated by the ESAS-r, it provided measurable additional clinically important benefit to almost half of the subjects with advanced COPD and severe refractory dyspnea.

Copyright © 2020 Daedalus Enterprises.

PMID

31719189 [<https://www.ncbi.nlm.nih.gov/pubmed/?term=31719189>]

Status

Embase

Institution

(Elbehairy, McIsaac, Hill, Neder, O'donnell, Harle) Palliative Care and Respirology Divisions, Department of Medicine, Queen's University and Kingston Health Sciences Centre, Kingston, ON, Canada (Elbehairy) Department of Chest Diseases, Faculty of Medicine, Alexandria University, Alexandria, Egypt

(Norman, Day) Kingston General Health Research Institute, Kingston Health Sciences Centre, Kingston, ON, Canada

Publisher

American Association for Respiratory Care

Year of Publication

2020

Link to the Ovid Full Text or citation:

[Click here for full text options](https://ovidsp.ovid.com/ovidweb.cgi?T=JS&CSC=Y&NEWS=N&PAGE=fulltext&D=emed21&AN=2005048762)

Link to the External Link Resolver:

[LibKey NHS](https://libkey.io/libraries/2789/openurl?genre=article&sid=OVID:emed21&genre=article&id=pmid:31719189&id=doi:10.4187%2Frespcare.06950&issn=0020-1324&volume=65&issue=4&spage=444&pages=444-454&date=2020&title=Respiratory+Care&atitle=Impact+of+a+specialized+ambulatory+clinic+on+refractory+breathlessness+in+subjects+with+advanced+copd&aulast=Elbehairy)

56.

Chronic pulmonary diseases and COVID-19.

Cakir Edis E.

Embase

Turkish Thoracic Journal. 21(5) (pp 345-349), 2020. Date of Publication: September 2020.

[Article]

AN: 2005170312

Over the past few months, coronavirus disease 2019 (COVID-19) has assumed the character of a pandemic, leading to significant global mortality mostly because of COVID-19-related pneumonia. Pneumonia is likely to progress more severely in patients with underlying chronic lung disease. The purpose of this review is to discuss the management strategies in patients with chronic lung disease such as chronic obstructive pulmonary disease, asthma, pleural diseases, and obstructive sleep apnea during the COVID-19 pandemic, with current literatures and international guidelines.

Copyright © 2020 by Turkish Thoracic Society.

Status

Embase

Institution

(Cakir Edis) Department of Pulmonary Medicine, Trakya University School of Medicine, Edirne, Turkey

Publisher

AVES

Year of Publication

2020

Link to the Ovid Full Text or citation:

[Click here for full text options](https://ovidsp.ovid.com/ovidweb.cgi?T=JS&CSC=Y&NEWS=N&PAGE=fulltext&D=emed21&AN=2005170312)

Link to the External Link Resolver:

[LibKey NHS](https://libkey.io/libraries/2789/openurl?genre=article&sid=OVID:emed21&genre=article&id=pmid:&id=doi:10.5152%2FTurkThoracJ.2020.20091&issn=2149-2530&volume=21&issue=5&spage=345&pages=345-349&date=2020&title=Turkish+Thoracic+Journal&atitle=Chronic+pulmonary+diseases+and+COVID-19&aulast=Cakir+Edis)

57.

Does Liuzijue Qigong affect anxiety in patients with chronic obstructive pulmonary disease, even during the COVID-19 outbreak? a randomized, controlled trial.

Zhang Y.-X., Quan Y., Chen M.-H., Zhang D., Zhang Y., Zhu Z.-G.

Embase

Traditional Medicine Research. 5(4) (pp 216-228), 2020. Date of Publication: 02 Jul 2020.

[Article]

AN: 2007113089

Background: Anxiety is a common comorbidity associated with chronic obstructive pulmonary disease (COPD), but no well-recognized method can provide effective relief. Liuzijue Qigong (LQG) is a traditional Chinese fitness method, based on breath pronunciation. This study aimed to examine the efficacy of LQG to relieve anxiety in COPD patients and to explore the factors that influence anxiety, including whether LQG is effective during the coronavirus disease 2019 (COVID-19) outbreak.

Method(s): We conducted an open-label, randomized, controlled, clinical trial. A total of 60 patients with stable COPD were randomly assigned to two groups. Both groups were given routine medical treatment, and the patients in the pulmonary rehabilitation (PR) group were given an extra intervention in the form of LQG, performed for 30 minutes each day for 12 weeks. Data collection was performed at baseline and 12 weeks (during the COVID-19 epidemic). The primary outcomes were the self-rating anxiety scale (SAS) scores, and the secondary outcomes were relevant information during the epidemic and analyses of the related factors that influenced SAS scores during the COVID-19 outbreak.

Result(s): Compared with baseline, patients in both groups demonstrated varying degrees of improvements in their SAS scores (all P < 0.01). An analysis of covariance, adjusted for baseline scores, indicated that the SAS scores improved more dramatically in the PR group than in the control group (F = 9.539, P = 0.004). During the outbreak, the SAS scores for sleep disorder were higher than all other factors, reaching 1.38 +/- 0.67, and the scores for "I can breathe in and out easily" for the PR group were lower than the scores for the control group (Z = -2.108, P = 0.035). Significant differences were identified between the two groups for the categories "How much has the outbreak affected your life", "Do you practice LQG during the epidemic" and "Do you practice other exercises during the epidemic" (all P < 0.05). Compared with current reports, LQG had a relatively high adherence rate (80.95%). A multiple linear regression analysis revealed multiple predictors for SAS scores during the outbreak: group (b = -3.907, t = -3.824, P < 0.001), COPD assessment test score (b = 0.309, t = 2.876, P = 0.006), SAS score at baseline (b = 0.189, t = 3.074, P = 0.004), and living in a village (b = 4.886, t = 2.085, P = 0.043).

Conclusion(s): LQG could effectively reduce the risks of anxiety among COPD patients, even during the COVID-19 outbreak. For those COPD patients with high COPD assessment test and high baseline SAS scores or who live in villages, we should reinforce the management and intervention of psychological factors during the epidemic.

Copyright © 2020 TMR Publishing Group. All rights reserved.

Status

Embase

Institution

(Zhang, Quan, Chen, Zhu) First Teaching Hospital of Tianjin University of Traditional Chinese Medicine, No.88 Changlin Road, Xiqing District, Tianjin 300381, China (Zhang) Tianjin University of Traditional Chinese Medicine, Tianjin 301617, China

(Zhang) Australian National University, Canberra 2601, Australia

Publisher

TMR Publishing Group

Year of Publication

2020

Link to the Ovid Full Text or citation:

[Click here for full text options](https://ovidsp.ovid.com/ovidweb.cgi?T=JS&CSC=Y&NEWS=N&PAGE=fulltext&D=emed21&AN=2007113089)

Link to the External Link Resolver:

[LibKey NHS](https://libkey.io/libraries/2789/openurl?genre=article&sid=OVID:emed21&genre=article&id=pmid:&id=doi:10.12032%2FTMR20200528183&issn=2413-3973&volume=5&issue=4&spage=216&pages=216-228&date=2020&title=Traditional+Medicine+Research&atitle=Does+Liuzijue+Qigong+affect+anxiety+in+patients+with+chronic+obstructive+pulmonary+disease%2C+even+during+the+COVID-19+outbreak%3F+a+randomized%2C+controlled+trial&aulast=Zhang)

58.

An audit of the management of chronic obstructive pulmonary disease (COPD) patients in an outpatient setting: Looking beyond the respiratory illness.

Mintoff M., Montaldo B.C., Azzopardi J.

Embase

Malta Medical Journal. 32(1) (pp 27-35), 2020. Date of Publication: 2020.

[Article]

AN: 2004837623

BACKGROUND COPD is a major public health concern due to its associated morbidity and mortality, most of which is respiratory-related. However, a number of associated conditions exist, which independently contribute to morbidity and mortality, and therefore must be recognised and treated. The aim was to study the quality of management of outpatients with COPD, analyse whether associated comorbidities were being identified and treated, and if not, establish more effective ways of recognising missed opportunities. METHODS This retrospective study examined 37 out-patients with COPD seen by one respiratory firm in a Maltese tertiary centre. Out-patients were randomly selected between 2013 and 2015. The inclusion criterion was a post-bronchodilator FEV1/FVC ratio of <0.7 measured during their most recent spirometry.Outcomemeasures included anaccurate diagnosis ofCOPD; documentation of smoking history and smoking cessation; appropriate COPD treatment including inhaler technique and assessment of non-adherence; appropriateprescriptionandusageofoxygen; referral topulmonary rehabilitation; vaccination status; and consideration of comorbidities. RESULTS 90%weremale, mean age 68.5 years, and had all beencorrectly diagnosedwith COPD, while 22% had a related comorbidity. The majority (81%) were ex-smokers. Virtually all were on inhaled bronchodilators, with 60% also on an inhaled corticosteroid. The uptake of Influenza and Pneumococcal vaccination was at 62% and 54% respectively. Only 24% of patients were given physical activity advice or referral to a pulmonary rehabilitation programme. CONCLUSION The investigators analysed whether practices from one firm are in-keeping with the recommended international guidelines. A number of practices were adequate, while others needed improvement. In order to narrow this discrepancy, the investigatorssuggest creating atemplate forCOPDpatients to be used at future visits which includes the factors investigated.

Copyright © 2020, University of Malta. All rights reserved.

Status

Embase

Institution

(Mintoff, Montaldo, Azzopardi) Department of Respiratory, Medicine Mater Dei Hospital, Msida, Malta

Publisher

University of Malta (E-mail: natalie.galea@um.edu.mt)

Year of Publication

2020

Link to the Ovid Full Text or citation:

[Click here for full text options](https://ovidsp.ovid.com/ovidweb.cgi?T=JS&CSC=Y&NEWS=N&PAGE=fulltext&D=emed21&AN=2004837623)

Link to the External Link Resolver:

[LibKey NHS](https://libkey.io/libraries/2789/openurl?genre=article&sid=OVID:emed21&genre=article&id=pmid:&id=doi:&issn=1813-3339&volume=32&issue=1&spage=27&pages=27-35&date=2020&title=Malta+Medical+Journal&atitle=An+audit+of+the+management+of+chronic+obstructive+pulmonary+disease+(COPD)+patients+in+an+outpatient+setting%3A+Looking+beyond+the+respiratory+illness&aulast=Mintoff)

59.

Effectiveness of structured teaching programme on pulmonary rehabilitation among patients with chronic obstructive pulmonary disease.

Eswari S., Pushpakala K.J.

Embase

Medico-Legal Update. 20(3) (pp 423-428), 2020. Date of Publication: July-September 2020.

[Article]

AN: 632432648

A quasi experimental research design was adopted for the study. The study was conducted in tertiary hospitals of kanchipuram district. The sample of the study well COPD (who are fulfilled the inclusion criteria between the age group of 25yrs-55yrs) the purposive sampling technique 56 samples are experimental group and 56 are considered control group. the data collection instrument was the structured interview to assess the knowledge on pulmonary rehabilitation the results revealed that in the post test of the experimental group, the overall mean 39.46 with SD: 0.738 in control group the overall mean 17.52 with SD 3.722 the impaired T-test value was t=37.808 experimental group the T=3.810 control group its depicts the + there is significant difference between the experimental and control group so that the computer based education is effective the past test of dyspnea scale in experimental group over all mean 0.58 with SD: 0.499 in control group over all mean 1.63 with SD:1.396. the impaired T test value was t=14.402 experimental group. The T=1.264 control group based education programme effective. The past test of cat scale in experimental group mean 9.21 SD=2.213, control group cat scale mean 25.88 SD=5.596 increased control group effective.

Copyright © 2020, World Informations Syndicate. All rights reserved.

Status

Embase

Institution

(Eswari, Pushpakala) Department of Medical Surgical Nursing, Chettinad Hospital and Research Institution, Chettinad Academy of Research and Education, Kanchipuram District, Kelambakkam, Tamil Nadu, India

Publisher

World Informations Syndicate

Year of Publication

2020

Link to the Ovid Full Text or citation:

[Click here for full text options](https://ovidsp.ovid.com/ovidweb.cgi?T=JS&CSC=Y&NEWS=N&PAGE=fulltext&D=emed21&AN=632432648)

Link to the External Link Resolver:

[LibKey NHS](https://libkey.io/libraries/2789/openurl?genre=article&sid=OVID:emed21&genre=article&id=pmid:&id=doi:&issn=0971-720X&volume=20&issue=3&spage=423&pages=423-428&date=2020&title=Medico-Legal+Update&atitle=Effectiveness+of+structured+teaching+programme+on+pulmonary+rehabilitation+among+patients+with+chronic+obstructive+pulmonary+disease&aulast=Eswari)

60.

Implementation of a real-world based ICF set for the rehabilitation of respiratory diseases: A pilot study.

Vitacca M., Giardini A., Corica G., Ceriana P., Carone M., Balbi B., Fracchia C., Maniscalco M., Fanfulla F., Sarno N., Raccanelli R., Traversoni S., Spanevello A.

Embase

Minerva Medica. 11(31) (pp 239-244), 2020. Date of Publication: June 2020.

[Article]

AN: 2006737221

Background: International Classification Functioning (ICF) Core Sets represent a holistic approach to functioning within rehabilitation field. Information-reporting efficacy of a rehabilitation-based Respiratory ICF set applied on a large scale throughout the ICS Maugeri network was tested.

Method(s): A prospective multi-center study (May-November 2018) was conducted for all respiratory inpatients consecutively admitted for rehabilitation. Doctors, physiotherapists, psychologists, nurses used an electronic Respiratory ICF set (33 items among the ICF body functions, activity and participations components) at admission and at discharge to assess the disability changes. The ICF report qualifiers, from 0 (no impairment) to 4 (maximum impairment), guided clinical, diagnostic and rehabilitation prescriptions.

Result(s): 1886 patients (69.6+/-10.8 years; M=1045) were admitted (589 chronic obstructive pulmonary disease, 494 chronic respiratory failure [CRF], 21 prolonged mechanical ventilation [PMV], 496 with other respiratory diseases), of whom 15 died, and 117 were transferred to acute care. The mean length of stay was 23.1+/-11.8 days (range 1-122). The mean time to fill in the ICF set was 23.16+/-0.70 min. The rate of filled charts improved from 16% in May to 100% in November. The baseline distribution of the more severe qualifiers (>2) progressively increased from the whole sample to the PMV subgroup. After rehabilitation, in the whole sample and in the CRF and PMV subgroups, the severity qualifiers significantly decreased (P<0.0001), showing a positive effect of the intervention on patients' disability.

Conclusion(s): Routine use of a Respiratory ICF set for chronic respiratory diseases helps to prepare a personalized rehabilitation program discriminating disability level in different respiratory diseases and assessing disability outcomes pre-post rehabilitation.

Copyright © 2019 Edizioni Minerva Medica.

PMID

31638363 [<https://www.ncbi.nlm.nih.gov/pubmed/?term=31638363>]

Status

Embase

Institution

(Vitacca, Giardini, Corica, Ceriana, Carone, Balbi, Fracchia, Maniscalco, Fanfulla, Sarno, Raccanelli, Traversoni, Spanevello) Department of Respiratory Rehabilitation, Istituti Clinici Scientifici Maugeri IRCCS, Pavia, Italy

Publisher

Edizioni Minerva Medica (E-mail: subscriptions.dept@minervamedica.it)

Year of Publication

2020

Link to the Ovid Full Text or citation:

[Click here for full text options](https://ovidsp.ovid.com/ovidweb.cgi?T=JS&CSC=Y&NEWS=N&PAGE=fulltext&D=emed21&AN=2006737221)

Link to the External Link Resolver:

[LibKey NHS](https://libkey.io/libraries/2789/openurl?genre=article&sid=OVID:emed21&genre=article&id=pmid:31638363&id=doi:10.23736%2FS0026-4806.19.06261-X&issn=0026-4806&volume=11&issue=31&spage=239&pages=239-244&date=2020&title=Minerva+Medica&atitle=Implementation+of+a+real-world+based+ICF+set+for+the+rehabilitation+of+respiratory+diseases%3A+A+pilot+study&aulast=Vitacca)

61.

Impact of a smartphone application (KAIA COPD app) in combination with Activity Monitoring as a maintenance prOgram following PUlmonary Rehabilitation in COPD: The protocol for the AMOPUR Study, an international, multicenter, parallel group, randomized, controlled study.

Spielmanns M., Boeselt T., Huber S., Kaur Bollinger P., Ulm B., Peckaka-Egli A.-M., Jarosch I., Schneeberger T., Schoendorf S., Gloeckl R., Koczulla A.R.

Embase

Trials. 21(1) (no pagination), 2020. Article Number: 636. Date of Publication: 11 Jul 2020.

[Article]

AN: 632284459

Background: Increasing physical activity (PA) is considered to be an important factor for the efficient management of chronic obstructive pulmonary disease (COPD). Successful methods required to achieve improvements in PA following pulmonary rehabilitation (PR), however, are rarely reported. Therefore, we will conduct this trial to evaluate the effectiveness of using a COPD management program delivered to the patient via the KAIA COPD app, a mobile medical application, after the completion of PR.

Method(s): This is the protocol for a randomized, controlled, open-label, multicentered trial that will be carried out at inpatient PR hospital centers in Germany and Switzerland. The interventions will involve the use of the KAIA COPD app program (Arm 1) or an active comparator, i.e., usual care (Arm 2). Patients completing an in-hospital PR program and consenting to participate in the study will be screened with the inclusion and exclusion criteria and enrolled in the study. After fulfilling the screening requirements, the patients will be randomized into one of the two arms with parallel group assignment in a 1:1 ratio. The training program will be delivered to the participants grouped in Arm 1 via the KAIA COPD app and to participants grouped in Arm 2 via the regular recommendations or standard of care by the PI. In total, 104 participants will be included in the trial. The treatment period will last for 24 weeks. Electronic versions of questionnaires will be used to collect patient-reported assessments remotely. The primary outcome measure is the change in physical activity of the intervention group in comparison to the control group, measured over 1 week as the mean steps per day with a Polar A 370 activity tracker, from baseline (end of PR) to the 6-month follow-up. The secondary outcome measures are functional exercise capacity, health status, sleep quality, exacerbation rate, and depression and anxiety symptoms assessed at several intervals.

Discussion(s): This study seeks to prove the effects of the KAIA COPD mobile application in COPD patients after PR. The app offers educational, exercise training plus activity monitoring and motivational programs that can be easily implemented in the patient's home setting, enabling patients to maintain the effects that are typically elicited in the short term after pulmonary rehabilitation for the long term. Trial registration: German Clinical Trials Register (DRKS00017275). Protocol version 2.0 dated 3 June 2019.

Copyright © 2020 The Author(s).

PMID

32653025 [<https://www.ncbi.nlm.nih.gov/pubmed/?term=32653025>]

Status

Embase

Institution

(Spielmanns, Peckaka-Egli, Schoendorf) Pneumologie, Zurcher RehaZentren Klinik Wald, Faltigbergstrasse 7, Wald 8636, Switzerland (Spielmanns) Faculty of Health, Department of Pulmonary Medicine, University of Witten/Herdecke, Witten 58448, Germany

(Boeselt, Koczulla) Department of Pulmonary Rehabilitation, Philipps-University of Marburg, German Center for Lung Research (DZL), Marburg, Germany

(Huber, Kaur Bollinger) Fa. Kaia Health GmbH, Siegfriedstr.8, Munich 80797, Germany

(Ulm) Unabhangiges Statistische Beratung Berhard Ulm, Kochelseestr 11, Munich D-81371, Germany

(Jarosch, Schneeberger, Gloeckl, Koczulla) Institute for Pulmonary Rehabilitation Research, Schoen Klinik Berchtesgadener Land, Schoenau am Koenigssee, Germany

(Gloeckl) Department of Prevention, Rehabilitation and Sports Medicine, Technical University of Munich (TUM), Munich, Germany

Publisher

BioMed Central (E-mail: info@biomedcentral.com)

Clinical Trial Number

00017275/DRKS

Year of Publication

2020

Link to the Ovid Full Text or citation:

[Click here for full text options](https://ovidsp.ovid.com/ovidweb.cgi?T=JS&CSC=Y&NEWS=N&PAGE=fulltext&D=emed21&AN=632284459)

Link to the External Link Resolver:

[LibKey NHS](https://libkey.io/libraries/2789/openurl?genre=article&sid=OVID:emed21&genre=article&id=pmid:32653025&id=doi:10.1186%2Fs13063-020-04538-1&issn=1745-6215&volume=21&issue=1&spage=636&pages=&date=2020&title=Trials&atitle=Impact+of+a+smartphone+application+(KAIA+COPD+app)+in+combination+with+Activity+Monitoring+as+a+maintenance+prOgram+following+PUlmonary+Rehabilitation+in+COPD%3A+The+protocol+for+the+AMOPUR+Study%2C+an+international%2C+multicenter%2C+parallel+group%2C+randomized%2C+controlled+study&aulast=Spielmanns)

62.

Goals of COPD treatment: Focus on symptoms and exacerbations.

Vogelmeier C.F., Roman-Rodriguez M., Singh D., Han M.K., Rodriguez-Roisin R., Ferguson G.T.

Embase

Respiratory Medicine. 166 (no pagination), 2020. Article Number: 105938. Date of Publication: May 2020.

[Review]

AN: 2005472007

Chronic obstructive pulmonary disease (COPD) is currently a leading cause of death worldwide, and its burden is expected to rise in the coming years. Common COPD symptoms include dyspnea, cough and/or sputum production. Some patients may experience acute worsening of symptoms (known as an exacerbation), and therefore require additional therapy. Exacerbations are mainly triggered by respiratory infections and environmental factors. Healthcare professionals face many challenges in COPD management, including the heterogeneity of the disease and under-reporting of symptoms. The authors review these challenges and provide recommendations for the best methods to assess COPD. The goals of COPD treatment include recognising the impact that both symptoms and exacerbations have on patients' lives when considering optimal patient-focused management. The review discusses the need for COPD management strategies to include both pharmacologic and non-pharmacologic approaches and provides recommendations for monitoring treatment outcomes and adjusting management strategies accordingly. Novel treatment strategies including precision medicine and point-of-care testing are also discussed.

Copyright © 2020 Elsevier Ltd

PMID

32250871 [<https://www.ncbi.nlm.nih.gov/pubmed/?term=32250871>]

Status

Embase

Author NameID

Vogelmeier, Claus F.; ORCID: <https://orcid.org/0000-0002-9798-2527>

Institution

(Vogelmeier) Department of Medicine, Pulmonary and Critical Care Medicine, University Medical Center Giessen and Marburg, Philipps University of Marburg, Member of the German Center for Lung Research (DZL), Marburg, Germany (Roman-Rodriguez) Son Pisa Primary Health Care Centre, Instituto de Investigacion Sanitaria de Las Islas Baleares (IdISBa), Palma de Mallorca, Spain

(Singh) Medicines Evaluation Unit, University of Manchester, Manchester University NHS Foundation Trust, Manchester, United Kingdom

(Han) Division of Pulmonary and Critical Care Medicine, Department of Internal Medicine, University of Michigan Health System, Ann Arbor, MI, United States

(Rodriguez-Roisin) University of Barcelona, Hospital Clinic, IDIBAPS-CIBERES, Barcelona, Spain

(Ferguson) Pulmonary Research Institute of Southeast Michigan, Farmington Hills, MI, United States

Publisher

W.B. Saunders Ltd

Year of Publication

2020

Link to the Ovid Full Text or citation:

[Click here for full text options](https://ovidsp.ovid.com/ovidweb.cgi?T=JS&CSC=Y&NEWS=N&PAGE=fulltext&D=emed21&AN=2005472007)

Link to the External Link Resolver:

[LibKey NHS](https://libkey.io/libraries/2789/openurl?genre=article&sid=OVID:emed21&genre=article&id=pmid:32250871&id=doi:10.1016%2Fj.rmed.2020.105938&issn=0954-6111&volume=166&issue=&spage=105938&pages=&date=2020&title=Respiratory+Medicine&atitle=Goals+of+COPD+treatment%3A+Focus+on+symptoms+and+exacerbations&aulast=Vogelmeier)

63.

Noninvasive positive pressure ventilation in stable patients with COPD.

Wiles S.P., Aboussouan L.S., Mireles-Cabodevila E.

Embase

Current Opinion in Pulmonary Medicine. 26(2) (pp 175-185), 2020. Date of Publication: 01 Mar 2020.

[Review]

AN: 631059344

Purpose of reviewLong-term noninvasive positive pressure ventilation (NIV) used to be a controversial form of therapy for patients with stable hypercapnic chronic obstructive pulmonary disease (SH-COPD). New evidence described in this review defines the optimal settings, timing and target population for NIV utilization in SH-COPD necessary to maximize its benefit.Recent findingsNIV, when titrated appropriately, leads to improved clinical outcomes. High inspiratory positive airway pressures aimed at decreasing CO2 levels can ensure NIV success in SH-COPD. NIV initiated when patients remain hypercapnic whereas in a clinical stable state following an acute exacerbation can prolong the time to a readmission. Technological advances in NIV algorithms and remote monitoring have the potential to improve use and titration. NIV and portable NIV improve exercise tolerance and may accentuate the benefits derived from pulmonary rehabilitation alone.SummaryUse of high-intensity NIV in SH-COPD is beneficial yet appropriate patient selection and implementation is paramount.

Copyright © 2020 Lippincott Williams and Wilkins. All rights reserved.

PMID

31895118 [<https://www.ncbi.nlm.nih.gov/pubmed/?term=31895118>]

Status

Embase

Institution

(Wiles, Aboussouan, Mireles-Cabodevila) Respiratory Institute, Cleveland Clinic, 9500 Euclid Avenue, Cleveland, OH 44195, United States

Publisher

Lippincott Williams and Wilkins (E-mail: kathiest.clai@apta.org)

Year of Publication

2020

Link to the Ovid Full Text or citation:

[Click here for full text options](https://ovidsp.ovid.com/ovidweb.cgi?T=JS&CSC=Y&NEWS=N&PAGE=fulltext&D=emed21&AN=631059344)

Link to the External Link Resolver:

[LibKey NHS](https://libkey.io/libraries/2789/openurl?genre=article&sid=OVID:emed21&genre=article&id=pmid:31895118&id=doi:10.1097%2FMCP.0000000000000657&issn=1070-5287&volume=26&issue=2&spage=175&pages=175-185&date=2020&title=Current+Opinion+in+Pulmonary+Medicine&atitle=Noninvasive+positive+pressure+ventilation+in+stable+patients+with+COPD&aulast=Wiles)

64.

Care for Patients with Advanced Chronic Obstructive Pulmonary Disease.

Harrison A., Robinson J., McDonald C.F., Kho Y.H.

Embase

Respirology. Conference: TSANZSRS 2020 Australia and New Zealand Society of Respiratory Science and the Thoracic Society of Australia and New Zealand Annual Scientific Meeting, ANZSRS/TSANZ. Melbourne, VIC Australia. 25 (pp 72), 2020. Date of Publication: June 2020.

[Conference Abstract]

AN: 633831671

Introduction/Aim. Patients with advanced chronic obstructive pulmonary disease (COPD) experience a high symptom burden with significant comorbidities. Their management often necessitates a multi-disciplinary approach, with both pharmacological and non-pharmacological approaches to treatment of their respiratory disease. Our study evaluated the respiratory management and care needs of patients with advanced COPD attending the Oxygen Service at a tertiary centre in Melbourne. Methods. We retrospectively analysed the medical records of patients who were prescribed domiciliary oxygen therapy for COPD between January 2012 and December 2018 at Austin Health. Data collected included patient demographics, comorbidities, physiologic parameters, and respiratory management. A random sample was selected for this preliminary analysis. Results. Of the 77 patients included (mean age 75.3 years, 42 female, FEV1 50.9% predicted), four had an overlap diagnosis of interstitial lung disease. Common comorbidities were cardiac disease (53%) pulmonary hypertension (51%), and sleep disordered breathing and hypoventilation syndrome (40%). The mean Charlson comorbidity index score was 6.5 (SD 2.3). Forty-seven (61%) patients were referred to pulmonary rehabilitation. Hospital Admission Risk Program and allied health services including dietician, occupational therapy, speech pathology and social work were involved in the management of 35 (45%) patients. Seventy-six patients were prescribed ambulatory oxygen therapy, one nocturnal oxygen therapy and 45 long-term oxygen therapy. In terms of inhaler therapies, 65 (84%) patients were prescribed "triple therapy" including inhaled corticosteroid, long-acting beta-2-agonist and long-acting muscarinic antagonist. Two patients were not using any regular inhaled therapies. Only 19% of patients were referred to palliative care services. Conclusion. Patients with advanced COPD have high levels of comorbidity and require multidisciplinary team management. The majority of patients received appropriate disease-specific pharmacological therapies, although referrals to pulmonary rehabilitation, a strongly evidence based intervention, was suboptimal and less than a fifth of patients had been referred to palliative care services.

Status

CONFERENCE ABSTRACT

Institution

(Harrison, Robinson, McDonald, Kho) Department of Respiratory Medicine, Austin Health (McDonald, Kho) Institute for Breathing and Sleep

(McDonald, Kho) Department of Medicine, University of Melbourne, VIC

Publisher

Blackwell Publishing

Year of Publication

2020

Link to the Ovid Full Text or citation:

[Click here for full text options](https://ovidsp.ovid.com/ovidweb.cgi?T=JS&CSC=Y&NEWS=N&PAGE=fulltext&D=emed21&AN=633831671)

Link to the External Link Resolver:

[LibKey NHS](https://libkey.io/libraries/2789/openurl?genre=article&sid=OVID:emed21&genre=article&id=pmid:&id=doi:10.1111%2Fresp.13777&issn=1323-7799&volume=25&issue=1&spage=72&pages=72&date=2020&title=Respirology&atitle=Care+for+Patients+with+Advanced+Chronic+Obstructive+Pulmonary+Disease&aulast=Harrison)

65.

The relationship between kinesiophobia, dyspnea level, functional exercise capacity and quality of life in patients with chronic obstructive pulmonary disease.

Kahraman B.O., Ozsoy I., Tanriverdi A., Acar S., Alpaydin A.O., Sevinc C., Savci S.

Embase

European Respiratory Journal. Conference: European Respiratory Society International Congress, ERS 2020. Virtual. 56(Supplement 64) (no pagination), 2020. Date of Publication: September 2020.

[Conference Abstract]

AN: 633803564

Aims and Objectives: Kinesiophobia is defined as fear of movement and activity resulting from a feeling of vulnerability to painful injury or reinjury. Although there are studies concerning kinesiophobia and its relationship with clinical variables in various chronic diseases, there are limited data about kinesiophobia in COPD. The aim of this study was to evaluate the relationship between kinesiophobia, dyspnea level, functional exercise capacity and quality of life in patients with COPD.

Method(s): Thirty-seven patients with moderate/severe COPD were participated in this study. Dyspnea level was determined using the modified Medical Research Council Dyspnea Scale. Kinesiophobia was measured using Tampa Scale of Kinesiophobia (TSK). Six-Minute Walk Test was used to evaluate functional exercise capacity. Nottingham Health Profile scale Health-related quality of life energy level, pain, emotional reactions, sleep, social isolation and physical abilities sub-domains were used to assess quality of life.

Result(s): TSK score was significantly associated with dyspnea level, functional exercise capacity, pain and physical abilities (p<0.05). Although there was no correlation between TSK scores and energy level, emotional reactions, sleep and social isolation sub-domains of quality of life (p>0.05)

Conclusion(s): Dyspnea level, functional exercise capacity and quality of life subdomains are all positively associated with higher degree of kinesiophobia in patients with COPD. It is important that kinesiophobia should be evaluate in routine assessments because of its effects on main outcomes of pulmonary rehabilitation programs.

Status

CONFERENCE ABSTRACT

Institution

(Kahraman) School of Physical Therapy and Rehabilitation, Dokuz Eylul University, Izmir, Turkey (Ozsoy) Department of Physiotherapy and Rehabilitation, Faculty of Health Sciences, Selcuk University, Konya, Turkey

(Tanriverdi) Graduate School of Health Sciences, Dokuz Eylul University, Izmir, Turkey

(Acar, Alpaydin, Sevinc, Savci) Department of Chest Disease, Faculty of Medicine, Dokuz Eylul University, Izmir, Turkey

Publisher

European Respiratory Society

Year of Publication

2020

Link to the Ovid Full Text or citation:

[Click here for full text options](https://ovidsp.ovid.com/ovidweb.cgi?T=JS&CSC=Y&NEWS=N&PAGE=fulltext&D=emed21&AN=633803564)

Link to the External Link Resolver:

[LibKey NHS](https://libkey.io/libraries/2789/openurl?genre=article&sid=OVID:emed21&genre=article&id=pmid:&id=doi:10.1183%2F13993003.congress-2020.2463&issn=1399-3003&volume=56&issue=Supplement+64&spage=&pages=&date=2020&title=European+Respiratory+Journal&atitle=The+relationship+between+kinesiophobia%2C+dyspnea+level%2C+functional+exercise+capacity+and+quality+of+life+in+patients+with+chronic+obstructive+pulmonary+disease&aulast=Kahraman)

66.

Pulmonary rehabilitation and its impact on the quality of life of patients with copd.

Rodrigues A.R., Lopes A.C., Ferreira V.B., Faleiros P.A.D.M.

Embase

Sleep Science. Conference: Congresso Brasileiro do Sono 2019. Foz do Iguacu Brazil. 13(Supplement 1) (pp 109-110), 2020. Date of Publication: 2020.

[Conference Abstract]

AN: 631496557

Introduction: Chronic obstructive pulmonary disease (COPD) is characterized by chronic airfow limitation, which is not completely reversible after bronchodilator use. Airfow limitation is often progressive and associated with the abnormal infammatory response of the lungs to toxic gases or particles. This disease is one of the major causes of morbidity and mortality in the world, defned by such nomenclature due to the diffculty of differentiating pulmonary emphysema from chronic bronchitis (PRESTO and DAMAZIO, 2005; ZANCHET et al, 2005 FERNANDES, 2009). Pulmonary rehabilitation (PR) is recommended for the treatment of COPD, as it generates improvement in exercise capacity, respiratory muscle strength and quality of life. Studies have found increased walking distance on the six-minute walk test, maximal upper limb load, maximal inspiratory pressure, and quality of life (ZANCHET et al, 2005). In relation to sleep, COPD causes intermittent hypoxemia with pulmonary alterations in patients, which can alter sleep and generate cough, dyspnea, decrease oxygen saturation and consequently worsen the quality of sleep and life of these individuals (MARTIN et al, 2018).

Aim(s): The aim of this report is to show the importance of pulmonary rehabilitation in sleep and life quality in COPD patientsMethods: The experience took place through a meeting with the presence of 21 people, aged 50 to 80 years, with COPD, participants of a Pulmonary Rehabilitation program in a city in the interior of Sao Paulo. In the frst moment, the COPD theme was approached with the participants and their families, in order to generate knowledge for them about their illnesses and enable them to recognize their potential and diffculties. After this presentation, a space was provided for participants, family members and/or companions present to refect and discuss the theme and their experiences with the disease, in addition to exposing their doubts and sharing their progress after pulmonary rehabilitation.

Result(s): Participants were pleased to participate, showing that opportunities such as these are critical for them to share their anxieties, answer questions and report on their progress. Moreover, they showed that participating in experiences such as these enables improvements in quality of life, even with the presence of the disease, and correct attitudes towards treatment.

Conclusion(s): Pulmonary rehabilitation has been shown to be critical for patients with lung disease and COPD treatment.

Status

CONFERENCE ABSTRACT

Institution

(Rodrigues, Lopes, Ferreira, Faleiros) Centro Universitario Municipal de Franca, Franca, Sao Paulo, Brazil

Publisher

Brazilian Association of Sleep and Latin American Federation of Sleep Societies

Year of Publication

2020

Link to the Ovid Full Text or citation:

[Click here for full text options](https://ovidsp.ovid.com/ovidweb.cgi?T=JS&CSC=Y&NEWS=N&PAGE=fulltext&D=emed21&AN=631496557)

Link to the External Link Resolver:

[LibKey NHS](https://libkey.io/libraries/2789/openurl?genre=article&sid=OVID:emed21&genre=article&id=pmid:&id=doi:&issn=1984-0063&volume=13&issue=Supplement+1&spage=109&pages=109-110&date=2020&title=Sleep+Science&atitle=Pulmonary+rehabilitation+and+its+impact+on+the+quality+of+life+of+patients+with+copd&aulast=Rodrigues)

67.

Effects of oral melatonin associated with pulmonary rehabilitation on sleep quality and daytime sleepiness in COPD.

Souza C.V., Viana S.M.N.R., Bruin V.M.S., Bruin P.F.C., Farias T.A.R., Rego O.M.V., Viana G.D.N.R.

Embase

Sleep Science. Conference: Congresso Brasileiro do Sono 2019. Foz do Iguacu Brazil. 13(Supplement 1) (pp 101), 2020. Date of Publication: 2020.

[Conference Abstract]

AN: 631496344

Introduction: Sleep problems are common in COPD. Melatonin (MLT), the main product of the pineal gland, is central to circadian rhythm regulation and its administration reportedly improves sleep in COPD. MLT has also been shown to improve lung oxidative stress in these patients. Pulmonary rehabilitation (PR) can increase exercise tolerance and quality of life and reduce symptoms but it is still unclear if it can improve sleep in patients with COPD. We hypothesized that concomitant administration of MLT could potentialize beneftial effects of PR.

Aim(s): To investigate the effects of oral MLT (3 mg/day) associated with pulmonary rehabilitation for 12 weeks on sleep quality and daytime sleepiness in COPD.

Method(s): This was a double-blind, randomized, placebo-controlled trial. Thirty-nine patients [24 males; mean age (+/-SD) = 66.6+/-10,4 years] regularly attending a COPD outpatient clinic at a University Hospital, who were referred for PR, were randomized to receive 3 mg MLT (n = 18) or placebo (n = 21) for the duration of the PR program (12 weeks). At baseline, sleep quality was assessed subjectively by the Pittsburgh Sleep Quality Index (PSQI) and objectively by actigraphy for 7 consecutive days. Daytime sleepiness was measured by the Epworth Sleepiness Scale (ESS). All patients flled a Two Week Sleep Diary. Measures were repeated at the end of the PR program for comparison.

Result(s): On average, patients who received MLT, but not placebo, showed a signifcant improvement in PSQI global score (respectively, 9.44 +/- 4.47 - 4.39 +/- 1.88; p < 0.05 vs 7.86 +/- 2.97 - 7.62 +/- 2.55 p = 0.6). A reduction in the EES score was observed only for the MLT group (respectively, 9.1 +/- 6.0 - 6.7 +/- 4.5; p = 0.02 vs 10.1 +/- 5.0 -9.5 +/- 5.1; p = 0.2). Data obtained from sleep diary showed an increase in total sleep time (329.1+/-667.0 - 391.9+/-73.0; p<0.05) and sleep effciency (80.64 +/- 9 - 87.44 +/- 6.7; p<0.05) and a reduction in sleep onset latency (-27.40 minutes; p < 0.05) in patients who received MLT, but not placebo. Actigraphy showed a signifcant increase in sleep effciency (68.5 +/- 14.1 - 80.3 +/- 12.9; p<0.05) for the MLT group, despite no changes in sleep latency or duration.

Conclusion(s): Melatonin supplementation 3 mg over a 12-week period can reduce daytime sleepiness and improve subjective sleep quality as well as some aspects of objective sleep quality in patients with COPD undergoing pulmonary rehabilitation.

Status

CONFERENCE ABSTRACT

Institution

(Souza, Viana, Bruin, Bruin, Farias, Rego, Viana) Universidade Federal Do Ceara, Fortaleza, Ceara, Brazil

Publisher

Brazilian Association of Sleep and Latin American Federation of Sleep Societies

Year of Publication

2020

Link to the Ovid Full Text or citation:

[Click here for full text options](https://ovidsp.ovid.com/ovidweb.cgi?T=JS&CSC=Y&NEWS=N&PAGE=fulltext&D=emed21&AN=631496344)

Link to the External Link Resolver:

[LibKey NHS](https://libkey.io/libraries/2789/openurl?genre=article&sid=OVID:emed21&genre=article&id=pmid:&id=doi:&issn=1984-0063&volume=13&issue=Supplement+1&spage=101&pages=101&date=2020&title=Sleep+Science&atitle=Effects+of+oral+melatonin+associated+with+pulmonary+rehabilitation+on+sleep+quality+and+daytime+sleepiness+in+COPD&aulast=Souza)

68.

Use of time in chronic obstructive pulmonary disease: Longitudinal associations with symptoms and quality of life using a compositional analysis approach.

Lewthwaite H., Olds T., Williams M.T., Effing T.W., Dumuid D.

Embase

PLoS ONE. 14(3) (no pagination), 2019. Article Number: e0214058. Date of Publication: March 2019.

[Article]

AN: 2001730751

Background and objectives This study explored whether, for people with chronic obstructive pulmonary disease (COPD), changes to the 24-hour composition of physical activity (PA), sedentary behaviour (SB) and sleep were associated with changes in symptoms and health-related quality of life (HRQoL); and how time re-allocations between these behaviours were associated with changes in outcomes. Methods This study pools data on people with COPD drawn from two previous studies: a randomised controlled trial of cognitive behavioural therapy and pulmonary rehabilitation and a usual care cohort. Participants recalled behaviours and completed symptom and HRQoL assessments at baseline (T0) and four months (T1). Linear mixed-effects models (pooled control/ intervention samples) predicted changes in outcomes from T0 to T1 with a change to the 24-hour behaviour composition; compositional isotemporal substitution predicted change in outcomes when re-allocating time between behaviours. Results Valid data were obtained for 95 participants (forced expiratory volume in one second %predicted = 49.6+/-15.3) at T0 and T1. A change in the 24-hour behaviour composition was associated with a change in anxiety (p<0.01) and mastery (p<0.01), but not breathlessness, depression or fatigue. When modelling time re-allocation with compositional isotemporal substitution, more time re-allocated to higher intensity PA or sleep was associated with favourable changes in outcomes; re-allocating time to SB or light PA was associated with unfavourable changes in outcomes. The direction of association, however, could not be determined. Conclusion To improve the overall health and wellbeing of people with COPD, intervention approaches that optimise the composition of PA, SB and sleep may be beneficial.

Copyright © 2019 Lewthwaite et al. This is an open access article distributed under the terms of the Creative Commons Attribution License, which permits unrestricted use, distribution, and reproduction in any medium, provided the original author and source are credited.

PMID

30897134 [<https://www.ncbi.nlm.nih.gov/pubmed/?term=30897134>]

Status

Embase

Institution

(Lewthwaite, Olds, Williams, Dumuid) Alliance for Research in Exercise, Nutrition and Activity, School of Health Sciences, University of South Australia, Adelaide, SA, Australia (Effing) College of Medicine and Public Health, Flinders University, Bedford Park, SA, Australia

(Effing) Department of Respiratory Medicine, Southern Adelaide Local Health Network, Bedford Park, SA, Australia

Publisher

Public Library of Science

Clinical Trial Number

12611000292976/ANZCTR

Year of Publication

2019

Link to the Ovid Full Text or citation:

[Click here for full text options](https://ovidsp.ovid.com/ovidweb.cgi?T=JS&CSC=Y&NEWS=N&PAGE=fulltext&D=emed20&AN=2001730751)

Link to the External Link Resolver:

[LibKey NHS](https://libkey.io/libraries/2789/openurl?genre=article&sid=OVID:emed20&genre=article&id=pmid:30897134&id=doi:10.1371%2Fjournal.pone.0214058&issn=1932-6203&volume=14&issue=3&spage=e0214058&pages=&date=2019&title=PLoS+ONE&atitle=Use+of+time+in+chronic+obstructive+pulmonary+disease%3A+Longitudinal+associations+with+symptoms+and+quality+of+life+using+a+compositional+analysis+approach&aulast=Lewthwaite)

69.

Pulmonary Rehabilitation does not Improve Objective Measures of Sleep Quality in People with Chronic Obstructive Pulmonary Disease.

Cox N.S., Pepin V., Burge A.T., Hill C.J., Lee A.L., Bondarenko J., Moore R., Nicolson C., Lahham A., Parwanta Z., McDonald C.F., Holland A.E.

Embase

COPD: Journal of Chronic Obstructive Pulmonary Disease. 16(1) (pp 25-29), 2019. Date of Publication: 2019.

[Article]

AN: 2014011807

Abnormal sleep duration is associated with poor health. Upwards of 50% of people with chronic obstructive pulmonary disease (COPD) report poor sleep quality. The effect of pulmonary rehabilitation on self-reported sleep quality is variable. The aim of this study was to assess the effect of pulmonary rehabilitation on objectively measured sleep quality (via actigraphy) in people with COPD. Sleep quality was assessed objectively using the SenseWear Armband (SWA, BodyMedia, Pittsburgh, PA), worn for >=4 days before and immediately after completing an 8-week pulmonary rehabilitation program. Sleep characteristics were derived from accelerometer positional data and registration of sleep state by the SWA, determined from energy expenditure. Forty-eight participants (n = 21 male) with COPD (mean (SD), age 70 (10) years, mean FEV1 55 (20) % predicted, mean 45 (24) pack year smoking history) contributed pre and post pulmonary rehabilitation sleep data to this analysis. No significant differences were seen in any sleep parameters after pulmonary rehabilitation (p = 0.07-0.70). There were no associations between sleep parameters and measures of quality of life or function (all p > 0.30). Sleep quality, measured objectively using actigraphy, did not improve after an 8-week pulmonary rehabilitation program in individuals with COPD. Whether on-going participation in regular exercise training beyond the duration of pulmonary rehabilitation may influence sleep quality, or whether improving sleep quality could enhance rehabilitation outcomes, is yet to be determined.

Copyright © 2019 Taylor & Francis Group, LLC.

PMID

30884984 [<https://www.ncbi.nlm.nih.gov/pubmed/?term=30884984>]

Status

Embase

Institution

(Cox, Lahham) Discipline of Physiotherapy, La Trobe University and Institute for Breathing and Sleep, Melbourne, Australia (Pepin, Parwanta) Department of Health, Kinesiology, and Applied Physiology, Axe Maladies Chroniques, Centre de Recherche de l'Hopital du Sacre-Coeur de Montreal, Concordia University, Montreal, Canada

(Burge, Holland) Discipline of Physiotherapy, Department of Physiotherapy, Alfred Health, Institute for Breathing and Sleep, La Trobe University, Melbourne, Australia

(Hill) Department of Physiotherapy, Austin Health, and Institute for Breathing and Sleep, Melbourne, Australia

(Lee) Department of Physiotherapy, Alfred Health, and Institute for Breathing and Sleep, Monash University, Frankston, Australia

(Bondarenko) Department of Physiotherapy, Alfred Health, Melbourne, Australia

(Moore) Institute for Breathing and Sleep, Melbourne, Australia

(Nicolson) Discipline of Physiotherapy, La Trobe University and Department of Physiotherapy, Alfred Health, Melbourne, Australia

(McDonald) Department of Respiratory Medicine, Austin Health, Institute for Breathing and Sleep, and School of Medicine, University of Melbourne, Melbourne, Australia

Publisher

Taylor and Francis Ltd.

Year of Publication

2019

Link to the Ovid Full Text or citation:

[Click here for full text options](https://ovidsp.ovid.com/ovidweb.cgi?T=JS&CSC=Y&NEWS=N&PAGE=fulltext&D=emed20&AN=2014011807)

Link to the External Link Resolver:

[LibKey NHS](https://libkey.io/libraries/2789/openurl?genre=article&sid=OVID:emed20&genre=article&id=pmid:30884984&id=doi:10.1080%2F15412555.2019.1567701&issn=1541-2555&volume=16&issue=1&spage=25&pages=25-29&date=2019&title=COPD%3A+Journal+of+Chronic+Obstructive+Pulmonary+Disease&atitle=Pulmonary+Rehabilitation+does+not+Improve+Objective+Measures+of+Sleep+Quality+in+People+with+Chronic+Obstructive+Pulmonary+Disease&aulast=Cox)

70.

The yellow brick road.

Tine M., Turrin M., Lokar-Oliani K., Cosio M.G., Saetta M.

Embase

Minerva Pneumologica. 58(4) (pp 118-121), 2019. Date of Publication: 2019.

[Article]

AN: 2005009928

Yellow nail syndrome is an uncommon condition characterized by dystrophic yellow nails, lymphedema and respiratory tract involvement. This syndrome typically shows up in middle-aged patients. Although several etiologies have been described, to date, the exact underlying mechanism remains unclear. The most supported pathogenetic hypothesis argues that it results from an abnormal lymphatic drainage. Hereby, we describe the associations of yellow nail syndrome typical features in a 73-year-old man with an acute onset of symptoms. He was admitted to our hospital with acute respiratory failure requiring non-invasive ventilation. Despite our advice, he rapidly relapsed. Taking care of his multiple comorbidities - cardiomyopathy, sleep apnea syndrome and severe obstructive deficit - his symptoms finally improved. Adherence to nocturnal non-invasive ventilation, bronchodilator therapy and pulmonary rehabilitation provided him stability. Acute and critical presentation could mystify the diagnosis of rare syndromes such as the yellow nail syndrome. Its precocious recognition reinforced our approach justifying a detailed screening of the patient's conditions.

Copyright © 2019 EDIZIONI MINERVA MEDICA

Status

Embase

Institution

(Tine, Turrin, Lokar-Oliani, Cosio, Saetta) Department of Cardiac, Thoracic, Vascular Sciences and Public Health, University of Padua, Padua, Italy (Cosio) Respiratory Division, Meakins-Christie Laboratories, McGill University, Montreal, QC, Canada

Publisher

Edizioni Minerva Medica (E-mail: subscriptions.dept@minervamedica.it)

Year of Publication

2019

Link to the Ovid Full Text or citation:

[Click here for full text options](https://ovidsp.ovid.com/ovidweb.cgi?T=JS&CSC=Y&NEWS=N&PAGE=fulltext&D=emed20&AN=2005009928)

Link to the External Link Resolver:

[LibKey NHS](https://libkey.io/libraries/2789/openurl?genre=article&sid=OVID:emed20&genre=article&id=pmid:&id=doi:10.23736%2FS0026-4954.19.01862-5&issn=0026-4954&volume=58&issue=4&spage=118&pages=118-121&date=2019&title=Minerva+Pneumologica&atitle=The+yellow+brick+road&aulast=Tine)

71.

Fentanyl nasal spray in a patient with end-stage COPD and severe chronic breathlessness.

Janssen D.J.A., van den Beuken-Van Everdingen M.H.J., Verberkt C.A., Creemers J.P.H.M., Wouters E.F.M.

Embase

Breathe. 15(3) (pp e122-e125), 2019. Date of Publication: 01 Sep 2019.

[Article]

AN: 2003372510

Status

Embase

Institution

(Janssen, Wouters) CIRO, Horn, Netherlands (Janssen, van den Beuken-Van Everdingen) Centre of Expertise for Palliative Care, Maastricht University Medical Centre (MUMC+), Maastricht, Netherlands

(Verberkt) Dept of Health Services Research, Maastricht University, Maastricht, Netherlands

(Creemers) Dept of Respiratory Medicine, Catharina Hospital, Eindhoven, Netherlands

(Wouters) Dept of Respiratory Medicine, Maastricht University Medical Centre (MUMC+), Maastricht, Netherlands

Publisher

European Respiratory Society (E-mail: info@ersnet.org)

Year of Publication

2019

Link to the Ovid Full Text or citation:

[Click here for full text options](https://ovidsp.ovid.com/ovidweb.cgi?T=JS&CSC=Y&NEWS=N&PAGE=fulltext&D=emed20&AN=2003372510)

Link to the External Link Resolver:

[LibKey NHS](https://libkey.io/libraries/2789/openurl?genre=article&sid=OVID:emed20&genre=article&id=pmid:&id=doi:10.1183%2F20734735.0183-2019&issn=1810-6838&volume=15&issue=3&spage=e122&pages=e122-e125&date=2019&title=Breathe&atitle=Fentanyl+nasal+spray+in+a+patient+with+end-stage+COPD+and+severe+chronic+breathlessness&aulast=Janssen)

72.

Triangulated perspectives on outcomes of pulmonary rehabilitation in patients with COPD: a qualitative study to inform a core outcome set.

Souto-Miranda S., Marques A.

Embase

Clinical rehabilitation. 33(4) (pp 805-814), 2019. Date of Publication: 01 Apr 2019.

[Article]

AN: 626895853

INTRODUCTION:: Pulmonary rehabilitation implies a comprehensive assessment. Although several outcomes are commonly measured, those are selected mainly by health professionals and researchers, with the voice of patients and informal caregivers being minimally captured. Qualitative studies are fundamental to enhance our knowledge on perspectives of different stakeholders involved in pulmonary rehabilitation.

OBJECTIVE(S):: This study aimed to explore the views of different stakeholders on outcomes of pulmonary rehabilitation, contributing to one of the stages of a core outcome set for pulmonary rehabilitation in patients with chronic obstructive pulmonary disease (COPD).

METHOD(S):: Semi-structured interviews were conducted with 12 patients with COPD, 11 informal carers and 10 health professionals. Data were analysed with content analysis, followed by thematic analysis to gain deeper understanding of the different perspectives.

RESULT(S):: A total of 44 outcomes were identified, being the most reported 'improving functional performance' (67%) and 'reducing and taking control over dyspnoea' (64%). Five relevant themes across stakeholders were generated: having a healthy mind in a healthy body; I can('t) do it; feeling fulfilled; knowing more, doing better and avoiding doctors and expenses. Although perspectives were mostly consensual, some outcomes were only valued by health professionals (e.g. pulmonary function) or by patients and informal carers (e.g. quality of sleep).

CONCLUSION(S):: Views of the different stakeholders on outcomes of pulmonary rehabilitation were similar although, some specificities existed. Comprehensive assessments are needed to reflect what is valued by the different stakeholders in pulmonary rehabilitation. This study contributed to a future core outcome set in this field.

PMID

30592227 [<https://www.ncbi.nlm.nih.gov/pubmed/?term=30592227>]

Author NameID

Marques, Alda; ORCID: <https://orcid.org/0000-0003-4980-6200>

Institution

(Souto-Miranda, Marques) Lab 3R - Respiratory Research and Rehabilitation Laboratory, School of Health Sciences (ESSUA) and Institute of Biomedicine (iBiMED), University of Aveiro, Aveiro, Portugal

Publisher

NLM (Medline)

Year of Publication

2019

Link to the Ovid Full Text or citation:

[Click here for full text options](https://ovidsp.ovid.com/ovidweb.cgi?T=JS&CSC=Y&NEWS=N&PAGE=fulltext&D=emed20&AN=626895853)

Link to the External Link Resolver:

[LibKey NHS](https://libkey.io/libraries/2789/openurl?genre=article&sid=OVID:emed20&genre=article&id=pmid:30592227&id=doi:10.1177%2F0269215518821405&issn=1477-0873&volume=33&issue=4&spage=805&pages=805-814&date=2019&title=Clinical+rehabilitation&atitle=Triangulated+perspectives+on+outcomes+of+pulmonary+rehabilitation+in+patients+with+COPD%3A+a+qualitative+study+to+inform+a+core+outcome+set&aulast=Souto-Miranda)

73.

Physiological and clinical characteristics of patients with COPD admitted to an inpatient pulmonary rehabilitation program: A real-life study.

Maestri R., Bruschi C., Fracchia C., Pinna G.D., Fanfulla F., Ambrosino N.

Embase

Pulmonology. 25(2) (pp 71-78), 2019. Date of Publication: March - April 2019.

[Article]

AN: 2001042303

Background and objective: Patient selection criteria and experimental interventions of randomized controlled trials may not reflect how things work in practice. The aim of this study was to describe the characteristics of chronic obstructive pulmonary disease (COPD) patients undergoing an inpatient pulmonary rehabilitation program (PRP) and the correlates of success.

Method(s): Retrospective database review of 975 consecutive patients transferred from acute care hospitals after an acute exacerbation (group A: 14.6%) or admitted from home (group B: 75.4%), from 2010 to 2017. Patients were also divided according to the associated registered main diagnosis: COPD (group 1: 30.6%); COPD and respiratory failure (group 2: 51.7%); COPD and obstructive sleep apnea (group 3: 17.6%). Baseline correlates of post-PRP changes in six minute walking test (6MWT) were also evaluated.

Result(s): Global Initiative for Chronic Obstructive Lung Disease stages 3 and 4 were the most commonly represented in group 2 (p = 0.0001). Comorbidity Index of all patients was 3.9 +/- 1.8. The overall in-hospital mortality rate was 1.3% (5.6% vs 0.6%, in groups A and B, respectively; p = 0.0001). Hypertension, cardiac diseases and obesity were observed in 65.2, 52.2 and 29.6% of patients, respectively. Post-PRP 6MWT increased in all groups. Age, male gender, airway obstruction and baseline 6MWT were correlated with a post-PRP 30 meter increase in 6MWT.

Conclusion(s): Confirming data of literature, this real-life study shows the characteristics of COPD patients undergoing an inpatient PRP with significant improvement in exercise capacity, independent of whether in stable state or after a recent exacerbation or of the associated main diagnosis.

Copyright © 2018 Sociedade Portuguesa de Pneumologia

PMID

30143469 [<https://www.ncbi.nlm.nih.gov/pubmed/?term=30143469>]

Status

Embase

Institution

(Maestri, Bruschi, Fracchia, Pinna, Fanfulla, Ambrosino) Istituti Clinici Scientifici Maugeri, IRCCS Istituto di Montescano, Pavia, Italy

Publisher

Elsevier Espana S.L.U

Year of Publication

2019

Link to the Ovid Full Text or citation:

[Click here for full text options](https://ovidsp.ovid.com/ovidweb.cgi?T=JS&CSC=Y&NEWS=N&PAGE=fulltext&D=emed20&AN=2001042303)

Link to the External Link Resolver:

[LibKey NHS](https://libkey.io/libraries/2789/openurl?genre=article&sid=OVID:emed20&genre=article&id=pmid:30143469&id=doi:10.1016%2Fj.pulmoe.2018.07.001&issn=2531-0429&volume=25&issue=2&spage=71&pages=71-78&date=2019&title=Pulmonology&atitle=Physiological+and+clinical+characteristics+of+patients+with+COPD+admitted+to+an+inpatient+pulmonary+rehabilitation+program%3A+A+real-life+study&aulast=Maestri)

74.

Clinical course and management of idiopathic pulmonary fibrosis.

Quinn C., Wisse A., Manns S.T.

Embase

Multidisciplinary Respiratory Medicine. 14(1) (no pagination), 2019. Article Number: 35. Date of Publication: 02 Dec 2019.

[Review]

AN: 629996138

Idiopathic pulmonary fibrosis (IPF) is a progressive, fatal interstitial lung disease (ILD) with an unpredictable clinical course. Although IPF is rare, healthcare professionals should consider IPF as a potential cause of unexplained chronic dyspnea and/or cough in middle-aged/elderly patients and refer patients to a pulmonologist for evaluation. Making a diagnosis of IPF requires specialist expertise. Multidisciplinary discussion, involving at minimum a pulmonologist and a radiologist with expertise in the differential diagnosis of ILDs, is required to ensure the most accurate diagnosis. Prompt diagnosis of IPF is important to enable patients to receive appropriate care from an early stage. Optimal management of IPF involves the use of antifibrotic drugs, as well as the provision of supportive care to alleviate symptoms and preserve patients' quality of life. Antifibrotic drugs have been shown to slow lung function decline seen in patients with IPF. Patients' symptoms and functional capacity can be improved through participation in pulmonary rehabilitation programs and the use of supplemental oxygen. Patient education is essential to help patients understand and manage their disease. The identification and management of comorbidities, such as obstructive sleep apnea, pulmonary hypertension, and emphysema, is also an important element of the overall care of patients with IPF. Patients with IPF should be evaluated for lung transplantation at an early stage to maximize their chances of meeting eligibility criteria. In this review, we describe the clinical course and impact of IPF and best practice in its management, highlighting the importance of taking a patient-centered approach.

Copyright © 2019 The Author(s).

Status

Embase

Author NameID

Quinn, Caitlin; ORCID: <https://orcid.org/0000-0001-7390-7728>

Institution

(Quinn) Emory Critical Care Center, 1364 Clifton Road, NE, Atlanta, GA 30322, United States (Wisse) Medical University of South Carolina (MUSC), 25 Courtenay Drive, MSC 114, Charleston, SC 29425, United States

(Manns) Pulmonary and Critical Care Medicine, Wake Med Brier Creek Healthplex, Brier Creek, 8001 TW Alexander Drive, Suite 218, Raleigh, NC 27617, United States

Publisher

BioMed Central Ltd. (E-mail: info@biomedcentral.com)

Year of Publication

2019

Link to the Ovid Full Text or citation:

[Click here for full text options](https://ovidsp.ovid.com/ovidweb.cgi?T=JS&CSC=Y&NEWS=N&PAGE=fulltext&D=emed20&AN=629996138)

Link to the External Link Resolver:

[LibKey NHS](https://libkey.io/libraries/2789/openurl?genre=article&sid=OVID:emed20&genre=article&id=pmid:&id=doi:10.1186%2Fs40248-019-0197-0&issn=1828-695X&volume=14&issue=1&spage=35&pages=&date=2019&title=Multidisciplinary+Respiratory+Medicine&atitle=Clinical+course+and+management+of+idiopathic+pulmonary+fibrosis&aulast=Quinn)

75.

Real-world comprehensive disease management of patients with idiopathic pulmonary fibrosis.

Mason W., McLaughlin S., Dedopoulos S., Mahoney E., Meadows T., Stauffer J.L., Lancaster L.H.

Embase

Current Respiratory Medicine Reviews. 15(1) (pp 4-15), 2019. Date of Publication: 2019.

[Review]

AN: 2002835185

Idiopathic Pulmonary Fibrosis (IPF) is a debilitating, progressive, and fatal fibrotic lung disease with a poor prognosis. Antifibrotic therapy slows but does not halt disease progression. Patient education and management needs change during disease progression. Management is complicated by comorbidities, adverse events associated with antifibrotic therapy, and difficulties with long-term oxygen therapy and pulmonary rehabilitation. Treating IPF requires coordination between physicians and nurses in community and interstitial lung disease center settings. This review provides guidance for the healthcare professional who manages the essential aspects of care in IPF from diagnosis, through disease progression, and to the end of life.

Copyright © 2019 Bentham Science Publishers.

Status

Embase

Institution

(Mason, Lancaster) Department of Medicine, Vanderbilt University, Nashville, TN, United States (McLaughlin) University of California, San Francisco, California, CA, United States

(Dedopoulos) Northwell Health, New Hyde Park, New York, NY, United States

(Mahoney) Loyola University Medical Center, Maywood, IL, United States

(Meadows) University of Alabama at Birmingham, Birmingham, AL, United States

(Stauffer) Genentech, Inc., South San Francisco, CL, United States

Publisher

Bentham Science Publishers (P.O. Box 294, Bussum 1400 AG, Netherlands)

Year of Publication

2019

Link to the Ovid Full Text or citation:

[Click here for full text options](https://ovidsp.ovid.com/ovidweb.cgi?T=JS&CSC=Y&NEWS=N&PAGE=fulltext&D=emed20&AN=2002835185)

Link to the External Link Resolver:

[LibKey NHS](https://libkey.io/libraries/2789/openurl?genre=article&sid=OVID:emed20&genre=article&id=pmid:&id=doi:10.2174%2F1573398X15666190212155051&issn=1573-398X&volume=15&issue=1&spage=4&pages=4-15&date=2019&title=Current+Respiratory+Medicine+Reviews&atitle=Real-world+comprehensive+disease+management+of+patients+with+idiopathic+pulmonary+fibrosis&aulast=Mason)

76.

Comorbidities in idiopathic pulmonary fibrosis: An underestimated issue.

Caminati A., Lonati C., Cassandro R., Elia D., Pelosi G., Torre O., Zompatori M., Uslenghi E., Harari S.

Embase

European Respiratory Review. 28(153) (no pagination), 2019. Article Number: 190044. Date of Publication: 30 Sep 2019.

[Review]

AN: 2002736090

Idiopathic pulmonary fibrosis (IPF) is a progressive and fibrosing lung disease with a poor prognosis. Between 60% and 70% of IPF patients die of IPF; the remaining causes of death may be due to comorbidities occurring in this ageing population. Interest in the role played by comorbidities in IPF has increased in the past few years. The optimal clinical management of IPF is multifaceted and not only involves antifibrotic treatment, but also vaccinations, oxygen supplementation, evaluation of nutritional status as well as psychological support and patient education. Symptom management, pulmonary rehabilitation, palliative care and treatment of comorbidities represent further areas of clinical intervention. This review analyses the major comorbidities observed in IPF, focusing on those that have the greatest impact on mortality and quality of life (QoL). The identification and treatment of comorbidities may help to improve patients' health-related QoL (i.e. sleep apnoea and depression), while some comorbidities (i.e. lung cancer, cardiovascular diseases and pulmonary hypertension) influence survival. It has been outlined that gathering comorbidities data improves the prediction of survival beyond the clinical and physiological parameters of IPF.

Copyright © ERS 2019.

PMID

31578211 [<https://www.ncbi.nlm.nih.gov/pubmed/?term=31578211>]

Status

Embase

Institution

(Caminati, Cassandro, Elia, Torre, Harari) UO di Pneumologia e Terapia Semi-Intensiva Respiratoria - Servizio di Fisiopatologia Respiratoria ed Emodinamica Polmonare, Ospedale San Giuseppe - MultiMedica IRCCS, Milan, Italy (Lonati, Harari) UO di Medicina Generale, Ospedale San Giuseppe - MultiMedica IRCCS, Milan, Italy

(Pelosi) Dipartimento di Oncologia ed Onco-ematologia, Universita degli Studi di Milano, Milan, Italy

(Pelosi) Servizio Interaziendale di Anatomia Patologica, Polo Scientifico e Tecnologico, MultiMedica IRCCS, Milan, Italy

(Zompatori, Uslenghi) Dipartimento di Diagnostica per Immagini e UO di Radiologia, MultiMedica IRCCS, Milan, Italy

(Zompatori) Dipartimento Universitario DIMES, Universita di Bologna, Bologna, Italy

Publisher

European Respiratory Society (E-mail: info@ersnet.org)

Year of Publication

2019

Link to the Ovid Full Text or citation:

[Click here for full text options](https://ovidsp.ovid.com/ovidweb.cgi?T=JS&CSC=Y&NEWS=N&PAGE=fulltext&D=emed20&AN=2002736090)

Link to the External Link Resolver:

[LibKey NHS](https://libkey.io/libraries/2789/openurl?genre=article&sid=OVID:emed20&genre=article&id=pmid:31578211&id=doi:10.1183%2F16000617.0044-2019&issn=0905-9180&volume=28&issue=153&spage=190044&pages=&date=2019&title=European+Respiratory+Review&atitle=Comorbidities+in+idiopathic+pulmonary+fibrosis%3A+An+underestimated+issue&aulast=Caminati)

77.

Treatable traits in acute exacerbations of chronic airway diseases.

McDonald V.M., Osadnik C.R., Gibson P.G.

Embase

Chronic Respiratory Disease. 16 (no pagination), 2019. Date of Publication: 12 Aug 2019.

[Review]

AN: 628942848

Acute exacerbations of chronic airway disease are common occurrences that cause a major burden of illness. Acute exacerbations are associated with impaired health status, increased lung function decline, hospitalization and increased risk of death. Exacerbation avoidance is a major priority. Despite this goal, exacerbations continue to occur and the need for effective models of care that optimize patient outcomes are urgently needed. 'Treatable Traits' is an approach to personalized medicine that has been proposed for the management of airway diseases. The treatable traits approach allows for the recognition of clinically important, identifiable and treatable disease characteristics, followed by targeted and individualized treatment interventions to address each trait. We review the literature relating to treatable traits in airway diseases; in particular, those traits that can predict exacerbations and approaches to management that aim to prevent exacerbations by using a treatable traits model of care. We propose this approach as a potentially useful model of care to both prevent and manage acute exacerbations.

Copyright © The Author(s) 2019.

PMID

31409129 [<https://www.ncbi.nlm.nih.gov/pubmed/?term=31409129>]

Status

Embase

Author NameID

McDonald, Vanessa M; ORCID: <https://orcid.org/0000-0001-9890-3408> Gibson, Peter G; ORCID: <https://orcid.org/0000-0001-5865-489X>

Osadnik, Christian R; ORCID: <https://orcid.org/0000-0001-9040-8007>

Institution

(McDonald, Gibson) Priority Research Centre for Healthy Lungs and Centre of Excellence in Severe Asthma, Faculty of Health and Medicine, University of Newcastle, NSW, Australia (McDonald, Gibson) Department of Respiratory and Sleep Medicine, John Hunter Hospital, Newcastle, Australia

(Osadnik) Department of Physiotherapy, Monash University, Melbourne, Australia

(Osadnik) Monash Lung and Sleep, Monash Health, Melbourne, Australia

Publisher

SAGE Publications Ltd (E-mail: info@sagepub.co.uk)

Year of Publication

2019

Link to the Ovid Full Text or citation:

[Click here for full text options](https://ovidsp.ovid.com/ovidweb.cgi?T=JS&CSC=Y&NEWS=N&PAGE=fulltext&D=emed20&AN=628942848)

Link to the External Link Resolver:

[LibKey NHS](https://libkey.io/libraries/2789/openurl?genre=article&sid=OVID:emed20&genre=article&id=pmid:31409129&id=doi:10.1177%2F1479973119867954&issn=1479-9723&volume=16&issue=&spage=&pages=&date=2019&title=Chronic+Respiratory+Disease&atitle=Treatable+traits+in+acute+exacerbations+of+chronic+airway+diseases&aulast=McDonald)

78.

Management of Chronic Respiratory Failure in Interstitial Lung Diseases: Overview and Clinical Insights.

Faverio P., De Giacomi F., Bonaiti G., Stainer A., Sardella L., Pellegrino G., Sferrazza Papa G.F., Bini F., Bodini B.D., Carone M., Annoni S., Messinesi G., Pesci A.

Embase

International Journal of Medical Sciences. 16(7) (pp 967-980), 2019. Date of Publication: 2019.

[Article]

AN: 2002401640

Interstitial lung diseases (ILDs) may be complicated by chronic respiratory failure (CRF), especially in the advanced stages. Aim of this narrative review is to evaluate the current evidence in management of CRF in ILDs. Many physiological mechanisms underlie CRF in ILDs, including lung restriction, ventilation/perfusion mismatch, impaired diffusion capacity and pulmonary vascular damage. Intermittent exertional hypoxemia is often the initial sign of CRF, evolving, as ILD progresses, into continuous hypoxemia. In the majority of the cases, the development of CRF is secondary to the worsening of the underlying disease; however, associated comorbidities may also play a role. When managing CRF in ILDs, the need for pulmonary rehabilitation, the referral to lung transplant centers and palliative care should be assessed and, if necessary, promptly offered. Long-term oxygen therapy is commonly prescribed in case of resting or exertional hypoxemia with the purpose to decrease dyspnea and improve exercise tolerance. High-Flow Nasal Cannula oxygen therapy may be used as an alternative to conventional oxygen therapy for ILD patients with severe hypoxemia requiring both high flows and high oxygen concentrations. Non-Invasive Ventilation may be used in the chronic setting for palliation of end-stage ILD patients, although the evidence to support this application is very limited.

Copyright © 2019 Ivyspring International Publisher. All rights reserved.

PMID

31341410 [<https://www.ncbi.nlm.nih.gov/pubmed/?term=31341410>]

Status

Embase

Institution

(Faverio, De Giacomi, Bonaiti, Stainer, Sardella, Messinesi, Pesci) School of Medicine and Surgery, University of Milano-Bicocca, Respiratory Unit, San Gerardo Hospital, ASST di Monza, Monza, Italy (Pellegrino, Sferrazza Papa) Casa di Cura del Policlinico, Dipartimento di Scienze Neuroriabilitative, Milan, Italy

(Bini) UOC Pulmonology, Department of Internal Medicine, Ospedale ASST-Rhodense, Garbagnate Milanese, Italy

(Bodini) Pulmonology Unit, Ospedale Maggiore della Carita, University of Piemonte Orientale, Novara, Italy

(Carone) UOC Pulmonology and Pulmonary Rehabilitation, Istituti Clinici Scientifici Maugeri, IRCCS di Cassano Murge (BA), Italy

(Annoni) Physical therapy and Rehabilitation Unit, San Gerardo Hospital, ASST di Monza, Monza, Italy

Publisher

Ivyspring International Publisher (E-mail: info@ivyspring.com)

Year of Publication

2019

Link to the Ovid Full Text or citation:

[Click here for full text options](https://ovidsp.ovid.com/ovidweb.cgi?T=JS&CSC=Y&NEWS=N&PAGE=fulltext&D=emed20&AN=2002401640)

Link to the External Link Resolver:

[LibKey NHS](https://libkey.io/libraries/2789/openurl?genre=article&sid=OVID:emed20&genre=article&id=pmid:31341410&id=doi:10.7150%2Fijms.32752&issn=1449-1907&volume=16&issue=7&spage=967&pages=967-980&date=2019&title=International+Journal+of+Medical+Sciences&atitle=Management+of+Chronic+Respiratory+Failure+in+Interstitial+Lung+Diseases%3A+Overview+and+Clinical+Insights&aulast=Faverio)

79.

The feasibility of domiciliary non-invasive mechanical ventilation due to chronic respiratory failure in very elderly patients.

Ocakli B.

Embase

Turkish Thoracic Journal. 20(2) (pp 130-135), 2019. Date of Publication: April 2019.

[Article]

AN: 2001760256

OBJECTIVES: The aim of this study was to investigate the use of domiciliary non-invasive mechanical ventilation (NIMV) in very elderly patients (age 80 and over). MATERIALS AND METHODS: This retrospective study included a total of 44 patients aged 80 years or older, who were admitted to the Health Sciences University, Sureyyapasa Chest Diseases and Thoracic Surgery Training and Research Hospital, Pulmonary Intensive Care Outpatient Clinic between 2012 and 2018 and applied NIMV for chronic respiratory failure. The patients were divided into two groups: survivors (n=15) and non-survivors (n=29). Data were obtained from the retrospectively formed hospital database. The characteristics of patients, comorbidities, NIMV compliance, pulmonary function tests, and blood gas analyses were compared between the survivors and non-survivors.

RESULT(S): From the retrospective analysis of 44 cases, the non-survivors were found to have a significantly shorter duration of domiciliary NIMV (737 days vs. 890 days, p=0.027) and lower hemoglobin concentration (11.1 g/L vs. 12.9 g/L, p=0.004). The number of comorbid conditions, pulmonary function test, and blood gas analyses results did not differ significantly between the groups. Compliance was moderate in this elderly population, at 4.9+/-1.9 h/day (range: 0.8-9.1 h/day). NIMV was well-tolerated in 36 of the 44 elderly patients (81.8%). Overall mortality was 65.9%.

CONCLUSION(S): Domiciliary NIMV can be of benefit to very elderly patients, and age is not an obstacle. Therefore, this population should not be excluded from this treatment solely on the basis of age.

Copyright © 2019 by Turkish Thoracic Society.

Status

Embase

Institution

(Ocakli) Intensive Care Unit, Health Sciences University, Sureyyapasa Chest Diseases and Thoracic Surgery Training and Research Hospital, Istanbul, Turkey

Publisher

AVES (105/9 Buyukdere Cad, Mecidiyekoy,Sisli, Istanbul 34394, Turkey)

Year of Publication

2019

Link to the Ovid Full Text or citation:

[Click here for full text options](https://ovidsp.ovid.com/ovidweb.cgi?T=JS&CSC=Y&NEWS=N&PAGE=fulltext&D=emed20&AN=2001760256)

Link to the External Link Resolver:

[LibKey NHS](https://libkey.io/libraries/2789/openurl?genre=article&sid=OVID:emed20&genre=article&id=pmid:&id=doi:10.5152%2FTurkThoracJ.2018.18119&issn=2149-2530&volume=20&issue=2&spage=130&pages=130-135&date=2019&title=Turkish+Thoracic+Journal&atitle=The+feasibility+of+domiciliary+non-invasive+mechanical+ventilation+due+to+chronic+respiratory+failure+in+very+elderly+patients&aulast=Ocakli)

80.

High-intensity non-invasive ventilation in stable hypercapnic COPD: Evidence of efficacy and practical advice.

van der Leest S., Duiverman M.L.

Embase

Respirology. 24(4) (pp 318-328), 2019. Date of Publication: April 2019.

[Review]

AN: 625340535

Patients with end-stage chronic obstructive pulmonary disease (COPD) frequently develop chronic hypercapnic respiratory failure (CHRF), with disabling symptoms and poor survival. The use of long-term nocturnal non-invasive ventilation (NIV) to treat CHRF in COPD has long been subject of debate due to conflicting evidence. However, since the introduction of high-intensity NIV (HI-NIV) in COPD, physiological and clinical benefits have been shown. HI-NIV refers to specific ventilator settings used for NIV aimed at achieving normocapnia or the lowest partial arterial carbon dioxide pressure (PaCO2) values as possible. This review will provide an overview of existing evidence of the efficacy of HI-NIV stable COPD patients with CHRF. Secondly, we will discuss hypotheses underlying NIV benefit in stable hypercapnic COPD, providing insight into better patient selection and hopefully more individually titrated HI-NIV. Finally, we will provide practical advice on how to initiate and follow-up patients on HI-NIV, with special emphasis on monitoring that should be available during the initiation and follow-up of HI-NIV, and will discuss more extended monitoring techniques that could improve HI-NIV treatment in the future.

Copyright © 2018 Asian Pacific Society of Respirology

PMID

30500099 [<https://www.ncbi.nlm.nih.gov/pubmed/?term=30500099>]

Status

Embase

Author NameID

Duiverman, Marieke L.; ORCID: <https://orcid.org/0000-0002-8818-9447>

Institution

(van der Leest) Cardiovascular and Respiratory Physiology Group, Technical Medical Centre, University of Twente, Enschede, Netherlands (van der Leest, Duiverman) Department of Pulmonary Diseases/Home Mechanical Ventilation, University of Groningen, University Medical Center Groningen, Groningen, Netherlands

(van der Leest, Duiverman) Groningen Research Institute of Asthma and COPD (GRIAC), University of Groningen, Groningen, Netherlands

Publisher

Blackwell Publishing

Year of Publication

2019

Link to the Ovid Full Text or citation:

[Click here for full text options](https://ovidsp.ovid.com/ovidweb.cgi?T=JS&CSC=Y&NEWS=N&PAGE=fulltext&D=emed20&AN=625340535)

Link to the External Link Resolver:

[LibKey NHS](https://libkey.io/libraries/2789/openurl?genre=article&sid=OVID:emed20&genre=article&id=pmid:30500099&id=doi:10.1111%2Fresp.13450&issn=1323-7799&volume=24&issue=4&spage=318&pages=318-328&date=2019&title=Respirology&atitle=High-intensity+non-invasive+ventilation+in+stable+hypercapnic+COPD%3A+Evidence+of+efficacy+and+practical+advice&aulast=van+der+Leest)

81.

Frailty of copd patients at the pulmonary rehabilitation clinic: An exploratory research in validity of the Kihon Checklist (KCL) in patients with chronic comorbidities for the registry study at the frailty prevention clinic in national center for geriatrics and gerontology, Japan.

Senda K., Satake S., Kondo I., Tokuda H., Matsui Y.

Embase

Aging Medicine and Healthcare. Conference: 5th Asian Conference for Frailty and Sarcopenia, ACFS 2019. Taipei Taiwan (Republic of China). 10(Supplement 1) (pp 38), 2019. Date of Publication: October 2019.

[Conference Abstract]

AN: 632977045

Background: We studied frailty of older COPD patients at the pulmonary rehabilitation clinic as an exploratory research in validity of the Kihon Checklist (KCL) in patients with chronic comorbidities for the registry study at the Frailty Prevention Clinic (FPC): the Locomo-frail Clinic in National Center for Geriatrics and Gerontology (NCGG), Japan. COPD is a prevalent chronic systemic inflammatory disease with multi-comorbidities and one of geriatric syndromes associated with sarcopenia leading to frailty. Older COPD patients carry the constellation of frailty, disabilities, and co-morbidities. KCL is a self-administrated, comprehensive 25 items questionnaire for screening tool for the Preventive Care Service in Japan.

Method(s): Stable outpatients with COPD at the Pulmonary Rehabilitation Clinic, NCGG, underwent a comprehensive geriatric assessment (CGA) and followed from October 2010 to March 2019 by a registered nurse. The cohort consisted of 40 males and 3 females; age: 74.9+/-5.9 (65-87) years, BMI: 21.7+/-3.2 kg/m2, appendicular skeletal muscle index (ASMI): 6.61+/-0.64 kg/m2, Charson Comorbidity Index (CCI): 1.7+/-1.2. Control group was ageand gender-matched outpatients with geriatric syndromes (including sleep apnea syndrome, DM, dementia, etc.) at NCGG, age: 75.1+/-5.8 (66- 87) years, BMI: 22.8+/-3.3 kg/m2, ASMI: 6.54+/-2.00 kg/m2, CCI: 1.7+/-1.4.

Result(s): Initial KCL of COPD group was 5.2/25+/-4.4 (0-18). With frailty evaluation by KCL, 9 were classified as frail (F, KCL >=8), 17 as pre-frail (7 >=P >=4), and 17 as robust (3 >=R). As J-CHS criteria, F/P/R in COPD group were 5/27/11. In control group, KCL was 6.1+/-5.4 (5-18), and F/P/R were 16/10/17 with KCL, and 10/19/14 with J-CHS. KCL was concordant with number of J-CHS frailty criteria and parameters of CGA in patients with COPD and geriatric syndromes. Twelve of fatal 16 COPD cases were P (KCL >4) and 4 of 5 fatal cases in control were F. Nine cases in control group were lost the continuity of care at transitions of care settings.

Conclusion(s): The classification of frailty status by KCL score could be a significant tool to predict the mortality in older outpatients with COPD and geriatric syndromes. KCL might be a useful tool for frailty screening in patients with chronic comorbidities. COPD patients showed same level of frailty as geriatric syndrome outpatients at FPC, NCGG. Fatal case in this study showed higher KCL, which indicated possibility of KCL to evaluate not only physical function but prognosis for COPD and geriatric syndrome patients in integrated care settings by interdisciplinary team. We conduct the registry study with KCL among the older people at the FPC in NCGG.

Status

CONFERENCE ABSTRACT

Institution

(Senda, Satake, Kondo, Tokuda, Matsui) Center for Frailty and Locomotive Syndrome, National Center for Geriatrics and Gerontology, Obu, Japan

Publisher

Full Universe Integrated Marketing Limited

Year of Publication

2019

Link to the Ovid Full Text or citation:

[Click here for full text options](https://ovidsp.ovid.com/ovidweb.cgi?T=JS&CSC=Y&NEWS=N&PAGE=fulltext&D=emed20&AN=632977045)

Link to the External Link Resolver:

[LibKey NHS](https://libkey.io/libraries/2789/openurl?genre=article&sid=OVID:emed20&genre=article&id=pmid:&id=doi:&issn=2663-8851&volume=10&issue=Supplement+1&spage=38&pages=38&date=2019&title=Aging+Medicine+and+Healthcare&atitle=Frailty+of+copd+patients+at+the+pulmonary+rehabilitation+clinic%3A+An+exploratory+research+in+validity+of+the+Kihon+Checklist+(KCL)+in+patients+with+chronic+comorbidities+for+the+registry+study+at+the+frailty+prevention+clinic+in+national+center+for+geriatrics+and+gerontology%2C+Japan&aulast=Senda)

82.

Impact of pulmonary rehabilitation on sleep in COPD patients as measured by actigraphy.

Thapamagar S.B., Ellstrom K.E., Anholm J.D., Dandamudi N.

Embase

American Journal of Respiratory and Critical Care Medicine. Conference: 2019 International Conference of the American Thoracic Society , ATS 2019. Dallas, TX United States. 199(9) (no pagination), 2019. Date of Publication: May 2019.

[Conference Abstract]

AN: 630351075

Introduction: Chronic Obstructive Pulmonary Disease (COPD) patients have poor sleep quality, longer time to sleep onset, frequent arousals and awakenings. On the other hand, poor sleep quality in COPD is associated with increased COPD exacerbations, poor quality of life and mortality. Pulmonary rehabilitation (PR) improves functional status and quality of life in COPD but its effect on sleep is unclear. PR does not improve subjective sleep quality measured by Pittsburg Sleep Quality Index (PSQI) but objective actigraphy data are not available. We hypothesized that actigraphy would demonstrate objective improvement in sleep following PR.

Method(s): This is a retrospective analysis of prospectively collected data in COPD patients undergoing PR at the VA Loma Linda Healthcare system from 2013 to 2017. Actigraphy watch recordings before and after 8-weeks of PR measured changes in sleep variables including total time in bed (TBT), total sleep time (TST), sleep onset latency (SOL), sleep efficiency (SE), wakefulness after sleep onset (WASO) and total awakenings. Changes in PSQI was a secondary outcome.

Result(s): Of 77 participants, 69 were included in the final analysis due to missing data. Most participants were male (97%), non-obese (BMI 29+/-6.9 kg/m2; mean+/-SD) with an average age of 69+/-8 years and 71% had severe COPD (GOLD stage 3 or 4). Paired non-parametric comparisons (Wilcoxon signed rank test) did not show any improvement in sleep parameters by actigraphy following 8-weeks PR despite significant improvements in 6-min-walk distance (6MWD - mean improvement of 54.0m, 95% CI 34m to 74m, p<0.0001) and St. Georges' Respiratory Questionnaire scores (SGRQ - mean improvement of 7.7 points, 95% CI 5.2 to 10.2, p<0.0001). Detailed actigraphy outcomes are shown in the table. Stratified analysis of all sleep variables by severity of COPD, BMI, mood, mental status, 6-min walk distance and SGRQ did not show significant improvement after PR. PR did not improve subjective sleep quality by PSQI (Pre-PR median PSQI 8, vs. post-PR PSQI 7, p=0.08).

Conclusion(s): PR did not improve either objective sleep parameters by actigraphy or subjective sleep quality by PSQI. Our PSQI data are in agreement with the published literature and are further supported by objective actigraphy data. The lack of improvement in sleep parameters following PR highlights the more complex interactions among COPD, sleep and exercise. Further studies with more advanced methods to assess sleep may demonstrate more subtle effects of PR on sleep.

Status

CONFERENCE ABSTRACT

Institution

(Thapamagar) Pulmonary and Critical Care Medicine, Loma Linda University, Redlands, CA, United States (Ellstrom, Anholm) J L Pettis VA Medical Center, Loma Linda, CA, United States

(Dandamudi) Pulmonary and Critical Care Medicine, J L Pettis VA Medical Center, Loma Linda, CA, United States

Publisher

American Thoracic Society

Year of Publication

2019

Link to the Ovid Full Text or citation:

[Click here for full text options](https://ovidsp.ovid.com/ovidweb.cgi?T=JS&CSC=Y&NEWS=N&PAGE=fulltext&D=emed20&AN=630351075)

Link to the External Link Resolver:

[LibKey NHS](https://libkey.io/libraries/2789/openurl?genre=article&sid=OVID:emed20&genre=article&id=pmid:&id=doi:&issn=1535-4970&volume=199&issue=9&spage=&pages=&date=2019&title=American+Journal+of+Respiratory+and+Critical+Care+Medicine&atitle=Impact+of+pulmonary+rehabilitation+on+sleep+in+COPD+patients+as+measured+by+actigraphy&aulast=Thapamagar)

83.

Disease-specific fears and health status during pulmonary rehabilitation in patients with COPD.

Janssens T., Van De Moortel Z., Geidl W., Carl J., Pfeifer K., Lehbert N., Wittmann M., Schultz K., Von Leupoldt A.

Embase

Pneumologie. Conference: 60. Kongress der Deutschen Gesellschaft fur Pneumologie und Beatmungsmedizin e. V.. Munchen Germany. 73(SUPPPL. 1) (no pagination), 2019. Date of Publication: March 2019.

[Conference Abstract]

AN: 628475204

Anxiety and depression are prevalent in patients with COPD and negatively impact pulmonary rehabilitation (PR) outcomes, including health status. More recently, disease-specific fears have been put forward as an important predictor of health status in COPD, but their role in PR contexts remains poorly understood. Therefore, we tested how different disease-specific fears in patients with COPD evolve over the course of PR and how these fears relate to health status. Patients with COPD (n = 146) participated in a 3-week inpatient multidisciplinary PR program. At baseline, after PR and at 6-months follow-up, disease-specific fears were assessed with the COPD-Anxiety-Questionnaire (CAF), which includes the subscales fear of dyspnea, fear of physical activity, fear of disease progression, fear of social exclusion and sleep-related worries. Subjective health status and depressive symptoms were assessed with the COPD Assessment Test (CAT) and the Patient Health Questionnaire-9, respectively. After PR, all disease-specific fears were significantly reduced. For fear of dyspnea, fear of physical activity and fear of disease progression, this improvement was maintained at follow-up. After PR, significant improvements in CAT scores were observed, which were partially maintained at follow-up. Baseline levels of disease-specific fears predicted CAT scores at baseline, with patients with higher CAF-subscale scores (1 SD above mean) showing CAT scores that were 2.5 (fear of disease progression) to 3.8 (sleep-related worries) points higher than in patients with average levels of CAF-subscale scores. The effects of baseline CAF scores on CAT scores were maintained after PR and at follow-up. Controlling for potential confounding variables (sex, smoking status, age, FEV1%predicted and depressive symptoms) resulted in comparable effects in all analyses. The present findings demonstrate that disease-specific fears improve during PR. However, increased baseline levels of disease-specific fears continue to have a negative impact on health status after PR and at 6-months follow-up. This suggests that PR programs should include diagnosing and targeting of disease-specific fears to further improve the beneficial effects of PR on health status in patients with COPD.

Status

CONFERENCE ABSTRACT

Institution

(Janssens, Van De Moortel, Von Leupoldt) Health Psychology, University of Leuven (Geidl, Pfeifer) Fhedhch-Alexander-Universitat Erlangen-Nurnberg, Department fur Sportwissenschaft und Sport, Lehrstuhl Bewegung und Gesundheit

(Carl) Fhedhch-Alexander-Universitat Erlangen-Nurnberg, Institut fur Sportwissenschaft und Sport

(Lehbert, Wittmann, Schultz) Klinik Bad Reichenhall der Drv Bayern Sud

Publisher

Georg Thieme Verlag

Year of Publication

2019

Link to the Ovid Full Text or citation:

[Click here for full text options](https://ovidsp.ovid.com/ovidweb.cgi?T=JS&CSC=Y&NEWS=N&PAGE=fulltext&D=emed20&AN=628475204)

Link to the External Link Resolver:

[LibKey NHS](https://libkey.io/libraries/2789/openurl?genre=article&sid=OVID:emed20&genre=article&id=pmid:&id=doi:10.1055%2Fs-0039-1678081&issn=1438-8790&volume=73&issue=SUPPPL.+1&spage=&pages=&date=2019&title=Pneumologie&atitle=Disease-specific+fears+and+health+status+during+pulmonary+rehabilitation+in+patients+with+COPD&aulast=Janssens)

84.

Noninvasive ventilation in stable hypercapnic COPD: What is the evidence?.

Duiverman M.L.

Embase

ERJ Open Research. 4(2) (no pagination), 2018. Article Number: 00012-2018. Date of Publication: 01 Apr 2018.

[Review]

AN: 622116595

Long-term noninvasive ventilation (NIV) to treat chronic hypercapnic respiratory failure is still controversial in severe chronic obstructive pulmonary disease (COPD) patients. However, with the introduction of high-intensity NIV, important benefits from this therapy have also been shown in COPD. In this review, the focus will be on the arguments for long-term NIV at home in patients with COPD. The rise of (high-intensity) NIV in COPD and the randomised controlled trials showing positive effects with this mode of ventilation will be discussed. Finally, the challenges that might be encountered (both in clinical practice and in research) in further optimising this therapy, monitoring and following patients, and selecting the patients who might benefit most will be reviewed.

Copyright © ERS 2018.

Status

Embase

Institution

(Duiverman) Dept of Pulmonary Diseases/Home Mechanical Ventilation, University of Groningen, University Medical Center Groningen, Groningen, Netherlands (Duiverman) Groningen Research Institute of Asthma and COPD, University of Groningen, Groningen, Netherlands

Publisher

European Respiratory Society

Year of Publication

2018

Link to the Ovid Full Text or citation:

[Click here for full text options](https://ovidsp.ovid.com/ovidweb.cgi?T=JS&CSC=Y&NEWS=N&PAGE=fulltext&D=emed19&AN=622116595)

Link to the External Link Resolver:

[LibKey NHS](https://libkey.io/libraries/2789/openurl?genre=article&sid=OVID:emed19&genre=article&id=pmid:&id=doi:10.1183%2F23120541.00012-2018&issn=2312-0541&volume=4&issue=2&spage=00012-2018&pages=&date=2018&title=ERJ+Open+Research&atitle=Noninvasive+ventilation+in+stable+hypercapnic+COPD%3A+What+is+the+evidence%3F&aulast=Duiverman)

85.

Chronic obstructive pulmonary disease, sleep apnea and fatigues.

Kawada T.

Embase

Clinical Respiratory Journal. 12(9) (pp 2459), 2018. Date of Publication: September 2018.

[Letter]

AN: 624116922

PMID

30070774 [<https://www.ncbi.nlm.nih.gov/pubmed/?term=30070774>]

Status

Embase

Author NameID

Kawada, Tomoyuki; ORCID: <https://orcid.org/0000-0002-4426-4644>

Institution

(Kawada) Department of Hygiene and Public Health, Nippon Medical School, Tokyo, Japan

Publisher

Blackwell Publishing Ltd

Year of Publication

2018

Link to the Ovid Full Text or citation:

[Click here for full text options](https://ovidsp.ovid.com/ovidweb.cgi?T=JS&CSC=Y&NEWS=N&PAGE=fulltext&D=emed19&AN=624116922)

Link to the External Link Resolver:

[LibKey NHS](https://libkey.io/libraries/2789/openurl?genre=article&sid=OVID:emed19&genre=article&id=pmid:30070774&id=doi:10.1111%2Fcrj.12949&issn=1752-6981&volume=12&issue=9&spage=2459&pages=2459&date=2018&title=Clinical+Respiratory+Journal&atitle=Chronic+obstructive+pulmonary+disease%2C+sleep+apnea+and+fatigues&aulast=Kawada)

86.

Diagnosis and management of idiopathic pulmonary fibrosis: Thoracic society of Australia and New Zealand and lung foundation australia position statements summary.

Jo H.E., Prasad J.D., Troy L.K., Mahar A., Bleasel J., Ellis S.J., Chambers D.C., Holland A.E., Lake F.R., Keir G., Goh N.S., Wilsher M., de Boer S., Moodley Y., Grainge C., Whitford H.M., Chapman S.A., Reynolds P.N., Beatson D., Jones L.J., Hopkins P., Allan H.M., Glaspole I., Corte T.J.

Embase

Medical Journal of Australia. 208(2) (pp 82-88), 2018. Date of Publication: 05 Feb 2018.

[Article]

AN: 620614066

Introduction: Idiopathic pulmonary fibrosis (IPF) is a fibrosing interstitial lung disease associated with debilitating symptoms of dyspnoea and cough, resulting in respiratory failure, impaired quality of life and ultimately death. Diagnosing IPF can be challenging, as it often shares many features with other interstitial lung diseases. In this article, we summarise recent joint position statements on the diagnosis and management of IPF from the Thoracic Society of Australia and New Zealand and Lung Foundation Australia, specifically tailored for physicians across Australia and New Zealand. Main suggestions: * A comprehensive multidisciplinary team meeting is suggested to establish a prompt and precise IPF diagnosis. * Antifibrotic therapies should be considered to slow disease progression. However, enthusiasm should be tempered by the lack of evidence in many IPF subgroups, particularly the broader disease severity spectrum. * Non-pharmacological interventions including pulmonary rehabilitation, supplemental oxygen, appropriate treatment of comorbidities and disease-related symptoms remain crucial to optimal management. * Despite recent advances, IPF remains a fatal disease and suitable patients should be referred for lung transplantation assessment.

Copyright © 2018 AMPCo Pty Ltd. Produced with Elsevier B.V. All rights reserved.

PMID

29385965 [<https://www.ncbi.nlm.nih.gov/pubmed/?term=29385965>]

Status

Embase

Institution

(Jo, Troy, Mahar, Corte) Royal Prince Alfred Hospital, Sydney, NSW, Australia (Prasad, Ellis, Holland, Whitford, Glaspole) Alfred Hospital, Melbourne, VIC, Australia

(Prasad) Royal Melbourne Hospital, Melbourne, VIC, Australia

(Bleasel) University of Sydney, Sydney, NSW, Australia

(Chambers) University of Queensland, Brisbane, QLD, Australia

(Chambers) Prince Charles Hospital, Brisbane, QLD, Australia

(Holland) La Trobe University, Melbourne, VIC, Australia

(Lake, Moodley) University of Western Australia, Perth, WA, Australia

(Keir) Princess Alexandra Hospital, Brisbane, QLD, Australia

(Goh) Austin Health, Melbourne, VIC, Australia

(Wilsher) Auckland District Health Board, Auckland, New Zealand

(Wilsher) University of Auckland, Auckland, New Zealand

(de Boer) Auckland City Hospital, Auckland, New Zealand

(Grainge, Jones) John Hunter Hospital, Newcastle, NSW, Australia

(Chapman, Reynolds) Royal Adelaide Hospital, Adelaide, SA, Australia

(Beatson) AucklandNew Zealand

(Hopkins) Queensland Lung Transplant Service, Prince Charles Hospital, Brisbane, QLD, Australia

(Allan) Lung Foundation Australia, Brisbane, QLD, Australia

(Glaspole) Monash University, Melbourne, VIC, Australia

Publisher

Australasian Medical Publishing Co. Ltd (E-mail: ampco@ampco.com.au)

Clinical Trial Number

<https://clinicaltrials.gov/show/NCT02802345>

Year of Publication

2018

Link to the Ovid Full Text or citation:

[Click here for full text options](https://ovidsp.ovid.com/ovidweb.cgi?T=JS&CSC=Y&NEWS=N&PAGE=fulltext&D=emed19&AN=620614066)

Link to the External Link Resolver:

[LibKey NHS](https://libkey.io/libraries/2789/openurl?genre=article&sid=OVID:emed19&genre=article&id=pmid:29385965&id=doi:10.5694%2Fmja17.00799&issn=0025-729X&volume=208&issue=2&spage=82&pages=82-88&date=2018&title=Medical+Journal+of+Australia&atitle=Diagnosis+and+management+of+idiopathic+pulmonary+fibrosis%3A+Thoracic+society+of+Australia+and+New+Zealand+and+lung+foundation+australia+position+statements+summary&aulast=Jo)

87.

Chronic respiratory failure in patients with chronic obstructive pulmonary disease under home noninvasive ventilation: Real-life study.

Durao V., Grafino M., Pamplona P.

Embase

Pulmonology. 24(5) (pp 280-288), 2018. Date of Publication: September - October 2018.

[Article]

AN: 2000626574

Background: Home noninvasive ventilation (NIV) has been increasingly used in stable chronic obstructive pulmonary disease (COPD) with chronic hypercapnic respiratory failure (CHRF). However its effectiveness remains debatable.

Aim(s): To describe a follow-up of COPD patients under home NIV.

Method(s): Retrospective descriptive study based on a prospective 3-year database that included COPD patients under home NIV between August 2011 and July 2014.

Result(s): Within the 334 patients initially screened, 109 (32.6%) had COPD with a mean +/- SD post-bronchodilator FEV1 of 38.6 +/- 14.9% predicted; age of 65.6 +/- 9.6 years. The mean +/- SD duration of ventilation was 63.4 +/- 51.1 months. Heterogeneous comorbidities that can contribute to CHRF were not excluded: obstructive sleep apnea and obesity were the most prevalent. Sixty-two (56.9%) patients started NIV during admission with acute respiratory failure. During follow-up there was a significant increase in mean inspiratory positive airway pressure (IPAP) and respiratory rate (19.5 +/- 4.4 vs. 23.6 +/- 5.3 cmH2O and 10.7 +/- 5.2 vs. 15.2 +/- 1.4 breaths/min, respectively, p < 0.0001), with a significant improvement in hypercapnia (PaCO2: 52.9 +/- 7.7 vs. 49.5 +/- 7.5 mmHg, p < 0.0001), with 93.3% of patients compliant to NIV. Admissions and days spent in hospital for respiratory illness significantly decreased after institution of NIV (respectively, 1.2 +/- 1.1 vs. 0.7 +/- 1.8 and 15.0 +/- 16.8 vs. 8.8 +/- 19.4, p < 0.001). At final evaluation, patients with severe hypercapnia (n = 47; PaCO2 >=50 mmHg) performing NIV at higher pressures (n = 30; IPAP >=25 cmH2O) were more compliant (10.1 +/- 3.3 vs. 6.1 +/- 3.6 h/day). Three-year mortality was 24.8% (27 of 109 patients).

Conclusion(s): This is a real-life retrospective study in COPD patients with CHRF which results suggest benefit from home NIV. For most, NIV was effective and tolerable even at high pressures.

Copyright © 2018 Sociedade Portuguesa de Pneumologia

PMID

29628437 [<https://www.ncbi.nlm.nih.gov/pubmed/?term=29628437>]

Status

Embase

Institution

(Durao, Grafino, Pamplona) Servico Pneumologia, Hospital Pulido Valente, Centro Hospitalar Lisboa Norte, Lisboa, Portugal

Publisher

Elsevier Espana S.L.U

Year of Publication

2018

Link to the Ovid Full Text or citation:

[Click here for full text options](https://ovidsp.ovid.com/ovidweb.cgi?T=JS&CSC=Y&NEWS=N&PAGE=fulltext&D=emed19&AN=2000626574)

Link to the External Link Resolver:

[LibKey NHS](https://libkey.io/libraries/2789/openurl?genre=article&sid=OVID:emed19&genre=article&id=pmid:29628437&id=doi:10.1016%2Fj.pulmoe.2018.02.007&issn=2531-0429&volume=24&issue=5&spage=280&pages=280-288&date=2018&title=Pulmonology&atitle=Chronic+respiratory+failure+in+patients+with+chronic+obstructive+pulmonary+disease+under+home+noninvasive+ventilation%3A+Real-life+study&aulast=Durao)

88.

Reliability and validity of the Brief Pain Inventory in individuals with chronic obstructive pulmonary disease.

Chen Y.-W., HajGhanbari B., Road J.D., Coxson H.O., Camp P.G., Reid W.D.

Embase

European Journal of Pain (United Kingdom). 22(10) (pp 1718-1726), 2018. Date of Publication: November 2018.

[Article]

AN: 624376035

Background: Pain is prevalent in chronic obstructive pulmonary disease (COPD) and the Brief Pain Inventory (BPI) appears to be a feasible questionnaire to assess this symptom. However, the reliability and validity of the BPI have not been determined in individuals with COPD. This study aimed to determine the internal consistency, test-retest reliability and validity (construct, convergent, divergent and discriminant) of the BPI in individuals with COPD.

Method(s): In order to examine the test-retest reliability, individuals with COPD were recruited from pulmonary rehabilitation programmes to complete the BPI twice 1 week apart. In order to investigate validity, de-identified data was retrieved from two previous studies, including forced expiratory volume in 1-s, age, sex and data from four questionnaires: the BPI, short-form McGill Pain Questionnaire (SF-MPQ), 36-Item Short Form Survey (SF-36) and Community Health Activities Model Program for Seniors (CHAMPS) questionnaire.

Result(s): In total, 123 participants were included in the analyses (eligible data were retrieved from 86 participants and additional 37 participants were recruited). The BPI demonstrated excellent internal consistency and test-retest reliability. It also showed convergent validity with the SF-MPQ and divergent validity with the SF-36. The factor analysis yielded two factors of the BPI, which demonstrated that the two domains of the BPI measure the intended constructs. The BPI can also discriminate pain levels among COPD patients with varied levels of quality of life (SF-36) and physical activity (CHAMPS).

Conclusion(s): The BPI is a reliable and valid pain questionnaire that can be used to evaluate pain in COPD.

Significance: This study formally established the reliability and validity of the BPI in individuals with COPD, which have not been determined in this patient group. The results of this study provide strong evidence that assessment results from this pain questionnaire are reliable and valid.

Copyright © 2018 European Pain Federation - EFIC

PMID

29883526 [<https://www.ncbi.nlm.nih.gov/pubmed/?term=29883526>]

Status

Embase

Institution

(Chen, HajGhanbari) Department of Physical Therapy, University of British Columbia, Vancouver, BC, Canada (Road) Division of Respiratory Medicine, Department of Medicine, University of British Columbia, Vancouver, BC, Canada

(Coxson) Department of Radiology, Centre for Heart Lung Innovation, University of British Columbia, Vancouver, BC, Canada

(Camp) Department of Physical Therapy, Centre for Heart Lung Innovation, University of British Columbia, Vancouver, BC, Canada

(Reid) Department of Physical Therapy, University of Toronto, ON, Canada

(Reid) Interdivisional Department of Critical Care Medicine, University of Toronto, ON, Canada

(Reid) Toronto Rehabilitation Institute, Toronto, ON, Canada

Publisher

Blackwell Publishing Ltd

Year of Publication

2018

Link to the Ovid Full Text or citation:

[Click here for full text options](https://ovidsp.ovid.com/ovidweb.cgi?T=JS&CSC=Y&NEWS=N&PAGE=fulltext&D=emed19&AN=624376035)

Link to the External Link Resolver:

[LibKey NHS](https://libkey.io/libraries/2789/openurl?genre=article&sid=OVID:emed19&genre=article&id=pmid:29883526&id=doi:10.1002%2Fejp.1258&issn=1090-3801&volume=22&issue=10&spage=1718&pages=1718-1726&date=2018&title=European+Journal+of+Pain+(United+Kingdom)&atitle=Reliability+and+validity+of+the+Brief+Pain+Inventory+in+individuals+with+chronic+obstructive+pulmonary+disease&aulast=Chen)

89.

The effect of continuous positive airway pressure on pulmonary function may depend on the basal level of forced expiratory volume in 1 second.

Schreiber A., Surbone S., Malovini A., Mancini M., Cemmi F., Piaggi G., Ceriana P., Carlucci A.

Embase

Journal of Thoracic Disease. 10(12) (pp 6819-6827), 2018. Date of Publication: 01 Dec 2018.

[Article]

AN: 625807992

Background: The coexistence of chronic obstructive pulmonary disease (COPD) and obstructive sleep apnea (OSA), also referred to as overlap syndrome (OS), is associated with a high rate of morbidity, COPD exacerbations and mortality. Treatment with continuous positive airway pressure (CPAP) has proven to significantly decrease the rate of these complications. However, data concerning the effect of CPAP on pulmonary function are scarce and conflicting. The aim of our study was to evaluate the effect of 1 year of CPAP treatment on arterial blood gases (ABGs) and pulmonary function tests in patients with OS and its potential relationship with the baseline severity of airway obstruction. A secondary aim was to search for predictors of changes in the evaluated parameters.

Method(s): A retrospective study on a cohort of 92 patients (74 males) discharged from the Pulmonary Rehabilitation Unit of the Istituti Clinici Scientifici Maugeri in Pavia (Italy) from January 2013 to January 2016, with a diagnosis of OS and a prescription of CPAP, was conducted. Collected data at discharge were compared with 1-year follow-up data.

Result(s): After 1 year of CPAP, we observed the following: (I) a significant improvement in ABGs in all patients [median pO2 65.0 (59.0-70.0) vs. 71 (64.8-77.1) mmHg, pCO2 39.8 (36.2-43.5) vs. 38.3 (32.3-44.2) at baseline and after 1 year respectively, P<0.001], which was more pronounced in patients who were hypercapnic at baseline; (II) no significant change in respiratory function in the whole population; (III) a significant change in forced expiratory volume in 1 second (FEV1) only under and above a threshold of 79.1% of basal FEV1 with an opposite trend. In particular, patients with a basal FEV1 below that threshold significantly improved [median FEV1 70 (-70 to 200) mL, P=0.001], whereas patients with a basal FEV1 above the same threshold significantly worsened [median FEV1 -270 (-370 to -130) mL, P=3.05mu10-5].

Conclusion(s): A population of overlap patients treated with CPAP may experience a different change in airflow obstruction after 1 year depending on the severity of baseline obstruction.

Copyright © Journal of Thoracic Disease. All rights reserved.

Status

Embase

Institution

(Schreiber, Piaggi, Ceriana, Carlucci) Pulmonary Rehabilitation Unit, Istituti Clinici Scientifici Maugeri, Via Salvatore Maugeri 10, Pavia 27100, Italy (Surbone) Unita Operativa di Pneumologia, Ospedale Asilo Vittoria di Mortara, Azienda Socio Sanitaria Territoriale di Pavia, Italy

(Malovini) Laboratory of Informatics and Systems Engineering for Clinical Research, Istituti Clinici Scientifici Maugeri, Pavia, Italy

(Mancini) Direzione Sanitaria, Istituti Clinici Scientifici Maugeri, Pavia, Italy

(Cemmi) Unita Operativa di Pneumologia, Ospedale Pederzoli, Peschiera del Garda (Verona), Italy

Publisher

AME Publishing Company (E-mail: info@amepc.org)

Year of Publication

2018

Link to the Ovid Full Text or citation:

[Click here for full text options](https://ovidsp.ovid.com/ovidweb.cgi?T=JS&CSC=Y&NEWS=N&PAGE=fulltext&D=emed19&AN=625807992)

Link to the External Link Resolver:

[LibKey NHS](https://libkey.io/libraries/2789/openurl?genre=article&sid=OVID:emed19&genre=article&id=pmid:&id=doi:10.21037%2Fjtd.2018.10.103&issn=2072-1439&volume=10&issue=12&spage=6819&pages=6819-6827&date=2018&title=Journal+of+Thoracic+Disease&atitle=The+effect+of+continuous+positive+airway+pressure+on+pulmonary+function+may+depend+on+the+basal+level+of+forced+expiratory+volume+in+1+second&aulast=Schreiber)

90.

The interrelations among aspects of dyspnea and symptoms of depression in COPD patients - a network analysis.

Schuler M., Wittmann M., Faller H., Schultz K.

Embase

Journal of Affective Disorders. 240 (pp 33-40), 2018. Date of Publication: November 2018.

[Article]

AN: 2000971201

Background: Depression is a frequent comorbidity in COPD. COPD symptoms such as dyspnea may play an important role in the causal relationship between COPD and depression. We investigated the interrelations among different aspects of dyspnea and other COPD parameters and symptoms of depression in COPD patients.

Method(s): This is a secondary analysis of N = 590 COPD patients. At the beginning (T0) and the end (T1) of a 3-week inpatient pulmonary rehabilitation, dyspnea aspects intensity (BORG scale), frequency (2 CCQ items), functioning (CCQ-function) and cognitive/emotional response (2 SGRQ items) as well as cough (2 CCQ items), functional capacity (6MWD), lung function (FEV1) and symptoms of depression (PHQ-9) were assessed. Regression analyses with PHQ-9 sum score as dependent variable as well as network analysis using PHQ-9 single items were performed. Structural invariance over time was examined.

Result(s): Dyspnea frequency, function, and cognitive/emotional response showed conditional independent relationships with PHQ-9 sum score. Network analysis showed that dyspnea frequency and dyspnea functioning were primarily associated with somatic depression symptoms (for example, sleep problems, loss of energy), while cognitive/emotional response was primarily related to cognitive-affective depression symptoms (for example, feeling down/depressed/hopeless). Regression parameters, network structure and network global strength did not differ between T0 and T1.

Limitation(s): Models are based on between-person relationships. Results should be confirmed using time-series data.

Conclusion(s): Dyspnea and depression seem to be interrelated through a variety of different and complex pathways in COPD patients. Results may be used to explain intervention effects and develop new intervention strategies to reduce depression in COPD.

Copyright © 2018 Elsevier B.V.

PMID

30048834 [<https://www.ncbi.nlm.nih.gov/pubmed/?term=30048834>]

Status

Embase

Author NameID

Schuler, Michael; ORCID: <https://orcid.org/0000-0001-6502-9232>

Institution

(Schuler, Faller) Department of Medical Psychology and Psychotherapy, Medical Sociology and Rehabilitation Science, University of Wurzburg, Wurzburg, Germany (Wittmann, Schultz) Klinik Bad Reichenhall, Center of Rehabilitation, Pulmonology and Orthopedics, Bad Reichenhall, Germany

Publisher

Elsevier B.V.

Year of Publication

2018

Link to the Ovid Full Text or citation:

[Click here for full text options](https://ovidsp.ovid.com/ovidweb.cgi?T=JS&CSC=Y&NEWS=N&PAGE=fulltext&D=emed19&AN=2000971201)

Link to the External Link Resolver:

[LibKey NHS](https://libkey.io/libraries/2789/openurl?genre=article&sid=OVID:emed19&genre=article&id=pmid:30048834&id=doi:10.1016%2Fj.jad.2018.07.021&issn=0165-0327&volume=240&issue=&spage=33&pages=33-40&date=2018&title=Journal+of+Affective+Disorders&atitle=The+interrelations+among+aspects+of+dyspnea+and+symptoms+of+depression+in+COPD+patients+-+a+network+analysis&aulast=Schuler)

91.

COPD stands for complex obstructive pulmonary disease.

Houben-Wilke S., Augustin I.M., Vercoulen J.H., van Ranst D., de Vaate E.B., Wempe J.B., Spruit M.A., Wouters E.F.M., Franssen F.M.E.

Embase

European Respiratory Review. 27(148) (no pagination), 2018. Article Number: 180027. Date of Publication: 30 Jun 2018.

[Review]

AN: 622497528

Chronic obstructive pulmonary disease (COPD) has extensively been reported as a complex disease affecting patients' health beyond the lungs with a variety of intra- and extrapulmonary components and considerable variability between individuals. This review discusses the assessment of this complexity and underlines the importance of transdisciplinary management programmes addressing the physical, emotional and social health of the individual patient. COPD management is challenging and requires advanced, sophisticated strategies meeting the patient's individual needs. Due to the heterogeneity and complexity of the disease leading to non-linear and consequently poorly predictable treatment responses, multidimensional patient profiling is crucial to identify the right COPD patient for the right treatment. Current methods are often restricted to general, well-known and commonly used assessments neglecting potentially relevant (interactions between) individual, unique "traits] to finally ensure personalised treatment. Dynamic, personalised and holistic approaches are needed to tackle this multifaceted disease and to ensure personalised medicine and valuebased healthcare.

Copyright © ERS 2018.

PMID

29875138 [<https://www.ncbi.nlm.nih.gov/pubmed/?term=29875138>]

Status

Embase

Institution

(Houben-Wilke) Dutch Lung Centers, Horn, Netherlands (Augustin, Spruit, Wouters, Franssen) CIRO+, Horn, Netherlands

(Vercoulen) Radboud University Medical Center, Nijmegen, Netherlands

(van Ranst) Revant, Breda, Netherlands

(de Vaate) Merem, Hilversum, Netherlands

(Wempe) University Medical Center Groningen, Groningen, Netherlands

(Spruit, Wouters, Franssen) Dept of Respiratory Medicine, Maastricht University Medical Center, Maastricht, Netherlands

Publisher

European Respiratory Society (E-mail: info@ersnet.org)

Year of Publication

2018

Link to the Ovid Full Text or citation:

[Click here for full text options](https://ovidsp.ovid.com/ovidweb.cgi?T=JS&CSC=Y&NEWS=N&PAGE=fulltext&D=emed19&AN=622497528)

Link to the External Link Resolver:

[LibKey NHS](https://libkey.io/libraries/2789/openurl?genre=article&sid=OVID:emed19&genre=article&id=pmid:29875138&id=doi:10.1183%2F16000617.0027-2018&issn=0905-9180&volume=27&issue=148&spage=180027&pages=&date=2018&title=European+Respiratory+Review&atitle=COPD+stands+for+complex+obstructive+pulmonary+disease&aulast=Houben-Wilke)

92.

Prevalence and Predictors of Obstructive Sleep Apnea in Patients with Chronic Obstructive Pulmonary Disease Undergoing Inpatient Pulmonary Rehabilitation.

Schreiber A., Cemmi F., Ambrosino N., Ceriana P., Lastoria C., Carlucci A.

Embase

COPD: Journal of Chronic Obstructive Pulmonary Disease. 15(3) (pp 265-270), 2018. Date of Publication: 04 May 2018.

[Article]

AN: 624005673

The aim of our study was to evaluate the prevalence and predictors of obstructive sleep apnea (OSA) in patients with chronic obstructive pulmonary disease (COPD) undergoing inpatient pulmonary rehabilitation programs (PRPs). A retrospective data review of consecutive stable patients with a known diagnosis of COPD, admitted for PRP between January 2007 and December 2013. Full overnight polysomnography (PSG) and Epworth Sleepiness Scale (ESS) were assessed in all patients. Out of 422 evaluated patients, 190 (45%) showed an Apnea Hypopnea Index (AHI) >= 15 events/hour and underwent OSA treatment. Patients with OSA were significantly younger and had a less severe airway obstruction as compared to patients without OSA. There were no significant differences in cardiac comorbidities nor in arterial blood gases. As expected, patients with OSA showed significantly more severe diurnal symptoms, as assessed by the ESS and higher body mass index (BMI). However, only 69 out of 190 patients with OSA (36.3%) showed an ESS >10, whereas 25% of them had BMI <=25 and 41% of them had a BMI <30. In all, 68% of patients with OSA were discharged with continuous positive airway pressure (CPAP), 15% with Bilevel ventilation, and 17% without any ventilatory treatment. In conclusion, in the population studied, the combination of OSA and COPD was frequent. BMI and ESS values commonly considered cutoff values for the prediction of OSA in the general population may not be accurate in a subgroup of patients with COPD.

Copyright © 2018, © 2018 Taylor & Francis Group, LLC.

PMID

30239226 [<https://www.ncbi.nlm.nih.gov/pubmed/?term=30239226>]

Status

In-Process

Institution

(Schreiber, Ceriana, Lastoria, Carlucci) Respiratory Intensive Care Unit and Pulmonary Rehabilitation Unit, Istituti Clinici Scientifici Maugeri IRCCS, Istituto Scientifico di Pavia, Pavia, Italy (Cemmi) Unita Operativa di Pneumologia, Ospedale Pederzoli, Peschiera del Garda, Verona, Italy

(Ambrosino) Istituti Clinici Scientifici Maugeri IRCCS, Istituto Scientifico di Montescano, Montescano, Pavia, Italy

Publisher

Taylor and Francis Ltd

Year of Publication

2018

Link to the Ovid Full Text or citation:

[Click here for full text options](https://ovidsp.ovid.com/ovidweb.cgi?T=JS&CSC=Y&NEWS=N&PAGE=fulltext&D=emed19&AN=624005673)

Link to the External Link Resolver:

[LibKey NHS](https://libkey.io/libraries/2789/openurl?genre=article&sid=OVID:emed19&genre=article&id=pmid:30239226&id=doi:10.1080%2F15412555.2018.1500533&issn=1541-2555&volume=15&issue=3&spage=265&pages=265-270&date=2018&title=COPD%3A+Journal+of+Chronic+Obstructive+Pulmonary+Disease&atitle=Prevalence+and+Predictors+of+Obstructive+Sleep+Apnea+in+Patients+with+Chronic+Obstructive+Pulmonary+Disease+Undergoing+Inpatient+Pulmonary+Rehabilitation&aulast=Schreiber)

93.

Daily use of guaifenesin (Mucinex) in a patient with chronic bronchitis and pathologic mucus hypersecretion: A case report.

Storms W.W., Miller J.E.

Embase

Respiratory Medicine Case Reports. 23 (pp 156-157), 2018. Date of Publication: 2018.

[Article]

AN: 620898553

We report an improvement in symptoms and quality of life with long-term use of guaifenesin for the treatment of mucus-related symptoms in a patient with chronic bronchitis, who presented with mucus hypersecretion, cough and dyspnea.

Copyright © 2018 The Authors

Status

Embase

Institution

(Storms) The William Storms Allergy Clinic, 1625 Medical Center Point, Suite 190, Colorado Springs, CO 80907, United States (Miller) SRxA Strategic Pharmaceutical Advisors, 1750 Tysons Boulevard, Suite 1500, McLean, VA 22102, United States

Publisher

W.B. Saunders Ltd

Year of Publication

2018

Link to the Ovid Full Text or citation:

[Click here for full text options](https://ovidsp.ovid.com/ovidweb.cgi?T=JS&CSC=Y&NEWS=N&PAGE=fulltext&D=emed19&AN=620898553)

Link to the External Link Resolver:

[LibKey NHS](https://libkey.io/libraries/2789/openurl?genre=article&sid=OVID:emed19&genre=article&id=pmid:&id=doi:10.1016%2Fj.rmcr.2018.02.009&issn=2213-0071&volume=23&issue=&spage=156&pages=156-157&date=2018&title=Respiratory+Medicine+Case+Reports&atitle=Daily+use+of+guaifenesin+(Mucinex)+in+a+patient+with+chronic+bronchitis+and+pathologic+mucus+hypersecretion%3A+A+case+report&aulast=Storms)

94.

Prevalence of overlap of COPD and OSAS in a Pulmonary Rehabilitation Centre.

Schreiber A.F., Cemmi F., Lastoria C., Ceriana P., Balestrino A., Carlucci A.

Embase

European Respiratory Journal. Conference: European Respiratory Society International Congress, ERS 2018. Paris France. 52(Supplement 62) (no pagination), 2018. Date of Publication: September 2018.

[Conference Abstract]

AN: 626629721

Background: The coexistence of obstructive sleep apnea syndrome (OSAS) and chronic obstructive pulmonary disease (COPD), namely overlap syndrome (OS), is associated with high morbidity, exacerbation rate and mortality. Data on its prevalence are conflicting and clear screening recommendations are still lacking; however a BMI<35 and an ESS<10 are often used to exclude OSAS in practice.

Aim(s): To evaluate the prevalence of OSAS in a series of patients with COPD admitted to a Pulmonary Rehabilitation centre.

Method(s): 422 consecutive COPD patients were prospectively screened for the concomitant presence of OSAS. An obstructive apnea-hypopnea index >=15 events/hour was considered diagnostic.

Result(s): 190 out of 422 COPD patients (45%) showed concomitant OSAS. OS patients were younger (mean age 69+/-9 vs 72+/-8, p<0.0001), had a higher body mass index (BMI) (31+/-7 vs 26+/-5, p<0.0001), milder obstruction (FEV1% 58+/-21 vs 44+/-18, p<0.0001), more severe symptoms (Epworth sleepiness scale ESS 6.2+/-4.4 vs 4.1+/-3.1, p<0.0001) and a higher oxygen desaturation index (ODI) (36+/-21 vs 9+/-11/h, p<0.0001) compared to patients with COPD alone. Moreover, 35% of OS patients showed frequent exacerbations. A multivariate logistic regression analysis revealed that BMI and ESS were independent predictors of OSAS (OR 1.12, p=0.05, OR 1.06, p=0.003 respectively). However, 64% of the OS patients showed an ESS<10 and 45% of them had a BMI<35.

Conclusion(s): OSAS may be relatively frequent in COPD patients hospitalized for pulmonary rehabilitation. In fact in our study it was present in approximately 50% of patients. The standard threshold values of BMI and ESS may not be accurate screening tools to discriminate the occurrence of OSAS in this population.

Status

CONFERENCE ABSTRACT

Institution

(Schreiber, Lastoria, Ceriana, Balestrino, Carlucci) Respiratory Intensive Care Unit and Pulmonary Rehabilitation Unit, Istituti Clinici Scientifici Maugeri IRCCS, Pavia, Italy (Cemmi) Unita Operativa di Pneumologia, Ospedale Pederzoli, Peschiera del Garda (Verona), Italy

Publisher

European Respiratory Society

Year of Publication

2018

Link to the Ovid Full Text or citation:

[Click here for full text options](https://ovidsp.ovid.com/ovidweb.cgi?T=JS&CSC=Y&NEWS=N&PAGE=fulltext&D=emed19&AN=626629721)

Link to the External Link Resolver:

[LibKey NHS](https://libkey.io/libraries/2789/openurl?genre=article&sid=OVID:emed19&genre=article&id=pmid:&id=doi:10.1183%2F13993003.congress-2018.PA2494&issn=1399-3003&volume=52&issue=Supplement+62&spage=&pages=&date=2018&title=European+Respiratory+Journal&atitle=Prevalence+of+overlap+of+COPD+and+OSAS+in+a+Pulmonary+Rehabilitation+Centre&aulast=Schreiber)

95.

A prospective observational study on long-term non invasive ventilation (L-T NIV) in COPD patients attending an inpatient pulmonary rehabilitation program (PRP).

Mannini C., Campana B., Banfi P.I., Volpato E., Arcadu A., Chellini E., Romagnoli I., Lanini B., Gigliotti F.

Embase

European Respiratory Journal. Conference: European Respiratory Society International Congress, ERS 2018. Paris France. 52(Supplement 62) (no pagination), 2018. Date of Publication: September 2018.

[Conference Abstract]

AN: 626628751

The onset of L-T NIV is often considered for patients with hypercapnic respiratory failure (HRF) due to COPD during an inpatient PRP. But despite the success of NIV in treating acute COPD exacerbations, its long-term use in stable COPD is still debated.

Aim(s): To investigate reasons for starting L-T NIV and its subsequent effects in COPD patients admitted to an inpatient PRP.

Method(s): Ongoing, multicentric, observational study of patients with HRF due to COPD adapted to L-T NIV during an inpatient PRP. At the time of the onset patients with alternative causes of HRF are excluded and clinical data, pulmonary function test, blood gases, sleep study and functional evaluation are collected. Preliminary Results refer to enrollment period (April 2017-December 2017):54 consecutive COPD patients were enrolled. 33 of them (mean [SD] age 73 [7,87] years, BMI of 24,55 [4,62], FEV1 34,09 % [6,36], pH 7,358[0.045], PaCO2 61 mmHg [8,41]) were eligible. 13 patients started L-T NIV for recurrent exacerbations of HRF requiring NIV; 12 for failed weaning from in hospital NIV; 4 for stable hypercapnia (PaCO2 > 55 mmHg); 3 after weaning from invasive mechanical ventilation. The mean ventilator settings were an inspiratory airway pressure of 17,7 [2,59]cmH2O, an expiratory positive airway pressure of 6,35[1,3]cmH2O and a backup rate of 12,06 [1,7] bpm. At the end of PRP patients showed a mean PH 7,405 [0,02] and mean PaCO2 51,10 mmHg [6,76].

Conclusion(s): Among COPD patients with HRF attending an inpatient PRP, recurrent exacerbation requiring NIV and failed weaning from hospital NIV were the most frequent reasons for starting L-T NIV.

Status

CONFERENCE ABSTRACT

Institution

(Mannini, Chellini, Romagnoli, Lanini, Gigliotti) Pulmonary Rehabilitation Unit IRCCS Fondazione Don Gnocchi, Firenze, Italy (Campana) Pulmonary Rehabilitation Unit IRCCS Fondazione Don Gnocchi, Sant'Angelo dei Lombardi, Italy

(Banfi, Volpato, Arcadu) Pulmonary Rehabilitation Unit IRCCS Fondazione Don Gnocchi, Milano, Italy

Publisher

European Respiratory Society

Year of Publication

2018

Link to the Ovid Full Text or citation:

[Click here for full text options](https://ovidsp.ovid.com/ovidweb.cgi?T=JS&CSC=Y&NEWS=N&PAGE=fulltext&D=emed19&AN=626628751)

Link to the External Link Resolver:

[LibKey NHS](https://libkey.io/libraries/2789/openurl?genre=article&sid=OVID:emed19&genre=article&id=pmid:&id=doi:10.1183%2F13993003.congress-2018.PA840&issn=1399-3003&volume=52&issue=Supplement+62&spage=&pages=&date=2018&title=European+Respiratory+Journal&atitle=A+prospective+observational+study+on+long-term+non+invasive+ventilation+(L-T+NIV)+in+COPD+patients+attending+an+inpatient+pulmonary+rehabilitation+program+(PRP)&aulast=Mannini)

96.

Effects of pulmonary rehabilitation on sleep quality in patients with chronic obstructive pulmonary disease (COPD).

Roberts M., Cho J., Wheatley J.R.

Embase

American Journal of Respiratory and Critical Care Medicine. Conference: American Thoracic Society International Conference, ATS 2018. San Diego, CA United States. 197(MeetingAbstracts) (no pagination), 2018. Date of Publication: 2018.

[Conference Abstract]

AN: 622970882

Rationale: Poor sleep quality is a common complaint reported by patients with chronic obstructive pulmonary disease (COPD). Exercise has been shown to improve sleep however there are conflicting reports regarding the effects of pulmonary rehabilitation (PR) on sleep quality. The aim of this study was to examine sleep quality before and after PR, and identify patient characteristics associated with a clinically significant improvement in sleep quality following PR.

Method(s): Retrospective chart review of all patients with a diagnosis of COPD who completed PR in Western Sydney from January 2012 to May 2017. The primary outcome was sleep quality measured by Pittsburgh Sleep Quality Index (PSQI; minimal clinically important difference - MCID>=3 units). We identified patients with poor sleep quality at baseline (PSQI>5 units), and compared baseline and post-PR characteristics of patients who improved PSQI following PR by >=3 units (i.e. responders) with those who did not (i.e. non-responders). We used paired t-tests for within-group comparisons, and Mann-Whitney tests for between-group comparisons. Data presented as mean+/-SD. p<0.05 was considered significant.

Result(s):. Complete data were available for 329 patients (52% male, 69.5+/-8.9 years, FEV1% predicted 48+/-16%). 219 (67%) had poor sleep quality with PSQI>5. Following PR, the group mean PSQI decreased by 0.95+/-3.14 units (p<0.0001) and Epworth Sleepiness Scale decreased by 0.63+/-4.07 units (p=0.005). 88 of 219 patients (40%) with poor sleep quality at baseline improved their PSQI by >=3 units following PR (i.e. responders). There was no difference at baseline between responders and non-responders for age, gender, lung function, six minute walk distance (6MWD), mood as measured by the Hospital Anxiety and Depression scale (HADs) or quality of life as measured by the St George Respiratory Questionnaire (SGRQ) - all p>0.05. However following PR, responders had a greater 6MWD compared with non-responders by 38.3m (p=0.005) and an improved SGRQ total score by 5.3 units (p=0.005).

Conclusion(s): Sleep quality improves following PR but is highly variable among patients. Greater gains in 6MWD and SGRQ occur in patients who have had a clinically significant improvement in sleep quality following PR. Future intervention studies targeting improved sleep quality may help to maximise the benefits of a PR program for patients with COPD.

Status

CONFERENCE ABSTRACT

Institution

(Roberts, Cho) Department of Respiratory and Sleep Medicine, Westmead Hospital, Wentworthville, Australia (Wheatley) Respiratory and Sleep Medicine, Westmead Hospital, Wentworthville, NSW, Australia

Publisher

American Thoracic Society

Year of Publication

2018

Link to the Ovid Full Text or citation:

[Click here for full text options](https://ovidsp.ovid.com/ovidweb.cgi?T=JS&CSC=Y&NEWS=N&PAGE=fulltext&D=emed19&AN=622970882)

Link to the External Link Resolver:

[LibKey NHS](https://libkey.io/libraries/2789/openurl?genre=article&sid=OVID:emed19&genre=article&id=pmid:&id=doi:&issn=1535-4970&volume=197&issue=MeetingAbstracts&spage=&pages=&date=2018&title=American+Journal+of+Respiratory+and+Critical+Care+Medicine&atitle=Effects+of+pulmonary+rehabilitation+on+sleep+quality+in+patients+with+chronic+obstructive+pulmonary+disease+(COPD)&aulast=Roberts)

97.

Pulmonary rehabilitation does not improve objective measures of sleep quality in people with chronic obstructive pulmonary disease.

Cox N.S., Pepin V., Burge A., Mahal A., Hill C.J., Lee A., Moore R., Nicolson C., O'Halloran P., Lahham A., Gillies R., McDonald C.F., Holland A.E.

Embase

American Journal of Respiratory and Critical Care Medicine. Conference: American Thoracic Society International Conference, ATS 2018. San Diego, CA United States. 197(MeetingAbstracts) (no pagination), 2018. Date of Publication: 2018.

[Conference Abstract]

AN: 622968928

Rationale: More than 50% of people with chronic obstructive pulmonary disease (COPD) report poor sleep quality which has been associated with increased morbidity and mortality. There are varying reports of the effect of pulmonary rehabilitation on self-reported sleep quality. We aimed to assess the effect of pulmonary rehabilitation on objectively measured sleep quality in people with COPD.

Method(s): This is a secondary analysis of data collected as part of a randomised controlled trial comparing home-based to centre-based pulmonary rehabilitation for COPD. Sleep quality was assessed objectively using the SenseWear Armband (SWA, Bodymedia USA), worn for 7 days before and after an 8-week PR programme. Sleep characteristics were derived from accelerometer positional data and registration of sleep state by the SWA, determined from energy expenditure.

Result(s): 33 participants (17 male; mean+/-SD age 68+/-11 years, FEV1 56+/-21 %predicted) had paired pre and post pulmonary rehabilitation sleep data. Twenty participants received centre-based pulmonary rehabilitation and n=13 were allocated to home-based pulmonary rehabilitation. Pre-pulmonary rehabilitation median [interquartile range] sleep onset latency (SOL) was 25 [9 to 41] minutes, total sleep time (TST) 390 [339 to 421] minutes and wake after sleep onset (WASO) 74 [43 to 106] minutes. Sleep efficiency (SE) was low (75 [64 to 84]%). No significant differences were seen in any sleep parameter (SOL, TST, WASO, SE) after pulmonary rehabilitation (all p>0.10), between intervention groups (all p>0.20), or within pulmonary rehabilitation groups (all p=0.13). There was no association between sleep parameters and measures of quality of life or function before or after pulmonary rehabilitation. Attendance at a greater proportion of pulmonary rehabilitation sessions was moderately associated with more TST at end rehabilitation (rs=0.5, p=0.009).

Conclusion(s): Sleep quality, measured objectively using actigraphy, did not improve after an 8-week pulmonary rehabilitation programme in individuals with COPD. Whether ongoing participation in regular exercise training beyond the duration of pulmonary rehabilitation may influence sleep quality is yet to be determined.

Status

CONFERENCE ABSTRACT

Institution

(Cox) School of Physiotherapy, La Trobe University and Institute for Breathing and Sleep, Melbourne, Australia (Pepin) Exercise Science, Concordia University, Montreal, QC, Canada

(Burge, Nicolson) Physiotherapy, La Trobe University and Alfred Health, Melbourne, Australia

(Mahal) School of Population and Global Health, University of Melbourne, Melbourne, Australia

(Hill, Moore, Gillies) Physiotherapy, Austin Health, Melbourne, Australia

(Lee) Physiotherapy, La Trobe University and Alfred Health, Institute for Breathing and Sleep, Melbourne, Australia

(O'Halloran) Psychology and Public Health, La Trobe University, Melbourne, Australia

(Lahham) Physiotherapy, La Trobe University, Institute for Breathing and Sleep, Melbourne, Australia

(McDonald) Austin Hospital, Institute for Breathing and Sleep, University of Melbourne, Melbourne, Australia

(Holland) La Trobe University and Alfred Health, Institute for Breathing and Sleep, Melbourne, Australia

Publisher

American Thoracic Society

Year of Publication

2018

Link to the Ovid Full Text or citation:

[Click here for full text options](https://ovidsp.ovid.com/ovidweb.cgi?T=JS&CSC=Y&NEWS=N&PAGE=fulltext&D=emed19&AN=622968928)

Link to the External Link Resolver:

[LibKey NHS](https://libkey.io/libraries/2789/openurl?genre=article&sid=OVID:emed19&genre=article&id=pmid:&id=doi:&issn=1535-4970&volume=197&issue=MeetingAbstracts&spage=&pages=&date=2018&title=American+Journal+of+Respiratory+and+Critical+Care+Medicine&atitle=Pulmonary+rehabilitation+does+not+improve+objective+measures+of+sleep+quality+in+people+with+chronic+obstructive+pulmonary+disease&aulast=Cox)

98.

Spontaneous intercostal lung hernia: A case of an unusual presentation.

Madanieh A., Faiz A., Acosta-Sanchez I., Banks D.

Embase

American Journal of Respiratory and Critical Care Medicine. Conference: American Thoracic Society International Conference, ATS 2018. San Diego, CA United States. 197(MeetingAbstracts) (no pagination), 2018. Date of Publication: 2018.

[Conference Abstract]

AN: 622966442

Introduction A spontaneous pulmonary hernia is defined as a protrusion of lung parenchyma through an intercostal defect in the chest wall without evidence of chest trauma. This condition is rare, with only few cases reported in current literature. Furthermore, the presentation of flank pain and ecchymosis over the flanks as the initial symptoms of spontaneous intercostal lung hernia has seldom been described. In this report, we present an uncommon presentation of spontaneous intercostal lung hernia. Case Presentation A 73-year-old man with a history significant for obesity, chronic obstructive pulmonary disease and obstructive sleep apnea presented with a one-week-history of left flank pain. His history was significant for multiple violent coughing spells for two weeks, and a large bruise over the left flank which he noticed two days prior to presentation. He denied any history of chest or abdominal trauma. The patient was in no acute distress, and vital signs were unremarkable. Examination revealed a large ecchymosis extending anteriorly from the left lateral T8 region into the left lower quadrant of the abdomen and spreading posteriorly to the left flank. A protruding bulge at the left 7th-8th intercostal space between the posterior and middle axillary lines was also appreciated. Auscultation revealed decreased breath sounds at the left lung base. Complete blood count, comprehensive metabolic panel and urinalysis were normal. Chest radiograph showed no significant findings. A chest computed tomography (CT) showed a partial herniation of the left lower lobe through the posterolateral 7th-8th intercostal space, with no evidence of rib fractures or parenchymal infiltrates (Figure below). (Figure presented) Owing to increased risk of strangulation, surgical repair was performed and the post-operative course was uneventful. The patient was discharged for pulmonary rehabilitation. Discussion A pulmonary hernia involves the protrusion of a portion of the lung through a weak anatomical structure and is often associated with an increased intra-thoracic pressure. Symptoms of spontaneous pulmonary herniation may include shortness of breath, chest pain, cough, hemoptysis, and rarely, flank hematomas as seen in our patient. Diagnosis is made via chest CT showing the protrusion of the lung through the lax chest wall or rarely abdominal wall. Lung hernias are typically managed conservatively with bandaging, analgesia, and antitussives. Surgical intervention is reserved for cases where the herniation is large, there is concern for strangulation or when there is considerable disability. Prognosis after surgical intervention remains excellent.

Status

CONFERENCE ABSTRACT

Institution

(Madanieh, Faiz, Acosta-Sanchez, Banks) University of Central Florida College of Medicine, Orlando, FL, United States

Publisher

American Thoracic Society

Year of Publication

2018

Link to the Ovid Full Text or citation:

[Click here for full text options](https://ovidsp.ovid.com/ovidweb.cgi?T=JS&CSC=Y&NEWS=N&PAGE=fulltext&D=emed19&AN=622966442)

Link to the External Link Resolver:

[LibKey NHS](https://libkey.io/libraries/2789/openurl?genre=article&sid=OVID:emed19&genre=article&id=pmid:&id=doi:&issn=1535-4970&volume=197&issue=MeetingAbstracts&spage=&pages=&date=2018&title=American+Journal+of+Respiratory+and+Critical+Care+Medicine&atitle=Spontaneous+intercostal+lung+hernia%3A+A+case+of+an+unusual+presentation&aulast=Madanieh)

99.

Pulmonary rehabilitation and sleep quality in patients with COPD. (repeat)

Roberts M., Cho J., Wheatley J.

Embase

Respirology. Conference: Australia and New Zealand Society of Respiratory Science and the Thoracic Society of Australia and New Zealand Annual Scientific Meeting , ANZSRS/TSANZ 2018. Adelaide, SA Australia. 23(Supplement 1) (pp 118), 2018. Date of Publication: March 2018.

[Conference Abstract]

AN: 622091450

Introduction/Aim: Poor sleep quality is a common complaint reported by patients with COPD. Exercise has been shown to improve sleep quality. However, the relationship between pulmonary rehabilitation (PR) and improved sleep quality is unknown. We examined sleep quality in patients with COPD before and after PR, to identify factors associated with improvement in sleep quality following PR.

Method(s): We performed a retrospective chart review of all patients with COPD who completed PR in Western Sydney from January 2012 to May 2017. Our primary outcome was sleep quality measured by Pittsburgh Sleep Quality Index (PSQI). We identified patients with poor sleep quality at baseline (PSQI>5 units), and compared baseline and post-PR characteristics of patients whose PSQI improved following PR by >=3 units (responders) with those who did not (non-responders). Data were compared using paired t-tests within group, and Mann-Whitney tests between groups. Data presented as mean+/-SD. p<0.05 was significant.

Result(s): Data were available for 329 patients (52% male, 69.5+/-8.9 years, FEV1% predicted 48+/-16%). 219 (67%) had poor sleep quality. Following PR, group mean PSQI decreased by 0.95+/-3.14 units (p<0.0001). 88 of 219 patients with poor sleep quality at baseline improved PSQI by >=3 units following PR. There was no difference at baseline between responders and non-responders for age, gender, lung function, 6MWD, mood or quality of life (QOL) (all p>0.05). However, following PR, responders had a greater 6MWD compared with nonresponders by 38.3m and an improved QOL score by 5.3 units (both p=0.005).

Conclusion(s): Sleep quality improves following PR for COPD, but is highly variable between individuals. Greater gains in 6MWD and QOL were also seen in association with a clinically significant improvement in sleep quality following PR. Future intervention studies targeting improved sleep quality for patients with COPD may help to maximise the benefits of a PR program.

Status

CONFERENCE ABSTRACT

Institution

(Roberts, Cho, Wheatley) Westmead Hospital, Wentworthville, NSW, Australia (Roberts, Cho, Wheatley) Ludwig Engel Centre for Respiratory Research, Westmead Institute of Medical Research, Westmead, NSW, Australia

(Cho, Wheatley) University of Sydney, Westmead Hospital, Westmead, NSW, Australia

Publisher

Blackwell Publishing

Year of Publication

2018

Link to the Ovid Full Text or citation:

[Click here for full text options](https://ovidsp.ovid.com/ovidweb.cgi?T=JS&CSC=Y&NEWS=N&PAGE=fulltext&D=emed19&AN=622091450)

Link to the External Link Resolver:

[LibKey NHS](https://libkey.io/libraries/2789/openurl?genre=article&sid=OVID:emed19&genre=article&id=pmid:&id=doi:10.1111%2Fresp.13268&issn=1440-1843&volume=23&issue=Supplement+1&spage=118&pages=118&date=2018&title=Respirology&atitle=Pulmonary+rehabilitation+and+sleep+quality+in+patients+with+COPD&aulast=Roberts)

100.

Participation in pulmonary rehabilitation did not significantly alter time use patterns in people with COPD.

Hunt T., Williams M., Olds T., Dumuid D.

Embase

Respirology. Conference: Australia and New Zealand Society of Respiratory Science and the Thoracic Society of Australia and New Zealand Annual Scientific Meeting , ANZSRS/TSANZ 2018. Adelaide, SA Australia. 23(Supplement 1) (pp 117), 2018. Date of Publication: March 2018.

[Conference Abstract]

AN: 622091434

Introduction/Aim: Whether participation in comprehensive pulmonary rehabilitation programs (CPRP) changes habitual time use in people with COPD is equivocal. This study aimed to explore whether time use patterns changed in the twelve months after CPRP participation.

Method(s): Using a non-randomised clinical trial design, adults with clinically stable moderate to severe COPD were recruited into an eightweek CPRP or Usual Care cohort. Baseline demographics, objective measures of function, self-reported COPD-related impairment and use-oftime recall interviews (24-hour profiles of habitual activity) were assessed at baseline and one, six and 12 months post CPRP. Activities sharing similar context were grouped into one of eight domains (chores, household administration, quiet time, screen time, self-care, sleep, socio-cultural, sports/exercise), which were then used to create compositional profiles representing an average day. Between compositions and between compositional components (at group and time point level) comparisons were conducted using ANOVA models and isometric log ratio differences respectively.

Result(s): 89 people (mean age 69.7 +/- 9.7 yrs. FEV1 51% +/- 9) were recruited into CPRP (n=49) or Usual Care (n=40). With the exception of FEV1 %pred (CPRP 47% +/- 19% vs. Usual Care 55% +/- 16% [p=0.032]), baseline demographics were similar. Both cohorts exhibited similar time use patterns where there were no statistically significant differences in time use patterns evident between cohorts or time points.

Conclusion(s): This study found CPRP did not significantly influence patterns of habitual time use in people with COPD. Given the observed similarities in use-of-time profiles between cohorts, this data suggests changing habitual patterns of time use in people with COPD requires more than enrolment, and participation in, an eight week CPRP.

Status

CONFERENCE ABSTRACT

Institution

(Hunt, Williams, Olds, Dumuid) University of South Australia, Adelaide, Australia

Publisher

Blackwell Publishing

Year of Publication

2018

Link to the Ovid Full Text or citation:

[Click here for full text options](https://ovidsp.ovid.com/ovidweb.cgi?T=JS&CSC=Y&NEWS=N&PAGE=fulltext&D=emed19&AN=622091434)

Link to the External Link Resolver:

[LibKey NHS](https://libkey.io/libraries/2789/openurl?genre=article&sid=OVID:emed19&genre=article&id=pmid:&id=doi:10.1111%2Fresp.13268&issn=1440-1843&volume=23&issue=Supplement+1&spage=117&pages=117&date=2018&title=Respirology&atitle=Participation+in+pulmonary+rehabilitation+did+not+significantly+alter+time+use+patterns+in+people+with+COPD&aulast=Hunt)

101.

Pulmonary rehabilitation does not improve objective measures of sleep quality in people with chronic obstructive pulmonary disease. (repeat)

Cox N., Pepin V., Burge A., Mahal A., Hill C., Lee A., Moore R., Nicholson C., O'halloran P., Lahham A., Gillies R., McDonald C., Holland A.

Embase

Respirology. Conference: Australia and New Zealand Society of Respiratory Science and the Thoracic Society of Australia and New Zealand Annual Scientific Meeting , ANZSRS/TSANZ 2018. Adelaide, SA Australia. 23(Supplement 1) (pp 89), 2018. Date of Publication: March 2018.

[Conference Abstract]

AN: 622091421

Introduction/Aim: More than 50% of people with chronic obstructive pulmonary disease (COPD) report poor sleep quality which has been associated with increased morbidity and mortality. There are varying reports of the effect of pulmonary rehabilitation (PR) on self-reported sleep quality. We aimed to assess the effect of PR on objectively measured sleep quality in people with COPD.

Method(s): This is a secondary analysis of data collected as part of an RCT comparing home-based to centre-based PR for COPD. Sleep quality was assessed objectively using the SenseWear Armband (SWA, Bodymedia USA), worn for 7 days before and after an 8-week PR programme. Sleep characteristics were derived from accelerometer positional data and registration of sleep state by the SWA, determined from energy expenditure.

Result(s): 33 participants (17 male; mean+/-SD age 68+/-11 years, FEV1 56+/-21 %predicted) had paired pre and post PR sleep data. Twenty participants received centre-based PR and n=13 were allocated to homebased PR. Pre-PR median [interquartile range] sleep onset latency (SOL) was 25 [9, 41] minutes, total sleep time (TST) 390 [339, 421] minutes and wake after sleep onset (WASO) 74 [43, 106] minutes. Sleep efficiency (SE) was low (75% [64, 84]). No significant differences were seen in any sleep parameter (SOL, TST, WASO, SE) after PR (all p>0.10), between intervention groups (all p>0.20), or within PR groups (all p=0.13). There was no association between sleep parameters and measures of quality of life or function before or after PR. Attendance at a greater proportion of PR sessions was moderately associated with more TST at end rehabilitation (rs=0.5, p=0.009).

Conclusion(s): Sleep quality, measured objectively using actigraphy, did not improve after an 8-week PR programme in individuals with COPD. Whether ongoing participation in regular exercise training beyond the duration of PR may influence sleep quality is yet to be determined.

Status

CONFERENCE ABSTRACT

Institution

(Cox, Burge, Lee, Nicholson, O'halloran, Lahham, Holland) La Trobe University, Melbourne, Australia (Cox, Hill, Lee, Lahham, Gillies, McDonald, Holland) Institute for Breathing and Sleep, Melbourne, Australia

(Pepin) Concordia University, Hopital du Sacre-Coeur de Montreal, Montreal, Canada

(Burge, Lee, Nicholson, Holland) Alfred Health, Melbourne, Australia

(Mahal, McDonald) University of Melbourne, Melbourne, Australia

(Hill, Moore, Gillies, McDonald) Austin Health, Melbourne, Australia

Publisher

Blackwell Publishing

Year of Publication

2018

Link to the Ovid Full Text or citation:

[Click here for full text options](https://ovidsp.ovid.com/ovidweb.cgi?T=JS&CSC=Y&NEWS=N&PAGE=fulltext&D=emed19&AN=622091421)

Link to the External Link Resolver:

[LibKey NHS](https://libkey.io/libraries/2789/openurl?genre=article&sid=OVID:emed19&genre=article&id=pmid:&id=doi:10.1111%2Fresp.13267&issn=1440-1843&volume=23&issue=Supplement+1&spage=89&pages=89&date=2018&title=Respirology&atitle=Pulmonary+rehabilitation+does+not+improve+objective+measures+of+sleep+quality+in+people+with+chronic+obstructive+pulmonary+disease&aulast=Cox)

102.

Risk stratification before thoracic surgery, perioperative pulmonary rehabilitation. A mellkassebeszeti muteti teherbiro kepesseg megitelese, perioperativ legzesrehabilitacio <A mellkassebeszeti muteti teherbiro kepesseg megitelese, perioperativ legzesrehabilitacio.>

Vagvolgyi A., Rozgonyi Z., Vadasz P., Varga J.T.

Embase

Orvosi Hetilap. 158(50) (pp 1989-1997), 2017. Date of Publication: December 2017.

[Article]

AN: 620015852

Introduction: Besides the oncology and operative surgical technics, functional aspects influence the operability of lung cancer. Preoperative risk stratification, evaluation of postoperative complications needs to be considered.

Aim(s): To review international literature and experiences of our institute.

Method(s): We focused the literature of risk stratification of thoracic surgery. Lung function, lung mechanics, chest kinematics, exercise physiology were considered. Effectiveness of pulmonary rehabilitation for cardiovascular system, lung mechanics, muscles, exercise capacity and quality of life were evaluated. Laboratory parameters, comorbidities, obesity, cachexia, smoking cessation were considered.

Result(s): Elevated blood sugar, kidney function, reduced albumin level increased the risk. COPD, sleep apnoea, heart failure, obesity and cachexia influences the outcome. Smoking cessation may reduce postoperative complications. Controlled breathing technics, chest wall mobilization, training have favourable effects. Psychosocial support and dietetics are important.

Conclusion(s): Risk stratification is supported by laboratory parameters, lung function, oxygen uptake and comorbidities. Pulmonary rehabilitation can improve functionality and quality of life.

Status

Embase

Institution

(Vagvolgyi, Vadasz) Orszagos Koranyi Pulmonologiai Intezet, Mellkassebeszeti Osztaly, Budapest, Hungary (Rozgonyi) Orszagos Koranyi Pulmonologiai Intezet, Aneszteziologiai Es Intenziv Terapias Osztaly, Budapest, Hungary

(Varga) Orszagos Koranyi Pulmonologiai Intezet, Legzesrehabilitacios Osztaly, Piheno ut 1., Budapest 1121, Hungary

(Vadasz) Semmelweis Egyetem, Altalanos Orvostudomanyi Kar, Mellkassebeszeti Tanszeki Csoport, Budapest, Hungary

Publisher

Akademiai Kiado ZRt.

Year of Publication

2017

Link to the Ovid Full Text or citation:

[Click here for full text options](https://ovidsp.ovid.com/ovidweb.cgi?T=JS&CSC=Y&NEWS=N&PAGE=fulltext&D=emed18&AN=620015852)

Link to the External Link Resolver:

[LibKey NHS](https://libkey.io/libraries/2789/openurl?genre=article&sid=OVID:emed18&genre=article&id=pmid:&id=doi:10.1556%2F650.2017.30862&issn=0030-6002&volume=158&issue=50&spage=1989&pages=1989-1997&date=2017&title=Orvosi+Hetilap&atitle=A+mellkassebeszeti+muteti+teherbiro+kepesseg+megitelese%2C+perioperativ+legzesrehabilitacio&aulast=Vagvolgyi)

103.

Obesity in COPD: to treat or not to treat?.

McDonald V.M., Wood L.G., Holland A.E., Gibson P.G.

Embase

Expert Review of Respiratory Medicine. 11(2) (pp 81-83), 2017. Date of Publication: 01 Feb 2017.

[Editorial]

AN: 613689542

PMID

27910701 [<https://www.ncbi.nlm.nih.gov/pubmed/?term=27910701>]

Status

Embase

Institution

(McDonald, Gibson) National Health and Medical Research Council Centre of Excellence in Severe Asthma, Newcastle, Australia (McDonald, Wood, Gibson) Priority Research Centre for Healthy Lungs, The University of Newcastle, Newcastle, Australia

(McDonald, Gibson) Department of Respiratory and Sleep Medicine, John Hunter Hospital, Newcastle, Australia

(Holland) Discipline of Physiotherapy, La Trobe University, Melbourne, Australia

(Holland) Department of Physiotherapy, Alfred Health, Melbourne, Australia

(Holland) Institute for Breathing and Sleep, Melbourne, Australia

Publisher

Taylor and Francis Ltd.

Year of Publication

2017

Link to the Ovid Full Text or citation:

[Click here for full text options](https://ovidsp.ovid.com/ovidweb.cgi?T=JS&CSC=Y&NEWS=N&PAGE=fulltext&D=emed18&AN=613689542)

Link to the External Link Resolver:

[LibKey NHS](https://libkey.io/libraries/2789/openurl?genre=article&sid=OVID:emed18&genre=article&id=pmid:27910701&id=doi:10.1080%2F17476348.2017.1267570&issn=1747-6348&volume=11&issue=2&spage=81&pages=81-83&date=2017&title=Expert+Review+of+Respiratory+Medicine&atitle=Obesity+in+COPD%3A+to+treat+or+not+to+treat%3F&aulast=McDonald)

104.

Obstructive and environmental respiratory diseases: Updates and new facets.

Varkey B., Joshi M.

Embase

Current Opinion in Pulmonary Medicine. 23(2) (pp 109-110), 2017. Date of Publication: 01 Mar 2017.

[Editorial]

AN: 613867932

PMID

28009645 [<https://www.ncbi.nlm.nih.gov/pubmed/?term=28009645>]

Status

Embase

Institution

(Varkey) Department of Medicine (Pulmonary and Critical Care), Medical College of Wisconsin, 120 Lakota Pass, Milwaukee, WI 78738, United States (Joshi) Pulmonary and Critical Care Division, University of Arkansas for Medical Sciences and Central, Arkansas Veterans Healthcare System, Little Rock, AR, United States

Publisher

Lippincott Williams and Wilkins (E-mail: kathiest.clai@apta.org)

Year of Publication

2017

Link to the Ovid Full Text or citation:

[Click here for full text options](https://ovidsp.ovid.com/ovidweb.cgi?T=JS&CSC=Y&NEWS=N&PAGE=fulltext&D=emed18&AN=613867932)

Link to the External Link Resolver:

[LibKey NHS](https://libkey.io/libraries/2789/openurl?genre=article&sid=OVID:emed18&genre=article&id=pmid:28009645&id=doi:10.1097%2FMCP.0000000000000359&issn=1070-5287&volume=23&issue=2&spage=109&pages=109-110&date=2017&title=Current+Opinion+in+Pulmonary+Medicine&atitle=Obstructive+and+environmental+respiratory+diseases%3A+Updates+and+new+facets&aulast=Varkey)

105.

A Comparison of Pain, Fatigue, Dyspnea and their Impact on Quality of Life in Pulmonary Rehabilitation Participants with Chronic Obstructive Pulmonary Disease.

Chen Y.-W., Camp P.G., Coxson H.O., Road J.D., Guenette J.A., Hunt M.A., Reid W.D.

Embase

COPD: Journal of Chronic Obstructive Pulmonary Disease. 15(1) (pp 65-72), 2018. Date of Publication: 02 Jan 2018.

[Article]

AN: 619670564

In addition to dyspnea and fatigue, pain is a prevalent symptom in chronic obstructive pulmonary disease (COPD). Understanding the relative prevalence, magnitude, and interference with aspects of daily living of these symptoms can improve COPD management. Therefore, the purposes of this study were to: (1) compare the prevalence and magnitude of dyspnea, fatigue, and pain and how each limits aspects of daily living; (2) determine the association between pain and the other two symptoms; and (3) assess the impact of these symptoms on quality of life in COPD. Participants were recruited from pulmonary rehabilitation programs. Pain, dyspnea, and fatigue were measured using the Brief Pain Inventory (BPI), Brief Fatigue Inventory (BFI), and Dyspnea Inventory (DI), respectively. Quality of life was measured using the Clinical COPD Questionnaire (CCQ). The prevalence of dyspnea, fatigue, and pain were 93%, 77%, and 74%, respectively. Individuals with COPD reported similar severity scores of the three symptoms. Dyspnea interfered with general activity more than pain (F1.7,79.9 = 3.1, p < 0.05), whilst pain interfered with mood (F1.8, 82.7 = 3.6, p < 0.05) and sleep (F1,46 = 7.4, p < 0.01) more than dyspnea and fatigue. These three symptoms were moderately-to-highly correlated with each other (rho = 0.49-0.78, p < 0.01) and all individually impacted quality of life. In summary, pain is a common symptom in addition to dyspnea and fatigue in COPD; all three interfere similarly among aspects of daily living with some exceptions. Accordingly, management of COPD should include a multifaceted approach that addresses pain as well as dyspnea and fatigue.

Copyright © 2018 Taylor & Francis Group, LLC.

PMID

29227712 [<https://www.ncbi.nlm.nih.gov/pubmed/?term=29227712>]

Status

Embase

Institution

(Chen, Hunt) Department of Physical Therapy, University of British Columbia, Vancouver, BC, Canada (Camp, Guenette) Department of Physical Therapy, and Centre for Heart Lung Innovation, University of British Columbia, Vancouver, BC, Canada

(Coxson) Department of Radiology, and Centre for Heart Lung Innovation, University of British Columbia, Vancouver, BC, Canada

(Road) Division of Respiratory Medicine, Department of Medicine, University of British Columbia, Vancouver, BC, Canada

(Reid) Department of Physical Therapy, University of Toronto, Toronto, ON, Canada

Publisher

Taylor and Francis Ltd (E-mail: healthcare.enquiries@informa.com)

Year of Publication

2018

Link to the Ovid Full Text or citation:

[Click here for full text options](https://ovidsp.ovid.com/ovidweb.cgi?T=JS&CSC=Y&NEWS=N&PAGE=fulltext&D=emed18&AN=619670564)

Link to the External Link Resolver:

[LibKey NHS](https://libkey.io/libraries/2789/openurl?genre=article&sid=OVID:emed18&genre=article&id=pmid:29227712&id=doi:10.1080%2F15412555.2017.1401990&issn=1541-2555&volume=15&issue=1&spage=65&pages=65-72&date=2018&title=COPD%3A+Journal+of+Chronic+Obstructive+Pulmonary+Disease&atitle=A+Comparison+of+Pain%2C+Fatigue%2C+Dyspnea+and+their+Impact+on+Quality+of+Life+in+Pulmonary+Rehabilitation+Participants+with+Chronic+Obstructive+Pulmonary+Disease&aulast=Chen)

106.

Revolving door respiratory patients: A rehabilitative perspective.

Polastri M., Pisani L., Dell'Amore A., Nava S.

Embase

Monaldi Archives for Chest Disease. 87(3) (pp 94-95), 2017. Date of Publication: 2017.

[Review]

AN: 622996579

Rehabilitation is an integral component of care for patients affected by either acute or chronic pulmonary diseases. The key elements of rehabilitation treatment for critical respiratory patients are as follows: weaning from mechanical ventilation, respiratory therapy, physical reconditioning, and occupational therapy. It should be noted that patients affected by pulmonary diseases are prone to hospital re-admission due to frequent exacerbations, especially in cases with more severe stages of chronic obstructive pulmonary disease. A periodical worsening of clinical conditions is common in asthma, acute respiratory distress syndrome survivors, obstructive sleep apnea syndrome, and pulmonary fibrosis, as well as in patients with severe neuromuscular diseases. These patients are often identified as "revolving door patients". Pulmonary patients are typically forced to maintain bed rest, or at least spend most of their waking hours dealing with mobility limitations, due to various pathological conditions including dyspnea, fatigue, and poor tolerance of movements. Alterations in mood are common in pulmonary patients who experience a decreased quality of life and limited social interactions. These negative emotional and cognitive aspects can be a major limitation to the provision of care, because to enhance and facilitate a degree of autonomy, the patient must be cooperative and pro-active.

Copyright © 2017 M. Polastri et al.

Status

Embase

Institution

(Polastri) Medical Department of Continuity of Care and Disability, Physical Medicine and Rehabilitation, University Hospital St. Orsola-Malpighi, Via G. Massarenti 9, Bologna 40138, Italy (Pisani, Nava) Department of Clinical, Integrated and Experimental Medicine (DIMES), Respiratory and Critical Care Unit, University Hospital St. Orsola-Malpighi, Bologna, Italy

(Dell'Amore) Department of Cardiac-Thoracic and Vascular Diseases, Unit of Thoracic Surgery, University Hospital St. Orsola-Malpighi, Bologna, Italy

Publisher

PAGEPress Publications (E-mail: emanuela.fusinato@pagepress.org)

Year of Publication

2017

Link to the Ovid Full Text or citation:

[Click here for full text options](https://ovidsp.ovid.com/ovidweb.cgi?T=JS&CSC=Y&NEWS=N&PAGE=fulltext&D=emed18&AN=622996579)

Link to the External Link Resolver:

[LibKey NHS](https://libkey.io/libraries/2789/openurl?genre=article&sid=OVID:emed18&genre=article&id=pmid:&id=doi:10.4081%2Fmonaldi.2017.857&issn=1122-0643&volume=87&issue=3&spage=94&pages=94-95&date=2017&title=Monaldi+Archives+for+Chest+Disease&atitle=Revolving+door+respiratory+patients%3A+A+rehabilitative+perspective&aulast=Polastri)

107.

Differential Pulmonary Rehabilitation Outcomes in Patients with and Without COPD: ROLE of GENDER.

Nguyen L.-P., Beck E., Cayetano K., Li C.-S., Hardin K.

Embase

Journal of Cardiopulmonary Rehabilitation and Prevention. 37(5) (pp 350-355), 2017. Date of Publication: 01 Sep 2017.

[Article]

AN: 617464116

Purpose: Individuals with all forms of pulmonary disease are referred for pulmonary rehabilitation. This study examines pulmonary rehabilitation outcomes between individuals with chronic obstructive lung disease (COPD) and non-COPD disease and the impact of gender.

Method(s): This is a retrospective study at a tertiary center. The primary endpoint was the difference in 6-min walk test distance. Secondary measurements included treadmill and NuStep minutes; biceps curls and front arm raises load; quality of life measured by the St George's Respiratory Questionnaire; and University of California San Diego-Shortness of Breath Questionnaire (UCSD-SOBQ) scores.

Result(s): Eighty patients were included: 38 men (23 COPD, 15 non-COPD) and 42 women (31 COPD, 11 non-COPD). There was a statistically significant improvement in 6-min walk test distances pre- to post-pulmonary rehabilitation for all participants, P =.0003. Although both the COPD and non-COPD groups demonstrated overall improvement (P <.0004 and P =.02, respectively), subgroup analysis showed no statistically significant change in the non-COPD group when divided by gender. There was a significant statistical improvement in lower and upper extremity strength in all participants. Only women with COPD showed a statistically significant improvement with respect to overall quality of life as measured by St. George's Respiratory Questionnaire (P =.01). Women showed significant improvement in their depression score, as well as a trend toward improvement in the University of California San Diego-Shortness of Breath Questionnaire, while only men with COPD showed any improvement in their sleep quality measured by the Pittsburgh Sleep Quality Index.

Conclusion(s): Pulmonary rehabilitation results in different but improved outcomes regardless of gender or disease state.

Copyright © 2017 Wolters Kluwer Health, Inc. All rights reserved.

PMID

28727669 [<https://www.ncbi.nlm.nih.gov/pubmed/?term=28727669>]

Status

Embase

Institution

(Nguyen, Beck, Cayetano, Hardin) Departments of Internal Medicine, Division of Pulmonary, Critical Care Sleep Medicine, 4150 V St, PSSB Ste 3100, Sacramento, CA 95817, United States (Nguyen, Beck, Cayetano, Li, Hardin) Biostatistics University of California, Davis and VA Northern California Health Care System, Sacramento VA Medical Center, Mather, United States

Publisher

Lippincott Williams and Wilkins (E-mail: kathiest.clai@apta.org)

Year of Publication

2017

Link to the Ovid Full Text or citation:

[Click here for full text options](https://ovidsp.ovid.com/ovidweb.cgi?T=JS&CSC=Y&NEWS=N&PAGE=fulltext&D=emed18&AN=617464116)

Link to the External Link Resolver:

[LibKey NHS](https://libkey.io/libraries/2789/openurl?genre=article&sid=OVID:emed18&genre=article&id=pmid:28727669&id=doi:10.1097%2FHCR.0000000000000275&issn=1932-7501&volume=37&issue=5&spage=350&pages=350-355&date=2017&title=Journal+of+Cardiopulmonary+Rehabilitation+and+Prevention&atitle=Differential+Pulmonary+Rehabilitation+Outcomes+in+Patients+with+and+Without+COPD%3A+ROLE+of+GENDER&aulast=Nguyen)

108.

Treatment of idiopathic pulmonary fibrosis in Australia and New Zealand: A position statement from the Thoracic Society of Australia and New Zealand and the Lung Foundation Australia.

Jo H.E., Troy L.K., Keir G., Chambers D.C., Holland A., Goh N., Wilsher M., de Boer S., Moodley Y., Grainge C., Whitford H., Chapman S., Reynolds P.N., Glaspole I., Beatson D., Jones L., Hopkins P., Corte T.J.

Embase

Respirology. 22(7) (pp 1436-1458), 2017. Date of Publication: October 2017.

[Article]

AN: 618025486

Idiopathic pulmonary fibrosis (IPF) is a fibrosing interstitial lung disease (ILD) of unknown aetiology with a median survival of only 2-5 years. It is characterized by progressive dyspnoea and worsening lung function, ultimately resulting in death. Until recently, there were no effective therapies for IPF; however, with the publication of two landmark clinical trials in 2014, the anti-fibrotic therapies, nintedanib and pirfenidone, have gained widespread approval. This position paper aims to highlight the current evidence for the treatment of IPF, with particular application to the Australian and New Zealand population. We also consider areas in which evidence is currently lacking, especially with regard to the broader IPF severity spectrum and treatment of co-morbid conditions. The utility of non-pharmacological therapies including pulmonary rehabilitation, oxygen as well as symptom management thought to be important in the holistic care of IPF patients are also discussed.

Copyright © 2017 The Authors. Respirology published by John Wiley & Sons Australia, Ltd on behalf of Asian Pacific Society of Respirology

PMID

28845557 [<https://www.ncbi.nlm.nih.gov/pubmed/?term=28845557>]

Status

Embase

Author NameID

Jo, Helen E.; ORCID: <https://orcid.org/0000-0003-1183-2729> Glaspole, Ian; ORCID: <https://orcid.org/0000-0002-5118-2890>

Institution

(Jo, Troy, Corte) Department of Respiratory Medicine, Royal Prince Alfred Hospital, Sydney, NSW, Australia (Jo, Troy, Corte) Faculty of Medicine, University of Sydney, Sydney, NSW, Australia

(Keir) Department of Respiratory Medicine, Princess Alexandra Hospital, Brisbane, QLD, Australia

(Chambers, Goh, Hopkins) Department of Respiratory Medicine, The Prince Charles Hospital, Brisbane, QLD, Australia

(Holland) Department of Physiotherapy, The Alfred Hospital, Melbourne, VIC, Australia

(Whitford, Glaspole) Department of Respiratory Medicine, The Alfred Hospital, Melbourne, VIC, Australia

(Goh) Department of Respiratory Medicine, Austin Hospital, Melbourne, VIC, Australia

(Wilsher, de Boer) Department of Respiratory Medicine, Auckland District Health Board, Auckland, New Zealand

(Moodley) Department of Respiratory Medicine, Fiona Stanley Hospital, Perth, WA, Australia

(Grainge, Jones) Department of Respiratory Medicine, John Hunter Hospital, Newcastle, NSW, Australia

(Chapman, Reynolds) Department of Respiratory Medicine, Royal Adelaide Hospital, Adelaide, SA, Australia

(Beatson) Patient Advocate, Auckland, New Zealand

Publisher

Blackwell Publishing

Clinical Trial Number

2014-004058-32/EudraCT <https://clinicaltrials.gov/show/NCT00189176>

<https://clinicaltrials.gov/show/NCT00203697>

<https://clinicaltrials.gov/show/NCT00262405>

<https://clinicaltrials.gov/show/NCT00463983>

<https://clinicaltrials.gov/show/NCT00786201>

<https://clinicaltrials.gov/show/NCT01254409>

<https://clinicaltrials.gov/show/NCT01371305>

<https://clinicaltrials.gov/show/NCT01462006>

<https://clinicaltrials.gov/show/NCT01619085>

<https://clinicaltrials.gov/show/NCT01766817>

<https://clinicaltrials.gov/show/NCT01872689>

<https://clinicaltrials.gov/show/NCT01890265>

<https://clinicaltrials.gov/show/NCT01919827>

<https://clinicaltrials.gov/show/NCT01982968>

<https://clinicaltrials.gov/show/NCT02036280>

<https://clinicaltrials.gov/show/NCT02085018>

<https://clinicaltrials.gov/show/NCT02173145>

<https://clinicaltrials.gov/show/NCT02257177>

<https://clinicaltrials.gov/show/NCT02286063>

<https://clinicaltrials.gov/show/NCT02315586>

<https://clinicaltrials.gov/show/NCT02345070>

<https://clinicaltrials.gov/show/NCT02397005>

<https://clinicaltrials.gov/show/NCT02538536>

<https://clinicaltrials.gov/show/NCT02612051>

<https://clinicaltrials.gov/show/NCT02648048>

<https://clinicaltrials.gov/show/NCT02688647>

<https://clinicaltrials.gov/show/NCT02738801>

<https://clinicaltrials.gov/show/NCT02759120>

<https://clinicaltrials.gov/show/NCT02802345>

<https://clinicaltrials.gov/show/NCT02874989>

Year of Publication

2017

Link to the Ovid Full Text or citation:

[Click here for full text options](https://ovidsp.ovid.com/ovidweb.cgi?T=JS&CSC=Y&NEWS=N&PAGE=fulltext&D=emed18&AN=618025486)

Link to the External Link Resolver:

[LibKey NHS](https://libkey.io/libraries/2789/openurl?genre=article&sid=OVID:emed18&genre=article&id=pmid:28845557&id=doi:10.1111%2Fresp.13146&issn=1323-7799&volume=22&issue=7&spage=1436&pages=1436-1458&date=2017&title=Respirology&atitle=Treatment+of+idiopathic+pulmonary+fibrosis+in+Australia+and+New+Zealand%3A+A+position+statement+from+the+Thoracic+Society+of+Australia+and+New+Zealand+and+the+Lung+Foundation+Australia&aulast=Jo)

109.

Global Strategy for the Diagnosis, Management, and Prevention of Chronic Obstructive Lung Disease 2017 Report: GOLD Executive Summary. Informe 2017 de la Iniciativa Global para el Diagnostico, Tratamiento y Prevencion de la Enfermedad Pulmonar Obstructiva Cronica: Resumen Ejecutivo de GOLD <Informe 2017 de la Iniciativa Global para el Diagnostico, Tratamiento y Prevencion de la Enfermedad Pulmonar Obstructiva Cronica: Resumen Ejecutivo de GOLD.>

Vogelmeier C.F., Criner G.J., Martinez F.J., Anzueto A., Barnes P.J., Bourbeau J., Celli B.R., Chen R., Decramer M., Fabbri L.M., Frith P., Halpin D.M.G., Varela M.V.L., Nishimura M., Roche N., Rodriguez-Roisin R., Sin D.D., Singh D., Stockley R., Vestbo J., Wedzicha J.A., Agusti A.

Embase

Archivos de Bronconeumologia. 53(3) (pp 128-149), 2017. Date of Publication: March 2017.

[Article]

AN: 617547111

This Executive Summary of the Global Strategy for the Diagnosis, Management, and Prevention of COPD (GOLD) 2017 Report focuses primarily on the revised and novel parts of the document. The most significant changes include: 1) the assessment of COPD has been refined to separate the spirometric assessment from symptom evaluation. ABCD groups are now proposed to be derived exclusively from patient symptoms and their history of exacerbations; 2) for each of the groups A to D, escalation strategies for pharmacological treatments are proposed; 3) the concept of de-escalation of therapy is introduced in the treatment assessment scheme; 4) nonpharmacologic therapies are comprehensively presented and; 5) the importance of comorbid conditions in managing COPD is reviewed.

Copyright © 2017 SEPAR

PMID

28274597 [<https://www.ncbi.nlm.nih.gov/pubmed/?term=28274597>]

Status

Embase

Institution

(Vogelmeier) University of Marburg, Member of the German Center for Lung Research (DZL), Marburg, Germany (Criner) Lewis Katz School of Medicine at Temple University, Philadelphia, Pennsylvania, United States

(Martinez) New York-Presbyterian Hospital, Weill Cornell Medical Center, New York, New York, United States

(Anzueto) University of Texas Health Science Center and South Texas Veterans Health Care System, San Antonio, Texas, United States

(Barnes) National Heart and Lung Institute, Imperial College, London, United Kingdom

(Bourbeau) McGill University Health Centre, McGill University, Montreal, Canada

(Celli) Brigham and Women's Hospital Boston, Massachusetts, United States

(Chen) State Key Lab for Respiratory Disease, Guangzhou Institute of Respiratory Disease, First Affiliated Hospital of Guangzhou Medical University, Guangzhou, China

(Decramer) University of Leuven, Leuven, Belgium

(Fabbri) University of Modena & Reggio Emilia, Modena, Italy

(Frith) Flinders University Faculty of Medicine, Bedford Park, South Australia, Australia

(Halpin) Royal Devon & Exeter Hospital, Exeter, United Kingdom

(Varela) Universidad de la Republica, Hospital Maciel, Montevideo, Uruguay

(Nishimura) Hokkaido University School of Medicine Sapporo, Japan

(Roche) Hopital Cochin (APHP), University Paris Descartes, Paris, France

(Rodriguez-Roisin) Thorax Institute, Hospital Clinic Universitat de Barcelona, Barcelona, Spain

(Sin) St. Paul's Hospital, University of British Columbia, Vancouver, Canada

(Singh, Vestbo) University of Manchester, Manchester, United Kingdom

(Stockley) University Hospital, Birmingham, United Kingdom

(Wedzicha) Imperial College London, London, United Kingdom

(Agusti) Hospital Clinic, Universitat de Barcelona, Ciberes, Barcelona, Spain

Publisher

Elsevier Doyma

Year of Publication

2017

Link to the Ovid Full Text or citation:

[Click here for full text options](https://ovidsp.ovid.com/ovidweb.cgi?T=JS&CSC=Y&NEWS=N&PAGE=fulltext&D=emed18&AN=617547111)

Link to the External Link Resolver:

[LibKey NHS](https://libkey.io/libraries/2789/openurl?genre=article&sid=OVID:emed18&genre=article&id=pmid:28274597&id=doi:10.1016%2Fj.arbr.2017.02.001&issn=0300-2896&volume=53&issue=3&spage=128&pages=128-149&date=2017&title=Archivos+de+Bronconeumologia&atitle=Informe+2017+de+la+Iniciativa+Global+para+el+Diagnostico%2C+Tratamiento+y+Prevencion+de+la+Enfermedad+Pulmonar+Obstructiva+Cronica%3A+Resumen+Ejecutivo+de+GOLD&aulast=Vogelmeier)

110.

Long-Term Oxygen Therapy in COPD Patients Who Do Not Meet the Actual Recommendations.

Ergan B., Nava S.

Embase

COPD: Journal of Chronic Obstructive Pulmonary Disease. 14(3) (pp 351-366), 2017. Date of Publication: 04 May 2017.

[Review]

AN: 616496877

Chronic respiratory failure due to chronic obstructive pulmonary disease (COPD) is an increasing problem worldwide. Many patients with severe COPD develop hypoxemic respiratory failure during the natural progression of disease. Long-term oxygen therapy (LTOT) is a well-established supportive treatment for COPD and has been shown to improve survival in patients who develop chronic hypoxemic respiratory failure. The degree of hypoxemia is severe when partial pressure of oxygen in arterial blood (PaO2) is <=55 mmHg and moderate if PaO2 is between 56 and 69 mmHg. Although current guidelines consider LTOT only in patients with severe resting hypoxemia, many COPD patients with moderate to severe disease experience moderate hypoxemia at rest or during special circumstances, such as while sleeping or exercising. The efficacy of LTOT in these patients who do not meet the actual recommendations is still a matter of debate, and extensive research is still ongoing to understand the possible benefits of LTOT for survival and/or functional outcomes such as the sensation of dyspnea, exacerbation frequency, hospitalizations, exercise capacity, and quality of life. Despite its frequent use, the administration of "palliative" oxygen does not seem to improve dyspnea except for delivery with high-flow humidified oxygen. This narrative review will focus on current evidence for the effects of LTOT in the presence of moderate hypoxemia at rest, during sleep, or during exercise in COPD.

Copyright © 2017 Taylor & Francis Group, LLC.

PMID

28506089 [<https://www.ncbi.nlm.nih.gov/pubmed/?term=28506089>]

Status

Embase

Institution

(Ergan) Department of Pulmonary and Critical Care, Faculty of Medicine, Dokuz Eylul University, Izmir, Turkey (Nava) Department of Clinical, Integrated and Experimental Medicine (DIMES), Respiratory and Critical Care Unit, S. Orsola-Malpighi Hospital, Alma Mater University, Bologna, Italy

Publisher

Taylor and Francis Ltd (E-mail: healthcare.enquiries@informa.com)

Year of Publication

2017

Link to the Ovid Full Text or citation:

[Click here for full text options](https://ovidsp.ovid.com/ovidweb.cgi?T=JS&CSC=Y&NEWS=N&PAGE=fulltext&D=emed18&AN=616496877)

Link to the External Link Resolver:

[LibKey NHS](https://libkey.io/libraries/2789/openurl?genre=article&sid=OVID:emed18&genre=article&id=pmid:28506089&id=doi:10.1080%2F15412555.2017.1319918&issn=1541-2555&volume=14&issue=3&spage=351&pages=351-366&date=2017&title=COPD%3A+Journal+of+Chronic+Obstructive+Pulmonary+Disease&atitle=Long-Term+Oxygen+Therapy+in+COPD+Patients+Who+Do+Not+Meet+the+Actual+Recommendations&aulast=Ergan)

111.

French practical guidelines for the diagnosis and management of idiopathic pulmonary fibrosis - 2017 update. Short-length version. Recommandations pratiques pour le diagnostic et la prise en charge de la fibrose pulmonaire idiopathique - Actualisation 2017. Version courte <Recommandations pratiques pour le diagnostic et la prise en charge de la fibrose pulmonaire idiopathique - Actualisation 2017. Version courte.>

Cottin V., Crestani B., Cadranel J., Cordier J.-F., Marchand-Adam S., Prevot G., Wallaert B., Bergot E., Camus P., Dalphin J.-C., Dromer C., Gomez E., Israel-Biet D., Jouneau S., Kessler R., Marquette C.-H., Reynaud-Gaubert M., Aguilaniu B., Bonnet D., Carre P., Danel C., Faivre J.-B., Ferretti G., Just N., Lebargy F., Philippe B., Terrioux P., Thivolet-Bejui F., Trumbic B., Valeyre D.

Embase

Revue des Maladies Respiratoires. 34(8) (pp 852-899), 2017. Date of Publication: October 2017.

[Article]

AN: 618363209

PMID

28939154 [<https://www.ncbi.nlm.nih.gov/pubmed/?term=28939154>]

Status

Embase

Institution

(Cottin, Cordier) Centre national de reference des maladies pulmonaires rares, pneumologie, hopital Louis-Pradel, hospices civils de Lyon, universite Claude-Bernard-Lyon 1, Lyon, France (Crestani) Service de pneumologie A, centre de competences pour les maladies pulmonaires rares, CHU Bichat, universite Paris Diderot, Paris, France

(Cadranel) Service de pneumologie et oncologie thoracique, centre de competences pour les maladies pulmonaires rares, hopital Tenon, universite Pierre-et-Marie-Curie, Paris 6, GH-HUEP, Assistance publique-Hopitaux de Paris, Paris, France

(Marchand-Adam) Service de pneumologie, centre de competences pour les maladies pulmonaires rares, CHU de Tours, Tours, France

(Prevot) Service de pneumologie, centre de competences pour les maladies pulmonaires rares, CHU Larrey, Toulouse, France

(Wallaert) Service de pneumologie et immuno-allergologie, centre de competences pour les maladies pulmonaires rares, hopital Calmette, CHRU de Lille, Lille, France

(Bergot) Service de pneumologie et oncologie thoracique, centre de competences pour les maladies pulmonaires rares, CHU de Caen, Caen, France

(Camus) Service de pneumologie et oncologie thoracique, centre de competences pour les maladies pulmonaires rares, CHU Dijon-Bourgogne, Dijon, France

(Dalphin) Service de pneumologie, allergologie et oncologie thoracique, centre de competences pour les maladies pulmonaires rares, hopital Jean-Minjoz, CHRU de Besancon, Besancon, France

(Dromer) Service de pneumologie, centre de competences pour les maladies pulmonaires rares, hopital Haut-Leveque, CHU de Bordeaux, Bordeaux, France

(Gomez) Departement de pneumologie, centre de competences pour les maladies pulmonaires rares, CHU de Nancy, Vandoeuvre-les-Nancy, France

(Israel-Biet) Service de pneumologie, centre de competences pour les maladies pulmonaires rares, hopital europeen Georges-Pompidou, universite Paris-Descartes, Paris, France

(Jouneau) Service de pneumologie, centre de competences pour les maladies pulmonaires rares, CHU de Rennes, IRSET UMR 1085, universite de Rennes 1, Rennes, France

(Kessler) Service de pneumologie, centre de competences pour les maladies pulmonaires rares, hopital civil, CHU de Strasbourg, Strasbourg, France

(Marquette) Service de pneumologie, centre de competences pour les maladies pulmonaires rares, CHU de Nice, FHU Oncoage, universite Cote d'Azur, France

(Reynaud-Gaubert) Service de pneumologie, centre de competence des maladies pulmonaires rares, CHU Nord, Marseille, France

(Aguilaniu) Universite Grenoble-Alpes, Grenoble, France

(Bonnet) Service de pneumologie, centre hospitalier de la Cote-Basque, Bayonne, France

(Carre) Service de pneumologie, centre hospitalier, Carcassonne, France

(Danel) Departement de pathologie, hopital Bichat-Claude-Bernard, universite Paris Diderot, Assistance publique-Hopitaux de Paris, Paris 7, Paris, France

(Faivre) Service d'imagerie thoracique, hopital Calmette, CHRU de Lille, Lille, France

(Ferretti) Clinique universitaire de radiologie et imagerie medicale, CHU Grenoble-Alpes, Grenoble, France

(Just) Service de pneumologie, centre hospitalier Victor-Provo, Roubaix, France

(Lebargy) Service des maladies respiratoires, CHU Maison-Blanche, Reims, France

(Philippe) Service de pneumologie, centre hospitalier Rene-Dubos, Pontoise, France

(Terrioux) Service de pneumologie, centre hospitalier de Meaux, Meaux, France

(Thivolet-Bejui) Service d'anatomie et cytologie pathologiques, hopital Louis-Pradel, Lyon, France

(Trumbic) Cap Evidence, Paris, France

(Valeyre) Service de pneumologie, centre de competences pour les maladies pulmonaires rares, hopital Avicenne, CHU Paris-Seine-Saint-Denis, Bobigny, France

Publisher

Elsevier Masson SAS (62 rue Camille Desmoulins, Issy les Moulineaux Cedex 92442, France)

Year of Publication

2017

Link to the Ovid Full Text or citation:

[Click here for full text options](https://ovidsp.ovid.com/ovidweb.cgi?T=JS&CSC=Y&NEWS=N&PAGE=fulltext&D=emed18&AN=618363209)

Link to the External Link Resolver:

[LibKey NHS](https://libkey.io/libraries/2789/openurl?genre=article&sid=OVID:emed18&genre=article&id=pmid:28939154&id=doi:10.1016%2Fj.rmr.2017.07.019&issn=0761-8425&volume=34&issue=8&spage=852&pages=852-899&date=2017&title=Revue+des+Maladies+Respiratoires&atitle=Recommandations+pratiques+pour+le+diagnostic+et+la+prise+en+charge+de+la+fibrose+pulmonaire+idiopathique+-+Actualisation+2017.+Version+courte&aulast=Cottin)

112.

French practical guidelines for the diagnosis and management of idiopathic pulmonary fibrosis - 2017 update. Full-length version. Recommandations pratiques pour le diagnostic et la prise en charge de la fibrose pulmonaire idiopathique - Actualisation 2017. Version longue <Recommandations pratiques pour le diagnostic et la prise en charge de la fibrose pulmonaire idiopathique - Actualisation 2017. Version longue.>

Cottin V., Crestani B., Cadranel J., Cordier J.-F., Marchand-Adam S., Prevot G., Wallaert B., Bergot E., Camus P., Dalphin J.-C., Dromer C., Gomez E., Israel-Biet D., Jouneau S., Kessler R., Marquette C.-H., Reynaud-Gaubert M., Aguilaniu B., Bonnet D., Carre P., Danel C., Faivre J.-B., Ferretti G., Just N., Lebargy F., Philippe B., Terrioux P., Thivolet-Bejui F., Trumbic B., Valeyre D.

Embase

Revue des Maladies Respiratoires. 34(8) (pp 900-968), 2017. Date of Publication: October 2017.

[Article]

AN: 618350536

PMID

28939155 [<https://www.ncbi.nlm.nih.gov/pubmed/?term=28939155>]

Status

Embase

Institution

(Cottin, Cordier) Centre national de reference des maladies pulmonaires rares, pneumologie, hopital Louis-Pradel, hospices civils de Lyon, universite Claude-Bernard-Lyon 1, Lyon, France (Crestani) Service de pneumologie A, centre de competences pour les maladies pulmonaires rares, CHU Bichat, universite Paris Diderot, Paris, France

(Cadranel) Service de pneumologie et oncologie thoracique, centre de competences pour les maladies pulmonaires rares, hopital Tenon, universite Pierre-et-Marie-Curie, Paris 6, GH-HUEP, Assistance publique-Hopitaux de Paris, Paris, France

(Marchand-Adam) Service de pneumologie, centre de competences pour les maladies pulmonaires rares, CHU de Tours, Tours, France

(Prevot) Service de pneumologie, centre de competences pour les maladies pulmonaires rares, CHU Larrey, Toulouse, France

(Wallaert) Service de pneumologie et immuno-allergologie, centre de competences pour les maladies pulmonaires rares, hopital Calmette, CHRU de Lille, Lille, France

(Bergot) Service de pneumologie et oncologie thoracique, centre de competences pour les maladies pulmonaires rares, CHU de Caen, Caen, France

(Camus) Service de pneumologie et oncologie thoracique, centre de competences pour les maladies pulmonaires rares, CHU Dijon-Bourgogne, Dijon, France

(Dalphin) Service de pneumologie, allergologie et oncologie thoracique, centre de competences pour les maladies pulmonaires rares, hopital Jean-Minjoz, UMR-CNRS Chrono-environnement, universite de Franche-Comte, CHRU de Besancon, Besancon, France

(Dromer) Service de pneumologie, centre de competences pour les maladies pulmonaires rares, hopital Haut-Leveque, CHU de Bordeaux, Bordeaux, France

(Gomez) Departement de pneumologie, centre de competences pour les maladies pulmonaires rares, CHU de Nancy, Vandoeuvre-les-Nancy, France

(Israel-Biet) Service de pneumologie, centre de competences pour les maladies pulmonaires rares, hopital europeen Georges-Pompidou, universite Paris-Descartes, Paris, France

(Jouneau) Service de pneumologie, centre de competences pour les maladies pulmonaires rares, CHU de Rennes, IRSET UMR 1085, universite de Rennes 1, Rennes, France

(Kessler) Service de pneumologie, centre de competences pour les maladies pulmonaires rares, hopital civil, CHU de Strasbourg, Strasbourg, France

(Marquette) Service de pneumologie, centre de competences pour les maladies pulmonaires rares, CHU de Nice, FHU Oncoage, universite Cote d'Azur, France, France

(Reynaud-Gaubert) Service de pneumologie, centre de competences des maladies pulmonaires rares, CHU Nord, URMITE, IHU Mediterranee infection, Aix-Marseille Universite, Marseille, France

(Aguilaniu) Universite Grenoble-Alpes, Grenoble, France

(Bonnet) Service de pneumologie, centre hospitalier de la Cote-Basque, Bayonne, France

(Carre) Service de pneumologie, centre hospitalier, Carcassonne, France

(Danel) Departement de pathologie, hopital Bichat-Claude-Bernard, universite Paris Diderot, Assistance publique-Hopitaux de Paris, Paris 7, Paris, France

(Faivre) Service d'imagerie thoracique, hopital Calmette, CHRU de Lille, Lille, France

(Ferretti) Clinique universitaire de radiologie et imagerie medicale, CHU Grenoble-Alpes, Grenoble, France

(Just) Service de pneumologie, centre hospitalier Victor-Provo, Roubaix, France

(Lebargy) Service des maladies respiratoires, CHU Maison-Blanche, Reims, France

(Philippe) Service de pneumologie, centre hospitalier Rene-Dubos, Pontoise, France

(Terrioux) Service de pneumologie, centre hospitalier de Meaux, Meaux, France

(Thivolet-Bejui) Service d'anatomie et cytologie pathologiques, hopital Louis-Pradel, Lyon, France

(Trumbic) Cap Evidence, Paris, France

(Valeyre) Service de pneumologie, centre de competences pour les maladies pulmonaires rares, hopital Avicenne, CHU Paris-Seine-Saint-Denis, Bobigny, France

Publisher

Elsevier Masson SAS (62 rue Camille Desmoulins, Issy les Moulineaux Cedex 92442, France)

Year of Publication

2017

Link to the Ovid Full Text or citation:

[Click here for full text options](https://ovidsp.ovid.com/ovidweb.cgi?T=JS&CSC=Y&NEWS=N&PAGE=fulltext&D=emed18&AN=618350536)

Link to the External Link Resolver:

[LibKey NHS](https://libkey.io/libraries/2789/openurl?genre=article&sid=OVID:emed18&genre=article&id=pmid:28939155&id=doi:10.1016%2Fj.rmr.2017.07.017&issn=0761-8425&volume=34&issue=8&spage=900&pages=900-968&date=2017&title=Revue+des+Maladies+Respiratoires&atitle=Recommandations+pratiques+pour+le+diagnostic+et+la+prise+en+charge+de+la+fibrose+pulmonaire+idiopathique+-+Actualisation+2017.+Version+longue&aulast=Cottin)

113.

French practical guidelines for the diagnosis and management of idiopathic pulmonary fibrosis: 2017 update. Summary. Recommandations pratiques pour le diagnostic et la prise en charge de la fibrose pulmonaire idiopathique: actualisation 2017. Resume <Recommandations pratiques pour le diagnostic et la prise en charge de la fibrose pulmonaire idiopathique: actualisation 2017. Resume.>

Cottin V., Crestani B., Cadranel J., Cordier J.-F., Marchand-Adam S., Prevot G., Wallaert B., Bergot E., Camus P., Dalphin J.-C., Dromer C., Gomez E., Israel-Biet D., Jouneau S., Kessler R., Marquette C.-H., Reynaud-Gaubert M., Aguilaniu B., Bonnet D., Carre P., Danel C., Faivre J.-B., Ferretti G., Just N., Lebargy F., Philippe B., Terrioux P., Thivolet-Bejui F., Trumbic B., Valeyre D.

Embase

Revue des Maladies Respiratoires. 34(8) (pp 834-851), 2017. Date of Publication: October 2017.

[Article]

AN: 618349453

PMID

28935495 [<https://www.ncbi.nlm.nih.gov/pubmed/?term=28935495>]

Status

Embase

Institution

(Cottin, Cordier) Centre national de reference des maladies pulmonaires rares, pneumologie, hopital Louis-Pradel, hospices civils de Lyon, universite Claude-Bernard-Lyon 1, Lyon, France (Crestani) Service de pneumologie A, centre de competences pour les maladies pulmonaires rares, CHU Bichat, universite Paris Diderot, Paris, France

(Cadranel) Service de pneumologie et oncologie thoracique, centre de competences pour les maladies pulmonaires rares, hopital Tenon, universite Pierre-et-Marie-Curie, Paris 6, GH-HUEP, Assistance publique-Hopitaux de Paris, Paris, France

(Marchand-Adam) Service de pneumologie, centre de competences pour les maladies pulmonaires rares, CHU de Tours, Tours, France

(Prevot) Service de pneumologie, centre de competences pour les maladies pulmonaires rares, CHU Larrey, Toulouse, France

(Wallaert) Service de pneumologie et immuno-allergologie, centre de competences pour les maladies pulmonaires rares, hopital Calmette, CHRU de Lille, Lille, France

(Bergot) Service de pneumologie et oncologie thoracique, centre de competences pour les maladies pulmonaires rares, CHU de Caen, Caen, France

(Camus) Service de pneumologie et oncologie thoracique, centre de competences pour les maladies pulmonaires rares, CHU Dijon-Bourgogne, Dijon, France

(Dalphin) Service de pneumologie, allergologie et oncologie thoracique, centre de competences pour les maladies pulmonaires rares, hopital Jean-Minjoz, CHRU de Besancon, Besancon, France

(Dromer) Service de pneumologie, centre de competences pour les maladies pulmonaires rares, hopital Haut-Leveque, CHU de Bordeaux, Bordeaux, France

(Gomez) Departement de pneumologie, centre de competences pour les maladies pulmonaires rares, CHU de Nancy, Vandoeuvre-les-Nancy, France

(Israel-Biet) Service de pneumologie, centre de competences pour les maladies pulmonaires rares, hopital europeen Georges-Pompidou, universite Paris-Descartes, Paris, France

(Jouneau) Service de pneumologie, centre de competences pour les maladies pulmonaires rares, CHU de Rennes, IRSET UMR 1085, universite de Rennes 1, Rennes, France

(Kessler) Service de pneumologie, centre de competences pour les maladies pulmonaires rares, hopital civil, CHU de Strasbourg, Strasbourg, France

(Marquette) Service de pneumologie, centre de competences pour les maladies pulmonaires rares, CHU de Nice, FHU Oncoage, universite Cote d'Azur, France

(Reynaud-Gaubert) Service de pneumologie, centre de competence des maladies pulmonaires rares, CHU Nord, Marseille, France

(Aguilaniu) Universite Grenoble-Alpes, Grenoble, France

(Bonnet) Service de pneumologie, centre hospitalier de la Cote-Basque, Bayonne, France

(Carre) Service de pneumologie, centre hospitalier, Carcassonne, France

(Danel) Departement de pathologie, hopital Bichat-Claude-Bernard, universite Paris Diderot, Assistance publique-Hopitaux de Paris, Paris 7, Paris, France

(Faivre) Service d'imagerie thoracique, hopital Calmette, CHRU de Lille, Lille, France

(Ferretti) Clinique universitaire de radiologie et imagerie medicale, CHU Grenoble-Alpes, Grenoble, France

(Just) Service de pneumologie, centre hospitalier Victor-Provo, Roubaix, France

(Lebargy) Service des maladies respiratoires, CHU Maison-Blanche, Reims, France

(Philippe) Service de pneumologie, centre hospitalier Rene-Dubos, Pontoise, France

(Terrioux) Service de pneumologie, centre hospitalier de Meaux, Meaux, France

(Thivolet-Bejui) Service d'anatomie et cytologie pathologiques, hopital Louis-Pradel, Lyon, France

(Trumbic) Cap Evidence, Paris, France

(Valeyre) Service de pneumologie, centre de competences pour les maladies pulmonaires rares, hopital Avicenne, CHU Paris-Seine-Saint-Denis, Bobigny, France

Publisher

Elsevier Masson SAS (62 rue Camille Desmoulins, Issy les Moulineaux Cedex 92442, France)

Year of Publication

2017

Link to the Ovid Full Text or citation:

[Click here for full text options](https://ovidsp.ovid.com/ovidweb.cgi?T=JS&CSC=Y&NEWS=N&PAGE=fulltext&D=emed18&AN=618349453)

Link to the External Link Resolver:

[LibKey NHS](https://libkey.io/libraries/2789/openurl?genre=article&sid=OVID:emed18&genre=article&id=pmid:28935495&id=doi:10.1016%2Fj.rmr.2017.07.022&issn=0761-8425&volume=34&issue=8&spage=834&pages=834-851&date=2017&title=Revue+des+Maladies+Respiratoires&atitle=Recommandations+pratiques+pour+le+diagnostic+et+la+prise+en+charge+de+la+fibrose+pulmonaire+idiopathique%3A+actualisation+2017.+Resume&aulast=Cottin)

114.

Evaluation of sleep quality and daily sleepiness in patients with chronic obstructive pulmonary disease in pulmonary rehabilitation.

Nobeschi L., Squassoni S., Cordoni P., Raimundo R., Fiss E.

Embase

European Respiratory Journal. Conference: European Respiratory Society International Congress, ERS 2017. Milan Italy. 50(Supplement 61) (no pagination), 2017. Date of Publication: September 2017.

[Conference Abstract]

AN: 625789336

Introduction: In addition to sleep disturbances, patients with chronic obstructive pulmonary disease (COPD) have common clinical features such as hypoventilation and sleep disorders. The sleep of patients with COPD is of lower efficiency, higher latency for onset and fragmented. Studies have shown that physical exercise may aid in the treatment of disturbances of the sleep-wake cycle. Pulmonary Rehabilitation Program (PRP) associates aerobic and anaerobic exercises, with the purpose of improving the signs and symptoms of the disease.

Objective(s): Identify if the PRP has an impact on the sleep quality and daily sleepiness of patients with COPD.

Method(s): 18 patients with moderate COPD who did not undergo treatment for sleep disorder and who quit smoking were recruited. The study was divided into two phases: 1-pre PRP and 2-post PRP. In phases 1 and 2, the Pittsburg scale for sleep quality assessment and the EPWORTH scale were used to assess the degree of daily sleepiness. Between phases 1 and 2 the patients performed 8 weeks of PR.

Result(s): Regarding sleep quality, 33.3% presented good sleep quality; 44.4% had poor sleep quality and 22.3% had sleep disturbance during phase 1. In stage 2, 55.6% had good sleep quality, 27.7% had poor sleep quality and 16.7% had sleep disturbance. Sleep, with significant improvement in sleep efficiency and latency. As for daytime sleepiness, 72.2% did not present daily somnolence and 27.8% presented daytime sleepiness. There was no difference in outcome for the degree of daytime somnolence between phases 1 and 2.

Conclusion(s): we observed that the PRP promotes the improvement in the sleep quality of patients with COPD.

Status

CONFERENCE ABSTRACT

Institution

(Nobeschi, Fiss) Faculdade De Medicina UNISA/Faculdade De Medician do ABC/Faculdade De Ciencias Medicas Santa Casa SP, Santo Andre/Sao Paulo, Brazil (Squassoni, Cordoni, Raimundo) Faculdade De Medicina do ABC, Santo Andre/Sao Paulo, Brazil

Publisher

European Respiratory Society

Year of Publication

2017

Link to the Ovid Full Text or citation:

[Click here for full text options](https://ovidsp.ovid.com/ovidweb.cgi?T=JS&CSC=Y&NEWS=N&PAGE=fulltext&D=emed18&AN=625789336)

Link to the External Link Resolver:

[LibKey NHS](https://libkey.io/libraries/2789/openurl?genre=article&sid=OVID:emed18&genre=article&id=pmid:&id=doi:10.1183%2F1393003.congress-2017.PA4906&issn=1399-3003&volume=50&issue=Supplement+61&spage=&pages=&date=2017&title=European+Respiratory+Journal&atitle=Evaluation+of+sleep+quality+and+daily+sleepiness+in+patients+with+chronic+obstructive+pulmonary+disease+in+pulmonary+rehabilitation&aulast=Nobeschi)

115.

Effectiveness of home based pulmonary rehabilitation for COPD patients with OSA: Overlap syndrome.

Oliveira J., Silva A., Brunetti A., Perez E., Urbano J., Insalaco G., Oliveira L.V., Sampaio L.

Embase

European Respiratory Journal. Conference: European Respiratory Society International Congress, ERS 2017. Milan Italy. 50(Supplement 61) (no pagination), 2017. Date of Publication: September 2017.

[Conference Abstract]

AN: 625788875

Introduction: Sleep disturbance is a common complaint in patients with chronic obstructive lung disease (COPD). The coexistence of obstructive sleep apnoea (OSA) and COPD is Known as overlap syndrome. Disturbed sleep has been found to be associated with more severe disease, frequent exacerbations and increased mortality in COPD. Pulmonary rehabilitation program (PRP) improves quality of life, exercise capacity, and anxiety, depression and quality of life. Its effect on sleep quality is uncertain. The potential for PRP, an important aspect in the management of COPD, but to improve sleep quality remains uncertain.

Objective(s): Our purpose was verify the effect of PRP in sleep disorders breathing in COPD patients.

Method(s): This was a randomized controlled clinical trial study, conducted in accordance with the CONSORT statement. COPD patients who adhere to the eligibility criteria were recruited consecutively, and referred to the Sleep Laboratory of Lung Institute of Cascavel (Parana, Brazil).

Result(s): The study involved 54 stable COPD patients (15 female) with a mean age of 72.7 +/- 8.1 years, mean BMI of 26.4 +/- 5.4 pre and post 26.6 +/- 5.4. The mean AHI pre PRP was 25 +/- 17 and 18.6 +/- 19 post, and lowest oxygen saturation average pre of 93.8 +/- 4.7and 94,2 +/- 4,5 post, and nadir oxygen saturation was 77,7 +/- 10 pre and post 80 +/- 9,5.

Conclusion(s): The PRP showed significant results regarding the presence of sleep disordered breathing in clinically stable COPD patients.

Status

CONFERENCE ABSTRACT

Institution

(Oliveira) Nove De Julho University (UNINOVE), Sao Paulo, Brazil (Silva, Brunetti, Urbano, Oliveira, Sampaio) Nove De Julho University (UNINOVE, Sao Paulo, Brazil

(Perez) Faculdade De Ciencias Medicas Santa Casa De Misericordia, Sao Paulo, Brazil

(Insalaco) Consiglio Nazionale delle Recherce, Palermo, Italy

Publisher

European Respiratory Society

Year of Publication

2017

Link to the Ovid Full Text or citation:

[Click here for full text options](https://ovidsp.ovid.com/ovidweb.cgi?T=JS&CSC=Y&NEWS=N&PAGE=fulltext&D=emed18&AN=625788875)

Link to the External Link Resolver:

[LibKey NHS](https://libkey.io/libraries/2789/openurl?genre=article&sid=OVID:emed18&genre=article&id=pmid:&id=doi:10.1183%2F1393003.congress-2017.PA4896&issn=1399-3003&volume=50&issue=Supplement+61&spage=&pages=&date=2017&title=European+Respiratory+Journal&atitle=Effectiveness+of+home+based+pulmonary+rehabilitation+for+COPD+patients+with+OSA%3A+Overlap+syndrome&aulast=Oliveira)

116.

A comparison of symptoms in individuals with chronic obstructive pulmonary disease.

Chen Y.-W., Road J.D., Camp P.G., Reid W.

Embase

American Journal of Respiratory and Critical Care Medicine. Conference: American Thoracic Society International Conference, ATS 2017. Washington, DC United States. 195 (no pagination), 2017. Date of Publication: 2017.

[Conference Abstract]

AN: 617709361

RATIONALE Pain, in addition to dyspnea and fatigue, is a very prevalent symptom in individuals with chronic obstructive pulmonary disease (COPD). These three symptoms are complex, may be interrelated and all are associated with decreased quality of life and physical activity levels. Evaluating dyspnea, fatigue, and pain using questionnaires with a parallel format will enable a direct comparison of the three symptoms. To date, no study has utilized questionnaires with a parallel format to quantify symptoms in COPD. The purpose of this study was to compare the relative magnitude and interference of dyspnea, fatigue, and pain in COPD. METHODS Individuals with COPD over 40 years of age were recruited from pulmonary rehabilitation programs at six sites in British Columbia, Canada. Participants were asked to complete: 1) Brief Pain Inventory (BPI); 2) Dyspnea Inventory (DI); 3) Brief Fatigue Inventory (BFI). The BPI, DI, and BFI are three questionnaires with a parallel format that include magnitude and interference domains that have 10-11 items ranked on a 10 point numeric scale. Demographic characteristics including forced expiratory volume in one second (FEV1), age, sex, height, and weight were collected. RESULTS In total, 96 respondents (57 males, 39 females) of 137 potential participants completed the questionnaires with a mean age was 71.2 +/- 9.6 years old. The mean FEV1 was 52 +/- 21% predicted, consistent with a sample of moderate to severe COPD patients. The prevalence of dyspnea, fatigue, and pain were 93%, 77%, and 71%, respectively. Comparing within participants that reported all three symptoms (n=48), the fatigue severity score was significantly higher than pain (Table 1). Dyspnea interfered with general activity more than pain (5.1 +/- 1.6 vs. 4.1 +/- 2.4; p < .05), whereas pain interfered with mood (3.6 +/- 2.8 vs. 3.3 +/- 2.6; p < .05) and sleep (4.1 +/- 2.9 vs. 3.1 +/- 2.7; p < .05) more than dyspnea and fatigue. For other items of daily living, no differences in interference were shown among the DI, BFI, and BPI. CONCLUSIONS Pain, dyspnea, and fatigue are common. Individuals with COPD reported similar severity scores on the three symptoms. Dyspnea interfered with physical activities whereas pain interfered with mood and sleep. When determining the goal of rehabilitation for individuals with COPD, how pain limits and interferes with aspects of living should be considered. (Table Presented).

Status

CONFERENCE ABSTRACT

Institution

(Chen) VancouverBCCanada (Road) Department of Medicine, University of British Columbia, Vancouver, BC, Canada

(Camp) Department of Physical Therapy, University of British Columbia, Vancouver, BC, Canada

(Reid) Department of Physical Therapy, University of Toronto, Toronto, ON, Canada

Publisher

American Thoracic Society

Year of Publication

2017

Link to the Ovid Full Text or citation:

[Click here for full text options](https://ovidsp.ovid.com/ovidweb.cgi?T=JS&CSC=Y&NEWS=N&PAGE=fulltext&D=emed18&AN=617709361)

Link to the External Link Resolver:

[LibKey NHS](https://libkey.io/libraries/2789/openurl?genre=article&sid=OVID:emed18&genre=article&id=pmid:&id=doi:10.1164%2Fajrccm-conference.2017.B49&issn=1535-4970&volume=195&issue=&spage=&pages=&date=2017&title=American+Journal+of+Respiratory+and+Critical+Care+Medicine&atitle=A+comparison+of+symptoms+in+individuals+with+chronic+obstructive+pulmonary+disease&aulast=Chen)

117.

The use of high frequency airway oscillations in chronic obstructive pulmonary disease-a pilot study.

Daynes E., Harvey-Dunstan T.C., Houchen-Wolloff L., Singh S.J.

Embase

American Journal of Respiratory and Critical Care Medicine. Conference: American Thoracic Society International Conference, ATS 2017. Washington, DC United States. 195 (no pagination), 2017. Date of Publication: 2017.

[Conference Abstract]

AN: 617709310

Rationale Chronic Obstructive Pulmonary Disease (COPD) is characterised by expiratory flow limitation resulting in symptomatic dyspnoea and reduced exercise capacity. On exertion, expiration becomes active and high pressures are necessary to achieve full expiration. Patients with COPD breathe closer to total lung capacity (TLC) and elastic recoil is reduced generating a mechanical disadvantage for muscles of respiration. [1,2] Continuous breathing near to TLC increases the sensation of dyspnoea resulting in premature exercise termination.[2] We hypothesised that using a High Frequency Airway Oscillating (HFAO) device may improve expiratory flow and decrease the sensation of dyspnoea. Methods Symptomatic patients with COPD were included (Medical Research Council [MRC] score >= 3, FEV1/FVC <0.70). Patients were excluded if they received pulmonary rehabilitation within the last 6 months. The intervention used a HFAO device, the Aerosure [Actegy Ltd], for 8 weeks, three times a day. Outcomes included the MRC Scale, maximal expiratory mouth pressures (PEmax), the incremental and endurance shuttle walk test (ISWT/ESWT), and the COPD Assessment Test (CAT). Median changes in outcomes were analysed using Wilcoxon signed rank test. Results 14 subjects with an established diagnosis of COPD (57.1% male, mean [SD] Age 66[5] years, FEV1 % predicted 46[16], FEV1/FVC ratio 0.45[0.13], median MRC 4) were included. One patient withdrew due to poor health. There was a significant change in MRC -1 [0.4] between visit one and visit two, meeting the minimal clinically important difference p=0.007. There was a statistically significant difference in PEmax p=0.013 (Table 1). There were no significant differences observed in the ISWT or ESWT. There was no significant difference in the CAT total, however a significant difference was observed in the sleep domain p=0.041. Conclusions The use of a HFAO device for 8 weeks improves PEmax and reduces symptomatic dyspnoea. This improvement was not translated to increased exercise capacity, however patients sleep quality improved. A full randomised control trial is necessary to explore the effects of HFAO further. (Table Presented).

Status

CONFERENCE ABSTRACT

Institution

(Daynes, Harvey-Dunstan, Houchen-Wolloff, Singh) University Hospitals of Leicester, Leicester, United Kingdom

Publisher

American Thoracic Society

Year of Publication

2017

Link to the Ovid Full Text or citation:

[Click here for full text options](https://ovidsp.ovid.com/ovidweb.cgi?T=JS&CSC=Y&NEWS=N&PAGE=fulltext&D=emed18&AN=617709310)

Link to the External Link Resolver:

[LibKey NHS](https://libkey.io/libraries/2789/openurl?genre=article&sid=OVID:emed18&genre=article&id=pmid:&id=doi:10.1164%2Fajrccm-conference.2017.B73&issn=1535-4970&volume=195&issue=&spage=&pages=&date=2017&title=American+Journal+of+Respiratory+and+Critical+Care+Medicine&atitle=The+use+of+high+frequency+airway+oscillations+in+chronic+obstructive+pulmonary+disease-a+pilot+study&aulast=Daynes)

118.

Expiratory central airway collapse and medical management: Outcome analysis.

Alape D., Kheir F., Gangadharan S., Mallur P., Rivera-Flores E., Majid A.

Embase

American Journal of Respiratory and Critical Care Medicine. Conference: American Thoracic Society International Conference, ATS 2017. Washington, DC United States. 195 (no pagination), 2017. Date of Publication: 2017.

[Conference Abstract]

AN: 617706719

Rationale Previous studies have shown that patients with severe symptomatic expiratory central airway collapse (ECAC) will benefit from tracheobronchoplasty (TBP) following a short stent trial.1 However, there is a subgroup of patients who are not candidates for surgery, do not tolerate airway stenting or do not wish to pursue an invasive procedure. In such population, the management of co-exiting conditions and ECAC-specific symptoms is the only therapeutic option. Currently, there is no treatment consensus. The aim of this study was to evaluate the impact of medical therapy in this patient population. Methods This was a retrospective review from prospectively collected data encompassing patients with severe and symptomatic ECAC unable or unwilling to undergo TBP. All patients were treated for comorbid conditions such as COPD, asthma, obstructive sleep apnea (OSA), gastroesophageal reflux disease (GERD) and paradoxical vocal cord motion (PVFM) in accordance with current guidelines. In addition, patients were started on ECAC-specific treatment such as flutter valve, pursed-lip breathing, noninvasive ventilation, pulmonary rehabilitation, antitussives and mucolytics based on symptoms. Demographic and clinical characteristics were described. Self- reported symptoms and dyspnea scale (modified Medical Research (mMRC)) were compared at baseline and at 3 month. Results 23 patients (18 women) with a median age of 65 years [IQR 54-73] were included. 18 (78.3%) had GERD, 10 (43.5%) had asthma, 7(30.4%) had OSA, 6 (26.1%) had COPD, 3 (13%) had PVFM. Presenting symptoms were cough in 21 patients (91.3%), dyspnea in 16(69.6%), mucostasis in 6 (26.1%) and recurrent infections in 5(21.7%). 12/18 patients (66%) with GERD, 1 /6 (16%) with COPD, 2 /10 (20%) with asthma and 3/3 with PVFM were not receiving optimal medical management. At baseline clinical visit, flutter valves were recommended in 20 patients (86%), mucolytics in 8 patients (34%), non-invasive ventilation in 7 patients (43%), pulmonary rehabilitation in 5 patients (21%) and antitussives in 4 patients (17%). Overall, there was a clinical significant improvement in mMRC (p 0.046). Dyspnea, cough, and secretion clearance improved in 56, 61, and 66%, respectively. Conclusion Medical management of ECAC-related symptoms and comorbidities such as GERD, PVFM, COPD, asthma and OSA improves respiratory symptoms and dyspnea score on such population. Our study high lights the importance of a multidisciplinary approach for the evaluation and management of patients with severe symptomatic ECAC.

Status

CONFERENCE ABSTRACT

Institution

(Alape, Kheir, Gangadharan, Rivera-Flores, Majid) Beth Israel Deaconess Medical Center, Harvard Medical School, Boston, MA, United States (Mallur) Beth Israel Deaconess Medical Center, Boston, MA, United States

Publisher

American Thoracic Society

Year of Publication

2017

Link to the Ovid Full Text or citation:

[Click here for full text options](https://ovidsp.ovid.com/ovidweb.cgi?T=JS&CSC=Y&NEWS=N&PAGE=fulltext&D=emed18&AN=617706719)

Link to the External Link Resolver:

[LibKey NHS](https://libkey.io/libraries/2789/openurl?genre=article&sid=OVID:emed18&genre=article&id=pmid:&id=doi:10.1164%2Fajrccm-conference.2017.A101&issn=1535-4970&volume=195&issue=&spage=&pages=&date=2017&title=American+Journal+of+Respiratory+and+Critical+Care+Medicine&atitle=Expiratory+central+airway+collapse+and+medical+management%3A+Outcome+analysis&aulast=Alape)

119.

Age, gender, neck circumference, and Epworth sleepiness scale do not predict obstructive sleep apnea (OSA) in moderate to severe chronic obstructive pulmonary disease (COPD): The challenge to predict OSA in advanced COPD.

Soler X., Liao S.-Y., Marin J.M., Lorenzi-Filho G., Jen R., DeYoung P., Owens R.L., Ries A.L., Malhotra A.

Embase

PLoS ONE. 12(5) (no pagination), 2017. Article Number: e0177289. Date of Publication: May 2017.

[Article]

AN: 616266670

The combination of chronic obstructive pulmonary disease (COPD) and obstructive sleep apnea (OSA) is associated with substantial morbidity and mortality. We hypothesized that predictors of OSA among patients with COPD may be distinct from OSA in the general population. Therefore, we investigated associations between traditional OSA risk factors (e.g. age), and sleep questionnaires [e.g. Epworth Sleepiness Scale] in 44 patients with advanced COPD. As a second aim we proposed a pilot, simplified screening test for OSA in patients with COPD. In a prospective, observational study of patients enrolled in the UCSD Pulmonary Rehabilitation Program we collected baseline characteristics, cardiovascular events (e.g. atrial fibrillation), and sleep questionnaires [e.g. Pittsburgh Sleep Quality Index (PSQI)]. For the pilot questionnaire, a BMI >=25 kg/m2 and the presence of cardiovascular disease were used to construct the pilot screening test. Male: 59%; OSA 66%. FEV1 (mean +/- SD) = 41.0+/-18.2% pred., FEV1/FVC = 41.5+/-12.7%]. Male gender, older age, and large neck circumference were not associated with OSA. Also, Epworth Sleepiness Scale and the STOP-Bang questionnaire were not associated with OSA in univariate logistic regression. In contrast, BMI >=25 kg/m2 (OR = 3.94, p = 0.04) and diagnosis of cardiovascular disease (OR = 5.06, p = 0.03) were significantly associated with OSA [area under curve (AUC) = 0.74]. The pilot COPD-OSA test (OR = 5.28, p = 0.05) and STOP-Bang questionnaire (OR = 5.13, p = 0.03) were both associated with OSA in Receiver Operating Characteristics (ROC) analysis. The COPD-OSA test had the best AUC (0.74), sensitivity (92%), and specificity (83%). A tenfold cross-validation validated our results. We found that traditional OSA predictors (e.g. gender, Epworth score) did not perform well in patients with more advanced COPD. Our pilot test may be an easy to implement instrument to screen for OSA. However, a larger validation study is necessary before further clinical implementation is warranted.

Copyright © 2017 Soler et al. This is an open access article distributed under the terms of the Creative Commons Attribution License, which permits unrestricted use, distribution, and reproduction in any medium, provided the original author and source are credited.

PMID

28510598 [<https://www.ncbi.nlm.nih.gov/pubmed/?term=28510598>]

Status

Embase

Institution

(Soler, Jen, DeYoung, Owens, Ries, Malhotra) Department of Medicine, Pulmonary, Critical Care and Sleep Division, UC San Diego, San Diego, CA, United States (Liao) Department of Medicine, UC Riverside, Riverside, CA, United States

(Marin) Pulmonary Division, University Hospital Miguel Servet, IISAragon, CIBERES, Zaragoza, Spain

(Lorenzi-Filho) Pulmonary Division, Sleep Laboratory, Hospital Das Clinicas, Faculda de de Medicina, Universidade de Sao Paulo, Sao Paulo, Brazil

Publisher

Public Library of Science (E-mail: plos@plos.org)

Year of Publication

2017

Link to the Ovid Full Text or citation:

[Click here for full text options](https://ovidsp.ovid.com/ovidweb.cgi?T=JS&CSC=Y&NEWS=N&PAGE=fulltext&D=emed18&AN=616266670)

Link to the External Link Resolver:

[LibKey NHS](https://libkey.io/libraries/2789/openurl?genre=article&sid=OVID:emed18&genre=article&id=pmid:28510598&id=doi:10.1371%2Fjournal.pone.0177289&issn=1932-6203&volume=12&issue=5&spage=e0177289&pages=&date=2017&title=PLoS+ONE&atitle=Age%2C+gender%2C+neck+circumference%2C+and+Epworth+sleepiness+scale+do+not+predict+obstructive+sleep+apnea+(OSA)+in+moderate+to+severe+chronic+obstructive+pulmonary+disease+(COPD)%3A+The+challenge+to+predict+OSA+in+advanced+COPD&aulast=Soler)

120.

Management of stable chronic obstructive pulmonary disease.

Aljaafareh A., Fakih H.A.M., Biswas A.

Embase

Journal of Clinical Outcomes Management. 24(6) (pp 273-288), 2017. Date of Publication: June 2017.

[Article]

AN: 616844432

* Objective: Objective: To review the management of stable chronic obstructive pulmonary disease (COPD). * Methods: Review of the peer-reviewed literature. * Results: Effective management of stable COPD requires the physician to apply a stepwise intensification of therapy depending on patient symptoms and functional reserve. Bronchodilators are the cornerstone of management. In addition to pharmacologic therapies, nonpharmacologic therapies, including smoking cessation, vaccinations, proper nutrition, and maintaining physical activity, are an important part of long-term management. Those who continue to be symptomatic despite appropriate maximal therapy may be candidates for lung volume reduction. Palliative care services for COPD patients, which can aid in reducing symptom burden and improving quality of life, should not be overlooked. *

Conclusion(s): Successful management of stable COPD requires a multidisciplinary approach that utilizes various medical therapies as well as nonpharmacologic interventions.

Copyright 2017 by Turner White Communications Inc. All rights reserved.

Status

Embase

Institution

(Aljaafareh, Fakih, Biswas) Division of Pulmonary Critical Care Medicine, University of Florida, Gainesville, FL, United States

Publisher

Turner White Communications Inc.

Year of Publication

2017

Link to the Ovid Full Text or citation:

[Click here for full text options](https://ovidsp.ovid.com/ovidweb.cgi?T=JS&CSC=Y&NEWS=N&PAGE=fulltext&D=emed18&AN=616844432)

Link to the External Link Resolver:

[LibKey NHS](https://libkey.io/libraries/2789/openurl?genre=article&sid=OVID:emed18&genre=article&id=pmid:&id=doi:&issn=1079-6533&volume=24&issue=6&spage=273&pages=273-288&date=2017&title=Journal+of+Clinical+Outcomes+Management&atitle=Management+of+stable+chronic+obstructive+pulmonary+disease&aulast=Aljaafareh)

121.

Pulmonary hypertension due to common respiratory conditions: Classification, evaluation and management strategies.

Fein D.G., Zaidi A.N., Sulica R.

Embase

Journal of Clinical Medicine. 5(9) (no pagination), 2016. Article Number: 75. Date of Publication: September 2016.

[Article]

AN: 612026521

Pulmonary hypertension (PH) due to chronic respiratory disease and/or hypoxia is classified as World Health Organization (WHO) Group III pulmonary hypertension. The patients most commonly encountered in clinical practice with group III PH include those with chronic obstructive lung disease (COPD), diffuse parenchymal lung disease, and sleep-disordered breathing. The purpose of this review is to outline the variable clinical significance of pulmonary hypertension in the most common pulmonary disease states and how a clinician may approach the management of these patients.

Copyright © 2016 by the authors; licensee MDPI, Basel, Switzerland.

Status

Embase

Institution

(Fein) Division of Pulmonary Medicine, Montefiore Medical Center, 111 East 210th Street, Bronx, NY 10467, United States (Zaidi) Division of Cardiology, Montefiore Medical Center, 111 East 210th Street, Bronx, NY 10467, United States

(Sulica) Division of Pulmonary Critical Care and Sleep Medicine, Mount Sinai Beth Israel, 7 Dazian Building, 1st Avenue at 16th Street, New York, NY 10003, United States

Publisher

MDPI

Year of Publication

2016

Link to the Ovid Full Text or citation:

[Click here for full text options](https://ovidsp.ovid.com/ovidweb.cgi?T=JS&CSC=Y&NEWS=N&PAGE=fulltext&D=emed17&AN=612026521)

Link to the External Link Resolver:

[LibKey NHS](https://libkey.io/libraries/2789/openurl?genre=article&sid=OVID:emed17&genre=article&id=pmid:&id=doi:10.3390%2Fjcm5090075&issn=2077-0383&volume=5&issue=9&spage=75&pages=&date=2016&title=Journal+of+Clinical+Medicine&atitle=Pulmonary+hypertension+due+to+common+respiratory+conditions%3A+Classification%2C+evaluation+and+management+strategies&aulast=Fein)

122.

Anxiety and depression: Easing the burden in COPD patients.

Marsh S., Guck T.P.

Embase

Journal of Family Practice. 65(4) (pp 246-256), 2016. Date of Publication: April 2016.

[Article]

AN: 610007826

PMID

27262248 [<https://www.ncbi.nlm.nih.gov/pubmed/?term=27262248>]

Status

Embase

Institution

(Marsh, Guck) Department of Family Medicine, Creighton University School of Medicine, 3009 S 35th Ave., Omaha, NE 68105, United States

Publisher

Family Physicians Inquiries Network

Year of Publication

2016

Link to the Ovid Full Text or citation:

[Click here for full text options](https://ovidsp.ovid.com/ovidweb.cgi?T=JS&CSC=Y&NEWS=N&PAGE=fulltext&D=emed17&AN=610007826)

Link to the External Link Resolver:

[LibKey NHS](https://libkey.io/libraries/2789/openurl?genre=article&sid=OVID:emed17&genre=article&id=pmid:27262248&id=doi:&issn=0094-3509&volume=65&issue=4&spage=246&pages=246-256&date=2016&title=Journal+of+Family+Practice&atitle=Anxiety+and+depression%3A+Easing+the+burden+in+COPD+patients&aulast=Marsh)

123.

Pulmonary rehabilitation in COPD according to global initiative for chronic obstructive lung disease categories.

Alfarroba S., Rodrigues F., Papoila A.L., Santos A.F., Morais L.

Embase

Respiratory Care. 61(10) (pp 1331-1340), 2016. Date of Publication: 01 Oct 2016.

[Article]

AN: 612459537

BACKGROUND: Pulmonary rehabilitation (PR) programs are a mainstay for treatment in COPD. Lung function impairment alone does not predict beneficial effects of PR. The new COPD categories take into account assessment of symptoms, such as dyspnea and exacerbations, which may be important indications for PR. This study evaluates the effect of PR on exercise capacity, symptoms, and health status in different COPD categories.

METHOD(S): Subjects with COPD referred for PR were classified into COPD categories A, B, C, and D. Exercise capacity (6-min walk distance [6MWD] and constant work rate at 80% of peak work rate), symptoms (Mahler's index), and health status (St George Respiratory Questionnaire) were compared before and after PR programs for each COPD category. Changes were analyzed using generalized estimating equations and logistic regression models.

RESULT(S): One hundred sixty-seven subjects were included (COPD categories A [16%], B [12%], C [31%], and D [41%]). Groups were homogeneous in age, body mass index, smoking pack-years, and comorbidities. Significant improvements in all outcomes were found after adjusting for COPD categories, age, sex, body mass index, and COPD-specific comorbidity index. All COPD categories improved exercise capacity (6MWD and constant work rate). Categories A and C had more pronounced improvements in 6MWD than categories B and D. Symptoms (Mahler's index) also improved significantly in categories A and C, whereas change was not significant in categories B and D. Global health status (St George Respiratory Questionnaire) improved significantly in all COPD categories. Despite these differences, the odds of achieving a minimum clinically important difference in each outcome were similar and without statistical significance for the A, B, and C categories when compared with D.

CONCLUSION(S): This study demonstrates that patients in all COPD categories may improve exercise capacity, symptoms, and health status with PR programs, and COPD categories alone may not be sufficient to discriminate which patients may benefit most from them.

Copyright © 2016 Daedalus Enterprises.

PMID

27407179 [<https://www.ncbi.nlm.nih.gov/pubmed/?term=27407179>]

Status

Embase

Institution

(Alfarroba, Rodrigues, Santos, Morais) Servico de Pneumologia, Hospital Pulido Valente, Centro Hospitalar Lisboa Norte, Lisbon, Portugal (Rodrigues, Papoila) NOVA Medical School/Faculdade de Ciencias Medicas, Universidade Nova de Lisboa, Lisbon, Portugal

(Papoila) Centro de Estatistica e Aplicacoes da Universidade de Lisboa, Lisbon, Portugal

Publisher

American Association for Respiratory Care

Year of Publication

2016

Link to the Ovid Full Text or citation:

[Click here for full text options](https://ovidsp.ovid.com/ovidweb.cgi?T=JS&CSC=Y&NEWS=N&PAGE=fulltext&D=emed17&AN=612459537)

Link to the External Link Resolver:

[LibKey NHS](https://libkey.io/libraries/2789/openurl?genre=article&sid=OVID:emed17&genre=article&id=pmid:27407179&id=doi:10.4187%2Frespcare.04414&issn=0020-1324&volume=61&issue=10&spage=1331&pages=1331-1340&date=2016&title=Respiratory+Care&atitle=Pulmonary+rehabilitation+in+COPD+according+to+global+initiative+for+chronic+obstructive+lung+disease+categories&aulast=Alfarroba)

124.

Action plans for COPD: Strategies to manage exacerbations and improve outcomes.

Jalota L., Jain V.V.

Embase

International Journal of COPD. 11(1) (pp 1179-1188), 2016. Date of Publication: 02 Jun 2016.

[Review]

AN: 610657890

COPD is the third-largest killer in the world, and certainly takes a toll on the health care system. Recurrent COPD exacerbations accelerate lung-function decline, worsen mortality, and consume over US$50 billion in health care spending annually. This has led to a tide of payment reforms eliciting interest in strategies reducing preventable COPD exacerbations. In this review, we analyze and discuss the evidence for COPD action plan-based self-management strategies. Although action plans may provide stabilization of acute symptomatology, there are several limitations. These include patient-centered attributes, such as comprehension and adherence, and nonadherence of health care providers to established guidelines. While no single intervention can be expected independently to translate into improved outcomes, structured together within a comprehensive integrated disease-management program, they may provide a robust paradigm.

Copyright © 2016 Jalota and Jain.

Status

Embase

Institution

(Jalota, Jain) Department of Internal Medicine, Division of Pulmonary and Critical Care Medicine, University of California, San Francisco, United States (Jain) UCSF-Fresno, Community Regional Medical Center, Fresno, CA, United States

Publisher

Dove Medical Press Ltd. (PO Box 300-008, Albany, Auckland, New Zealand)

Year of Publication

2016

Link to the Ovid Full Text or citation:

[Click here for full text options](https://ovidsp.ovid.com/ovidweb.cgi?T=JS&CSC=Y&NEWS=N&PAGE=fulltext&D=emed17&AN=610657890)

Link to the External Link Resolver:

[LibKey NHS](https://libkey.io/libraries/2789/openurl?genre=article&sid=OVID:emed17&genre=article&id=pmid:&id=doi:10.2147%2FCOPD.S76970&issn=1176-9106&volume=11&issue=1&spage=1179&pages=1179-1188&date=2016&title=International+Journal+of+COPD&atitle=Action+plans+for+COPD%3A+Strategies+to+manage+exacerbations+and+improve+outcomes&aulast=Jalota)

125.

Patient Involvement in the Design of a Patient-Centered Clinical Trial to Promote Adherence to Supplemental Oxygen Therapy in COPD.

Holm K.E., Casaburi R., Cerreta S., Gussin H.A., Husbands J., Porszasz J., Prieto-Centurion V., Sandhaus R.A., Sullivan J.L., Walsh L.J., Krishnan J.A.

Embase

Patient. 9(3) (pp 271-279), 2016. Date of Publication: 01 Jun 2016.

[Article]

AN: 606695125

Background: Patients are increasingly viewed as key stakeholders who can contribute in meaningful ways to clinical research and are emphasized in research funded by the Patient-Centered Outcomes Research Institute (PCORI). We are not aware of other peer-reviewed publications that report methods and outcomes of patient engagement to refine study design for a PCORI-sponsored clinical effectiveness trial.

Objective(s): The aim of this report was to describe the process and outcomes of involving patients in the design of a clinical trial to promote adherence to supplemental oxygen therapy among patients with chronic obstructive pulmonary disease.

Method(s): In-person focus groups and individual discussions via telephone and email were used to elicit feedback to refine the intervention and clarify outcomes of highest importance to patients.

Result(s): A total of 25 patients and five caregivers provided feedback. Their feedback has informed decisions regarding the length of intervention sessions (20 min and in some cases longer was acceptable), the importance of including caregivers, and discussion topics (e.g., social discomfort about using oxygen in public, identifying personally relevant reasons to use oxygen, pulmonary rehabilitation). Multiple outcomes were rated as highly important to patients (physical function, fatigue, sleep, anxiety, depression, and ability to participate in social roles and activities), and the outcome that was ranked as most important varied by individual. Therefore, multiple patient-reported outcomes will be used as endpoints for the clinical trial.

Conclusion(s): Patient involvement led to refinements of the intervention and clinical trial endpoints to better address the expressed needs and concerns of patients and caregivers.

Copyright © 2015, Springer International Publishing Switzerland.

Status

In-Process

Institution

(Holm, Sandhaus) Department of Medicine, National Jewish Health, Denver, CO, United States (Holm) Department of Community and Behavioral Health, Colorado School of Public Health, Aurora, CO, United States

(Casaburi, Porszasz) Rehabilitation Clinical Trials Center, Los Angeles Biomedical Research Institute at Harbor-UCLA Medical Center, Torrance, CA, United States

(Cerreta, Sullivan, Walsh) COPD Foundation, Washington, DC, United States

(Gussin, Krishnan) Population Health Sciences Program, University of Illinois Hospital and Health Sciences System, Chicago, IL, United States

(Husbands) Apria Healthcare, Lake Forest, CA, United States

(Prieto-Centurion, Krishnan) Department of Medicine, University of Illinois at Chicago, Chicago, IL, United States

(Sandhaus) AlphaNet, Miami, FL, United States

(Sandhaus) Alpha-1 Foundation, Miami, FL, United States

Publisher

Springer International Publishing

Year of Publication

2016

Link to the Ovid Full Text or citation:

[Click here for full text options](https://ovidsp.ovid.com/ovidweb.cgi?T=JS&CSC=Y&NEWS=N&PAGE=fulltext&D=emed17&AN=606695125)

Link to the External Link Resolver:

[LibKey NHS](https://libkey.io/libraries/2789/openurl?genre=article&sid=OVID:emed17&genre=article&id=pmid:&id=doi:10.1007%2Fs40271-015-0150-z&issn=1178-1653&volume=9&issue=3&spage=271&pages=271-279&date=2016&title=Patient&atitle=Patient+Involvement+in+the+Design+of+a+Patient-Centered+Clinical+Trial+to+Promote+Adherence+to+Supplemental+Oxygen+Therapy+in+COPD&aulast=Holm)

126.

A feasibility pilot study of promis measures in people with copd participating in pulmonary rehabilitation.

Tappan R., Kalhan R., Yount S.

Embase

Cardiopulmonary Physical Therapy Journal. Conference: Combined Sections Meeting of the American Physical Therapy Association, CSM 2016. Anaheim, CA United States. 27(1) (pp 36), 2016. Date of Publication: January 2016.

[Conference Abstract]

AN: 623934530

Purpose/Hypothesis: Pulmonary rehabilitation (PR) hasdemonstrated benefits in people with chronic obstructivepulmonary disease (COPD) for cardiovascular function, walkingability, dyspnea, and health-related quality of life. While the St.George's Respiratory Questionnaire (SGRQ) and the ChronicRespiratory Questionnaire, have been shown to be reliable, validand responsive for measuring change in people with COPDparticipating in PR, they are cumbersome to administer and score.This can result in incomplete and/or missing data. Measures fromthe Patient Reported Outcomes Measurement Information System(PROMIS) can be administered as computer adaptive tests (CATs),providing brief, efficient and precise assessment and scoring ofrelevant health domains. The PROMIS Dyspnea measures havebeen shown to be reliable and valid in people with COPD, butPROMIS measures haven't been utilized in PR. Objectives are to:(1) evaluate the feasibility and acceptability of in-clinic, longitudinal data collection via iPad; and (2) explore the responsiveness ofPROMIS measures in a small sample of people with COPDparticipating in PR. Number of Subjects: 14 enrolled, 3 todate have completed the study. Materials/Methods: Participants in an 8-week PR program were administered the ModifiedMedical Research Council dyspnea scale, the PROMIS DyspneaFunctional Limitations (short form) and PROMIS Function,Fatigue, Depression, Anxiety, Sleep Disturbance, and SleepRelated Impairment CATs via iPad. Regularly administered PRoutcome measures (OMs), SGRQ and 6-Minute Walk Test, werealso completed. Measures were collected prior to initiating PR(baseline), midway through PR (4-6 weeks), and at the conclusionPR (8-10 weeks).

Result(s): Of 16 consecutive people eligible forthe study, 14 agreed to participate. To date, 3 have completed thestudy, and +/- of 7 current participants have completed the midwayassessment. 4 withdrew from PR (and study): 2 for medicalreasons, 1 completed the program early, and 1 for personalreasons. While 7 of 14 participants were not experienced with theiPad prior to this study, all were able to use the iPad to completethe CATs after 5 to 15 minutes of instruction. We will presentlongitudinal PROMIS data, incomplete/missing data percentagesand anecdotal comments of participants about completing assessments with an iPad.

Conclusion(s): We anticipate thesepreliminary data will show PROMIS CATs via iPad to be a feasible,efficient and effective method of gathering OMs from people withCOPD participating in PR. We also expect to provide preliminaryevidence of the responsiveness of PROMIS measures in peoplewith COPD undergoing PR. CLINICAL RELEVANCE: OMs arecritical for determining improvement in individual patients and formeasuring impact of PR programs. These preliminary Resultssupport the use of an iPad and PROMIS measures compared toother commonly used measures in order to more efficiently collectdata in the clinic.

Status

CONFERENCE ABSTRACT

Institution

(Tappan, Kalhan, Yount) Allied Health Outpatient, Rehabilitation Institute of Chicago, Chicago, IL; Department of Medicine and Preventive Medicine, Northwestern University Feinberg School of Medicine, Chicago, IL; Department of Medical Social Sciences, Northwestern University Feinberg School of Medicine, Chicago, IL

Publisher

Lippincott Williams and Wilkins

Year of Publication

2016

Link to the Ovid Full Text or citation:

[Click here for full text options](https://ovidsp.ovid.com/ovidweb.cgi?T=JS&CSC=Y&NEWS=N&PAGE=fulltext&D=emed17&AN=623934530)

Link to the External Link Resolver:

[LibKey NHS](https://libkey.io/libraries/2789/openurl?genre=article&sid=OVID:emed17&genre=article&id=pmid:&id=doi:10.1097%2FCPT.0000000000000025&issn=2374-8907&volume=27&issue=1&spage=36&pages=36&date=2016&title=Cardiopulmonary+Physical+Therapy+Journal&atitle=A+feasibility+pilot+study+of+promis+measures+in+people+with+copd+participating+in+pulmonary+rehabilitation&aulast=Tappan)

127.

Sleep disorders breathing in COPD patients after pulmonary rehabilitation.

Oliveira J.C., Perez E.A., Insalaco G., Oliveira L.V.F.

Embase

Journal of Sleep Research. Conference: 23rd Congress of the European Sleep Research Society, ESRS 2016. Bologna Italy. 25(Supplement 1) (pp 270), 2016. Date of Publication: September 2016.

[Conference Abstract]

AN: 612113826

Objectives: Verify the effect of pulmonary rehabilitation in sleep disorders breathing in COPD patients.

Method(s): This was a randomized controlled clinical trial study, Conducted in Accordance with the CONSORT statement. COPD Patients who adhere to the eligibility criteria Were Recruited consecutively, and Referred to the Sleep Laboratory of Lung Institute of Cascavel (Parana, Brazil).

Result(s): The study Involved 40 stable COPD Patients (8 female) with a mean age of 72.6 +/- 9 years, mean body mass index of 25.8 +/- 4.7 pre and post 25.6 +/- 4.8 (P value 00:56). The mean apnea/hypopnea index per hour pre pulmonary rehabilitation program was 25 +/- 3.1 and 19.2 +/- 13.3 pos (P value 0.045), and lowest oxygen saturation average pre of 90.2 +/- 3.7 and 91.3 +/- 3.7 pos (P value 0.042); and nadir oxygen saturation was 76.6 +/- 1 pre and post 81.19 +/- 8.2 (P value 0.007).

Conclusion(s): The pulmonary rehabilitation program showed significant results regarding the presence of sleep-disordered breathing in Clinically stable COPD Patients.

Status

CONFERENCE ABSTRACT

Institution

(Oliveira, Oliveira) Sleep Laboratory, Rehabilitation Sciences Master's and PhD Degree Program, Nove De Julho University (UNINOVE), Sao Paulo, Brazil (Perez) Faculdade De Ciencias Medicas Da Santa Casa De Sao Paulo, Sao Paulo, Brazil

(Insalaco) National Research Council of Italy, Institute of Biomedicine and Molecular Immunology Alberto Monroy - CNR, Palermo, Italy

Publisher

Blackwell Publishing Ltd

Year of Publication

2016

Link to the Ovid Full Text or citation:

[Click here for full text options](https://ovidsp.ovid.com/ovidweb.cgi?T=JS&CSC=Y&NEWS=N&PAGE=fulltext&D=emed17&AN=612113826)

Link to the External Link Resolver:

[LibKey NHS](https://libkey.io/libraries/2789/openurl?genre=article&sid=OVID:emed17&genre=article&id=pmid:&id=doi:10.1111%2Fjsr.12446&issn=1365-2869&volume=25&issue=Supplement+1&spage=270&pages=270&date=2016&title=Journal+of+Sleep+Research&atitle=Sleep+disorders+breathing+in+COPD+patients+after+pulmonary+rehabilitation&aulast=Oliveira)

128.

Overlap syndrome (chronic obstructive pulmonary disease and respiratory sleep disorders)-experience of Pulmonology Clinic Tg. Mures, Romania.

Jimborean G., Dombi I., Comes A., Ianosi E.S.

Embase

Journal of Sleep Research. Conference: 23rd Congress of the European Sleep Research Society, ESRS 2016. Bologna Italy. 25(Supplement 1) (pp 357), 2016. Date of Publication: September 2016.

[Conference Abstract]

AN: 612113410

Objectives: Analysis of the clinical aspects and treatment in overlap syndrome (OS) (chronic obstructive pulmonary disease COPD and respiratory sleep disorders RSD).

Method(s): 90 COPD patients (85.3% males, 85.3% smokers) were suspected having concomitant RSD after clinical examination and sleep questionnaires thereby we performed sleep polygraphy and extended consults.

Result(s): 91.1% from our group had RSD: obstructive sleep apnea (OSA) 90.2% (24.3% moderate, 65.7% severe) and 19.5% hypoventilation- obesity syndrome (HOS). 18.3% of OS were overweight and 73.1% obese. Age: 80% between 30-70 years old (54.8% <60). Patients with OS presented severe complication/comorbidities: hypertension 63.4%, cor pulmonale 43.9%, 31.7% arrithmia, 32.9% cardiac failure; 39% dyslipidemia; 31.7% diabetes. 91.4% of OS were never investigated for RSD. We performed in the second night titration for CPAP/BiPAP therapy. Treatment had an interdisciplinary approach: combined bronchodilators, treatment of comorbities, pulmonary rehabilitation, tobacco/alcohol cessation counseling, CPAP/ BiPAP + oxygen in remaining hypoxemic patients. 51.2% of patients had not accessibility to CPAP (lack of financial coverage by the public health system).

Conclusion(s): OS included clinical aspects of severity due to both COPD and RSD. Detailed medical history, sleep questionnaires are recommended for active RSD seeking in COPD. Sleep polygraphy was a cheap tool for OSA diagnosis. Adherence to CPAP is still low despite the severe OSA and the repeated educational messages.

Status

CONFERENCE ABSTRACT

Institution

(Jimborean, Dombi, Comes, Ianosi) Department M4 Pulmonology Discipline, University of Medicine and Pharmacy Tg. Mures, Tirgu Mures, Romania

Publisher

Blackwell Publishing Ltd

Year of Publication

2016

Link to the Ovid Full Text or citation:

[Click here for full text options](https://ovidsp.ovid.com/ovidweb.cgi?T=JS&CSC=Y&NEWS=N&PAGE=fulltext&D=emed17&AN=612113410)

Link to the External Link Resolver:

[LibKey NHS](https://libkey.io/libraries/2789/openurl?genre=article&sid=OVID:emed17&genre=article&id=pmid:&id=doi:10.1111%2Fjsr.12446&issn=1365-2869&volume=25&issue=Supplement+1&spage=357&pages=357&date=2016&title=Journal+of+Sleep+Research&atitle=Overlap+syndrome+(chronic+obstructive+pulmonary+disease+and+respiratory+sleep+disorders)-experience+of+Pulmonology+Clinic+Tg.+Mures%2C+Romania&aulast=Jimborean)

129.

Limited recommendations within copd clinical practice guidelines concerning optimising physical activity, sedentary behaviour and sleep: A systematic review.

Lewthwaite H., Effing T., Olds T., Williams M.

Embase

Respirology. Conference: 2016 Annual Scientific Meetings of the Thoracic Society of Australia and New Zealand and the Australian and New Zealand Society of Respiratory Science, TSANZSRS 2016. Perth, WA Australia. Conference Publication: (var.pagings). 21(SUPPL. 2) (pp 61), 2016. Date of Publication: April 2016.

[Conference Abstract]

AN: 72316588

Introduction/Aim: Physical activity, sedentary behaviour and sleep have strong associations with health. This systematic review aimed to identify recommendations and strategies reported in clinical practice guidelines for COPD for adopting and maintaining optimal physical activity, sedentary behaviour and sleep.

Method(s): A systematic search of databases (Medline, Scopus, CiNAHL, EMbase and clinical guildine) identified current versions of evidence-based clinical practice guidelines for the management of COPD created by authorative bodies published in English. Studies published from 2005 to present were sought. Primary data regarding recommendations (frequency, duration, intensity and type) and strategies for adopting and maintaining optimal physical activity, sedentary behaviour and sleep were extracted verbatim and synthesized into common categories.

Result(s): Twenty clinical guidelines (from 2379 citations identified) were eligible for inclusion. All guidelines recommended pulmonary rehabilitation (PR); however, only 11 recommended physical activity outside the PR program and reported optimal frequency (n = 7), duration (n = 2), intensity (n = 1) and type (n = 3). No guideline provided recommendations for optimal sedentary behaviour or sleep. Strategies to adopt or maintain optimal behaviours were reported for physical activity (n = 16), sedentary behaviour (n = 2) and sleep (n = 4). Physician encouragement (physical activity n = 8, sedentary n = 1), education (physical activity n = 6, sedentary n = 1) and supplemental oxygen (physical activity n = 5, sleep n = 3) were the most frequently reported strategies.

Conclusion(s): Current evidence-based clinical practice guidelines for the management of COPD do not have consistent recommendations for optimal physical activity, sedentary behaviour or sleep. While all guidelines recommend referral to PR, there is inconsistency amongst guidelines for effective strategies to optimize physical activity, and no guideline provides strategies to optimize sedentary behaviour or sleep. It is difficult to include detailed information on all aspects of COPD management in a single management guideline; however, optimizing use of time should be an important focus given the associated health benefits.

Status

CONFERENCE ABSTRACT

Institution

(Lewthwaite, Olds, Williams) Alliance for Research In Exercise, Nutrition and Activity, School of Health Sciences, University of South Australia, SA, Australia (Effing) Department of Respiratory Medicine, Southern Adelaide Local Health Network, SA, Australia

(Effing) School of Medicine, Flinders University, SA, Australia

Publisher

Blackwell Publishing

Year of Publication

2016

Link to the Ovid Full Text or citation:

[Click here for full text options](https://ovidsp.ovid.com/ovidweb.cgi?T=JS&CSC=Y&NEWS=N&PAGE=fulltext&D=emed17&AN=72316588)

Link to the External Link Resolver:

[LibKey NHS](https://libkey.io/libraries/2789/openurl?genre=article&sid=OVID:emed17&genre=article&id=pmid:&id=doi:10.1111%2Fresp.12754&issn=1440-1843&volume=21&issue=SUPPL.+2&spage=61&pages=61&date=2016&title=Respirology&atitle=Limited+recommendations+within+copd+clinical+practice+guidelines+concerning+optimising+physical+activity%2C+sedentary+behaviour+and+sleep%3A+A+systematic+review&aulast=Lewthwaite)

130.

Sleep quality in patients with chronic obstructive pulmonary disease referred for pulmonary rehabilitation.

Roberts M., Cho J., Wheatley J.

Embase

Respirology. Conference: 2016 Annual Scientific Meetings of the Thoracic Society of Australia and New Zealand and the Australian and New Zealand Society of Respiratory Science, TSANZSRS 2016. Perth, WA Australia. Conference Publication: (var.pagings). 21(SUPPL. 2) (pp 60), 2016. Date of Publication: April 2016.

[Conference Abstract]

AN: 72316587

Introduction: Chronic obstructive pulmonary disease (COPD) is a common chronic lung disease with both daytime and nighttime symptoms such as cough and breathlessness and is associated with poor quality of life. In addition, patients with COPD often report poor sleep quality regardless of the presence or absence of underlying sleep disorders.

Aim(s): Our aimis to determine (i) the prevalence of poor sleep quality (Pittsburgh Sleep Quality Index (PSQI) >= 5 a.u.) in patients with COPD referred for pulmonary rehabilitation (PR) and (ii) the factors associated with poor sleep quality.

Method(s):We had a retrospective chart review of COPD patients assessed for PR in Western Sydney over an 18-month period. Associations between sleep quality (PSQI) and continuous physiological and symptom-based COPD variables were made using Pearson's correlations. Categorical COPD variables were examined using one-way analysis of variance. Data were expressed as mean +/- SD, and p < 0.05 was significant.

Result(s): Sleep quality data were available for 337 patients: 51%male; age 69.5 +/- 9.2 years; FEV1 46 +/- 17% predicted; body mass index 27.7 +/- 7.7 kg/m2; Epworth sleepiness scale 5.9 +/- 4.5 a.u. Poor sleep quality was experienced by 267 patients (80%; PSQI 8.5 +/- 4.5 a.u.). Univariate analyses showed positive correlations between PSQI and St George RespiratoryQuestionnaire (r = 0.35, p < 0.001), Hospital Anxiety and Depression Scale (r = 0.40, p < 0.001 for anxiety; r = 0.28, p < 0.001 for depression), modified Medical Research Council dyspnoea scale (r = 0.24, p < 0.001) and COPD Assessment Test (r = 0.36, p < 0.001). There were weaker negative correlations between sleep quality and age (r = -0.13, p < 0.02).

Conclusion(s): In a PR program, there was a high prevalence of non-sleepy COPD patients with poor sleep quality. Factors which were associated with poor sleep quality include anxiety, depression, poorer quality of life, breathlessness, reduced exercise tolerance and COPD symptoms, but not measures of COPD severity or lung function. Further studies are required to explore causal links between COPD and poor sleep quality.

Status

CONFERENCE ABSTRACT

Institution

(Roberts, Cho, Wheatley) Westmead Hospital, Australia (Cho, Wheatley) Ludwig Engel Centre for Respiratory Research, Westmead Millennium Institute, Australia

(Cho, Wheatley) University of Sydney at Westmead Hospital, Australia

Publisher

Blackwell Publishing

Year of Publication

2016

Link to the Ovid Full Text or citation:

[Click here for full text options](https://ovidsp.ovid.com/ovidweb.cgi?T=JS&CSC=Y&NEWS=N&PAGE=fulltext&D=emed17&AN=72316587)

Link to the External Link Resolver:

[LibKey NHS](https://libkey.io/libraries/2789/openurl?genre=article&sid=OVID:emed17&genre=article&id=pmid:&id=doi:10.1111%2Fresp.12754&issn=1440-1843&volume=21&issue=SUPPL.+2&spage=60&pages=60&date=2016&title=Respirology&atitle=Sleep+quality+in+patients+with+chronic+obstructive+pulmonary+disease+referred+for+pulmonary+rehabilitation&aulast=Roberts)

131.

Clinical management of chronic obstructive pulmonary disease patients with muscle dysfunction.

Gea J., Casadevall C., Pascual S., Mauricio Orozco-Levi, Barreiro E.

Embase

Journal of Thoracic Disease. 8(11) (pp 3379-3400), 2016. Date of Publication: 2016.

[Review]

AN: 614204085

Muscle dysfunction is frequently observed in chronic obstructive pulmonary disease (COPD) patients, contributing to their exercise limitation and a worsening prognosis. The main factor leading to limb muscle dysfunction is deconditioning, whereas respiratory muscle dysfunction is mostly the result of pulmonary hyperinflation. However, both limb and respiratory muscles are also influenced by other negative factors, including smoking, systemic inflammation, nutritional abnormalities, exacerbations and some drugs. Limb muscle weakness is generally diagnosed through voluntary isometric maneuvers such as handgrip or quadriceps muscle contraction (dynamometry); while respiratory muscle loss of strength is usually recognized through a decrease in maximal static pressures measured at the mouth. Both types of measurements have validated reference values. Respiratory muscle strength can also be evaluated determining esophageal, gastric and transdiaphragmatic maximal pressures although there is a lack of widely accepted reference equations. Non-volitional maneuvers, obtained through electrical or magnetic stimulation, can be employed in patients unable to cooperate. Muscle endurance can also be assessed, generally using repeated submaximal maneuvers until exhaustion, but no validated reference values are available yet. The treatment of muscle dysfunction is multidimensional and includes improvement in lifestyle habits (smoking abstinence, healthy diet and a good level of physical activity, preferably outside), nutritional measures (diet supplements and occasionally, anabolic drugs), and different modalities of general and muscle training.

Copyright © Journal of Thoracic Disease. All rights reserved.

Status

Embase

Institution

(Gea, Casadevall, Pascual, Barreiro) Servei de Pneumologia, Hospital del Mar - IMIM, Experimental Sciences and Health Department (DCEXS), Universitat Pompeu Fabra, CIBERES, ISC III, Barcelona, Catalonia, Spain (Mauricio Orozco-Levi) Department of Respiratory, Cardiovascular Foundation from Colombia Floridablanca, Santander, Colombia

(Mauricio Orozco-Levi) CIBERES, ISC III, Barcelona, Catalonia, Spain

Publisher

AME Publishing Company (E-mail: jtd@thepbpc.org)

Year of Publication

2016

Link to the Ovid Full Text or citation:

[Click here for full text options](https://ovidsp.ovid.com/ovidweb.cgi?T=JS&CSC=Y&NEWS=N&PAGE=fulltext&D=emed17&AN=614204085)

Link to the External Link Resolver:

[LibKey NHS](https://libkey.io/libraries/2789/openurl?genre=article&sid=OVID:emed17&genre=article&id=pmid:&id=doi:10.21037%2Fjtd.2016.11.105&issn=2072-1439&volume=8&issue=11&spage=3379&pages=3379-3400&date=2016&title=Journal+of+Thoracic+Disease&atitle=Clinical+management+of+chronic+obstructive+pulmonary+disease+patients+with+muscle+dysfunction&aulast=Gea)

132.

Sleep-disordered breathing in patients with chronic obstructive pulmonary disease.

Sankari A., Badr M.S.

Embase

Annals of the American Thoracic Society. 12(9) (pp 1419-1420), 2015. Date of Publication: 01 Sep 2015.

[Letter]

AN: 606145682

PMID

26372809 [<https://www.ncbi.nlm.nih.gov/pubmed/?term=26372809>]

Status

Embase

Institution

(Sankari, Badr) John D. Dingell VA Medical Center, Detroit, MI, United States (Sankari, Badr) Wayne State University, Detroit, MI, United States

Publisher

American Thoracic Society (E-mail: malexander@thoracic.org)

Year of Publication

2015

Link to the Ovid Full Text or citation:

[Click here for full text options](https://ovidsp.ovid.com/ovidweb.cgi?T=JS&CSC=Y&NEWS=N&PAGE=fulltext&D=emed16&AN=606145682)

Link to the External Link Resolver:

[LibKey NHS](https://libkey.io/libraries/2789/openurl?genre=article&sid=OVID:emed16&genre=article&id=pmid:26372809&id=doi:10.1513%2FAnnalsATS.201506-313LE&issn=2325-6621&volume=12&issue=9&spage=1419&pages=1419-1420&date=2015&title=Annals+of+the+American+Thoracic+Society&atitle=Sleep-disordered+breathing+in+patients+with+chronic+obstructive+pulmonary+disease&aulast=Sankari)

133.

Improving outcomes in chronic obstructive pulmonary disease: The role of the interprofessional approach.

Amalakuhan B., Adams S.G.

Embase

International Journal of COPD. 10 (pp 1225-1232), 2015. Date of Publication: 26 Jun 2015.

[Review]

AN: 605123957

Chronic obstructive pulmonary disease (COPD) is associated with significant morbidity, places substantial time and cost burden on the health care system, and is now the third leading cause of death in the US. Many interventions are available to appropriately manage patients with COPD; however, fully implementing these strategies to help improve outcomes may be difficult. Collaboration between an interprofessional team of health care professionals (which includes physicians, nurses, respiratory therapists, physical therapists, dietitians, pharmacists, and many others) and COPD patients and caregivers is necessary to optimally manage these patients and to truly impact outcomes in this devastating disease. Prescribing evidence-based non-pharmacological and pharmacological therapies is an important start, but a true team-based approach is critical to successfully implement comprehensive care in patients with COPD. The goal of this review is to employ a case-based approach to provide practical information regarding the roles of the interprofessional team in implementing strategies to optimally manage COPD patients.

Copyright © 2015 Amalakuhan and Adams.

PMID

26170651 [<https://www.ncbi.nlm.nih.gov/pubmed/?term=26170651>]

Status

Embase

Institution

(Amalakuhan, Adams) Department of Medicine, Division of Pulmonary Diseases/Critical Care Medicine, The University of Texas Health Science Center at San Antonio, San Antonio, TX, United States (Amalakuhan, Adams) South Texas Veterans Health Care System, Audie L. Murphy Division, San Antonio, TX, United States

Publisher

Dove Medical Press Ltd. (PO Box 300-008, Albany, Auckland, New Zealand)

Year of Publication

2015

Link to the Ovid Full Text or citation:

[Click here for full text options](https://ovidsp.ovid.com/ovidweb.cgi?T=JS&CSC=Y&NEWS=N&PAGE=fulltext&D=emed16&AN=605123957)

Link to the External Link Resolver:

[LibKey NHS](https://libkey.io/libraries/2789/openurl?genre=article&sid=OVID:emed16&genre=article&id=pmid:26170651&id=doi:10.2147%2FCOPD.S71450&issn=1176-9106&volume=10&issue=&spage=1225&pages=1225-1232&date=2015&title=International+Journal+of+COPD&atitle=Improving+outcomes+in+chronic+obstructive+pulmonary+disease%3A+The+role+of+the+interprofessional+approach&aulast=Amalakuhan)

134.

High prevalence of obstructive sleep apnea in patients with moderate to severe chronic obstructive pulmonary disease.

Soler X., Gaio E., Powell F.L., Ramsdell J.W., Loredo J.S., Malhotra A., Ries A.L.

Embase

Annals of the American Thoracic Society. 12(8) (pp 1219-1225), 2015. Date of Publication: 01 Aug 2015.

[Article]

AN: 606144111

Rationale: When obstructive sleep apnea (OSA) and chronic obstructive pulmonary disease (COPD) coexist in the so-called "overlap" syndrome,a high risk formortalityandmorbidity hasbeenreported.There is controversy about the prevalence of OSA in people affected by COPD.

Objective(s): The purpose of this study was to investigate objective meaures of sleep-disordered breathing in patients with moderate to severe COPD to test the hypothesis that COPD is associated with an increased prevalence of OSA.

Method(s): Fifty-four patients (54% men) with moderate to severe COPD were enrolled prospectively (mean+/-SD, FEV1 = 42.8+/- 19.8% predicted, and FEV1/FVC = 42.3+/-13.1). Twenty patients (37%) were on supplemental oxygen at baseline. Exercise tolerance; questionnaires related to symptoms, sleep, and quality of life; and home polysomnography were obtained.

Measurements and Main Results: Forty-four patients had full polysomnography suitable for analysis. OSA (apnea-hypopnea index>5/h) was present in 29 subjects (65.9%). Sleep efficiency was poor in 45% of subjects.

Conclusion(s): OSA is highly prevalent in patients with moderate to severe COPD referred to pulmonary rehabilitation. Sleep quality is also poor among this selected group. These patients have greaterthan- expected sleep-disordered breathing, which could be an important contributory factor to morbidity and mortality. Pulmonary rehabilitation programs should consider including a sleep assessment in patients with moderate to severe COPD and interventions when indicated to help reduce the impact of OSA in COPD.

Copyright © 2015 by the American Thoracic Society.

PMID

25871443 [<https://www.ncbi.nlm.nih.gov/pubmed/?term=25871443>]

Status

Embase

Institution

(Soler, Loredo, Malhotra, Ries) Pulmonary, Critical Care, and Sleep Division, Department of Medicine, University of California, San Diego, 200 West Arbor Drive, M/C 8377, San Diego, CA 92103-8377, United States (Gaio) Pulmonary Division, University of Brasilia, Brasilia, Brazil

(Gaio, Powell) Physiology Division, Department of Medicine, University of California, San Diego, San Diego, CA, United States

(Ramsdell) General Internal Medicine Division, Department of Medicine, University of California, San Diego, San Diego, CA, United States

Publisher

American Thoracic Society (E-mail: malexander@thoracic.org)

Year of Publication

2015

Link to the Ovid Full Text or citation:

[Click here for full text options](https://ovidsp.ovid.com/ovidweb.cgi?T=JS&CSC=Y&NEWS=N&PAGE=fulltext&D=emed16&AN=606144111)

Link to the External Link Resolver:

[LibKey NHS](https://libkey.io/libraries/2789/openurl?genre=article&sid=OVID:emed16&genre=article&id=pmid:25871443&id=doi:10.1513%2FAnnalsATS.201407-336OC&issn=2325-6621&volume=12&issue=8&spage=1219&pages=1219-1225&date=2015&title=Annals+of+the+American+Thoracic+Society&atitle=High+prevalence+of+obstructive+sleep+apnea+in+patients+with+moderate+to+severe+chronic+obstructive+pulmonary+disease&aulast=Soler)

135.

Zopiclone effects on breathing at sleep in stable chronic obstructive pulmonary disease.

Holmedahl N.H., Overland B., Fondenes O., Ellingsen I., Hardie J.A.

Embase

Sleep and Breathing. 19(3) (pp 921-930), 2015. Date of Publication: 13 Dec 2014.

[Article]

AN: 600781568

Purpose: More than half of patients with chronic obstructive pulmonary disease (COPD) experiences sleep-related problems and about one fourth uses hypnotics regularly. We explored what the effect zopiclone, a commonly used hypnotic, had on nocturnal gas exchange and the apnea/hypopnea frequency in stable COPD.

Method(s): Randomized crossover study of 31 (ten males) inpatients at a pulmonary rehabilitation hospital, median age 64 years, of which 20 had a forced expiratory volume first second <50 % of predicted. Subjects investigated in randomized order of either baseline sleep or intervention with 5 mg zopiclone by polysomnography including transcutaneous measurement of carbon dioxide pressure increased (DELTAPtcCO2).

Result(s): Zopiclone increased the mean DELTAPtcCO2 from baseline both in rapid eye movement (REM) sleep, non-REM sleep, and even in stage N0 (awake after sleep onset) with a mean (SD) of 0.25 (0.40) kPa, 0.22 (0.32) kPa, and 0.14 (0.27) kPa, respectively. Subjects with sleep hypoventilation as defined by the American Academy of Sleep Medicine increased from 6 subjects (19 %) to 13 subjects (42 %) (P = 0.020). REM sleep minimum oxygen saturation (minSpO2) did not change significantly from baseline median (interquartile range [IQR]) minSpO2 81.8 (12.1) % to zopiclone sleep median (IQR) minSpO2 80.0 (12.0) % (P = 0.766). Interestingly, zopiclone reduced the number of apneas/hypopneas per hour (AHI) in subjects with overlap (AHI >= 15) with a median difference (IQR) of -8.5 (7.8) (N = 11, P = 0.016).

Conclusion(s): In stable COPD, zopiclone moderately increases the mean DELTAPtcCO2 without changing minSpO2 at night and reduces AHI in overlap (COPD and obstructive sleep apnea) subjects.

Copyright © 2014, Springer-Verlag Berlin Heidelberg.

PMID

25501294 [<https://www.ncbi.nlm.nih.gov/pubmed/?term=25501294>]

Status

Embase

Institution

(Holmedahl, Ellingsen) LHL-klinikkene Glittre, Glittreklinikken, postboks 104 Aneby, Hakadal 1485, Norway (Overland) ENT Department, Lovisenberg Diakonale Hospital, Oslo, Norway

(Fondenes) Norwegian National Centre of Excellence in Home Mechanical Ventilation, Haukeland University Hospital, Bergen, Norway

(Holmedahl, Hardie) Department of Clinical Science, University of Bergen, Bergen, Norway

Publisher

Springer Verlag (E-mail: service@springer.de)

Year of Publication

2015

Link to the Ovid Full Text or citation:

[Click here for full text options](https://ovidsp.ovid.com/ovidweb.cgi?T=JS&CSC=Y&NEWS=N&PAGE=fulltext&D=emed16&AN=600781568)

Link to the External Link Resolver:

[LibKey NHS](https://libkey.io/libraries/2789/openurl?genre=article&sid=OVID:emed16&genre=article&id=pmid:25501294&id=doi:10.1007%2Fs11325-014-1084-8&issn=1520-9512&volume=19&issue=3&spage=921&pages=921-930&date=2015&title=Sleep+and+Breathing&atitle=Zopiclone+effects+on+breathing+at+sleep+in+stable+chronic+obstructive+pulmonary+disease&aulast=Holmedahl)

136.

Advances in the treatment of idiopathic pulmonary fibrosis.

Sergew A., Brown K.K.

Embase

Expert Opinion on Emerging Drugs. 20(4) (pp 537-552), 2015. Date of Publication: 02 Oct 2015.

[Review]

AN: 607135759

Introduction: Idiopathic pulmonary fibrosis (IPF) is a lung limited, progressive fibrotic disease with a poor prognosis. The cause is unknown, and currently there is no treatment that reverses the disease or stops progression. This combination of a poor prognosis and the absence of curative therapy has prompted a sustained investigative effort to identify beneficial treatments. Recently released trial results suggest progress.Areas covered: Although the mechanism of disease is poorly understood, a number of compounds that influence pathways thought to play a mechanistic role have been studied for use in IPF. This article discusses a number of these landmark trials.Expert opinion: From these studies we conclude that the future treatment of IPF will include expanding pharmacological options. Recent studies have identified two agents that appear to slow disease progression and may offer a window into pathogenesis and future drug targets.

Copyright © 2015 Taylor & Francis.

PMID

26629731 [<https://www.ncbi.nlm.nih.gov/pubmed/?term=26629731>]

Status

Embase

Institution

(Sergew, Brown) Department of Medicine, National Jewish Health, 1400 Jackson St, M336, Denver, CO 80206, United States

Publisher

Taylor and Francis Ltd (E-mail: healthcare.enquiries@informa.com)

Clinical Trial Number

<https://clinicaltrials.gov/show/NCT00063869>

Year of Publication

2015

Link to the Ovid Full Text or citation:

[Click here for full text options](https://ovidsp.ovid.com/ovidweb.cgi?T=JS&CSC=Y&NEWS=N&PAGE=fulltext&D=emed16&AN=607135759)

Link to the External Link Resolver:

[LibKey NHS](https://libkey.io/libraries/2789/openurl?genre=article&sid=OVID:emed16&genre=article&id=pmid:26629731&id=doi:10.1517%2F14728214.2015.1102886&issn=1472-8214&volume=20&issue=4&spage=537&pages=537-552&date=2015&title=Expert+Opinion+on+Emerging+Drugs&atitle=Advances+in+the+treatment+of+idiopathic+pulmonary+fibrosis&aulast=Sergew)

137.

Hot topics in noninvasive ventilation: Report of a working group at the international symposium on sleep-disordered breathing in Leuven, Belgium.

Vrijsen B., Chatwin M., Contal O., Derom E., Janssens J.-P., Kampelmacher M.J., Muir J.-F., Pinto S., Rabec C., Ramsay M., Randerath W.J., Storre J.H., Wijkstra P.J., Windisch W., Testelmans D.

Embase

Respiratory Care. 60(9) (pp 1337-1362), 2015. Date of Publication: 01 Sep 2015.

[Article]

AN: 606574348

During the last few decades, attention has increasingly focused on noninvasive ventilation (NIV) in the treatment of chronic respiratory failure. The University of Leuven and the University Hospitals Leuven therefore chose this topic for a 2-day working group session during their International Symposium on Sleep-Disordered Breathing. Numerous European experts took part in this session and discussed (1) NIV in amyotrophic lateral sclerosis (when to start NIV, NIV and sleep, secretion management, and what to do when NIV fails), (2) recent insights in NIV and COPD (high-intensity NIV, NIV in addition to exercise training, and NIV during exercise training), (3) monitoring of NIV (monitoring devices, built-in ventilator software, leaks, and asynchronies) and identifying events during NIV; and (4) recent and future developments in NIV (target-volume NIV, electromyography- triggered NIV, and autoregulating algorithms).

Copyright © 2015 Daedalus Enterprises.

PMID

26130816 [<https://www.ncbi.nlm.nih.gov/pubmed/?term=26130816>]

Status

Embase

Institution

(Vrijsen, Testelmans) Department of Respiratory Diseases, University Hospitals Leuven, Katholieke Universiteit Leuven/University of Leuven, Leuven, Belgium (Chatwin) Sleep and Ventilation Unit, Royal Brompton Hospital, London, United Kingdom

(Contal, Janssens) Division of Pulmonary Diseases, Geneva University Hospitals, Geneva, Switzerland

(Derom) Respiratory Medicine, Ghent University Hospital, Ghent, Belgium

(Kampelmacher) HomeVentilation Service, University Medical Center Utrecht, Utrecht, Netherlands

(Muir) Service de Pneumologie et Unite de Soins Intensifs Respiratoires, Centre Hospitalier Universitaire de Rouen, Rouen, France

(Pinto) Institute of Molecular Medicine, Translational Clinical Physiology Unit, University of Lisbon, Lisbon, Portugal

(Rabec) Pneumologie et Reanimation Respiratoire, Centre Hospitalier Universitaire de Dijon, Dijon, France

(Ramsay) Division of Asthma, Allergy and Lung Biology, King's College London, London, United Kingdom

(Randerath) Pneumologie an der Universitat Witten/Herdecke, Klinik fur Pneumologie und Allergologie, and Zentrum fur Schlaf- und Beatmungsmedizin, Krankenhaus Bethanien, Solingen, Germany

(Storre, Wijkstra) Kliniken der Stadt Koln gGmbH, Witten/ Herdecke University Hospital, Department of Pneumology, Cologne- Merheim Hospital, Cologne, Germany

(Windisch) Center for Home Mechanical Ventilation, University of Groningen, and Department of Pulmonology, Groningen University Medical Center Groningen, Groningen, Netherlands

Publisher

American Association for Respiratory Care

Year of Publication

2015

Link to the Ovid Full Text or citation:

[Click here for full text options](https://ovidsp.ovid.com/ovidweb.cgi?T=JS&CSC=Y&NEWS=N&PAGE=fulltext&D=emed16&AN=606574348)

Link to the External Link Resolver:

[LibKey NHS](https://libkey.io/libraries/2789/openurl?genre=article&sid=OVID:emed16&genre=article&id=pmid:26130816&id=doi:10.4187%2Frespcare.03796&issn=0020-1324&volume=60&issue=9&spage=1337&pages=1337-1362&date=2015&title=Respiratory+Care&atitle=Hot+topics+in+noninvasive+ventilation%3A+Report+of+a+working+group+at+the+international+symposium+on+sleep-disordered+breathing+in+Leuven%2C+Belgium&aulast=Vrijsen)

138.

The Role of Noninvasive Ventilation in the Hospital and Outpatient Management of Chronic Obstructive Pulmonary Disease.

Hoo G.W.S.

Embase

Seminars in Respiratory and Critical Care Medicine. 36(4) (pp 616-629), 2015. Article Number: 01139. Date of Publication: 05 Aug 2015.

[Article]

AN: 605454820

Positive pressure noninvasive ventilation (NIV) has become widely accepted in the treatment of both hospitalized and outpatient subjects with chronic obstructive pulmonary disease (COPD). The support has evolved over the past two decades to be part of first-line management in acute exacerbations of COPD and is also instrumental in discontinuing mechanical ventilation in COPD patients with acute respiratory failure. It is also suitable for treatment of COPD with other associated conditions including pneumonia, following lung resectional surgery, with concomitant obstructive sleep apnea and as part of end-of-life care. Short-term application can also facilitate some endoscopic procedures that may otherwise require endotracheal intubation. Outpatient use of NIV in COPD has garnered much attention, but the support has not been as robust as with NIV in hospitalized patients. However, an approach with higher pressures with a goal of significant reduction in daytime PaCO2 may be an effective strategy. NIV can also facilitate exercise training in pulmonary rehabilitation. A portable device which can augment tidal volume during ambulation and other activities of daily living may further expand the use of NIV in COPD patients.

Copyright © 2015 by Thieme Medical Publishers, Inc.

PMID

26238646 [<https://www.ncbi.nlm.nih.gov/pubmed/?term=26238646>]

Status

Embase

Institution

(Hoo) Pulmonary and Critical Care Section (111Q), West Los Angeles VA Healthcare Center, VA Greater Los Angeles Healthcare System, Geffen School of Medicine at UCLA, 11301 Wilshire Blvd., Los Angeles, CA 90073, United States

Publisher

Thieme Medical Publishers, Inc. (E-mail: custserv@thieme.com)

Year of Publication

2015

Link to the Ovid Full Text or citation:

[Click here for full text options](https://ovidsp.ovid.com/ovidweb.cgi?T=JS&CSC=Y&NEWS=N&PAGE=fulltext&D=emed16&AN=605454820)

Link to the External Link Resolver:

[LibKey NHS](https://libkey.io/libraries/2789/openurl?genre=article&sid=OVID:emed16&genre=article&id=pmid:26238646&id=doi:10.1055%2Fs-0035-1556074&issn=1069-3424&volume=36&issue=4&spage=616&pages=616-629&date=2015&title=Seminars+in+Respiratory+and+Critical+Care+Medicine&atitle=The+Role+of+Noninvasive+Ventilation+in+the+Hospital+and+Outpatient+Management+of+Chronic+Obstructive+Pulmonary+Disease&aulast=Hoo)

139.

Reply: Sleep-disordered breathing in patients with chronic obstructive pulmonary disease.

Soler X., Malhotra A.

Embase

Annals of the American Thoracic Society. 12(9) (pp 1420-1421), 2015. Date of Publication: 01 Sep 2015.

[Letter]

AN: 606145689

PMID

26372810 [<https://www.ncbi.nlm.nih.gov/pubmed/?term=26372810>]

Status

Embase

Institution

(Soler, Malhotra) University of California, San Diego, San Diego, CA, United States

Publisher

American Thoracic Society (E-mail: malexander@thoracic.org)

Year of Publication

2015

Link to the Ovid Full Text or citation:

[Click here for full text options](https://ovidsp.ovid.com/ovidweb.cgi?T=JS&CSC=Y&NEWS=N&PAGE=fulltext&D=emed16&AN=606145689)

Link to the External Link Resolver:

[LibKey NHS](https://libkey.io/libraries/2789/openurl?genre=article&sid=OVID:emed16&genre=article&id=pmid:26372810&id=doi:10.1513%2FAnnalsATS.201506-379LE&issn=2325-6621&volume=12&issue=9&spage=1420&pages=1420-1421&date=2015&title=Annals+of+the+American+Thoracic+Society&atitle=Reply%3A+Sleep-disordered+breathing+in+patients+with+chronic+obstructive+pulmonary+disease&aulast=Soler)

140.

Managing comorbidities in COPD.

Hillas G., Perlikos F., Tsiligianni I., Tzanakis N.

Embase

International Journal of COPD. 10 (pp 95-109), 2015. Date of Publication: 07 Jan 2015.

[Review]

AN: 601241173

Chronic obstructive pulmonary disease (COPD) is a leading cause of morbidity and mortality worldwide. Age and smoking are common risk factors for COPD and other illnesses, often leading COPD patients to demonstrate multiple coexisting comorbidities. COPD exacerbations and comorbidities contribute to the overall severity in individual patients. Clinical trials investigating the treatment of COPD routinely exclude patients with multiple comorbidities or advanced age. Clinical practice guidelines for a specific disease do not usually address comorbidities in their recommendations. However, the management and the medical intervention in COPD patients with comorbidities need a holistic approach that is not clearly established worldwide. This holistic approach should include the specific burden of each comorbidity in the COPD severity classification scale. Further, the pharmacological and nonpharmacological management should also include optimal interventions and risk factor modifications simultaneously for all diseases. All health care specialists in COPD management need to work together with professionals specialized in the management of the other major chronic diseases in order to provide a multidisciplinary approach to COPD patients with multiple diseases. In this review, we focus on the major comorbidities that affect COPD patients. We present an overview of the problems faced, the reasons and risk factors for the most commonly encountered comorbidities, and the burden on health care costs. We also provide a rationale for approaching the therapeutic options of the COPD patient afflicted by comorbidity.

Copyright © 2015 Hillas et al.

PMID

25609943 [<https://www.ncbi.nlm.nih.gov/pubmed/?term=25609943>]

Status

Embase

Institution

(Hillas, Perlikos) Department of Critical Care and Pulmonary Services, University of Athens Medical School, Evangelismos Hospital, Athens, Greece (Tsiligianni, Tzanakis) Department of Thoracic Medicine, University Hospital of Heraklion, Medical School, University of Crete, Crete, Greece

(Tsiligianni) Department of General Practice, University Medical Centre of Groningen, Groningen, Netherlands

Publisher

Dove Medical Press Ltd. (PO Box 300-008, Albany, Auckland, New Zealand)

Year of Publication

2015

Link to the Ovid Full Text or citation:

[Click here for full text options](https://ovidsp.ovid.com/ovidweb.cgi?T=JS&CSC=Y&NEWS=N&PAGE=fulltext&D=emed16&AN=601241173)

Link to the External Link Resolver:

[LibKey NHS](https://libkey.io/libraries/2789/openurl?genre=article&sid=OVID:emed16&genre=article&id=pmid:25609943&id=doi:10.2147%2FCOPD.S54473&issn=1176-9106&volume=10&issue=&spage=95&pages=95-109&date=2015&title=International+Journal+of+COPD&atitle=Managing+comorbidities+in+COPD&aulast=Hillas)

141.

Treating COPD in older and oldest old patients.

Corsonello A., Scarlata S., Pedone C., Bustacchini S., Fusco S., Zito A., Incalzi R.A.

Embase

Current Pharmaceutical Design. 21(13) (pp 1672-1689), 2015. Date of Publication: 2015.

[Article]

AN: 604847818

The treatment of older and oldest old patients with COPD poses several problems and should be tailored to specific outcomes, such as physical functioning. Indeed, impaired homeostatic mechanisms, deteriorated physiological systems, and limited functional reserve mainly contribute to this complex scenario. Therefore, we reviewed the main difficulties in managing therapy for these patients and possible remedies. Inhaled long acting beta-agonists (LABA) and anticholinergics (LAMA) are the mainstay of therapy in stable COPD, but it should be considered that pharmacological response and safety profile may vary significantly in older patients with multimorbidity. Their association with inhaled corticosteroids is recommended only for patients with severe or very severe air-flow limitation or with frequent exacerbations despite bronchodilator treatment. In hypoxemic patients, long-term oxygen therapy (LTOT) may improve not only general comfort and exercise tolerance, but also cognitive functions and sleep. Non-pharmacological interventions, including education, physical exercise, nutritional support, pulmonary rehabilitation and telemonitoring can importantly contribute to improve outcomes. Older patients with COPD should be systematically evaluated for the presence of risk factors for non-adherence, and the inhaler device should be chosen very carefully. Comorbidities, such as cardiovascular diseases, chronic kidney disease, osteoporosis, obesity, cognitive, visual and auditory impairment, may significantly affect treatment choices and should be scrutinized. Palliative care is of paramount importance in end-stage COPD. Finally, treatment of COPD exacerbations has been also reviewed. Therapeutic decisions should be founded on a careful assessment of cognitive and functional status, comorbidity, polypharmacy, and age-related changes in pharmacokinetics and pharmacodynamics in order to minimize adverse drug events, drug-drug or drug-disease interactions, and non-adherence to treatment.

Copyright © 2015 Bentham Science Publishers.

PMID

25633118 [<https://www.ncbi.nlm.nih.gov/pubmed/?term=25633118>]

Status

Embase

Institution

(Corsonello, Fusco) Unit of Geriatric Pharmacoepidemiology, Italian National Research Center on Aging (INRCA), Cosenza, Italy (Scarlata, Pedone, Zito, Incalzi) Chair of Geriatric Medicine, University Campus Biomedico, Rome, Italy

(Bustacchini) Scientific Direction, Italian National Research Center on Aging (INRCA), Cosenza, Italy

Publisher

Bentham Science Publishers B.V. (P.O. Box 294, Bussum 1400 AG, Netherlands)

Year of Publication

2015

Link to the Ovid Full Text or citation:

[Click here for full text options](https://ovidsp.ovid.com/ovidweb.cgi?T=JS&CSC=Y&NEWS=N&PAGE=fulltext&D=emed16&AN=604847818)

Link to the External Link Resolver:

[LibKey NHS](https://libkey.io/libraries/2789/openurl?genre=article&sid=OVID:emed16&genre=article&id=pmid:25633118&id=doi:10.2174%2F1381612821666150130121229&issn=1381-6128&volume=21&issue=13&spage=1672&pages=1672-1689&date=2015&title=Current+Pharmaceutical+Design&atitle=Treating+COPD+in+older+and+oldest+old+patients&aulast=Corsonello)

142.

Non-pharmacological management of chronic obstructive pulmonary disease.

Safka K.A., Andrew McIvor R.

Embase

Ulster Medical Journal. 84(1) (pp 13-21), 2015. Date of Publication: 01 Jan 2015.

[Review]

AN: 602082460

Status

Embase

Institution

(Safka, Andrew McIvor) McMaster University, Canada

Publisher

Ulster Medical Society

Year of Publication

2015

Link to the Ovid Full Text or citation:

[Click here for full text options](https://ovidsp.ovid.com/ovidweb.cgi?T=JS&CSC=Y&NEWS=N&PAGE=fulltext&D=emed16&AN=602082460)

Link to the External Link Resolver:

[LibKey NHS](https://libkey.io/libraries/2789/openurl?genre=article&sid=OVID:emed16&genre=article&id=pmid:&id=doi:&issn=0041-6193&volume=84&issue=1&spage=13&pages=13-21&date=2015&title=Ulster+Medical+Journal&atitle=Non-pharmacological+management+of+chronic+obstructive+pulmonary+disease&aulast=Safka)

143.

Prevention of Postoperative Pulmonary Complications.

Taylor A., DeBoard Z., Gauvin J.M.

Embase

Surgical Clinics of North America. 95(2) (pp 237-254), 2015. Date of Publication: 01 Apr 2015.

[Review]

AN: 603282639

Postoperative pulmonary complications (PPCs) occur frequently among general surgical patients. The spectrum of illness is broad and includes preventable causes of morbidity and death. Careful preoperative evaluation can identify undiagnosed and undertreated illness and allow for preoperative intervention. Optimization of patient, surgical, and anesthetic factors is crucial in the prevention of PPCs.

Copyright © 2015 Elsevier Inc.

PMID

25814104 [<https://www.ncbi.nlm.nih.gov/pubmed/?term=25814104>]

Status

Embase

Institution

(Taylor, DeBoard, Gauvin) Surgical Education Department, Santa Barbara Cottage Hospital, 400 West Pueblo Street, Santa Barbara, CA 93105, United States

Publisher

W.B. Saunders

Year of Publication

2015

Link to the Ovid Full Text or citation:

[Click here for full text options](https://ovidsp.ovid.com/ovidweb.cgi?T=JS&CSC=Y&NEWS=N&PAGE=fulltext&D=emed16&AN=603282639)

Link to the External Link Resolver:

[LibKey NHS](https://libkey.io/libraries/2789/openurl?genre=article&sid=OVID:emed16&genre=article&id=pmid:25814104&id=doi:10.1016%2Fj.suc.2014.11.002&issn=0039-6109&volume=95&issue=2&spage=237&pages=237-254&date=2015&title=Surgical+Clinics+of+North+America&atitle=Prevention+of+Postoperative+Pulmonary+Complications&aulast=Taylor)

144.

Predictors of obstructive sleep apnea in patients with chronic obstructive pulmonary disease.

Liao S.-Y., Gaio E., Loredo J.S., Ries A.L., Malhotra A., Soler X.

Embase

American Journal of Respiratory and Critical Care Medicine. Conference: American Thoracic Society International Conference, ATS 2015. Denver, CO United States. Conference Publication: (var.pagings). 191(MeetingAbstracts) (no pagination), 2015. Date of Publication: 2015.

[Conference Abstract]

AN: 72053604

Background: The association of chronic obstructive pulmonary disease (COPD) and obstructive sleep apnea (OSA), the so called overlap syndrome, leads to severe nocturnal desaturation and high morbidity and mortality. In previous work we found poor sleep quality and high prevalence of OSA in patients with moderate to severe COPD. Based on those findings, we investigated traditional predictors (age, obesity, gender, neck circumference) and other potential predictors (dyspnea, quality of life, and sleepiness) of OSA with the hypothesis that predictors of OSA may be different in patients with moderate to severe COPD compared to the general population.

Method(s): Forty-four subjects (59% male) with moderate to severe COPD [mean FEV1=41.0+/-18.2(SD) %pred, FEV1/FVC=41.5+/-12.7(SD)%] enrolled prospectively in the UCSD pulmonary rehabilitation (PR) Program completed both full-unattended polysomnogram (PSG) and outcome measures to evaluate dyspnea [UCSD Shortness of Breath Questionnaire (SOBQ)], quality of life [St. George's Respiratory Questionnaire (SGRQ)], sleep [Epworth Sleepiness Scale (ESS) and Insomnia Severity Index (ISI)]. Diagnosis and severity of OSA was based on the Apnea-Hypopnea Index (AHI>=5/h). We evaluated the sensitivity and specificity of male gender, obesity (BMI>=30kg/m2), large neck circumference (male >=41cm, female >=38cm), and Epworth Sleepiness Scale (ESS) >10/24 as predictors of OSA. Receiver Operating Characteristics (ROC) analysis was used to evaluate the effectiveness of the continuous scoring questionnaires including SOBQ, SGRQ, ESS and ISI to predict OSA. Odds ratios were calculated with univariate logistic regression analysis.

Result(s): Twenty-nine subjects (66%) had OSA. Nine subjects (20%) had severe OSA (AHI>=30/h). Eighteen patients were on supplemental oxygen. Male gender had the highest sensitivity (0.62) in detecting OSA, while large neck circumference, obesity, and ESS>10 had low sensitivity (0.29, 0.31, and 0.34, respectively) but with high specificity (0.79, 0.80, 0.80, respectively). For ROC analysis, ESS demonstrated moderate performance as a predictor of OSA with area under curve (AUC) of 0.67. SGRQ, SOBQ, and ISI had low performance with AUC of 0.51, 0.56, and 0.43, respectively. Univariate logistic regression analysis found that obesity (OR 5.0, p=0.04) and large neck circumference (OR 5.6, p=0.03) were predictors of severe OSA (AHI>=30/h) only.

Conclusion(s): In a cohort of patients with moderate to severe COPD enrolled in PR, male gender had the highest sensitivity for the presence of OSA. Large neck circumference and obesity were independent predictors of severe OSA, while ESS, SOBQ, SGRQ and ISI were not. Differences in OSA predictors exist between patients with more advanced COPD and the general population.

Status

CONFERENCE ABSTRACT

Institution

(Liao) University of California, Riverside, CA, United States (Gaio) University of Brasilia, Brasilia, Brazil

(Loredo, Ries, Malhotra, Soler) University of California, San Diego, San Diego, CA, United States

Publisher

American Thoracic Society

Year of Publication

2015

Link to the Ovid Full Text or citation:

[Click here for full text options](https://ovidsp.ovid.com/ovidweb.cgi?T=JS&CSC=Y&NEWS=N&PAGE=fulltext&D=emed16&AN=72053604)

Link to the External Link Resolver:

[LibKey NHS](https://libkey.io/libraries/2789/openurl?genre=article&sid=OVID:emed16&genre=article&id=pmid:&id=doi:&issn=1073-449X&volume=191&issue=MeetingAbstracts&spage=&pages=&date=2015&title=American+Journal+of+Respiratory+and+Critical+Care+Medicine&atitle=Predictors+of+obstructive+sleep+apnea+in+patients+with+chronic+obstructive+pulmonary+disease&aulast=Liao)

145.

The effect of pulmonary rehabilitation on objective sleep measurements in patients with COPD.

Kittah J.K., Roberts S., Haggerty M., Weir I.

Embase

American Journal of Respiratory and Critical Care Medicine. Conference: American Thoracic Society International Conference, ATS 2015. Denver, CO United States. Conference Publication: (var.pagings). 191(MeetingAbstracts) (no pagination), 2015. Date of Publication: 2015.

[Conference Abstract]

AN: 72052911

Rationale: Poor sleep is common among subjects with chronic obstructive pulmonary disease (COPD) and most subjects with respiratory symptoms report sleep complaints and have objective measurements of sleep disturbance compared to controls. While pulmonary rehabilitation (PR) has a proven efficacy in improving dyspnea and fatigue, its specific effect on objective sleep measurements is unknown.

METHOD(S): We performed a prospective observational cohort study to examine the impact of PR on objective and subjective sleep characteristics in COPD patients. Participants had a FEV1/FVC ratio <0.7 with a clinical diagnosis of COPD and referred for PR between September 2012 and June 2014. Subjects were excluded if they had a known sleep disorder, unstable medical or mental health condition, or previous pulmonary rehabilitation within last 12 months of enrollment. Subjects completed baseline documentation including the chronic respiratory questionnaire (CRQ), insomnia severity index (ISI) scale, Berlin questionnaire, and Epworth Sleepiness Scale (ESS) score. Overall sleep quality was measured by Pittsburg sleep quality index (PSQI). Sleep onset latency (SOL), wake after sleep onset (WASO), sleep efficiency (SE), and total sleep time (TST) were measured by wrist actigraphy (Motionlogger, Ambulatory Monitoring, Inc. Ardsley, NY) and sleep logs for 7 days before PR. Subjects who successfully completed 9 weeks of PR had repeat measurements of CRQ, PSQI, ISI, ESS, and SOL, WASO, TST, SE by actigraphy. Primary outcome was sleep measurements by actigraphy and PSQI pre and post PR.

RESULT(S): Twenty six subjects were recruited and 9 did not complete PR and were excluded. Of the remaining 17 subjects, 53% had a high risk Berlin score; 29% had at least moderate insomnia with ISI>14; 65% were classified as poor sleepers with a PSQI>5; and 12% had ESS>10. After PR, there was no statistically significant improvements in SE (p=0.35), SOL (p=0.23), WASO (p=0.51), and TST (p=0.46) as measured by wrist actigraphy or PSQI (p=0.43). Minimally clinically important difference (MCID) improvements for CRQ were observed in 88% of subjects for dyspnea and 65% for fatigue.

CONCLUSION(S): PR is not associated with significant improvements in objective sleep characteristics measured by actigraphy despite most subjects having MCID improvements in CRQ. Additional sleep specific treatments that are added to PR may be necessary to improve sleep in these patients.

Status

CONFERENCE ABSTRACT

Institution

(Kittah, Roberts, Haggerty, Weir) Norwalk Hospital, Norwalk, CT, United States

Publisher

American Thoracic Society

Year of Publication

2015

Link to the Ovid Full Text or citation:

[Click here for full text options](https://ovidsp.ovid.com/ovidweb.cgi?T=JS&CSC=Y&NEWS=N&PAGE=fulltext&D=emed16&AN=72052911)

Link to the External Link Resolver:

[LibKey NHS](https://libkey.io/libraries/2789/openurl?genre=article&sid=OVID:emed16&genre=article&id=pmid:&id=doi:&issn=1073-449X&volume=191&issue=MeetingAbstracts&spage=&pages=&date=2015&title=American+Journal+of+Respiratory+and+Critical+Care+Medicine&atitle=The+effect+of+pulmonary+rehabilitation+on+objective+sleep+measurements+in+patients+with+COPD&aulast=Kittah)

146.

Effects of 1-year treatment with nocturnal continuous positive pressure ventilation (CPAP) in overlap syndrome.

Schreiber A., Surbone S., Malovini A., Mancini M., Tedeschi N., Piaggi G., Carlucci A.

Embase

American Journal of Respiratory and Critical Care Medicine. Conference: American Thoracic Society International Conference, ATS 2015. Denver, CO United States. Conference Publication: (var.pagings). 191(MeetingAbstracts) (no pagination), 2015. Date of Publication: 2015.

[Conference Abstract]

AN: 72050180

Rationale: In patients with chronic obstructive pulmonary disease (COPD), the coexistence of obstructive sleep apnea (OSA) (overlap syndrome) is associated with a heavy health burden and treatment with continuous positive pressure ventilation (CPAP) turned out to improve outcome.

Method(s): In a selected group of overlap patients admitted to our Pulmonary Rehabilitation Unit in the last three years, we evaluated the effects of CPAP therapy on respiratory function and arterial blood gases and, in case of amelioration, searched for predictors of improvement.

Result(s): Ninety-four overlap patients (76 males, median age 66 (60.374)) were enrolled. At the enrollment, a stepwise logistic regression analysis, showed that a lower forced expiratory volume in one second (FEV1) and a higher apnea-hypopnea index (AHI) at the baseline were independent predictors of hypercapnia. In a multivariate analysis, basal FEV1/VC ratio was an independent predictive factor for severity of oxygen desaturation during the night, expressed as percentage of time spent with saturation < 90%. After 1 year of treatment there was a significant improvement of PaO2 (median= 65, IQR = 59.05:70 vs. 71, IQR = 64.7:77.1, p < 1x10^-7^) and PaCO2 (median= 39.85, IQR= 36.18:43.55 vs. 38, IQR= 34:43, p< 0.001). FEV1 and vital capacity (VC) didn't show a significant change after 1 year (p > 0.05). This may be due to an underpowered sample (statistical power= 0.25 and 0.12, respectively). However we found that the improvement of FEV1 after 1 year was significantly and inversely correlated with the basal FEV1 expressed as % of reference value (FEV1%) (r= -0.36, p<0.05). In particular, we identified a threshold of basal FEV1%: patients with values < 69.6% showed a statistically significant improvement in FEV1 after 1 year of CPAP treatment. In this sub-group of patients the median improvement of FEV1 was 0.32 L with respect to the rest of the cohort (median=-0.25, IQR= -0.34:-0.08 vs. median= +0.07, IQR= -0.08:+0.22, p < 1x10^-7^).

Conclusion(s): In conclusion in overlap syndrome one year of CPAP treatment improves the FEV1 of a clinically relevant amount only in patients with moderate to severe airways obstruction.

Status

CONFERENCE ABSTRACT

Institution

(Schreiber, Malovini, Mancini, Piaggi, Carlucci) IRCCS Fondazione Salvatore Maugeri, Pavia, Italy (Surbone) Fondazione Policlinico IRCCS San Matteo, Pavia, Italy

(Tedeschi) University of Pavia, Pavia, Italy

Publisher

American Thoracic Society

Year of Publication

2015

Link to the Ovid Full Text or citation:

[Click here for full text options](https://ovidsp.ovid.com/ovidweb.cgi?T=JS&CSC=Y&NEWS=N&PAGE=fulltext&D=emed16&AN=72050180)

Link to the External Link Resolver:

[LibKey NHS](https://libkey.io/libraries/2789/openurl?genre=article&sid=OVID:emed16&genre=article&id=pmid:&id=doi:&issn=1073-449X&volume=191&issue=MeetingAbstracts&spage=&pages=&date=2015&title=American+Journal+of+Respiratory+and+Critical+Care+Medicine&atitle=Effects+of+1-year+treatment+with+nocturnal+continuous+positive+pressure+ventilation+(CPAP)+in+overlap+syndrome&aulast=Schreiber)

147.

A comparison between COPD and non-COPD patients and pulmonary rehabilitation outcomes. Does gender matter?.

Nguyen L.-P., Beck E., Cayetano K., Li C.S., Hardin K.

Embase

American Journal of Respiratory and Critical Care Medicine. Conference: American Thoracic Society International Conference, ATS 2015. Denver, CO United States. Conference Publication: (var.pagings). 191(MeetingAbstracts) (no pagination), 2015. Date of Publication: 2015.

[Conference Abstract]

AN: 72049811

Introduction: Pulmonary rehabilitation (PR) programs traditionally instituted for patients with chronic obstructive pulmonary disease (COPD are now treating patients with all forms of pulmonary disease. Little is known regarding differences in exercise and PR outcomes in non-COPD patients, particularly between genders. This study extends our prior investigation on gender differences and exams the response to PR in both men and women with COPD and non-COPD by examining exercise and functional capacities, exploring patients' understanding of their disease processes, as well as perceived quality of life (QOL).

Method(s): This is a retrospective analysis examining patients who have participated in the outpatient UC Davis (UCD) Medical Center PR program from January 2011 to June 2014. The following dependent variables were measured at baseline and after 8 weeks of PR: 6 minute-walk-test (6MWT), treadmill distance and duration, load and duration using the NuStep TM, biceps curls, front arm raises, St. George's respiratory questionnaire, Pittsburgh sleep quality index (PSQI) scores, and UC San Diego shortness of breath (SOB) scores.

Result(s): Eighty patients were included: 38 male (22 COPD, 16 non-COPD) and 42 female (32 COPD, 10 non-COPD). Although there was an overall improvement in 6MW distance in all patients (+31.37 meters, p=0.0003), subgroup analysis showed that only COPD patients, in both females and males (40.7 meters, p=0.0087; +37.45 meters, p=0.0223, respectively) accounted for the difference. Both genders also showed significant improvement in mean treadmill distance (+0.96 mile, p<0.0001) and NuStep load resistance (+1.42, p<0.0001); however due to the small number of non-COPD individuals in each exercise group, comparison between disease groups was not performed. There was a statistical improvement in all patients in upper body strength: biceps curl load (+107.03, p<0.0001) and front arm raise load (+74.21, p<0.0001). When comparing gender and disease group, no difference was noted between COPD and non-COPD across gender. Regarding functional capacity, only the following subgroups showed statistically significant improvement: St. George's respiratory questionnaire (COPD female -6.06, p=0.0025), **PSQI (all female -0.78, p=0.0467 and COPD male -1.3, p=0.0462 respectively**), UCSD SOB questionnaire (non-COPD female -9.5, p=0.0145).

Conclusion(s): This study revealed that pulmonary rehabilitation is beneficial in both males and females with COPD and non-COPD, although the improvement may occur in different outcome parameters. COPD patients seem to have greater lower extremity performance, whereas there is equal improvement in upper body performance. Contrary to most studies, females showed more consistent symptom improvement than males.

Status

CONFERENCE ABSTRACT

Institution

(Nguyen, Beck, Li) UC Davis Medical Center, Sacramento, CA, United States (Cayetano, Hardin) University of California, Davis, Sacramento, CA, United States

Publisher

American Thoracic Society

Year of Publication

2015

Link to the Ovid Full Text or citation:

[Click here for full text options](https://ovidsp.ovid.com/ovidweb.cgi?T=JS&CSC=Y&NEWS=N&PAGE=fulltext&D=emed16&AN=72049811)

Link to the External Link Resolver:

[LibKey NHS](https://libkey.io/libraries/2789/openurl?genre=article&sid=OVID:emed16&genre=article&id=pmid:&id=doi:&issn=1073-449X&volume=191&issue=MeetingAbstracts&spage=&pages=&date=2015&title=American+Journal+of+Respiratory+and+Critical+Care+Medicine&atitle=A+comparison+between+COPD+and+non-COPD+patients+and+pulmonary+rehabilitation+outcomes.+Does+gender+matter%3F&aulast=Nguyen)

148.

Pulmonary rehabilitation for obstructive sleep apnea patients.

Windt M.R., Rice C., Regan S., Chase R.

Embase

American Journal of Respiratory and Critical Care Medicine. Conference: American Thoracic Society International Conference, ATS 2015. Denver, CO United States. Conference Publication: (var.pagings). 191(MeetingAbstracts) (no pagination), 2015. Date of Publication: 2015.

[Conference Abstract]

AN: 72049802

Rationale: Because obstructive sleep apnea (OSA) and chronic obstructive pulmonary disease (COPD) share symptoms such as dyspnea, and diminished functional capacity, and reduce quality-of-life, it would follow that pulmonary rehabilitation would be effective in treating OSA. In fact, the "ATS/ERS Statement on Pulmonary Rehabilitation" states that, while its effectiveness has only been clearly established for COPD, it "may be of value for all patients in whom respiratory symptoms are associated with diminished functional capacity or reduced HRQL"1 However, there are few published studies of exercise therapy as a treatment for OSA. Those studies, that do exist, show some moderate gains for OSA patients undergoing exercise therapy.2,3 We hypothesized that pulmonary rehabilitation, using similar assesment tools as with COPD, could be an effective element in the treatment of patients with OSA.

Method(s): Patients undergo an eight week, one session per week course which includes pre-and post-assessment, cardiovascular and strength training, respiratory muscle training, energy conservation, balance training, and nutritional education. Patients are given a detailed training syllabus, at home weekly assignments and are required to maintain daily data sheets. The exercise component is divided into exercise performed on-site and prescriptive training that the patient incorporates into his/her daily routine at home. Patients with type I diabetes, coronary artery disease as a primary condition, or malignant hypertension were excluded from this study. Patients must have had a pulmonary stress test and a pulmonary function test or methacholine challenge test in the prior four months. Pre-and post-assessment included the 6 min. walk test for distance and gait speed. Physiological measurements included body composition, weight, BMI, hips and waist circumference, lipid panel, blood glucose, and G.O.L.D. assessment. Other measures of fitness included right and left leg balance, sit to stand, push-ups, and use of the inspiratory muscle trainer. Quality of life was assessed with the FOSQ questionnaire and fitness, reporting level of physical activity at work and during leisure time, was assessed with the Baecke questionnaire. Results : Pre-and post-outcome measurements showed a statistical improvement in right leg balance (P=.001) and left leg balance (P=.008), 6 min. walk (P=.008), gait speed (P=.022), inspiratory muscle training (P=.001) and sit to stand (P=.028). No significance was seen in BMI, body fat %, Weight, waist circumference, FOSQ questionnaire and Baecke questionnaire.

Conclusion(s): Our study shows that assessment tools used in pulmonary rehabilitation are helpful in the comprehensive management of OSA and demonstrate improvement in functional capacity. (Figure Presented).

Status

CONFERENCE ABSTRACT

Institution

(Windt, Rice, Regan, Chase) Center for Asthma Allergy and Respiratory Disease, North Hampton, NH, United States

Publisher

American Thoracic Society

Year of Publication

2015

Link to the Ovid Full Text or citation:

[Click here for full text options](https://ovidsp.ovid.com/ovidweb.cgi?T=JS&CSC=Y&NEWS=N&PAGE=fulltext&D=emed16&AN=72049802)

Link to the External Link Resolver:

[LibKey NHS](https://libkey.io/libraries/2789/openurl?genre=article&sid=OVID:emed16&genre=article&id=pmid:&id=doi:&issn=1073-449X&volume=191&issue=MeetingAbstracts&spage=&pages=&date=2015&title=American+Journal+of+Respiratory+and+Critical+Care+Medicine&atitle=Pulmonary+rehabilitation+for+obstructive+sleep+apnea+patients&aulast=Windt)

149.

Pulmonary rehabilitation in severely comorbid patients with COPD-OSA overlap syndrome.

Kuchelan D., Campbell S., Martin T., Macrea M.

Embase

Sleep. Conference: 29th Annual Meeting of the Associated Professional Sleep Societies, LLC, SLEEP 2015. Seattle, WA United States. Conference Publication: (var.pagings). 38(SUPPL. 1) (pp A304), 2015. Date of Publication: 2015.

[Conference Abstract]

AN: 71876137

Introduction: The co-existence of both OSA and COPD in a single patient is called "Overlap Syndrome" (OS) and is associated with worse outcomes than that of OSA or COPD-only patients. As in COPD patients pulmonary rehabilitation (PR) reduces symptoms, disability, number of hospitalizations, emergency department or physician office visits and improves the overall quality of life and survival, PR could be a promising strategy towards improving outcome of OS patients. Little is known about the need of PR in OS patients. Therefore, our project aimed to evaluate the prevalence and possible predictors of PR in a cohort of OS patients.

Method(s): Individuals with COPD and severe comorbidities (CAN score above 95) were selected from the Salem VAMC outpatient clinics for Tele-Health services over 1 year period of time. Information on age, body mass index (BMI), home oxygen therapy (HOT), FEV1, and comorbidities (Charlson Comorbidity Index) were available from the Electronic Medical Record. OSA was defined as per AASM guidelines. The need for PR in OS patients was determined by applying GOLD recommendations.

Result(s): Data was available on 38 patients with COPD and severe comorbidities, 14 (36%) of whom also had OSA and were considered OS. In COPD patients the age, BMI, FE VI and CCI were as follows (mean +/- SEM): 72 +/- 1.2 years; 29 +/- 1.2; 50 +/- 2.4%; 5.7 +/- 2.9, respectively. No differences between age, FEV1 and CCI were found between OS and COPD-only patients. Only one patient with OS had mild COPD. Patients with more severe COPD had a higher frequency of HOT (OR 2; 95% CI) independent of age, BMI and CCI.

Conclusion(s): A third of COPD patients with severe comorbidities had OS. A majority of these patients qualified for PR. HOT predicted need for PR in OS. OS patients need to be identified within the OSA and/or COPD population and targeted for PR intervention.

Status

CONFERENCE ABSTRACT

Institution

(Kuchelan, Campbell) Virginia Tech., Carilion School of Medicine, Roanoke, VA, United States (Martin, Macrea) Veterans Affairs Medical Center, Salem, VA, United States

Publisher

Associated Professional Sleep Societies,LLC

Year of Publication

2015

Link to the Ovid Full Text or citation:

[Click here for full text options](https://ovidsp.ovid.com/ovidweb.cgi?T=JS&CSC=Y&NEWS=N&PAGE=fulltext&D=emed16&AN=71876137)

Link to the External Link Resolver:

[LibKey NHS](https://libkey.io/libraries/2789/openurl?genre=article&sid=OVID:emed16&genre=article&id=pmid:&id=doi:&issn=0161-8105&volume=38&issue=SUPPL.+1&spage=A304&pages=A304&date=2015&title=Sleep&atitle=Pulmonary+rehabilitation+in+severely+comorbid+patients+with+COPD-OSA+overlap+syndrome&aulast=Kuchelan)

150.

Alcohol at bedtime induces minor changes in sleep stages and blood gases in stable chronic obstructive pulmonary disease.

Holmedahl N.H., Overland B., Fondenes O., Ellingsen I., Hardie J.A.

Embase

Sleep and Breathing. 19(1) (pp 307-314), 2015. Date of Publication: 01 Mar 2015.

[Article]

AN: 608263475

Purpose/background: The purpose of this study is to explore the effect of a moderate dose of alcohol on sleep architecture and respiration in chronic obstructive pulmonary disease (COPD). Alcohol depresses both hypercapnic and hypoxic ventilatory drives in awake, normal individuals and reduces the amount of rapid eye movement (REM) sleep and oxygen saturation (SpO2) in sleeping COPD subjects.

Method(s): Prospectively designed, open-label interventional study in a pulmonary rehabilitation hospital. Twenty-six (nine males) stable inpatients, median forced expiratory volume first second (FEV1) 40.5 % of predicted, median age 65 years, investigated by polysomnography including transcutaneous measurement of carbon dioxide pressure increase (DELTAPtcCO2) in randomized order of either control sleep or intervention with 0.5 g of ethanol/kilogram bodyweight, taken orally immediately before lights off.

Result(s): Alcohol induced a mean increase (95 % confidence interval, [CI]) in the mean DELTAPtcCO2 of 0.10 kPa (0.002-0.206, P = 0.047) and a mean decrease (CI) in the REM-sleep percentage of total sleep time (REM % of TST) of 3.1 % (0.2-6.0), (P = 0.020). Six subjects with apnea/hypopnea index (AHI) >=15 had fewer apneas/hypopneas during alcohol versus control sleep (mean reduction of AHI 11 (1-20), P = 0.046). Alcohol-sleep changes in SpO2, but not in DELTAPtcCO2, correlated with daytime arterial pressures of carbon dioxide (PaCO2) and oxygen (PaO2).

Conclusion(s): Occasional use of a moderate, bedtime dose of alcohol has only minor respiratory depressant effects on the majority of COPD subjects, and in a minority even slightly improves respiration during sleep. However, caution is appropriate as this study is small and higher doses of alcohol may result in major respiratory depressive and additional negative health effects.

Copyright © 2014, The Author(s).

PMID

24935686 [<https://www.ncbi.nlm.nih.gov/pubmed/?term=24935686>]

Status

Embase

Institution

(Holmedahl, Ellingsen) LHL-klinikkene, Glittre, Glittreklinikken, postboks 104 Aneby, Hakadal 1485, Norway (Overland) ENT-Department, Lovisenberg Diakonale Hospital, Oslo, Norway

(Fondenes) Norwegian National Centre of Excellence in Home Mechanical Ventilation, Haukeland University Hospital, Bergen, Norway

(Holmedahl, Hardie) Department of Clinical Science, University of Bergen, Bergen, Norway

Publisher

Springer Verlag (E-mail: service@springer.de)

Year of Publication

2015

Link to the Ovid Full Text or citation:

[Click here for full text options](https://ovidsp.ovid.com/ovidweb.cgi?T=JS&CSC=Y&NEWS=N&PAGE=fulltext&D=emed16&AN=608263475)

Link to the External Link Resolver:

[LibKey NHS](https://libkey.io/libraries/2789/openurl?genre=article&sid=OVID:emed16&genre=article&id=pmid:24935686&id=doi:10.1007%2Fs11325-014-1020-y&issn=1520-9512&volume=19&issue=1&spage=307&pages=307-314&date=2015&title=Sleep+and+Breathing&atitle=Alcohol+at+bedtime+induces+minor+changes+in+sleep+stages+and+blood+gases+in+stable+chronic+obstructive+pulmonary+disease&aulast=Holmedahl)

151.

Pulmonary rehabilitation. Rehabilitacion respiratoria <Rehabilitacion respiratoria.>

Guell Rous M.R., Diaz Lobato S., Rodriguez Trigo G., Morante Velez F., San Miguel M., Cejudo P., Ortega Ruiz F., Munoz A., Galdiz Iturri J.B., Garcia A., Servera E.

Embase

Archivos de Bronconeumologia. 50(8) (pp 332-344), 2014. Date of Publication: August 2014.

[Article]

AN: 53228426

Pulmonary rehabilitation (PR) has been shown to improve dyspnea, exercise capacity and health-related quality of life in patients with chronic obstructive pulmonary disease (COPD). PR has also shown benefits in diseases other than COPD but the level of evidence is lower. The fundamental components of PR programs are muscle training, education and chest physiotherapy. Occupational therapy, psychosocial support and nutritional intervention should also be considered. Home programs have been shown to be as effective as hospital therapy. The duration of rehabilitation programs should not be less than 8 weeks or 20 sessions. Early initiation of PR, even during exacerbations, has proven safe and effective. The use of oxygen or noninvasive ventilation during training is controversial and dependent on the patient's situation. At present, the best strategy for maintaining the benefits of PR in the long term is unknown. Longer PR programs or telemedicine could play a key role in extending the results obtained.

Copyright © 2013 SEPAR

Status

Embase

Institution

(Guell Rous, Morante Velez) Servicio de Neumologia, Hospital de la Santa Creu i Sant Pau, Barcelona, Spain (Diaz Lobato) Servicio de Neumologia, Hospital Universitario Ramon y Cajal, Madrid, Spain

(Rodriguez Trigo) Servicio de Neumologia, Hospital Clinico San Carlos, Facultad de Medicina, Universidad Complutense, Madrid, Spain

(San Miguel) Facultad de Ciencias de la Salud, Universidad San Jorge, Villanueva de Gallego, Zaragoza, Spain

(Cejudo, Ortega Ruiz) Servicio de Neumologia, Hospital Virgen del Rocio, CIBERES, IBIS, Sevilla, Spain

(Munoz) Servicio de Neumologia, Hospital General Universitario de Elda, Elda, Alicante, Spain

(Galdiz Iturri) Servicio de Neumologia, Hospital de Cruces, Ciberes UPV/EHU, Barakaldo, Bizkaia, Spain

(Garcia) Servicio de Neumologia, Hospital Universitario Central de Asturias, Oviedo, Spain

(Servera) Servicio de Neumologia, Hospital Clinico de Valencia, Valencia, Spain

Publisher

Elsevier Doyma (E-mail: editorial@elsevier.com)

Year of Publication

2014

Link to the Ovid Full Text or citation:

[Click here for full text options](https://ovidsp.ovid.com/ovidweb.cgi?T=JS&CSC=Y&NEWS=N&PAGE=fulltext&D=emed15&AN=53228426)

Link to the External Link Resolver:

[LibKey NHS](https://libkey.io/libraries/2789/openurl?genre=article&sid=OVID:emed15&genre=article&id=pmid:&id=doi:10.1016%2Fj.arbr.2014.06.011&issn=0300-2896&volume=50&issue=8&spage=332&pages=332-344&date=2014&title=Archivos+de+Bronconeumologia&atitle=Rehabilitacion+respiratoria&aulast=Guell+Rous)

152.

Sleep hypoventilation and daytime hypercapnia in stable chronic obstructive pulmonary disease.

Holmedahl N.H., Overland B., Fondenes O., Ellingsen I., Hardie J.A.

Embase

International Journal of COPD. 9 (pp 265-275), 2014. Date of Publication: 27 Feb 2014.

[Article]

AN: 372504472

Purpose: To explore the associations between sleep hypoventilation (SH) and daytime arterial pressures of carbon dioxide (PaCO2), sleep stages, and sleep apneas/hypopneas (AHI) in subjects with chronic obstructive pulmonary disease (COPD). SH has previously been found in COPD-subjects with chronic hypercapnic respiratory failure (CHRF) using supplementary oxygen (LTOT), and has been proposed as a possible predictor for CHRF.

Patients and Methods: A prospectively designed observational study in a pulmonary rehabilitation hospital of 100 (39 male) stable COPD inpatients with a mean forced expiratory volume in 1 second (FEV1) of 1.1 L (42% of predicted) and a mean age of 64 years, using polysomnography with transcutaneous measurement of carbon dioxide pressure increase (DELTAPtcCO2).

Result(s): SH as defined by the American Academy of Sleep Medicine (AASM) was found in 15 of the subjects, seven of whom used LTOT. However, six had SH despite being normocapnic during the daytime (only one on LTOT). Subjects with SH had a greater DELTAPtcCO2 increase from nonrapid eye movement (NREM) to rapid eye movement (REM) sleep stages compared to non-SH subjects (mean [standard deviation] between-groups difference =0.23(0.20) kPa, P<0.0005). Subjects with apnea/hypopnea index >=15 (overlap, N=27) did not differ from those with COPD alone (AHI<5, N=25) in sleep DELTAPtcCO2 or daytime PaCO2. A regression model with the variables FEV1, LTOT, and sleep maximum DELTAPtcCO2 explained 56% of the variance in daytime PaCO2 (F(3, 94) =40.37, P<0.001).

Conclusion(s): In stable COPD, SH as defined by the AASM was found both in normocapnic, non-LTOT subjects and in hypercapnic, LTOT-using subjects. Between-sleep-stage increase in DELTAPtcCO2 was higher in subjects with SH. Overlap subjects did not differ from simple COPD subjects in sleep DELTAPtcCO2 or daytime PaCO2. © 2014 Holmedahl et al.

PMID

24600219 [<https://www.ncbi.nlm.nih.gov/pubmed/?term=24600219>]

Status

Embase

Institution

(Holmedahl, Ellingsen) Glittreklinikken LHL Helse as, Hakadal, Norway (Overland) Lovisenberg Diakonale Hospital, EN T Department, Oslo, Norway

(Fondenes) Norwegian National Centre of Excellence in Home Mechanical Ventilation, Haukeland University Hospital, Bergen, Norway

(Hardie) Department of Clinical Science, University of Bergen, Norway

Publisher

Dove Medical Press Ltd. (PO Box 300-008, Albany, Auckland, New Zealand)

Year of Publication

2014

Link to the Ovid Full Text or citation:

[Click here for full text options](https://ovidsp.ovid.com/ovidweb.cgi?T=JS&CSC=Y&NEWS=N&PAGE=fulltext&D=emed15&AN=372504472)

Link to the External Link Resolver:

[LibKey NHS](https://libkey.io/libraries/2789/openurl?genre=article&sid=OVID:emed15&genre=article&id=pmid:24600219&id=doi:10.2147%2FCOPD.S57576&issn=1176-9106&volume=9&issue=&spage=265&pages=265-275&date=2014&title=International+Journal+of+COPD&atitle=Sleep+hypoventilation+and+daytime+hypercapnia+in+stable+chronic+obstructive+pulmonary+disease&aulast=Holmedahl)

153.

Systemic manifestations and comorbidities of chronic obstructive pulmonary disease.

Most J.F., Possick J., Rochester C.L.

Embase

Clinical Pulmonary Medicine. 21(4) (pp 155-166), 2014. Date of Publication: July 2014.

[Article]

AN: 373457529

Systemic inflammation is associated with chronic obstructive pulmonary disease (COPD) and many other chronic diseases. It is increasingly recognized that individuals with COPD frequently have underlying comorbidities including cardiovascular disease, skeletal muscle dysfunction, depression and anxiety, obstructive sleep apnea, osteoporosis, gastroesophageal reflux disease, anemia, lung cancer, cognitive impairment, diabetes, renal insufficiency, and chronic infections. The link between systemic inflammation, COPD, and its common comorbidities is incompletely understood. However, persons with COPD and comorbidities have a reduced quality of life and functional status, utilize more health care resources, and are at higher risk of hospitalization and death. Screening for and treating comorbidities are important in the integrated care of the COPD patient and may have a substantial impact on morbidity and mortality. © 2014 by Lippincott Williams & Wilkins.

Status

Embase

Institution

(Most) Division of Pulmonary, Critical Care, and Sleep Medicine, Drexel University College of Medicine, 245 North 15th Street, Philadelphia, PA 19102, United States (Possick, Rochester) Section of Pulmonary, Critical Care, and Sleep Medicine, Yale University School of Medicine, NH, United States

(Rochester) VA Connecticut Healthcare System, West Haven, CT, United States

Publisher

Lippincott Williams and Wilkins (E-mail: LRorders@phl.lrpub.com)

Year of Publication

2014

Link to the Ovid Full Text or citation:

[Click here for full text options](https://ovidsp.ovid.com/ovidweb.cgi?T=JS&CSC=Y&NEWS=N&PAGE=fulltext&D=emed15&AN=373457529)

Link to the External Link Resolver:

[LibKey NHS](https://libkey.io/libraries/2789/openurl?genre=article&sid=OVID:emed15&genre=article&id=pmid:&id=doi:10.1097%2FCPM.0000000000000044&issn=1068-0640&volume=21&issue=4&spage=155&pages=155-166&date=2014&title=Clinical+Pulmonary+Medicine&atitle=Systemic+manifestations+and+comorbidities+of+chronic+obstructive+pulmonary+disease&aulast=Most)

154.

The successful treatment of hypercapnic respiratory failure with oral modafinil.

Parnell H., Quirke G., Farmer S., Adeyemo S., Varney V.

Embase

International Journal of COPD. 9 (pp 413-419), 2014. Date of Publication: 30 Apr 2014.

[Article]

AN: 373001752

Hypercapnic respiratory failure is common in advanced chronic obstructive pulmonary disease and is usually treated by nasal ventilation. Not all patients requiring such ventilation can tolerate it, with anxiety and phobia influencing their reaction, along with treatment failure. We report the case histories of six patients with hypercapnic respiratory failure who were at risk of death due to refusal of nasal ventilation or its failure despite ongoing treatment. We report their improvement with oral modafinil 200 mg tablets used as a respiratory stimulant, which led to discharge, improved arterial blood gases, and offset further admissions with hypercapnic respiratory failure. This drug is licensed for narcolepsy and is said to stimulate the respiratory system via the central nervous system. Its use in respiratory failure is an unlicensed indication, and there are no case reports or studies of such use in the literature. Its respiratory stimulant effects appear better than those with protriptyline, which was a drug previously used until its production was discontinued. Our findings suggest that a study of modafinil in hypercapnic respiratory failure would be warranted, especially for patients with treatment failure or intolerance to nasal ventilation. This may offer a way of shortening hospital stay, improving outcome and quality of life, and reducing death and readmissions. © 2014 Parnell et al.

PMID

24812505 [<https://www.ncbi.nlm.nih.gov/pubmed/?term=24812505>]

Status

Embase

Institution

(Parnell, Quirke, Farmer, Varney) Respiratory Department, St Helier Hospital, Carshalton, Surrey, United Kingdom (Adeyemo) Pharmacy Department, St Helier Hospital, Carshalton, Surrey, United Kingdom

Publisher

Dove Medical Press Ltd. (PO Box 300-008, Albany, Auckland, New Zealand)

Year of Publication

2014

Link to the Ovid Full Text or citation:

[Click here for full text options](https://ovidsp.ovid.com/ovidweb.cgi?T=JS&CSC=Y&NEWS=N&PAGE=fulltext&D=emed15&AN=373001752)

Link to the External Link Resolver:

[LibKey NHS](https://libkey.io/libraries/2789/openurl?genre=article&sid=OVID:emed15&genre=article&id=pmid:24812505&id=doi:10.2147%2FCOPD.S54507&issn=1176-9106&volume=9&issue=&spage=413&pages=413-419&date=2014&title=International+Journal+of+COPD&atitle=The+successful+treatment+of+hypercapnic+respiratory+failure+with+oral+modafinil&aulast=Parnell)

155.

Pulmonary rehabilitation improves subjective sleep quality in COPD.

Lan C.-C., Huang H.-C., Yang M.-C., Lee C.-H., Huang C.-Y., Wu Y.-K.

Embase

Respiratory Care. 59(10) (pp 1569-1576), 2014. Date of Publication: October 2014.

[Article]

AN: 606770570

BACKGROUND: Poor sleep quality is often reported among patients with COPD. Pulmonary rehabilitation (PR) is beneficial in improving exercise capacity and health-related quality of life (HRQOL). However, its benefit in terms of sleep quality in patients with COPD remains unclear. This study aimed to investigate the effects of PR on sleep quality of patients with COPD.

METHOD(S): Thirty-four subjects with COPD were studied. All subjects participated in a 12-week (2 sessions/ week) hospital-based out-patient PR study. Baseline and post-PR status were evaluated by spirometry, a sleep questionnaire (Pittsburgh Sleep Quality Index [PSQI]), a disease-specific questionnaire of HRQOL (St George Respiratory Questionnaire [SGRQ]), cardiopulmonary exercise testing, respiratory muscle strength, and the Borg dyspnea scale.

RESULT(S): Mean FEV1/FVC in the subjects was 0.49 +/- 0.13, and the mean FEV1 was 1.06 +/- 0.49 L/min (49.7 +/- 18.0% of predicted). After PR, the PSQI score decreased from 9.41 +/- 4.33 to 7.82 +/- 3.90 (P <.001). The number of subjects with a PSQI score > 5 also decreased (85.3-64.7%, P=.006). There were significant improvements in HRQOL (SGRQ, P =.003), exercise capacity (peak oxygen uptake, P <.001; and work rate, P <.001), dyspnea score (P <.001), and respiratory muscle strength (inspiratory muscle strength, P =.005; and expiratory muscle strength, P =.004) after PR. There were no significant changes in pulmonary function test results (FEV1, P =.77; FVC, P =.90; FEV1/FVC, P =.90).

CONCLUSION(S): PR results in significant improvement in sleep quality, along with concurrent improvements in HRQOL and exercise capacity. PR is an effective nonpharmacologic treatment to improve sleep quality in patients with COPD and should be part of their clinical management.

Copyright © 2014 Daedalus Enterprises.

PMID

24917453 [<https://www.ncbi.nlm.nih.gov/pubmed/?term=24917453>]

Status

Embase

Institution

(Lan, Huang, Yang, Lee, Huang, Wu) Division of Pulmonary Medicine, Taipei Tzu Chi Hospital, Buddhist Tzu Chi Medical Foundation, New Taipei City, Taiwan (Republic of China) (Lan, Huang, Yang, Lee, Huang, Wu) School of Medicine, Tzu Chi University, Hualien, Taiwan (Republic of China)

(Huang) Department of Nursing, Cardinal Tien College of Healthcare and Management, New Taipei City, Taiwan (Republic of China)

Publisher

American Association for Respiratory Care

Year of Publication

2014

Link to the Ovid Full Text or citation:

[Click here for full text options](https://ovidsp.ovid.com/ovidweb.cgi?T=JS&CSC=Y&NEWS=N&PAGE=fulltext&D=emed15&AN=606770570)

Link to the External Link Resolver:

[LibKey NHS](https://libkey.io/libraries/2789/openurl?genre=article&sid=OVID:emed15&genre=article&id=pmid:24917453&id=doi:10.4187%2Frespcare.02912&issn=0020-1324&volume=59&issue=10&spage=1569&pages=1569-1576&date=2014&title=Respiratory+Care&atitle=Pulmonary+rehabilitation+improves+subjective+sleep+quality+in+COPD&aulast=Lan)

156.

Idiopathic pulmonary fibrosis: Diagnosis and treatment in 2013. Fibrose pulmonaire idiopathique: Prise en charge diagnostique et therapeutique en 2013 <Fibrose pulmonaire idiopathique: Prise en charge diagnostique et therapeutique en 2013.>

Israel-Biet D., Juvin K., Dang Tran K., Badia A., Cazes A., Delclaux C.

Embase

Revue de Pneumologie Clinique. 70(1-2) (pp 108-117), 2014. Date of Publication: 2014.

[Review]

AN: 53019615

Idiopathic pulmonary fibrosis (IPF), the etiopathogeny of which is still unknown, is the most frequent and severe of idiopathic interstitial pneumonias. It progressively leads, sometimes more acutely when exacerbations occur, to a restrictive respiratory insufficiency. Its prognosis is very dark with a median survival of 3-5 years. No treatment so far has been curative. Its diagnostic and therapeutic management has been greatly improved due to the technical progress in terms of high-resolution tomodensitometry, to the availability of new drugs with a real antifibrotic potential and to the production of international recommendations. The diagnosis is reached in 2/3 of IPF patients presenting with a typical usual interstitial pneumonitis (UIP) CT-scan pattern. It requires a videothoracoscopic biopsy in the remaining patients. Multidisciplinary discussions are key to a proper diagnosis of IPF. Pirfenidone is presently the only drug with a real antifibrotic potential in mild to moderate forms of the disease (FVC. >. 50% and DLCO. >. 35% predicted). The other ones have proved either inefficient or toxic. It is highly recommended to include patients in innovative targeted protocols. Non-pharmacological management of these patients comprises long-term oxygen therapy, pulmonary rehabilitation and overall lung transplantation. Pulmonary hypertension, to be detected regularly during the follow-up, is associated to a dark prognosis. No specific treatment is efficient in this context. Several comorbidities, particularly frequent in IPF, should be treated when present: gastro-oesophageal reflux, obstructive sleep apnea, emphysema. The particular high frequency of bronchopulmonary cancer should be highlighted. © 2014 Elsevier Masson SAS.

PMID

24566024 [<https://www.ncbi.nlm.nih.gov/pubmed/?term=24566024>]

Status

Embase

Institution

(Israel-Biet, Cazes, Delclaux) Universite Paris-Descartes, Faculte de medecine, 75005 Paris, France (Israel-Biet, Juvin) Service de pneumologie et de soins intensifs, HOpital europeen Georges-Pompidou, AP-HP, 20, rue Leblanc, 75015 Paris, France

(Dang Tran) Service de radiologie, HOpital europeen Georges-Pompidou, AP-HP, 20, rue Leblanc, 75015 Paris, France

(Badia) Service de chirurgie thoracique, HOpital europeen Georges-Pompidou, AP-HP, 20, rue Leblanc, 75015 Paris, France

(Cazes) Service d'anatomopathologie, HOpital europeen Georges-Pompidou, AP-HP, 20, rue Leblanc, 75015 Paris, France

(Delclaux) Service de physiologie, HOpital europeen Georges-Pompidou, AP-HP, 20, rue Leblanc, 75015 Paris, France

Publisher

Elsevier Masson SAS (62 rue Camille Desmoulins, Issy les Moulineaux Cedex 92442, France)

Year of Publication

2014

Link to the Ovid Full Text or citation:

[Click here for full text options](https://ovidsp.ovid.com/ovidweb.cgi?T=JS&CSC=Y&NEWS=N&PAGE=fulltext&D=emed15&AN=53019615)

Link to the External Link Resolver:

[LibKey NHS](https://libkey.io/libraries/2789/openurl?genre=article&sid=OVID:emed15&genre=article&id=pmid:24566024&id=doi:10.1016%2Fj.pneumo.2013.12.004&issn=0761-8417&volume=70&issue=1-2&spage=108&pages=108-117&date=2014&title=Revue+de+Pneumologie+Clinique&atitle=Fibrose+pulmonaire+idiopathique%3A+Prise+en+charge+diagnostique+et+therapeutique+en+2013&aulast=Israel-Biet)

157.

Pulmonary rehabilitation and sleep quality: A before and after controlled study of patients with chronic obstructive pulmonary disease.

McDonnell L.M., Hogg L., McDonnell L., White P.

Embase

npj Primary Care Respiratory Medicine. 24 (no pagination), 2014. Article Number: 14028. Date of Publication: 10 Jul 2014.

[Article]

AN: 373588471

Background:Poor sleep quality is common in chronic obstructive pulmonary disease (COPD). It is associated with poor quality of life. Pulmonary rehabilitation (PR) improves quality of life, exercise capacity, and anxiety and depression. Its effect on sleep quality is uncertain.

Aim(s):To determine whether PR improves sleep quality in COPD.

Method(s):A prospective controlled 'before and after' study of sleep quality in COPD patients attending a community PR programme was conducted. Sleep quality was measured using the Pittsburgh Sleep Quality Index (PSQI). Lung function, disease-specific quality of life (COPD assessment test-CAT), exercise capacity (incremental shuttle walk test-ISWT), and anxiety and depression (Hospital Anxiety and Depression Scale-HADS) were measured. Change in sleep quality was compared with a COPD control group.

Result(s):Twenty-eight participants completed PR. The control group comprised 24 patients. Prevalence of poor sleep quality (PSQI >= 3/45) was 78%. There were no differences between observation and control groups in sleep quality, age or severity. Quality of life was strongly correlated with quality of sleep (r=0.64, P<0.001). PR improved the quality of life (CAT change 3.0; 95% CI, 0.7-5.3), exercise capacity (ISWT change (metres) 81.0; 15.3-146.6), anxiety (HADS score >= 3/48: change 2.33; 0.45-4.22), and depression (HADS score >= 3/48: change 2.90; 1.92-3.88). PR did not improve sleep quality (PSQI mean change 0.79; '0.35 to 1.93).

Conclusion(s):PR did not improve sleep quality in COPD despite improving quality of life, exercise capacity, anxiety and depression. New strategies, independent of PR, are required to improve sleep quality in COPD. © 2014 Primary Care Respiratory Society UK/Macmillan Publishers Limited.

PMID

25010602 [<https://www.ncbi.nlm.nih.gov/pubmed/?term=25010602>]

Status

Embase

Institution

(McDonnell, White) Department of Primary Care and Public Health Sciences, King's College London, King's Health Partners, London, United Kingdom (Hogg, McDonnell) Department of Physiotherapy, Guy's and St Thomas' NHS Foundation Trust, St Thomas' Hospital, London, United Kingdom

Publisher

Nature Publishing Group (Houndmills, Basingstoke, Hampshire RG21 6XS, United Kingdom)

Year of Publication

2014

Link to the Ovid Full Text or citation:

[Click here for full text options](https://ovidsp.ovid.com/ovidweb.cgi?T=JS&CSC=Y&NEWS=N&PAGE=fulltext&D=emed15&AN=373588471)

Link to the External Link Resolver:

[LibKey NHS](https://libkey.io/libraries/2789/openurl?genre=article&sid=OVID:emed15&genre=article&id=pmid:25010602&id=doi:10.1038%2Fnpjpcrm.2014.28&issn=2055-1010&volume=24&issue=&spage=14028&pages=&date=2014&title=npj+Primary+Care+Respiratory+Medicine&atitle=Pulmonary+rehabilitation+and+sleep+quality%3A+A+before+and+after+controlled+study+of+patients+with+chronic+obstructive+pulmonary+disease&aulast=McDonnell)

158.

Ventilatory support during sleep in patients with chronic obstructive pulmonary disease.

Wijkstra P.J., Duiverman M.L.

Embase

Sleep Medicine Clinics. 9(3) (pp 381-390), 2014. Date of Publication: September 2014.

[Article]

AN: 53200199

In conclusion, currently there is no conclusive evidence that NIV should be provided routinely to stable patients with COPD. Nevertheless, patients who are clearly hypercapnic, who receive confirmed effective ventilation by applying higher inspiratory pressures, and have a better compliance might show clinical benefits. The combination of rehabilitation and nocturnal ventilatory support seems to provide more benefits than rehabilitation alone, so this might be a situation in which chronic NIV is effective. © 2014 Elsevier Inc. All rights reserved.

Status

Embase

Institution

(Wijkstra, Duiverman) Department of Pulmonary Diseases/Home Mechanical Ventilation, University Medical Centre Groningen, Post-Box 30.001, Groningen 9700 RB, Netherlands

Publisher

W.B. Saunders

Year of Publication

2014

Link to the Ovid Full Text or citation:

[Click here for full text options](https://ovidsp.ovid.com/ovidweb.cgi?T=JS&CSC=Y&NEWS=N&PAGE=fulltext&D=emed15&AN=53200199)

Link to the External Link Resolver:

[LibKey NHS](https://libkey.io/libraries/2789/openurl?genre=article&sid=OVID:emed15&genre=article&id=pmid:&id=doi:10.1016%2Fj.jsmc.2014.05.012&issn=1556-407X&volume=9&issue=3&spage=381&pages=381-390&date=2014&title=Sleep+Medicine+Clinics&atitle=Ventilatory+support+during+sleep+in+patients+with+chronic+obstructive+pulmonary+disease&aulast=Wijkstra)

159.

Sleep disorders in asthma and chronic obstructive pulmonary disease (COPD)-impact, diagnosis and treatment strategies. Bedeutung von schlafstorungen, deren abklarung und behandlung bei asthma und COPD <Bedeutung von schlafstorungen, deren abklarung und behandlung bei asthma und COPD.>

Boing S., Randerath W.J.

Embase

Therapeutische Umschau. 71(5) (pp 301-308), 2014. Date of Publication: May 2014.

[Review]

AN: 373031457

Sleep disturbances (SD) are a frequent finding in patients with asthma and chronic obstructive pulmonary disease (COPD) and have a negative impact on quality of life and the clinical course of the disease. The causes of SD are multiple and include for example respiratory symptoms and comorbidities. On the other hand sleep goes along with multiple physiological changes in respiration, so that sleep itself interacts with asthma and COPD. This interaction favors respiratory symptoms and may lead to hypoxemia and hypercapnia. A further complication of the respiratory situation and the clinical course can be found in asthma and COPD patients with coexisting obstructive sleep apnea syndrome (OSAS). Due to the heterogeneity of SD in asthma and COPD, a detailed patient survey is the most important diagnostical tool. Based on the survey further technical examinations should be considered. Treatment strategies for the reduction of SD in asthma and COPD include an optimized medication and treatment of comorbidities. If indicated oxygen therapy, positive pressure breathing and pulmonary rehabilitation can contribute. © 2014 Verlag Hans Huber, Hogrefe AG, Bern.

PMID

24794341 [<https://www.ncbi.nlm.nih.gov/pubmed/?term=24794341>]

Status

Embase

Institution

(Boing) Institut fur Pneumologie, Universitat Witten Herdecke, Germany (Randerath) Klinik fur Pneumologie und Allergologie, Schlaf-und Beatmungsmedizin, Bethanien Krankenhaus, Solingen, Germany

Publisher

Verlag Hans Huber AG (E-mail: verlag@hanshuber.com)

Year of Publication

2014

Link to the Ovid Full Text or citation:

[Click here for full text options](https://ovidsp.ovid.com/ovidweb.cgi?T=JS&CSC=Y&NEWS=N&PAGE=fulltext&D=emed15&AN=373031457)

Link to the External Link Resolver:

[LibKey NHS](https://libkey.io/libraries/2789/openurl?genre=article&sid=OVID:emed15&genre=article&id=pmid:24794341&id=doi:10.1024%2F0040-5930%2Fa000517&issn=0040-5930&volume=71&issue=5&spage=301&pages=301-308&date=2014&title=Therapeutische+Umschau&atitle=Bedeutung+von+schlafstorungen%2C+deren+abklarung+und+behandlung+bei+asthma+und+COPD&aulast=Boing)

160.

Pulmonary rehabilitation improves only some domains of health-related quality of life measured by the Short Form-36 questionnaire.

Limsuwat C., McClellan R., Amiri H.M., Nugent K.

Embase

Annals of Thoracic Medicine. 9(3) (pp 144-148), 2014. Date of Publication: July-September 2014.

[Article]

AN: 373324349

Background: Pulmonary rehabilitation (PR) has inconsistent effects on health-related quality of life (HRQL) in patients with chronic lung diseases. We evaluated the effect of PR on HRQL outcomes using the 36-item short form of the medical outcomes (SF-36).

Method(s): We retrospectively reviewed the files of all patients who completed PR in 2010, 2011, and first half of 2012. We collected information on demographics, symptoms, pulmonary function tests, 6-minute walk tests (6-MWT), and responses on the SF-36 survey, including the physical component score (PCS) and mental component score (MCS).

Result(s): The study included 19 women and 22 men. The mean age was 69.8 +/- 8.5 years. The diagnoses included chronic obstructive pulmonary disease (COPD; n = 31), asthma (n = 3), interstitial lung disease (n = 5), and obstructive sleep apnea (OSA; n = 2). The mean forced expiratory volume-one second (FEV1) was 1.16 +/- 0.52 L (against 60.5 +/- 15.9% of predicted value). There was a significant improvement in 6-MWT (P < 0.0001). The PCS improved post-PR from 33.8 to 34.5 (P = 0.02); the MCS did not change.

Conclusion(s): These patients had low SF-36 scores compared to the general population; changes in scores after PR were low. These patients may need frequent HRQL assessment during rehabilitation, and PR programs should consider program modification in patients with small changes in mental health.

Status

Embase

Institution

(Limsuwat, McClellan, Amiri, Nugent) Department of Internal Medicine, Texas Tech Health Sciences Center, Lubbock, TX, United States

Publisher

Medknow Publications (B9, Kanara Business Centre, off Link Road, Ghatkopar (E), Mumbai 400 075, India)

Year of Publication

2014

Link to the Ovid Full Text or citation:

[Click here for full text options](https://ovidsp.ovid.com/ovidweb.cgi?T=JS&CSC=Y&NEWS=N&PAGE=fulltext&D=emed15&AN=373324349)

Link to the External Link Resolver:

[LibKey NHS](https://libkey.io/libraries/2789/openurl?genre=article&sid=OVID:emed15&genre=article&id=pmid:&id=doi:10.4103%2F1817-1737.134068&issn=1817-1737&volume=9&issue=3&spage=144&pages=144-148&date=2014&title=Annals+of+Thoracic+Medicine&atitle=Pulmonary+rehabilitation+improves+only+some+domains+of+health-related+quality+of+life+measured+by+the+Short+Form-36+questionnaire&aulast=Limsuwat)

161.

High prevalence of obstructive sleep apnea in patients with moderate to severe COPD.

Soler X., Gaio E., DeYoung P., Elliott A.R., Limberg T., Villa A., Baylon M., Sison-Tojino R., Mohney A., Smales E., Loredo J.S., Ramsdell J.W., Malhotra A., Ries A.L.

Embase

American Journal of Respiratory and Critical Care Medicine. Conference: American Thoracic Society International Conference, ATS 2014. San Diego, CA United States. Conference Publication: (var.pagings). 189(MeetingAbstracts) (no pagination), 2014. Date of Publication: 2014.

[Conference Abstract]

AN: 72047821

Introduction: Chronic obstructive pulmonary disease (COPD) and obstructive sleep apnea (OSA) are common disorders associated with substantial morbidity, mortality, and healthcare costs. Recent data (2007-10) indicate an increasing trend with 26% of persons 30-70 years of age who may have mild to severe OSA (AHI >=5/h). However, epidemiologic studies have reported mixed findings regarding the overlap of OSA and COPD. Multiple possible mechanisms could underlie OSA in these patients (anatomical, physiological etc). We hypothesized that patients with advanced COPD would have a high prevalence of OSA. Such findings could contribute to poor sleep and quality of life and support a mechanism underlying cardiovascular events in patients with severe COPD. To that end, we investigated the prevalence of OSA in patients with moderate to severe COPD in the UCSD Pulmonary Rehabilitation (PR) Program.

Method(s): From September 2010 to August 2012, 54 subjects with moderate to severe COPD enrolled in the UCSD PR were recruited for this protocol. At baseline all subjects completed outcome measures of exercise tolerance, questionnaires to assess symptoms and health-related quality of life, and full-unattended polysomnography. OSA was considered present if Apnea-Hypopnea Index (AHI) was 5/h or greater. Also, OSA was also suggested by Oxygen Desaturation Index (ODI) > 5/h and 3% desaturation. as the presence of supplemental oxygen may make AHI assessment in COPD difficult. Results [mean +/-SD]. Forty-four subjects (81%) completed the study and all outcome measures: male 56%; age 67+/-12 years; BMI 27+/-6 kg/m2; pack-years 38 +/-12; 19 (43%) on supplemental oxygen; baseline spirometric FEV1 (% pred.) 42.8+/-19.8, FVC (% pred.) 75.9+/-18.4, FEV1/FVC = 42.3+/-13.1. Twenty-seven subjects (61%) were found to have OSA. Based on ODI and desaturation criteria 52% had OSA. In the subgroup of patients without supplemental oxygen (n=25), 72% had OSA based on AHI. Twenty patients (45%) had poor sleep efficiency (<85%) [80% +/-14]. Sleep architecture was abnormal with the presence of more light sleep [S1 22% +/-15, S2 59%+/-14, S3 7%+/-8, REM 12%+/-8].

Status

CONFERENCE ABSTRACT

Institution

(Soler, Gaio, DeYoung, Elliott, Limberg, Villa, Baylon, Sison-Tojino, Mohney, Smales, Loredo, Ramsdell, Malhotra, Ries) UC San Diego, San Diego, CA, United States

Publisher

American Thoracic Society

Year of Publication

2014

Link to the Ovid Full Text or citation:

[Click here for full text options](https://ovidsp.ovid.com/ovidweb.cgi?T=JS&CSC=Y&NEWS=N&PAGE=fulltext&D=emed15&AN=72047821)

Link to the External Link Resolver:

[LibKey NHS](https://libkey.io/libraries/2789/openurl?genre=article&sid=OVID:emed15&genre=article&id=pmid:&id=doi:&issn=1073-449X&volume=189&issue=MeetingAbstracts&spage=&pages=&date=2014&title=American+Journal+of+Respiratory+and+Critical+Care+Medicine&atitle=High+prevalence+of+obstructive+sleep+apnea+in+patients+with+moderate+to+severe+COPD&aulast=Soler)

162.

Comorbidity burden and healthcare resource use in patients with idiopathic pulmonary fibrosis (IPF) in the united states (US) military health system.

Yu Y., Goehring E., Nguyen-Khoa B.-A., Holmes J., Jones J., Evans A., Sicignano N.

Embase

Chest. Conference: CHEST 2014. Austin, TX United States. Conference Publication: (var.pagings). 146(4 MEETING ABSTRACT) (no pagination), 2014. Date of Publication: October 2014.

[Conference Abstract]

AN: 71780503

PURPOSE: Limited data have been published on comorbidity burden and healthcare resource use (HRU) in patients with IPF. The objectives of this study were to quantify comorbidity burden, mortality and HRU among IPF patients in the US Department of Defense (DoD) Military Health System (MHS).

METHOD(S): Adult patients with a new IPF diagnosis were identified in the DoD-MHS database from 10/2006 to 3/2012 if they had >=2 claims of idiopathic interstitial pneumonia (ICD-9-CM 516.3) OR one claim of 516.3 and one claim of post-inflammatory pulmonary fibrosis (ICD-9-CM 515). The date of the first 516.3 claim was defined as the index date. The IPF diagnosis was confirmed through review of clinical notes including computed tomography/lung biopsy results. IPF-related comorbidities defined in the 2011 ATS guidelines were assessed for one year pre-index; mortality was estimated for one-year and 5-year post-index; all-cause HRU was assessed for one year post-index and measured as hospitalizations, outpatient visits/services, emergency room (ER) visits, and non-drug interventions (oxygen therapy/pulmonary rehabilitation).

RESULT(S): 67 newly diagnosed IPF patients (mean age: 66 years; 43% female) were confirmed by clinical notes. Comorbidities assessed at baseline include hypertension (76%), chronic obstructive pulmonary disease (33%), diabetes mellitus (30%), obstructive sleep apnea (16%), and congestive heart failure (13%). Observed mortality rates at year 1 and year 5 post-index were 15% (n=10) and 22% (n=15) respectively. Among the 51 patients with >= 12 months of data: 59% were admitted to hospital [mean (SD) length of stay=9.3 (12.4) days] and 43% to ER within the first year post-index; they accrued 30 hospitalizations, 235 ER visits, 612 outpatient service encounters, 317 office visits, 276 claims for oxygen therapy, 81 claims for pulmonary rehabilitation, 15 lung biopsy procedures, and 34 CT/HRCT procedures within the first year post-index.

CONCLUSION(S): High comorbidity burden and mortality as well as considerable HRU post-IPF diagnosis were observed in this study cohort. This descriptive analysis provides a basis for future investigations in IPF. CLINICAL IMPLICATIONS: Innovative disease management and effective treatments are needed for IPF patients.

Status

CONFERENCE ABSTRACT

Institution

(Yu, Goehring, Nguyen-Khoa, Holmes, Jones, Evans, Sicignano) Naval Hospital Camp Lejeune, Camp Lejeune, NC, United States

Publisher

American College of Chest Physicians

Year of Publication

2014

Link to the Ovid Full Text or citation:

[Click here for full text options](https://ovidsp.ovid.com/ovidweb.cgi?T=JS&CSC=Y&NEWS=N&PAGE=fulltext&D=emed15&AN=71780503)

Link to the External Link Resolver:

[LibKey NHS](https://libkey.io/libraries/2789/openurl?genre=article&sid=OVID:emed15&genre=article&id=pmid:&id=doi:10.1378%2Fchest.1991635&issn=0012-3692&volume=146&issue=4+MEETING+ABSTRACT&spage=373a&pages=&date=2014&title=Chest&atitle=Comorbidity+burden+and+healthcare+resource+use+in+patients+with+idiopathic+pulmonary+fibrosis+(IPF)+in+the+united+states+(US)+military+health+system&aulast=Yu)

163.

Logan hospital ward based NIV database audit.

Law D., Tran K.

Embase

Respirology. Conference: 2014 Annual Scientific Meetings of the Thoracic Society of Australia and New Zealand and the Australian and New Zealand Society of Respiratory Science. Adelaide, SA Australia. Conference Publication: (var.pagings). 19(SUPPL. 2) (pp 104), 2014. Date of Publication: April 2014.

[Conference Abstract]

AN: 71605650

Aim: To review the first year of ward based Non-Invasive ventilation (NIV) at Logan Hospital. To identify patient demographics, outcomes, length of stay versus duration of NIV therapy in addition to smoking status, pulmonary rehab completion and home oxygen status in an effort to improve patient care.

Method(s): An audit of a clinical database was undertaken. Patient admissions from 19/05/2011 to 19/05/2012 were reviewed. Patients requiring admission for NIV were reviewed. Admissions from the Intensive Care Unit and interhospital transfers already established on therapy were excluded as the initial care was not provided in the ward. Thirty three admissions were identified as requiring NIV which was provided in the ward.

Result(s): Thirty three admissions (17 Female) with an mean age 65.9 +- SD 11.9 (range 32-83). In the COPD cohort the FEV1 ranged from 0.30-1.94 litres (18%-52% predicted). The most common indication for NIV was type 2 respiratory failure (85%), other indications included pneumonia, decompensated obstructive sleep apnoea and hypoxic respiratory failure. 51.5% of patients were on home oxygen therapy, 27% had completed a pulmonary rehabilitation programme, 64% were ex-smokers and 33% were current smokers. Twenty eight (85%) patients were successfully managed with ward based NIV. Three patients required transfer to ICU and required invasive ventilation, two survived. Two patients had NIV withdrawn and died.

Conclusion(s): Ward based NIV is safe and achieves acceptable clinical outcomes.

Status

CONFERENCE ABSTRACT

Institution

(Law) Greenslopes Private Hospital, Australia	(Tran) Logan Hospital, Australia

Publisher

Blackwell Publishing

Year of Publication

2014

Link to the Ovid Full Text or citation:

[Click here for full text options](https://ovidsp.ovid.com/ovidweb.cgi?T=JS&CSC=Y&NEWS=N&PAGE=fulltext&D=emed15&AN=71605650)

Link to the External Link Resolver:

[LibKey NHS](https://libkey.io/libraries/2789/openurl?genre=article&sid=OVID:emed15&genre=article&id=pmid:&id=doi:10.1111%2Fresp.12263&issn=1323-7799&volume=19&issue=SUPPL.+2&spage=104&pages=104&date=2014&title=Respirology&atitle=Logan+hospital+ward+based+NIV+database+audit&aulast=Law)

164.

[Evidences related to pulmonary rehabilitation in the respiratory pathology]. Evidente legate de reabilitarea pulmonara in patologia respiratorie <Evidente legate de reabilitarea pulmonara in patologia respiratorie.>

Croitoru A., Bogdan M.A.

Embase

Pneumologia (Bucharest, Romania). 63(2) (pp 88-90, 92-95), 2014. Date of Publication: 2014 Apr-Jun.

[Review]

AN: 373983534

Pulmonary rehabilitation represents a modern therapeutic approach of respiratory diseases, with a multidisciplinary character, including: physical training, therapeutic education, psychosocial counseling, and nutritional approach. Pulmonary rehabilitation addresses to any patient suffering from pulmonary pathology whose quality of life is affected by the respiratory symptoms, regardless of the degree of functional impairment. This article is an analysis of evidences from the medical literature on outcomes of rehabilitation programs conducted in various lung diseases. The main indication of pulmonary rehabilitation is the chronic obstructive pulmonary disease - COPD (stable state and exacerbation) in which most studies were carried out. Pulmonary rehabilitation in patients with other respiratory pathology has its reason in the pathophysiological changes that they undergo (impaired lung function and gas exchange, muscle atrophy and deconditioning etc.) leading to symptoms, lower exercise tolerance and decrease daily physical activity, all of which ultimately result in impaired quality of life. The role of rehabilitation is reviewed in the following diseases: post-surgical lung volume reduction for emphysema, asthma, bronchiectasis, interstitial lung disease, cystic fibrosis, lung cancer, neuromuscular disease, intensive care, obstructive sleep apnea, pulmonary hypertension, post-tuberculous sequelae, lung transplantation.

PMID

25241555 [<https://www.ncbi.nlm.nih.gov/pubmed/?term=25241555>]

Year of Publication

2014

Link to the Ovid Full Text or citation:

[Click here for full text options](https://ovidsp.ovid.com/ovidweb.cgi?T=JS&CSC=Y&NEWS=N&PAGE=fulltext&D=emed15&AN=373983534)

Link to the External Link Resolver:

[LibKey NHS](https://libkey.io/libraries/2789/openurl?genre=article&sid=OVID:emed15&genre=article&id=pmid:25241555&id=doi:&issn=2067-2993&volume=63&issue=2&spage=88&pages=88-90%2C+92-95&date=2014&title=Pneumologia+(Bucharest%2C+Romania)&atitle=Evidente+legate+de+reabilitarea+pulmonara+in+patologia+respiratorie&aulast=Croitoru)

165.

Nocturnal non-invasive ventilation for cardio-respiratory disorders in adults.

Wahab R., Basner R.C.

Embase

Expert Review of Respiratory Medicine. 7(6) (pp 615-629), 2013. Date of Publication: 2013.

[Review]

AN: 370317000

Following the classic 'iron lung' non-invasive negative pressure ventilator, non-invasive positive pressure ventilation (NIPPV), particularly used 'nocturnally' has developed a broad role in both the acute hospital setting and domiciliary long-term use for many cardio-respiratory disorders associated with acute and chronic ventilatory failure. This role is based in part upon the perceived relative ease of application and discontinuation of NIPPV, ability to avoid intubation or tracheostomy and their associated morbidities and availability of increasingly portable pressure and volume cycled NIPPV devices. Nevertheless, the many methodologies necessary for optimal NIPPV use are often underappreciated by health care workers and patients alike. This review focuses on the rationale, practice, and future directions for 'nocturnal' use of non-invasive positive pressure ventilation (nNIV) in cardio-respiratory disorders in adults which are commonly associated with sleep-related apnea, hypoventilation and hypoxemia: congestive heart failure (CHF), chronic obstructive pulmonary disease (COPD), obesity hypoventilation syndrome (OHS), cystic fibrosis (CF) and neuromuscular disorders. © 2013 Informa UK Ltd.

PMID

24175738 [<https://www.ncbi.nlm.nih.gov/pubmed/?term=24175738>]

Status

Embase

Institution

(Wahab, Basner) Division of Pulmonary, Allergy, and Critical Care Medicine, Columbia University College, 622 West 168th Street, Columbia NY 10032, United States

Publisher

Expert Reviews Ltd.

Clinical Trial Number

<https://clinicaltrials.gov/show/NCT00733343> <https://clinicaltrials.gov/show/NCT01128816>

<https://clinicaltrials.gov/show/NCT01405976>

<https://clinicaltrials.gov/show/NCT01748656>

Year of Publication

2013

Link to the Ovid Full Text or citation:

[Click here for full text options](https://ovidsp.ovid.com/ovidweb.cgi?T=JS&CSC=Y&NEWS=N&PAGE=fulltext&D=emed14&AN=370317000)

Link to the External Link Resolver:

[LibKey NHS](https://libkey.io/libraries/2789/openurl?genre=article&sid=OVID:emed14&genre=article&id=pmid:24175738&id=doi:10.1586%2F17476348.2013.839246&issn=1747-6348&volume=7&issue=6&spage=615&pages=615-629&date=2013&title=Expert+Review+of+Respiratory+Medicine&atitle=Nocturnal+non-invasive+ventilation+for+cardio-respiratory+disorders+in+adults&aulast=Wahab)

166.

Guidelines for the Diagnosis and Treatment of Idiopathic Pulmonary Fibrosis. Normativa sobre el diagnostico y tratamiento de la fibrosis pulmonar idiopatica <Normativa sobre el diagnostico y tratamiento de la fibrosis pulmonar idiopatica.>

Xaubet A., Ancochea J., Bollo E., Fernandez-Fabrellas E., Franquet T., Molina-Molina M., Montero M.A., Serrano-Mollar A.

Embase

Archivos de Bronconeumologia. 49(8) (pp 343-353), 2013. Date of Publication: August 2013.

[Article]

AN: 619907768

Idiopathic pulmonary fibrosis is defined as a chronic fibrosing interstitial pneumonia limited to the lung, of unknown cause, with poor prognosis and few treatment options. In recent years there has been an increase in their prevalence, probably due to the optimization of diagnostic methods and increased life expectancy. The ATS/ERS Consensus (2000) established the diagnostic criteria and recommendations for the assessment of the disease course and treatment. Later studies have helped to redefine diagnostic criteria and treatment options. In 2011, an international consensus was published, establishing diagnostic criteria and new treatment strategies. These guidelines have been updated with the newest aspects of diagnosis and treatment of idiopathic pulmonary fibrosis. A level of evidence has been identified for the most relevant questions, particularly with regard to treatment options.

Copyright © 2012 SEPAR

Status

Embase

Institution

(Xaubet) Servicio de Neumologia, Hospital Clinic, Barcelona, Spain (Xaubet, Molina-Molina, Serrano-Mollar) Centro de Investigaciones Biomedicas en Red de Enfermedades Respiratorias (CIBER), Spain

(Ancochea) Servicio de Neumologia, Hospital Universitario de la Princesa, Madrid, Spain

(Bollo) Servicio de Neumologia, Complejo Asistencial Universitario, Leon, Spain

(Fernandez-Fabrellas) Servicio de Neumologia, Consorcio Hospital General Universitario, Valencia, Spain

(Franquet) Servicio de Radiologia, Hospital de Sant Pau, Barcelona, Spain

(Molina-Molina) Servicio de Neumologia, Hospital Universitario de Bellvitge, L'Hospitalet de Llobregat, Barcelona, Spain

(Montero) Department of Histopathology, Royal Brompton and Haresfield Hospitals NHS, London, United Kingdom

(Serrano-Mollar) Institut d'Investigacions Biomediques de Barcelona, Consejo Superior de Investigaciones Cientificas, Spain

Publisher

Elsevier Doyma (E-mail: editorial@elsevier.com)

Year of Publication

2013

Link to the Ovid Full Text or citation:

[Click here for full text options](https://ovidsp.ovid.com/ovidweb.cgi?T=JS&CSC=Y&NEWS=N&PAGE=fulltext&D=emed14&AN=619907768)

Link to the External Link Resolver:

[LibKey NHS](https://libkey.io/libraries/2789/openurl?genre=article&sid=OVID:emed14&genre=article&id=pmid:&id=doi:10.1016%2Fj.arbr.2013.06.003&issn=0300-2896&volume=49&issue=8&spage=343&pages=343-353&date=2013&title=Archivos+de+Bronconeumologia&atitle=Normativa+sobre+el+diagnostico+y+tratamiento+de+la+fibrosis+pulmonar+idiopatica&aulast=Xaubet)

167.

Major care gaps in asthma, sleep and chronic obstructive pulmonary disease: A road map for knowledge translation.

Boulet L.-P., Bourbeau J., Skomro R., Gupta S.

Embase

Canadian Respiratory Journal. 20(4) (pp 265-269), 2013. Date of Publication: July-August 2013.

[Review]

AN: 369610112

Large gaps between best evidence-based care and actual clinical practice exist in respiratory medicine, and carry a significant health burden. The authors reviewed two key care gaps in each of asthma, chronic obstructive pulmonary disease and obstructive sleep apnea. Using the 'Knowledge-to- Action Framework', the nature of each gap, its magnitude, the barriers that cause and perpetuate it, and past and future strategies that might address the problem were considered. In asthma: disease control is ascertained inadequately, leading to a prevalence of poor asthma control of approximately 50%; and asthma action plans, a key component of asthma management, are provided by only 22% of physicians. In obstructive sleep apnea: disease is under-recognized, with sleep histories ascertained in only 10% of patients; and Canadian polysomnography wait times remain longer than recommended, leading to unnecessary morbidity and societal cost. In chronic obstructive pulmonary disease: a large proportion of patients seen in primary care remain undiagnosed, mainly due to underuse of spirometry; and >10% of patients are referred for pulmonary rehabilitation, despite strong evidence demonstrating its cost effectiveness. Given the prevalence of these chronic conditions and the size and nature of these gaps, the latter exact an important toll on patients, the health care system and society. In turn, complex barriers at the patient, provider and health care system levels contribute to each gap. There have been few previous attempts to bridge these gaps. Innovative and multifaceted implementation approaches are needed and have the potential to make a large impact on Canadian respiratory health. © 2013 Pulsus Group Inc. All rights reserved.

PMID

23936884 [<https://www.ncbi.nlm.nih.gov/pubmed/?term=23936884>]

Status

Embase

Institution

(Boulet) Institut Universitaire de Cardiologie et de Pneumologie de Quebec, Universite Laval, Quebec, Canada (Bourbeau) Respiratory Epidemiology and Clinical Research Unit, Montreal Chest Institute, McGill University Health Centre, Canada

(Bourbeau) Department of Epidemiology, Biostatistics and Occupational Health, McGill University, Montreal, QC, Canada

(Skomro) University of Saskatchewan, Saskatoon, SK, Canada

(Gupta) Department of Medicine, Li Ka Shing Knowledge Institute of St Michael's Hospital, University of Toronto, Bond Wing, 30 Bond Street, Toronto, ON M5B 1W8, Canada

Publisher

Hindawi Limited

Year of Publication

2013

Link to the Ovid Full Text or citation:

[Click here for full text options](https://ovidsp.ovid.com/ovidweb.cgi?T=JS&CSC=Y&NEWS=N&PAGE=fulltext&D=emed14&AN=369610112)

Link to the External Link Resolver:

[LibKey NHS](https://libkey.io/libraries/2789/openurl?genre=article&sid=OVID:emed14&genre=article&id=pmid:23936884&id=doi:10.1155%2F2013%2F496923&issn=1198-2241&volume=20&issue=4&spage=265&pages=265-269&date=2013&title=Canadian+Respiratory+Journal&atitle=Major+care+gaps+in+asthma%2C+sleep+and+chronic+obstructive+pulmonary+disease%3A+A+road+map+for+knowledge+translation&aulast=Boulet)

168.

Management of depression and anxiety in COPD.

Cafarella P.A., Effing T.W., Barton C., Ahmed D., Frith P.A.

Embase

European Respiratory Monograph. 59 (pp 144-163), 2013. Date of Publication: 2013.

[Article]

AN: 368517517

Depression and anxiety are prevalent comorbidities in COPD and often appear together. Numerous theoretical models have been proposed to explain this relationship, with most suggesting bidirectional and complex pathways. Mental health assessment in COPD remains too infrequent and should be integrated into standard practice. Appropriate use of mental health screening tools, diagnostic resources and referral pathways should be implemented for optimal management. There is evidence that depression and anxiety in COPD negatively impacts on important health outcomes, such as COPD symptom burden, physical function, health-related quality of life, adherence with recommended treatments and mortality whilst increasing disability, exacerbation rates, hospitalisations and length of stay. Treatment options for managing depression and anxiety in COPD are less frequently documented. Whilst some evidence exists supporting the efficacy of pulmonary rehabilitation, pharmacological therapy, cognitive behavioural therapy, self-management programmes, relaxation and palliative care interventions in managing depression and anxiety in COPD, there remains a paucity of high-quality studies in the field consequently limiting integration into evidence-based clinical guidelines. © ERS 2013.

Status

Embase

Institution

(Cafarella, Effing, Ahmed, Frith) Dept of Respiratory Medicine, Repatriation General Hospital, Daw Park, SA 5041, Australia (Barton) Social Health Sciences, Flinders University, Bedford Park, Australia

Publisher

European Respiratory Society (442 Glossop Road, Sheffield S10 2PX, United Kingdom)

Year of Publication

2013

Link to the Ovid Full Text or citation:

[Click here for full text options](https://ovidsp.ovid.com/ovidweb.cgi?T=JS&CSC=Y&NEWS=N&PAGE=fulltext&D=emed14&AN=368517517)

Link to the External Link Resolver:

[LibKey NHS](https://libkey.io/libraries/2789/openurl?genre=article&sid=OVID:emed14&genre=article&id=pmid:&id=doi:10.1183%2F1025448x.10012112&issn=1025-448X&volume=59&issue=59&spage=144&pages=144-163&date=2013&title=European+Respiratory+Monograph&atitle=Management+of+depression+and+anxiety+in+COPD&aulast=Cafarella)

169.

Therapeutic paralysis in veterans with COPD.

Mulhall A.M., Lach L.A., Krzywkowski-Mohn S.M., Welge J.A., Panos R.J.

Embase

Respiratory Medicine. 107(10) (pp 1547-1557), 2013. Date of Publication: October 2013.

[Article]

AN: 52657637

Background Chronic Obstructive Pulmonary Disease (COPD) is a common disorder of Veterans that causes significant morbidity and mortality. To measure Veterans' perceptions about COPD, the effect of COPD on their lives and health, and their needs for improved health, we performed a postal survey. Methods 3263 Veterans with a diagnosis of COPD who received care at the Cincinnati Veterans Affairs Medical Center in 2008 were stratified into quintiles by Veterans Health Administration-associated COPD healthcare cost and uniformly sampled. Results 493 of 1000 surveys (49%) were completed and returned. COPD had different effects on respondents in top and bottom quintiles (highest and lowest COPD-related cost) for: knowledge of COPD diagnosis (89% vs 73%, p = 0.03); activities affected by breathing, including work (69% vs 45%), recreation (85% vs 62%), change in living arrangements (36% vs 16%), and increased need for help (54% vs 25%) (p < 0.05 for all comparisons); emotional effect of respiratory symptoms, including depression (53% vs 30%), fear (41% vs 15%), and helplessness (49% vs 24%) (p < 0.05 for all comparisons). 91% of Veterans were prescribed inhalers and one-quarter had difficulties using them. 25% of Veterans did nothing when they had symptoms of an exacerbation. Conclusions COPD has profound effects on Veterans' breathing related activities and generates many negative emotions. Primary care providers are critical in conveying the diagnosis of COPD and providing information about the disease and its management. Veterans with COPD adhere poorly to their medications, and report little instruction about COPD or its management.

PMID

23827725 [<https://www.ncbi.nlm.nih.gov/pubmed/?term=23827725>]

Status

Embase

Institution

(Mulhall, Panos) Department of Internal Medicine, University of Cincinnati Academic Health Center, 231 Albert Sabin Way, Cincinnati, OH 45267, United States (Lach, Panos) Department of Internal Medicine, Pulmonary Critical Care, and Sleep Division, Cincinnati Veterans Affairs Medical Center, 3200 Vine Street, Cincinnati, OH 45220, United States

(Krzywkowski-Mohn) Primary Care Division, Cincinnati Veterans Affairs Medical Center, 3200 Vine Street, Cincinnati, OH 45220, United States

(Welge) Department of Psychiatry and Behavioral Neuroscience, University of Cincinnati Academic Health Center, 231 Albert Sabin Way, Cincinnati, OH 45267, United States

(Welge) Department of Environmental Health, Division of Epidemiology and Biostatistics, University of Cincinnati Academic Health Center, 231 Albert Sabin Way, Cincinnati, OH 45267, United States

Publisher

W.B. Saunders Ltd (32 Jamestown Road, London NW1 7BY, United Kingdom)

Year of Publication

2013

Link to the Ovid Full Text or citation:

[Click here for full text options](https://ovidsp.ovid.com/ovidweb.cgi?T=JS&CSC=Y&NEWS=N&PAGE=fulltext&D=emed14&AN=52657637)

Link to the External Link Resolver:

[LibKey NHS](https://libkey.io/libraries/2789/openurl?genre=article&sid=OVID:emed14&genre=article&id=pmid:23827725&id=doi:10.1016%2Fj.rmed.2013.05.013&issn=0954-6111&volume=107&issue=10&spage=1547&pages=1547-1557&date=2013&title=Respiratory+Medicine&atitle=Therapeutic+paralysis+in+veterans+with+COPD&aulast=Mulhall)

170.

Pulmonary rehabilitation improves sleep quality in chronic lung disease.

Soler X., Diaz-Piedra C., Ries A.L.

Embase

COPD: Journal of Chronic Obstructive Pulmonary Disease. 10(2) (pp 156-163), 2013. Date of Publication: April 2013.

[Article]

AN: 368666759

Sleep-related disorders are common in patients with chronic obstructive pulmonary disease (COPD) and, possibily, other lung disorders. Exercise has been shown to improve sleep disturbances. In patients with COPD, pulmonary rehabilitation (PR) produces important health benefits with improvement in symptoms, exercise tolerance, and quality of life. However, the effect of PR on sleep quality remains unknown. The aim of this observational study was to evaluate sleep quality in patients with chronic lung disease and the role of PR as a non-pharmacologic treatment to improve sleep. Sixty-four patients with chronic lung disease enrolled in an 8-week comprehensive PR program, and completed the study (48% male; obstructive 72%, restrictive 20%, mixed 8%; 44% on supplemental oxygen). Baseline spirometry mean (SD): FEV1% pred = 48.9 (17.4), FVC% pred = 72.5 (18.1), and FEV1/FVC% = 53.1 (18.9). Exercise tolerance and questionnaires related to symptoms, health-related quality of life (HRQL), and sleep quality using the Pittsburgh Sleep Quality Index (PSQI) were obtained before and after PR. 58% reported poor sleep quality (PSQI > 5) at baseline. Sleep quality improved by 19% (p = 0.017) after PR, along with significant improvements in dyspnea, exercise tolerance, self-efficacy, and HRQL. Sleep quality in patients with chronic lung disease was poor. In addition to expected improvements in symptoms, exercise tolerance, and HRQL after PR, the subgroup of patients with COPD had a significant improvement in sleep quality. These findings suggest that PR may be an effective, non-pharmacologic treatment option for sleep problems in patients with COPD. © 2013 Informa Healthcare USA, Inc.

PMID

23514215 [<https://www.ncbi.nlm.nih.gov/pubmed/?term=23514215>]

Status

Embase

Institution

(Soler, Ries) Division of Pulmonary and Critical Care Medicine, Department of Medicine, University of California, San Diego (UCSD), 200 West Arbor Dr. M/C 8377, San Diego, CA 92103-8377, United States (Diaz-Piedra) Department of Personality, Assessment and Psychological Treatment, University of Granada, Spain

Publisher

Informa Healthcare (52 Vanderbilt Ave., New York 10017, United States)

Year of Publication

2013

Link to the Ovid Full Text or citation:

[Click here for full text options](https://ovidsp.ovid.com/ovidweb.cgi?T=JS&CSC=Y&NEWS=N&PAGE=fulltext&D=emed14&AN=368666759)

Link to the External Link Resolver:

[LibKey NHS](https://libkey.io/libraries/2789/openurl?genre=article&sid=OVID:emed14&genre=article&id=pmid:23514215&id=doi:10.3109%2F15412555.2012.729622&issn=1541-2555&volume=10&issue=2&spage=156&pages=156-163&date=2013&title=COPD%3A+Journal+of+Chronic+Obstructive+Pulmonary+Disease&atitle=Pulmonary+rehabilitation+improves+sleep+quality+in+chronic+lung+disease&aulast=Soler)

171.

Managing severe COPD: Much can be done.

Detering K., Smith T., McDonald C.F.

Embase

Medicine Today. 14(5) (pp 39-44), 2013. Date of Publication: May 2013.

[Article]

AN: 369156483

Severe COPD may be challenging to manage as patients often experience debilitating symptoms that fail to respond completely to disease-specific treatment. Clear communication about the goals of care combined with management of symptoms and comorbidities should enable patients to enjoy a better quality of life.

Status

Embase

Institution

(Detering) Respecting Patient Choices Program, Austin Health, Heidelberg, VIC, Australia (Smith) Campbelltown Hospital, Sydney, NSW, Australia

(Smith) Respiratory Medicine, St. George Hospital, Sydney, NSW, Australia

(Smith) Faculty of Medicine, University of Western Sydney, Sydney, NSW, Australia

(McDonald) Department of Respiratory and Sleep Medicine, Austin Health, Melbourne, VIC, Australia

Publisher

Medicine Today Pty Ltd (P.O. Box 1473, Neutral Bay NSW 2089, Australia)

Year of Publication

2013

Link to the Ovid Full Text or citation:

[Click here for full text options](https://ovidsp.ovid.com/ovidweb.cgi?T=JS&CSC=Y&NEWS=N&PAGE=fulltext&D=emed14&AN=369156483)

Link to the External Link Resolver:

[LibKey NHS](https://libkey.io/libraries/2789/openurl?genre=article&sid=OVID:emed14&genre=article&id=pmid:&id=doi:&issn=1443-430X&volume=14&issue=5&spage=39&pages=39-44&date=2013&title=Medicine+Today&atitle=Managing+severe+COPD%3A+Much+can+be+done&aulast=Detering)

172.

An official American thoracic society/European respiratory society statement: Key concepts and advances in pulmonary rehabilitation.

Spruit M.A., Singh S.J., Garvey C., Zu Wallack R., Nici L., Rochester C., Hill K., Holland A.E., Lareau S.C., Man W.D.-C., Pitta F., Sewell L., Raskin J., Bourbeau J., Crouch R., Franssen F.M.E., Casaburi R., Vercoulen J.H., Vogiatzis I., Gosselink R., Clini E.M., Effing T.W., Maltais F., Van Der Palen J., Troosters T., Janssen D.J.A., Collins E., Garcia-Aymerich J., Brooks D., Fahy B.F., Puhan M.A., Hoogendoorn M., Garrod R., Schols A.M.W.J., Carlin B., Benzo R., Meek P., Morgan M., Rutten-Van Molken M.P.M.H., Ries A.L., Make B., Goldstein R.S., Dowson C.A., Brozek J.L., Donner C.F., Wouters E.F.M.

Embase

American Journal of Respiratory and Critical Care Medicine. 188(8) (pp e13-e64), 2013. Date of Publication: 15 Oct 2013.

[Article]

AN: 370114964

Background: Pulmonary rehabilitation is recognized as a core component of themanagement of individuals with chronic respiratory disease. Since the 2006 American Thoracic Society (ATS)/European Respiratory Society (ERS) Statement on Pulmonary Rehabilitation, there has been considerable growth in our knowledge of its efficacy and scope.

Purpose(s): The purpose of this Statement is to update the 2006 document, including a new definition of pulmonary rehabilitation and highlighting key concepts and major advances in the field.

Method(s): A multidisciplinary committee of experts representing the ATS Pulmonary Rehabilitation Assembly and the ERS Scientific Group 01.02, "Rehabilitation and Chronic Care," determined the overall scope of this update through group consensus. Focused literature reviews in key topic areas were conducted by committee members with relevant clinicaland scientific expertise.The final content of this Statement was agreed on by all members.

Result(s): An updated definition of pulmonary rehabilitation is proposed. New data are presented on the science and application of pulmonary rehabilitation, including its effectiveness in acutely ill individuals with chronic obstructive pulmonary disease, and in individuals with other chronic respiratory diseases.The important role of pulmonary rehabilitation in chronic disease management is highlighted. In addition, the role of health behavior change in optimizing and maintaining benefits is discussed.

Conclusion(s): The considerable growth in the science and application of pulmonary rehabilitation since 2006 adds further support for its efficacy in a wide range of individuals with chronic respiratory disease. Copyright © 2013 by the American Thoracic Society.

PMID

24127811 [<https://www.ncbi.nlm.nih.gov/pubmed/?term=24127811>]

Status

Embase

Publisher

American Thoracic Society (61 Broadway 4th Floor, New York NY 10006 - 2755, United States)

Year of Publication

2013

Link to the Ovid Full Text or citation:

[Click here for full text options](https://ovidsp.ovid.com/ovidweb.cgi?T=JS&CSC=Y&NEWS=N&PAGE=fulltext&D=emed14&AN=370114964)

Link to the External Link Resolver:

[LibKey NHS](https://libkey.io/libraries/2789/openurl?genre=article&sid=OVID:emed14&genre=article&id=pmid:24127811&id=doi:10.1164%2Frccm.201309-1634ST&issn=1073-449X&volume=188&issue=8&spage=e13&pages=e13-e64&date=2013&title=American+Journal+of+Respiratory+and+Critical+Care+Medicine&atitle=An+official+American+thoracic+society%2FEuropean+respiratory+society+statement%3A+Key+concepts+and+advances+in+pulmonary+rehabilitation&aulast=Spruit)

173.

Audit of acute exacerbations of chronic obstructive pulmonary disease at Waitemata District Health Board, New Zealand.

Johnson C., Connolly M.J., Clover S., Campbell L., Goonan R., Salmon E., Hopley M., Phillips M., Sood J.

Embase

New Zealand Medical Journal. 126(1378) (no pagination), 2013. Date of Publication: 2013.

[Article]

AN: 369314977

Aim To examine management and outcome of patients admitted to Waitemata District Health Board (WDHB) with acute exacerbations of chronic obstructive pulmonary disease (AECOPD) and determine performance according to evidence-based guidelines. Methods Retrospective chart review of all patients admitted to WDHB hospitals with primary diagnosis of AECOPD during May and October 2010. 195 admissions (156 patients) were audited. Results Patients comprised 72 females and 84 males; mean age 73.1 years. 96% were ever-smokers. 10% of patients had BMI <18 kg/m2 and 40% of these received no dietician input. Spirometry was recorded in 72% within the previous 5 years. Chest X-ray was performed in 96% in the first 24 hours and 33% had arterial blood gas (ABG) performed. Twenty-three patients (29%) had acute respiratory acidosis. Continuous positive airways pressure (CPAP) was used in 11 but none received non-invasive ventilation (NIV). Systemic corticosteroids and antibiotics were prescribed to 87% and 84% respectively. Ten percent of patients were referred for pulmonary rehabilitation (PR). Overall 90-day mortality was 6.7% with 3.1% inpatient mortality. Mean length of stay was 5 days. 90- day re-admission rate was 44%. Conclusion Areas of good adherence to best practice guidelines. Room for improvement in use of NIV, ABG and spirometry measurement, and PR referral. © NZMA.

PMID

24045312 [<https://www.ncbi.nlm.nih.gov/pubmed/?term=24045312>]

Status

Embase

Institution

(Johnson, Connolly, Clover, Campbell, Goonan, Salmon, Hopley, Phillips, Sood) North Shore Hospital, Auckland, New Zealand (Connolly) University of Auckland, Auckland, New Zealand

Publisher

New Zealand Medical Association (26 The Terrace, P.O. Box 156, Wellington, New Zealand)

Year of Publication

2013

Link to the Ovid Full Text or citation:

[Click here for full text options](https://ovidsp.ovid.com/ovidweb.cgi?T=JS&CSC=Y&NEWS=N&PAGE=fulltext&D=emed14&AN=369314977)

Link to the External Link Resolver:

[LibKey NHS](https://libkey.io/libraries/2789/openurl?genre=article&sid=OVID:emed14&genre=article&id=pmid:24045312&id=doi:&issn=0028-8446&volume=126&issue=1378&spage=15&pages=&date=2013&title=New+Zealand+Medical+Journal&atitle=Audit+of+acute+exacerbations+of+chronic+obstructive+pulmonary+disease+at+Waitemata+District+Health+Board%2C+New+Zealand&aulast=Johnson)

174.

Better care for patients with COPD and cognitive impairment.

Campman C.A.M., Sitskoorn M.M.

Embase

The Lancet Respiratory Medicine. 1(7) (pp 504-506), 2013. Date of Publication: September 2013.

[Letter]

AN: 52712367

PMID

24461603 [<https://www.ncbi.nlm.nih.gov/pubmed/?term=24461603>]

Status

Embase

Institution

(Campman, Sitskoorn) Department of Cognitive Neuropsychology, Tilburg University, 5000 LE Tilburg, Netherlands (Sitskoorn) Center of Research on Psychology in Somatic Diseases, Tilburg University, 5000 LE Tilburg, Netherlands

Publisher

Lancet Publishing Group (E-mail: cususerv@lancet.com)

Year of Publication

2013

Link to the Ovid Full Text or citation:

[Click here for full text options](https://ovidsp.ovid.com/ovidweb.cgi?T=JS&CSC=Y&NEWS=N&PAGE=fulltext&D=emed14&AN=52712367)

Link to the External Link Resolver:

[LibKey NHS](https://libkey.io/libraries/2789/openurl?genre=article&sid=OVID:emed14&genre=article&id=pmid:24461603&id=doi:10.1016%2FS2213-2600%252813%252970163-2&issn=2213-2600&volume=1&issue=7&spage=504&pages=504-506&date=2013&title=The+Lancet+Respiratory+Medicine&atitle=Better+care+for+patients+with+COPD+and+cognitive+impairment&aulast=Campman)

175.

Nonventilatory strategies to prevent postoperative pulmonary complications.

Guldner A., Pelosi P., De Abreu M.G.

Embase

Current Opinion in Anaesthesiology. 26(2) (pp 141-151), 2013. Date of Publication: April 2013.

[Review]

AN: 52426292

Purpose of Review: In this review, we aimed at providing the most recent and relevant clinical evidence regarding the use of nonventilatory strategies to prevent postoperative pulmonary complications (PPCs) after noncardiac surgery. Recent Findings: Although nonavoidable, most comorbidities can be modified in order to reduce the incidence of pulmonary events postoperatively. The physical status of patients suffering from chronic obstructive pulmonary disease, asthma, obstructive sleep apnea, and congestive heart failure can be improved preoperatively, and a number of measures can be undertaken to prevent PPCs, including physiotherapy for pulmonary rehabilitation and drug therapies. Also, smokers may benefit from both short and long-term smoke cessation. Furthermore, the risk of PPCs may be reduced upon: choice of an adequate anesthesia strategy (e.g. regional vs. general); appropriate neuromuscular blockade and reversal; use of volatile instead of intravenous anesthetics in lung surgery; judicious intravascular volume expansion (restrictive vs. liberal strategy); regional instead of systemic analgesia after major surgery in high-risk patients; more strict indication for nasogastric decompression in order to avoid silent aspiration; and laparoscopic instead of open bariatric surgery.

Summary: Nonventilatory strategies can play an important role in reducing PPCs and improving clinical outcome after noncardiac surgery, especially in high-risk patients. © 2013 Wolters Kluwer Health

Lippincott Williams & Wilkins.

PMID

23385322 [<https://www.ncbi.nlm.nih.gov/pubmed/?term=23385322>]

Status

Embase

Institution

(Guldner, De Abreu) Pulmonary Engineering Group, Department of Anesthesiology and Intensive Care Medicine, University Hospital Dresden, Fetscherstr. 74, 01307 Dresden, Germany (Pelosi) Department of Surgical Sciences and Integrated Diagnostics, University of Genoa, Genoa, Italy

Publisher

Lippincott Williams and Wilkins (250 Waterloo Road, London SE1 8RD, United Kingdom)

Year of Publication

2013

Link to the Ovid Full Text or citation:

[Click here for full text options](https://ovidsp.ovid.com/ovidweb.cgi?T=JS&CSC=Y&NEWS=N&PAGE=fulltext&D=emed14&AN=52426292)

Link to the External Link Resolver:

[LibKey NHS](https://libkey.io/libraries/2789/openurl?genre=article&sid=OVID:emed14&genre=article&id=pmid:23385322&id=doi:10.1097%2FACO.0b013e32835e8bac&issn=0952-7907&volume=26&issue=2&spage=141&pages=141-151&date=2013&title=Current+Opinion+in+Anaesthesiology&atitle=Nonventilatory+strategies+to+prevent+postoperative+pulmonary+complications&aulast=Guldner)

176.

Advances in the outpatient management of chronic obstructive pulmonary disease.

Possick J.D., Most J., Rochester C.L.

Embase

Clinical Pulmonary Medicine. 20(6) (pp 259-270), 2013. Date of Publication: November 2013.

[Article]

AN: 370495076

Chronic obstructive pulmonary disease (COPD) remains highly prevalent, underdiagnosed, and a major cause of morbidity and mortality. Disease rates and cost of care continue to rise annually in the United States and worldwide. Recent years have seen advances in our understanding of COPD as a heterogenous disease with distinct phenotypes. It is increasingly recognized that COPD severity and impact is complex and is inadequately described by airflow obstruction alone. The revised 2011 GOLD guidelines now incorporate multidimensional measures of symptom burden, functional limitations, and exacerbation frequency in patient assessment. Moreover, COPD is commonly associated with multiple medical co-morbidities that also have an impact on the symptom burden, disease severity, and mortality for individual patients. This conceptual frame-shift to recognition of COPD as a heterogenous and multifaceted disease with varying impact on the patient has begun to revolutionize the way COPD care is approached in the outpatient setting. Current management guidelines now emphasize broader-based patient assessment including characterization of patients' phenotypes, symptom burden, functional limitations, exacerbation risk, and co-morbidities to guide therapeutic interventions. Although some forms of therapy continue to be applicable to all patients with COPD, others seem to only benefit patients with selected disease phenotypes. Here we explore some of the recent advances in outpatient COPD management. We consider the varying manifestations of COPD, treatment approaches that remain broadly applicable, as well as selected phenotype-specific treatments, interventions targeted at reducing exacerbations, and management of intractable symptoms. We also suggest areas for future exploration. Copyright © 2013 by Lippincott Williams & Wilkins.

Status

Embase

Institution

(Possick, Most, Rochester) Section of Pulmonary, Critical Care, and Sleep Medicine, Yale University School of Medicine, PO Box 208057, New Haven, CT 06510, United States (Rochester) VA Connecticut Healthcare System, West Haven, CT, United States

Publisher

Lippincott Williams and Wilkins (530 Walnut Street,P O Box 327, Philadelphia PA 19106-3621, United States)

Year of Publication

2013

Link to the Ovid Full Text or citation:

[Click here for full text options](https://ovidsp.ovid.com/ovidweb.cgi?T=JS&CSC=Y&NEWS=N&PAGE=fulltext&D=emed14&AN=370495076)

Link to the External Link Resolver:

[LibKey NHS](https://libkey.io/libraries/2789/openurl?genre=article&sid=OVID:emed14&genre=article&id=pmid:&id=doi:10.1097%2FCPM.0000000000000001&issn=1068-0640&volume=20&issue=6&spage=259&pages=259-270&date=2013&title=Clinical+Pulmonary+Medicine&atitle=Advances+in+the+outpatient+management+of+chronic+obstructive+pulmonary+disease&aulast=Possick)

177.

Medical management of fatigue.

Sharafkhaneh A., Velamuri S., Melendez J., Akhtar F., Hirshkowitz M.

Embase

Sleep Medicine Clinics. 8(2) (pp 265-276), 2013. Date of Publication: June 2013.

[Article]

AN: 369097055

Fatigue is frequently reported in chronic medical conditions. Improvement of the primary condition positively affects health-related quality of life and fatigue. Interventions to achieve exercise rehabilitation robustly affect the fatigue in these patients. Improving sleep may improve the quality of life and fatigue. In some cases, medical management may use wake-promoting medications. The risks and benefits must be carefully weighed. When insomnia is contributing significantly to the overall fatigue, pharmacotherapy may be appropriate. The clinical picture is often complicated by potential interactions with other medications and contraindications. It is important for the physician to be aware of any self-medication. © 2013 Elsevier Inc.

Status

Embase

Institution

(Sharafkhaneh, Velamuri, Melendez, Akhtar, Hirshkowitz) Section of Pulmonary and Critical Care and Sleep Medicine, Baylor College of Medicine, Houston, TX, United States (Sharafkhaneh, Velamuri, Melendez, Akhtar, Hirshkowitz) Sleep Disorders and Research Center, Michael E.DeBakey VA Medical Center, Baylor College of Medicine, Houston, TX, United States

(Hirshkowitz) Menninger Department of Psychiatry, Baylor College of Medicine, Houston, TX, United States

Publisher

W.B. Saunders (Independence Square West, Philadelphia PA 19106-3399, United States)

Year of Publication

2013

Link to the Ovid Full Text or citation:

[Click here for full text options](https://ovidsp.ovid.com/ovidweb.cgi?T=JS&CSC=Y&NEWS=N&PAGE=fulltext&D=emed14&AN=369097055)

Link to the External Link Resolver:

[LibKey NHS](https://libkey.io/libraries/2789/openurl?genre=article&sid=OVID:emed14&genre=article&id=pmid:&id=doi:10.1016%2Fj.jsmc.2013.04.002&issn=1556-407X&volume=8&issue=2&spage=265&pages=265-276&date=2013&title=Sleep+Medicine+Clinics&atitle=Medical+management+of+fatigue&aulast=Sharafkhaneh)

178.

Chronic obstructive pulmonary disease.

Vijayan V.K.

Embase

Indian Journal of Medical Research. 137(2) (pp 251-269), 2013. Date of Publication: February 2013.

[Review]

AN: 368612488

The global prevalence of physiologically defined chronic obstructive pulmonary disease (COPD) in adults aged >40 yr is approximately 9-10 per cent. Recently, the Indian Study on Epidemiology of Asthma, Respiratory Symptoms and Chronic Bronchitis in Adults had shown that the overall prevalence of chronic bronchitis in adults >35 yr is 3.49 per cent. The development of COPD is multifactorial and the risk factors of COPD include genetic and environmental factors. Pathological changes in COPD are observed in central airways, small airways and alveolar space. The proposed pathogenesis of COPD includes proteinase-antiproteinase hypothesis, immunological mechanisms, oxidant-antioxidant balance, systemic inflammation, apoptosis and ineffective repair. Airflow limitation in COPD is defined as a postbronchodilator FEV1 (forced expiratory volume in 1 sec) to FVC (forced vital capacity) ratio <0.70. COPD is characterized by an accelerated decline in FEV1. Co morbidities associated with COPD are cardiovascular disorders (coronary artery disease and chronic heart failure), hypertension, metabolic diseases (diabetes mellitus, metabolic syndrome and obesity), bone disease (osteoporosis and osteopenia), stroke, lung cancer, cachexia, skeletal muscle weakness, anaemia, depression and cognitive decline. The assessment of COPD is required to determine the severity of the disease, its impact on the health status and the risk of future events (e.g., exacerbations, hospital admissions or death) and this is essential to guide therapy. COPD is treated with inhaled bronchodilators, inhaled corticosteroids, oral theophylline and oral phosphodiesterase-4 inhibitor. Non pharmacological treatment of COPD includes smoking cessation, pulmonary rehabilitation and nutritional support. Lung volume reduction surgery and lung transplantation are advised in selected severe patients. Global strategy for the diagnosis, management and prevention of Chronic Obstructive Pulmonary Disease guidelines recommend influenza and pneumococcal vaccinations.

PMID

23563369 [<https://www.ncbi.nlm.nih.gov/pubmed/?term=23563369>]

Status

Embase

Institution

(Vijayan) Vallabhbhai Patel Chest Institute, University of Delhi, Delhi, India (Vijayan) ICMR Bhopal Memorial Hospital and Research Centre, National Institute for Research in Environmental Health, Bhopal, India

Publisher

Indian Council of Medical Research (Ansari Nagar, P.O. Box 4911, New Delhi 110029, India)

Year of Publication

2013

Link to the Ovid Full Text or citation:

[Click here for full text options](https://ovidsp.ovid.com/ovidweb.cgi?T=JS&CSC=Y&NEWS=N&PAGE=fulltext&D=emed14&AN=368612488)

Link to the External Link Resolver:

[LibKey NHS](https://libkey.io/libraries/2789/openurl?genre=article&sid=OVID:emed14&genre=article&id=pmid:23563369&id=doi:&issn=0971-5916&volume=137&issue=2&spage=251&pages=251-269&date=2013&title=Indian+Journal+of+Medical+Research&atitle=Chronic+obstructive+pulmonary+disease&aulast=Vijayan)

179.

Managing comorbidity in COPD: A difficult task.

Tsiligianni I.G., Kosmas E., van der Molen T., Tzanakis N.

Embase

Current Drug Targets. 14(2) (pp 158-176), 2013. Date of Publication: 2013.

[Review]

AN: 368247007

Chronic obstructive pulmonary disease is a public health problem that results in high morbidity, disability and mortality. Comorbidities are highly prevalent in COPD patients because of aging, common risk factors and pathways, rising mortality, and disability. In this review article we present the most prevalent co-morbidities in COPD patients, we face the issue of multimorbidity and discuss the practical management approach relevant to chest physicians and general practitioners. Issues on comorbidities management according to general guidelines as well as their implications for COPD are raised. The aim is to give clinicians an easy update with specific recommendations for each comorbidity. The implications of several medications used for comorbidities in COPD in terms of benefits, concerns, medication preference, medication avoidance and contraindications are also discussed. © 2013 Bentham Science Publishers.

PMID

23256716 [<https://www.ncbi.nlm.nih.gov/pubmed/?term=23256716>]

Status

Embase

Institution

(Tsiligianni, Tzanakis) Department of Thoracic Medicine, School of Medicine, University of Crete, Heraklion, Crete, Greece (Tsiligianni, van der Molen) Department of General Practice, University Medical Centre of Groningen, Groningen, Netherlands

(Kosmas) Department of Pneumonology, Metropolitan Hospital Athens, Athens, Greece

Publisher

Bentham Science Publishers B.V. (P.O. Box 294, Bussum 1400 AG, Netherlands)

Year of Publication

2013

Link to the Ovid Full Text or citation:

[Click here for full text options](https://ovidsp.ovid.com/ovidweb.cgi?T=JS&CSC=Y&NEWS=N&PAGE=fulltext&D=emed14&AN=368247007)

Link to the External Link Resolver:

[LibKey NHS](https://libkey.io/libraries/2789/openurl?genre=article&sid=OVID:emed14&genre=article&id=pmid:23256716&id=doi:10.2174%2F1389450111314020004&issn=1389-4501&volume=14&issue=2&spage=158&pages=158-176&date=2013&title=Current+Drug+Targets&atitle=Managing+comorbidity+in+COPD%3A+A+difficult+task&aulast=Tsiligianni)

180.

Psychopathology in chronic obstructive pulmonary disease patients with severely impaired health status.

Pommer A., Pouwer F., Denollet J., Meijer J.-W., Van Ranst D., Prins L., Pop V.

Embase

American Journal of Respiratory and Critical Care Medicine. Conference: American Thoracic Society International Conference, ATS 2013. Philadelphia, PA United States. Conference Publication: (var.pagings). 187(MeetingAbstracts) (no pagination), 2013. Date of Publication: 2013.

[Conference Abstract]

AN: 71984522

Rationale: There is a substantial discrepancy between reported symptoms and the number of exacerbations in Chronic Obstructive Pulmonary Disease (COPD) and objectively found abnormal lung-parameters (FEV1, physical-fitness (w-max)). The current study explores the psychological characteristics of COPD patients in a tertiary care rehabilitation center. We hypothesized that patients with mild to moderate COPD would perform better on disease-specific measurements, but would report higher levels of psychopathology compared to more severe COPD patients. Moreover we hypothesized that the number of exacerbations reported during the previous 12 months was highly correlated with psychopathology.

Method(s): During a period of five years, 935 COPD patients entered a tertiary care rehabilitation program. Disease specific measurements were lung-function, physical-fitness (w-max), number of self-reported exacerbations in the year prior to rehabilitation, and health-status (St.George's respiratory questionnaire); psychopathology was measured with the Symptoms CheckList-90 (SCL90). Psychopathology-scores were compared in different subgroups of patients by means of MANOVA and Pearson correlations.

Result(s): Compared to the general population COPD patients reported significantly higher levels of psychopathology on the total SCL90 scale and all its subscales. On three subscales of the SCL90 (sleeping problems, phobic anxiety, and somatization), levels of distress were even similar to those of a psychiatric norm-population. The highest levels of psychopathology were reported by patients with mild/moderate COPD according to the Global Initiative for Chronic Obstructive Lung Disease guidelines (GOLD I-II). Moreover, these patients reported a similar number of exacerbations compared to more severe patients (GOLD III-IV), despite better health-status, lung-function and physical-fitness, after adjusting for age and gender. There was a highly significant correlation (p<0.001 two-tailed) between SCL90 scores and the number of reported exacerbations in both GOLD I (r=0.71) as well as GOLD II patients (r=0.34), with moderate to large effect sizes. Conversely, the number of exacerbations did not correlate at all with objective parameters (lung-function and w-max) in any of the GOLD-classes.

Conclusion(s): This study suggests that the high level of psychopathology found in patients at a tertiary care lung rehabilitation centre may influence the way patients perceive the impact of their disease. Especially in patients with mild/moderate COPD who enter a tertiary care rehabilitation program, psychological characteristics rather than objective physical parameters should be taken into account when starting therapy. Also, when COPD patients do not respond to treatment, it is important to be aware of the possible impact of psychopathology in reporting signs and symptoms as well as the number of exacerbations.

Status

CONFERENCE ABSTRACT

Institution

(Pommer, Pouwer, Denollet, Pop) Tilburg University, Tilburg, Netherlands (Meijer, Van Ranst) Revant Pulmonary Rehabilitation Centre 'Schoondonck', Breda, Netherlands

(Prins) Rehabilitation Centre Heideheuvel, Hilversum, Netherlands

Publisher

American Thoracic Society

Year of Publication

2013

Link to the Ovid Full Text or citation:

[Click here for full text options](https://ovidsp.ovid.com/ovidweb.cgi?T=JS&CSC=Y&NEWS=N&PAGE=fulltext&D=emed14&AN=71984522)

Link to the External Link Resolver:

[LibKey NHS](https://libkey.io/libraries/2789/openurl?genre=article&sid=OVID:emed14&genre=article&id=pmid:&id=doi:&issn=1073-449X&volume=187&issue=MeetingAbstracts&spage=&pages=&date=2013&title=American+Journal+of+Respiratory+and+Critical+Care+Medicine&atitle=Psychopathology+in+chronic+obstructive+pulmonary+disease+patients+with+severely+impaired+health+status&aulast=Pommer)

181.

Pulmonary rehabilitation improves sleep efficiency in patients with chronic obstructive pulmonary disease.

Shah H., Ellstrom K., Fargo R., Dandamudi N.

Embase

American Journal of Respiratory and Critical Care Medicine. Conference: American Thoracic Society International Conference, ATS 2013. Philadelphia, PA United States. Conference Publication: (var.pagings). 187(MeetingAbstracts) (no pagination), 2013. Date of Publication: 2013.

[Conference Abstract]

AN: 71981192

Introduction Chronic Obstructive Pulmonary Disease (COPD) is the third leading cause of mortality in the United States. It is well known that sleep has profound adverse effects on respiration and gas exchange in patients with COPD. However, the effects of COPD on sleep are equally important and not well studied. Patients with COPD have a longer time to sleep onset (latency), frequent arousals and sleep stage shifts, and reduced overall sleep efficiency. Pulmonary rehabilitation (PR) is an important component in the state-of-the-art care of patients with COPD. It improves dyspnea, exercise tolerance, and quality of life. Remarkably, little is known about the effects of PR on sleep. Actigraphy is a simplified alternative to polysomnography to study sleep. It allows objective assessment of sleep for extended periods with minimal inconvenience, and is a useful tool to study sleep for intervention studies. The purpose of this study was to see if PR leads to improved sleep in COPD patients, as measured by actigraphy. Methods This study used a prospective, observational, within-subject design. Patients undergoing pulmonary rehabilitation were invited to participate. After informed consent, patients underwent actigraphy for 4 nights before PR and for 4 nights after completion of PR. A paired t-test was used to assess for difference in sleep parameters before and after PR. The goal was to enroll 30 patients. Sleep measurements obtained by actigraphy included total time in bed, total sleep time, sleep onset latency, sleep efficiency, wake after sleep onset(WASO) time, and number of awakenings. Results To date, ten patients have completed this study. Two patients who were hospitalized or were ill at time of their repeat, post-rehabilitation actigraphy were excluded from analysis. Patients who completed PR had significant improvement in sleep efficiency from 64 +/- 16 % to 81 +/- 6 % (p<.05). In addition, the WASO time also improved from 112 +/- 44 minutes to 45 +/- 24 minutes (p <.05). There was a trend towards improvement in total sleep time from 304 +/- 104 minutes to 379 +/- 83 minutes, though not statistically significant (p =.06). The number of awakenings and sleep onset latency were not different. Conclusion Patients who complete pulmonary rehabilitation have an improvement in sleep quality. Specifically, patients have better overall sleep efficiency and spend less time awake after initially falling asleep.

Status

CONFERENCE ABSTRACT

Institution

(Shah, Ellstrom, Fargo, Dandamudi) VA Loma Linda Health Care System, Loma Linda, CA, United States

Publisher

American Thoracic Society

Year of Publication

2013

Link to the Ovid Full Text or citation:

[Click here for full text options](https://ovidsp.ovid.com/ovidweb.cgi?T=JS&CSC=Y&NEWS=N&PAGE=fulltext&D=emed14&AN=71981192)

Link to the External Link Resolver:

[LibKey NHS](https://libkey.io/libraries/2789/openurl?genre=article&sid=OVID:emed14&genre=article&id=pmid:&id=doi:&issn=1073-449X&volume=187&issue=MeetingAbstracts&spage=&pages=&date=2013&title=American+Journal+of+Respiratory+and+Critical+Care+Medicine&atitle=Pulmonary+rehabilitation+improves+sleep+efficiency+in+patients+with+chronic+obstructive+pulmonary+disease&aulast=Shah)

182.

Exercise and lung cancer survivorship.

Corcoran S.

Embase

Journal of Thoracic Oncology. Conference: 15th World Conference on Lung Cancer. Sydney, NSW Australia. Conference Publication: (var.pagings). 8(SUPPL. 2) (pp S90), 2013. Date of Publication: November 2013.

[Conference Abstract]

AN: 71395870

An estimated 225,000 men and woman will be diagnosed with lung cancer in 2013 (NCI-SEER Data). Approximately 80% of lung cancer patients will be diagnosed with non-small cell lung cancer (NSCLC), and an estimated 25% will present with earlystage or operable disease (Jones 2009). The overall 5 year survival rate for lung cancer is 16%. However, if detected early the survival rate is 53% (ACS 2010). Improvements in surgical techniques, coupled with more effective chemotherapy regimens, have led to significant survival gains for patients with operable disease (Jones 2009). With improved survival rates, long-term treatment sequelae and quality of life (QOL) are gaining increasing attention in terms of post-treatment management of early stage patients. For patients with inoperable or more advanced disease, measures to improve symptoms and QOL are also being evaluated. An individual is considered a cancer survivor from the time of diagnosis, through the balance of his or her life (NCI- Survivorship Definitions 2004). Lung cancer survivors may experience a myriad of long-term and late effects related to their diagnosis and treatment exposures. Symptoms may include dyspnea, fatigue, decreased physical endurance, depression, inability to sleep and weight loss (Anderson 2011). Anticipated age-related losses in physical function and comorbidities such as chronic obstructive pulmonary disease (COPD), ischemic heart disease and hypertension can add to post treatment complications, long term effects and overall recovery. Exercise therapy continues to gain recognition as an effective intervention in cancer rehabilitation. While early studies mainly focused on breast cancer survivors, a growing number of studies have been conducted over the past several years evaluating exercise following a lung cancer diagnosis in the post treatment setting. A review of literature suggests lung cancer patients are good candidates for pulmonary rehabilitation following treatment. Peddle- McIntyre et al describe progressive resistance exercise training, or PRET, as an intervention that has been successful in improving muscular strength, body composition, physical fitness, physical function and QOL in some cancer survivor groups citing, however, that no studies have focused solely on lung cancer survivors. Their prospective study including early stage NSCLS survivors who had undergone surgical resection preliminarily revealed significant improvement in muscle strength, muscular endurance and peak inspiratory pressure. The 6-minute walk distance (6MWD), regarded as a prognostic indicator in certain chronic illnesses, also demonstrated improvements suspected to correlate with preserving body function and delaying onset of mobility limitations. Several studies have demonstrated the benefits of exercise in COPD patients, who are known to experience respiratory symptoms comparable to lung cancer patients. A prospective study conducted by Anderson et al utilized an established COPD rehabilitation program in lung cancer patients to evaluate impact on fitness and QOL. Patients who had undergone pulmonary surgery as well as those who were surgically ineligible (comprising the majority of participants) were included in the study. Interventions included supervised exercise in the clinic setting and instruction on home exercising. Walking was the main element. Results showed improved physical fitness and performance as measured by the Incremental Shuttle Walk Test (ISWT), Endurance Shuttle Walk Test (ESWT) and spirometry after the exercise intervention. Pulmonary function and self repor ted QOL, however, did not demonstrate improvement. Spruit et al (2006) conducted a non-randomized clinical trial in patients who had a history of surgery, chemotherapy and/or radiotherapy evaluating an inpatient rehabilitation program on pulmonary function, 6MWD and peak cycling load. Patients were trained in daily cycling, walking, weight training, and gymnastics (focusing on flexibility and mobility). No change was seen in pulmonary function post intervention. However, significant improvements were seen in the 6MWD and peak cycling load. Symptom scores for dyspnea and fatigue also improved significantly. In focusing on patients with newly diagnosed advanced NSCLC with good performance status, Temel et al conducted a study to determine feasibility of implementing a twice weekly physical therapy (PT) run program using aerobic exercise (bicycle and treadmill) and weight training. Functional capacity as measured by the 6-minute walk test (6MWT), muscle strength, QOL and lung cancer symptoms and fatigue were evaluated. Results included no significant post intervention improvement in QOL or fatigue. Lung cancer symptoms, though, were significantly improved. Additionally, no deterioration in the 6MWT or muscle strength were reported. Lastly, Jones et al (2008) studied the effects of aerobic cycle training on changes in VO2peak and QOL among post surgery NSCLC patients. Results included increased VO2peak, improved QOL and decreased fatigue. Exercise therapy is becoming increasingly acknowledged as an integral component of cancer rehabilitation. The limited literature focusing on lung cancer survivors at different stages post diagnosis support exercise training as safe and well tolerated (Jones 2009). Adherence and ability to complete prescribed programs remains an issue, not surprisingly, for this patient population. While studies thus far have other limitations, they illustrate the need for large randomized trials to better determine the timing, support and specific interventions that will optimally benefit lung cancer survivors.

Status

CONFERENCE ABSTRACT

Institution

(Corcoran) Survivorship Program, Memorial Sloan-Kettering Cancer Center, United States

Publisher

International Association for the Study of Lung Cancer

Year of Publication

2013

Link to the Ovid Full Text or citation:

[Click here for full text options](https://ovidsp.ovid.com/ovidweb.cgi?T=JS&CSC=Y&NEWS=N&PAGE=fulltext&D=emed14&AN=71395870)

Link to the External Link Resolver:

[LibKey NHS](https://libkey.io/libraries/2789/openurl?genre=article&sid=OVID:emed14&genre=article&id=pmid:&id=doi:10.1097%2F01.JTO.0000438438.14562.c8&issn=1556-0864&volume=8&issue=SUPPL.+2&spage=S90&pages=S90&date=2013&title=Journal+of+Thoracic+Oncology&atitle=Exercise+and+lung+cancer+survivorship&aulast=Corcoran)

183.

Respiratory clinic referral patterns to an outpatient pulmonary rehabilitation program.

Hill C.J., Holland A.E., Stodden N., Oliveira C.C., Mcdonald C.F.

Embase

Respirology. Conference: 2013 Annual Scientific Meetings of the Thoracic Society of Australia and New Zealand and the Australian and New Zealand Society of Respiratory Science. Darwin, NT Australia. Conference Publication: (var.pagings). 18(SUPPL. 2) (pp 51), 2013. Date of Publication: April 2013.

[Conference Abstract]

AN: 71011101

Aim To identify barriers to referral to pulmonary rehabilitation (PR) from specialist respiratory outpatient (ROP) clinics. Methods Medical staff were invited to complete a brief checklist for each patient seen in a ROP clinic over a 3 month period at a tertiary hospital in Melbourne. Sleep clinics were excluded. Questions consisted of whether or not the patient was referred to PR; if referral was declined, and why. Results Responses were received for 471 (87%) of 539 appointments. Patients had a mean (SD) age of 60 (16) years and 242 (51%) had a diagnosis of COPD. Other common diagnoses were sleep disorders 140 (29%), asthma 31 (6%), ILD 14 (3%) and chronic cough 8 (2%). Of patients with COPD, referral to PR was made for 31 (13%), a further 25 (5%) declined. One non- COPD patient with dyspnoea and obesity was referred. A total of 211 patients with COPD were not referred to PR for the following reasons reported by their doctor: Conclusion 29% of patients with COPD attending a ROP clinic were referred to PR or were already enrolled. The main reasons for non-referral was lack of perceived benefit by patients, or PR deemed not applicable by doctors. Previously reported patient-related barriers to attendance such as access or transport were not significant factors in referral. Patients with chronic respiratory disease other than COPD were not referred to PR. (Table Presented).

Status

CONFERENCE ABSTRACT

Institution

(Hill, Stodden, Mcdonald) Austin Health, VIC 3084, Australia (Hill, Holland, Mcdonald) Institute for Breathing and Sleep, VIC 3084, Australia

(Holland, Oliveira) LaTrobe University, VIC 3084, Australia

(Holland) Alfred Health, VIC 3004, Australia

(Oliveira, Mcdonald) University of Melbourne, VIC 3052, Australia

Publisher

Blackwell Publishing

Year of Publication

2013

Link to the Ovid Full Text or citation:

[Click here for full text options](https://ovidsp.ovid.com/ovidweb.cgi?T=JS&CSC=Y&NEWS=N&PAGE=fulltext&D=emed14&AN=71011101)

Link to the External Link Resolver:

[LibKey NHS](https://libkey.io/libraries/2789/openurl?genre=article&sid=OVID:emed14&genre=article&id=pmid:&id=doi:10.1111%2Fresp.12046&issn=1323-7799&volume=18&issue=SUPPL.+2&spage=51&pages=51&date=2013&title=Respirology&atitle=Respiratory+clinic+referral+patterns+to+an+outpatient+pulmonary+rehabilitation+program&aulast=Hill)

184.

Effect of pulmonary rehabilitation on sleep as measured by actigraphy. (repeat)

Shah H., Dandamudi N., Fargo R., Ellstrom K.

Embase

Journal of Investigative Medicine. Conference: American Federation for Medical Research Western Regional Meeting, AFMR 2013. Carmel, CA United States. Conference Publication: (var.pagings). 61(1) (pp 183), 2013. Date of Publication: January 2013.

[Conference Abstract]

AN: 70993709

Purpose of Study: Chronic Obstructive Pulmonary Disease (COPD) is now the third leading cause of mortality. Sleep has profound adverse effects on respiration and gas exchange in patients with COPD. Patients with COPD have a longer time to sleep onset (latency), frequent arousals, frequent sleep stage shifts, and reduced sleep efficiency. Pulmonary rehabilitation (PR) is the standard of care for patients suffering from COPD. It improves dyspnea, exercise tolerance, and quality of life. Remarkably, the effect of PR on sleep has not been studied. Actigraphy is a non-invasive alternative to polysomnography to study sleep. It allows objective assessment of sleep for extended periods with minimal inconvenience, and has become a useful tool to study sleep for intervention studies. The purpose of this study was to see if PR leads to improved sleep in COPD patients as measured by actigraphy. Methods Used: This study was a prospective, observational, within-subject design. After informed consent, patients who were selected for our nationally accredited rehabilitation program had actigraphy done for 4 nights before and then after completion of the program. A repeated measures paired t-test was used to assess for difference in sleep parameters before and after PR. Sleep measurements obtained by actigraphy included total time in bed, total sleep time, onset latency, sleep efficiency, wake after sleep onset (WASO), and number of awakenings. Summary of Results: Ten patients completed PR. Two patients who were hospitalized or were ill at time of repeat actigraphy were excluded. Data presented are from the remaining 8 patients. Patients who completed PR had a significant improvement in sleep efficiency (81% vs 64%, p <.05) and wake after sleep onset (45 minutes vs. 111 minutes, p <.05). There was a trend towards improvement in total sleep time from 304 to 379 minutes, although not statistically significant. (p=0.06). The number of awakenings and sleep onset latency were not different.

Conclusion(s): Patients who complete PR have improvement in their sleep quality. More specifically, patients have better overall sleep efficiency and spend less time awake at night after initially falling asleep. Thus, improved sleep is another benefit of pulmonary rehabilitation.

Status

CONFERENCE ABSTRACT

Institution

(Shah, Dandamudi, Fargo, Ellstrom) VA Loma Linda Healthcare System, Loma Linda, CA, United States (Shah, Dandamudi, Fargo) Loma Linda University, Medical Center, Loma Linda, CA, United States

Publisher

Lippincott Williams and Wilkins

Year of Publication

2013

Link to the Ovid Full Text or citation:

[Click here for full text options](https://ovidsp.ovid.com/ovidweb.cgi?T=JS&CSC=Y&NEWS=N&PAGE=fulltext&D=emed14&AN=70993709)

Link to the External Link Resolver:

[LibKey NHS](https://libkey.io/libraries/2789/openurl?genre=article&sid=OVID:emed14&genre=article&id=pmid:&id=doi:10.231%2FJIM.0b013e31827d3ac9&issn=1081-5589&volume=61&issue=1&spage=183&pages=183&date=2013&title=Journal+of+Investigative+Medicine&atitle=Effect+of+pulmonary+rehabilitation+on+sleep+as+measured+by+actigraphy&aulast=Shah)

185.

Respiratory diseases and muscle dysfunction.

Gea J., Casadevall C., Pascual S., Orozco-Levi M., Barreiro E.

Embase

Expert Review of Respiratory Medicine. 6(1) (pp 75-90), 2012. Date of Publication: February 2012.

[Review]

AN: 364175118

Many respiratory diseases lead to impaired function of skeletal muscles, influencing quality of life and patient survival. Dysfunction of both respiratory and limb muscles in chronic obstructive pulmonary disease has been studied in depth, and seems to be caused by the complex interaction of general (inflammation, impaired gas exchange, malnutrition, comorbidity, drugs) and local factors (changes in respiratory mechanics and muscle activity, and molecular events). Some of these factors are also present in cystic fibrosis and asthma. In obstructive sleep apnea syndrome, repeated exposure to hypoxia and the absence of reparative rest are believed to be the main causes of muscle dysfunction. Deconditioning appears to be crucial for the functional impairment observed in scoliosis. Finally, cachexia seems to be the main mechanism of muscle dysfunction in advanced lung cancer. A multidimensional therapeutic approach is recommended, including pulmonary rehabilitation, an adequate level of physical activity, ventilatory support and nutritional interventions. © 2012 Expert Reviews Ltd.

PMID

22283581 [<https://www.ncbi.nlm.nih.gov/pubmed/?term=22283581>]

Status

Embase

Institution

(Gea, Casadevall, Pascual, Orozco-Levi, Barreiro) Servei de Pneumologia, Hospital Del Mar - IMIM, CIBER de Enfermedades Respiratorias (CIBERES) ISC III, Barcelona, Catalunya, Spain

Publisher

Expert Reviews Ltd.

Year of Publication

2012

Link to the Ovid Full Text or citation:

[Click here for full text options](https://ovidsp.ovid.com/ovidweb.cgi?T=JS&CSC=Y&NEWS=N&PAGE=fulltext&D=emed13&AN=364175118)

Link to the External Link Resolver:

[LibKey NHS](https://libkey.io/libraries/2789/openurl?genre=article&sid=OVID:emed13&genre=article&id=pmid:22283581&id=doi:10.1586%2Fers.11.81&issn=1747-6348&volume=6&issue=1&spage=75&pages=75-90&date=2012&title=Expert+Review+of+Respiratory+Medicine&atitle=Respiratory+diseases+and+muscle+dysfunction&aulast=Gea)

186.

Chronic obstructive pulmonary disease: A review of nonpharmacologic therapies.

Srinivasan M., Shafazand S.

Embase

Journal of Clinical Outcomes Management. 19(4) (pp 167-177), 2012. Date of Publication: April 2012.

[Review]

AN: 364674591

* Objective: To provide an evidence-based review of the important nonpharmacologic therapies in the care of patients with chronic obstructive pulmonary disease (COPD). * Methods: Literature review. * Results: COPD, including emphysema and chronic bronchitis, is the fourth leading cause of death in the United States. COPD represents a significant economic burden both for individuals and for society. COPD is a chronic, systemic disease requiring a multidisciplinary and multimodal approach to its care and management. Treatment approaches include a combination of pharmacologic and nonpharmacologic therapies applied in a step-wise fashion based on airflow limitation severity criteria and patient symptoms. Smoking cessation, vaccinations, maintaining physical activity, adequate nutrition, reducing occupational and environmental exposures, and ensuring psychological health are the cornerstones of longterm management of COPD. *

Conclusion(s): The care of patients with COPD requires a multidisciplinary approach that involves not only pharmacologic therapies but also meticulous attention to the physical, social, and psychological well being of the individual. An individualized prescription of nonpharmacologic therapies is necessary to address the systemic disease that is COPD.

Status

Embase

Institution

(Srinivasan, Shafazand) Department of Medicine, Division of Pulmonary, Critical Care and Sleep Medicine, University of Miami, Miami, FL, United States

Publisher

Turner White Communications Inc. (125 Strafford Avenue, Suite 220, Wayne PA 19087-3391, United States)

Year of Publication

2012

Link to the Ovid Full Text or citation:

[Click here for full text options](https://ovidsp.ovid.com/ovidweb.cgi?T=JS&CSC=Y&NEWS=N&PAGE=fulltext&D=emed13&AN=364674591)

Link to the External Link Resolver:

[LibKey NHS](https://libkey.io/libraries/2789/openurl?genre=article&sid=OVID:emed13&genre=article&id=pmid:&id=doi:&issn=1079-6533&volume=19&issue=4&spage=167&pages=167-177&date=2012&title=Journal+of+Clinical+Outcomes+Management&atitle=Chronic+obstructive+pulmonary+disease%3A+A+review+of+nonpharmacologic+therapies&aulast=Srinivasan)

187.

Transition of patients with COPD across different care settings: challenges and opportunities for hospitalists.

Chuang C.

Embase

Hospital practice (1995). 40(1) (pp 176-185), 2012. Date of Publication: Feb 2012.

[Article]

AN: 364818688

Hospitalists play an important role in treating current and preventing future acute exacerbations of chronic obstructive pulmonary disease (AECOPD), which are associated with high rates of medical resource use and morbidity. Comprehensive admission screening and diagnostic tests are important in enabling hospitalists to reliably identify patients with AECOPD, the severity of the episode, and related issues that may prolong patients' hospital length of stay. Recurring exacerbations, especially those that require repeated acute care, can reduce physical activity and accelerate pulmonary decline and risk of death. Recommended pharmacotherapies for AECOPD should include short-acting bronchodilators, systemic corticosteroids, and appropriate antibiotics in cases of suspected bacterial infection. Patients with demonstrable hypoxemia or respiratory failure may benefit from oxygen and/or ventilatory support. Long-term disease management with the goal of preventing future exacerbations should include ongoing emphasis toward smoking cessation and up-to-date vaccination, in addition to prescribing maintenance pharmacotherapies in accordance with respiratory treatment guidelines. Additional benefits may be derived from nonpharmacologic therapies, such as pulmonary rehabilitation, weight-loss recommendations, and treatment of obstructive sleep dyspnea when present. Effective communication among members of the inpatient and outpatient health care teams, the patient, and his or her caregivers is an important aspect of care transitions. Hospital discharge summaries should be transmitted to the patient's primary care physician and be readily available at the first follow-up visit. Discharge coaches and other allied health care providers can aid hospitalists in reinforcing self-management skills and patient education, and in emphasizing the importance of follow-up visits. Recent findings suggest that health and cost benefits are associated with improved COPD management. This article focuses on the pivotal role of the hospitalist in promoting and facilitating the steps toward improving quality outcomes and transitions of care for patients with COPD.

PMID

22406893 [<https://www.ncbi.nlm.nih.gov/pubmed/?term=22406893>]

Institution

(Chuang) HealthCare Partners Medical Group, Torrance, CA 90502, USA.

Year of Publication

2012

Link to the Ovid Full Text or citation:

[Click here for full text options](https://ovidsp.ovid.com/ovidweb.cgi?T=JS&CSC=Y&NEWS=N&PAGE=fulltext&D=emed13&AN=364818688)

Link to the External Link Resolver:

[LibKey NHS](https://libkey.io/libraries/2789/openurl?genre=article&sid=OVID:emed13&genre=article&id=pmid:22406893&id=doi:10.3810%2Fhp.2012.02.958&issn=2154-8331&volume=40&issue=1&spage=176&pages=176-185&date=2012&title=Hospital+practice+(1995)&atitle=Transition+of+patients+with+COPD+across+different+care+settings%3A+challenges+and+opportunities+for+hospitalists&aulast=Chuang)

188.

Modifying the course of chronic obstructive pulmonary disease: Looking beyond the FEV1.

Zuwallack R.L., Nici L.

Embase

COPD: Journal of Chronic Obstructive Pulmonary Disease. 9(6) (pp 637-648), 2012. Date of Publication: December 2012.

[Review]

AN: 366324036

COPD is defined by airflow limitation that is not fully reversible and is usually progressive. Thus, airflow obstruction (measured as FEV1) has traditionally been used as the benchmark defining disease modification with therapy. However, COPD exacerbations and extrapulmonary effects are common and burdensome and generally become more prominent as the disease progresses. Therefore, disease progression should be broader than FEV1 alone. Interventions that reduce the frequency or severity of exacerbations or ameliorate extrapulmonary effects should also be considered disease modifiers. A narrow focus on FEV1 will fail to capture all the beneficial effects of therapy on disease modification. Although smoking cessation has been unequivocally demonstrated to slow the rate of FEV1 decline, inhaled corticosteroid-long- acting bronchodilator therapy may also have modest effects according to post hoc analysis. Maintenance pharmacotherapy with inhaled long-acting anti-muscarinic or -adrenergic agents or combined -adrenergic-inhaled corticosteroid reduces symptoms, improves lung function, reduces the frequency of exacerbations, and improves exercise capacity and HRQL. Pulmonary rehabilitation reduces symptom burden, increases exercise capacity, improves HRQL, and reduces health care utilization, probably through reducing the severity of exacerbations. Smoking cessation, lung volume reduction surgery, inhaled maintenance pharmacotherapy, and pulmonary rehabilitation administered in the post-exacerbation period may reduce mortality in COPD. These improvements over multiple outcome areas and over relatively long durations suggest that disease modification is indeed possible with existing therapies for COPD. Therefore, therapeutic nihilism in COPD is no longer warranted. © 2012 Informa Healthcare USA, Inc.

PMID

22958136 [<https://www.ncbi.nlm.nih.gov/pubmed/?term=22958136>]

Status

Embase

Institution

(Zuwallack) Section of Pulmonary and Critical Care, St. Francis Hospital and Medical Center, 114 Woodland Street, Hartford, CT 06105, United States (Nici) Providence VA Medical Center, Providence, RI, United States

Publisher

Informa Healthcare (52 Vanderbilt Ave., New York 10017, United States)

Year of Publication

2012

Link to the Ovid Full Text or citation:

[Click here for full text options](https://ovidsp.ovid.com/ovidweb.cgi?T=JS&CSC=Y&NEWS=N&PAGE=fulltext&D=emed13&AN=366324036)

Link to the External Link Resolver:

[LibKey NHS](https://libkey.io/libraries/2789/openurl?genre=article&sid=OVID:emed13&genre=article&id=pmid:22958136&id=doi:10.3109%2F15412555.2012.710668&issn=1541-2555&volume=9&issue=6&spage=637&pages=637-648&date=2012&title=COPD%3A+Journal+of+Chronic+Obstructive+Pulmonary+Disease&atitle=Modifying+the+course+of+chronic+obstructive+pulmonary+disease%3A+Looking+beyond+the+FEV1&aulast=Zuwallack)

189.

Treatments for anxiety and depression in patients with chronic obstructive pulmonary disease: A literature review.

Cafarella P.A., Effing T.W., Usmani Z.-A., Frith P.A.

Embase

Respirology. 17(4) (pp 627-638), 2012. Date of Publication: May 2012.

[Review]

AN: 364647933

Chronic obstructive pulmonary disease (COPD) is a serious contemporary health issue. Psychological co-morbidities such as anxiety and depression are common in COPD. Current evidence for treatment options to reduce anxiety and depression in patients with COPD was examined. There is evidence available for the efficacy of pharmacological treatments, cognitive behavioural therapy, pulmonary rehabilitation, relaxation therapy and palliative care in COPD. Therapeutic modalities that have not been proven effective in decreasing anxiety and depression in COPD, but which have theoretical potential among patients, include interpersonal psychotherapy, self-management programmes, more extensive disease management programmes, supportive therapy and self-help groups. Besides pulmonary rehabilitation that is only available for a small percentage of patients, management guidelines make scant reference to other options for the treatment of mental health problems. The quantity and quality of research on mental health treatments in COPD have historically been insufficient to support their inclusion in COPD treatment guidelines. In this review, recommendations regarding assessment, treatment and future research in this important field were made. © 2012 Asian Pacific Society of Respirology.

PMID

22309179 [<https://www.ncbi.nlm.nih.gov/pubmed/?term=22309179>]

Status

Embase

Institution

(Cafarella, Effing, Frith) Department of Respiratory Medicine, Repatriation General Hospital, Daw Park, Daws Road, Adelaide, SA 5041, Australia (Usmani) Adelaide Institute for Sleep Health, Repatriation General Hospital, Australia

(Effing, Frith) School of Medicine, Flinders University, Adelaide, SA, Australia

Publisher

Blackwell Publishing (550 Swanston Street, Carlton South VIC 3053, Australia)

Year of Publication

2012

Link to the Ovid Full Text or citation:

[Click here for full text options](https://ovidsp.ovid.com/ovidweb.cgi?T=JS&CSC=Y&NEWS=N&PAGE=fulltext&D=emed13&AN=364647933)

Link to the External Link Resolver:

[LibKey NHS](https://libkey.io/libraries/2789/openurl?genre=article&sid=OVID:emed13&genre=article&id=pmid:22309179&id=doi:10.1111%2Fj.1440-1843.2012.02148.x&issn=1323-7799&volume=17&issue=4&spage=627&pages=627-638&date=2012&title=Respirology&atitle=Treatments+for+anxiety+and+depression+in+patients+with+chronic+obstructive+pulmonary+disease%3A+A+literature+review&aulast=Cafarella)

190.

Mechanisms of non-pharmacologic adjunct therapies used during exercise in COPD.

Moga A.M., De Marchie M., Saey D., Spahija J.

Embase

Respiratory Medicine. 106(5) (pp 614-626), 2012. Date of Publication: May 2012.

[Review]

AN: 364521847

Individuals with chronic obstructive pulmonary disease (COPD) are often limited in their ability to perform exercise due to a heightened sense of dyspnea and/or the occurrence of leg fatigue associated with a reduced ventilatory capacity and peripheral skeletal muscle dysfunction, respectively. Pulmonary rehabilitation programs have been shown to improve exercise tolerance and health related quality of life. Additional therapeutic approaches such as non-invasive ventilatory support (NIVS), heliox (He-O2) and supplemental oxygen have been used as non-pharmacologic adjuncts to exercise to enhance the ability of patients with COPD to exercise at a higher exercise-intensity and thus improve the physiological benefits of exercise. The purpose of the current review is to examine the pathophysiology of exercise limitation in COPD and to explore the physiological mechanisms underlying the effect of the adjunct therapies on exercise in patients with COPD. This review indicates that strategies that aim to unload the respiratory muscles and enhance oxygen saturation during exercise alleviate exercise limiting factors and improve exercise performance in patients with COPD. However, available data shows significant variability in the effectiveness across patients. Further research is needed to identify the most appropriate candidates for these forms of therapies. © 2011 Elsevier Ltd. All rights reserved.

PMID

22341681 [<https://www.ncbi.nlm.nih.gov/pubmed/?term=22341681>]

Status

Embase

Institution

(Moga, Spahija) School of Physical and Occupational Therapy, McGill University, 3654 Promenade Sir William Osler, Montreal, QC H3G 1Y5, Canada (Moga, Spahija) Hopital du Sacre-Coeur de Montreal, Axe de Recherche en Sante Respiratoire, 5400 boul. Gouin Ouest, Montreal, QC H4J 1C5, Canada

(De Marchie) Department of Adult Critical Care, Sir Mortimer B. Davis Jewish General Hospital, McGill University, Montreal, QC, Canada

(Saey) Centre de Recherche, Institut Universitaire de Cardiologie et de Pneumologie de Quebec, Universite Laval, QC, Canada

(Spahija) Center for Interdisciplinary Research in Rehabilitation in Montreal, Jewish Rehabilitation Hospital, 3205, Place Alton-Goldbloom, Laval, QC H7V 1J1, Canada

Publisher

W.B. Saunders Ltd (32 Jamestown Road, London NW1 7BY, United Kingdom)

Year of Publication

2012

Link to the Ovid Full Text or citation:

[Click here for full text options](https://ovidsp.ovid.com/ovidweb.cgi?T=JS&CSC=Y&NEWS=N&PAGE=fulltext&D=emed13&AN=364521847)

Link to the External Link Resolver:

[LibKey NHS](https://libkey.io/libraries/2789/openurl?genre=article&sid=OVID:emed13&genre=article&id=pmid:22341681&id=doi:10.1016%2Fj.rmed.2012.01.006&issn=0954-6111&volume=106&issue=5&spage=614&pages=614-626&date=2012&title=Respiratory+Medicine&atitle=Mechanisms+of+non-pharmacologic+adjunct+therapies+used+during+exercise+in+COPD&aulast=Moga)

191.

Changing demographics of patients with copd admitted to a sub-acute pulmonary rehabilitation unit.

Prefontaine D., Dajczman E., Fiter R., Cohen L., Baltzan M., Wolkove N.

Embase

American Journal of Respiratory and Critical Care Medicine. Conference: American Thoracic Society International Conference, ATS 2012. San Francisco, CA United States. Conference Publication: (var.pagings). 185(MeetingAbstracts) (no pagination), 2012. Date of Publication: 2012.

[Conference Abstract]

AN: 71989956

Introduction: Variations in the chronic obstructive pulmonary disease (COPD) population admitted for sub-acute care and pulmonary rehabilitation were observed clinically. We aimed to retrospectively characterize what specific changes in demographics and severity of disease occurred between the years 2000 and 2010.

Method(s): Patients with a primary diagnosis of COPD admitted to the respiratory in 2000 and 2010 were identified through a database search. The age at admission, gender, length of stay, and co-morbidities as per International Classification of Diseases coding system (ICD9 and ICD10 for 2000 and 2010, respectively) were obtained. Charts were reviewed for patient origin (home vs. hospital transfer), smoking history, lung function of long-term oxygen therapy (LTOT) on admission, body mass index (BMI, WHO standards), and 6-minute walk distance (6MWD) pre- and post-rehabilitation.

Result(s): Patients with COPD admitted in 2010 were younger (69.5 vs. 72.5 years of age, p<0.01), predominantly female (60.1 vs. 49.8%, p=0.02), and had shorter length of stay than those in 2000. Dyslipidemia, hypertension, diabetes, anemia, sleep apnea and history of malignancy were more prevalent among patients from 2010 compared with 2000 (p<0.05). In 2010, 58.2% of patients were admitted from home compared with 46.8% in 2000 (p<0.01). Smoking history, FEV1 % of predicted value (33.7 vs. 31.4, p=0.13), and presence of LTOT were similar for patients admitted in 2010 vs. 2000. Patients admitted in 2010 had higher BMI (27.0 vs. 25.2, p=0.05), and greater incidence of obesity. Patients had higher age-adjusted 6MWD on admission (42.8 vs. 33.7% of predicted value, p<0.01) as well as a clinically-important post-rehabilitation 6MWD change in 2010 compared with results in 2000.

Conclusion(s): Significant demographic changes in age, sex, functional capacity and co-morbidity profiles were identified in this retrospective study. Younger patients and more women were admitted for care in 2010. Many of the epidemiologic trends in society were seen in this comparison of COPD populations, including obesity and the increased prevalence of diseases associated with metabolic syndrome. Trends in patient origin and functional status may result from differences in discharge planning, home care and disease management strategies over time.

Status

CONFERENCE ABSTRACT

Institution

(Prefontaine, Dajczman, Fiter, Cohen, Baltzan, Wolkove) Mount Sinai Hospital Center, Montreal, QC, Canada

Publisher

American Thoracic Society

Year of Publication

2012

Link to the Ovid Full Text or citation:

[Click here for full text options](https://ovidsp.ovid.com/ovidweb.cgi?T=JS&CSC=Y&NEWS=N&PAGE=fulltext&D=emed13&AN=71989956)

Link to the External Link Resolver:

[LibKey NHS](https://libkey.io/libraries/2789/openurl?genre=article&sid=OVID:emed13&genre=article&id=pmid:&id=doi:&issn=1073-449X&volume=185&issue=MeetingAbstracts&spage=&pages=&date=2012&title=American+Journal+of+Respiratory+and+Critical+Care+Medicine&atitle=Changing+demographics+of+patients+with+copd+admitted+to+a+sub-acute+pulmonary+rehabilitation+unit&aulast=Prefontaine)

192.

Nocturnal oxygen desaturation in patients with COPD: The impact on cognitive function, quality of life, fatigue and depression?.

Prefontaine D., Scott A.S., Dajczman E., Baltzan M., Wolkove N.

Embase

American Journal of Respiratory and Critical Care Medicine. Conference: American Thoracic Society International Conference, ATS 2012. San Francisco, CA United States. Conference Publication: (var.pagings). 185(MeetingAbstracts) (no pagination), 2012. Date of Publication: 2012.

[Conference Abstract]

AN: 71988631

Introduction/rationale: The importance of nocturnal oxygen desaturation (NOD) in patients with chronic obstructive pulmonary disease (COPD) are still debated. Hypoxemia in COPD patients may cause neuropsychological impairment, yet cognitive screening is rarely part of their routine assessment. Patients with COPD also report reduced quality of life (QoL), increased fatigue and depressive symptoms. We aimed to determine whether COPD patients with NOD display altered cognitive function, QoL, fatigue and depressive symptoms compared to patients who do not desaturate during their sleep.

Method(s): Fourty patients with COPD were enrolled from a sub-acute pulmonary rehabilitation unit. Participants determined to be clinically stable completed overnight oximetry (>6h) on room air, spirometric evaluation, Mini-Mental State Examination (MMSE), Montreal Cognitive Assessment (MoCA) test, St. George Respiratory Questionnaire, Epworth Sleepiness Score, Manchester COPD Fatigue Scale and the short Geriatric Depression Scale. Participants with significant NOD spent >30% of their sleep time with a saturation <90%.

Result(s): COPD participants with NOD scored significantly lower on the MMSE than those who do not desaturate (26.1 vs. 27.7; p=0.03), but the percentage of participants with clinically-significant cognitive impairment remained similar in both groups (38.1 vs. 22.2%; p=0.32). Using the MoCA, both groups had lower mean scores (22.3 and 22.4; p=0.93) and a high proportion of individuals with cognitive impairment (76.2 vs. 66.7%; p=0.72). There was no difference between COPD patients with/without NOD on the basis of QoL data (p=0.93), daytime sleepiness (p=0.17), fatigue (p=0.57) or depressive symptoms (p=0.84).

Conclusion(s): Despite limited sample size, this study indicates a lack of greater cognitive impairment in COPD patients with NOD compared to those without overnight desaturation. There was also no difference in QoL, fatigue and depressive symptoms. Our comparison of two validated screening tools to measure cognition in COPD participants demonstrates a striking difference in their ability to detect impairment which could be missed by using the MMSE alone. These results support the importance of thoroughly assessing cognition in patients with COPD as it potentially impacts on patient education and self-management strategies.

Status

CONFERENCE ABSTRACT

Institution

(Prefontaine, Scott, Dajczman, Baltzan, Wolkove) Mount Sinai Hospital Center, Montreal, QC, Canada

Publisher

American Thoracic Society

Year of Publication

2012

Link to the Ovid Full Text or citation:

[Click here for full text options](https://ovidsp.ovid.com/ovidweb.cgi?T=JS&CSC=Y&NEWS=N&PAGE=fulltext&D=emed13&AN=71988631)

Link to the External Link Resolver:

[LibKey NHS](https://libkey.io/libraries/2789/openurl?genre=article&sid=OVID:emed13&genre=article&id=pmid:&id=doi:&issn=1073-449X&volume=185&issue=MeetingAbstracts&spage=&pages=&date=2012&title=American+Journal+of+Respiratory+and+Critical+Care+Medicine&atitle=Nocturnal+oxygen+desaturation+in+patients+with+COPD%3A+The+impact+on+cognitive+function%2C+quality+of+life%2C+fatigue+and+depression%3F&aulast=Prefontaine)

193.

Defining Patient-Reported Outcomes in Chronic Obstructive Pulmonary Disease. The Patient-Centered Experience.

Singer J.P., Yusen R.D.

Embase

Medical Clinics of North America. 96(4) (pp 767-787), 2012. Date of Publication: July 2012.

[Review]

AN: 365248621

Chronic obstructive pulmonary disease (COPD) causes significant morbidity and mortality. Patients with COPD experience impaired health-related quality of life (HRQOL) and disability. However, many challenges exist in the evolving field of HRQOL measurement and interpretation. This article defines HRQOL, discusses instruments used to measure HRQOL, and reviews related literature. © 2012 Elsevier Inc.

PMID

22793944 [<https://www.ncbi.nlm.nih.gov/pubmed/?term=22793944>]

Status

Embase

Institution

(Singer) Div. of Pulmonary, Critical Care, Allergy and Sleep Medicine and Cardiovascular Research Institute, University of California, San Francisco, 350 Parnassus, Suite 609, San Francisco, CA 94117, United States (Yusen) Divisions of Pulmonary and Critical Care Medicine and General Medical Sciences, Washington University, St Louis, MO, United States

Publisher

W.B. Saunders (Independence Square West, Philadelphia PA 19106-3399, United States)

Year of Publication

2012

Link to the Ovid Full Text or citation:

[Click here for full text options](https://ovidsp.ovid.com/ovidweb.cgi?T=JS&CSC=Y&NEWS=N&PAGE=fulltext&D=emed13&AN=365248621)

Link to the External Link Resolver:

[LibKey NHS](https://libkey.io/libraries/2789/openurl?genre=article&sid=OVID:emed13&genre=article&id=pmid:22793944&id=doi:10.1016%2Fj.mcna.2012.05.005&issn=0025-7125&volume=96&issue=4&spage=767&pages=767-787&date=2012&title=Medical+Clinics+of+North+America&atitle=Defining+Patient-Reported+Outcomes+in+Chronic+Obstructive+Pulmonary+Disease.+The+Patient-Centered+Experience&aulast=Singer)

194.

Contribution of the environment and comorbidities to chronic obstructive pulmonary disease phenotypes.

Martinez C.H., Han M.K.

Embase

Medical Clinics of North America. 96(4) (pp 713-727), 2012. Date of Publication: July 2012.

[Review]

AN: 365248617

Chronic obstructive pulmonary disease (COPD) is characterized by significant disease heterogeneity. This heterogeneity suggests that various influences, including environmental and biological factors, probably contribute to the disease, but the validation of specific phenotypes has been problematic. This article reviews differences in the presentation and progression of COPD in relation to environmental risk factors and comorbidities. The clustering of certain comorbidities in COPD could help to identify patients who share similar inflammatory pathways. New research covering large patient populations with extensive clinical and biological characterization, and advanced analytical methods can expand the potential to identify COPD phenotypes and develop targeted therapies. © 2012 Elsevier Inc.

PMID

22793940 [<https://www.ncbi.nlm.nih.gov/pubmed/?term=22793940>]

Status

Embase

Institution

(Martinez, Han) Division of Pulmonary and Critical Care Medicine, University of Michigan Health System, 1500 East Medical Center Drive, 3916 Taubman Center, Ann Arbor, MI 48109-5360, United States

Publisher

W.B. Saunders (Independence Square West, Philadelphia PA 19106-3399, United States)

Year of Publication

2012

Link to the Ovid Full Text or citation:

[Click here for full text options](https://ovidsp.ovid.com/ovidweb.cgi?T=JS&CSC=Y&NEWS=N&PAGE=fulltext&D=emed13&AN=365248617)

Link to the External Link Resolver:

[LibKey NHS](https://libkey.io/libraries/2789/openurl?genre=article&sid=OVID:emed13&genre=article&id=pmid:22793940&id=doi:10.1016%2Fj.mcna.2012.02.007&issn=0025-7125&volume=96&issue=4&spage=713&pages=713-727&date=2012&title=Medical+Clinics+of+North+America&atitle=Contribution+of+the+environment+and+comorbidities+to+chronic+obstructive+pulmonary+disease+phenotypes&aulast=Martinez)

195.

Oxygen desaturation during a 6 min walk test is a sign of nocturnal hypoxemia.

Scott A.S., Baltzan M.A., Chan R., Wolkove N.

Embase

Canadian Respiratory Journal. 18(6) (pp 333-337), 2011. Date of Publication: November-December 2011.

[Article]

AN: 364710964

BACKGROUND/OBJECTIVES: Patients with chronic obstructive pulmonary disease (COPD) may experience sleep disordered breathing with nocturnal desaturation. An exploratory study was performed to determine whether any commonly measured clinical parameters were useful in predicting nocturnal desaturation in patients with COPD. A validation study was subsequently performed to confirm the utility of the parameter identified in the exploratory study as most useful in this regard.

METHOD(S): A total of 103 (exploratory cohort) and 200 (validation cohort) consecutive patients with COPD admitted for pulmonary rehabilitation were evaluated. Standard outcome measures including nocturnal oximetry and the 6 min walk test (6MWT) on room air with continuous pulse oximetry were assessed. Patients with sleep apnea or those undergoing long-term oxygen therapy were excluded.

RESULT(S): In the exploratory study, the mean (+/- SD) patient age was 70+/-9.9 years, with forced expiratory volume in 1 s of 0.76+/-0.34 L, which was 36+/-16% of predicted. Body mass index, arterial oxygen tension, oxygen saturation by pulse oximetry at rest and during the 6MWT all demonstrated significant correlations with percentage of time spent with a saturation <90%. When the lowest pulse oximetry during the 6MWT was <=88%, 10 of 21 patients demonstrated a saturation <90% for at least 30% of sleep time. This measure yielded a positive likelihood ratio of 3.77 (95% CI 1.87 to 7.62) compared with those who did not reach this threshold value. The validation study confirmed similar detection characteristics.

CONCLUSION(S): Results from the present study suggest that monitoring oxygen saturation changes during a 6MWT is useful in helping to identify COPD patients who may experience significant nocturnal desaturation. ©2011 Pulsus Group Inc. All rights reserved.

PMID

22187689 [<https://www.ncbi.nlm.nih.gov/pubmed/?term=22187689>]

Status

Embase

Institution

(Scott, Baltzan, Wolkove) Reasearch Department, Mount Sinai Hospital Center, McGill University, Montreal, QC, Canada (Chan) Faculty of Medicine, School of Physical and Occupational Therapy, McGill University, Montreal, QC, Canada

Publisher

Hindawi Limited

Year of Publication

2011

Link to the Ovid Full Text or citation:

[Click here for full text options](https://ovidsp.ovid.com/ovidweb.cgi?T=JS&CSC=Y&NEWS=N&PAGE=fulltext&D=emed12&AN=364710964)

Link to the External Link Resolver:

[LibKey NHS](https://libkey.io/libraries/2789/openurl?genre=article&sid=OVID:emed12&genre=article&id=pmid:22187689&id=doi:10.1155%2F2011%2F242636&issn=1198-2241&volume=18&issue=6&spage=333&pages=333-337&date=2011&title=Canadian+Respiratory+Journal&atitle=Oxygen+desaturation+during+a+6+min+walk+test+is+a+sign+of+nocturnal+hypoxemia&aulast=Scott)

196.

Series "novelties in pulmonary rehabilitation": Determinants of success.

Garrod R., Malerba M., Crisafulli E.

Embase

European Respiratory Journal. 38(5) (pp 1215-1218), 2011. Date of Publication: 01 Nov 2011.

[Article]

AN: 362860682

In chronic obstructive pulmonary disease (COPD) patients, pulmonary rehabilitation is a nonpharmacological intervention aimed at improving physical exercise tolerance, dyspnoea and perceived quality of life. However, identifying predictors of clinical response and which patients achieve benefit remains a difficult question to answer with no conclusive data available. Baseline characteristics of COPD patients, such as degree of breathlessness, body weight and arterial partial pressure of oxygen, generally appear to be too direct to have a correlation with improvement of post-rehabilitation outcomes. Furthermore, some additional benefits of patients treated with rehabilitation are simply not detected by usual measures (social interaction, sleep quality and confidence). Although there are some data suggesting that some medical conditions frequently associated with COPD (osteoporosis, metabolic syndrome and heart diseases) may negatively influence rehabilitation outcomes, at present the evidence is contradictory. Copyright©ERS 2011.

PMID

22045787 [<https://www.ncbi.nlm.nih.gov/pubmed/?term=22045787>]

Status

Embase

Institution

(Garrod) Dept. of Pulmonary Rehabilitation, King's College Hospital NHS Foundation Trust, London, United Kingdom (Malerba) Dept. of Internal Medicine, University of Brescia, Brescia, Italy

(Crisafulli) Dept. of Pulmonary Rehabilitation, Villa Pineta Hospital, Via Gaiato 127, 41026 Pavullo Modena, Italy

Publisher

European Respiratory Society (4 Ave Sainte-Luce, Lausanne CH-1003, Switzerland)

Year of Publication

2011

Link to the Ovid Full Text or citation:

[Click here for full text options](https://ovidsp.ovid.com/ovidweb.cgi?T=JS&CSC=Y&NEWS=N&PAGE=fulltext&D=emed12&AN=362860682)

Link to the External Link Resolver:

[LibKey NHS](https://libkey.io/libraries/2789/openurl?genre=article&sid=OVID:emed12&genre=article&id=pmid:22045787&id=doi:10.1183%2F09031936.00088611&issn=0903-1936&volume=38&issue=5&spage=1215&pages=1215-1218&date=2011&title=European+Respiratory+Journal&atitle=Series+"novelties+in+pulmonary+rehabilitation"%3A+Determinants+of+success&aulast=Garrod)

197.

The impact of pulmonary rehabilitation on sleep quality in chronic lung disease.

Soler X., Lui A., Larsen C., Diaz-Piedra C., Limberg T., Nunn A., Correa M., Ries A.

Embase

American Journal of Respiratory and Critical Care Medicine. Conference: American Thoracic Society International Conference, ATS 2011. Denver, CO United States. Conference Publication: (var.pagings). 183(1 MeetingAbstracts) (no pagination), 2011. Date of Publication: 01 May 2011.

[Conference Abstract]

AN: 70848289

RATIONALE: Sleep-related disorders are common in patients with chronic lung disease. In other populations, exercise has been shown to improve sleep health. In patients with chronic lung disease, pulmonary rehabilitation (PR) produces important health benefits in symptoms, exercise tolerance, and quality of life. However, the effect of PR on sleep quality has not been studied previously. The aims of this observational study were to characterize sleep quality in patients with chronic lung disease and evaluate the effect of PR on sleep quality.

METHOD(S): 86 patients with moderate to severe chronic lung disease enrolled in our PR program were asked to complete a 6-minute walk test (6MW) and questionnaires related to symptoms, quality of life, and sleep quality before and after the 8-week comprehensive PR program. Outcome measures included: UCSD Shortness of Breath Questionnaire (SOBQ); SF-36 Physical (PCS) and Mental (MCS) Component Scores; Self-efficacy for Walking (SEW); and Pittsburgh Sleep Quality Index (PSQI).

RESULT(S): There were 42 (49%) males and 44 (51%) females. Diagnoses included 63 (73%) chronic obstructive pulmonary disease (COPD), 17 (20%) restrictive lung disease, and 6 (7%) mixed obstructive/restrictive lung disease. 37 (43%) used long-term oxygen. Baseline spirometry [mean(SD)] demonstrated an FEV1 % pred = 50 (18), FVC % pred = 72 (19), and FEV1/FVC % = 53 (19). Results pre/post-PR in the 64 patients (74%) who completed the PR program and were re-evaluated were: Results (Table Presented)

CONCLUSION(S): Sleep quality in patients with chronic lung disease enrolled in our PR program was poor. As expected, symptoms, exercise tolerance, and quality of life improved after PR. Additionally, PR had a significant positive impact on overall sleep quality, suggesting that PR maybe an effective non-pharmacologic treatment that improves sleep quality in these patients.

Status

CONFERENCE ABSTRACT

Institution

(Soler, Lui, Larsen, Limberg, Nunn, Correa, Ries) University of California San Diego, San Diego, CA, United States (Diaz-Piedra) Universidad de Granada, Granada, Spain

Publisher

American Thoracic Society

Year of Publication

2011

Link to the Ovid Full Text or citation:

[Click here for full text options](https://ovidsp.ovid.com/ovidweb.cgi?T=JS&CSC=Y&NEWS=N&PAGE=fulltext&D=emed12&AN=70848289)

Link to the External Link Resolver:

[LibKey NHS](https://libkey.io/libraries/2789/openurl?genre=article&sid=OVID:emed12&genre=article&id=pmid:&id=doi:&issn=1073-449X&volume=183&issue=1+MeetingAbstracts&spage=&pages=&date=2011&title=American+Journal+of+Respiratory+and+Critical+Care+Medicine&atitle=The+impact+of+pulmonary+rehabilitation+on+sleep+quality+in+chronic+lung+disease&aulast=Soler)

198.

The relationship of sleep quality and quality of life in chronic lung disease.

Diaz-Piedra C., Soler X., Lui A., Larsen C., Limberg T., Nunn A., Correa M., Ries A.

Embase

American Journal of Respiratory and Critical Care Medicine. Conference: American Thoracic Society International Conference, ATS 2011. Denver, CO United States. Conference Publication: (var.pagings). 183(1 MeetingAbstracts) (no pagination), 2011. Date of Publication: 01 May 2011.

[Conference Abstract]

AN: 70848288

Rationale: Patients with chronic lung diseases have low health-related quality of life (HRQOL) and commonly complain about sleep disturbances including poor sleep quality, sleepiness and other sleep-related disorders. It has been demonstrated that sleep disturbances are associated with reduced HRQOL in many chronic diseases, but this has not been well characterized in this population. The aim of this study was to evaluate the relationship of sleep quality with quality of life in patients enrolled in the UCSD Pulmonary Rehabilitation (PR) program.

Method(s): As part of their initial evaluation for the PR program, patients with chronic lung disease completed the Pittsburgh Sleep Quality Index (PSQI) along with other measures including spirometry, exercise performance [six-minute walk (6MW)], HRQOL [SF-36 Mental (MCS) and Physical (PCS) component scores], dyspnea [UCSD-Shortness of Breath Questionnaire (SOBQ)], and self-efficacy for walking (SEW).

Result(s): Data were collected on 86 patients including 42 (49%) males and 44 (51%) females, age [mean (SD), range = 67.8 (10.2), 36-85]. Diagnoses included 63 (73%) chronic obstructive pulmonary disease (COPD), 17 (20%) restrictive lung disease, and 6 (7%) mixed obstructive/restrictive disease. 37 (43%) used long-term oxygen. Baseline spirometry demonstrated an FEV1 % pred = 50 (18), FVC % pred = 72 (19), and FEV1/FVC % = 53 (19). The patients reported poor sleep quality [PSQI = 7.1 (4.1)] along with moderate dyspnea, low exercise tolerance, low HRQOL, and low self-efficacy [SOBQ = 55 (21), 6MW = 345m (115), MCS = 53 (9), PCS = 31 (8), SEW = 3.6 (2.8)]. PSQI was significantly correlated with both MCS (R= -0.47) and PCS (R= -0.34). Multiple stepwise regression analyses sowed that significant, independent predictors 1) for MCS were PSQI, SOBQ, PCS, and 6MW (multiple R=0.66, adjusted R2=0.39) and 2) for PCS were SOBQ, MCS, and PSQI (multiple R=0.65, adjusted R2 = 0.40).

Conclusion(s): In patients with moderate to severe chronic lung disease, sleep quality is poor and significantly related to both mental and physical aspects of HRQOL. Adjusted for the other measured variables, sleep quality was more strongly related to the mental component of HRQOL than the physical component. Poor sleep quality may be an important contributor to low HRQOL and should be considered in appropriate treatment programs for these patients.

Status

CONFERENCE ABSTRACT

Institution

(Diaz-Piedra) Universidad de Granada, Granada, Spain (Soler, Lui, Larsen, Limberg, Nunn, Correa, Ries) University of California San Diego, San Diego, CA, United States

Publisher

American Thoracic Society

Year of Publication

2011

Link to the Ovid Full Text or citation:

[Click here for full text options](https://ovidsp.ovid.com/ovidweb.cgi?T=JS&CSC=Y&NEWS=N&PAGE=fulltext&D=emed12&AN=70848288)

Link to the External Link Resolver:

[LibKey NHS](https://libkey.io/libraries/2789/openurl?genre=article&sid=OVID:emed12&genre=article&id=pmid:&id=doi:&issn=1073-449X&volume=183&issue=1+MeetingAbstracts&spage=&pages=&date=2011&title=American+Journal+of+Respiratory+and+Critical+Care+Medicine&atitle=The+relationship+of+sleep+quality+and+quality+of+life+in+chronic+lung+disease&aulast=Diaz-Piedra)

199.

Multidimensional assessment and individualized management (MDAIM) of obstructive airway diseases (OAD) in older adults- A pilot clinical trial.

McDonald V.M., Higgins I., Wood L.G., Gibson P.G.

Embase

Respirology. Conference: TSANZ and ANZSRS Annual Scientific Meetings. Perth, WA Australia. Conference Publication: (var.pagings). 16(SUPPL. 1) (pp 13), 2011. Date of Publication: April 2011.

[Conference Abstract]

AN: 70382950

Aim To test the feasibility and effect of multidimensional assessment (MDA) and individualized management (IM) in older people with OAD. We hypothesized that this approach would lead to a clinically signifi cant improvement in quality of life (QOL) using the St George Respiratory Questionnaire (SGRQ). Methods A controlled clinical trial of MDAIM versus usual care (UC) was conducted in people (>55 years) with asthma and/or COPD. All underwent a baseline MDA of biological, functional, clinical and behavioural measures. The UC group received physician management and pulmonary rehabilitation (PR). The MDAIM group received PR plus case management and infl ammometrybased management tailored according to the outcomes of the MDA. Results We recruited 29 participants, 14 cases and 15 controls; 52% were female. The mean (SD) age was 71.1 (8) years and post b-d FEV1 was 53.6% (20). With MDAIM, 57% received tailored therapy (steroids and/or macrolides) to normalize sputum cell counts, 57% received statins for systemic infl ammation, 64% were treated for mucus hypersecretion and 36% for sleep disorders. MDAIM resulted in signifi cant improvements in QOL: the mean (SD) pre- and post-intervention SGRQ scores in the MDAIM group were 55.3 (11) versus 41.1 (15), p = 0.009, compared to 50.2 (12) versus 49.3 (11), p = 0.8 for UC. The mean (SD) difference in SGRQ following MDAIM was -14.3 (12) versus -0.5 (8), p = 0.003 for UC. Conclusions MDAIM is feasible and results in clinically and statistically signifi cant improvements in QOL in older adults with OAD.

Status

CONFERENCE ABSTRACT

Institution

(McDonald, Wood, Gibson) Centre for Asthma and Respiratory Disease, John Hunter Hospital, HMRI, NSW, Australia (McDonald, Higgins) School of Nursing and Midwifery, John Hunter Hospital, HMRI, NSW, Australia

(McDonald, Wood, Gibson) Department of Respiratory and Sleep Medicine, John Hunter Hospital, HMRI, NSW, Australia

(Wood) School of Biomedical Science, University of Newcastle, Australia

Publisher

Blackwell Publishing

Year of Publication

2011

Link to the Ovid Full Text or citation:

[Click here for full text options](https://ovidsp.ovid.com/ovidweb.cgi?T=JS&CSC=Y&NEWS=N&PAGE=fulltext&D=emed12&AN=70382950)

Link to the External Link Resolver:

[LibKey NHS](https://libkey.io/libraries/2789/openurl?genre=article&sid=OVID:emed12&genre=article&id=pmid:&id=doi:10.1111%2Fj.1440-1843.2011.01936.x&issn=1323-7799&volume=16&issue=SUPPL.+1&spage=13&pages=13&date=2011&title=Respirology&atitle=Multidimensional+assessment+and+individualized+management+(MDAIM)+of+obstructive+airway+diseases+(OAD)+in+older+adults-+A+pilot+clinical+trial&aulast=McDonald)

200.

Year in review 2010: Interstitial lung diseases, acute lung injury, sleep, physiology, imaging, bronchoscopic intervention and lung cancer.

Eastwood P.R., Takahashi K., Lee P., Maher T.M.

Embase

Respirology. 16(3) (pp 553-563), 2011. Date of Publication: April 2011.

[Review]

AN: 361536033

PMID

21244574 [<https://www.ncbi.nlm.nih.gov/pubmed/?term=21244574>]

Status

Embase

Institution

(Eastwood) West Australian Sleep Disorders Research Institute, Department of Pulmonary Physiology, Sir Charles Gairdner Hospital, Perth, WA, Australia (Eastwood) School of Anatomy and Human Biology, University of Western Australia, Perth, WA, Australia

(Takahashi) Department of Respiratory Medicine, Juntendo University School of Medicine, Tokyo, Japan

(Lee) Department of Medicine, National University Hospital, Singapore, Singapore

(Maher) Interstitial Lung Disease Unit, Royal Brompton Hospital, London, United Kingdom

(Maher) National Heart Lung Institute, Imperial College, London, United Kingdom

(Maher) Centre for Respiratory Research, University College London, London, United Kingdom

Publisher

Blackwell Publishing (550 Swanston Street, Carlton South VIC 3053, Australia)

Year of Publication

2011

Link to the Ovid Full Text or citation:

[Click here for full text options](https://ovidsp.ovid.com/ovidweb.cgi?T=JS&CSC=Y&NEWS=N&PAGE=fulltext&D=emed12&AN=361536033)

Link to the External Link Resolver:

[LibKey NHS](https://libkey.io/libraries/2789/openurl?genre=article&sid=OVID:emed12&genre=article&id=pmid:21244574&id=doi:10.1111%2Fj.1440-1843.2011.01927.x&issn=1323-7799&volume=16&issue=3&spage=553&pages=553-563&date=2011&title=Respirology&atitle=Year+in+review+2010%3A+Interstitial+lung+diseases%2C+acute+lung+injury%2C+sleep%2C+physiology%2C+imaging%2C+bronchoscopic+intervention+and+lung+cancer&aulast=Eastwood)

201.

Association of change in depression and anxiety symptoms with functional outcomes in pulmonary rehabilitation patients.

Pirraglia P.A., Casserly B., Velasco R., Borgia M.L., Nici L.

Embase

Journal of Psychosomatic Research. 71(1) (pp 45-49), 2011. Date of Publication: July 2011.

[Article]

AN: 51298652

Objective: Pulmonary rehabilitation (PR) has emerged over the last decade as an essential component of an integrated approach to managing patients with chronic respiratory diseases such as chronic obstructive pulmonary disease (COPD). We sought to examine how depression and anxiety symptom changes relate to disease-specific quality of life outcomes following PR.

Method(s): We performed a cohort study of 81 patients with COPD who completed PR at a Veterans Administration Medical Center. Pulmonary rehabilitation consisted of supervised exercise training and education twice weekly for 8 weeks. Beck Depression and Anxiety Inventories (BDI and BAI) assessed symptom burden at baseline and completion of PR. We measured change in disease-specific quality of life using the dyspnea, mastery, emotion and fatigue domains of the Chronic Respiratory Questionnaire Self-Reported (CRQ-SR) from baseline to completion of PR.

Result(s): Participants were 69.8+/-9.1 years old and all male. Forced expiratory volume in 1 s (FEV1) was 1.23+/-0.39 L. The CRQ-SR scores improved significantly: dyspnea (P<.0001), mastery (P=.015) and fatigue (P=.017). The BDI scores improved significantly (13.1+/-10.5 to 10.8+/-9.9, P=.003; BAI: 13.1+/-10.1 to 12.1+/-11.7). Multivariate regression models controlling for age, FEV1, depression treatment and anxiety treatment showed that improvement in depressive symptoms were associated with improvement in fatigue (P=.003), emotion (P=.003) and mastery (P=.01). Anxiety symptom change was not significantly associated with change in disease-specific quality of life domains.

Conclusion(s): Addressing anxiety symptoms in PR patients may be indicated because disease-specific quality of life improvement appears to be associated with mood. © 2011.

PMID

21665012 [<https://www.ncbi.nlm.nih.gov/pubmed/?term=21665012>]

Status

Embase

Institution

(Pirraglia, Borgia) Primary Care Service and the Systems Outcomes and Quality in Chronic disease, Rehabilitation (SOQCR) Research Enhancement Award Program, Providence Veterans Affairs Medical Center, Providence, RI, United States (Pirraglia, Casserly, Velasco, Nici) Alpert Medical School of Brown University, Providence, RI, United States

(Casserly) Memorial Hospital, Pawtucket, RI, United States

(Borgia) Brown University, Providence, RI, United States

(Nici) Medical Service Providence Veterans Affairs Medical Center, Providence, RI, United States

Publisher

Elsevier Inc. (360 Park Avenue South, New York NY 10010, United States)

Year of Publication

2011

Link to the Ovid Full Text or citation:

[Click here for full text options](https://ovidsp.ovid.com/ovidweb.cgi?T=JS&CSC=Y&NEWS=N&PAGE=fulltext&D=emed12&AN=51298652)

Link to the External Link Resolver:

[LibKey NHS](https://libkey.io/libraries/2789/openurl?genre=article&sid=OVID:emed12&genre=article&id=pmid:21665012&id=doi:10.1016%2Fj.jpsychores.2011.01.002&issn=0022-3999&volume=71&issue=1&spage=45&pages=45-49&date=2011&title=Journal+of+Psychosomatic+Research&atitle=Association+of+change+in+depression+and+anxiety+symptoms+with+functional+outcomes+in+pulmonary+rehabilitation+patients&aulast=Pirraglia)

202.

Fatigue in patients with COPD participating in a pulmonary rehabilitation program.

Wong C.J., Goodridge D., Marciniuk D.D., Rennie D.

Embase

International journal of chronic obstructive pulmonary disease. 5 (pp 319-326), 2010. Date of Publication: 2010.

[Article]

AN: 360266049

Fatigue is a distressing, complex, multidimensional sensation common in individuals with chronic obstructive pulmonary disease (COPD). While fatigue negatively impacts functional performance and quality of life, there has been little study of the fatigue that affects participants in pulmonary rehabilitation programs. The purpose of this study was to examine the emotional, behavioral, cognitive, and physical dimensions of fatigue and their relationships to dyspnea, mental health, sleep, and physiologic factors. A convenience sample of 42 pulmonary rehabilitation participants with COPD completed self-report questionnaires which measured dimensions of fatigue using the Multidimensional Fatigue Inventory, anxiety and depression using the Hospital Anxiety and Depression Scale, and sleep quality using the Pittsburgh Sleep Quality Index. Data on other clinical variables were abstracted from pulmonary rehabilitation program health records. Almost all (95.3%) participants experienced high levels of physical fatigue. High levels of fatigue were also reported for the dimensions of reduced activity (88.1%), reduced motivation (83.3%), mental fatigue (69.9%), and general fatigue (54.5%). Close to half (42.9%) of participants reported symptoms of anxiety, while almost one quarter (21.4%) reported depressive symptoms. Age was related to the fatigue dimensions of reduced activity (rho = 0.43, P < 0.01) and reduced motivation (rho = 0.31, P < 0.05). Anxiety was related to reduced motivation (rho = -0.47, P < 0.01). Fatigue was not associated with symptoms of depression, sleep quality, gender, supplemental oxygen use, smoking status, or Medical Research Council dyspnea scores. Fatigue (particularly the physical and reduced motivation dimensions of fatigue) was experienced by almost all participants with COPD attending this pulmonary rehabilitation program. Fatigue affected greater proportions of participants than either anxiety or depression. The high prevalence of fatigue may impact on enrolment, participation, and attrition in pulmonary rehabilitation programs. Further investigation of the nature, correlates, and impact of fatigue in this population is required.

PMID

21037955 [<https://www.ncbi.nlm.nih.gov/pubmed/?term=21037955>]

Institution

(Wong) College of Nursing, University of Saskatchewan, Saskatoon, Saskatchewan, Canada

Year of Publication

2010

Link to the Ovid Full Text or citation:

[Click here for full text options](https://ovidsp.ovid.com/ovidweb.cgi?T=JS&CSC=Y&NEWS=N&PAGE=fulltext&D=emed11&AN=360266049)

Link to the External Link Resolver:

[LibKey NHS](https://libkey.io/libraries/2789/openurl?genre=article&sid=OVID:emed11&genre=article&id=pmid:21037955&id=doi:10.2147%2Fcopd.s12321&issn=1178-2005&volume=5&issue=&spage=319&pages=319-326&date=2010&title=International+journal+of+chronic+obstructive+pulmonary+disease&atitle=Fatigue+in+patients+with+COPD+participating+in+a+pulmonary+rehabilitation+program&aulast=Wong)

203.

Comorbidities of advanced lung disease.

Nations J.A., Nathan S.D.

Embase

Mount Sinai Journal of Medicine. 76(1) (pp 53-62), 2009. Date of Publication: 2009.

[Article]

AN: 354642863

Lung disease in advanced stages is often accompanied by multiple comorbidities. Often, these processes lead to significant morbidity and occasionally mortality. Symptoms of pulmonary comorbidities, including pulmonary embolus, pulmonary hypertension, sleep-disordered breathing, and lung malignancy, may be intertwined with symptoms of advancing lung disease. However, the occurrence of extra-pulmonary processes, including depression, diabetes mellitus, osteoporosis, and gastroesophageal reflux disease, may be subtle. Clinicians caring for advanced lung disease should be aware of the many comorbid processes that may accompany advancing lung disease. © 2009 Mount Sinai School of Medicine.

PMID

19170218 [<https://www.ncbi.nlm.nih.gov/pubmed/?term=19170218>]

Status

Embase

Institution

(Nations) Department of Pulmonary and Critical Care Medicine, National Naval Medicine Center, Bethesda, MD, United States (Nathan) Inova Fairfax Hospital, Falls Church, VA, United States

Publisher

John Wiley and Sons Inc. (111 River Street, Hoboken NJ 07030-5774, United States)

Year of Publication

2009

Link to the Ovid Full Text or citation:

[Click here for full text options](https://ovidsp.ovid.com/ovidweb.cgi?T=JS&CSC=Y&NEWS=N&PAGE=fulltext&D=emed11&AN=354642863)

Link to the External Link Resolver:

[LibKey NHS](https://libkey.io/libraries/2789/openurl?genre=article&sid=OVID:emed11&genre=article&id=pmid:19170218&id=doi:10.1002%2Fmsj.20086&issn=0027-2507&volume=76&issue=1&spage=53&pages=53-62&date=2009&title=Mount+Sinai+Journal+of+Medicine&atitle=Comorbidities+of+advanced+lung+disease&aulast=Nations)

204.

A Breath of Fresh Air: Abstracts from the 2010 Canadian Respiratory Conference.

Anonymous

Embase

Canadian Respiratory Journal. Conference: 2010 Canadian Respiratory Conference: A Breath of Fresh Air. Halifax, NS Canada. Conference Publication: (var.pagings). 17(SUPPL. SB) (no pagination), 2010. Date of Publication: September-October 2010.

[Conference Review]

AN: 71040631

The proceedings contain 50 papers. The topics discussed include: muscle weakness and respiratory failure from an orphan disease: adult onset Pompe disease (acid alpha-glucosidase deficiency); analysis of hospital discharge data to characterize obstructive sleep apnea and its management in pediatric and adolescent patients hospitalized in Canada: 2006 to 2007; risk of malignancy of pulmonary nodules with low 18F-FDG uptake on integrated PET/CT images; carrying on with living: the impact of pulmonary rehabilitation on the health behaviour of older adults with chronic obstructive pulmonary disease; behaviour of older adults with chronic obstructive pulmonary disease growing old with chronic obstructive pulmonary disease; pediatric pulmonary rehabilitation: using a multi-media approach to improve fitness in children with chronic lung disease; and new antistatic youth valved holding chamber (VHC) can be used out-of-package and deliver beta2-agonist medication effectively.

Status

CONFERENCE ABSTRACT

Publisher

Pulsus Group Inc.

Year of Publication

2010

Link to the Ovid Full Text or citation:

[Click here for full text options](https://ovidsp.ovid.com/ovidweb.cgi?T=JS&CSC=Y&NEWS=N&PAGE=fulltext&D=emed11&AN=71040631)

Link to the External Link Resolver:

[LibKey NHS](https://libkey.io/libraries/2789/openurl?genre=article&sid=OVID:emed11&genre=article&id=pmid:&id=doi:&issn=1198-2241&volume=17&issue=SUPPL.+SB&spage=&pages=&date=2010&title=Canadian+Respiratory+Journal&atitle=A+Breath+of+Fresh+Air%3A+Abstracts+from+the+2010+Canadian+Respiratory+Conference&aulast=)

205.

Desaturation on a 6-minute walk test may predict nocturnal hypoxemia in COPD: A validation study.

Scott A.S., Chan R., Baltzan M.A., Wolkove N.

Embase

American Journal of Respiratory and Critical Care Medicine. Conference: American Thoracic Society International Conference, ATS 2010. New Orleans, LA United States. Conference Publication: (var.pagings). 181(1 MeetingAbstracts) (no pagination), 2010. Date of Publication: 01 May 2010.

[Conference Abstract]

AN: 70841026

Introduction Patients with chronic obstructive pulmonary disease (COPD) may have significant nocturnal desaturation (SND) in the absence of of obstructive sleep apnea (OSA). We have previously shown that desaturation following a 6-minute walk test (6MWT) is useful in predicting SND in patients with COPD.The purpose of this study was to validate the finding that oxygen saturation (SpO2) of 88% or less during or after a 6MWT is predictive of SND in this population. Methods A retrospective chart review was conducted on patients attending pulmonary rehabilitation between 2008 and 2009. Patients with OSA and those on chronic supplemental oxygen were excluded. Demographic data was tabulated and the following pre-rehabilitation test results collected: spirometry, arterial blood gas, 6MWT, and overnight oximetry. Results One hundred and ninety-four patients (47% men) with COPD, mean age 70 (9.9) yrs were studied. Seventy subjects (36%) desaturated (<= 88%) with the 6MWT. The desaturator group had a lower mean FEV1 [0.73(0.33) vs 0.96(0.42) L, p < 0.01], a lower post exercise saturation [85.5(2.6) vs 93.1(2.1)%, p < 0.01], a greater change in SpO2 with exercise [7.1(2.4) vs 1.9(1.7)%, p < 0.01)], a lower mean nocturnal saturation [89.6(3.6) vs 92.0(3.2)%, p < 0.01] and more sleep time below SpO2 of 90% [43.5(35.2) vs 17.5(24.5)%, p < 0.01]. In total 56% (39/70) of the exercise desaturators and 21% (26/124) of the non-desaturators had SND. A threshold of 88% resulted in a sensitivity and specificity of 54% and 79% correspondingly, and a positive and negative likelihood ratio of 2.57 and 0.58 respectively. Conclusion This study provides more evidence that monitoring SpO2 changes during a 6MWT to saturation levels of 88% or less is useful in helping to identify patients with COPD who may have nocturnal desaturation.

Status

CONFERENCE ABSTRACT

Institution

(Scott, Baltzan, Wolkove) Mount Sinai Hospital, Montreal, Canada (Chan) McGill University, Montreal, Canada

Publisher

American Thoracic Society

Year of Publication

2010

Link to the Ovid Full Text or citation:

[Click here for full text options](https://ovidsp.ovid.com/ovidweb.cgi?T=JS&CSC=Y&NEWS=N&PAGE=fulltext&D=emed11&AN=70841026)

Link to the External Link Resolver:

[LibKey NHS](https://libkey.io/libraries/2789/openurl?genre=article&sid=OVID:emed11&genre=article&id=pmid:&id=doi:&issn=1073-449X&volume=181&issue=1+MeetingAbstracts&spage=&pages=&date=2010&title=American+Journal+of+Respiratory+and+Critical+Care+Medicine&atitle=Desaturation+on+a+6-minute+walk+test+may+predict+nocturnal+hypoxemia+in+COPD%3A+A+validation+study&aulast=Scott)

206.

Pulmonary rehabilitation fails to increase the level of physical activity in COPD.

Morris N., Seale H., Stroud N., Walsh J.

Embase

Respirology. Conference: 2010 Annual Scientific Meetings of the Thoracic Society of Australia and New Zealand (TSANZ) and the Australian and New Zealand Society of Respiratory Science (ANZSRS), Brisbane 2010. Brisbane, QLD Australia. Conference Publication: (var.pagings). 15(SUPPL. 1) (pp A40), 2010. Date of Publication: March 2010.

[Conference Abstract]

AN: 70115545

Introduction: For Chronic Obstructive Pulmonary Disease (COPD) patients, physical inactivity is a major health management concern. Recent studies have shown that individuals with higher levels of physical activity (PAL) had decreased hospital admissions and prolonged survival. In Australia, the implementation of effective exercise-based pulmonary rehabilitation (PR) has translated into improved quality of life and decreased direct and indirect costs for health care; however the impact of PR in Australia on PAL remains unknown. Therefore the aim, of this study was to examine the impact of a standard, 8-week exercise-based PR program on PAL.

Method(s): About 19 subjects (65 (9) years) with COPD (FEV1% predicted = 58 (22)) completed PR where they undertook twice weekly exercise classes consisting of one hour of upper and lower limb strengthening exercise and aerobic exercise. PAL was estimated using a multi-sensor device (SenseWear, Healthware Bodymedia) worn for a 7 day period. An index of PAL was derived by dividing total daily energy expenditure in metabolic equivalents (METS) by whole night sleeping energy expenditure (average of 3 nights sleeping). PAL was measured in the week immediately prior and in the immediately following PR.

Result(s): Despite a significant increase in six minute walk distance (6MWD), PR resulted in no change in PAL (PAL pre: 1.50 (0.22), post 1.51 (0.18), p = 0.79). Moreover there was no significant change in the daily number of steps (Steps pre: 6461 (3521), post: 6332 (3152), p = 0.74). All results are presented as mean, (SD).

Conclusion(s): While the current study demonstrated improvements in standard outcome measures, COPD patients failed to increase their PAL. While changes in PAL may take longer to elicit i.e. the change in PAL may be delayed following PR, it is possible that the current focus of PR on increasing outcomes such as 6MWD may be too narrow to elicit changes in PAL.

Status

CONFERENCE ABSTRACT

Institution

(Morris, Stroud) School of Physiotherapy and Exercise Science, Griffith University, Gold Coast, Australia (Seale, Walsh) Physiotherapy Department, Prince Charles Hospital, Chermside, QLD, Australia

Publisher

Blackwell Publishing

Year of Publication

2010

Link to the Ovid Full Text or citation:

[Click here for full text options](https://ovidsp.ovid.com/ovidweb.cgi?T=JS&CSC=Y&NEWS=N&PAGE=fulltext&D=emed11&AN=70115545)

Link to the External Link Resolver:

[LibKey NHS](https://libkey.io/libraries/2789/openurl?genre=article&sid=OVID:emed11&genre=article&id=pmid:&id=doi:10.1111%2Fj.1400-1843.2010.01735.x&issn=1323-7799&volume=15&issue=SUPPL.+1&spage=A40&pages=A40&date=2010&title=Respirology&atitle=Pulmonary+rehabilitation+fails+to+increase+the+level+of+physical+activity+in+COPD&aulast=Morris)

207.

American Association of Cardiovascular and Pulmonary Rehabilitation 25th Annual Meeting.

Anonymous

Embase

Journal of Cardiopulmonary Rehabilitation and Prevention. Conference: 25th Annual Meeting of the American Association of Cardiovascular and Pulmonary Rehabilitation. Milwaukee, WI United States. Conference Publication: (var.pagings). 30(4) (no pagination), 2010. Date of Publication: July-August 2010.

[Conference Review]

AN: 70312926

The proceedings contain 51 papers. The topics discussed include: efficacy of using telehealth technology to provide education for patients participating in a phase ii cardiac rehabilitation program; exercise performance in peripheral arterial disease patients with and without chronic kidney disease; coping style and illness knowledge as predictors of change in emotional quality of life among cardiac rehabilitation patients; associations of sleep disorders and fitness and fatness in overweight and obese women with pre-diabetes or diabetes; once weekly combined resistance and endurance training in lung function and bode index in COPD patients; the impact of early cardiac rehabilitation on myocardial function in acute myocardial infarction; incorporation of core component guidelines into cardiac rehabilitation practice; and directly measured physical function in chronic heart failure: favorable effects of high intensity resistance training.

Status

CONFERENCE ABSTRACT

Publisher

Lippincott Williams and Wilkins

Year of Publication

2010

Link to the Ovid Full Text or citation:

[Click here for full text options](https://ovidsp.ovid.com/ovidweb.cgi?T=JS&CSC=Y&NEWS=N&PAGE=fulltext&D=emed11&AN=70312926)

Link to the External Link Resolver:

[LibKey NHS](https://libkey.io/libraries/2789/openurl?genre=article&sid=OVID:emed11&genre=article&id=pmid:&id=doi:&issn=1932-7501&volume=30&issue=4&spage=&pages=&date=2010&title=Journal+of+Cardiopulmonary+Rehabilitation+and+Prevention&atitle=American+Association+of+Cardiovascular+and+Pulmonary+Rehabilitation+25th+Annual+Meeting&aulast=)

208.

Pulmonary hypertension in COPD: Epidemiology, significance, and management: Pulmonary vascular disease: The global perspective.

Minai O.A., Chaouat A., Adnot S.

Embase

Chest. 137(6 SUPPL.) (pp 39S-51S), 2010. Date of Publication: 01 Jun 2010.

[Review]

AN: 359053862

Pulmonary hypertension (PH) associated with parenchymal lung diseases is one of the most common forms of PH. Studies in patients with advanced COPD and hypoxemia have shown a very high prevalence of PH; however, prevalence in mild and moderate COPD is not known. Typical hemodynamic abnormalities include mild-to-moderate elevations in pulmonary artery pressure (PAP) and pulmonary vascular resistance with a preserved cardiac output. A small proportion (, 5%) of patients may have significant elevations in PAP (mean PAP. 35-40 mm Hg) in the presence of mild airflow limitation and are believed to have disproportionate PH. COPD-associated PH has significant clinical implications because it can produce functional limitation and has a negative impact on prognosis. Doppler echocardiography is the best noninvasive test, but noninvasive methods used for diagnosis are prone to error and cannot be relied on when making or refuting the diagnosis of PH. All patients require right-sided heart catheterization if treatment with PH-specific medications is contemplated. The most important steps in managing these patients are: (1) confirm the diagnosis; (2) optimize COPD management; (3) rule out comorbidities; (4) assess and treat hypoxemia; and (5) enroll the patient in pulmonary rehabilitation, if indicated. In patients with PH and advanced airflow limitation, lung transplantation offers the best opportunity for long-term benefit. The role of PH-specific medications remains poorly defined and requires further study but may be considered in patients with disproportionate PH. © 2010 American College of Chest Physicians.

PMID

20522579 [<https://www.ncbi.nlm.nih.gov/pubmed/?term=20522579>]

Status

Embase

Institution

(Minai) Department of Pulmonary and Critical Care Medicine, Cleveland Clinic, Cleveland, OH, United States (Chaouat) Universitaire de Nancy, Service des Maladies Respiratoires et Reanimation Respiratoire, Hopital d'Adultes de Brabois, Vandoeuvre-les-Nancy, Cedex, France

(Adnot) Medical School of Creteil, Hopital Henri Mondor, Creteil, France

Publisher

American College of Chest Physicians (3300 Dundee Road, Northbrook IL 60062-2348, United States)

Year of Publication

2010

Link to the Ovid Full Text or citation:

[Click here for full text options](https://ovidsp.ovid.com/ovidweb.cgi?T=JS&CSC=Y&NEWS=N&PAGE=fulltext&D=emed11&AN=359053862)

Link to the External Link Resolver:

[LibKey NHS](https://libkey.io/libraries/2789/openurl?genre=article&sid=OVID:emed11&genre=article&id=pmid:20522579&id=doi:10.1378%2Fchest.10-0087&issn=0012-3692&volume=137&issue=6+SUPPL.&spage=39S&pages=39S-51S&date=2010&title=Chest&atitle=Pulmonary+hypertension+in+COPD%3A+Epidemiology%2C+significance%2C+and+management%3A+Pulmonary+vascular+disease%3A+The+global+perspective&aulast=Minai)

209.

Development of electronic medical record (EMR) templates to optimize evidence-based management of COPD outpatients.

Gronkiewicz C., Borkgren-Okonek M., Diamond E., Hickam P.L.

Embase

Chest. Conference: American College of Chest Physicians Annual Meeting, CHEST 2009. San Diego, CA United States. Conference Publication: (var.pagings). 136(4) (no pagination), 2009. Date of Publication: 01 Oct 2009.

[Conference Abstract]

AN: 70203983

PURPOSE: To develop disease-specific EMR templates for outpatient COPD management to promote compliance with evidence-based guidelines and to facilitate organized performance measurements.

METHOD(S): This abstract describes the initial phase of a multiphase EMR implementation project for COPD at a large suburban pulmonary/allergy/sleep/critical care medicine practice. Fifteen providers conducted 28,484 outpatient COPD visits at three sites during the 14-month pre-implementation period. Four templates were developed by APNs and MDs over 6 months. Technology consultants facilitated integration of developed content into the existing EMR framework. Two COPD templates were created for consult/initial visit and follow-up/exacerbation visit. Key elements of the GOLD recommendations for patient evaluation utilizing narrative text and validated tools (MMRC, CCQ) were grouped into 8 domains: symptoms, cough, dyspnea, exposure/acuity, oxygen usage, activity level, quality of life, and COPD severity level based on spirometry. Two APN patient education and exercise templates were created to address GOLD recommendations for improving health education and functional status. The education templates and corresponding patient handouts address thirteen topic areas: correct medication and oxygen use, quality of life (sleep, nutrition, mood), activity level (exercise plan), and selfmanagement (recognition of exacerbation, energy conservation). An exercise template was designed for patients not enrolled in formal pulmonary rehabilitation. This individualized plan of strengthening and aerobic exercises is based on each patient's dynamic health status and revised on an ongoing basis. The four templates were placed in a test mode for one month for provider feedback prior to clinical implementation.

RESULT(S): 1. Four COPD templates provide a standardized framework for practice-wide implementation of GOLD guidelines in evaluation and management of COPD outpatients. 2. EMR documentation yields an ongoing and organized record of clinical assessment, COPD severity level, health education, and functional status.

CONCLUSION(S): COPD templates provide a comprehensive, individualized, and organized assessment tool based on GOLD guidelines. CLINICAL IMPLICATIONS: 1. Electronic data search and analysis will facilitate performance measurement to improve clinical management and patient outcomes. 2. Data captured support future focus on automated processes to enhance clinical decision making.

Status

CONFERENCE ABSTRACT

Institution

(Gronkiewicz, Borkgren-Okonek, Diamond, Hickam) Suburban Lung Associates, Elk Grove Village, IL, United States

Publisher

American College of Chest Physicians

Year of Publication

2009

Link to the Ovid Full Text or citation:

[Click here for full text options](https://ovidsp.ovid.com/ovidweb.cgi?T=JS&CSC=Y&NEWS=N&PAGE=fulltext&D=emed11&AN=70203983)

Link to the External Link Resolver:

[LibKey NHS](https://libkey.io/libraries/2789/openurl?genre=article&sid=OVID:emed11&genre=article&id=pmid:&id=doi:&issn=0012-3692&volume=136&issue=4&spage=&pages=&date=2009&title=Chest&atitle=Development+of+electronic+medical+record+(EMR)+templates+to+optimize+evidence-based+management+of+COPD+outpatients&aulast=Gronkiewicz)

210.

Assessment of fatigue in patients with chronic obstructive pulmonary disease participating in a pulmonary rehabilitation program: A feasibility study.

Wong C., Goodridge D., Marciniuk D., Rennie D.

Embase

Chest. Conference: American College of Chest Physicians Annual Meeting, CHEST 2009. San Diego, CA United States. Conference Publication: (var.pagings). 136(4) (no pagination), 2009. Date of Publication: 01 Oct 2009.

[Conference Abstract]

AN: 70203542

PURPOSE: Fatigue is a prominent complaint of persons with COPD. Fatigue is a distressing, multidimensional sensation that adversely affects quality of life. Few studies have examined factors that influence various dimensions of subjective fatigue in these individuals. The purpose of this study was to examine the emotional, behavioural, cognitive, and physical dimensions of fatigue and their relationships to dyspnea, mental health, sleep, and physiological factors in individuals with COPD enrolled in a pulmonary rehabilitation program.

METHOD(S): A convenience sample of 42 pulmonary rehabilitation participants with COPD completed self-report questionnaires which measured: dimensions of fatigue using the Multidimensional Fatigue Inventory, anxiety and depression using the Hospital Anxiety and Depression Scale, and sleep quality using the Pittsburgh Sleep Quality Index. Data on other variables were abstracted from pulmonary rehabilitation program health records.

RESULT(S): The majority of the participants (61.9% - 81.0%) experienced moderate levels of subjective fatigue across emotional, behavioural, cognitive and physical dimensions. Moderate to severe levels of physical fatigue were experienced by 95.3% of participants. Age was related to behavioural fatigue (P = 0.43, p < 0.01) and emotional fatigue (P = 0.31, p < 0.05). Anxiety was related to emotional fatigue (P = -0.47, p < 0.01). Fatigue was not associated with sex, supplemental oxygen use, smoking status, or MRC dyspnea scores. Nearly half (42.9%) the participants reported symptoms of anxiety, while almost one-quarter (21.4%) reported depressive symptoms.

CONCLUSION(S): Fatigue, particularly physical fatigue, was experienced by the overwhelming majority of participants in this study. Further research is needed: to determine the prevalence of fatigue, anxiety and depression using a representative sample of pulmonary rehabilitation participants; to identify how these factors are related to participation in and outcomes of pulmonary rehabilitation programs; and to compare fatigue, anxiety and depression between pulmonary rehabilitation participants and non-participants. CLINICAL IMPLICATIONS: While dyspnea is often assessed in individuals with COPD, fatigue is often inadequately assessed and monitored. Pulmonary rehabilitation programs may incorporate measures of fatigue and interventions intended to alleviate fatigue into their curricula.

Status

CONFERENCE ABSTRACT

Institution

(Wong, Goodridge, Marciniuk, Rennie) University of Saskatchewan, Saskatoon, SK, Canada

Publisher

American College of Chest Physicians

Year of Publication

2009

Link to the Ovid Full Text or citation:

[Click here for full text options](https://ovidsp.ovid.com/ovidweb.cgi?T=JS&CSC=Y&NEWS=N&PAGE=fulltext&D=emed11&AN=70203542)

Link to the External Link Resolver:

[LibKey NHS](https://libkey.io/libraries/2789/openurl?genre=article&sid=OVID:emed11&genre=article&id=pmid:&id=doi:&issn=0012-3692&volume=136&issue=4&spage=&pages=&date=2009&title=Chest&atitle=Assessment+of+fatigue+in+patients+with+chronic+obstructive+pulmonary+disease+participating+in+a+pulmonary+rehabilitation+program%3A+A+feasibility+study&aulast=Wong)

211.

COPD: Optimal current treatment strategies and promising new therapeutic modalities.

Starakis I.

Embase

Current Respiratory Medicine Reviews. 5(2) (pp 73-80), 2009. Date of Publication: 2009.

[Review]

AN: 355426394

COPD is a condition characterized by progressive airflow obstruction which is rather insensible to bronchodilators. The pathogenetic mechanisms of this disorder are multifaceted, encompassing pulmonary inflammation, small airway remodelling, emphysema and mucous hypersecretion. As our comprehension of the heterogeneous pathology of the disease has improved it has been recognized that the ongoing inflammatory process in COPD is correlated with the relentlessness of this condition. Therefore, the scientific interest has been directed in the development of new anti-inflammatory drugs targeting pulmonary inflammation. In this survey, we will try to evaluate current treatment options and the potential and efficacy of new treatment modalities. © 2009 Bentham Science Publishers Ltd.

Status

Embase

Institution

(Starakis) Patras University Hospital, 26500 Rion-Patras, Greece

Publisher

Bentham Science Publishers B.V. (P.O. Box 294, Bussum 1400 AG, Netherlands)

Year of Publication

2009

Link to the Ovid Full Text or citation:

[Click here for full text options](https://ovidsp.ovid.com/ovidweb.cgi?T=JS&CSC=Y&NEWS=N&PAGE=fulltext&D=emed11&AN=355426394)

Link to the External Link Resolver:

[LibKey NHS](https://libkey.io/libraries/2789/openurl?genre=article&sid=OVID:emed11&genre=article&id=pmid:&id=doi:10.2174%2F157339809788189923&issn=1573-398X&volume=5&issue=2&spage=73&pages=73-80&date=2009&title=Current+Respiratory+Medicine+Reviews&atitle=COPD%3A+Optimal+current+treatment+strategies+and+promising+new+therapeutic+modalities&aulast=Starakis)

212.

Identification and Evaluation of the Patient with Lung Disease.

Sweitzer B.J., Smetana G.W.

Embase

Anesthesiology Clinics. 27(4) (pp 673-686), 2009. Date of Publication: December 2009.

[Review]

AN: 355677399

Preoperative pulmonary evaluation and optimization improves postoperative patient outcomes. Clinicians frequently evaluate patients with pulmonary disease before surgery who are at increased risk for pulmonary and nonpulmonary perioperative complications. Postoperative pulmonary complications are as common and costly as cardiac complications. In this article, the evaluation of patients with the most common conditions encountered in the preoperative setting, including unexplained dyspnea, asthma, chronic obstructive pulmonary disease, obstructive sleep apnea, and cigarette use, are discussed. Risk stratification for postoperative pulmonary complications and strategies to reduce them for high-risk patients are also discussed. From the available literature, high-risk patients and those patients for whom a multidisciplinary collaboration will be most helpful can be accurately identified. © 2009 Elsevier Inc. All rights reserved.

Status

Embase

Institution

(Sweitzer) Department of Anesthesia and Critical Care, University of Chicago, 5841 S, Maryland Avenue, Chicago, IL 60637, United States (Sweitzer) Department of Medicine, University of Chicago, 5841 S, Maryland Avenue, Chicago, IL 60637, United States

(Sweitzer) Anesthesia Perioperative Medicine Clinic, University of Chicago Medical Center, 5841 S, Maryland Avenue, Chicago, IL 60637, United States

(Smetana) Division of General Medicine and Primary Care, Beth Israel Deaconess Medical Center, Harvard Medical School, 330 Brookline Avenue, Boston, MA, United States

Publisher

W.B. Saunders (Independence Square West, Philadelphia PA 19106-3399, United States)

Year of Publication

2009

Link to the Ovid Full Text or citation:

[Click here for full text options](https://ovidsp.ovid.com/ovidweb.cgi?T=JS&CSC=Y&NEWS=N&PAGE=fulltext&D=emed11&AN=355677399)

Link to the External Link Resolver:

[LibKey NHS](https://libkey.io/libraries/2789/openurl?genre=article&sid=OVID:emed11&genre=article&id=pmid:&id=doi:10.1016%2Fj.anclin.2009.09.004&issn=1932-2275&volume=27&issue=4&spage=673&pages=673-686&date=2009&title=Anesthesiology+Clinics&atitle=Identification+and+Evaluation+of+the+Patient+with+Lung+Disease&aulast=Sweitzer)

213.

Noninvasive ventilation in pulmonary rehabilitation of COPD patients.

Kohnlein T., Schonheit-Kenn U., Winterkamp S., Welte T., Kenn K.

Embase

Respiratory Medicine. 103(9) (pp 1329-1336), 2009. Date of Publication: September 2009.

[Article]

AN: 50484892

Noninvasive positive pressure ventilation (NIPPV) has been shown to improve exercise tolerance and health-related quality of life in patients with advanced COPD. This study tested the feasibility of nocturnal NIPPV as an additional tool in a hospital-based pulmonary rehabilitation program. This prospective observational trial included forty COPD patients in GOLD stage IV. NIPPV was successfully introduced and accepted during sleep by all patients. All patients received pressure support ventilation for 7.9 +/- 0.5 h per day with an inspiratory support of 17.5 +/- 4.4 cmH2O, and an expiratory pressure of 4.5 +/- 0.9 cmH2O. The outcome of pulmonary rehabilitation in patients receiving nocturnal NIPPV was compared with the results of forty matched control patients who underwent the same program. Rehabilitation with nocturnal NIPPV resulted in the 6-minute walk test and in the longest non-stop walk distance in improvements of 82 and 89 m, respectively, while patients without nocturnal ventilatory support improved by 50 and 51 m (p < 0.04 and p < 0.03 between groups, respectively). Further significant improvements were found for FEV1, lung hyperinflation, and blood gases in the NIPPV treated, but not in the control subjects. Health-related quality of life, assessed by the SF-36 questionnaire, improved moderately or largely in patients receiving NIPPV in the categories role-physical, vitality, social function, and mental health. Control subjects improved moderately in vitality only. In conclusion, nocturnal NIPPV is feasible and enhances the effects of pulmonary rehabilitation in advanced stage COPD. © 2009 Elsevier Ltd. All rights reserved.

PMID

19362809 [<https://www.ncbi.nlm.nih.gov/pubmed/?term=19362809>]

Status

Embase

Institution

(Kohnlein, Welte) Hannover Medical School, Department of Respiratory Medicine, Carl-Neuberg-Strasse 1, D-30625 Hannover, Germany (Schonheit-Kenn, Winterkamp, Kenn) Klinikum Berchtesgadener Land, Department of Respiratory Medicine, Malterhoh 1, D-83471 Schoenau, Germany

Publisher

W.B. Saunders Ltd (32 Jamestown Road, London NW1 7BY, United Kingdom)

Clinical Trial Number

<https://clinicaltrials.gov/show/NCT00710463>

Year of Publication

2009

Link to the Ovid Full Text or citation:

[Click here for full text options](https://ovidsp.ovid.com/ovidweb.cgi?T=JS&CSC=Y&NEWS=N&PAGE=fulltext&D=emed11&AN=50484892)

Link to the External Link Resolver:

[LibKey NHS](https://libkey.io/libraries/2789/openurl?genre=article&sid=OVID:emed11&genre=article&id=pmid:19362809&id=doi:10.1016%2Fj.rmed.2009.03.016&issn=0954-6111&volume=103&issue=9&spage=1329&pages=1329-1336&date=2009&title=Respiratory+Medicine&atitle=Noninvasive+ventilation+in+pulmonary+rehabilitation+of+COPD+patients&aulast=Kohnlein)

214.

Chronic obstructive pulmonary disease in long-term care.

Pleasants R.A.

Embase

Annals of Long-Term Care. 17(3) (pp 24-30), 2009. Date of Publication: March 2009.

[Review]

AN: 354932753

Chronic obstructive pulmonary disease (COPD) is a disease with a progressive and complex nature, and therefore is commonly found in nursing home residents. COPD has been found to be as likely as kidney disease to lead to admission to a skilled nursing facility in elderly patients who underwent coronary artery bypass surgery. Whereas death from heart disease and stroke are declining, the death rate from COPD is on the increase. According to one study, one in every six admissions to nursing homes was for patients with a history of emphysema or COPD. Tobacco smoking is the primary cause of COPD in 80-90% of persons who have the disease. This article discusses relevant issues for persons with COPD residing in LTC facilities.

Status

Embase

Institution

(Pleasants) Campbell University, School of Pharmacy, Buies Creek, NC (Pleasants) Division of Pulmonary and Critical Care Medicine, Duke University, School of Medicine, Durham, NC

Publisher

HMP Communications LLP (4365 U.S. Highway 1 Suite 250, Princeton NJ 08540, United States)

Year of Publication

2009

Link to the Ovid Full Text or citation:

[Click here for full text options](https://ovidsp.ovid.com/ovidweb.cgi?T=JS&CSC=Y&NEWS=N&PAGE=fulltext&D=emed11&AN=354932753)

Link to the External Link Resolver:

[LibKey NHS](https://libkey.io/libraries/2789/openurl?genre=article&sid=OVID:emed11&genre=article&id=pmid:&id=doi:&issn=1524-7929&volume=17&issue=3&spage=24&pages=24-30&date=2009&title=Annals+of+Long-Term+Care&atitle=Chronic+obstructive+pulmonary+disease+in+long-term+care&aulast=Pleasants)

215.

Systemic manifestations and comorbidities of COPD.

Barnes P.J., Celli B.R.

Embase

European Respiratory Journal. 33(5) (pp 1165-1185), 2009. Date of Publication: May 2009.

[Review]

AN: 354698367

Increasing evidence indicates that chronic obstructive pulmonary disease (COPD) is a complex disease involving more than airflow obstruction. Airflow obstruction has profound effects on cardiac function and gas exchange with systemic consequences. In addition, as COPD results from inflammation and/or alterations in repair mechanisms, the "spill-over" of inflammatory mediators into the circulation may result in important systemic manifestations of the disease, such as skeletal muscle wasting and cachexia. Systemic inflammation may also initiate or worsen comorbid diseases, such as ischaemic heart disease, heart failure, osteoporosis, normocytic anaemia, lung cancer, depression and diabetes. Comorbid diseases potentiate the morbidity of COPD, leading to increased hospitalisations, mortality and healthcare costs. Comorbidities complicate the management of COPD and need to be evaluated carefully. Current therapies for comorbid diseases, such as statins and peroxisome proliferator-activated receptor-agonists, may provide unexpected benefits for COPD patients. Treatment of COPD inflammation may concomitantly treat systemic inflammation and associated comorbidities. However, new broad-spectrum anti-inflammatory treatments, such as phosphodiesterase 4 inhibitors, have significant side-effects so it may be necessary to develop inhaled drugs in the future. Another approach is the reversal of corticosteroid resistance, for example with effective antioxidants. More research is needed on COPD comorbidities and their treatment. Copyright © ERS Journals Ltd 2009.

PMID

19407051 [<https://www.ncbi.nlm.nih.gov/pubmed/?term=19407051>]

Status

Embase

Institution

(Barnes) National Heart and Lung Institute, Imperial College, London, United Kingdom (Celli) Caritas St. Elizabeth's Medical Center, Tufts University School of Medicine, Boston, MA, United Kingdom

(Barnes) Airway Disease Section, National Heart and Lung Institute, Dovehouse Street, London SW3 6LY, United Kingdom

Publisher

European Respiratory Society (4 Ave Sainte-Luce, Lausanne CH-1003, Switzerland)

Year of Publication

2009

Link to the Ovid Full Text or citation:

[Click here for full text options](https://ovidsp.ovid.com/ovidweb.cgi?T=JS&CSC=Y&NEWS=N&PAGE=fulltext&D=emed11&AN=354698367)

Link to the External Link Resolver:

[LibKey NHS](https://libkey.io/libraries/2789/openurl?genre=article&sid=OVID:emed11&genre=article&id=pmid:19407051&id=doi:10.1183%2F09031936.00128008&issn=0903-1936&volume=33&issue=5&spage=1165&pages=1165-1185&date=2009&title=European+Respiratory+Journal&atitle=Systemic+manifestations+and+comorbidities+of+COPD&aulast=Barnes)

216.

Wrist actigraphy validation of exercise movement in COPD.

Bauldoff G.S., Ryan-Wenger N.A., Diaz P.T.

Embase

Western Journal of Nursing Research. 29(7) (pp 789-802), 2007. Date of Publication: November 2007.

[Article]

AN: 47512936

A wrist actigraph is a device used in sleep research studies to measure whole body movements. The purpose of this study was to evaluate the feasibility, sensitivity, and validity of wrist actigraphy during pulmonary rehabilitation (PR) upper-extremity exercise in chronic obstructive pulmonary disease (COPD) patients. In this study, 20 patients wore Octagonal Basic Motionlogger actigraphs during two 90-minute PR sessions while the investigator recorded details of the subject's upper-extremity movements. Concurrent validity with supervised exercise records was supported for upper-extremity endurance (UEE) intensity at baseline (r =.885, p <.001) and 1 week (r =.935, p <.001). Criterion validity was supported for UEE (r =.56, p =.01) and combined lower- and upper-extremity resistance (r =.72, p <.01) compared with rank-ordered type of exercise. Wrist actigraphy is shown to be a feasible, sensitive, and valid instrument to measure upper-extremity movement during PR in COPD patients. © 2007 Sage Publications.

PMID

17636244 [<https://www.ncbi.nlm.nih.gov/pubmed/?term=17636244>]

Institution

(Bauldoff, Ryan-Wenger) Ohio State University, College of Nursing, United States (Diaz) College of Medicine and Public Health, United States

Publisher

SAGE Publications Inc. (2455 Teller Road, Thousand Oaks CA 91320, United States)

Year of Publication

2007

Link to the Ovid Full Text or citation:

[Click here for full text options](https://ovidsp.ovid.com/ovidweb.cgi?T=JS&CSC=Y&NEWS=N&PAGE=fulltext&D=emed10&AN=47512936)

Link to the External Link Resolver:

[LibKey NHS](https://libkey.io/libraries/2789/openurl?genre=article&sid=OVID:emed10&genre=article&id=pmid:17636244&id=doi:10.1177%2F0193945906297371&issn=0193-9459&volume=29&issue=7&spage=789&pages=789-802&date=2007&title=Western+Journal+of+Nursing+Research&atitle=Wrist+actigraphy+validation+of+exercise+movement+in+COPD&aulast=Bauldoff)

217.

Patients with chronic obstructive pulmonary disease: Between reality and expectations - A patient's perspective.

Rozenbaum W.V.

Embase

Disease Management and Health Outcomes. 16(5) (pp 353-358), 2008. Date of Publication: 2008.

[Note]

AN: 352832133

For many years, chronic obstructive pulmonary disease (COPD) has been under-recognized and stigmatized. Misconceptions about this disease have led to under-treatment and under-funding, resulting in an increase in the burden of COPD. In 2005, the National Emphysema/COPD Association published the results of a set of national surveys of patients, primary care physicians, and pulmonologists. The findings of these surveys indicated that activity limitation was prevalent among patients with COPD, and that, although most physicians believed that effective therapy could slow the progression of COPD, their inadequate knowledge and poor adherence to practice guidelines had a negative impact on the care of patients with COPD. Patients with COPD may not be optimally treated by physicians. Greater attention needs to be paid to effective smoking cessation programs and self-management. Many physicians under-prescribe effective therapies, with some patients experiencing the ill effects of long-term systemic corticosteroids. Ambulatory oxygen is an effective therapy for COPD. However, the use of ambulatory oxygen can make daily living and leisure activities difficult for patients, and thus physicians have difficulty convincing patients to initiate this therapy. Attention needs to be paid to finding the right oxygen delivery system for the patient, and to educating patients on the correct use of this therapy, particularly when travelling. Another important issue surrounding oxygen therapy is sleep anxiety and the fear of breathlessness or dying in one's sleep. COPD exacerbations have a major impact on quality of life; however, most therapies used to treat exacerbations have been designed for the treatment of asthma. Corticosteroids, antibacterials, and bronchodilators are routinely used for the treatment of exacerbations, but physicians often do not follow practice guidelines. Exacerbation management is too often 'too little, too late.' Another area of concern for patients is effective use of inhalers; incorrect inhaler technique is too common. It has been established that pulmonary rehabilitation programs should be an integral part of the management of COPD, particularly in patients with moderate or severe disease. Currently, the availability of pulmonary rehabilitation programs in the US is limited, as reimbursement is either inadequate or not available. In addition, many physicians do not refer patients to these programs when they are available. Medical advances notwithstanding, most patients with COPD demand therapies that are more effective, more enabling, and cause fewer adverse effects than current therapies. The challenge for medical science and the pharmaceutical industry is to bring about a qualitative change in therapy for the acute exacerbations of COPD as well as for the perpetual shortness of breath, which has such a devastating effect on quality of life. It is very encouraging that the medical community is beginning to recognize this challenge and is moving towards treating patients with COPD as 'whole people' and training them to self-manage at home. The overall success of these efforts is dependent on the recognition of COPD as a national health priority. © 2008 Adis Data Information BV. All rights reserved.

Status

Embase

Institution

(Rozenbaum) COPD-ALERT, International Online Patient Support and Advocacy Group, Silver Spring, MD, United States (Rozenbaum) International Online Patient Support and Advocacy Group, 3210 N. Leisure World Blvd., No. 614, Silver Spring, MD 20906, United States

Publisher

Adis International Ltd (41 Centorian Drive, Private Bag 65901, Mairangi Bay, Auckland 10 1311, New Zealand)

Year of Publication

2008

Link to the Ovid Full Text or citation:

[Click here for full text options](https://ovidsp.ovid.com/ovidweb.cgi?T=JS&CSC=Y&NEWS=N&PAGE=fulltext&D=emed10&AN=352832133)

Link to the External Link Resolver:

[LibKey NHS](https://libkey.io/libraries/2789/openurl?genre=article&sid=OVID:emed10&genre=article&id=pmid:&id=doi:10.2165%2F0115677-200816050-00012&issn=1173-8790&volume=16&issue=5&spage=353&pages=353-358&date=2008&title=Disease+Management+and+Health+Outcomes&atitle=Patients+with+chronic+obstructive+pulmonary+disease%3A+Between+reality+and+expectations+-+A+patient's+perspective&aulast=Rozenbaum)

218.

Pulmonary hypertension in COPD.

Chaouat A., Naeije R., Weitzenblum E.

Embase

European Respiratory Journal. 32(5) (pp 1371-1385), 2008. Date of Publication: November 2008.

[Review]

AN: 354120587

Mild-to-moderate pulmonary hypertension is a common complication of chronic obstructive pulmonary disease (COPD); such a complication is associated with increased risks of exacerbation and decreased survival. Pulmonary hypertension usually worsens during exercise, sleep and exacerbation. Pulmonary vascular remodelling in COPD is the main cause of increase in pulmonary artery pressure and is thought to result from the combined effects of hypoxia, inflammation and loss of capillaries in severe emphysema. A small proportion of COPD patients may present with "out-of-proportion" pulmonary hypertension, defined by a mean pulmonary artery pressure >35-40 mmHg (normal is no more than 20 mmHg) and a relatively preserved lung function (with low to normal arterial carbon dioxide tension) that cannot explain prominent dyspnoea and fatigue. The prevalence of out-of-proportion pulmonary hypertension in COPD is estimated to be very close to the prevalence of idiopathic pulmonary arterial hypertension. Cor pulmonale, defined as right ventricular hypertrophy and dilatation secondary to pulmonary hypertension caused by respiratory disorders, is common. More studies are needed to define the contribution of cor pulmonale to decreased exercise capacity in COPD. These studies should include improved imaging techniques and biomarkers, such as the B-type natriuretic peptide and exercise testing protocols with gas exchange measurements. The effects of drugs used in pulmonary arterial hypertension should be tested in chronic obstructive pulmonary disease patients with severe pulmonary hypertension. In the meantime, the treatment of cor pulmonale in chronic obstructive pulmonary disease continues to rest on supplemental oxygen and a variety of measures aimed at the relief of airway obstruction. Copyright©ERS Journals Ltd 2008.

PMID

18978137 [<https://www.ncbi.nlm.nih.gov/pubmed/?term=18978137>]

Status

Embase

Institution

(Chaouat) Dept. for Respiratory Diseases and Respiratory Intensive Care, Centre Hospitalier Universitaire de Nancy, Vandoeuvre-les-Nancy, France (Weitzenblum) Dept. of Pneumology, Centre Hospitalier Universitaire de Strasbourg, Strasbourg, France

(Naeije) Dept. of Pathophysiology, Erasme Campus, Brussels, Belgium

(Chaouat) Service des Maladies Respiratoires et Reanimation Respiratoire, Hopital de Brabois, Allee du Morvan, 54511 Vandoeuvre-les-Nancy Cedex, France

Publisher

European Respiratory Society (4 Ave Sainte-Luce, Lausanne CH-1003, Switzerland)

Year of Publication

2008

Link to the Ovid Full Text or citation:

[Click here for full text options](https://ovidsp.ovid.com/ovidweb.cgi?T=JS&CSC=Y&NEWS=N&PAGE=fulltext&D=emed10&AN=354120587)

Link to the External Link Resolver:

[LibKey NHS](https://libkey.io/libraries/2789/openurl?genre=article&sid=OVID:emed10&genre=article&id=pmid:18978137&id=doi:10.1183%2F09031936.00015608&issn=0903-1936&volume=32&issue=5&spage=1371&pages=1371-1385&date=2008&title=European+Respiratory+Journal&atitle=Pulmonary+hypertension+in+COPD&aulast=Chaouat)

219.

Sleep abnormalities and treatment in emphysema.

Krachman S., Minai O.A., Scharf S.M.

Embase

Proceedings of the American Thoracic Society. 5(4) (pp 536-542), 2008. Date of Publication: May 2008.

[Review]

AN: 351669183

Sleep abnormalities are common in severe emphysema, and include poor sleep quality, the development of nocturnal oxygen desaturation, and the presence of coexistent obstructive sleep apnea. With lower baseline oxygenation and abnormal respiratory mechanics in patients with severe emphysema, alterations inventilatory control and respiratorymuscle function that normally occur during sleep can have profound effects, and contribute to the development of sleep abnormalities. The impact on quality of life, cardiopulmonary hemodynamics, and overall survival remains uncertain. In addition, treatment for chronic obstructive pulmonary disease and its effect on sleep abnormalities have demonstrated conflicting results. More recently, as part of the National Emphysema Treatment Trial, lung volume reduction surgery has been shown to improve both sleep quality and nocturnal oxygenation in emphysema. Although indications for performing an overnight polysomnogram in patients with emphysema have been debated, recommendations have been presented. Future studies investigating disease mechanism and response to therapy in patients with sleep abnormalities and severe emphysema are warranted.

PMID

18453368 [<https://www.ncbi.nlm.nih.gov/pubmed/?term=18453368>]

Status

Embase

Institution

(Krachman) Sleep Disorders Center, Division of Pulmonary and Critical Care, Temple University School of Medicine, Philadelphia, PA, United States (Minai) Division of Pulmonary, Allergy, and Critical Care, Cleveland Clinic, Cleveland, OH, United States

(Scharf) Sleep Disorders Center, Division of Pulmonary and Critical Care, University of Maryland, Baltimore, MD, United States

(Krachman) Temple University School of Medicine, 3401 North Broad Street, Philadelphia, PA 19140, United States

Publisher

American Thoracic Society (61 Broadway 4th Floor, New York NY 10006 - 2755, United States)

Year of Publication

2008

Link to the Ovid Full Text or citation:

[Click here for full text options](https://ovidsp.ovid.com/ovidweb.cgi?T=JS&CSC=Y&NEWS=N&PAGE=fulltext&D=emed10&AN=351669183)

Link to the External Link Resolver:

[LibKey NHS](https://libkey.io/libraries/2789/openurl?genre=article&sid=OVID:emed10&genre=article&id=pmid:18453368&id=doi:10.1513%2Fpats.200708-134ET&issn=1546-3222&volume=5&issue=4&spage=536&pages=536-542&date=2008&title=Proceedings+of+the+American+Thoracic+Society&atitle=Sleep+abnormalities+and+treatment+in+emphysema&aulast=Krachman)

220.

The development of anticholinergics in the management of COPD.

Scullion J.E.

Embase

International Journal of COPD. 2(1) (pp 33-40), 2007. Date of Publication: 2007.

[Review]

AN: 46915826

Anticholinergics have been used to treat obstructive respiratory disease for many years from historical preparations of the deadly nightshade genus, to the more recent developments of ipratropium, oxitropium, and tiotropium. The medical treatment of airways obstruction has focused on achieving maximal airway function through bronchodilators. Of the two main bronchodilators, beta2-agonists are often the first treatment choice although there is evidence of equivalence and some suggestions of the superiority of anticholinergics in chronic obstructive pulmonary disease (COPD). The following review looks at the background of anticholinergics, their pharmacological properties, and the evidence for use with suggestions for their place in the treatment of COPD. © 2007 Dove Medical Press Limited. All rights reserved.

PMID

18044064 [<https://www.ncbi.nlm.nih.gov/pubmed/?term=18044064>]

Status

Embase

Institution

(Scullion) University Hospitals of Leicester Glenfield Site, Institute for Lung Health, Leicester, United Kingdom (Scullion) Department of General Practice and Primary Care, Aberdeen University, Aberdeen, United Kingdom

Publisher

DOVE Medical Press Ltd. (PO Box 300-008, Albany, Auckland, New Zealand)

Year of Publication

2007

Link to the Ovid Full Text or citation:

[Click here for full text options](https://ovidsp.ovid.com/ovidweb.cgi?T=JS&CSC=Y&NEWS=N&PAGE=fulltext&D=emed10&AN=46915826)

Link to the External Link Resolver:

[LibKey NHS](https://libkey.io/libraries/2789/openurl?genre=article&sid=OVID:emed10&genre=article&id=pmid:18044064&id=doi:10.2147%2Fcopd.2007.2.1.33&issn=1176-9106&volume=2&issue=1&spage=33&pages=33-40&date=2007&title=International+Journal+of+COPD&atitle=The+development+of+anticholinergics+in+the+management+of+COPD&aulast=Scullion)

221.

Sleep in Chronic Obstructive Pulmonary Disease.

Mohsenin V.

Embase

Sleep Medicine Clinics. 2(1) (pp 1-8), 2007. Date of Publication: March 2007.

[Review]

AN: 354477264

COPD is the fourth leading cause of death in the US, affecting 14 millions adults. Symptoms related to sleep disturbances are common in individuals with moderate to severe COPD, particularly in elderly patients. One cause of morbidity in this population is abnormalities in gas exchange and resultant hypoxemia, which can lead to elevated pulmonary pressures, dyspnea, and right ventricular overload and failure. Sleep has profound adverse effects on respiration and gas exchange in patients with COPD. Smoking cessation, bronchodilation, inhaled steroids in those with a reversible component, and pulmonary rehabilitation are cornerstones of treatment. Improvement in lung mechanics and gas exchange should lead to better sleep quality and health status. © 2007 Elsevier Inc. All rights reserved.

Status

Embase

Institution

(Mohsenin) Section of Pulmonary and Critical Care Medicine, Yale Center for Sleep Medicine, Yale University School of Medicine, 40 Temple Street, Suite 3C, New Haven, CT 06510, United States

Publisher

W.B. Saunders (Independence Square West, Philadelphia PA 19106-3399, United States)

Year of Publication

2007

Link to the Ovid Full Text or citation:

[Click here for full text options](https://ovidsp.ovid.com/ovidweb.cgi?T=JS&CSC=Y&NEWS=N&PAGE=fulltext&D=emed10&AN=354477264)

Link to the External Link Resolver:

[LibKey NHS](https://libkey.io/libraries/2789/openurl?genre=article&sid=OVID:emed10&genre=article&id=pmid:&id=doi:10.1016%2Fj.jsmc.2006.11.013&issn=1556-407X&volume=2&issue=1&spage=1&pages=1-8&date=2007&title=Sleep+Medicine+Clinics&atitle=Sleep+in+Chronic+Obstructive+Pulmonary+Disease&aulast=Mohsenin)

222.

Clinical and prognostic implications of cognitive dysfunction and depression in COPD.

Corsonello A., Pedone C., Incalzi R.A.

Embase

Current Respiratory Medicine Reviews. 3(2) (pp 107-115), 2007. Date of Publication: May 2007.

[Review]

AN: 46773917

In chronic obstructive pulmonary disease (COPD), cognitive dysfunction - mainly of secondary memory, executive, and constructional abilities - is a marker of disease severity with important clinical and prognostic implications. It correlates with poor compliance with pharmacological therapy, and poor compliance, in turn, increases the cost/effectiveness ratio of home care programs. Executive dysfunction is associated with difficulty with inhaler devices and should be systematically evaluated before prescribing. Drawing impairment has been associated with mortality, although the underlying neurologic abnormalities (right insular damage with autonomic dysfunction or subcortical damage) remain to be defined. Depression is also negatively correlated with survival, besides being a risk factor for quitting respiratory rehabilitation. Cognitive dysfunction should always be considered among the systemic effects of COPD and be the object of systematic screening in COPD patients. Identifying cognitive impairment would prompt either interventions promoting the adherence to the therapy or further investigations to exclude respiratory (nocturnal or effort hypoxemia, coexistent obstructive sleep apnoea) or non respiratory (decompensated diabetes, hypoglycaemic crises, poorly controlled hypertension etc.) causes of cognitive dysfunction. Cognitive enhancing strategies deserve consideration in the framework of a comprehensive approach aimed at improving the health status of people with COPD. © 2007 Bentham Science Publishers Ltd.

Status

Embase

Institution

(Corsonello) Istituto Nazionale di Ricovero e Cura per Anziani (INRCA), Cosenza, Italy (Pedone, Incalzi) Cattedra di Geriatria, Universita Campus BioMedico, Rome, Italy

(Incalzi) Fondazione San Raffaele, Cittadella della Carita, Taranto, Italy

(Corsonello) Viale della Resistenza Pal. Alfa Sca, I-87036 Rende, CS, Italy

Publisher

Bentham Science Publishers B.V. (P.O. Box 294, Bussum 1400 AG, Netherlands)

Year of Publication

2007

Link to the Ovid Full Text or citation:

[Click here for full text options](https://ovidsp.ovid.com/ovidweb.cgi?T=JS&CSC=Y&NEWS=N&PAGE=fulltext&D=emed10&AN=46773917)

Link to the External Link Resolver:

[LibKey NHS](https://libkey.io/libraries/2789/openurl?genre=article&sid=OVID:emed10&genre=article&id=pmid:&id=doi:10.2174%2F157339807780599162&issn=1573-398X&volume=3&issue=2&spage=107&pages=107-115&date=2007&title=Current+Respiratory+Medicine+Reviews&atitle=Clinical+and+prognostic+implications+of+cognitive+dysfunction+and+depression+in+COPD&aulast=Corsonello)

223.

Respirology year-in-review 2006: Clinical science.

Lee Y.C.G., Beasley R.

Embase

Respirology. 12(1) (pp 6-15), 2007. Date of Publication: January 2007.

[Review]

AN: 44968193

PMID

17207019 [<https://www.ncbi.nlm.nih.gov/pubmed/?term=17207019>]

Status

Embase

Institution

(Lee) Centre for Respiratory Research, University College London, London, United Kingdom (Lee) Oxford Centre of Respiratory Medicine, University of Oxford, Oxford, United Kingdom

(Beasley) University of Southampton, Southampton, United Kingdom

(Beasley) Medical Research Institute of New Zealand, Wellington, New Zealand

(Lee) Centre for Respiratory Research, University College London, 5 University Street, London WC1E 6JJ, United Kingdom

Publisher

Blackwell Publishing (550 Swanston Street, Carlton South VIC 3053, Australia)

Year of Publication

2007

Link to the Ovid Full Text or citation:

[Click here for full text options](https://ovidsp.ovid.com/ovidweb.cgi?T=JS&CSC=Y&NEWS=N&PAGE=fulltext&D=emed10&AN=44968193)

Link to the External Link Resolver:

[LibKey NHS](https://libkey.io/libraries/2789/openurl?genre=article&sid=OVID:emed10&genre=article&id=pmid:17207019&id=doi:10.1111%2Fj.1440-1843.2006.01004.x&issn=1323-7799&volume=12&issue=1&spage=6&pages=6-15&date=2007&title=Respirology&atitle=Respirology+year-in-review+2006%3A+Clinical+science&aulast=Lee)

224.

COPD and mood disorders, part 1: Anxiety and depression.

Yawn B.P., Giardino N.D., Criner G.J.

Embase

Journal of Respiratory Diseases. 28(3) (pp 94-103), 2007. Date of Publication: March 2007.

[Article]

AN: 46493649

Depression and anxiety are common comorbidities in patients with chronic obstructive pulmonary disease (COPD), and like COPD, they are often underrecognized. Both of these comorbidities can adversely affect the course of COPD. Anxiety, for example, is associated with more severe dyspnea, greater disability, and impaired functional status; it also is a significant predictor of hospitalizations for acute exacerbations of COPD. When evaluating depressive symptoms, it is important to rule out cognitive impairment, particularly in patients with severe COPD and hypoxemia. Treatment options include antidepressants and cognitive behavioral therapy. Participation in a pulmonary rehabilitation program also can help reduce anxiety and depressive symptoms in patients with COPD.

Status

Embase

Institution

(Yawn) Department of Family and Community Medicine, University of Minnesota, Minneapolis, MN, United States (Yawn) Olmsted Medical Center, Rochester, MN, United States

(Giardino) Department of Psychiatry, University of Michigan Health System, Ann Arbor, MI, United States

(Criner) Division of Pulmonary and Critical Care Medicine, Temple University School of Medicine, Philadelphia, United States

Publisher

Cliggott Publishing Co. (330 Boston Post Road, Box 4027, Darien CT 06820-4027, United States)

Year of Publication

2007

Link to the Ovid Full Text or citation:

[Click here for full text options](https://ovidsp.ovid.com/ovidweb.cgi?T=JS&CSC=Y&NEWS=N&PAGE=fulltext&D=emed10&AN=46493649)

Link to the External Link Resolver:

[LibKey NHS](https://libkey.io/libraries/2789/openurl?genre=article&sid=OVID:emed10&genre=article&id=pmid:&id=doi:&issn=0194-259X&volume=28&issue=3&spage=94&pages=94-103&date=2007&title=Journal+of+Respiratory+Diseases&atitle=COPD+and+mood+disorders%2C+part+1%3A+Anxiety+and+depression&aulast=Yawn)

225.

Medical Therapy for Chronic Obstructive Pulmonary Disease in 2007.

Ingenito E.P.

Embase

Seminars in Thoracic and Cardiovascular Surgery. 19(2) (pp 142-150), 2007. Date of Publication: 2007.

[Article]

AN: 47408678

Medical treatment for patients with stable chronic obstructive pulmonary disease (COPD) has evolved significantly over the past 2 decades. Current World Health Organization recommendations suggest a stepwise approach to therapy depending upon disease severity. As-needed use of short-acting bronchodilators is recommended for patients with mild disease. Scheduled dosing of bronchodilators is recommended for patients with more advanced disease. Inhaled beta-agonists and anti-cholinergic agents in combination have proved to be more effective than either agent alone. Long-acting preparations are associated with better disease control and have not been associated with tachyphylaxis. Inhaled corticosteroids are useful for reducing the frequency of exacerbations in patients who experience one or more episodes per year. Oxygen therapy is clearly beneficial in patients with advanced COPD and chronic respiratory failure, and its potential benefits in less severe disease are currently being studied. Pulmonary rehabilitation benefits patients with mild-to-severe disease, although the greatest benefits have been demonstrated in those with moderate COPD. New ultra-long-acting inhaled bronchodilators, phosphodiesterase inhibitors, protease inhibitors, and retinoids intended to promote tissue regeneration are currently being evaluated in clinical trials as future therapeutic agents. © 2007 Elsevier Inc. All rights reserved.

PMID

17870010 [<https://www.ncbi.nlm.nih.gov/pubmed/?term=17870010>]

Status

Embase

Institution

(Ingenito) Division of Pulmonary and Critical Care Medicine, Brigham and Women's Hospital, Boston, MA, United States

Publisher

W.B. Saunders (Independence Square West, Philadelphia PA 19106-3399, United States)

Year of Publication

2007

Link to the Ovid Full Text or citation:

[Click here for full text options](https://ovidsp.ovid.com/ovidweb.cgi?T=JS&CSC=Y&NEWS=N&PAGE=fulltext&D=emed10&AN=47408678)

Link to the External Link Resolver:

[LibKey NHS](https://libkey.io/libraries/2789/openurl?genre=article&sid=OVID:emed10&genre=article&id=pmid:17870010&id=doi:10.1053%2Fj.semtcvs.2007.06.003&issn=1043-0679&volume=19&issue=2&spage=142&pages=142-150&date=2007&title=Seminars+in+Thoracic+and+Cardiovascular+Surgery&atitle=Medical+Therapy+for+Chronic+Obstructive+Pulmonary+Disease+in+2007&aulast=Ingenito)

226.

Chronic obstructive pulmonary disease and sleep: The interaction.

Urbano F., Mohsenin V.

Embase

Panminerva Medica. 48(4) (pp 223-230), 2006. Date of Publication: December 2006.

[Review]

AN: 46361247

Chronic obstructive pulmonary disease (COPD) is the fourth leading cause of death, affecting 14 millions adults in the United States. Symptoms related to sleep disturbances are common in individuals with moderate to severe COPD, particularly in the elderly, which is commonly manifested as morning fatigue and early awakenings. One major cause of morbidity in this population is abnormalities in gas exchange and resultant hypoxemia as they can lead to elevated pulmonary pressures, dyspnea and in severe cases right ventricular overload and failure. Sleep has profound adverse effects on respiration and gas exchange in patients with COPD. There are several mechanisms underlying nonapneic oxygen desaturation during sleep. They include decreased functional residual capacity, diminished ventilatory responses to hypoxia and hypercapnia, impaired respiratory mechanics, diminished arousal, respiratory muscle fatigue, diminished nonchemical respiratory drive, increased upper airway resistance, and the starting point of baseline saturation values while awake on the oxyhemoglobin dissociation curve. Smoking cessation, bronchodilation, inhaled steroids in those with a reversible component and pulmonary rehabilitation are corner stones of treatment of COPD. The goals of therapy for the clinician should be to improve lung mechanics as well as gas exchange ultimately leading to better sleep quality and health status.

PMID

17215794 [<https://www.ncbi.nlm.nih.gov/pubmed/?term=17215794>]

Institution

(Urbano, Mohsenin) Section of Pulmonary and Critical Care Medicine, Yale University School of Medicine, Yale Center for Sleep Medicine, New Haven, CT, United States (Mohsenin) Yale Center for Sleep Medicine, Yale University, School of Medicine, 40 Temple Street, New Haven, CT 06510, United States

Publisher

Edizioni Minerva Medica S.p.A. (Corso Bramante 83-85, Torino 10126, Italy)

Year of Publication

2006

Link to the Ovid Full Text or citation:

[Click here for full text options](https://ovidsp.ovid.com/ovidweb.cgi?T=JS&CSC=Y&NEWS=N&PAGE=fulltext&D=emed9&AN=46361247)

Link to the External Link Resolver:

[LibKey NHS](https://libkey.io/libraries/2789/openurl?genre=article&sid=OVID:emed9&genre=article&id=pmid:17215794&id=doi:&issn=0031-0808&volume=48&issue=4&spage=223&pages=223-230&date=2006&title=Panminerva+Medica&atitle=Chronic+obstructive+pulmonary+disease+and+sleep%3A+The+interaction&aulast=Urbano)

227.

Cardiopulmonary rehabilitation and cancer rehabilitation. 2. Pulmonary rehabilitation review.

Alba A.S., Kim H., Whiteson J.H., Bartels M.N.

Embase

Archives of Physical Medicine and Rehabilitation. 87(3 SUPPL.) (pp 57-64), 2006. Date of Publication: March 2006.

[Review]

AN: 43286970

Pulmonary rehabilitation includes the rehabilitation of not only patients with respiratory failure in need of ventilatory support but also patients with primary pulmonary disease. New advances in medical management now offer treatment to patients with end-stage emphysema, pulmonary hypertension, and interstitial disease, and the principles of rehabilitation can add both function and quality to the lives of these patients. New surgical approaches and better transplantation outcomes that restore pulmonary function have also been introduced. Rehabilitation professionals need to be aware of these advances and be able to incorporate this knowledge into the practice of rehabilitation medicine. Overall Article Objectives: (a) To identify major categories of pulmonary disease seen in pulmonary rehabilitation, (b) to know appropriate interventions and support for patients with respiratory failure, (c) to describe the new interventions available for end-stage lung disease, and (d) to describe the appropriate pulmonary rehabilitation for people with pulmonary disease. © 2006 by the American Academy of Physical Medicine and Rehabilitation.

PMID

16500193 [<https://www.ncbi.nlm.nih.gov/pubmed/?term=16500193>]

Status

Embase

Institution

(Alba, Whiteson) Rusk Institute of Rehabilitation Medicine, New York University, School of Medicine, New York, NY, United States (Kim) Pediatric Rehabilitation Medicine, Children's Hospital of Philadelphia, University of Pennsylvania Health System, Philadelphia, PA, United States

(Bartels) Rehabilitation Medicine Department, Columbia University, College of Physicians and Surgeons, New York, NY, United States

(Alba) Coler-Goldwater Specialty Memorial Hospital and Nursing Facility, Dept. of Rehab. Med., E-3, 1 Main St, New York, NY 10044, United States

Publisher

W.B. Saunders (Independence Square West, Philadelphia PA 19106-3399, United States)

Year of Publication

2006

Link to the Ovid Full Text or citation:

[Click here for full text options](https://ovidsp.ovid.com/ovidweb.cgi?T=JS&CSC=Y&NEWS=N&PAGE=fulltext&D=emed9&AN=43286970)

Link to the External Link Resolver:

[LibKey NHS](https://libkey.io/libraries/2789/openurl?genre=article&sid=OVID:emed9&genre=article&id=pmid:16500193&id=doi:10.1016%2Fj.apmr.2005.12.007&issn=0003-9993&volume=87&issue=3+SUPPL.&spage=57&pages=57-64&date=2006&title=Archives+of+Physical+Medicine+and+Rehabilitation&atitle=Cardiopulmonary+rehabilitation+and+cancer+rehabilitation.+2.+Pulmonary+rehabilitation+review&aulast=Alba)

228.

Assurance of process-quality in pulmonary practice by means of a peer-review system - A pilot project. Sicherung der prozessqualitat in der pneumologischen praxis mittels peer-review-verfahren - Ein pilotprojekt <Sicherung der prozessqualitat in der pneumologischen praxis mittels peer-review-verfahren - Ein pilotprojekt.>

Fischer J., Kohler D., Magnussen H.

Embase

Pneumologie. 60(8) (pp 485-492), 2006. Date of Publication: August 2006.

[Review]

AN: 44358624

Quality assurance and quality-management have been well anchored in social legislation for years. Peer-reviews to assure process-quality and quality of outcomes have been conducted repeatedly in centres for pulmonary medicine, facilities for pulmonary rehabilitation as well as centres for sleep-medicine. By means of a pilot study we wanted to investigate if assurance of process-quality is feasible and if a peer review system turns out to be a suitable method in pulmonary practice. Medical reports and/or diagnostic protocols of the investigational procedures such as pulmonary function tests, allergy-tests, induction test of bronchial hyperresponsiveness et cetera were randomly collected from nine patients with bronchial asthma or COPD in each of the 44 participating pulmonary practices and forwarded to three peer-reviewers respectively. 396 selected patient-datasets resulted in 1188 reviews. Peer review was conducted by means of a specially developed checklist of markers of process-quality accompanied by an explanatory manual. Measures of quality were determined for every pulmonary practice and a report of results was generated. The health economical efficacy of the therapy and the diagnostic process as well as an assessment of the overall process was evaluated by means of a visual analogue scale. No deficiencies could be detected in clearly more than half of the assessments. Major deficiencies or an unacceptable result in different items could be detected in every sixth assessment. For every item a graphic ranking in terms of benchmarking was generated for each individual report. Since peers were reviewers and subjects of review at the same time, they can now clearly improve and assure their diagnostic and therapeutic process-quality in patients with bronchial asthma and COPD by means of the experience they gained during the review process, as well as by the assessment they received. We were able to demonstrate that a peer-review-system is appropriate to assure process-quality in pulmonary practice and that it is feasible to achieve a continuous assurance and improvement of quality in the long run. © Georg Thieme Verlag KG Stuttgart.

PMID

16933192 [<https://www.ncbi.nlm.nih.gov/pubmed/?term=16933192>]

Status

Embase

Institution

(Fischer) Institut fur Rehabilitationsforschung Norderney, Lehrstuhl fur Klinische Rehabilitations-Wissenschaften, Universitat Witten/Herdecke, Germany (Kohler) Krankenhaus Kloster Grafschaft, Schmallenberg, Germany

(Magnussen) Krankenhaus Groshansdorf, Zentrum fur Pneumologie und Thoraxchirurgie, Germany

(Fischer) Klinik Norderney der Deutschen Rentenversicherung Westfalen, Klinik der Universitat Witten/Herdecke, Kaiserstrase 26, 26548 Norderney, Germany

Publisher

Georg Thieme Verlag (Rudigerstrasse 14, Stuttgart D-70469, Germany)

Year of Publication

2006

Link to the Ovid Full Text or citation:

[Click here for full text options](https://ovidsp.ovid.com/ovidweb.cgi?T=JS&CSC=Y&NEWS=N&PAGE=fulltext&D=emed9&AN=44358624)

Link to the External Link Resolver:

[LibKey NHS](https://libkey.io/libraries/2789/openurl?genre=article&sid=OVID:emed9&genre=article&id=pmid:16933192&id=doi:10.1055%2Fs-2006-932212&issn=0934-8387&volume=60&issue=8&spage=485&pages=485-492&date=2006&title=Pneumologie&atitle=Sicherung+der+prozessqualitat+in+der+pneumologischen+praxis+mittels+peer-review-verfahren+-+Ein+pilotprojekt&aulast=Fischer)

229.

Effects of lung volume reduction surgery on sleep quality and nocturnal gas exchange in patients with severe emphysema.

Krachman S.L., Chatila W., Martin U.J., Nugent T., Crocetti J., Gaughan J., Criner G.J.

Embase

Chest. 128(5) (pp 3221-3228), 2005. Date of Publication: November 2005.

[Article]

AN: 41698643

Study objectives: We hypothesized that associated with improvements in respiratory mechanics, lung volume reduction surgery (LVRS) would result in an improvement in both sleep quality and nocturnal oxygenation in patients with severe emphysema.

Design(s): Prospective randomized controlled trial.

Setting(s): University hospital.

Patient(s): Sixteen patients (10 men, 63 +/- 6 years [+/- SD]) with severe airflow limitation (FEV1, 28 +/- 10% predicted) and hyperinflation (total lung capacity, 123 +/- 14% predicted) who were part of the National Emphysema Treatment Trial. Interventions and measurements: Patients completed 6 to 10 weeks of outpatient pulmonary rehabilitation. Spirometry, measurement of lung volumes, arterial blood gas analysis, and polysomnography were performed prior to randomization and again 6 months after therapy. Ten patients underwent LVRS and optimal medical therapy, while 6 patients received optimal medical therapy only.

Result(s): Total sleep time and sleep efficiency improved following LVRS (from 184 +/- 111 to 272 +/- 126 min [p = 0.007], and from 45 +/- 26 to 61 +/- 26% [p = 0.01], respectively), while there was no change with medical therapy alone (236 +/- 75 to 211 +/- 125 min [p = 0.8], and from 60 +/- 18 to 52 +/- 17% [p = 0.5], respectively). The mean and lowest oxygen saturation during the night improved with LVRS (from 90 +/- 7 to 93 +/- 4% [p = 0.05], and from 83 +/- 10 to 86 +/- 10% [p = 0.03], respectively), while no change was noted in the medical therapy group (from 91 +/- 5 to 91 +/- 5 [p = 1.0], and from 84 +/- 5 to 82 +/- 6% [p = 0.3], respectively). There was a correlation between the change in FEV1 and change in the lowest oxygen saturation during the night (r = 0.6, p = 0.02). In addition, there was an inverse correlation between the change in the lowest oxygen saturation during the night and the change in residual volume (- r = 0.5, p = 0.04) and functional residual capacity (- r = 0.6, p = 0.03).

Conclusion(s): In patients with severe emphysema, LVRS, but not continued optimal medical therapy, results in improved sleep quality and nocturnal oxygenation. Improvements in nocturnal oxygenation correlate with improved airflow and a decrease in hyperinflation and air trapping.

PMID

16304265 [<https://www.ncbi.nlm.nih.gov/pubmed/?term=16304265>]

Status

Embase

Institution

(Krachman, Chatila, Martin, Nugent, Crocetti, Criner) Division of Pulmonary and Critical Care Medicine, Temple University School of Medicine, Philadelphia, PA, United States (Gaughan) Division of Biostatistics, Temple University School of Medicine, Philadelphia, PA, United States

(Krachman) Temple University School of Medicine, 767 Parkinson Pavilion, Broad and Tioga Sts., Philadelphia, PA 19140, United States

Publisher

American College of Chest Physicians (3300 Dundee Road, Northbrook IL 60062-2348, United States)

Year of Publication

2005

Link to the Ovid Full Text or citation:

[Click here for full text options](https://ovidsp.ovid.com/ovidweb.cgi?T=JS&CSC=Y&NEWS=N&PAGE=fulltext&D=emed9&AN=41698643)

Link to the External Link Resolver:

[LibKey NHS](https://libkey.io/libraries/2789/openurl?genre=article&sid=OVID:emed9&genre=article&id=pmid:16304265&id=doi:10.1378%2Fchest.128.5.3221&issn=0012-3692&volume=128&issue=5&spage=3221&pages=3221-3228&date=2005&title=Chest&atitle=Effects+of+lung+volume+reduction+surgery+on+sleep+quality+and+nocturnal+gas+exchange+in+patients+with+severe+emphysema&aulast=Krachman)

230.

State of the art: Sequelae of tuberculosis.

Machida K., Maekura R.

Embase

Kekkaku. 80(10) (pp 655-674), 2005. Date of Publication: October 2005.

[Conference Paper]

AN: 41613698

Sequela of tuberculosis (TB sequela) is denned as the state with various secondary complications after healing of TB, such as chronic respiratory failure (CRF), cor pulmonale or chronic pulmonary inflammation. Pathophysiology of TB sequelae is consisted of disturbed pulmonary function, CRF, sleep disorder and pulmonary hypertention. In addition, secondary pulmonary infection with mycosis or nontuberculous mycobacteriosis (NTM) is difficult to be controlled. The issue of "chronic respiratory failure in patients with post-tuberculosis lung disorder-historical view and clinical" was presented by Dr Iwanaga. He demonstrated the overview of tuberculosis sequelae and compared the clinical course between with and without previous surgical intervention in patients with domiciliary oxygen therapy. In the NHO Toneyama National Hospital, the 10 years survival rate of TB sequelae patients with domiciliary oxygen therapy has been improving to over 70%. It seemed to be achieved to the goal of respiratory care for chronic respiratory failure caused by post-tuberculosis lung disorder. The mean age of these patients was consequently elevated to 72 years old. Most patients have required the health care service in their daily life. However, in post-tuberculosis patients, the grading of official respiratory disability was acknowledged as a lower level than that in COPD patients. Furthermore, there are many cases whose long-term care insurance has been unavailable. It could be argued that the public health care service for patients with TB sequelae should be improved. The strategy to improve the prognosis of survival has been shown by Dr Hiraga and Dr Tsuboi. Dr Hiraga presented "Order made pulmonary rehabilitation". It is that appropriate pulmonary rehabilitation according to the pathophysiology of individual patients based on the results of cardiopulmonary exercise testing, improves his or her prognosis. Dr Tsuboi presented "Noninvasive positive pressure ventilation" which can improve long-term survival and rescue the patients from acute deterioration. However, this strategy has not become popular in Japan. We would argue that the pulmonary rehabilitation strategy for patients with tuberculosis sequelae should be recommended. Dr Tatsumi presented "Pathophysiology and therapeutic strategies of pulmonary hypertension and sleep disorderd breathing in TB sequelae". He summarized that oxygen desaturation during sleep combines with pulmonary hypertension, so therapy of sleep oxygen desaturation can improve pulmonary hemodynamics and prognosis of patients with TB sequelae. Treatment of aspergillosis and NTM, especially MAC has remained difficult in spite of various efforts. Dr Ogawa presented "Medical intervention in pulmonary aspergillosis and NTM", especially related to blood level of CAM. Dr Ouchi presented "Surgical intervention in pulmonary aspergillosis and NTM" on his six year's experience. In summary, Dr Miwa proposed some important points for the management of patients with TB sequelae. These are constructed of management of elderly patients with complications, adequate diagnosis of disturbance and education of pulmonary rehabilitation to prevent worsening, administrative correspondence to welfare and treatment of the disabled, and cooperation with various patient parties. 1. Chronic respiratory failure in patients with post-tuberculosis lung disorder-Historical view and clinical survey: Tomoaki IWANAGA (NHO Fukuoka-Higashi Medical Center) Prior to emergence of effective agents, surgical procedures including thoracoplasty and lung resection were once popular for managing pulmonary tuberculosis. Collapse procedures such as artificial pneumothorax were also performed. These treatment, though unfortunately, lead to chronic respiratory failure because of deterioration of lung function in several decades. Clinical survey, underwent from 1998 through 2001 in national hospital network, of patients with newly-introduced domiciliary oxygen for this disorder revealed the different clinical course with or without previous surgical intervention. 2. Order made pulmonary rehabilitation: Toru HIRAGA, Ryoji MAEKURA and Seigo KITADA (Department of Pulmonary Medicine, NHO Toneyama National Hospital) We have been carried out cardiopulmonary exercise testing (CPET) in order to perform pulmonary rehabilitation (PR) appropriately and safely. In patients with tuberculosis sequelae (TBS), VO2, VE, V T, and O2-pulse at peak exercise were significantly lower, PaCO2 was significantly higher, and PaO2-Slope (DELTAPaO 2/DELTAVO2) was significantly steeper than those in patients with COPD during CPET. Low peak VO2, high VE/VO2, and steep PaO2-slope were significant poor prognostic predictors. The 6-minute walking distance and Borg scale were significantly improved by PR. The improvement of these parameters did not differ between TBS and COPD patients with a similar exercise capacity. The survival time of patients with TBS was significantly prolonged by PR. Especially, the effectiveness of PR for the survival of patients with poor prognosis (i.e. patients with steeper PaO2-slope) was clearly clarified. We also demonstrated that non-invasive positive pressure ventilation (NPPV) improved not only arterial blood gases but also VE/ VO2. So, the combination of PR and NPPV could be useful for improvement of the survival prognosis and QOL in patient with tuberculosis sequelae. Order made PR, appropriately programmed according to accurate assessment for patients' impairments, can give relief from dyspnea and increases exercise performance in patients with TBS. Consequently, PR can improve his or her prognosis. 3. Noninvasive positive pressure ventilation in post-tuberculosis patients: Tomomasa TSUBOI (Department of Respirology, NHO Minami-Kyoto Natioinal Hospital) There is sufficient clinical evidence to justify the use of long-term NPPV in patients with restrictive thoracic disease including post-tuberculosis. In those patients, long-term NPPV can improve their arterial blood gases and symptoms, reduce their hospital stay, and increase their QOL. Using T mode in long term NPPV can increase their survival rate more than using ST mode. For RTD patients, NPPV can also decrease rates of endotracheal intubation and reduce rates of mortality, in their acute exacerbation (NPPV success rate is more than 90% in our experience). 4. Pathophysiology and therapeutic strategies of pulmonary hypertension and sleep disordered breathing in sequelae of pulmonary tuberculosis: Koichiro TATSUMI, Tetsuya SAKUMA, Takayuki KURIYAMA (Department of Respirology, Graduate School of Medicine, Chiba University) Yuka SASAKI (Department of Chest Medicine, NHO Chiba-East National Hospital) Tsukasa YAMAMOTO (Department of Internal Medicine, Chiba Rosai Hospital) Sequelae of pulmonary tuberculosis are often associated with sleep disordered breathing, pulmonary hypertension and dyspnea on exertion, mainly due to constrictive and obstructive pulmonary impairements. Generally, pulmonary hypertension is deteriorated by hypoxemia due to sleep disordered breathing and exercise. Pulmonary hypertension is usually associated with a decrease in cardiac output and tissue oxygenation, and as a result, the decreased cardiac output and tissue oxygenation may further deteriorate the degree of desaturation during exercise and increase the magnitude of dyspnea on exertion, that is so called a vicious cycle. In this presentation, pathophysiology and therapeutic strategies are evaluated on the basis of previous studies in COPD and sequelae of pulmonary tuberculosis. Regarding pulmonary hypertension in sequelae of pulmonary tuberculosis, the first question is whether pulmonary hypertension at rest or during exercise is related to the prognosis or health status. The previous study suggested that the patients group, who had pulmonary hypertension, showed worse prognosis compared with those, who had not. Then, the answer is possibly yes. The second question is whether any kind of therapy such as oxygen inhalation, NPPV or drug therapy to pulmonary hypertension could improve the prognosis or health status. We have shown that the pulmonary hemodynamics had been improved by home oxygen therapy in patients with sequelae of pulmonary tuberculosis. Then the answer is also possibly yes. Regarding sleep disordered breathing in sequelae of pulmonary tuberculosis, the first question is whether sleep hypoxemia itself, associated with sleep disordered breathing, is related to the prognosis or health status. The previous studies about the influence of sleep desaturation on prognosis in patients with COPD suggested that multiple factors, including sleep desaturation, may be related to prognosis. Then, the answer is probably no. The second question is whether any kind of therapy such as oxygen inhalation, NPPV or drug therapy to sleep oxygen desaturation could improve the oxygen desaturation. The answer is definitely yes, because sleep oxygen desaturation is not due to sleep apnea in patients with sequelae of pulmonary tuberculosis. The third question is whether sleep oxygen desaturation is related to the progression of pulmonary hypertension in sequelae of pulmonary tuberculosis. The answer is possibly yes, because hypoxic pulmonary vasoconstriction could be one of the factors in the development of pulmonary hypertension. The forth question is whether the therapy to sleep oxygen desaturation could prevent the progression of pulmonary hypertension. The previous study suggested that home oxygen therapy had improved the degree of pulmonary hypertension. Then, the answer is possibly yes.

PMID

16296394 [<https://www.ncbi.nlm.nih.gov/pubmed/?term=16296394>]

Status

Embase

Institution

(Machida) Department of Pulmonary Medicine, National Hospital Organization (NHO) Tokyo National Hospital, Japan (Maekura) Department of Pulmonary Medicine, NHO Toneyama National Hospital, Japan

(Machida) Department of Pulmonary Medicine, NHO Tokyo National Hospital, 3-1-1, Takeoka, Kiyose-shi, Tokyo 204-8585, Japan

Publisher

Japanese Society for Tuberculosis (3-1-24 Matsuyama, Kiyose-shi, Tokyo 204, Japan)

Year of Publication

2005

Link to the Ovid Full Text or citation:

[Click here for full text options](https://ovidsp.ovid.com/ovidweb.cgi?T=JS&CSC=Y&NEWS=N&PAGE=fulltext&D=emed9&AN=41613698)

Link to the External Link Resolver:

[LibKey NHS](https://libkey.io/libraries/2789/openurl?genre=article&sid=OVID:emed9&genre=article&id=pmid:16296394&id=doi:&issn=0022-9776&volume=80&issue=10&spage=655&pages=655-674&date=2005&title=Kekkaku&atitle=State+of+the+art%3A+Sequelae+of+tuberculosis&aulast=Machida)

231.

Sleep in chronic obstructive pulmonary disease.

Mohsenin V.

Embase

Seminars in Respiratory and Critical Care Medicine. 26(1) (pp 109-116), 2005. Date of Publication: February 2005.

[Review]

AN: 40315557

Chronic obstructive pulmonary disease (COPD) is the fourth leading cause of death, affecting 14 million adults in the United States. Symptoms related to sleep disturbances are common in moderate to severe COPD, particularly in elderly patients, in the form of morning tiredness and early awakenings. One major cause of morbidity in this population is abnormalities in gas exchange and resultant hypoxemia. Sleep has profound adverse effects on respiration and gas exchange in patients with COPD. There are several mechanisms underlying nonapneic oxygen desaturation during sleep. They include decreased functional residual capacity, diminished ventilatory responses to hypoxia and hypercapnia, impaired respiratory mechanical effectiveness, diminished arousal responses, respiratory muscle fatigue, diminished nonchemical respiratory drive, increased upper airway resistance, and the position of baseline saturation values on the oxyhemoglobin dissociation curve. Smoking cessation, bronchodilation, and pulmonary rehabilitation are cornerstones of treatment of COPD. Improvement in lung mechanics and gas exchange should lead to better sleep quality and health status. Copyright © 2005 by Thieme Medical Publishers, Inc.

PMID

16052423 [<https://www.ncbi.nlm.nih.gov/pubmed/?term=16052423>]

Status

Embase

Institution

(Mohsenin) Yale Center for Sleep Medicine, Yale University School of Medicine, 40 Temple St., New Haven, CT 06510, United States (Mohsenin) Yale University School of Medicine, New Haven, CT, United States

(Mohsenin) Yale Center for Sleep Medicine, New Haven, CT, United States

Publisher

Thieme Medical Publishers, Inc. (333 7th Avenue, New York NY 10001-5004, United States)

Year of Publication

2005

Link to the Ovid Full Text or citation:

[Click here for full text options](https://ovidsp.ovid.com/ovidweb.cgi?T=JS&CSC=Y&NEWS=N&PAGE=fulltext&D=emed9&AN=40315557)

Link to the External Link Resolver:

[LibKey NHS](https://libkey.io/libraries/2789/openurl?genre=article&sid=OVID:emed9&genre=article&id=pmid:16052423&id=doi:10.1055%2Fs-2005-864204&issn=1069-3424&volume=26&issue=1&spage=109&pages=109-116&date=2005&title=Seminars+in+Respiratory+and+Critical+Care+Medicine&atitle=Sleep+in+chronic+obstructive+pulmonary+disease&aulast=Mohsenin)

232.

Advances in pulmonary rehabilitation and management of chronic respiratory failure. Introduction to the proceedings.

Lusuardi M., Donner C.F.

Embase

Monaldi Archives for Chest Disease. 59(3) (pp 224-226), 2003. Date of Publication: July/September 2003.

[Article]

AN: 38401420

In summary, there is a significant body of evidence, at least for COPD, that pulmonary rehabilitation programmes are effective in achieving the individual's best functional capability and health status. A socio-economic rationale for PR is, on the contrary, in need of solid confirmation in the setting of each different healthcare system; therefore, at present only specialist centres should be in charge of implementing PR programmes [8]. On the ground of evidence-based data and along the lines of controversial aspects of pulmonary Rehabilitation as defined above, the 4th International Conference on "Advances in Pulmonary Rehabilitation and Management of Chronic Respiratory Failure" was held in Stresa, Italy, from 19-22 March 2003. The conference was sponsored by the European Respiratory Society and the Italian Association of Hospital Pneumologists (AIPO) and promoted by the Salvatore Maugeri Foundation (Dept. of Respiratory Rehabilitation) and our journal. It has become a traditional appointment every 5 years to discuss topical arguments on Pulmonary Rehabilitation, each conference being focused on a definite subject of particular interest. This year the main topic was "Critical events in COPD and chronic respiratory failure", developed in a number of subtopics of particular relevance to pulmonary rehabilitation such as: predictors of survival after PR, preventing deterioration of chronic respiratory failure, long term management and home ventilation, causes of death in respiratory failure, PR of the severely disabled patient, coping strategies, cardiovascular comorbidities, outcome measures, nutrition, weaning from mechanical ventilation, and so on. In this and in the following issue of the journal the proceedings of the conference are published with the aim to give those attending the conference an extensive compendium of the scientific discussion and those who were unable to come the most updated material on the topical arguments in PR presented by some of the most distinguished Authors among the international experts of the discipline. In response to numerous requests from participants, it has been decided to hold this international event in future permanently in Stresa every 3 years, instead of 5 years as previously, in view of the more rapid acquisition of technical and scientific data. Hence, the 5th International Conference on Advances in Pulmonary Rehabilitation and Management of Chronic Respiratory Failure will be held in Stresa (Italy), on March 20-25, 2006. The main topic will be "From ICU to home: a multidisciplinary management approach" and will include subtopics such as rehabilitation in the ICU and transition to home, ventilatory assistance in the hospital, long-term ventilation at home, sleep disorders in the severe patient and definition of outcomes and optimization of health care resources.

PMID

15065321 [<https://www.ncbi.nlm.nih.gov/pubmed/?term=15065321>]

Status

Embase

Institution

(Lusuardi) Division Cardio-Pulmonary Rehab., S. Sebastiano Hospital, AUSL RE, Corregio, RE, Italy (Donner) Division of Pulmonary Disease, S. Maugeri Foundation IRCCS, Scientific Institute of Veruno, Veruno, NO, Italy

Publisher

PI-ME Tipografia Editrice S.r.l.

Year of Publication

2003

Link to the Ovid Full Text or citation:

[Click here for full text options](https://ovidsp.ovid.com/ovidweb.cgi?T=JS&CSC=Y&NEWS=N&PAGE=fulltext&D=emed8&AN=38401420)

Link to the External Link Resolver:

[LibKey NHS](https://libkey.io/libraries/2789/openurl?genre=article&sid=OVID:emed8&genre=article&id=pmid:15065321&id=doi:&issn=1122-0643&volume=59&issue=3&spage=224&pages=224-226&date=2003&title=Monaldi+Archives+for+Chest+Disease&atitle=Advances+in+pulmonary+rehabilitation+and+management+of+chronic+respiratory+failure.+Introduction+to+the+proceedings&aulast=Lusuardi)

233.

Relationship between sleep disordered breathing and body weight loss in patients with chronic obstructive pulmonary disease.

Ito E., Murata A., Yamamoto K., Kudo S.

Embase

Nihon Kokyuki Gakkai zasshi = the journal of the Japanese Respiratory Society. 41(4) (pp 268-275), 2003. Date of Publication: Apr 2003.

[Article]

AN: 36746921

We evaluated body weight loss and growth hormone secretion in patients with sleep-disordered breathing associated with chronic obstructive pulmonary disease. Of 11 patients hospitalized for pulmonary rehabilitation, five (WL group) had a history of body weight loss within two years before their interviews, while the other 6 patients (NWL group) had no changes in body weight. All patients underwent body index measurements, pulmonary function tests, blood gas analyses, assessments of nutritional status, and full night polysomnography for two consecutive days. Growth hormone levels were measured in the first 3-hour period following falling asleep. There were no significant inter-group differences between the results of pulmonary function tests, blood gas analyses, or nutritional status assessment. The WL group had a significantly higher percentage loss of body weight than the NWL group (mean +/- S.D. 11.5 +/- 4.7% in the WL group versus 2.7 +/- 1.8% in the NWL group, p < 0.01). The WL group had a significantly higher sleep apnea/hypopnea index than the NWL group (42.4 +/- 9.5/hr in the WL group versus 7.8 +/- 2.9/hr in the NWL group, p < 0.01). The WL group showed a higher rate of stage I + II sleep than the NWL group (84.9 +/- 7.0% versus 64.5 +/- 8.7%), with lower rates of slow wave sleep (2.2 +/- 2.1% versus 15.0 +/- 8.7%) and rapid eye movement sleep (12.9 +/- 6.3% versus 20.6 +/- 1.0%). The WL group showed a low level of growth hormone secretion with no peak in the sequential curve, but had a higher level of insulin growth factor-1 than the NWL group (148 +/- 36 ng/ml versus 90 +/- 22 ng/ml, p < 0.01). These results suggest that chronic obstructive pulmonary disease patients undergoing weight loss are likely to have an increase of growth hormone secretions in the daytime, possibly induced by underlying psychiatric disorders such as depression. Patients with chronic obstructive pulmonary disease may lose weight regardless of nutritional status because of a disturbance of growth hormone secretion resulting of sleep-disordered breathing.

PMID

12795181 [<https://www.ncbi.nlm.nih.gov/pubmed/?term=12795181>]

Institution

(Ito, Murata, Yamamoto, Kudo) 4th Department of Internal Medicine, Nippon Medical School, 1-1-5 Sendagi, Bunkyo-ku, Tokyo 113-8603, Japan.

Year of Publication

2003

Link to the Ovid Full Text or citation:

[Click here for full text options](https://ovidsp.ovid.com/ovidweb.cgi?T=JS&CSC=Y&NEWS=N&PAGE=fulltext&D=emed8&AN=36746921)

Link to the External Link Resolver:

[LibKey NHS](https://libkey.io/libraries/2789/openurl?genre=article&sid=OVID:emed8&genre=article&id=pmid:12795181&id=doi:&issn=1343-3490&volume=41&issue=4&spage=268&pages=268-275&date=2003&title=Nihon+Kokyuki+Gakkai+zasshi+%3D+the+journal+of+the+Japanese+Respiratory+Society&atitle=Relationship+between+sleep+disordered+breathing+and+body+weight+loss+in+patients+with+chronic+obstructive+pulmonary+disease&aulast=Ito)

234.

Translating new understanding into better care for the patient with chronic obstructive pulmonary disease.

Pierson D.J.

Embase

Respiratory care. 49(1) (pp 99-109), 2004. Date of Publication: Jan 2004.

[Article]

AN: 38563074

Despite an enormous amount of research and many official statements, the definition, diagnosis, and staging of chronic obstructive pulmonary disease (COPD) remain inconsistent, and we have yet to agree on who should be tested with spirometry or on where and how to do it. We know that inflammation, not just airflow limitation, is important in determining the course of COPD, especially with respect to exacerbations. We can detect and treat alpha-1 antitrypsin deficiency, an under-recognized condition, but whether alpha-1 antitrypsin augmentation therapy affects the disease's clinical course remains unclear. Smoking cessation is the most important of all interventions for COPD, with proven techniques and adjuncts, but implementation remains difficult and success rates are disappointingly low. Similarly, pulmonary rehabilitation has well-documented benefits but is grossly underutilized because it is difficult to pay for and is not made available to most patients. Symptoms, costs, and other outcomes can be improved through comprehensive disease management, including the use of practice guidelines, yet multiple barriers prevent the potential benefits of these interventions to patients from being realized. Many patients who do not meet threshold oxygenation criteria for oxygen therapy during the daytime desaturate during sleep, but evidence that nocturnal oxygen administration benefits these patients is lacking. However, other sleep-related breathing disorders are common in COPD patients. Lung volume reduction surgery has recently been shown to improve function and survival for certain COPD patients, but lung transplantation has generally been disappointing. New pharmaceutical agents are being developed for treating COPD, and at least one of them (tiotropium) should soon be available in the United States. Noninvasive ventilation is effective in treating acute decompensations of COPD and should be the standard of care in that setting; evidence supporting its use in stable patients with end-stage disease is scant. Appropriate palliative care can greatly benefit patients and their families in the terminal phase of COPD and needs to be more widely applied.

PMID

14733626 [<https://www.ncbi.nlm.nih.gov/pubmed/?term=14733626>]

Institution

(Pierson) Division of Pulmonary and Critical Care Medicine, Harborview Medical Center and University of Washington, 325 Ninth Avenue, Box 359762, Seattle WA 98104, USA.

Year of Publication

2004

Link to the Ovid Full Text or citation:

[Click here for full text options](https://ovidsp.ovid.com/ovidweb.cgi?T=JS&CSC=Y&NEWS=N&PAGE=fulltext&D=emed8&AN=38563074)

Link to the External Link Resolver:

[LibKey NHS](https://libkey.io/libraries/2789/openurl?genre=article&sid=OVID:emed8&genre=article&id=pmid:14733626&id=doi:&issn=0020-1324&volume=49&issue=1&spage=99&pages=99-109&date=2004&title=Respiratory+care&atitle=Translating+new+understanding+into+better+care+for+the+patient+with+chronic+obstructive+pulmonary+disease&aulast=Pierson)

235.

Oxygen saturation at rest, on exercise and during sleep in patients with COPD underogoing pulmonary rehabilitation program. Two years, prospective, controlled study. Kontrolowane badanie przezskorne wysycenia tlenem krwi tetniczej w czasie dnia, w nocy oraz w czasie wysilku u rehabilitowanych chorych na pochp <Kontrolowane badanie przezskorne wysycenia tlenem krwi tetniczej w czasie dnia, w nocy oraz w czasie wysilku u rehabilitowanych chorych na pochp.>

Piszko P., Lewczuk J., Kowalska-Superlak M., Wrabec K.

Embase

Pneumonologia i Alergologia Polska. 70(11-12) (pp 566-572), 2002. Date of Publication: 2002.

[Article]

AN: 36342535

The role of pulmonary rehabilitation (PR) in COPD patients is well established. However, the PR programs includes physical exercise that may deteriorate chronic hypoxemia. The objective of the study was to evaluate impact of a long term PR program on oxygen saturation at rest, on exercise and during sleep in COPD patients not receiving supplemental oxygenation. A two - year controlled study involved 27 rehabilitated (PR-COPD patients (6 females) aged 32-76 years, mean 58.1 years on overage and 19 control (C-COPD patients (3 females) aged 58-83 years, mean 68. COPD patients participating in PR program patients were younger, had higher value of FVC and FEV1 and better results of METs during symptom-limited exercise test (ET, p=0.007). They also had better results of transcutaneus oxygen saturation (SaO2) at rest (p=0.02), a lower maximal decrease in SaO2 during ET(p=0.003) and a higher value of medan SaO2 during (p=0.02). Following PR program the number of METs achieved during ET increased in PR-COPD patients (p=0.007) and did not change in C-COPD, p=NS. The SaO2 at rest did not change significantly in both groups. Maximal drop in SaO2 during ET decreased in PR-COPD patients (p=0.02) and did not change in C-COPD(p=NS) group. Median SaO2 at sleep did not change in PR-COPD patients (p=NS) but it decreased in C-COPD patients (p=0.02). The study indicates that long term PR does not deteriorate the resting diurnal hypoxaemia, it prevent COPD patients from time depending exacerbation of nocturnal hypoxaemia and can improve oxygen saturation on exertion. The results could be influenced by baseline differences between PR-COPD and C-COPD patients.

PMID

12884566 [<https://www.ncbi.nlm.nih.gov/pubmed/?term=12884566>]

Status

Embase

Institution

(Piszko, Lewczuk, Kowalska-Superlak, Wrabec) Oddzial Kardiologicznego Wojewodzki, Szpital Specjalistycznego, ul Kamienskiego 73a, 51-124 Wroclaw, Poland

Publisher

Instytut Gruzlicy i Chorob Pluc (ul. Plocka 26, Warsaw 01-138, Poland)

Year of Publication

2002

Link to the Ovid Full Text or citation:

[Click here for full text options](https://ovidsp.ovid.com/ovidweb.cgi?T=JS&CSC=Y&NEWS=N&PAGE=fulltext&D=emed7&AN=36342535)

Link to the External Link Resolver:

[LibKey NHS](https://libkey.io/libraries/2789/openurl?genre=article&sid=OVID:emed7&genre=article&id=pmid:12884566&id=doi:&issn=0867-7077&volume=70&issue=11-12&spage=566&pages=566-572&date=2002&title=Pneumonologia+i+Alergologia+Polska&atitle=Kontrolowane+badanie+przezskorne+wysycenia+tlenem+krwi+tetniczej+w+czasie+dnia%2C+w+nocy+oraz+w+czasie+wysilku+u+rehabilitowanych+chorych+na+pochp&aulast=Piszko)

236.

Management of chronic obstructive pulmonary disease (COPD).

Gibson P.G.

Embase

Australian Prescriber. 24(6) (pp 152-155), 2001. Date of Publication: 2001.

[Review]

AN: 34000959

The expiratory airflow obstruction that characterises chronic obstructive pulmonary disease is usually progressive over time and caused by emphysema, obliterative bronchiolitis, and mucus hypersecretion. Stopping smoking is the only measure that slows the progression of chronic obstructive pulmonary disease, and smokers should be encouraged to stop at all stages of the disease. The effects of medication are limited, and need to be balanced against cost and adverse effects. Bronchodilators, given by puffer and spacer rather than by nebuliser, are effective. Avoid inhaled corticosteroids unless there is associated asthma. Pulmonary rehabilitation leads to important improvements in quality of life. Influenza vaccination is helpful. Comorbidity from cardiac disease and sleep disordered breathing are common and can be effectively treated. New therapies under evaluation include lung volume reduction surgery, non-invasive ventilation, and anti-inflammatory drugs.

Status

Embase

Institution

(Gibson) Department of Respiratory and Sleep Medicine, John Hunter Hospital, Australia (Gibson) School of Medical Practice, Faculty of Medicine, University of Newcastle, Australia

(Gibson) Asthma, Infection and Immunology Research Group, Hunter Medical Research Institute, Newcastle, NSW, Australia

Publisher

National Prescribing Service (3/2 Phipps Close, Deakin ACT 2602, Australia)

Year of Publication

2001

Link to the Ovid Full Text or citation:

[Click here for full text options](https://ovidsp.ovid.com/ovidweb.cgi?T=JS&CSC=Y&NEWS=N&PAGE=fulltext&D=emed7&AN=34000959)

Link to the External Link Resolver:

[LibKey NHS](https://libkey.io/libraries/2789/openurl?genre=article&sid=OVID:emed7&genre=article&id=pmid:&id=doi:10.18773%2Faustprescr.2001.158&issn=0312-8008&volume=24&issue=6&spage=152&pages=152-155&date=2001&title=Australian+Prescriber&atitle=Management+of+chronic+obstructive+pulmonary+disease+(COPD)&aulast=Gibson)

237.

Long-term oxygen therapy.

Wedzicha J.A.

Embase

European Respiratory Monograph. 5(13) (pp 143-154), 2000. Date of Publication: 2000.

[Review]

AN: 30230189

Long-term oxygen therapy has a vital role in pulmonary rehabilitation and is one of the few interventions that reduces mortality in hypoxaemic chronic obstructive pulmonary disease patients. Other effects include improvement in sleep quality, neuropsychological benefits and reduction of secondary polycythaemia. Prescription of long-term oxygen therapy should include appropriate education and a home care programme. Ambulatory oxygen therapy is effective in patients who develop oxygen desaturation on exercise, with improvement in exercise capacity and a greater use of daily oxygen therapy. Careful patient assessment is required to assess the effects and to chose the appropriate oxygen flow rate. There have been few longer term studies, using appropriate outcome measures, of the use of ambulatory oxygen in this patient group. Patients on long-term oxygen therapy should be provided with ambulatory oxygen during a physical training programme, though the role of ambulatory oxygen in the rehabilitation of patients who show desaturation during exercise only, without chronic hypoxaemia, requires further study. Any ambulatory oxygen device provided for rehabilitation must be appropriate to the expected usage of the particular patient.

Status

Embase

Institution

(Wedzicha) Academic Respiratory Medicine, Royal London Sch. of Med. and Dent., St Bartholomew's Hospital, West Smithfield, London EC1A 7BE, United Kingdom

Publisher

Maney Publishing (Suite 1C, Joseph's Well, Hanover Walk, Leeds LS3 1AB, United Kingdom)

Year of Publication

2000

Link to the Ovid Full Text or citation:

[Click here for full text options](https://ovidsp.ovid.com/ovidweb.cgi?T=JS&CSC=Y&NEWS=N&PAGE=fulltext&D=emed7&AN=30230189)

Link to the External Link Resolver:

[LibKey NHS](https://libkey.io/libraries/2789/openurl?genre=article&sid=OVID:emed7&genre=article&id=pmid:&id=doi:&issn=1025-448X&volume=5&issue=13&spage=143&pages=143-154&date=2000&title=European+Respiratory+Monograph&atitle=Long-term+oxygen+therapy&aulast=Wedzicha)

238.

Selection of candidates and programmes.

Donner C.F., Lusuardi M.

Embase

European Respiratory Monograph. 5(13) (pp 132-142), 2000. Date of Publication: 2000.

[Review]

AN: 30230188

Candidate for a rehabilitation programme is theoretically any nonsmoking symptomatic chronic respiratory patient willing to participate, but prime candidates are those patients with more severe deconditioning, muscle weakness and exercise intolerance. A global evaluation of the patient is necessary within the conceptual frame of health status and functional assessment, including physical functioning, emotional functioning, sense of well-being, social functioning and social role. The composition of a rehabilitation programme has to be tailored to the individual patient. Rehabilitation deals in most cases with incurable diseases and results of different programmes require periodical reinforcing sessions. Planning and performance of rehabilitation programmes must rely on team cooperation, including a chest physician as coordinator, nurses, physiotherapists, respiratory therapists and for particular purposes an occupational therapist, psychologist, dietician, social worker, and speech therapist. Inpatient rehabilitation should be reserved for patients with unstable disease, patients needing complex functional evaluation, and patients with major mobility problems. Outpatient programmes are generally reserved for subjects with mild to moderate disease who still lead an active life, and in particular for those cases where only periodic follow-up is needed, but also severe, relatively stable patients may be considered for inclusion. Home respiratory care programmes are designed to provide the best possible lifestyle for the individual, ideally at significant savings in costs. A well structured home care programme can assure the treatment of the patient with most of the therapeutic modalities available in the hospital setting. Most experience in pulmonary rehabilitation has been accumulated in the management of patients with chronic obstructive pulmonary disease, but several other conditions are worth considering, such as cystic fibrosis, asthma, thoracic wall deformities, neuromuscular disorders, postsurgical sequelae, sleep- related disorders.

Status

Embase

Institution

(Donner, Lusuardi) Division of Pulmonary Disease, Medical Center of Rehabilitation, I-28010 Veruno (NO), Italy

Publisher

Maney Publishing (Suite 1C, Joseph's Well, Hanover Walk, Leeds LS3 1AB, United Kingdom)

Year of Publication

2000

Link to the Ovid Full Text or citation:

[Click here for full text options](https://ovidsp.ovid.com/ovidweb.cgi?T=JS&CSC=Y&NEWS=N&PAGE=fulltext&D=emed7&AN=30230188)

Link to the External Link Resolver:

[LibKey NHS](https://libkey.io/libraries/2789/openurl?genre=article&sid=OVID:emed7&genre=article&id=pmid:&id=doi:&issn=1025-448X&volume=5&issue=13&spage=132&pages=132-142&date=2000&title=European+Respiratory+Monograph&atitle=Selection+of+candidates+and+programmes&aulast=Donner)

239.

Effects of pulmonary rehabilitation in chronic obstructive pulmonary disease out patients on exercise tolerance and quality of life. EFFETS DE LA REHABILITATION RESPIRATOIRE EN AMBULATOIRE SUR LA TOLERANCE A L'EFFORT ET LA QUALITE DE VIE DES PATIENTS ATTEINTS DE BRONCHOPNEUMOPATHIE CHRONIQUE OBSTRUCTIVE <EFFETS DE LA REHABILITATION RESPIRATOIRE EN AMBULATOIRE SUR LA TOLERANCE A L'EFFORT ET LA QUALITE DE VIE DES PATIENTS ATTEINTS DE BRONCHOPNEUMOPATHIE CHRONIQUE OBSTRUCTIVE.>

Grosbois J.-M., Douay B., Fortin F., Dernis J.-M., Lemaire B., Boutemy J., Gosset P., Plez R., Van-Hecke A., Vanpoulle C.

Embase

Revue des Maladies Respiratoires. 13(1) (pp 61-67), 1996. Date of Publication: March 1996.

[Article]

AN: 26157816

Respiratory rehabilitation is a multidisciplinary medical approach which allows a total care of patients suffering from COPD. Optimisation of bronchodilator treatment, health education, cessation of smoking, dietetic, relaxation and re-entrainment to effort. We report our experience concerning 88 BPCO (mean age 62.1, FEV1 of 1.4 litres, or 48% of predicted normal); these 88 patients were cared for on an ambulatory basis at our centre for two hours per session, three times per week for seven weeks. The objective results were analysed on exercise tests before and after treatment. For ventilation, there was a significant improvement in the power developed (from 45.5 +/- 17.1 to 53.4 +/- 23 watts; p < 0.001) without any change in the oxygen consumption (VO2), ventilation (VE) or heart rate (FC) and of oxygen pulse (VO2/FC). For the same level of power (80% of maximum power for the initial exercise test) there was a significant lowering of ventilation (V = 33.5 +/- 9.4 to 30.7 +/- 7.4 litres per minute, p < 0.001), cardiac frequency (FC: from 116.9 +/- 16 to 111.1 +/- 13.1 beats per minute, p < 0.001) as well as the oxygen pulse (VO2/FC: from 7.9 +/- 2.7 to 8.3 +/- 3.7). At the maximum on the exercise test all the parameters studied were significantly better: watts, VO2, VE, cardiac frequency and VO2/FC. A study of the visual analogue scale (EVA), analysing sleep, anxiety, dyspnoea and the physical aspects showed a significant improvement in the four subjective parameters. Respiratory rehabilitation of BPCO practised as an out patient has shown an improvement in exercise tolerance in every day activities and improvement in dyspnoea and in the quality of life.

PMID

8650419 [<https://www.ncbi.nlm.nih.gov/pubmed/?term=8650419>]

Status

Embase

Institution

(Grosbois, Douay, Fortin, Dernis, Lemaire, Boutemy, Gosset, Plez, Van-Hecke, Vanpoulle) CLEFAR Rehabilitation Respiratoire, Clinique de la Louviere, 69, rue de la Louviere, F 59800 Lille, France

Publisher

Elsevier Masson SAS (62 rue Camille Desmoulins, Issy les Moulineaux Cedex 92442, France)

Year of Publication

1996

Link to the Ovid Full Text or citation:

[Click here for full text options](https://ovidsp.ovid.com/ovidweb.cgi?T=JS&CSC=Y&NEWS=N&PAGE=fulltext&D=emed6&AN=26157816)

Link to the External Link Resolver:

[LibKey NHS](https://libkey.io/libraries/2789/openurl?genre=article&sid=OVID:emed6&genre=article&id=pmid:8650419&id=doi:&issn=0761-8425&volume=13&issue=1&spage=61&pages=61-67&date=1996&title=Revue+des+Maladies+Respiratoires&atitle=EFFETS+DE+LA+REHABILITATION+RESPIRATOIRE+EN+AMBULATOIRE+SUR+LA+TOLERANCE+A+L'EFFORT+ET+LA+QUALITE+DE+VIE+DES+PATIENTS+ATTEINTS+DE+BRONCHOPNEUMOPATHIE+CHRONIQUE+OBSTRUCTIVE&aulast=Grosbois)

240.

Rehabilitation of hypoxemic patients with COPD at low altitude at the Dead Sea, the lowest place on earth.

Kramer M.R., Springer C., Berkman N., Glazer M., Bublil M., Bar-Yishay E., Godfrey S.

Embase

Chest. 113(3) (pp 571-575), 1998. Date of Publication: 1998.

[Article]

AN: 28130925

Background: In patients with COPD, oxygen therapy has been shown to improve exercise capacity and survival. Increase in barometric pressure at low altitude can serve as a simple way to improve arterial oxygenation in hypoxemic patients. We have tried to evaluate the effect of staying at low altitude on arterial oxygenation and exercise performance in patients with COPD. Patients and method: Eleven patients with COPD (9 male, 2 female) aged 38 to 79 years (mean FEV1, 0.96 L; 36% predicted) with hypoxemia (mean PaO2, 54.2 +/- 8.9 mm Hg) at Jerusalem (altitude 800 m above sea level) were taken down to the Dead Sea area (altitude 402 m below sea level) for 3 weeks. At both locations we tested arterial blood gases, spirometry, progressive exercise, 6-minute walking distance, and sleep oximetry. The study was repeated 2 weeks after returning to Jerusalem.

Result(s): Spirometry results were unchanged. Mean arterial PaO2 rose from 54.2 +/- 8.9 mm Hg to 69.5 +/- 11 at the first week and to 66.6 +/- 11 at the third week of stay (p<0.001). PaCO2 rose from 43.5 +/- 9.8 mm Hg to 47.7 +/- 9 and 49.5 +/- 8.4 (p<0.006). Six-minute walking distance rose from 337 +/- 107 m to 449 +/- 73 and 507 +/- 91 in the third week (p<0.005). Maximum oxygen consumption (VO2max) rose from 901 +/- 257 mL/min to 1,099 +/- 255 and 1,063 +/- 250 mL/min (p=0.01). Sleep oximetry showed an increase in mean sleep arterial oxygen saturation from 86.0 +/- 4.3% to 89.9 +/- 4.2% and 88.3 +/- 3.0 at 1 and 3 weeks, respectively (p<0.05). Following the return to Jerusalem, arterial gases returned to their baseline levels (PaO2, 52.9 +/- 9.4 mm Hg) but 6-min walking distance remained significantly high, 453 +/- 47 (p<0.02), and VO2max remained high as well (1,102 +/- 357 mL/min), although it did not reach statistical significance.

Conclusion(s): Decline to low altitude or staying at high oxygen environment improves arterial oxygenation and exercise capacity in hypoxemic patients residing in moderate or high altitude. Low altitude (or pressurized wards) can improve pulmonary rehabilitation of hypoxemic patients with COPD.

PMID

9515826 [<https://www.ncbi.nlm.nih.gov/pubmed/?term=9515826>]

Status

Embase

Institution

(Kramer, Springer, Berkman, Glazer, Bublil, Bar-Yishay, Godfrey) Pulmonary Institute, Hadassah Univ. Hospital, Ein Kerem, Hebrew University, Jerusalem, Israel (Kramer) Pulmonary Institute, Rabin Medical Center, (Beilinson) Petach Tikra 49100, Israel

Publisher

American College of Chest Physicians (3300 Dundee Road, Northbrook IL 60062-2348, United States)

Year of Publication

1998

Link to the Ovid Full Text or citation:

[Click here for full text options](https://ovidsp.ovid.com/ovidweb.cgi?T=JS&CSC=Y&NEWS=N&PAGE=fulltext&D=emed6&AN=28130925)

Link to the External Link Resolver:

[LibKey NHS](https://libkey.io/libraries/2789/openurl?genre=article&sid=OVID:emed6&genre=article&id=pmid:9515826&id=doi:10.1378%2Fchest.113.3.571&issn=0012-3692&volume=113&issue=3&spage=571&pages=571-575&date=1998&title=Chest&atitle=Rehabilitation+of+hypoxemic+patients+with+COPD+at+low+altitude+at+the+Dead+Sea%2C+the+lowest+place+on+earth&aulast=Kramer)

241.

Guidelines for the management of chronic obstructive pulmonary disease.

Bateman E.D., Abdullah I., Ainslie G., Bardin P., Blott J., Coetzee A., Cohen D., Feldman C., Greenblatt M., Jansen J., Joubert J., Lalloo U., Moodley Y., Morar R., Mzileni O., O'Brien J., Otto W., Pansegrouw D., Flit M., Raine R., Kamdar M., Richards G., Tintinger G., Van Der Wal B., Van Schalkwyk E., Visser S., Walzl G., Wong M.

Embase

South African Medical Journal. 88(8) (pp 999-1010), 1998. Date of Publication: 1998.

[Article]

AN: 28431836

Objective. This guideline has been developed in order to optimise the management of patients with chronic obstructive pulmonasy disease (COPD) at all levels of the health care system in South Africa. It contains an action plan for early recognition and appropriate treatment of this common condition. Options. Treatment regimens are recommended for patients with mild (stage I), moderate (stage II) and severe (stage III) disease. Outcomes. Optimal management of patients with COPD may achieve a reduction in breathlessness, improved quality of life, prevention of complications and limitation of disease progression. Enidence. The Working Group comprised mainly pulmonologists, but included an anaesthetist, a pharmacologist and a physiotherapist. Detailed literature review with particular attention to similar guideline documents from Europe and the USA was performed before the meeting. Recommendations. Steps in the management of patients with COPD include early recognition of the disease, smoking cessation, treatment of airflow obstruction with appropriate drugs (singly or in combination), education and pulmonary rehabilitation, and limitation of disease progression and complications. Detailed recommendations are made with regard to the use and interpretation of a trial of oral corticosteroid therapy. Indications for hospitalisation, intensive care unit admission and ventilatory support are provided. Validation. This guideline is similar to those recommended by other groups outside South Africa. It was developed by a working group of the South African Pulmonology Society and is endorsed by the Medical Association of South Africa. Sponsors. The meeting of the Working Group was sponsored by Boehringer Ingelheim. This sponsorship did not influence the activities of the Group.

PMID

9754214 [<https://www.ncbi.nlm.nih.gov/pubmed/?term=9754214>]

Status

Embase

Institution

(Bateman, Abdullah, Ainslie, Bardin, Blott, Coetzee, Cohen, Feldman, Greenblatt, Jansen, Joubert, Lalloo, Moodley, Morar, Mzileni, O'Brien, Otto, Pansegrouw, Flit, Raine, Kamdar, Richards, Tintinger, Van Der Wal, Van Schalkwyk, Visser, Walzl, Wong) PO Box 16433, Vlaeberg, 8018

Publisher

South African Medical Association (Private Bag X1, Pinelands 7430, South Africa)

Year of Publication

1998

Link to the Ovid Full Text or citation:

[Click here for full text options](https://ovidsp.ovid.com/ovidweb.cgi?T=JS&CSC=Y&NEWS=N&PAGE=fulltext&D=emed6&AN=28431836)

Link to the External Link Resolver:

[LibKey NHS](https://libkey.io/libraries/2789/openurl?genre=article&sid=OVID:emed6&genre=article&id=pmid:9754214&id=doi:&issn=0256-9574&volume=88&issue=8&spage=999&pages=999-1010&date=1998&title=South+African+Medical+Journal&atitle=Guidelines+for+the+management+of+chronic+obstructive+pulmonary+disease&aulast=Bateman)

242.

Disease management of COPD with pulmonary rehabilitation.

Tiep B.L.

Embase

Chest. 112(6) (pp 1630-1656), 1997. Date of Publication: 1997.

[Review]

AN: 28007773

Pulmonary rehabilitation is a set of tools and disciplines that attends to the multiple needs of the COPD patient. It extends beyond standard care by addressing the disabling features of chronic and progressive lung disease. It centers on self-management, exercise, functional training, psychosocial skills, and contributes to the optimization of medical management. Exercise enables other components by building strength, endurance, confidence, and reducing dyspnea. Patients who have undergone rehabilitation often enjoy a reduced need for health-care utilization. On the downside, rehabilitation is a one-time intervention, the benefits of which dissolve over time. The patient's physician is rarely a participant in the program; thus, the physician is at a disadvantage in being able to support a long-term response. Rehabilitation is available to a small percentage of a large patient population who could benefit. Optimal disease management would entail redesigning standard medical care to integrate rehabilitative elements into a system of patient self-management and regular exercise. It should emphasize physician involvement in self-management, which is essential in developing and maintaining an effective exacerbation protocol. Pulmonary rehabilitation should take its place in the mainstream of disease management through its integrative and reconciliative role in the multidisciplinary continuum of services, as defined by the National Institutes of Health, Pulmonary Rehabilitation Research, Workshop of 1994.

PMID

9404764 [<https://www.ncbi.nlm.nih.gov/pubmed/?term=9404764>]

Status

Embase

Institution

(Tiep) Pulmonary Care Continuum at Halcyon, 5467 Second St., Irwindale, CA 91706, United States

Publisher

American College of Chest Physicians (3300 Dundee Road, Northbrook IL 60062-2348, United States)

Year of Publication

1997

Link to the Ovid Full Text or citation:

[Click here for full text options](https://ovidsp.ovid.com/ovidweb.cgi?T=JS&CSC=Y&NEWS=N&PAGE=fulltext&D=emed6&AN=28007773)

Link to the External Link Resolver:

[LibKey NHS](https://libkey.io/libraries/2789/openurl?genre=article&sid=OVID:emed6&genre=article&id=pmid:9404764&id=doi:10.1378%2Fchest.112.6.1630&issn=0012-3692&volume=112&issue=6&spage=1630&pages=1630-1656&date=1997&title=Chest&atitle=Disease+management+of+COPD+with+pulmonary+rehabilitation&aulast=Tiep)

243.

ATS standards for the optimal management of chronic obstructive pulmonary disease.

Celli B.R.

Embase

Respirology. 2(SUPPL. 1) (pp S1-S4), 1997. Date of Publication: 1997.

[Conference Paper]

AN: 27467270

Tobacco smoking is the main cause of chronic obstructive pulmonary disease (COPD), and the provision of encouragement and support in smoking cessation is the best way to help patients with COPD. The three major goals of COPD management are to lessen airflow limitation, to prevent and treat secondary medical complications and to decrease respiratory symptoms and improve quality of life. Outpatient pharmacotherapy should be organized in a stepwise manner according the severity of disease, the aims being to induce bronchodilation, reduce inflammation and facilitate expectoration, although the role of anti-inflammatory and mucolytic treatment in COPD has not been clearly established. Patients who are not well controlled on optimal pharmacotherapy are candidates for enrolment in a pulmonary rehabilitation programme. Correction or prevention of hypoxaemia is a priority, and long- term oxygen therapy may prolong survival in hypoxaemic patients. With only limited data on criteria for hospital admission and the objectives of hospitalization, the published ATS standards on the management of COPD include expert consensus statements on these aspects of hospital care. Special considerations, such as surgery in the COPD patient, sleep disorders, nutrition, air travel, and ethical issues, are discussed.

PMID

9400687 [<https://www.ncbi.nlm.nih.gov/pubmed/?term=9400687>]

Status

Embase

Institution

(Celli) Pulmonary and Critical Care Division, St. Elizabeth's Medical Center, 736 Cambridge Street, Boston, MA 02135-2997, United States

Publisher

Blackwell Publishing (550 Swanston Street, Carlton South VIC 3053, Australia)

Year of Publication

1997

Link to the Ovid Full Text or citation:

[Click here for full text options](https://ovidsp.ovid.com/ovidweb.cgi?T=JS&CSC=Y&NEWS=N&PAGE=fulltext&D=emed6&AN=27467270)

Link to the External Link Resolver:

[LibKey NHS](https://libkey.io/libraries/2789/openurl?genre=article&sid=OVID:emed6&genre=article&id=pmid:9400687&id=doi:&issn=1323-7799&volume=2&issue=SUPPL.+1&spage=S1&pages=S1-S4&date=1997&title=Respirology&atitle=ATS+standards+for+the+optimal+management+of+chronic+obstructive+pulmonary+disease&aulast=Celli)

244.

ATS standards for the diagnosis and care of patients with chronic obstructive pulmonary disease.

Celli B.R.

Embase

European Respiratory Review. 6(39) (pp 276-281), 1996. Date of Publication: 1996.

[Conference Paper]

AN: 27060922

The American Thoracic Society (ATS) standards for the diagnosis and care of patients with chronic obstructive pulmonary disease (COPD) were published in November 1995. The standards aim to assist chest specialists in the management of patients and include: basic definitions and concepts; ethical issues; management both in out-patient and in-patient settings; possible hazards and special considerations during surgery; sleep; nutrition; and air travel in patients with COPD. The document stresses the importance of therapeutic interventions to help slow the decline of lung function and to treat symptoms. It provides a comprehensive guide to smoking cessation, pharmacological therapy, oxygen therapy, pulmonary rehabilitation and ventilation, all of which may play a role in the optimal management of patients with COPD.

Status

Embase

Institution

(Celli) St Elizabeth's Medical Center Boston, 736 Cambridge Street, Boston MA, United States

Publisher

European Respiratory Society (4 Ave Sainte-Luce, Lausanne CH-1003, Switzerland)

Year of Publication

1996

Link to the Ovid Full Text or citation:

[Click here for full text options](https://ovidsp.ovid.com/ovidweb.cgi?T=JS&CSC=Y&NEWS=N&PAGE=fulltext&D=emed6&AN=27060922)

Link to the External Link Resolver:

[LibKey NHS](https://libkey.io/libraries/2789/openurl?genre=article&sid=OVID:emed6&genre=article&id=pmid:&id=doi:&issn=0905-9180&volume=6&issue=39&spage=276&pages=276-281&date=1996&title=European+Respiratory+Review&atitle=ATS+standards+for+the+diagnosis+and+care+of+patients+with+chronic+obstructive+pulmonary+disease&aulast=Celli)

245.

Portable oxygen therapy with oxygen conserving devices and methodologies.

Tiep B.

Embase

Monaldi Archives for Chest Disease. 50(1) (pp 51-57), 1995. Date of Publication: 1995.

[Conference Paper]

AN: 25118528

Oxygen therapy for the end-stage COPD patient should include a portable component. Accordingly, any system should be small and lightweight in order to enable the chronically debilitated patient to be as active and mobile as possible. Standard continuous flow oxygen via nasal cannulae is reliable and effective but it is also very wasteful. As a result, oxygen conserving methodologies have emerged to improve the efficiency of oxygen delivery and have served to lighten the load for these patients. These devices include: reservoir cannulas, transtracheal catheters and demand pulsing delivery devices. They provide oxygen savings from 2:1 to 7:1 over continuous flow delivery. In addition to rendering the newer oxygen systems more portable, they can also reduce the cost of oxygen and its associated service. As patient requirements are highly individual, the wide choice of available methodologies permits the clinician to prescribe the system that best suits the needs of the patient.

PMID

7742825 [<https://www.ncbi.nlm.nih.gov/pubmed/?term=7742825>]

Status

Embase

Institution

(Tiep) Pulmonary Rehabilitation Programme, Casa Colina Hospital, 255 E Bonita Ave, Pomona, CA 91767, United States

Publisher

PI-ME Tipografia Editrice S.r.l. (E-mail: emanuela.fusinato@pagepress.org)

Year of Publication

1995

Link to the Ovid Full Text or citation:

[Click here for full text options](https://ovidsp.ovid.com/ovidweb.cgi?T=JS&CSC=Y&NEWS=N&PAGE=fulltext&D=emed5&AN=25118528)

Link to the External Link Resolver:

[LibKey NHS](https://libkey.io/libraries/2789/openurl?genre=article&sid=OVID:emed5&genre=article&id=pmid:7742825&id=doi:&issn=1122-0643&volume=50&issue=1&spage=51&pages=51-57&date=1995&title=Monaldi+Archives+for+Chest+Disease&atitle=Portable+oxygen+therapy+with+oxygen+conserving+devices+and+methodologies&aulast=Tiep)

246.

Long-term home oxygen therapy.

Tiep B.L.

Embase

Clinics in Chest Medicine. 11(3) (pp 505-521), 1990. Date of Publication: 1990.

[Review]

AN: 20257786

Oxygen therapy improves mortality and morbidity in hypoxemic patients with COPD. For best results, oxygen should be administered continuously. The general guidelines that qualify a patient for long-term home oxygen therapy are rather explicit, although there are some gray areas, such as increasing exercise performance in normoxemic COPD patients and the treatment of dyspnea in certain patients. The physiologic goals of oxygen therapy are to reverse or prevent tissue hypoxia under the various conditions of life. Patients often experience a fall in oxygen saturation during REM sleep and during exercise. Thus, the oxygen prescription should specify the oxygen setting during wakeful rest, sleep, and exertion. Pulmonary rehabilitation is the definitive long-term management for patients with chronic lung diseases by building endurance, strength, and coping skills. Patients undergoing pulmonary rehabilitation are taught to become active and mobile. The rehabilitation programs have a special role in helping patients to accept and properly administer their own oxygen therapy. Oxygen therapy should be based on mobility and portability. Improvements in the weight and bulk of oxygen containers and the introduction of oxygen-conserving devices have rendered oxygen systems more portable and less costly. The prescription initiates a clinical trial relationship between the patient, physician, and home oxygen vendor. Prescribing home oxygen is now more time-consuming, and the prescription process requires the careful attention of the physician. The prescription must include patient data, the diagnostic reasons for oxygen, the blood gases, the type of system, and the literflow for the various living conditions such as rest, sleep, and exertion. Reimbursement for oxygen via Medicare and other carriers will depend upon the physiologic data as well as the successful completion of a rather complex form. Oxygen therapy should be monitored by regular arterial blood gases or pulse oximetry where appropriate. Patients should have a blood gas sample taken upon any change of condition or adjustment of the oxygen flow setting. Successful oxygen therapy should be physician directed, based on a solid understanding of the physiologic processes involved.

PMID

2205441 [<https://www.ncbi.nlm.nih.gov/pubmed/?term=2205441>]

Status

Embase

Institution

(Tiep) Casa Colina Hosp. Rehab. Med., 255 East Bonita Avenue, Pomona, CA 91767, United States

Publisher

W.B. Saunders (Independence Square West, Philadelphia PA 19106-3399, United States)

Year of Publication

1990

Link to the Ovid Full Text or citation:

[Click here for full text options](https://ovidsp.ovid.com/ovidweb.cgi?T=JS&CSC=Y&NEWS=N&PAGE=fulltext&D=emed4&AN=20257786)

Link to the External Link Resolver:

[LibKey NHS](https://libkey.io/libraries/2789/openurl?genre=article&sid=OVID:emed4&genre=article&id=pmid:2205441&id=doi:&issn=0272-5231&volume=11&issue=3&spage=505&pages=505-521&date=1990&title=Clinics+in+Chest+Medicine&atitle=Long-term+home+oxygen+therapy&aulast=Tiep)

247.

Exercise in the pulmonary patient.

Hodgkin J.E.

Embase

Respiratory Care. 27(6) (pp 671-673), 1982. Date of Publication: 1982.

[Article]

AN: 13213331

Exercise conditioning is one of the therapeutic modalities in a comprehensive pulmonary rehabilitation program that has sound objective scientific data to support its efficacy. Reported clinical benefits of a regular exercise program for patients with chronic obstructive pulmonary disease (COPD) include decreased fear of exercise, increased tolerance of dyspnea, better appetite, improved sleep, increased exercise tolerance, and an enhancement of the patient's ability to handle activities of daily living. Unfortunately, many patients with respiratory dysfunction are never encouraged to exercise and, in fact, may be warned against the 'dangers of dyspnea'.

Status

Embase

Institution

(Hodgkin) Dep. Med., Loma Linda Univ. Sch. Med., Loma Linda, CA United States

Year of Publication

1982

Link to the Ovid Full Text or citation:

[Click here for full text options](https://ovidsp.ovid.com/ovidweb.cgi?T=JS&CSC=Y&NEWS=N&PAGE=fulltext&D=emed3&AN=13213331)

Link to the External Link Resolver:

[LibKey NHS](https://libkey.io/libraries/2789/openurl?genre=article&sid=OVID:emed3&genre=article&id=pmid:&id=doi:&issn=0020-1324&volume=27&issue=6&spage=671&pages=671-673&date=1982&title=Respiratory+Care&atitle=Exercise+in+the+pulmonary+patient&aulast=Hodgkin)

248.

Eccentric Training in Pulmonary Rehabilitation of Post-COVID-19 Patients: An Alternative for Improving the Functional Capacity, Inflammation, and Oxidative Stress. [Review]

Contreras-Briceno F, Espinosa-Ramirez M, Rozenberg D, Reid WD

Ovid MEDLINE(R) ALL

Biology. 11(10), 2022 Oct 01.

[Journal Article. Review]

UI: 36290350

The purpose of this narrative review is to highlight the oxidative stress induced in COVID-19 patients (SARS-CoV-2 infection), describe longstanding functional impairments, and provide the pathophysiologic rationale that supports aerobic eccentric (ECC) exercise as a novel alternative to conventional concentric (CONC) exercise for post-COVID-19 patients. Patients who recovered from moderate-to-severe COVID-19 respiratory distress demonstrate long-term functional impairment. During the acute phase, SARS-CoV-2 induces the generation of reactive oxygen species that can be amplified to a "cytokine storm". The resultant inflammatory and oxidative stress process causes organ damage, particularly in the respiratory system, with the lungs as the tissues most susceptible to injury. The acute illness often requires a long-term hospital stay and consequent sarcopenia. Upon discharge, muscle weakness compounded by limited lung and cardiac function is often accompanied by dyspnea, myalgia, anxiety, depression, and sleep disturbance. Consequently, these patients could benefit from pulmonary rehabilitation (PR), with exercise as a critical intervention (including sessions of strength and endurance or aerobic exercises). Unfortunately, conventional CONC exercises induce significant cardiopulmonary stress and increase inflammatory and oxidative stress (OS) when performed at moderate/high intensity, which can exacerbate debilitating dyspnoea and muscle fatigue post-COVID-19. Eccentric training (ECC) is a well-tolerated alternative that improves muscle mass while mitigating cardiopulmonary stress in patients with COPD and other chronic diseases. Similar benefits could be realized in post-COVID-19 patients. Consequently, these patients could benefit from PR with exercise as a critical intervention.

Version ID

1

Status

PubMed-not-MEDLINE

Author Initials

Contreras-Briceno, Felipe; ORCID: <https://orcid.org/0000-0002-0674-7506> Espinosa-Ramirez, Maximiliano; ORCID: <https://orcid.org/0000-0002-0840-1904>

Rozenberg, Dmitry; ORCID: <https://orcid.org/0000-0001-8786-9152>

Reid, W Darlene; ORCID: <https://orcid.org/0000-0001-9980-8699>

Authors Full Name

Contreras-Briceno, Felipe, Espinosa-Ramirez, Maximiliano, Rozenberg, Dmitry, Reid, W Darlene

Institution

Contreras-Briceno, Felipe. Laboratory of Exercise Physiology, Department of Health Science, Faculty of Medicine, Pontificia Universidad Catolica de Chile, Av. Vicuna Mackenna #4860, Santiago 7820436, Chile. Contreras-Briceno, Felipe. Physiology Section, Department of Cell Biology, Physiology and Immunology, Faculty of Biology, Universitat de Barcelona, 08028 Barcelona, Spain.

Contreras-Briceno, Felipe. Advanced Center for Chronic Diseases (ACCDiS), Division of Cardiovascular Diseases, Facultad de Medicina, Pontificia Universidad Catolica de Chile, Marcoleta #367, Santiago 8380000, Chile.

Contreras-Briceno, Felipe. Millennium Institute for Intelligent Healthcare Engineering, Av. Vicuna Mackenna #4860, Santiago 7820436, Chile.

Espinosa-Ramirez, Maximiliano. Laboratory of Exercise Physiology, Department of Health Science, Faculty of Medicine, Pontificia Universidad Catolica de Chile, Av. Vicuna Mackenna #4860, Santiago 7820436, Chile.

Rozenberg, Dmitry. Department of Medicine, Respirology, University of Toronto, Toronto, ON M5G 2C4, Canada.

Rozenberg, Dmitry. Toronto General Hospital, Research Institute, University Health Network, Toronto, ON M5G 2C4, Canada.

Reid, W Darlene. Department of Physical Therapy and Interdepartmental Division of Critical Care Medicine, University of Toronto, Toronto, ON M5G 2C4, Canada.

Reid, W Darlene. KITE Research Institute, Toronto Rehabilitation Institute, University Health Network, Toronto, ON M5G 2A2, Canada.

PMID

<https://www.ncbi.nlm.nih.gov/pmc/articles/PMC9598133>

Year of Publication

2022

Link to the Ovid Full Text or citation:

[Click here for full text options](https://ovidsp.ovid.com/ovidweb.cgi?T=JS&CSC=Y&NEWS=N&PAGE=fulltext&D=pmnm7&AN=36290350)

Link to the External Link Resolver:

[LibKey NHS](https://libkey.io/libraries/2789/openurl?genre=article&sid=OVID:pmnm7&genre=article&id=pmid:36290350&id=doi:10.3390%2Fbiology11101446&issn=2079-7737&volume=11&issue=10&spage=&pages=&date=2022&title=Biology&atitle=Eccentric+Training+in+Pulmonary+Rehabilitation+of+Post-COVID-19+Patients%3A+An+Alternative+for+Improving+the+Functional+Capacity%2C+Inflammation%2C+and+Oxidative+Stress.&aulast=Contreras-Briceno)

249.

Effects of Altitude on Chronic Obstructive Pulmonary Disease Patients: Risks and Care. [Review]

Georges T, Le Blanc C, Ferreol S, Menu P, Dauty M, Fouasson-Chailloux A

Ovid MEDLINE(R) ALL

Life. 11(8), 2021 Aug 07.

[Journal Article. Review]

UI: 34440542

Air travel and altitude stays have become increasingly frequent within the overall population but also in patients suffering from chronic obstructive pulmonary disease (COPD), which is the most common respiratory disease worldwide. While altitude is well tolerated by most individuals, COPD patients are exposed to some serious complications, that could be life-threatening. COPD patients present not only a respiratory illness but also frequent comorbidities. Beyond oxygen desaturation, it also affects respiratory mechanics, and those patients are at high risk to decompensate a cardiac condition, pulmonary hypertension, or a sleep disorder. Recently, there has been considerable progress in the management of this disease. Nocturnal oxygen therapy, inhaled medications, corticosteroids, inspiratory muscle training, and pulmonary rehabilitation are practical tools that must be developed in the comprehensive care of those patients so as to enable them to afford altitude stays.

Version ID

1

Status

PubMed-not-MEDLINE

Author Initials

Le Blanc, Camille; ORCID: <https://orcid.org/0000-0003-0806-3770> Fouasson-Chailloux, Alban; ORCID: <https://orcid.org/0000-0002-8139-814X>

Authors Full Name

Georges, Thomas, Le Blanc, Camille, Ferreol, Sophie, Menu, Pierre, Dauty, Marc, Fouasson-Chailloux, Alban

Institution

Georges, Thomas. CHU Nantes, Service de Medecine Physique et Readapatation Locomotrice et Respiratoire, 44093 Nantes, France. Le Blanc, Camille. CHU Nantes, Service de Medecine Physique et Readapatation Locomotrice et Respiratoire, 44093 Nantes, France.

Ferreol, Sophie. CHU Nantes, Service de Medecine Physique et Readapatation Locomotrice et Respiratoire, 44093 Nantes, France.

Menu, Pierre. CHU Nantes, Service de Medecine Physique et Readapatation Locomotrice et Respiratoire, 44093 Nantes, France.

Menu, Pierre. CHU Nantes, Service de Medecine du Sport, 44093 Nantes, France.

Menu, Pierre. IRMS, Institut Regional de Medecine du Sport, 44093 Nantes, France.

Menu, Pierre. Inserm, UMR 1229, RMeS, Regenerative Medicine and Skeleton, Universite de Nantes, ONIRIS, 44042 Nantes, France.

Dauty, Marc. CHU Nantes, Service de Medecine Physique et Readapatation Locomotrice et Respiratoire, 44093 Nantes, France.

Dauty, Marc. CHU Nantes, Service de Medecine du Sport, 44093 Nantes, France.

Dauty, Marc. IRMS, Institut Regional de Medecine du Sport, 44093 Nantes, France.

Dauty, Marc. Inserm, UMR 1229, RMeS, Regenerative Medicine and Skeleton, Universite de Nantes, ONIRIS, 44042 Nantes, France.

Fouasson-Chailloux, Alban. CHU Nantes, Service de Medecine Physique et Readapatation Locomotrice et Respiratoire, 44093 Nantes, France.

Fouasson-Chailloux, Alban. CHU Nantes, Service de Medecine du Sport, 44093 Nantes, France.

Fouasson-Chailloux, Alban. IRMS, Institut Regional de Medecine du Sport, 44093 Nantes, France.

Fouasson-Chailloux, Alban. Inserm, UMR 1229, RMeS, Regenerative Medicine and Skeleton, Universite de Nantes, ONIRIS, 44042 Nantes, France.

PMID

<https://www.ncbi.nlm.nih.gov/pmc/articles/PMC8401125>

Year of Publication

2021

Link to the Ovid Full Text or citation:

[Click here for full text options](https://ovidsp.ovid.com/ovidweb.cgi?T=JS&CSC=Y&NEWS=N&PAGE=fulltext&D=pmnm6&AN=34440542)

Link to the External Link Resolver:

[LibKey NHS](https://libkey.io/libraries/2789/openurl?genre=article&sid=OVID:pmnm6&genre=article&id=pmid:34440542&id=doi:10.3390%2Flife11080798&issn=2075-1729&volume=11&issue=8&spage=&pages=&date=2021&title=Life&atitle=Effects+of+Altitude+on+Chronic+Obstructive+Pulmonary+Disease+Patients%3A+Risks+and+Care.&aulast=Georges)

250.

[Care-Related intervention in Rehabilitative Pneumology: Pulmonary Rehabilitation in Chronic Obstructive Broncopneumopathies (COPD) can benefit from a multidisciplinary approach?]. [Italian] Cure correlate in Pneumologia Riabilitativa: la Riabilitazione Polmonare nelle Broncopneumopatie Croniche Ostruttive (BPCO) puo trarre beneficio da un approccio multidisciplinare? <Cure correlate in Pneumologia Riabilitativa: la Riabilitazione Polmonare nelle Broncopneumopatie Croniche Ostruttive (BPCO) puo trarre beneficio da un approccio multidisciplinare?>

Vitacca M, Visca D, Spanevello A

Ovid MEDLINE(R) ALL

Giornale Italiano di Medicina del Lavoro Ed Ergonomia. 40(1):37-41, 2018 02.

[Journal Article]

UI: 29916565

OBJECTIVES: Chronic Obstructive Pulmonary Disease (COPD) is a common, preventable and treatable disease that is characterized by persistent respiratory symptoms and airflow limitation. Cardiovascular diseases, osteoporosis, depression/anxiety, musculoskeletal impairment and obstructive sleep apnea are frequent and important comorbidities in COPD, often under-diagnosed, and associated with poor health status and prognosis.

METHODS: Pulmonary rehabilitation improves symptoms, quality of life, pulmonary function, and health care in patients with chronic respiratory disease. By definition it is a comprehensive intervention based on a thorough patient assessment followed by patient-tailored therapies that include, but are not limited to, exercise training, education, and behavior change and designed to improve the physical and psychological condition of people with chronic respiratory disease and to promote the long-term adherence to health-enhancing behaviors. Exercise limitation in patients with COPD is multifactorial and includes ventilatory limitation, gas transfer abnormalities, pulmonary vascular and cardiac dysfunction, limb muscle dysfunction, and comorbid impairments.

RESULTS: Overall, pulmonary rehabilitation aims to improve cardiorespiratory and skeletal muscle function improving respiratory symptoms and quality of life in daily life activities adding a synergic support to the pharmacologic inhaled therapy. COPD has a variable natural history, but most of the time chronic respiratory failure complicates disease progression. Supplemental oxygen and noninvasive mechanical ventilation have been proven to improve survival and reduce hospital admissions in COPD patients. Furthermore additional studies have shown that exercise performance benefit from supplemental oxygen and NIV used both during rehabilitation exercise programs and over the night.

CONCLUSIONS: In conclusion, an overarching approach to diagnosis, assessment of severity of COPD and its frequent comorbidities should guide to a multidisciplinary and synergic approach in terms of pharmacological and nonpharmacological management of a systemic inflammatory syndrome.

Copyright© by Aracne Editrice, Roma, Italy.

Version ID

1

Status

MEDLINE

Authors Full Name

Vitacca, Michele, Visca, Dina, Spanevello, Antonio

Institution

Vitacca, Michele. Pneumologia Riabilitativa ICS Maugeri IRCCS Lumezzane (BS). Visca, Dina. Pneumologia Riabilitativa ICS Maugeri IRCCS Tradate (VA), Universita degli Studi dell'Insubria Varese Como.

Spanevello, Antonio. Pneumologia Riabilitativa ICS Maugeri IRCCS Tradate (VA), Universita degli Studi dell'Insubria Varese Como.

Collaborator Alias

Publisher

La Broncopneumopatia Cronica Ostruttiva (BPCO) e una comune malattia, prevenibile e trattabile, caratterizzata da persistenti sintomi respiratori e limitazione al flusso aereo. Patologie cardiovascolari, osteoporosi, depressione e ansia, condizionamento neuromuscolare e apnee ostruttive del sonno sono comorbilita frequenti e importanti nella BPCO, spesso sotto diagnosticate e associate a peggiore stato di salute e prognosi. La riabilitazione respiratoria migliora i sintomi, la qualita della vita, la funzione polmonare e lo stato di salute in pazienti con patologie croniche ostruttive. Per definizione e un intervento ad ampio spettro che si basa su una valutazione del paziente seguita da terapie personalizzate, essa include inoltre riallenamento allo sforzo, intervento educazionale e modifica dello stile di vita volto al miglioramento della condizione fisica e psicologica di persone affette da patologie croniche respiratorie e al miglioramento della aderenza alla terapia e allo stile di vita. La limitazione all'esercizio fisico in pazienti con BPCO e multifattoriale, e dovuta a una limitazione ventilatoria, alterazione degli scambi intrapolmonari dei gas, alterazione vascolare polmonare e cardiaca, disfunzione muscolare e a presenza di comorbilita. La riabilitazione pneumologica ha come obiettivo il miglioramento della funzione cardiorespiratoria e muscolare, miglioramento dei sintomi e della qualita di vita nelle attivita quotidiane, agendo in sinergia con l'effetto della terapia inalatoria. La BPCO ha una storia naturale variabile, spesso l'insufficienza respiratoria cronica complica le fasi di progressione della malattia. E stato dimostrato che il supplemento di ossigeno e la ventilazione meccanica non invasiva migliorano la sopravvivenza e riducono il rischio di ricoveri ospedalieri in pazienti affetti da BPCO. Studi successivi hanno poi evidenziato il ruolo benefico di utilizzare supplemento di ossigeno e NIV durante sia i programmi di fisioterapia respiratoria sia durante le ore notturne. In conclusione, un approccio ad ampio spettro per diagnosi e stadiazione della BPCO anche alla luce delle comorbidita spesso presenti potrebbe convogliare verso un approccio multidisciplinare e sinergico sia in termini di trattamento farmacologico che non farmacologico di una sindrome infiammatoria sistemica.

Language: Italian

Year of Publication

2018

Link to the Ovid Full Text or citation:

[Click here for full text options](https://ovidsp.ovid.com/ovidweb.cgi?T=JS&CSC=Y&NEWS=N&PAGE=fulltext&D=med15&AN=29916565)

Link to the External Link Resolver:

[LibKey NHS](https://libkey.io/libraries/2789/openurl?genre=article&sid=OVID:med15&genre=article&id=pmid:29916565&id=doi:&issn=1592-7830&volume=40&issue=1&spage=37&pages=37-41&date=2018&title=Giornale+Italiano+di+Medicina+del+Lavoro+Ed+Ergonomia&atitle=Cure+correlate+in+Pneumologia+Riabilitativa%3A+la+Riabilitazione+Polmonare+nelle+Broncopneumopatie+Croniche+Ostruttive+(BPCO)+puo+trarre+beneficio+da+un+approccio+multidisciplinare%3F&aulast=Vitacca)

251.

Comorbidities, Complications and Non-Pharmacologic Treatment in Idiopathic Pulmonary Fibrosis. [Review]

Millan-Billi P, Serra C, Alonso Leon A, Castillo D

Ovid MEDLINE(R) ALL

Medical Sciences. 6(3), 2018 Jul 24.

[Journal Article. Review]

UI: 30042369

Idiopathic pulmonary fibrosis (IPF) is a chronic, progressive and fatal disease. The treatment is challenging and nowadays a comprehensive approach based not only in pharmacological strategies is necessary. Identification and control of comorbidities, non-pharmacological treatment, prevention and management of exacerbations as well as other areas of care (social, psychological) are fundamental for a holistic management of IPF. Gastroesophageal reflux, pulmonary hypertension, obstructive sleep apnea, combined with emphysema, lung cancer and cardiovascular involvement are the main comorbidities associated with IPF. Non-pharmacological treatment includes the use of oxygen in patients with rest or nocturnal hypoxemia and other support therapies such as non-invasive ventilation or even a high-flow nasal cannula to improve dyspnea. In some patients, lung transplant should be considered as this enhances survival. Pulmonary rehabilitation can add benefits in outcomes such control of dyspnea, exercise capacity distance and, overall, improve the quality of life; therefore it should be considered in patients with IPF. Also, multidisciplinary palliative care programs could help with symptom control and psychological support, with the aim of maintaining quality of life during the whole process of the disease. This review intends to provide clear information to help those involved in IPF follow up to improve patients' daily care.

Version ID

1

Status

PubMed-not-MEDLINE

Authors Full Name

Millan-Billi, Paloma, Serra, Candela, Alonso Leon, Ana, Castillo, Diego

Institution

Millan-Billi, Paloma. Department of Respiratory Medicine, Hospital de la Santa Creu I Sant Pau, C/Sant Antoni M. Claret, 167, 08025 Barcelona, Spain. PMillan@santpau.cat. Serra, Candela. Department of Respiratory Medicine, Hospital de la Santa Creu I Sant Pau, C/Sant Antoni M. Claret, 167, 08025 Barcelona, Spain. CSerraX@santpau.cat.

Alonso Leon, Ana. Department of Respiratory Medicine, Hospital de la Santa Creu I Sant Pau, C/Sant Antoni M. Claret, 167, 08025 Barcelona, Spain. AAlonso@santpau.cat.

Castillo, Diego. Department of Respiratory Medicine, Hospital de la Santa Creu I Sant Pau, C/Sant Antoni M. Claret, 167, 08025 Barcelona, Spain. dcastillo@santpau.cat.

PMID

<https://www.ncbi.nlm.nih.gov/pmc/articles/PMC6164236>

Year of Publication

2018

Link to the Ovid Full Text or citation:

[Click here for full text options](https://ovidsp.ovid.com/ovidweb.cgi?T=JS&CSC=Y&NEWS=N&PAGE=fulltext&D=pmnm4&AN=30042369)

Link to the External Link Resolver:

[LibKey NHS](https://libkey.io/libraries/2789/openurl?genre=article&sid=OVID:pmnm4&genre=article&id=pmid:30042369&id=doi:10.3390%2Fmedsci6030059&issn=2076-3271&volume=6&issue=3&spage=&pages=&date=2018&title=Medical+Sciences&atitle=Comorbidities%2C+Complications+and+Non-Pharmacologic+Treatment+in+Idiopathic+Pulmonary+Fibrosis.&aulast=Millan-Billi)

252.

[Sleep disorders in asthma and chronic obstructive pulmonary disease (COPD)]. [Review] [German] Bedeutung von Schlafstorungen, deren Abklarung und Behandlung bei Asthma und COPD. <Bedeutung von Schlafstorungen, deren Abklarung und Behandlung bei Asthma und COPD.>

Boing S, Randerath WJ

Ovid MEDLINE(R) ALL

Therapeutische Umschau. 71(5):301-8, 2014 May.

[English Abstract. Journal Article. Review]

UI: 24794341

Sleep disturbances (SD) are a frequent finding in patients with asthma and chronic obstructive pulmonary disease (COPD) and have a negative impact on quality of life and the clinical course of the disease. The causes of SD are multiple and include for example respiratory symptoms and comorbidities. On the other hand sleep goes along with multiple physiological changes in respiration, so that sleep itself interacts with asthma and COPD. This interaction favors respiratory symptoms and may lead to hypoxemia and hypercapnia. A further complication of the respiratory situation and the clinical course can be found in asthma and COPD patients with coexisting obstructive sleep apnea syndrome (OSAS). Due to the heterogeneity of SD in asthma and COPD, a detailed patient survey is the most important diagnostical tool. Based on the survey further technical examinations should be considered. Treatment strategies for the reduction of SD in asthma and COPD include an optimized medication and treatment of comorbidities. If indicated oxygen therapy, positive pressure breathing and pulmonary rehabilitation can contribute.

Version ID

1

Status

MEDLINE

Authors Full Name

Boing, Sebastian, Randerath, Winfried J

Institution

Boing, Sebastian. Institut fur Pneumologie an der Universitat Witten Herdecke. Randerath, Winfried J. Klinik fur Pneumologie und Allergologie, Schlaf- und Beatmungsmedizin, Bethanien Krankenhaus, Solingen.

Collaborator Alias

Publisher

Schlafstorungen stellen bei Patienten mit Asthma bronchiale und chronisch obstruktiver Lungenerkrankung (COPD) ein haufiges Problem dar, das sich negativ auf Lebensqualitat und Krankheitsverlauf auswirken kann. Als Ursache der Schlafstorungen kommen multiple Faktoren wie beispielweise respiratorische Symptome und Komorbiditaten in Betracht. Gleichzeitig lasst auch Schlaf selber eine Interaktion mit beiden obstruktiven Atemwegserkrankungen erkennen. Sie begunstigt respiratorische Symptome und kann bei fortgeschrittener Erkrankung in schlafbezogenen Hypoxamien und Hyperkapnien resultieren. Das obstruktive Schlafapnoe-Syndrom ist eine haufige und wichtige Begleiterkrankung bei Asthma und COPD. Es fuhrt zu vermehrten respiratorischen Symptomen bei beiden Erkrankungen und ging in Studien bei der COPD mit einer gesteigerten Mortalitat einher. Aufgrund der Heterogenitat von Schlafstorungen bei Asthma und COPD stellt die Anamnese das wichtigste Instrument zur Abklarung dar und ist gleichzeitig Weichensteller fur eine weitere apparative Diagnostik. Therapeutische Ziele zur Reduktion von Schlafstorungen sind die optimale medikamentose Einstellung und die Behandlung relevanter Komorbiditaten. Weitere Therapiesaulen sind bei entsprechender Indikation die Verfahren der Positivdruckatmung, die Sauerstofftherapie und die pneumologische Rehabilitation.

Language: German

Year of Publication

2014

Link to the Ovid Full Text or citation:

[Click here for full text options](https://ovidsp.ovid.com/ovidweb.cgi?T=JS&CSC=Y&NEWS=N&PAGE=fulltext&D=med11&AN=24794341)

Link to the External Link Resolver:

[LibKey NHS](https://libkey.io/libraries/2789/openurl?genre=article&sid=OVID:med11&genre=article&id=pmid:24794341&id=doi:10.1024%2F0040-5930%2Fa000517&issn=0040-5930&volume=71&issue=5&spage=301&pages=301-8&date=2014&title=Therapeutische+Umschau&atitle=Bedeutung+von+Schlafstorungen%2C+deren+Abklarung+und+Behandlung+bei+Asthma+und+COPD.&aulast=Boing)

253.

Perioperative Evaluation of Patients with Pulmonary Conditions Undergoing Non-Cardiothoracic Surgery. [Review]

Diaz-Fuentes G, Hashmi HR, Venkatram S

Ovid MEDLINE(R) ALL

Health Services Insights. 9(Suppl 1):9-23, 2016.

[Review. Journal Article]

UI: 27867301

This review describes the perioperative management of patients with suspected or established pulmonary conditions undergoing non-cardiothoracic surgery, with a focus on common pulmonary conditions such as obstructive airway disease, pulmonary hypertension, obstructive sleep apnea, and chronic hypoxic respiratory conditions. Considering that postoperative pulmonary complications are common and given the increasing number of surgical procedures and the size of the aging population, familiarity with current guidelines for preoperative risk assessment and intra- and postoperative patient management is recommended to decrease the morbidity and mortality. In particular, smoking cessation and pulmonary rehabilitation are perioperative strategies for improving patients' short- and long-term outcomes. Understanding the potential risk for pulmonary complications allows the medical team to appropriately plan the intra- and postoperative care of each patient.

Version ID

1

Status

PubMed-not-MEDLINE

Authors Full Name

Diaz-Fuentes, Gilda, Hashmi, Hafiz Rizwan Talib, Venkatram, Sindhaghatta

Institution

Diaz-Fuentes, Gilda. Chief, Division of Pulmonary and Critical Care Medicine, Bronx Lebanon Hospital Center, Bronx, NY, USA.; Associate Professor. Hashmi, Hafiz Rizwan Talib. Fellow, Division of Pulmonary and Critical Care Medicine, Bronx Lebanon Hospital Center, Bronx, NY, USA.

Venkatram, Sindhaghatta. Assistant Professor, Clinical Medicine, Icahn School of Medicine at Mount Sinai, New York, NY, USA.; Attending, Division of Pulmonary and Critical Care Medicine, Bronx Lebanon Hospital Center, Bronx, NY, USA.

PMID

<https://www.ncbi.nlm.nih.gov/pmc/articles/PMC5104294>

Year of Publication

2016

Link to the Ovid Full Text or citation:

[Click here for full text options](https://ovidsp.ovid.com/ovidweb.cgi?T=JS&CSC=Y&NEWS=N&PAGE=fulltext&D=pmnm3&AN=27867301)

Link to the External Link Resolver:

[LibKey NHS](https://libkey.io/libraries/2789/openurl?genre=article&sid=OVID:pmnm3&genre=article&id=pmid:27867301&id=doi:10.4137%2FHSI.S40541&issn=1178-6329&volume=9&issue=1&spage=9&pages=9-23&date=2016&title=Health+Services+Insights&atitle=Perioperative+Evaluation+of+Patients+with+Pulmonary+Conditions+Undergoing+Non-Cardiothoracic+Surgery.&aulast=Diaz-Fuentes)

254.

Determinants of success. [Review]

Garrod R, Malerba M, Crisafulli E

Ovid MEDLINE(R) ALL

European Respiratory Journal. 38(5):1215-8, 2011 Nov.

[Journal Article. Review]

UI: 22045787

In chronic obstructive pulmonary disease (COPD) patients, pulmonary rehabilitation is a nonpharmacological intervention aimed at improving physical exercise tolerance, dyspnoea and perceived quality of life. However, identifying predictors of clinical response and which patients achieve benefit remains a difficult question to answer with no conclusive data available. Baseline characteristics of COPD patients, such as degree of breathlessness, body weight and arterial partial pressure of oxygen, generally appear to be too direct to have a correlation with improvement of post-rehabilitation outcomes. Furthermore, some additional benefits of patients treated with rehabilitation are simply not detected by usual measures (social interaction, sleep quality and confidence). Although there are some data suggesting that some medical conditions frequently associated with COPD (osteoporosis, metabolic syndrome and heart diseases) may negatively influence rehabilitation outcomes, at present the evidence is contradictory.

Version ID

1

Status

MEDLINE

Authors Full Name

Garrod, R, Malerba, M, Crisafulli, E

Institution

Garrod, R. Dept of Pulmonary Rehabilitation, King's College Hospital NHS Foundation Trust, London, UK.

Year of Publication

2011

Link to the Ovid Full Text or citation:

[Click here for full text options](https://ovidsp.ovid.com/ovidweb.cgi?T=JS&CSC=Y&NEWS=N&PAGE=fulltext&D=med8&AN=22045787)

Link to the External Link Resolver:

[LibKey NHS](https://libkey.io/libraries/2789/openurl?genre=article&sid=OVID:med8&genre=article&id=pmid:22045787&id=doi:10.1183%2F09031936.00088611&issn=0903-1936&volume=38&issue=5&spage=1215&pages=1215-8&date=2011&title=European+Respiratory+Journal&atitle=Determinants+of+success.&aulast=Garrod)

255.

[Home pulmonary rehabilitation: results in a cohort of 37 patients with respiratory handicap]. [French] Mise en oeuvre et bilan d'un reentrainement a l'effort a domicile chez 37 patients avec handicap respiratoire. <Mise en oeuvre et bilan d'un reentrainement a l'effort a domicile chez 37 patients avec handicap respiratoire.>

Borel JC, Wuyam B, Veale D, Maclet E, Pison C

Ovid MEDLINE(R) ALL

Revue des Maladies Respiratoires. 21(4 Pt 1):711-7, 2004 Sep.

[Clinical Trial. English Abstract. Journal Article. Research Support, Non-U.S. Gov't]

UI: 15536372

INTRODUCTION: The purpose of this open, prospective, non-controlled study is to evaluate the feasibility and effect of 10 weeks of home-based exercise retraining on a cyclo-ergometer in respiratory patients.

METHODS: 75 patients are consecutively referred. 37 patients, (75% COPD), took part in the programme, 38 (51%) did not. Nine dropped out, five had missing data, therefore the analysis is of 23 patients.

RESULTS: There was a significant improvement in Quality of life (CRQ) (p=0,02), in endurance and in work rate on the bicycle (p<0.001). There is a correlation between the increase in work rate on the bicycle and the improvement in quality of life (p=0.01, r=0.5). When the patients are divided according to the level of improvement in work rate on the cycle-ergometer (mild 0 to 10% improvement; moderate 11 to 49% and strong > 50%) we find that only mild improvement is found in those with a low initial work rate.

CONCLUSION: We confirm the effectiveness of home based training, this being feasible in one out of two patients referred. Co-morbidity and the psycho-social situation were the main exclusion factors. The improvement in effort capacity could be an important determinant of quality of life. Cyclo-ergometer could required too great an effort for training more severe patients.

Version ID

1

Status

MEDLINE

Authors Full Name

Borel, J-C, Wuyam, B, Veale, D, Maclet, E, Pison, C

Institution

Borel, J-C. Service d'Explorations Fonctionnelles Cardio-Respiratoire, CHU de Grenoble, France. jc.borel@agiradom.com

Comments

Comment in (CIN)

Year of Publication

2004

Link to the Ovid Full Text or citation:

[Click here for full text options](https://ovidsp.ovid.com/ovidweb.cgi?T=JS&CSC=Y&NEWS=N&PAGE=fulltext&D=med5&AN=15536372)

Link to the External Link Resolver:

[LibKey NHS](https://libkey.io/libraries/2789/openurl?genre=article&sid=OVID:med5&genre=article&id=pmid:15536372&id=doi:10.1016%2Fs0761-8425(04)71412-6&issn=0761-8425&volume=21&issue=4&spage=711&pages=711-7&date=2004&title=Revue+des+Maladies+Respiratoires&atitle=Mise+en+oeuvre+et+bilan+d'un+reentrainement+a+l'effort+a+domicile+chez+37+patients+avec+handicap+respiratoire.&aulast=Borel)

256. (repeat)

[Oxygen saturation at rest, on exercise and during sleep in COPD patients undergoing pulmonary rehabilitation program. Two years prospective controlled study]. [Polish] Kontrolowane badanie przezskorne wysycenia tlenem krwi tetniczej w czasie dnia, w nocy oraz w czasie wysilku u rehabilitowanych chorych na POChP. <Kontrolowane badanie przezskorne wysycenia tlenem krwi tetniczej w czasie dnia, w nocy oraz w czasie wysilku u rehabilitowanych chorych na POChP.>

Piszko P, Lewczuk J, Kowalska-Superlak M, Wrabec K

Ovid MEDLINE(R) ALL

Pneumonologia i Alergologia Polska. 70(11-12):566-72, 2002.

[Clinical Trial. Controlled Clinical Trial. English Abstract. Journal Article]

UI: 12884566

The role of pulmonary rehabilitation (PR) in COPD patients is well established. However, the PR programs includes physical exercise that may deteriorate chronic hypoxemia. The objective of the study was to evaluate impact of a long term PR program on oxygen saturation at rest, on exercise and during sleep in COPD patients no receiving supplemental oxygenation. A two--year controlled study involved 27 rehabilitated (PR-COPD patients (6 females) aged 32-76 years, mean 58.1 years on average and 19 control (C-COPD patients (3 females) aged 58-83 years, mean 68. COPD patients participating in PR program patients were younger, had higher value of FVC and FEV1 and better results of METs during symptom-limited exercise test (ET, p = 0.007). They also had better results of transcutaneus oxygen saturation (SaO2) at rest (p = 0.02), a lower maximal decrease in SaO2 during ET(p = 0.003) and a higher value of medan SaO2 during (p = 0.02). Following PR program the number of METs achieved during ET increased in PR-COPD patients (p = 0.007) and did not change in C-COPD, p = NS. The SaO2 at rest did not change significantly in both groups. Maximal drop in SaO2 during ET decreased in PR-COPD patients (p = 0.02) and did not change in C-COPD(p = NS) group. Median SaO2 at sleep did not change in PR-COPD patients (p = NS) but it decreased in C-COPD patients (p = 0.02). The study indicates that long term PR does not deteriorate the resting diurnal hypoxaemia, it prevent COPD patients from time depending exacerbation of nocturnal hypoxaemia and can improve oxygen saturation on exertion. The results could be influenced by baseline differences between PR-COPD and C-COPD patients.

Version ID

1

Status

MEDLINE

Authors Full Name

Piszko, Piotr, Lewczuk, Jerzy, Kowalska-Superlak, Maria, Wrabec, Krzysztof

Institution

Piszko, Piotr. Oddzialu Kardiologicznego Wojewodzkiego, Szpitala Specjalistycznego we Wroclawiu.

Year of Publication

2002

Link to the Ovid Full Text or citation:

[Click here for full text options](https://ovidsp.ovid.com/ovidweb.cgi?T=JS&CSC=Y&NEWS=N&PAGE=fulltext&D=med4&AN=12884566)

Link to the External Link Resolver:

[LibKey NHS](https://libkey.io/libraries/2789/openurl?genre=article&sid=OVID:med4&genre=article&id=pmid:12884566&id=doi:&issn=0867-7077&volume=70&issue=11-12&spage=566&pages=566-72&date=2002&title=Pneumonologia+i+Alergologia+Polska&atitle=Kontrolowane+badanie+przezskorne+wysycenia+tlenem+krwi+tetniczej+w+czasie+dnia%2C+w+nocy+oraz+w+czasie+wysilku+u+rehabilitowanych+chorych+na+POChP.&aulast=Piszko)

257.

Standards for the optimal management of COPD: a summary. [Review] [22 refs]

Celli BR

Ovid MEDLINE(R) ALL

Chest. 113(4 Suppl):283S-287S, 1998 Apr.

[Journal Article. Review]

UI: 9552019

Tobacco smoking is the main cause of COPD, and encouragement and support in smoking cessation is the best way to help the patient with COPD. The three major goals of COPD management are to lessen airflow limitation, to prevent and treat secondary medical complications, and to decrease respiratory symptoms and improve quality of life. Outpatient pharmacotherapy should be organized in a stepwise manner according the severity of disease, the aims being to induce bronchodilation, reduce inflammation, and facilitate expectoration, although the role of anti-inflammatory and mucolytic treatment of COPD has not been clearly established. Patients whose conditions are not well controlled with optimal pharmacotherapy are candidates for enrollment in a pulmonary rehabilitation program. Correction or prevention of hypoxemia is a priority, and long-term oxygen therapy supplementation prolongs survival in hypoxemic patients. With only limited data on criteria for hospital admission and the objectives of hospitalization, the published standards on the management of COPD include an expert consensus statement on these aspects of hospital care. Surgery, special considerations such as sleep, nutrition, and air travel, and ethical issues are discussed. [References: 22]

Version ID

1

Status

MEDLINE

Authors Full Name

Celli, B R

Institution

Celli, B R. Tufts University School of Medicine, and Pulmonary and Critical Care Division, St. Elizabeth's Medical Center of Boston, MA 02135-2997, USA.

Year of Publication

1998

Link to the Ovid Full Text or citation:

[Click here for full text options](https://ovidsp.ovid.com/ovidweb.cgi?T=JS&CSC=Y&NEWS=N&PAGE=fulltext&D=med4&AN=9552019)

Link to the External Link Resolver:

[LibKey NHS](https://libkey.io/libraries/2789/openurl?genre=article&sid=OVID:med4&genre=article&id=pmid:9552019&id=doi:10.1378%2Fchest.113.4_supplement.283s&issn=0012-3692&volume=113&issue=4&spage=283S&pages=283S-287S&date=1998&title=Chest&atitle=Standards+for+the+optimal+management+of+COPD%3A+a+summary.&aulast=Celli)

258.

Mechanical exsufflation, noninvasive ventilation, and new strategies for pulmonary rehabilitation and sleep disordered breathing. [Review] [57 refs]

Bach JR

Ovid MEDLINE(R) ALL

Bulletin of the New York Academy of Medicine. 68(2):321-40, 1992 Mar-Apr.

[Journal Article. Review]

UI: 1586868

Manual and mechanical exsufflation are important but underutilized ways to clear airway secretions. These methods are especially useful when used in concert with noninvasive intermittent positive airway pressure ventilatory assistance to facilitate extubation and ventilator weaning. This can be used as much as 24 hours a day as an alternative to tracheostomy ventilation or body ventilator use for patients with paralytic restrictive ventilatory insufficiency. These techniques expedite community management of ventilator assisted individuals by avoiding tracheostomy and need for invasive suctioning and ongoing wound care. For these techniques to be effective and to prevent further suppression of ventilatory drive, supplemental oxygen administration must be avoided unless pO2 is less than 60 mm Hg despite normalization of pCO2. Custom molded interfaces for the delivery of noninvasive intermittent positive airway pressure ventilatory assistance can also be used to facilitate the delivery of variable inspiratory expiratory positive airway pressure for patients with obstructive sleep apnea. Noninvasive intermittent positive airway pressure ventilatory assistance or body ventilator use can rest the respiratory muscles of patients with advanced chronic obstructive pulmonary disease. This and pulmonary rehabilitation programs geared to exercise reconditioning are therapeutic options that significantly improve the quality of life of these patients. For both paralytic restrictive and obstructive pulmonary patients, these techniques decrease cost and frequency of hospitalizations. [References: 57]

Version ID

1

Status

MEDLINE

Authors Full Name

Bach, J R

Institution

Bach, J R. New Jersey Medical School-UMDNJ, Newark.

PMID

<https://www.ncbi.nlm.nih.gov/pmc/articles/PMC1810174>

Year of Publication

1992

Link to the Ovid Full Text or citation:

[Click here for full text options](https://ovidsp.ovid.com/ovidweb.cgi?T=JS&CSC=Y&NEWS=N&PAGE=fulltext&D=med3&AN=1586868)

Link to the External Link Resolver:

[LibKey NHS](https://libkey.io/libraries/2789/openurl?genre=article&sid=OVID:med3&genre=article&id=pmid:1586868&id=doi:&issn=0028-7091&volume=68&issue=2&spage=321&pages=321-40&date=1992&title=Bulletin+of+the+New+York+Academy+of+Medicine&atitle=Mechanical+exsufflation%2C+noninvasive+ventilation%2C+and+new+strategies+for+pulmonary+rehabilitation+and+sleep+disordered+breathing.&aulast=Bach)
