# Supplementary material for: The impact of pulmonary rehabilitation on sleep quality in patients with chronic obstructive pulmonary disease: A systematic review and meta-analysis
Source: PLoS One. 2025 Jun 4;20(6):e0318424. doi: 10.1371/journal.pone.0318424 (PMC12136455; doi:10.1371/journal.pone.0318424)
Supplement: S3 File — (DOCX) [file pone.0318424.s003.docx]

90 Trials matching (pulmonary rehabilitat*) AND (sleep) AND (chronic obstructive pulmonary disease OR COPD OR chronic bronchitis OR emphysema) in All Text

Cochrane Central Register of Controlled Trials

Issue 2 of 12, February 2023

Select all (90) Export selected citation(s)

Order by

Date added to CENTRAL - New to Old

1

Reiki and Qi-gong Therapy to Improve Negative Emotional States of Anxiety, Depression, and Stress in Type-2 Diabetics

NCT05704465

https://clinicaltrials.gov/show/NCT05704465, 2023 | added to CENTRAL: 28 February 2023 | 2023 Issue 2

CT.gov

2

The Effect of a Technology-Mediated Integrated Walking and Tai Chi Intervention on Physical Function in Veterans With COPD and Chronic Musculoskeletal Pain

NCT05701982

https://clinicaltrials.gov/show/NCT05701982, 2023 | added to CENTRAL: 28 February 2023 | 2023 Issue 2

CT.gov

3

Promoting Chronic Obstructive Pulmonary Disease Wellness through Remote Monitoring and Health Coaching A Clinical Trial (repeat)

R Benzo, J Hoult, C McEvoy, M Clark, M Benzo, M Johnson, P Novotny

Annals of the American Thoracic Society, 2022, 19(11), 1808‐1817 | added to CENTRAL: 31 January 2023 | 2023 Issue 1

PubMed Embase

4

Analysis of the Effect of Mindfulness Behavior Intervention Combined with Progressive Breathing Training on Pulmonary Function Rehabilitation in Patients with Chronic Obstructive Pulmonary Disease

S Yu, H Fan

Emergency medicine international, 2022, 2022 | added to CENTRAL: 30 November 2022 | 2022 Issue 11

Embase

5

Promoting Chronic Obstructive Pulmonary Disease Wellness through Remote Monitoring and Health Coaching: a Clinical Trial

R Benzo, J Hoult, C McEvoy, M Clark, M Benzo, M Johnson, P Novotny

Annals of the American Thoracic Society, 2022, 19(11), 1808‐1817 | added to CENTRAL: 30 November 2022 | 2022 Issue 11

PubMed

6

Efficacy of acupuncture on quality of life, functional performance, shortness of breath, and lung function in patients with Pulmonary emphysema: randomized clinical trial

RBR-99m424c

https://trialsearch.who.int/Trial2.aspx?TrialID=RBR-99m424c, 2022 | added to CENTRAL: 31 October 2022 | 2022 Issue 10

ICTRP

7

HOME REHABILITATION FOR COPD: a RANDOMIZED STUDY OF REMOTE ACTIVITY MONITORING AND HEALTH COACHING (repeat)

RP Benzo, E Mcevoy C, M Clark M, M Benzo, M Johnson M, PAUL Novotny

Chest, 2022, 162(4), A1981‐ | added to CENTRAL: 31 October 2022 | 2022 Issue 10

Embase

8

Unsupported Upper Limb Exercise test modification and assessment of measure properties in individuals with Chronic Respiratory Diseases and Post Covid 19 (no data)

RBR-6myq2rc

https://trialsearch.who.int/Trial2.aspx?TrialID=RBR-6myq2rc, 2022 | added to CENTRAL: 31 October 2022 | 2022 Issue 10

ICTRP

9

Evaluation of pulmonary rehabilitation training in patients with chronic obstructive pulmonary disease complicated with obstructive sleep apnea hypopnea syndrome: a 12-week, single center, double-blind, randomized controlled study

ChiCTR2100052959

https://trialsearch.who.int/Trial2.aspx?TrialID=ChiCTR2100052959, 2021 | added to CENTRAL: 30 September 2022 | 2022 Issue 9

ICTRP

10

Promoting COPD Wellness through Remote Monitoring and Health Coaching: a Randomized Study

R Benzo, J Hoult, C McEvoy, M Clark, M Benzo, M Johnson, P Novotny

Annals of the American Thoracic Society, 2022 | added to CENTRAL: 31 August 2022 | 2022 Issue 8

PubMed Embase

11

Efeitos da suplementação de arginina e beterraba desidratada na função vasos sanguíneos de obrigação com Doença Pulmonar Obstrutiva Crônica relacionada à pulmonar pulmonar

RBR-8hhzfv9

https://trialsearch.who.int/Trial2.aspx?TrialID=RBR-8hhzfv9, 2021 | added to CENTRAL: 31 August 2022 | 2022 Issue 8

ICTRP

12

Prospective Out-Patient Study of Pulmonary Rehabilitation for Long COVID: pilot Study

MR Windt, L Flanagan, H Mullaney

American journal of respiratory and critical care medicine, 2022, 205(1) | added to CENTRAL: 31 July 2022 | 2022 Issue 07

Embase

13

Effect of Home-Based Rehabilitation with Health Coaching on Chronic Obstructive Pulmonary Disease Outcomes: a Randomized Study

R Benzo, JP Hoult, CE Mcevoy, M Clark, MV Benzo, MM Johnson, P Novotny

American journal of respiratory and critical care medicine, 2022, 205(1) | added to CENTRAL: 31 July 2022 | 2022 Issue 07

Embase

14

Six weeks of oral Echinacea purpurea supplementation does not enhance the production of serum erythropoietin or erythropoietic status in recreationally active males with above-average aerobic fitness

TD Martin, MS Green, MT Whitehead, TP Scheett, MJ Webster, GM Hudson

Applied physiology, nutrition & metabolism, 2019, 44(7), 791‐795 | added to CENTRAL: 31 July 2022 | 2022 Issue 07

CINAHL

15

Effects of the P‐Glycoprotein Inhibitor Clarithromycin on the Pharmacokinetics of Intravenous and Oral Trospium Chloride: a 4‐Way Crossover Drug‐Drug Interaction Study in Healthy Subjects

BT Abebe, M Weiss, C Modess, T Roustom, T Tadken, D Wegner, U Schwantes, C Neumeister, H Schulz, E Scheuch, W Siegmund

Journal of clinical pharmacology, 2019, 59(10), 1319‐1330 | added to CENTRAL: 31 July 2022 | 2022 Issue 07

CINAHL

16

Enhancing skill transfer in tennis using representative learning design

L Krause, D Farrow, R Pinder, T Buszard, S Kovalchik, M Reid

Journal of sports sciences, 2019, 37(22), 2560‐2568 | added to CENTRAL: 31 July 2022 | 2022 Issue 07

CINAHL

17

The Effect of Progressive Relaxation and Breathing Exercise on Sleep Quality and Exercise Self-Efficacy in Patients With COPD(no data)

NCT05397275

https://clinicaltrials.gov/show/NCT05397275, 2022 | added to CENTRAL: 30 June 2022 | 2022 Issue 06

CT.gov

18

Using a smartphone application maintains physical activity following pulmonary rehabilitation in patients with COPD: a randomised controlled trial(repeat)

M Spielmanns, R Gloeckl, I Jarosch, D Leitl, T Schneeberger, T Boeselt, S Huber, P Kaur-Bollinger, B Ulm, C Mueller, J Bjoerklund, S Spielmanns, W Windisch, AM Pekacka-Egli, AR Koczulla

Thorax, 2022 | added to CENTRAL: 31 May 2022 | 2022 Issue 05

PubMed Embase

19

The Effect of Interval Exercise on Functional Outcomes in Veterans With COPD and OSA (no data)

NCT05254431

https://clinicaltrials.gov/show/NCT05254431, 2022 | added to CENTRAL: 31 March 2022 | 2022 Issue 03

CT.gov

20

Long-term exercise after pulmonary rehabilitation (LEAP): a pilot randomised controlled trial of Tai Chi in COPD

ML Moy, PM Wayne, D Litrownik, D Beach, ES Klings, RB Davis, A Pinheiro, GY Yeh

ERJ open research, 2021, 7(3) | added to CENTRAL: 31 August 2021 | 2021 Issue 08

Embase

21

The effects of morphine on breathlessness and sleep in chronic obstructive pulmonary disease: a randomised, double-blind, placebo controlled cross-over study

ACTRN12621000752864

https://trialsearch.who.int/Trial2.aspx?TrialID=ACTRN12621000752864, 2021 | added to CENTRAL: 31 July 2021 | 2021 Issue 07

ICTRP

22

Analysis of the Cost-Effectiveness of Same-day Discharge Surgery for Primary Total Hip Arthroplasty

NCT04900181

https://clinicaltrials.gov/show/NCT04900181, 2021 | added to CENTRAL: 30 June 2021 | 2021 Issue 06

CT.gov

23

Feasibility of a Self-guided Exercise Program Among Hospitalized COVID-19 Patients

NCT04821531

https://clinicaltrials.gov/show/NCT04821531, 2021 | added to CENTRAL: 30 April 2021 | 2021 Issue 04

CT.gov

24

Internet-based Cognitive-behavioral Treatment for Insomnia in COPD Patients Undergoing Pulmonary Rehabilitation (no data)

NCT04700098

https://clinicaltrials.gov/show/NCT04700098, 2021 | added to CENTRAL: 28 February 2021 | 2021 Issue 02

CT.gov

25

Rehabilitation With HHFNC in COPD in Nocturnal NIV

NCT04683952

https://clinicaltrials.gov/show/NCT04683952, 2020 | added to CENTRAL: 31 January 2021 | 2021 Issue 01

CT.gov

26

The Impact of Pulmonary Rehabilitation on 24-Hour Movement Behavior in People With Chronic Obstructive Pulmonary Disease: new Insights From a Compositional Perspective (repeat)

AT Burge, J Palarea-Albaladejo, AE Holland, MJ Abramson, CF McDonald, A Mahal, CJ Hill, AL Lee, NS Cox, A Lahham, R Moore, C Nicolson, P O'Halloran, R Gillies, SFM Chastin

Journal of physical activity & health, 2021, 18(1), 13‐20 | added to CENTRAL: 31 January 2021 | 2021 Issue 01

PubMed Embase

27

Short- and long-term changes in cognitive function after exercise-based rehabilitation in people with COPD: a pilot study

B Rosenstein, A Smyrnova, A Rizk, F Escudier, J-F Gagnon, V Pepin

Canadian journal of respiratory, critical care, and sleep medicine, 2020 | added to CENTRAL: 31 December 2020 | 2020 Issue 12

Embase

28

Effects of personalized exercise training on physical activity, health-related fitness, sleep quality, and quality of life among middle-aged and older adults with multiple health problems

ISRCTN44497123

https://trialsearch.who.int/Trial2.aspx?TrialID=ISRCTN44497123, 2020 | added to CENTRAL: 30 November 2020 | 2020 Issue 11

ICTRP

29

Efficacy of nasal high flow therapy on the coordination between breathing and swallowing of saliva during daytime nap in chronic obstructive pulmonary disease patients: a single center, randomized crossover controlled study

T Ayuse, N Hisamatsu, T Yamaguchi, Y Takahashi, Y Tamada, S Kurata, G Mishima, M Pinkham, S Tatkov, H Takahata, T Ayuse

Medicine, 2020, 99(34), e21778 | added to CENTRAL: 30 September 2020 | 2020 Issue 09

PubMed

30

Does Liuzijue Qigong affect anxiety in patients with chronic obstructive pulmonary disease, even during the COVID-19 outbreak? a randomized, controlled trial

Y-X Zhang, Y Quan, M-H Chen, D Zhang, Y Zhang, Z-G Zhu

Traditional medicine research, 2020, 5(4), 216‐228 | added to CENTRAL: 30 September 2020 | 2020 Issue 09

Embase

31

Impact of a smartphone application (KAIA COPD app) in combination with Activity Monitoring as a maintenance prOgram following PUlmonary Rehabilitation in COPD: the protocol for the AMOPUR Study, an international, multicenter, parallel group, randomized, controlled study

M Spielmanns, T Boeselt, S Huber, P Kaur Bollinger, B Ulm, AM Peckaka-Egli, I Jarosch, T Schneeberger, S Schoendorf, R Gloeckl, AR Koczulla

Trials, 2020, 21(1), 636 | added to CENTRAL: 31 August 2020 | 2020 Issue 08

PubMed Embase

32

Effects of oral melatonin associated with pulmonary rehabilitation on sleep quality and daytime sleepiness in COPD

CV Souza, SMNR Viana, VMS Bruin, PFC Bruin, TAR Farias, OMV Rego, GDNR Viana

Sleep Science, 2020, 13, 101‐ | added to CENTRAL: 31 May 2020 | 2020 Issue 05

Embase

33

Study on the Prevention and Control System of Chronic Airway Diseases

NCT04348344

https://clinicaltrials.gov/show/NCT04348344, 2020 | added to CENTRAL: 30 April 2020 | 2020 Issue 04

CT.gov

34

Smartphone-App as Maintenance Program in COPD

NCT04299165

https://clinicaltrials.gov/show/NCT04299165, 2020 | added to CENTRAL: 31 March 2020 | 2020 Issue 03

CT.gov

35

Effects of pulmonary rehabilitation-associated melatonin hormone on sensation of difficulty breathing, respiratory muscle strength, ability to perform daily living activities and sleep quality in individuals with chronic lung disease

RBR-99gyq8

https://trialsearch.who.int/Trial2.aspx?TrialID=RBR-99gyq8, 2019 | added to CENTRAL: 29 February 2020 | 2020 Issue 02

ICTRP

36

The Effect of Body Awareness Therapy on Postural Stability, Balance and Fear of Falling in Patients With COPD

NCT04212676

https://clinicaltrials.gov/show/NCT04212676, 2019 | added to CENTRAL: 31 January 2020 | 2020 Issue 01

CT.gov

37

The Additional Value of Noninvasive Ventilation Next to Rehabilitation in Hypercapnic COPD Patients

NCT00135538

https://clinicaltrials.gov/show/NCT00135538, 2005 | added to CENTRAL: 31 January 2020 | 2020 Issue 01

CT.gov

38

Use of time in chronic obstructive pulmonary disease: longitudinal associations with symptoms and quality of life using a compositional analysis approach (repeat)

H Lewthwaite, T Olds, MT Williams, TW Effing, D Dumuid

PloS one, 2019, 14(3), e0214058 | added to CENTRAL: 31 January 2020 | 2020 Issue 01

PubMed

39

Changes in cognitive function after exercise-based rehabilitation in people with chronic obstructive pulmonary disease (COPD)

B Rosenstein, A Rizk, F Escudier, J-F Gagnon, V Pepin

Canadian journal of respiratory, critical care, and sleep medicine, 2019, 3, 39‐ | added to CENTRAL: 30 September 2019 | 2019 Issue 09

Embase

40

Evaluating a group-based maintenance self-management intervention for patients with COPD

ISRCTN30110012

https://trialsearch.who.int/Trial2.aspx?TrialID=ISRCTN30110012, 2019 | added to CENTRAL: 30 September 2019 | 2019 Issue 09

ICTRP

41

Impact of a Smartphone application (KAIA COPD-App) in combination with Activity Monitoring as maintenance program following pulmonary rehabilitation in COPD : an international multi-centered randomised controlled trial

DRKS00017275

https://trialsearch.who.int/Trial2.aspx?TrialID=DRKS00017275, 2019 | added to CENTRAL: 30 September 2019 | 2019 Issue 09

ICTRP

42

Evaluation of the canadian standardized rehabilitation efficacy (CONSPIRE) trial

A-M Selzler, T Jourdain, J Wald, M Sedeno, T Janaudis-Ferreira, R Goldstein, J Bourbeau, M Stickland

Canadian journal of respiratory, critical care, and sleep medicine, 2019, 3, 47‐48 | added to CENTRAL: 30 September 2019 | 2019 Issue 09

Embase

43

Relationship of the COTE index with pulmonary rehabilitation outcomes

A Burge, C Mcdonald, A Mahal, C Hill, A Lee, N Cox, R Moore, C Nicolson, P O'Halloran, A Lahham, R Gillies, A Holland

European respiratory journal, 2018, 52 | added to CENTRAL: 30 April 2019 | 2019 Issue 04

Embase

44

Functionality of patients with Chronic Obstructive Pulmonary Disease at 3 months follow-up after elastic resistance training: a randomized clinical trial

IG Silva, BSA Silva, APCF Freire, APSD Santos, FF Lima, D Ramos, EMC Ramos

Pulmonology, 2018, 24(6), 354‐357 | added to CENTRAL: 31 March 2019 | 2019 Issue 3

PubMed Embase

45

Functionality of patients with Chronic Obstructive Pulmonary Disease at 3 months follow-up after elastic resistance training: a randomized clinical trial

IRCT2016080124080N3

https://trialsearch.who.int/Trial2.aspx?TrialID=IRCT2016080124080N3, 2017 | added to CENTRAL: 31 March 2019 | 2019 Issue 3

ICTRP

46

Effectiveness of home based pulmonary rehabilitation for COPD patients with OSA: overlap syndrome

J Oliveira, A Silva, A Brunetti, E Perez, J Urbano, G Insalaco, LV Oliveira, L Sampaio

European respiratory journal, 2017, 50 | added to CENTRAL: 31 March 2019 | 2019 Issue 3

Embase

47

Controlled breathing and sleep quality in patient with Chronic Obstructive Pulmonary Disease

IRCT2014060717989N1

https://trialsearch.who.int/Trial2.aspx?TrialID=IRCT2014060717989N1, 2015 | added to CENTRAL: 31 March 2019 | 2019 Issue 3

ICTRP

48

Does exercising COPD patients on non invasive ventilation (NIV) enable them to walk further? A randomised controlled trial

K Buchan, K Badlan, M Fletcher, A Kendrick

European respiratory journal, 2014, 44 | added to CENTRAL: 31 March 2019 | 2019 Issue 3

Embase

49

The effect of dual bronchodilation versus single bronchodilation on 24-hour hyperinflation in COPD patients

EUCTR2016-003989-12-NL

https://trialsearch.who.int/Trial2.aspx?TrialID=EUCTR2016-003989-12-NL, 2017 | added to CENTRAL: 31 March 2019 | 2019 Issue 3

ICTRP

50

A clinical trial to identify factors affecting quality of life in COPD

CTRI/2018/09/015707

https://trialsearch.who.int/Trial2.aspx?TrialID=CTRI/2018/09/015707, 2018 | added to CENTRAL: 31 March 2019 | 2019 Issue 3

ICTRP

51

The effects of pulmonary rehabilitation in patients with non-cystic fibrosis bronchiectasis-A randomised controlled trial

R Kumar, R Guleria, GC Khilnani, A Mohan, K Madan, V Hadda, RM Pandey

European respiratory journal, 2017, 50 | added to CENTRAL: 31 March 2019 | 2019 Issue 3

Embase

52

Effect of opioids on outcomes of pulmonary rehabilitation

ACTRN12615000121561

https://trialsearch.who.int/Trial2.aspx?TrialID=ACTRN12615000121561, 2015 | added to CENTRAL: 31 March 2019 | 2019 Issue 3

ICTRP

53

Breathing new life into respiratory medicine? Report of the 2004 BTS Winter Meeting

DJ Powrie, JR Hurst

Thorax, 2005, 60(3), 183‐186 | added to CENTRAL: 31 March 2019 | 2019 Issue 3

PubMed Embase

54

The interrelations among aspects of dyspnea and symptoms of depression in COPD patients - a network analysis

M Schuler, M Wittmann, H Faller, K Schultz

Journal of affective disorders, 2018, 240, 33‐40 | added to CENTRAL: 28 February 2019 | 2019 Issue 2

PubMed

55

Nordic walking using activator poles increases exercise tolerance in individuals with COPD compared to healthy controls

D Anekwe, R Lisa, M De Marchie, S Antoniades, J Chung Lim, N Gandhi, M Montambault, I Barreira, A Morin, N Muntini, J Spahija

Canadian journal of respiratory critical care and sleep medicine, 2017, 1(2), 112‐ | added to CENTRAL: 30 November 2018 | 2018 Issue 11

Embase

56

Development of the Canadian standardized pulmonary rehabilitation efficacy trial: a protocol update

A-M Selzler, T Jourdain, M Sedeno, J Wald, T Janaudis-Ferreira, R Goldstein, J Bourbeau, M Stickland

Canadian journal of respiratory critical care and sleep medicine, 2017, 1(3), 170‐ | added to CENTRAL: 30 November 2018 | 2018 Issue 11

Embase

57

Lung function parameters: good predictors of change in physical activity after pulmonary rehabilitation program?

S Georges, V Pepin, K Duckworth, R Yasri, G Moullec

Canadian journal of respiratory critical care and sleep medicine, 2017, 1(3), 167‐168 | added to CENTRAL: 30 November 2018 | 2018 Issue 11

Embase

58

Let's boogie: feasibility of a dance intervention in patients with chronic obstructive pulmonary disease

A Wshah, S Butler, R Goldstein, D Brooks

Canadian journal of respiratory critical care and sleep medicine, 2018, 2(2), 112‐ | added to CENTRAL: 30 November 2018 | 2018 Issue 11

Embase

59

The impact of pulmonary rehabilitation on people with mild chronic obstructive pulmonary disease: a randomised controlled trial

A Lahham, CF McDonald, NS Cox, S Rawlings, A Nichols, R Moore, A Liacos, AE Holland

American journal of respiratory and critical care medicine, 2018, 197(MeetingAbstracts) | added to CENTRAL: 31 August 2018 | 2018 Issue 8

Embase

60

Pulmonary rehabilitation does not improve objective measures of sleep quality in people with chronic obstructive pulmonary disease

NS Cox, V Pepin, A Burge, A Mahal, CJ Hill, A Lee, R Moore, C Nicolson, P O'Halloran, A Lahham, R Gillies, CF McDonald, AE Holland

American journal of respiratory and critical care medicine, 2018, 197(MeetingAbstracts) | added to CENTRAL: 31 August 2018 | 2018 Issue 8

Embase

61

Pulmonary rehabilitation does not improve objective measures of sleep quality in people with chronic obstructive pulmonary disease

N Cox, V Pepin, A Burge, A Mahal, C Hill, A Lee, R Moore, C Nicholson, P O'halloran, A Lahham, R Gillies, C McDonald, A Holland

Respirology (Carlton, Vic.), 2018, Conference: Australia and New Zealand Society of Respiratory Science and the Thoracic Society of Australia and New Zealand Annual Scientific Meeting , ANZSRS/TSANZ 2018. Australia. 23(Supplement 1), 89 | added to CENTRAL: 31 August 2018 | 2018 Issue 8

Embase

62

Effects of losartan and allopurinol on cardiorespiratory regulation in obstructive sleep apnoea

BJ Morgan, M Teodorescu, DF Pegelow, ER Jackson, DL Schneider, DT Plante, JP Gapinski, SJ Hetzel, JM Dopp

Experimental physiology, 2018, 103(7), 941‐955 | added to CENTRAL: 31 August 2018 | 2018 Issue 8

PubMed Embase

63

Telerehabilitation versus traditional centre-based pulmonary rehabilitation for people with chronic respiratory disease: protocol for a randomised controlled trial

NS Cox, CF McDonald, JA Alison, A Mahal, R Wootton, CJ Hill, J Bondarenko, H Macdonald, P O'Halloran, P Zanaboni, K Clarke, D Rennick, K Borgelt, AT Burge, A Lahham, B Wageck, H Crute, P Czupryn, A Nichols, AE Holland

BMC pulmonary medicine, 2018, 18(1) | added to CENTRAL: 30 June 2018 | 2018 Issue 6

Embase

64

Participation in pulmonary rehabilitation did not significantly alter time use patterns in people with COPD

T Hunt, M Williams, T Olds, D Dumuid

Respirology (Carlton, Vic.), 2018, 23, 117‐ | added to CENTRAL: 30 June 2018 | 2018 Issue 6

Embase

65

Validation of an Exercise DVD for Maintenance After Pulmonary Rehabilitation

NCT01235481

https://clinicaltrials.gov/show/NCT01235481, 2010 | added to CENTRAL: 31 May 2018 | 2018 Issue 5

CT.gov

66

Problem-Solving Therapy for People With Major Depression and Chronic Obstructive Pulmonary Disease

NCT00601055

https://clinicaltrials.gov/show/NCT00601055, 2008 | added to CENTRAL: 31 May 2018 | 2018 Issue 5

CT.gov

67

A Trial to Study the Effects of Pulmonary Rehabilitation Program on Exercise Capacity and Quality of Life in Patients With Severe Form of Chronic Obstructive Pulmonary Disease (COPD)

NCT02512549

https://clinicaltrials.gov/show/NCT02512549, 2015 | added to CENTRAL: 31 May 2018 | 2018 Issue 5

CT.gov

68

Effects of a Comprehensive Health Coaching Program in Advanced Chronic Obstructive Pulmonary Disease (cannot available)

NCT03398772

https://clinicaltrials.gov/show/NCT03398772, 2018 | added to CENTRAL: 31 May 2018 | 2018 Issue 5

CT.gov

69

Pulmonary rehabilitation with balance training for fall reduction in chronic obstructive pulmonary disease: protocol for a randomized controlled trial

MK Beauchamp, D Brooks, C Ellerton, A Lee, J Alison, PG Camp, G Dechman, K Haines, SL Harrison, AE Holland, A Marques, R Moineddin, EH Skinner, L Spencer, MK Stickland, F Xie, RS Goldstein

JMIR research protocols, 2017, 6(11), e228 | added to CENTRAL: 28 February 2018 | 2018 Issue 2

PubMed

70

Interdisciplinary model of care (RADICALS) for early detection and management of chronic obstructive pulmonary disease (COPD) in Australian primary care: study protocol for a cluster randomised controlled trial

J Liang, MJ Abramson, N Zwar, G Russell, AE Holland, B Bonevski, A Mahal, BV Hecke, K Phillips, P Eustace, E Paul, K Petrie, S Wilson, J George

BMJ open, 2017, 7(9), e016985 | added to CENTRAL: 31 December 2017 | 2017 Issue 12

PubMed

71

Does home-based pulmonary rehabilitation improve functional capacity, peripheral muscle strength and quality of life in patients with bronchiectasis compared to standard care?

A José, AE Holland, CS Oliveira, JPR Selman, RAS Castro, RA Athanazio, SZ Rached, A Cukier, R Stelmach, SD Corso

Brazilian journal of physical therapy, 2017, 21(6), 473‐480 | added to CENTRAL: 31 December 2017 | 2017 Issue 12

PubMed

72

The use of high frequency airway oscillations in chronic obstructive pulmonary disease-a pilot study

E Daynes, TC Harvey-Dunstan, L Houchen-Wolloff, SJ Singh

American journal of respiratory and critical care medicine, 2017, 195 | added to CENTRAL: 30 September 2017 | 2017 Issue 9

Embase

73

Patient Involvement in the Design of a Patient-Centered Clinical Trial to Promote Adherence to Supplemental Oxygen Therapy in COPD

KE Holm, R Casaburi, S Cerreta, HA Gussin, J Husbands, J Porszasz, V Prieto-Centurion, RA Sandhaus, JL Sullivan, LJ Walsh, JA Krishnan

Patient, 2016, 9(3), 271‐279 | added to CENTRAL: 31 March 2017 | 2017 Issue 3

PubMed

74

Aerobic Exercise Combined With Noninvasive Positive Pressure Ventilation Increases Serum Brain-Derived Neurotrophic Factor in Healthy Males

T Kawazu, T Nakamura, T Moriki, Y-I Kamijo, Y Nishimura, T Kinoshita, F Tajima

PM and R. (no pagination), 2016, 2016, Date of Publication: September 16 | added to CENTRAL: 28 February 2017 | 2017 Issue 2

Embase

75

Sleep disorders breathing in COPD patients after pulmonary rehabilitation

JC Oliveira, EA Perez, G Insalaco, LVF Oliveira

Journal of sleep research, 2016, 25, 270‐ | added to CENTRAL: 31 October 2016 | 2016 Issue 10

Embase

76

Alcohol at bedtime induces minor changes in sleep stages and blood gases in stable chronic obstructive pulmonary disease

NH Holmedahl, B Øverland, O Fondenes, I Ellingsen, JA Hardie

Schlaf & Atmung [Sleep & breathing], 2015, 19(1), 307‐314 | added to CENTRAL: 30 April 2016 | 2016 Issue 4

PubMed Embase

77

Cardiovascular risk and mortality in end-stage renal disease patients undergoing dialysis: sleep study, pulmonary function, respiratory mechanics, upper airway collapsibility, autonomic nervous activity, depression, anxiety, stress and quality of life: a prospective, double blind, randomized controlled clinical trial

I dos Reis Santos, AR Danaga, I de Carvalho Aguiar, EF Oliveira, IS Dias, JJ Urbano, AA Martins, LM Ferraz, NT Fonsêca, V Fernandes, VA Fernandes, VC Lopes, FS Leitão Filho, SR Nacif, T de Carvalho Pde, LM Sampaio, LC Giannasi, S Romano, G Insalaco, AK Araujo, H Dellê, NK Souza, D Giannella-Neto, LV Oliveira

BMC nephrology, 2013, 14, 215 | added to CENTRAL: 31 January 2016 | 2016 Issue 1

PubMed

78

Qualitative analysis of satisfaction of patients with chronic obstructive pulmonary disease (COPD) submitted to exercise training in water and on land

DR Carvalho, LS Vidotto, MF Merli, JM Felcar, F Pitta, DS Fujisawa, VS Probst

European respiratory journal, 2014, 44 | added to CENTRAL: 30 November 2015 | 2015 Issue 11

Embase

79

Effectiveness of repeated courses of pulmonary rehabilitation on functional exercise capacity in patients with COPD

A Atabaki, J Fine, M Haggerty, C Marolda, D Wakefield, A Yu, R ZuWallack

Journal of cardiopulmonary rehabilitation and prevention, 2015, 35(4), 272‐277 | added to CENTRAL: 30 November 2015 | 2015 Issue 11

PubMed Embase

80

Zopiclone effects on breathing at sleep in stable chronic obstructive pulmonary disease

NH Holmedahl, B Øverland, O Fondenes, I Ellingsen, JA Hardie

Schlaf & Atmung [Sleep & breathing], 2015, 19(3), 921‐930 | added to CENTRAL: 28 February 2015 | 2015 Issue 2

PubMed

81

Benefits of pulmonary rehabilitation in idiopathic pulmonary fibrosis

JJ Swigris, DL Fairclough, M Morrison, B Make, E Kozora, KK Brown, FS Wamboldt

Respiratory care, 2011, 56(6), 783‐789 | added to CENTRAL: 31 January 2014 | 2014 Issue 1

Embase

82

Subjective sleep quality during average volume assured pressure support (AVAPS) ventilation in patients with hypercapnic COPD: a physiological pilot study

E Crisafulli, G Manni, M Kidonias, L Trianni, EM Clini

Lung, 2009, 187(5), 299‐305 | added to CENTRAL: 30 April 2010 | 2010 Issue 2

PubMed

83

Feasibility study of noninvasive ventilation with helium-oxygen gas flow for chronic obstructive pulmonary disease during exercise

PF Allan, KV Thomas, MR Ward, AD Harris, GA Naworol, JA Ward

Respiratory care, 2009, 54(9), 1175‐1182 | added to CENTRAL: 31 January 2010 | 2010 Issue 1

PubMed

84

Effects of lung volume reduction surgery on sleep quality and nocturnal gas exchange in patients with severe emphysema

SL Krachman, W Chatila, UJ Martin, T Nugent, J Crocetti, J Gaughan, GJ Criner

Chest, 2005, 128(5), 3221‐3228 | added to CENTRAL: 30 April 2006 | 2006 Issue 2

PubMed Embase

85

Endurance and strength training in patients with COPD

MJ Mador, E Bozkanat, A Aggarwal, M Shaffer, TJ Kufel

Chest, 2004, 125(6), 2036‐2045 | added to CENTRAL: 31 October 2004 | 2004 Issue 4

PubMed

86

Oxygen saturation at rest, on exercise and during sleep in COPD patients undergoing pulmonary rehabilitation program. Two years prospective controlled study

P Piszko, J Lewczuk, M Kowalska-Superlak, K Wrabec

Pneumonologia i alergologia polska, 2002, 70(11‐12), 566‐572 | added to CENTRAL: 31 January 2004 | 2004 Issue 1

PubMed

87

The Italian multicentre study on noninvasive ventilation in chronic obstructive pulmonary disease patients

E Clini, C Sturani, A Rossi, S Viaggi, A Corrado, CF Donner, N Ambrosino

The european respiratory journal, 2002, 20(3), 529‐538 | added to CENTRAL: 30 April 2003 | 2003 Issue 2

PubMed Embase

88

Development of nocturnal oxygen desaturation, sleep disorders, and pulmonary function during rehabilitation in chronic obstructive bronchitis

F Raschke, E Schlenker, J Fischer

European respiratory journal - supplement, 1990, 3(Suppl 10), 326s | added to CENTRAL: 31 January 2003 | 2003 Issue 1

89

Therpeutic experiences with a new antitussive principle

V Böhlau, G Schildwächter

Fortschritte der Medizin, 1976, 94(34), 2051‐2054 | added to CENTRAL: 30 April 1999 | 1999 Issue 2

PubMed

90

The effects of doxofylline versus theophylline on sleep architecture in COPD patients

C Sacco, A Braghiroli, E Grossi, CF Donner

Monaldi archives for chest disease = archivio monaldi per LE malattie del torace, 1995, 50(2), 98‐103 | added to CENTRAL: 31 January 1998 | 1998 Issue 1

PubMed Embase
