# Supplementary material for: The impact of pulmonary rehabilitation on sleep quality in patients with chronic obstructive pulmonary disease: A systematic review and meta-analysis
Source: PLoS One. 2025 Jun 4;20(6):e0318424. doi: 10.1371/journal.pone.0318424 (PMC12136455; doi:10.1371/journal.pone.0318424)
Supplement: S6 File — (DOCX) [file pone.0318424.s006.docx]

| **Section and Topic** | **Item #** | **Checklist item** | **Location where item is reported** |
| --- | --- | --- | --- |
| **TITLE** | | |  |
| Title | 1 | The impact of pulmonary rehabilitation on sleep quality in patients with chronic obstructive pulmonary disease: A systematic review and meta-analysis | P1 |
| **ABSTRACT** | | |  |
| Abstract | 2 | **Background:** Pulmonary rehabilitation (PR) is a significant component of chronic obstructive pulmonary disease (COPD) management, but whether it has any impact on patient sleep quality is unknown.  **Methods:** We conducted a systematic review of the literature to evaluate the impact of PR on sleep quality in patients with COPD. Searches of the MEDLINE, EMBASE and Cochrane Library databases were performed, and data were extracted from relevant studies. Random effects meta-analysis was performed to determine if PR is associated with any difference in the Pittsburgh Sleep Quality Index (PSQI).  **Results:** A total of 16 studies were included in this review with 1,478 patients, of which 1,169 took part in PR. The PR programmes were variable, some being 8-week programmes while others were 12-week, and some lasted for 6 months. The pooled results of 372 patients suggest that PR is associated with a significant improvement in the PSQI (mean difference 2.33 95% CI 0.46 to 4.20, I2=93%, 6 studies). Removal of one study, significantly reduced the statistical heterogeneity and the effect size (mean difference 0.91 95% CI 0.35-1.48, I2=0%, 5 studies). For other outcomes such as sleep onset latency, wake after sleep onset, total sleep time, sleep efficiency, and number of awakenings, there were no consistencies to suggest any benefit associated with PR.  **Conclusions:** PR appears to be associated with improvements in sleep quality in patients with COPD. More studies are needed to determine what the ideal PR programme is that best improves sleep quality and whether certain patients benefit more compared to others. | P2 |
| **INTRODUCTION** | | |  |
| Rationale | 3 | Approximately 70 per cent of patients with COPD have typical complaints of sleep difficulties, and PR can help to reduce anxiety and depression in patients with COPD and have a positive impact on sleep quality. However, whether PR influences sleep in patients with COPD is controversial, as some studies have had inconsistent results in this area. There is no systematic review to integrate the results of these studies to draw robust conclusions. | P3 |
| Objectives | 4 | To explore whether PR can improve sleep quality in COPD patients. | P4 |
| **METHODS** | | |  |
| Eligibility criteria | 5 | Studies were included if they evaluated sleep quality in people with COPD who underwent PR. There was no restriction on study design which could be observational or clinical trial. However, studies which included adults were included. Studies had to have original data, so letters, editorials, comments, and reviews were excluded. The reference lists of potentially relevant studies were checked for additional studies. | P4 |
| Information sources | 6 | We used the OVID platform to search for relevant studies on MEDLINE and EMBASE in February 2023 and Cochrane Library database in March 2023. | P4 |
| Search strategy | 7 | The specific search terms were (pulmonary rehabilitat*) AND (sleep) AND (chronic obstructive pulmonary disease OR COPD OR chronic bronchitis OR emphysema). | P4 |
| Selection process | 8 | The screening of studies was done independently by two reviewers (SD and CSK). | P5 |
| Data collection process | 9 | Two reviewers (SD and CSK) independently collected data. | P5 |
| Data items | 10a | Both reviewers (SD and CSK) collected data on study design, country, year, number of patients, mean age, proportion of men and study inclusion criteria. In addition, definitions of PR, definitions of sleep quality, follow up and result, and concordance to PR were also collected. | P5 |
|  | 10b | / |  |
| Study risk of bias assessment | 11 | Study quality assessment was conducted independently by two reviewers (SD and CSK) using the Ottawa-Newcastle scale. Stars were awarded, out of a maximum possible score of 9 stars, based on whether a study was representative of a general cohort of patients with COPD, involved the selection of a control group (cohort prior to PR), ascertainment of PR, demonstration that sleep quality was assessed at baseline, comparability of the cohort, ascertainment of the sleep quality, adequacy of the length of follow up, and information on loss to follow up. | P5 |
| Effect measures | 12 | Mean difference used in the synthesis or presentation of results. | P5 |
| Synthesis methods | 13a | / |  |
|  | 13b | / |  |
|  | 13c | Collect and tabulate the data mentioned above, synthesise, and analyse the data using narrative and meta-analysis. |  |
|  | 13d | RevMan 5.4 (The Nordic Cochrane Centre, The Cochrane Collaboration, Copenhagen, Denmark) was employed in performing random-effects meta-analysis using the mean difference method. The mean and standard deviation in the Pittsburgh Sleep Quality Index (PSQI) before and after PR were pooled. | P5 |
|  | 13e | Statistical heterogeneity was assessed using the I^2^ statistic, with I^2^ values of between 30%-60% representing a moderate degree of heterogeneity. | P5 |
|  | 13f | We undertook leave-one-out sensitivity analyses to determine the source of statistical heterogeneity. | P5 |
| Reporting bias assessment | 14 | we planned to perform an asymmetry test to determine whether there was publication bias. | P5 |
| Certainty assessment | 15 | / |  |
| **RESULTS** | | |  |
| Study selection | 16a | The search of MEDLINE and EMBASE yielded 258 potentially relevant records and the search of the Cochrane Trial Register yielded 90 potentially relevant records. A total of 16 studies were included after screening titles and abstracts and reviewing full texts of the relevant studies. | P5 |
|  | 16b | See Figure 1 PRISMA flow diagram of study selection in manuscript for details. | P6 |
| Study characteristics | 17 | The included studies in this review were 16 studies which comprised 4 randomized controlled trials, 9 prospective cohort studies, and 3 retrospective cohort studies. Among these studies, there were a total of 1478 patients of which 1169 took part in PR. The average age across 13 studies, that reported mean age, was 66.6 years and the proportion of male patients was 64.1%. | P6 |
| Risk of bias in studies | 18 | Apart from two studies that only included veterans, all the remaining studies were considered to be representative of a COPD cohort. All studies had reliable control group selections, reliable ascertainment that patients received PR treatment, and sleep quality ascertainment measures. Sleep quality was assessed at baseline, so for all studies, the demonstration that the outcome was not present at the start of the study was reliable. Four studies were randomized controlled trials which included a comparable cohort with and without PR. Follow ups were variable, up to 12 months and 8 studies had low levels of missing data or data that were lost to follow up. The average number of stars derived using the Newcastle-Ottawa scale was 6.9. | P7 |
| Results of individual studies | 19 | The results of the individual studies are detailed in Tables 1 and 3. | P6-P7 |
| Results of syntheses | 20a | The PR programmes were variable, some were 8-weeks in length while others were 12-weeks or 12 months. | P7 |
|  | 20b | A total of 6 studies were included in the pooled analysis of PR on sleep quality as assessed by the PSQI. A total of 372 patients underwent PR. The pooled results suggest that PR is associated with a significant improvement in sleep quality (mean difference 2.33 95% CI 0.46 to 4.20, I^2^=93%, 6 studies). | P8 |
|  | 20c | However, the source of the heterogeneity was the study by Jin et al. | P8 |
|  | 20d | If Jin et al is removed the estimate would still be significantly different but the effect size would be smaller with no statistical heterogeneity (mean difference 0.91 95%CI 0.35-1.48, I^2^=0%, 5 studies). | P8-P9 |
| Reporting biases | 21 | / |  |
| Certainty of evidence | 22 | / |  |
| **DISCUSSION** | | |  |
| Discussion | 23a | PR appears to be associated with improvements in sleep quality in patients with COPD and PR should be recommended for patients who feel that their sleep quality requires improvement. | P10-P11 |
|  | 23b | The included studies did not have a consistent definition of PR and sleep quality. | P11 |
|  | 23c | Firstly, six of the included studies were only available as conference abstracts, which lack detailed information regarding methodology and results. Secondly, the average score for the quality of studies included was only 6.9, suggesting that the overall quality could be improved. Thirdly, there is significant methodology heterogeneity in the included studies as there is no consistent definition for PR in terms of time, activities, or periodicity of implementation. Finally, the measurement of sleep quality was variable, and the use of the PSQI could be considered subjective compared to more objective markers for evaluating sleep. | P13-P14 |
|  | 23d | The clinical impact of this review is that its findings provide evidence for a positive association between PR and sleep quality in patients with COPD. Several recommendations for future randomised controlled trials appear to be justified based on the current findings. In particular, outstanding questions remain regarding what type of PR program is associated with the most significant improvements in sleep quality and whether certain types of patients benefit most from PR programs. | P13 |
| **OTHER INFORMATION** | | |  |
| Registration and protocol | 24a | The systematic review was registered on PROSPERO [CRD42023403543]. | P4 |
|  | 24b | The review protocol can be accessed on PROSPERO. | P4 |
|  | 24c | / |  |
| Support | 25 | No source of funding. | P14 |
| Competing interests | 26 | The authors declare they have no conflict of interest. | P14 |
| Availability of data, code and other materials | 27 | The collected data and tables can be seen in the manuscript, and the editors can contact the authors if they need additional data. | P16-P36 |

*From:*  Page MJ, McKenzie JE, Bossuyt PM, Boutron I, Hoffmann TC, Mulrow CD, et al. The PRISMA 2020 statement: an updated guideline for reporting systematic reviews. BMJ 2021;372:n71. doi: 10.1136/bmj.n71

For more information, visit: <http://www.prisma-statement.org/>
