# Supplementary material for: The impact of pulmonary rehabilitation on sleep quality in patients with chronic obstructive pulmonary disease: A systematic review and meta-analysis
Source: PLoS One. 2025 Jun 4;20(6):e0318424. doi: 10.1371/journal.pone.0318424 (PMC12136455; doi:10.1371/journal.pone.0318424)
Supplement: Attachment — Submitted filename: Response to Reviewers.docx [file pone.0318424.s008.docx]

23 May 2024

Dr. Alison Wang

Academic Editor

*PLOS One*

Dear Dr. Wang,

Please find enclosed our manuscript entitled “**The impact of pulmonary rehabilitation on sleep quality in patients with chronic obstructive pulmonary disease: A systematic review and meta-analysis”** for consideration for publication in *PLOS One*.

Firstly, may we thank the Reviewers for their careful appraisal our paper and their detailed points, to which we respond to below. We feel that the reviewer’s comments have significantly improved the quality of our manuscript. We have attempted to answer all reviewer’s comments fully.

We hope that we have addressed all reviewers’ comments sufficiently and hope that these changes will enable publication of our paper in your journal.

Signed on behalf of submitting authors,

Ms Shuiyan Dai and Dr Chun Shing Kwok, MBBS PhD MSc BSc MRCP

*Nurse and Senior Lecturer in Research Methodology and Cardiology Registrar*

**Reviewer #1: Thank you for inviting me to review this systematic review and meta-analysis which evaluate the impact of PR on sleep quality in COPD. The authors aligned with guidelines of the systematic review and register the review prior to conduct the study. The outcome indicated that PR is improving sleep quality in patients with COPD. I admire the effort here in conducting and writing the paper. However, there are some concerns need attention from the reviewer point of view.**

**- As author indicated the meta-analysis included RCT and non RCT (observational) studies. It is highly recommended by the Cochrane guidelines that RCT must be treated alone in the meta-analysis because the study designs are different. Pooled results must be presenting RCTs only.**

**Response:** We agree with the review that RCTs provide the highest quality of evidence which is superior to observational studies. However, trials are limited that they may not include real-world patients encountered in everyday practice. We do agree that it is important to highlight the findings from RCT and cohort studies so we have stratified the analysis. This is important as we feel that a review should include all the evidence not just those of higher quality so that we can fairly represent all the literature before making conclusions. The Figure 2 is now revised as follows:

In the methods, we have added the following:

“The main analysis which included studies that were of randomized controlled trial (RCT) and cohort in design was stratified by study design so that the pooled findings from RCT could be distinguished from cohort studies.”

In the results we have added the following:

“The benefit of PR was observed to a greater extent among studies that were of RCT design (mean difference 3.95 95% CI 0.36 to 7.55, I^2^=97%, 2 studies) compared to cohort studies (mean difference 0.92 95% CI 0.12 to 1.71, I^2^=0%, 4 studies).”

**- Heterogeneity checked via RevMan present 93%. That's fine. Then the author removed one study. Then, the heterogeneity dropped to 0%! I am impressed that one study can represent all the heterogeneity in the pooled analysis. It acceptable in the guidelines to deal with small to moderate heterogeneity in the analysis because studies are different. I suggest sperate the analysis for example do the pooled analysis for RCT only. Then, authors can present the pooled analysis for other studies if they can. However, authors admired in the limitations "there is significant methodology heterogeneity in the included studies as there is no consistent definition for PR in terms of time, activities, or periodicity of 300 implementation."**

**Response:** We have stratified the analysis by RCT and non-RCT and made the RCT subgroup analysis the primary outcome as shown above.

**- For better presenting in the result, present one study designed with pooled result for one outcome for example RCT and PSQI**

**Response:** We have stratified the analysis for RCT and non-RCT but kept the overall pooled results across all studies so the collective effect can be examined as well as within the subgroups.

**- Including results from conference abstract can be risky. I suggest focusing on the study that presents data (full text publications)**

**Response:** We included conference abstracts in this manuscript because inclusion of grey literature would reduce the risk of publication bias.

**- The keywords in the search strategy are not clear! Did the authors only searched these three terms. What bout Mesh terms? Any language filters?**

**Response:** We used a broad search using few terms in order to maximize sensitivity (including more results to reduce the risk of missing studies) as oppose to a narrow search with many search terms including Mesh terms (this strategy often results in fewer potential studies so that there are fewer studies to screen thus more specific). If the broad terms of sleep quality and pulmonary rehabilitation were used then it would also encompass the Mesh terms and therefore the Mesh search was not done. There were not language filters to reduce the chance of missing relevant studies.

**- Author mentioned "we planned to perform an asymmetry test to determine 113 whether there was publication bias" Can not find the funnel plot in the figures? Or the sensitivity analysis?**

**Response:** It is stated in our methods:

“If there were more than 10 studies in the meta-analysis and the statistical heterogeneity was less than 50%, we planned to perform an asymmetry test to determine whether there was publication bias.”

None of the pooled analyses had greater than 10 studies so asymmetry testing was not performed. The sensitivity analysis was performed to explore statistical heterogeneity. The results of the sensitivity analysis is stated as follows in the methods:

“However, the source of the heterogeneity was the study by Jin et al. If this study is removed the estimate would still be significantly different but the effect size would be smaller with no statistical heterogeneity (mean difference 0.91 95%CI 0.35-1.48, I^2^=0%, 5 studies).”

**- Quality assessment is not clear. Did the author used Ottawa-Newcastle Scale for all studies including the RCT? What about Cochrane risk of bias tool for RCTs?**

**Response:** In order to maintain consistency of the risk of bias assessment we used the Ottawa-Newcastle Scale. Many of the criteria used to assess quality such as ascertainment of exposure and outcome apply to both cohort studies and RCT. The consistency was important so that we can consider quality across all of the studies.

**- It would be better to present the data as systematic review and meta-analysis the main outcome PSQI.**

**Response:** We have added to the methods:

“The primary outcome of this study was the Pittsburgh Sleep Quality Index (PSQI) and the mean and standard deviation in the Pittsburgh Sleep Quality Index (PSQI) before and after PR were pooled.”

**- In the abstract as well as in the methods. It is random effect model not "Random effects meta-analysis."**

**Response:** Thank you for drawing our attention to this error. It is now corrected as follows in the abstract:

“Meta-analysis with the random effects model was performed to determine if PR is associated with any difference in the Pittsburgh Sleep Quality Index (PSQI).”

And in the methods:

“RevMan 5.4 (The Nordic Cochrane Centre, The Cochrane Collaboration, Copenhagen, Denmark) was employed in performing meta-analysis with the random effects model using the mean difference method.”

**Thank you.**

**Response:** Thank you for taking the time to review our manuscript.

**Reviewer #2: This is a creative topic, and the authors had done a good reviewing work. However, the manuscript should undergo major revision to meet the high standard basic requirements of the Journal. Here are some suggestions to further enhance the manuscript:**

**1. In the Introduction section, focus on the sleep quality issues among COPD patients and specifically detailing the positive and/or negative impacts of sleep quality on their quality of life.**

**Response:** We have revised the text in the introduction as follows:

“Sleep quality is poor in patients with severe COPD compared with normative populations of similar age, and daytime hypoxaemia is independently associated with impaired sleep efficiency. Approximately 70% of COPD patients typically complain of difficulty sleeping, two to three times more than the general population. One study suggests that 39% of patients with nocturnal cough or wheeze report difficulty initiating or maintaining sleep. Patients with COPD may experience anxiety, depression, shortness of breath and coughing, sputum, and hypoxaemia during the night. There is also some evidence to suggest that patients with COPD may be affected by an overlap with obstructive sleep apnoea as the prevalence of obstructive sleep apnoea ranges from 10 to 65%. There appears to be a bidirectional relationship between sleep quality and clinical outcomes, with sleep disturbances leading to systemic inflammation, immune impairment, lack of exercise and cognitive changes, which may affect medication adherence and lead to poor clinical outcomes, nocturnal COPD symptoms, and reduced physical activity. Sleep disturbance in patients with COPD likely contributes to the non-specific daytime symptoms of chronic fatigue, lethargy and overall impairment in quality of life.

**2. In the introduction section, clarify the association of PR and sleep quality in COPD patients or the potential mechanism of PR on sleep quality. More details need to be specified on the knowledge gaps.**

**Response:** Thank you for the suggestion. The information on mechanism for PR and sleep quality is in the discussion currently because we feel that the introduction text is long enough. If the reviewer feels that it is better placed there then we can move it there but the introduction will become long. The text in the discussion is written as follows:

“PR is based on a comprehensive, multidisciplinary, patient-centred interventions, including exercise training, self-management, education and psychosocial support, relieves dyspnoea and fatigue, improves emotional functioning, increases self-awareness of the disease, and contributes to improved health-related quality of life and exercise capacity. For physiological reasons, PR further improves respiratory muscle strength by increasing aerobic enzymes to relax the muscles. This improvement has been correlated with a reduction in dynamic hyperinflation and improved ventilation in COPD patients. Moreover, PR also helps to improve systemic inflammation. Pinho et al concluded that patients with COPD are characterized by increased systemic and pulmonary oxidative stress and that PR is related to a reduction in oxidative stress. Exercise training is also recommended to improve sleep quality by increasing energy expenditure, endorphin secretion and body temperature, thus promoting sleep to restore the body. Physical health is said to be associated with sleep quality and greater improvements in health status following exercise training are associated with better sleep outcomes. Psychologically, as mentioned above anxiety and depression can contribute to poor sleep quality in people with COPD and there is a high prevalence of depression and anxiety in COPD. PR led to better psychological status on the Anxiety-Depression Scale in patients with COPD. Therefore, exercise training promotes sleep through its anxiolytic or antidepressant effects.”

**3. In the Methods section, clarify the PICO principle and list the inclusion criteria and exclusion criteria more specifically.**

**Response:** We have placed the inclusion criteria within the PICO framework as suggested as follows:

“The participants were patients with a diagnosis of COPD. The intervention is pulmonary rehabilitation and the comparator is the same patient prior to taking part in pulmonary rehabilitation. The outcome is sleep quality.”

**4. Clarify the reason of the PSQI as the primary outcomes on sleep quality in the methods section.**

**Response:** We have added the following text in the methods manuscript:

“The PSQI was chosen because it is the most commonly used generic measure of sleep quality in clinical and research settings.”

**5. Clarify the actions if the two reviewers (SD and CSK) have a disagreement while engaging in study selection, data extraction and quality assessment?**

**Response:** We have added the following text in the methods manuscript:

“Where there were disagreements in study selection, data extraction and quality assessment between the two reviewers, the third reviewer (SS) was consulted and a decision was made by consensus.”

**6. Clarify the definition/criteria of each study quality for the NOS, e.g., NOS scores less than or equal to 4 points as low quality? Or how to identify the overall quality of 6.9 scores as the low or moderate or high quality.**

**Response:** The NOS score was present for each study which has an integer value from 0 to 9 where lower quality studies are represented by lower values while higher values represent higher quality studies. We describe the overall quality of the studies as the average score across all included studies.

**7. In the data synthesis, how about the subgroup analysis. Specify the reason of using the random-effects meta-analysis.**

**Response:** We have performed subgroup analysis according to study design. This is shown below. The random effects meta-analysis was used because we could not be certain that there is homogeneity among the methodology of the different studies which would support fixed-effects meta-analysis.

**8. In the results section, the contents of the PRISMA flow diagram were not completed. Please use the latest PRISMA 2020 flow diagram and provide sufficient information of the literature screening process.**

**Response:** The flow diagram is shown below based on the PRISMA 2020 flow diagram. We were able to retrieve all studies that were potentially relevant in the screening process so the box for studies which were not retrieved was not included.

**9. The APA suggest " p value" The p is lowercase and italicized. Please re-check the manuscript.**

**Response:** We have revised as suggested.

**10. In the discussion, use a cautious or tentative tone when describing or reporting the interpretation and explanation of the results.**

**Response:** We have revised the text as suggested as follows:

“Firstly, there is some evidence to suggest that PR has a beneficial effect on sleep quality in COPD patients as defined by the PSQI.”

“Overall, these findings suggest that PR may be associated with improvements in sleep quality in patients with COPD and PR may be recommended for patients who feel that their sleep quality requires improvement.”

We would welcome any additional specific changes the reviewer can suggest.

**11. Lacking note on abbreviation in the Table 2 and Table 3.**

**Response:** We have explained the abbreviations as follows:

Table 2

COPD=chronic obstructive pulmonary disease, PSQI=Pittsburgh Sleep Quality Index, PR=pulmonary rehabilitation, MDT=multidisciplinary team

Table 3

PSQI=Pittsburgh Sleep Quality Index, PR=pulmonary rehabilitation, COPD=chronic obstructive pulmonary disease

**12. Reformat the reference style.**

**Response:** We have revised as suggested.

**13. The manuscript should undergo careful language editing for spelling, grammar errors and be re-checked for any errors to meet the high standard basic requirements of the Journal.**

**Response:** We have checked and would welcome any suggestions on improving the text of the manuscript.
