# Supplementary material for: The impact of pulmonary rehabilitation on sleep quality in patients with chronic obstructive pulmonary disease: A systematic review and meta-analysis
Source: PLoS One. 2025 Jun 4;20(6):e0318424. doi: 10.1371/journal.pone.0318424 (PMC12136455; doi:10.1371/journal.pone.0318424)
Supplement: Attachment — Submitted filename: Response_to_Reviewers_auresp_2.docx [file pone.0318424.s009.docx]

14 September 2024

Dr. Alison Wang

Academic Editor

*PLOS One*

Dear Dr. Wang,

Please find enclosed our manuscript entitled “**The impact of pulmonary rehabilitation on sleep quality in patients with chronic obstructive pulmonary disease: A systematic review and meta-analysis”** for consideration for publication in *PLOS One*.

Firstly, may we thank the Reviewers for their careful appraisal our paper and their detailed points, to which we respond to below. We feel that the reviewer’s comments have significantly improved the quality of our manuscript. We have attempted to answer all reviewer’s comments fully.

We hope that we have addressed all reviewers’ comments sufficiently and hope that these changes will enable publication of our paper in your journal.

Signed on behalf of submitting authors,

Ms Shuiyan Dai and Dr Chun Shing Kwok, MBBS PhD MSc BSc MRCP

*Nurse and Senior Lecturer in Research Methodology and Consultant Cardiologist*

Reviewer's Responses to Questions

**Comments to the Author**

**1. If the authors have adequately addressed your comments raised in a previous round of review and you feel that this manuscript is now acceptable for publication, you may indicate that here to bypass the "Comments to the Author" section, enter your conflict of interest statement in the "Confidential to Editor" section, and submit your "Accept" recommendation.**

**Reviewer #2: (No Response)**

**Reviewer #3: (No Response)**

**Response:** We hope the following response are satisfactory to address your comments.

**2. Is the manuscript technically sound, and do the data support the conclusions?

The manuscript must describe a technically sound piece of scientific research with data that supports the conclusions. Experiments must have been conducted rigorously, with appropriate controls, replication, and sample sizes. The conclusions must be drawn appropriately based on the data presented.**

**Reviewer #2: Partly**

**Reviewer #3: Yes**

**Response:** We hope the following response are satisfactory to address your comments.

**3. Has the statistical analysis been performed appropriately and rigorously?**

**Reviewer #2: Yes**

**Reviewer #3: Yes**

**Response:** Thank you.

**4. Have the authors made all data underlying the findings in their manuscript fully available?

The** [**PLOS Data policy**](http://c05y1x9s.r.us-east-2.awstrack.me/L0/http:%2F%2Fwww.plosone.org%2Fstatic%2Fpolicies.action%23sharing/1/010f0191a01ba329-8202a29d-1561-4677-baf3-61714edc9748-000000/oHHOc0PrHRVjKEG5FmtUZyXBZvg=174) **requires authors to make all data underlying the findings described in their manuscript fully available without restriction, with rare exception (please refer to the Data Availability Statement in the manuscript PDF file). The data should be provided as part of the manuscript or its supporting information, or deposited to a public repository. For example, in addition to summary statistics, the data points behind means, medians and variance measures should be available. If there are restrictions on publicly sharing data—e.g. participant privacy or use of data from a third party—those must be specified.**

**Reviewer #2: No**

**Reviewer #3: Yes**

**Response:** There is no original data in this manuscript as it is a systematic review. All the data is available from original sources and we do not have permission to make the fully available other than seeking them directly from authors and journals.

**5. Is the manuscript presented in an intelligible fashion and written in standard English?

PLOS ONE does not copyedit accepted manuscripts, so the language in submitted articles must be clear, correct, and unambiguous. Any typographical or grammatical errors should be corrected at revision, so please note any specific errors here.**

**Reviewer #2: Yes**

**Reviewer #3: Yes**

**Response:** Thank you.

**6. Review Comments to the Author

Please use the space provided to explain your answers to the questions above. You may also include additional comments for the author, including concerns about dual publication, research ethics, or publication ethics. (Please upload your review as an attachment if it exceeds 20,000 characters)**

**Reviewer #2: I admire the effort here in conducting such work. However, there are still some concerns were not solved clearly and need further attention from the reviewer point.

1.The definition/description of the Comparator(s)/control in your paper was misunderstanding and did not keep consistent with your registered protocol.**

**Response:** We do not mention comparator or control group so there is no misunderstanding or inconsistency as there is no restriction based on comparator group. We have revised the text as follows:

“Studies were included if they evaluated sleep quality in people with COPD who underwent pulmonary rehabilitation and there was no restriction based on the presence or type of comparator group.”

**2. For the search strategy, I recommend to follow the JBI methodology and PRISMA reporting guideline using the appropriate search strategy. Only these three terms didn't mean a broader search. How about the articles that may use different words (e.g., synonyms and variations) to describe the same concept. To ensure the sensitivity and accuracy during the retrieval, also the broader search, that is why we need a combination of mesh terms and free/key words, along with the use of appropriate search queries. This can enhance efficiency, accuracy, and comprehensiveness of searches in medical and scientific literature databases. Additionally, describe at least one database that utilizes a search strategy enabling others to replicate your retrieval results.**

**Response:** We understand the approach that many organizations promote but the senior author who has extensive research experience who has used this approach before in a Cochrane review does not agree with this approach. We believe that in the current work additional terms will not add anything that is not captured in the parent term. The current approach was what was registered with PROSPERO and we should not deviate from it.

However, if the reviewer insists on having a new search, please suggest a strategy and we will try to accommodate it.

**3. In the section of Study inclusion and exclusion criteria, it's said "Studies had to have original data which meant that letters, editorials, comments, and reviews were excluded." However, this paper included the abstracts WITHOUT original data. If the abstracts were included as you explained "the grey literature would reduce the risk of publication bias " in the response letter, then you have to search the grey literature databases to minimize the publication bias as much as possible, but you did not. These statements are mutually contradictory.**

**Response:** I think there is misunderstanding about what original data is. Abstracts contain original data as they may report studies which collect data from patients. Their reporting may not be as complete as full manuscripts and they may not undergo the same level of peer review. If we excluded abstracts which may contain less publishable results then there may be publication bias and inclusion of this grey literature is important. Therefore, the statements are not mutually contradictory. We have added the following to clarify:

“Studies had to have original data which could be in full manuscript or abstract form so letters, editorials, comments, and reviews without original data were excluded.”

**4. The Newcastle Ottawa Scale (NOS) recommended by the Cochrane Collaboration was used as a quality assessment scale for case-control and cohort studies but NOT for randomized studies. Using the Newcastle Ottawa Scale (NOS) for RCT's quality assessment seems inappropriate, iI suggest to use the specific quality assessment tools for RCT.**

**Response:** We agree with the reviewer that the NOS is for observational studies and not designed for RCT but we used a single risk assessment for consistency. However we have now revised the risk assessment for trials to one recommended by Cochrane. Results are on the next page and we have added the following test in the methods and results:

“Study quality assessments for observational studies were carried out using the Ottawa-Newcastle Scale for observational studies [25].”

“The study quality assessment for randomised trials was performed using the Cochrane Risk of Bias Tool [26]. The domains assessed were bias from randomization process, bias due to deviations from intended intervention, bias in measurement of the outcome, and bias in selection of the reported result.”

“Among the five randomized controlled trials included in the review, two studies were classified as low risk for all five domains of the Cochrane risk of bias tool.”

**Table 3: Study Quality Assessment by Cochrane Risk of Bias Tool for Randomised trials**

| Study ID | Bias from randomization process | Bias due to deviations from intended interventions |  | Bias due to missing outcome data |  | Bias in measurement of the outcome |  | Bias in selection of the reported result |
| --- | --- | --- | --- | --- | --- | --- | --- | --- |
| Benzo 2022 | Some concern, randomized in a 1:1 ratio on the basis of a pregenerated sequence of assignments  through a computer-generated permuted  block randomization with blocks of size four, allocation concealment unclear. | Low risk, 12-week program with weekly healthcare calls and a remote monitoring system, Garmin Vivofit activity monitor and oximeter vs usual care and educational packet  for weekly self-study. | | Low risk, participants in both arms did not complete measures at 3 months, lost contact and refused participation. Some patient died and became ineligible in usual care group. | | Some concern, assessors were not blinded to intervention received. | | Low risk, trial analysed according with pre-specified plan. |
| Cox 2019 | Low risk, randomisation undertaken using a computer-generated sequence and allocation will be concealed using sealed, opaque envelopes. | Low risk, home versus hospital-based rehabilitation program. | | Low risk, no missing outcome data reported. | | Low risk, Objective measures of sleep quality were obtained from the SenseWear Armband. | | Low risk, trial analysed according with pre-specified plan although this was post-hoc analysis. |
| Jin 2021 | Some concern, random number table used for randomisation but allocation concealment unclear. | Low risk, pulmonary rehabilitation training three times a week in addition to conventional drug treatment vs drug treatment alone. | | Low risk, no missing outcome data reported. | | Some concern, participants and assessors were not blinded. | | Low risk, trial analysed according with pre-specified plan. |
| Spielmanns 2022 | Low risk, randomisation using a software randomizer and fabrication of lists done by the sponsor for allocation concealment. | Low risk, KAIA COPD app program vs usual care. | | Low risk, only 6 out of 67 withdrew informed consent or discontinued by investigators. | | Low risk, electronic patient-reported outcomes and activity and sleep measured passively and continuously for each participant by the activity tracker. | | Low risk, trial analysed according with pre-specified plan. |
| Yu 2022 | Some concern, randomization by random number table but allocation concealment unclear. | Low risk, mindfulness behavioural intervention combined with progressive breathing training vs symptomatic treatment during hospitalization and routine nursing measures in internal medicine. | | Low risk, no missing outcome data reported. | | Some concern, participants and assessors were not blinded. | | Low risk, trial analysed according with pre-specified plan. |

**5. Refer to the results of quality assessment, we did not see how many papers were identified as high or low or moderate quality. As the quality of the included studies would impact the robustness of conclusions. There is indeed a cut-off score of NOS.**

**Response:** The cutoffs for number of stars are arbitrary as there are 9 possible stars and it should sort of depend on the research question and the extent to which study designs can best answer the question. In this case we can assign high quality of 7-9 stars, 4-6 stars as moderate quality and 0-3 stars as low quality. We have added the following:

“Among the eleven observational studies, six had seven out of nine stars and would be considered as high quality compared to the other five studies which had six out of nine stars based on the Ottawa-Newcastle Scale.”

**Reviewer #3: (No Response)**

**Response:** Thank you.
